# Supplementary material for: Brønsted acid-catalysed enantioselective construction of axially chiral arylquinazolinones
Source: Nat Commun. 2017 May 19;8:15489. doi: 10.1038/ncomms15489 (PMC5454535; doi:10.1038/ncomms15489)
Supplement: Supplementary Information — Supplementary figures, supplementary tables, supplementary notes and supplementary references. [file ncomms15489-s1.pdf]

# Supplementary Figures

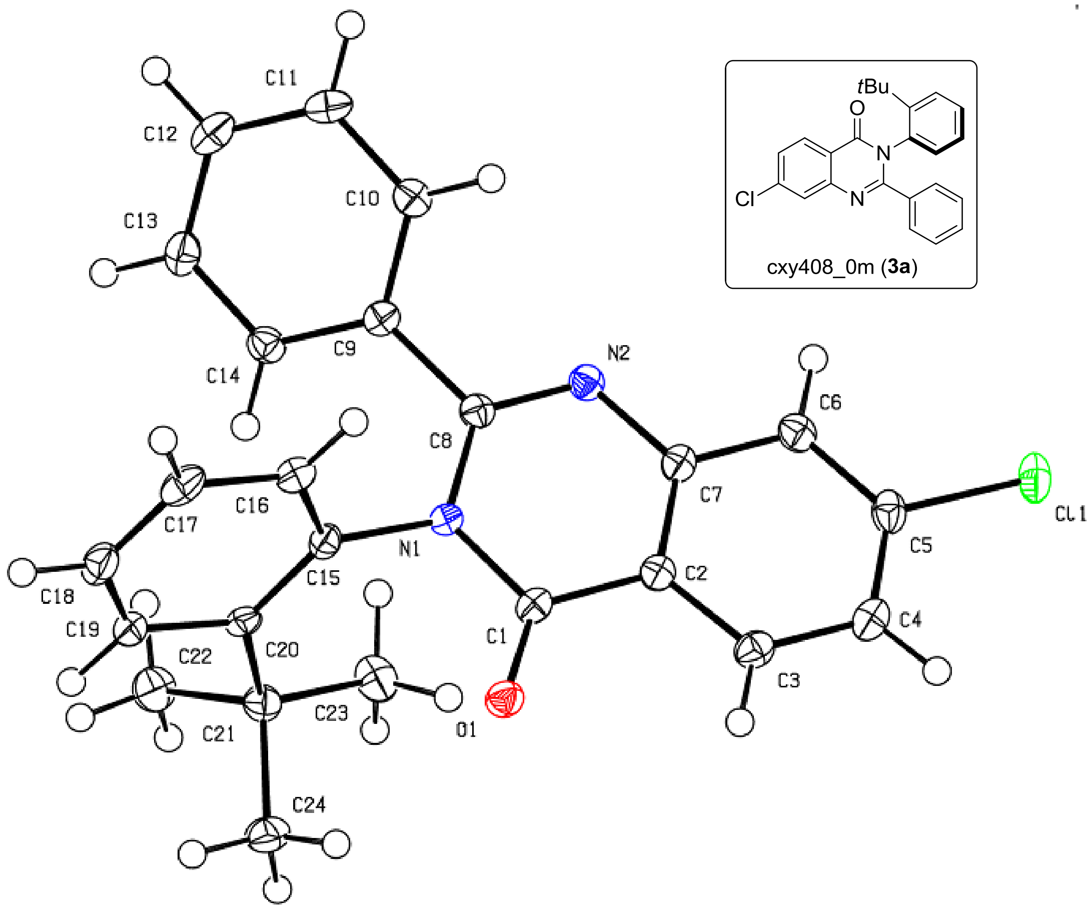


Supplementary Figure 1. X-ray crystal structure of 3a.

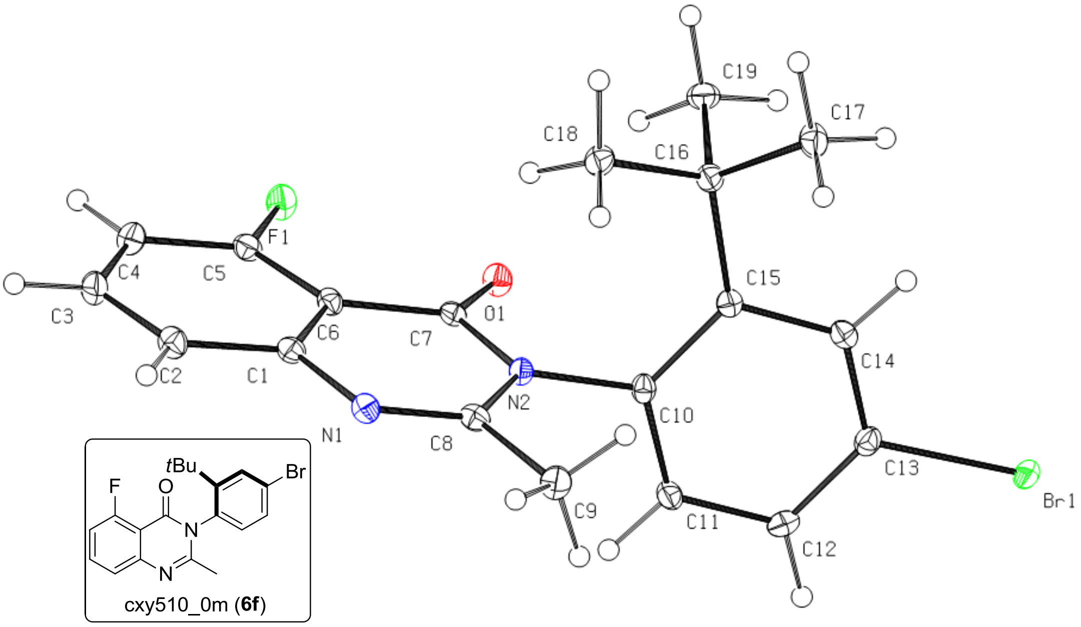


**Supplementary Figure 2. X-ray crystal structure of 6f.**

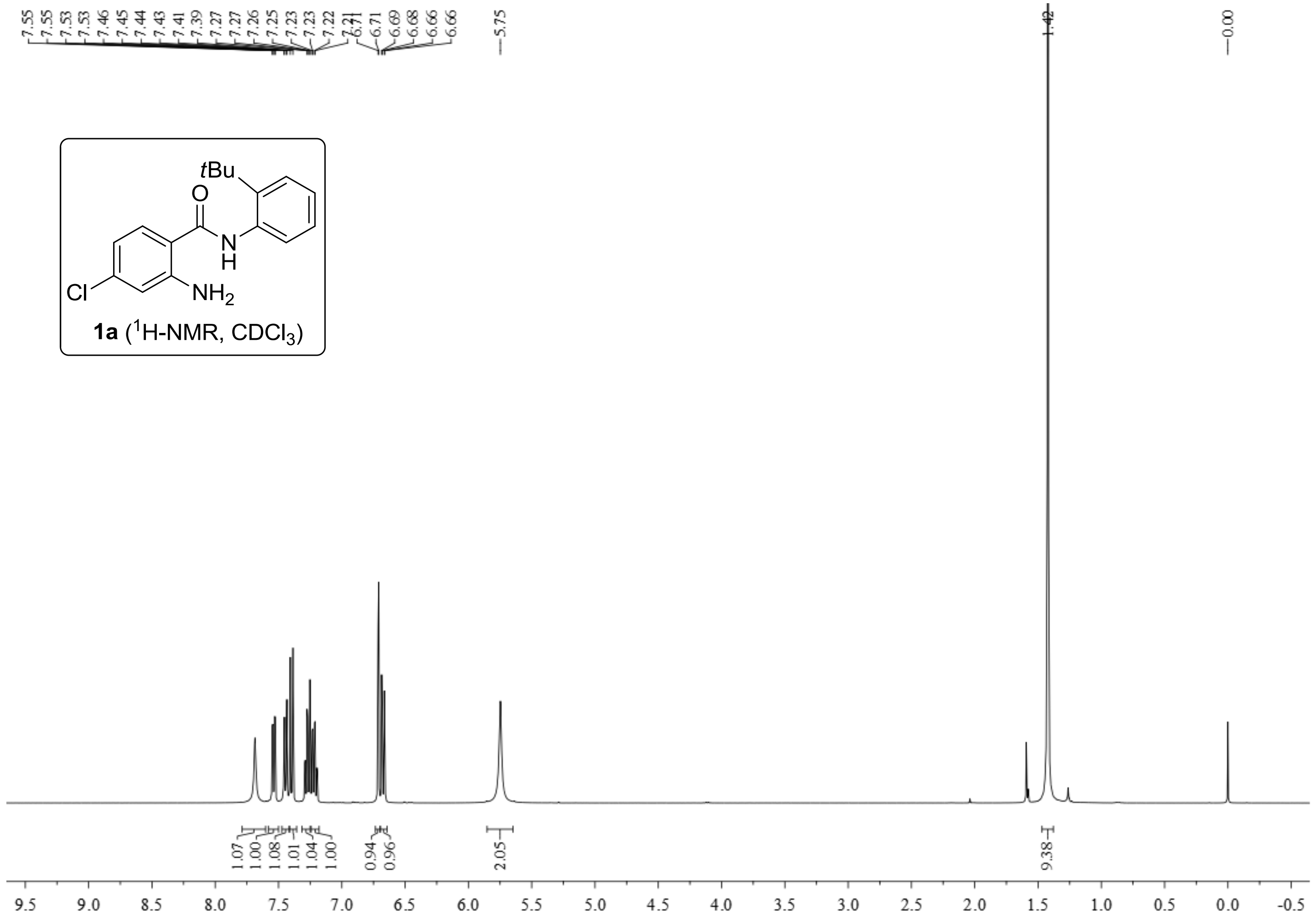


**Supplementary Figure 3.** $^{1}$H NMR of **1a**

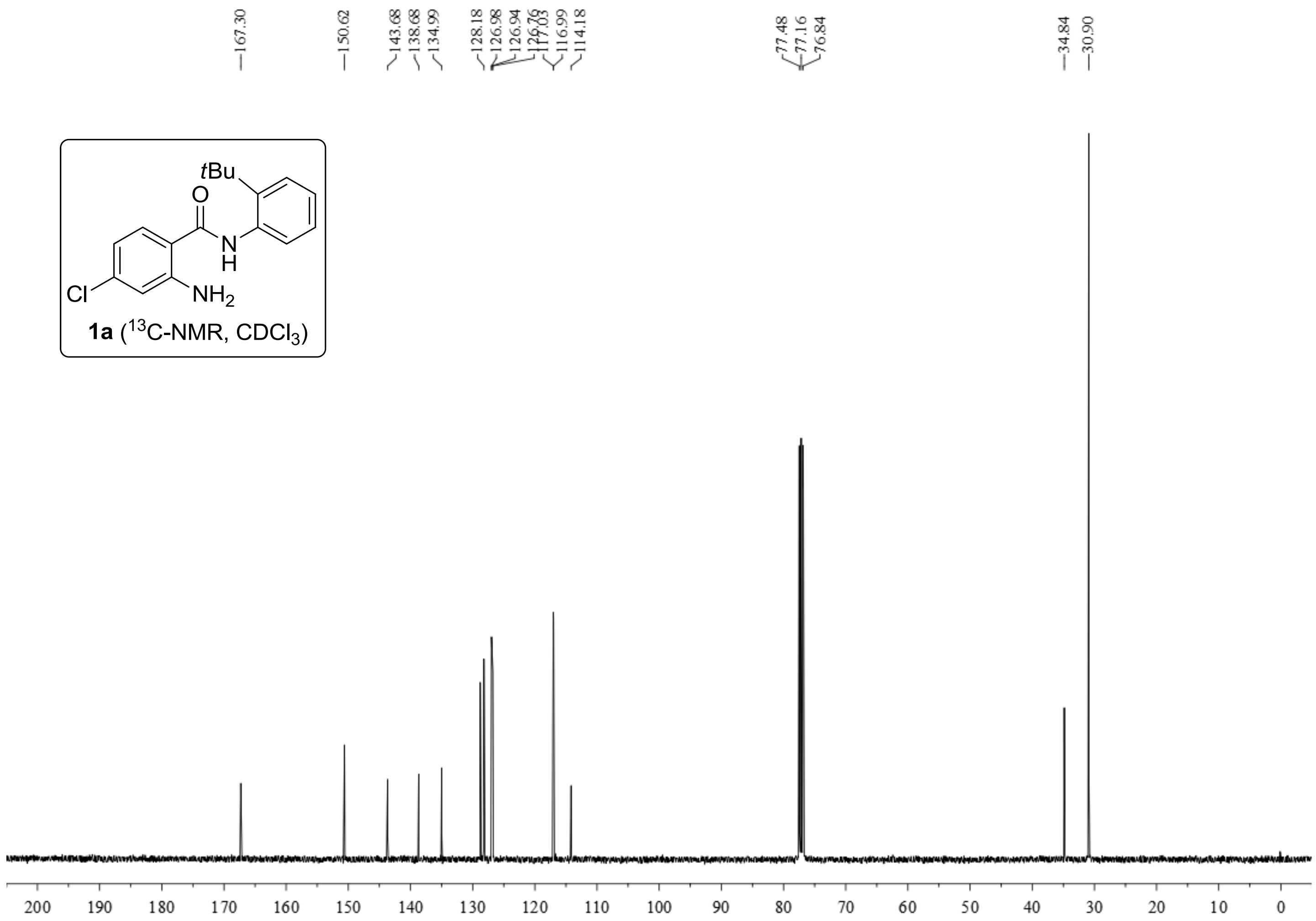


**Supplementary Figure 4.** $^{13}$C NMR of **1a**

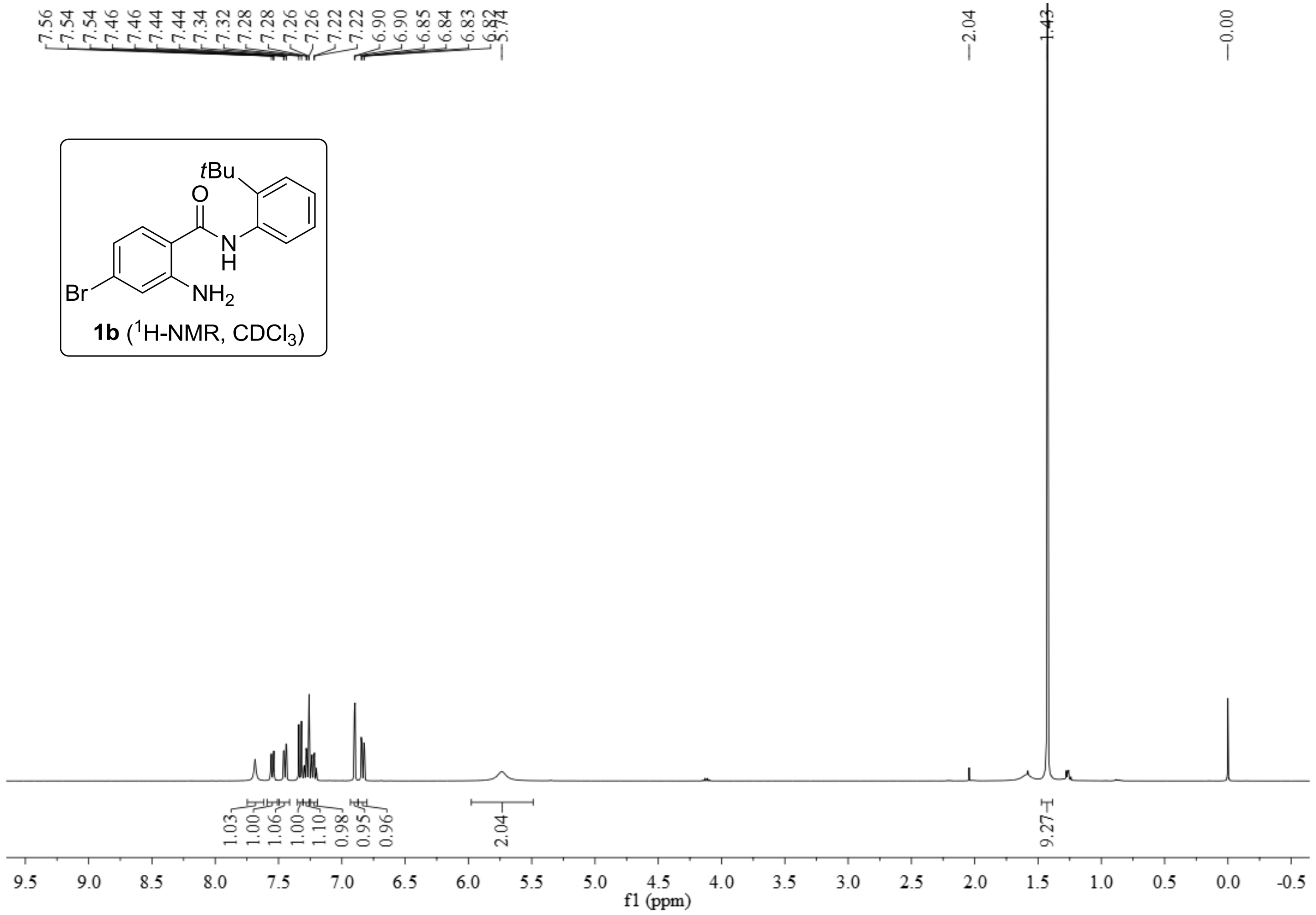


**Supplementary Figure 5.** $^{1}$H NMR of **1b**

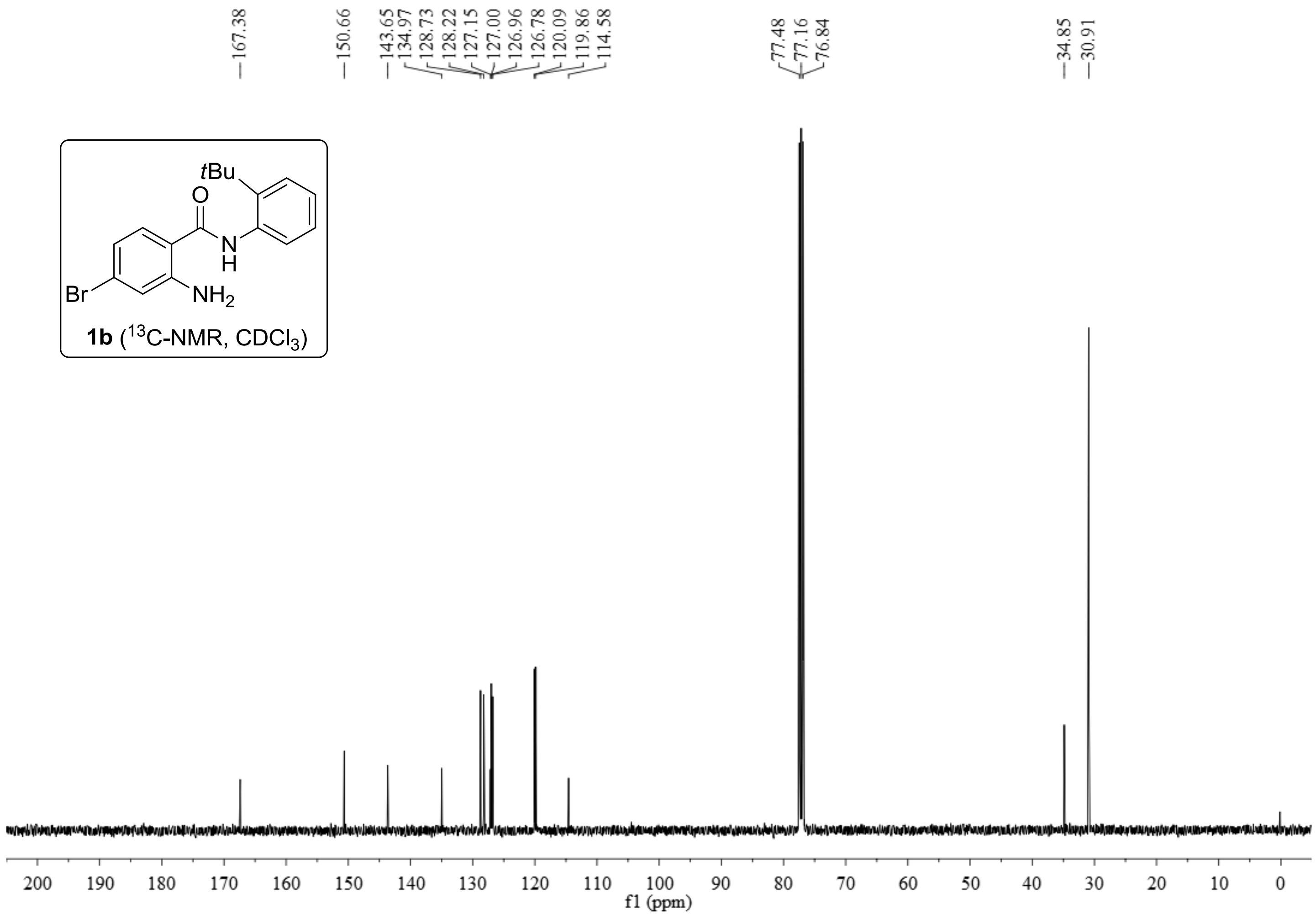


**Supplementary Figure 6.** $^{13}$C NMR of **1b**

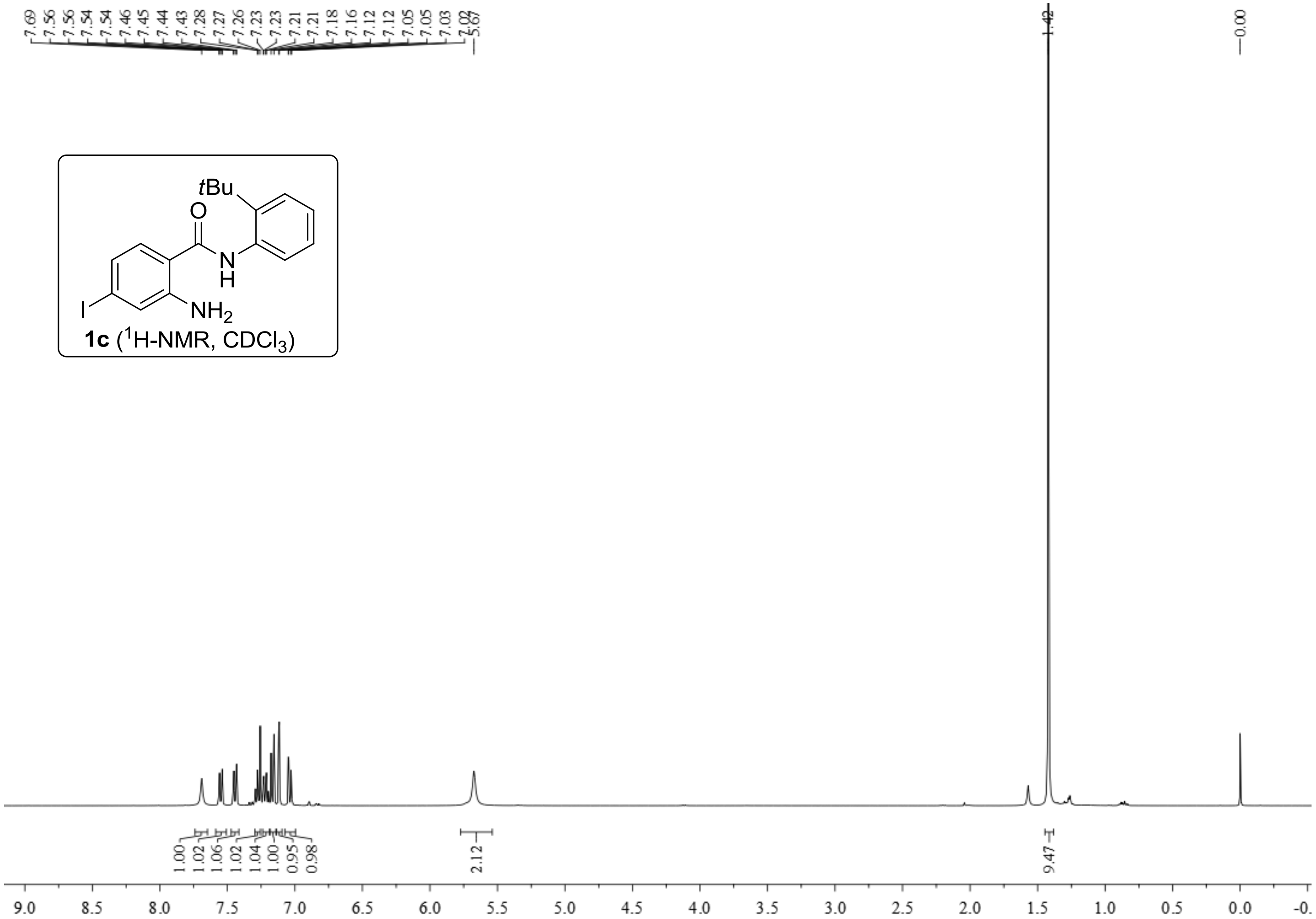


**Supplementary Figure 7.** $^{1}$H NMR of **1c**

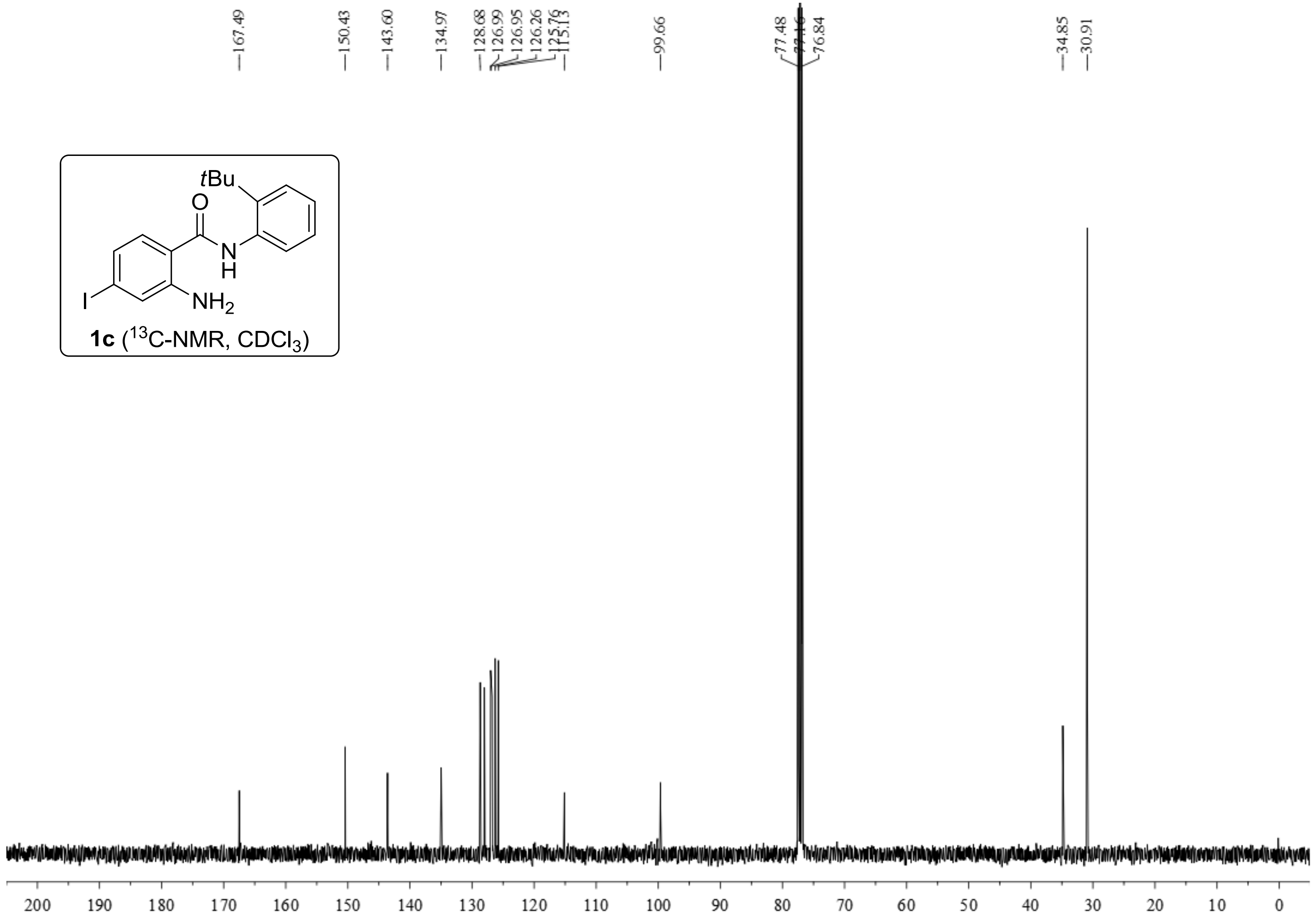


**Supplementary Figure 8.** $^{13}$C NMR of **1c**

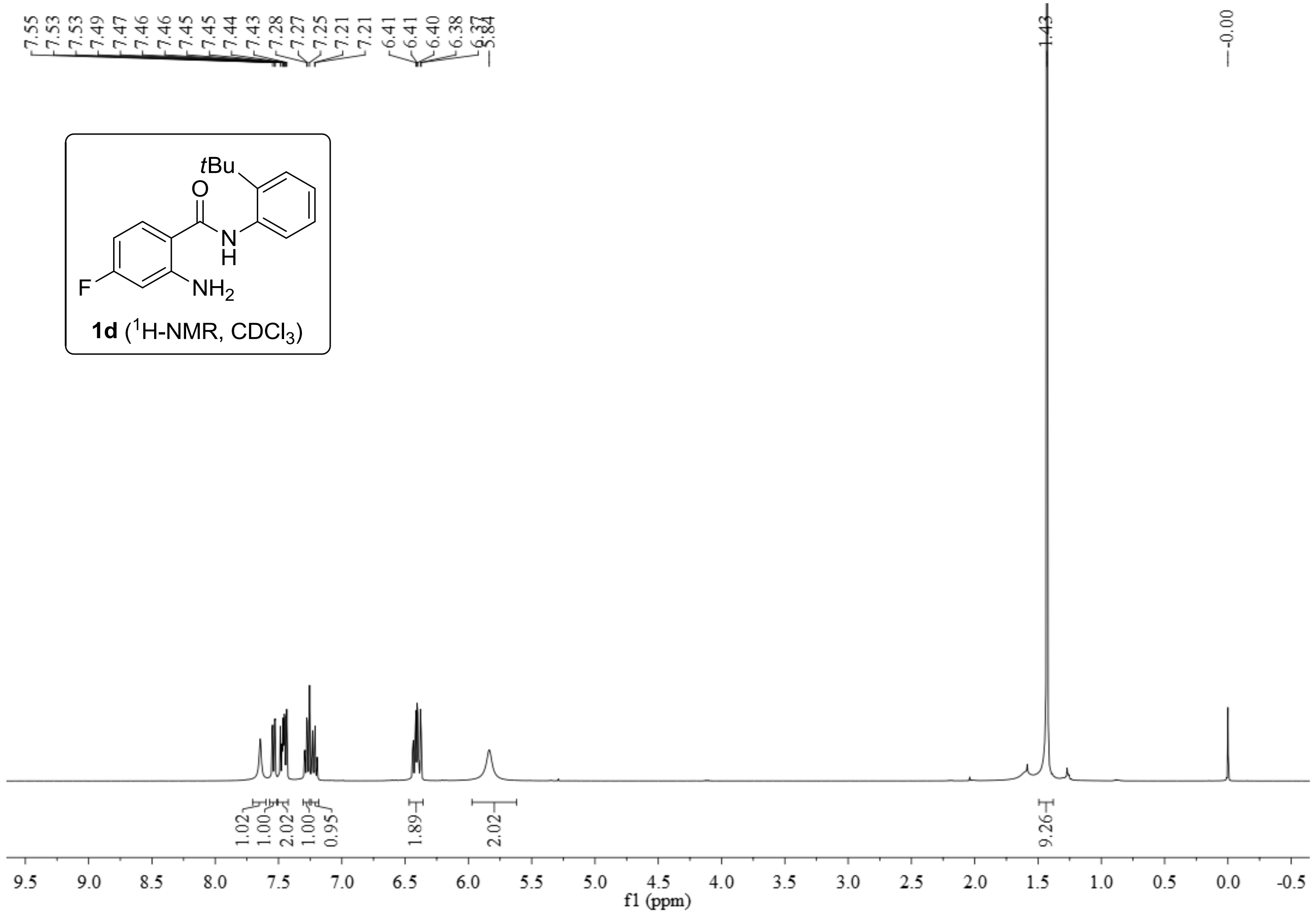


**Supplementary Figure 9.** $^{1}$H NMR of **1d**

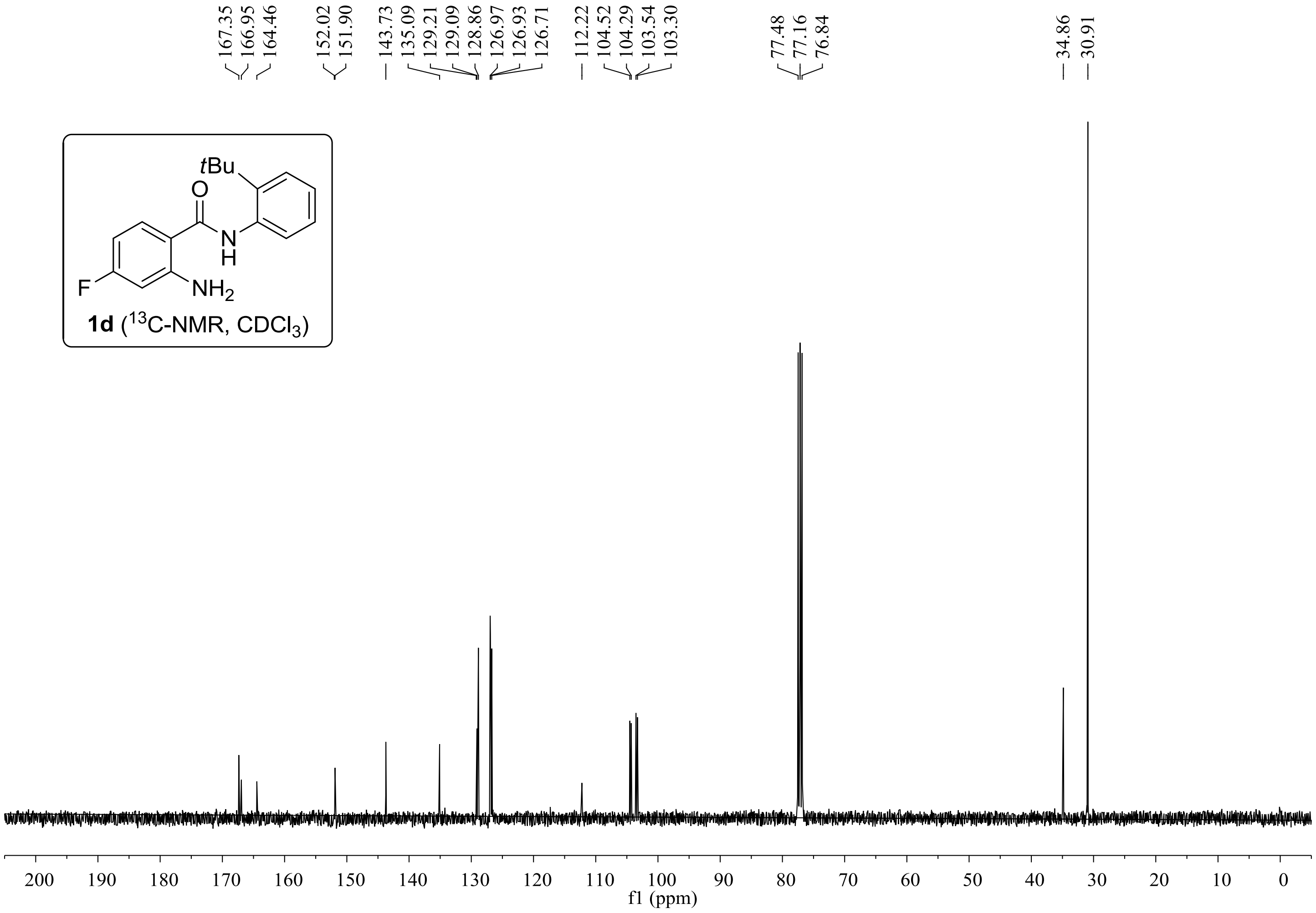


**Supplementary Figure 10.** $^{13}$C NMR of **1d**

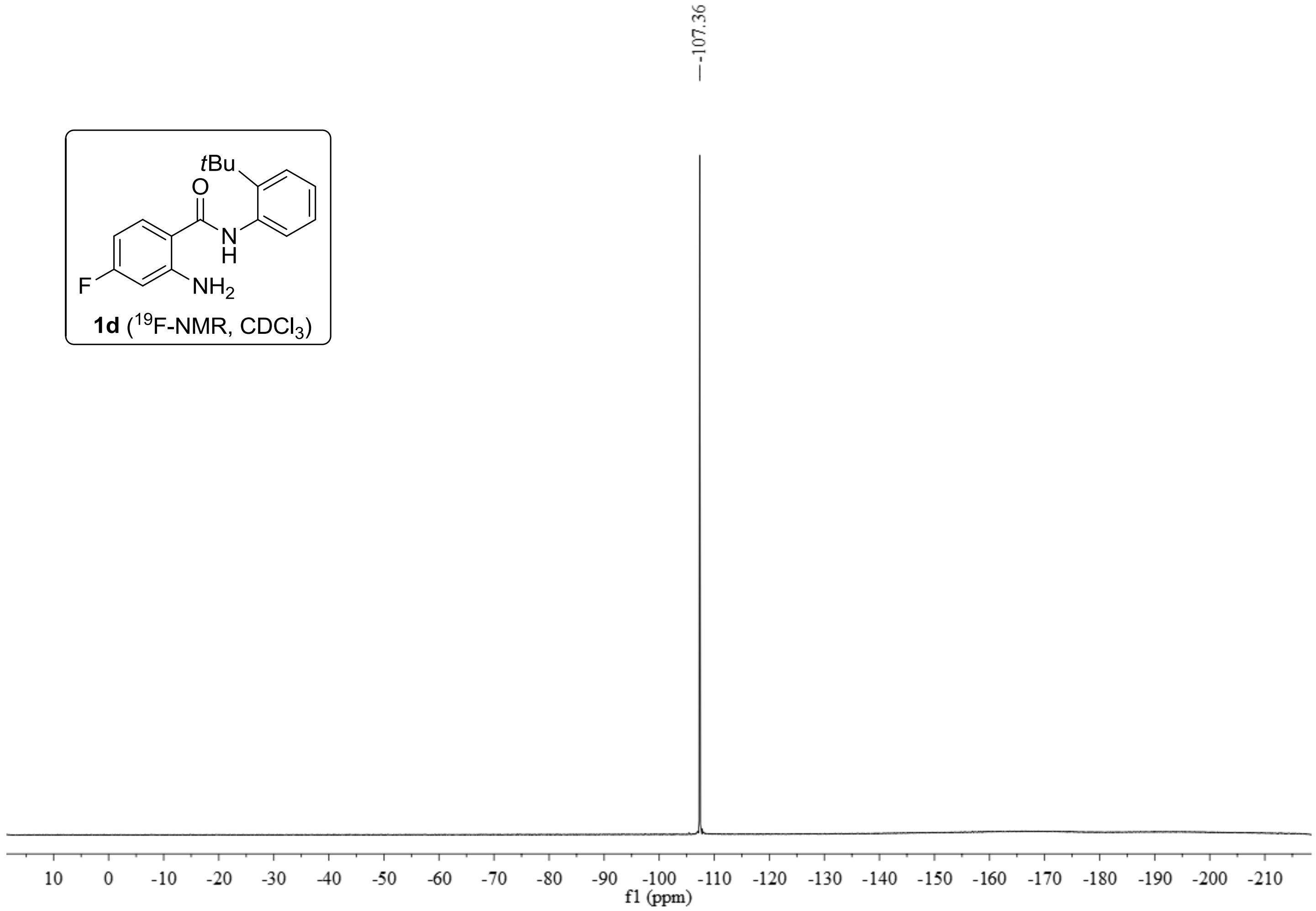


**Supplementary Figure 11.** $^{19}F$ NMR of **1d**

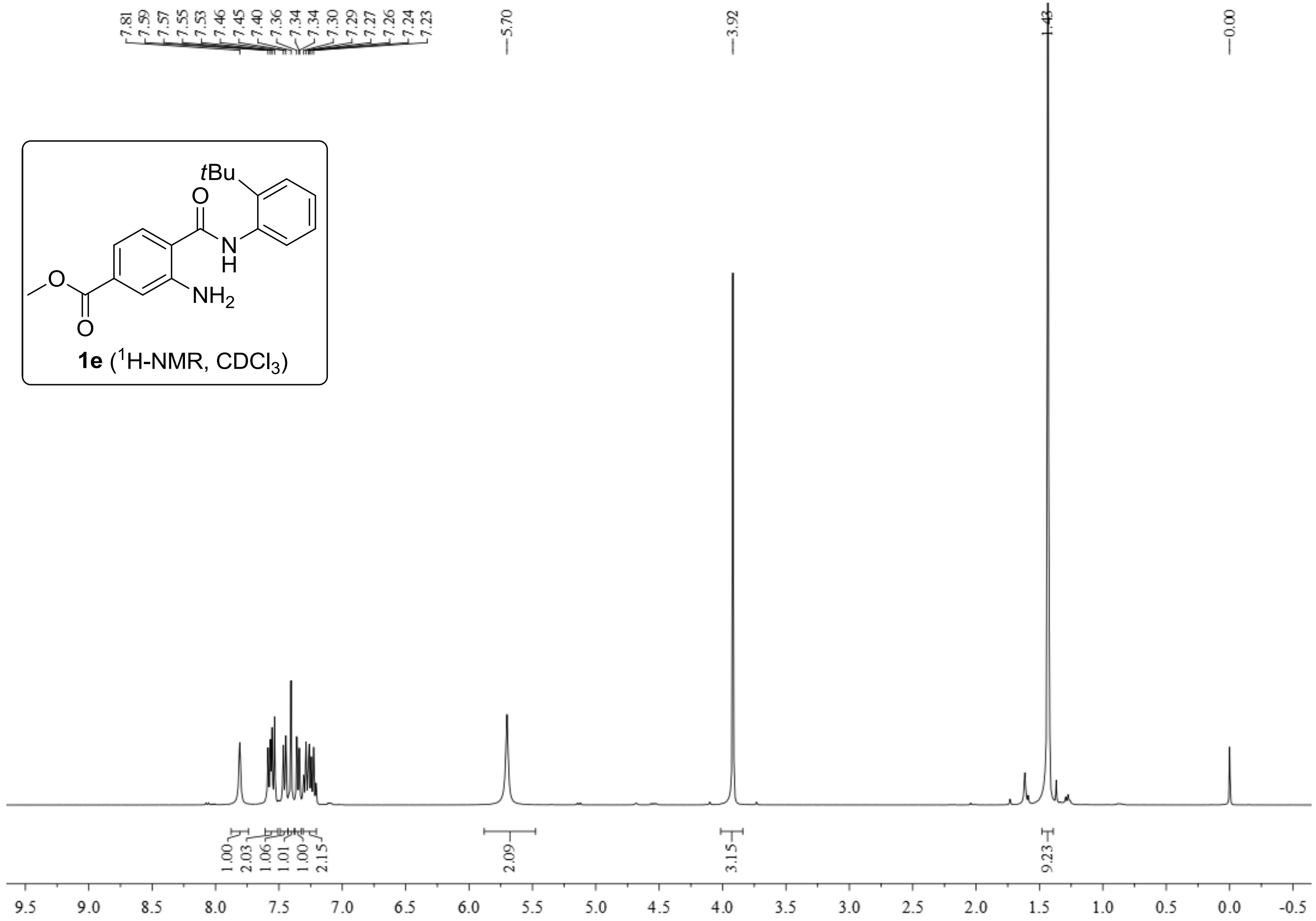


**Supplementary Figure 12.** $^1$H NMR of **1e**

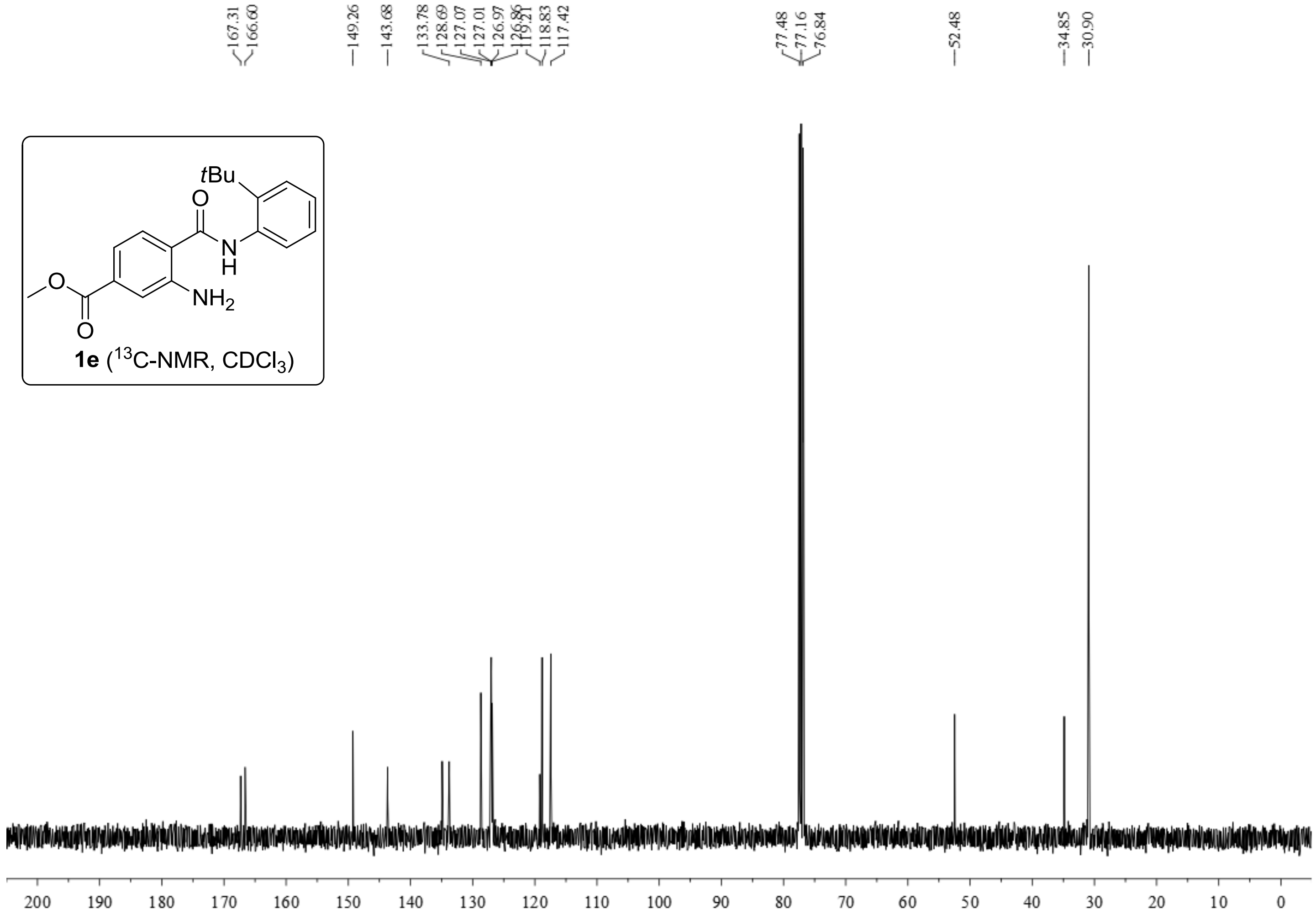


**Supplementary Figure 13.** $^{13}$C NMR of **1e**

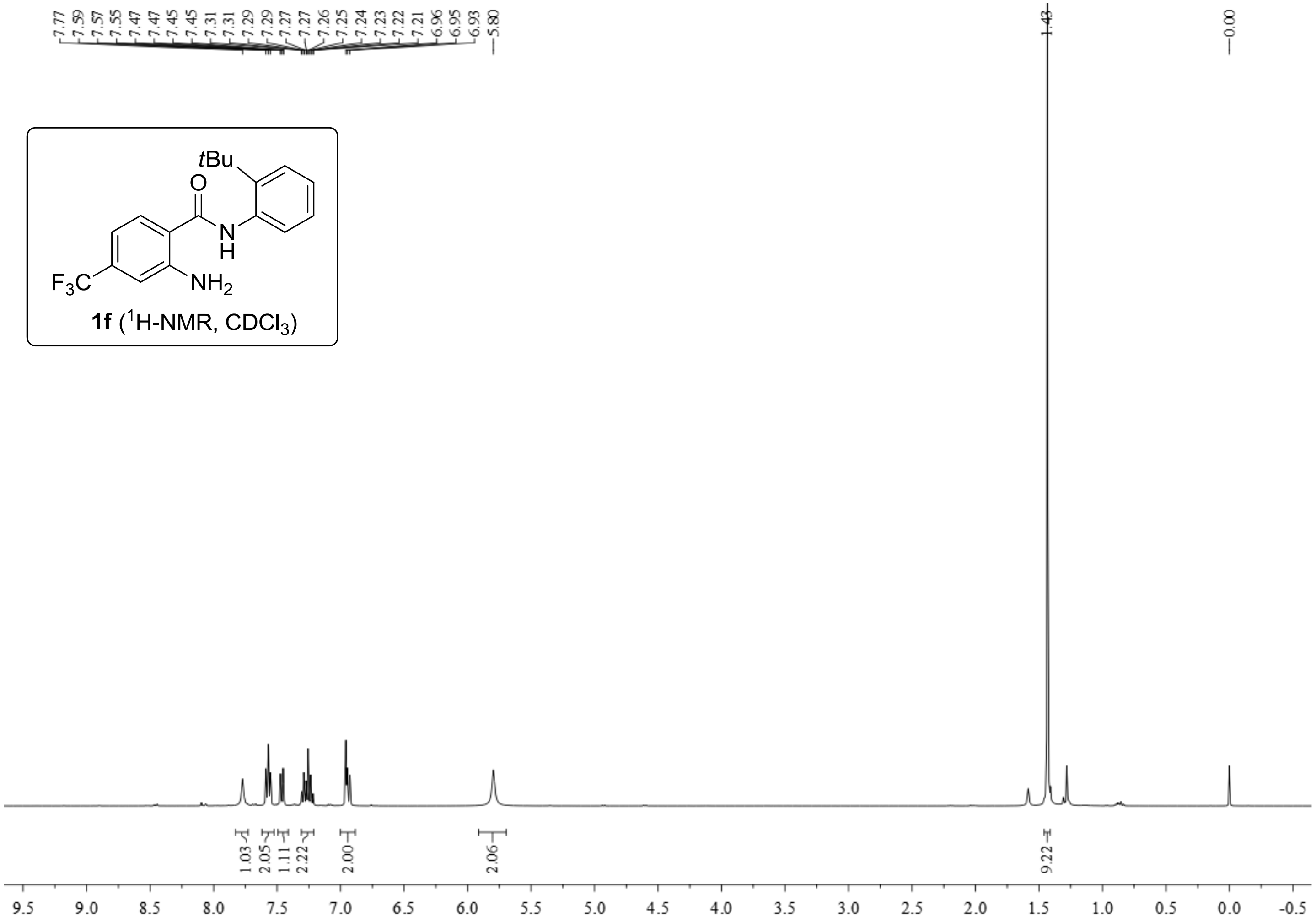


**Supplementary Figure 14.** $^{1}$H NMR of **1f**

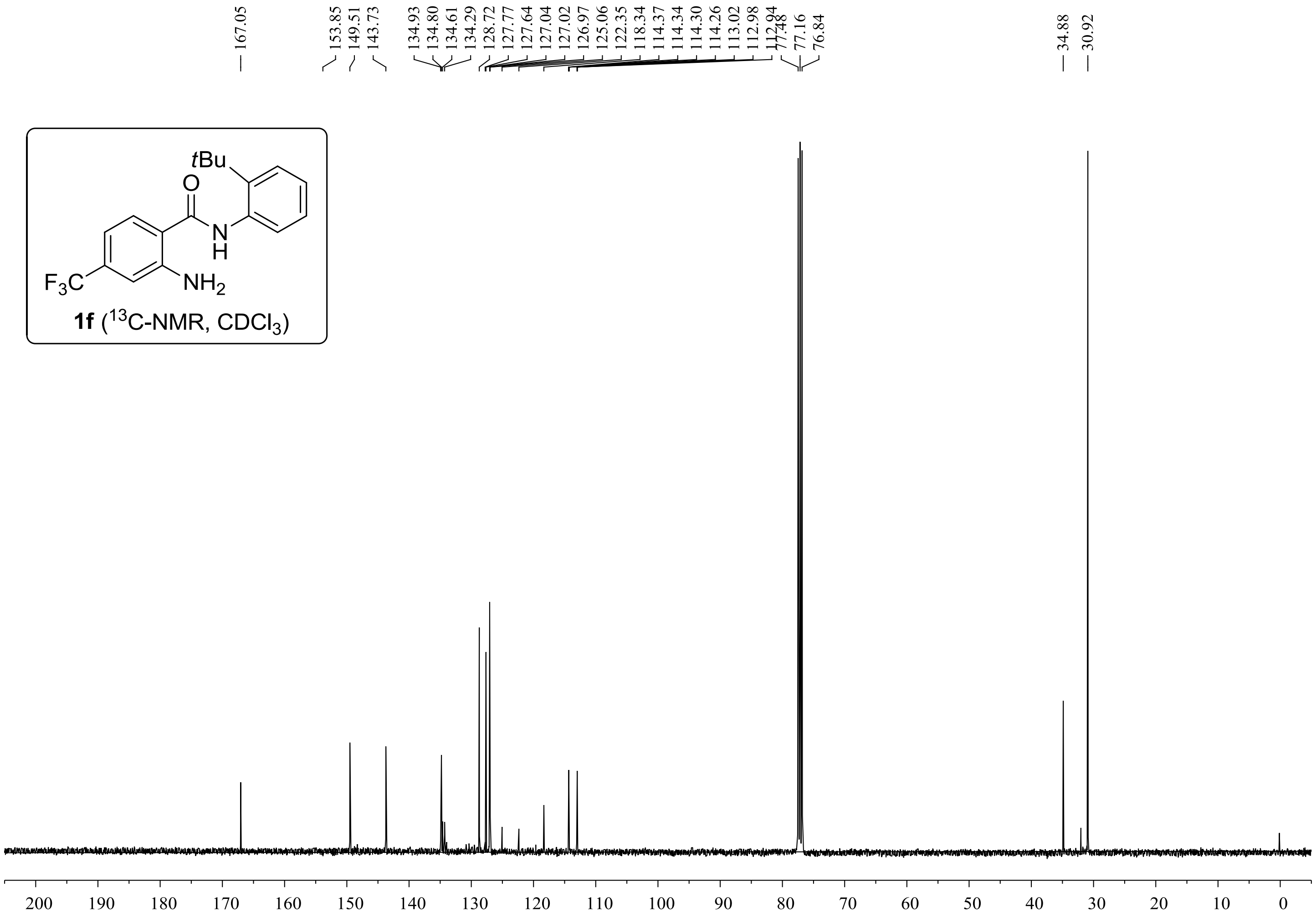


**Supplementary Figure 15.** $^{13}$C NMR of **1f**

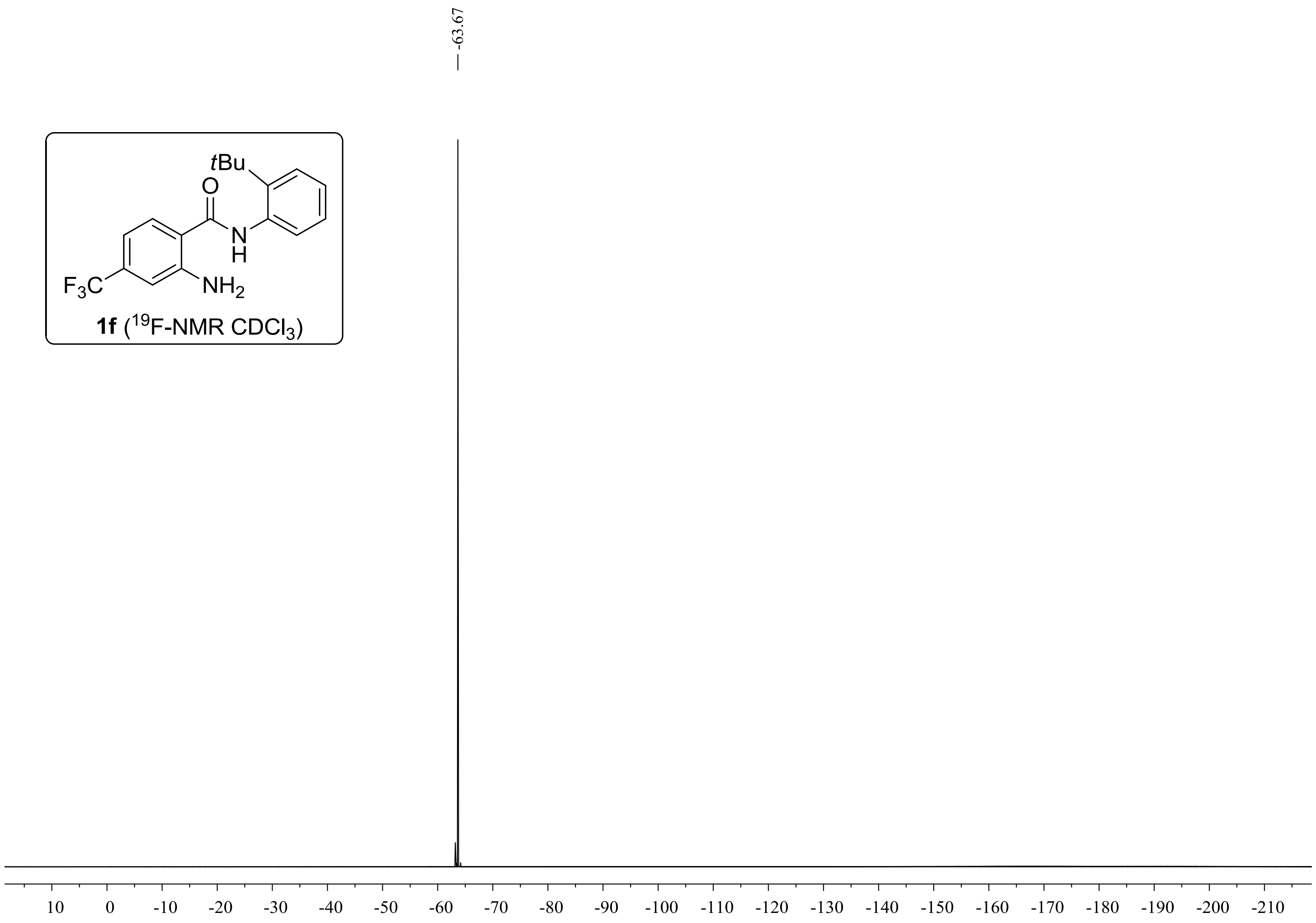


**Supplementary Figure 16.** $^{19}F$ NMR of **1f**

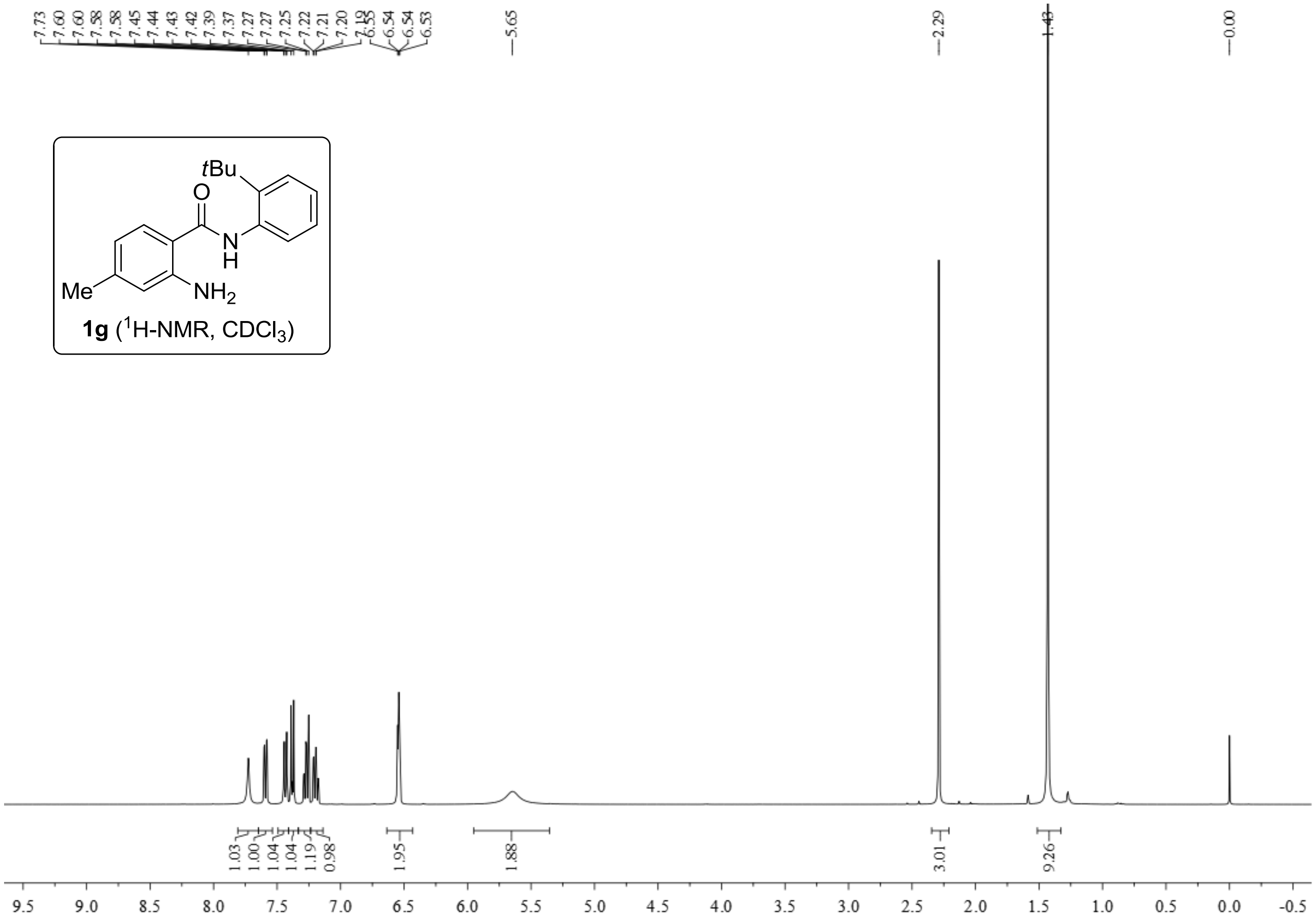


**Supplementary Figure 17.** $^{1}$H NMR of **1g**

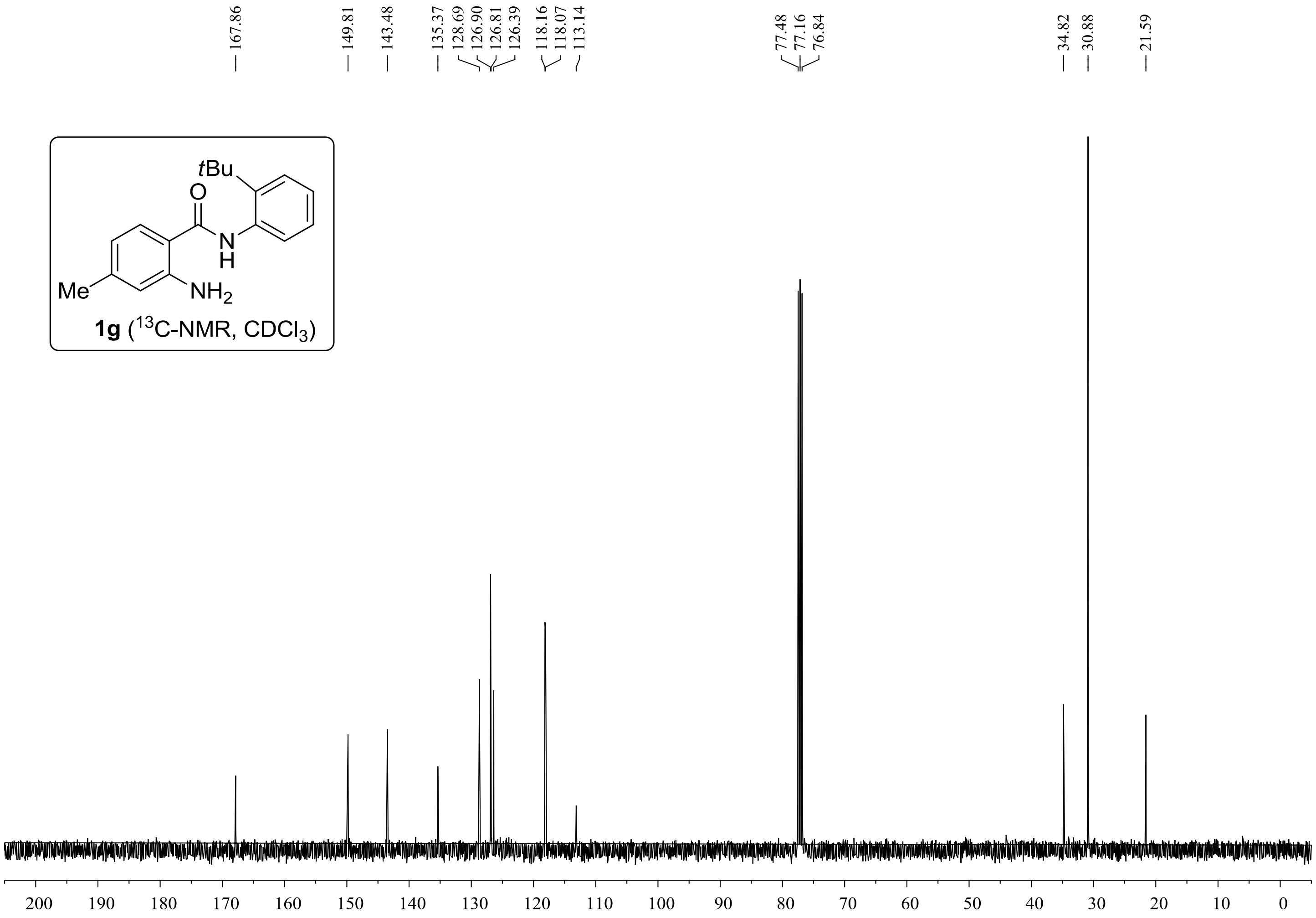


**Supplementary Figure 18.** $^{13}$C NMR of **1g**

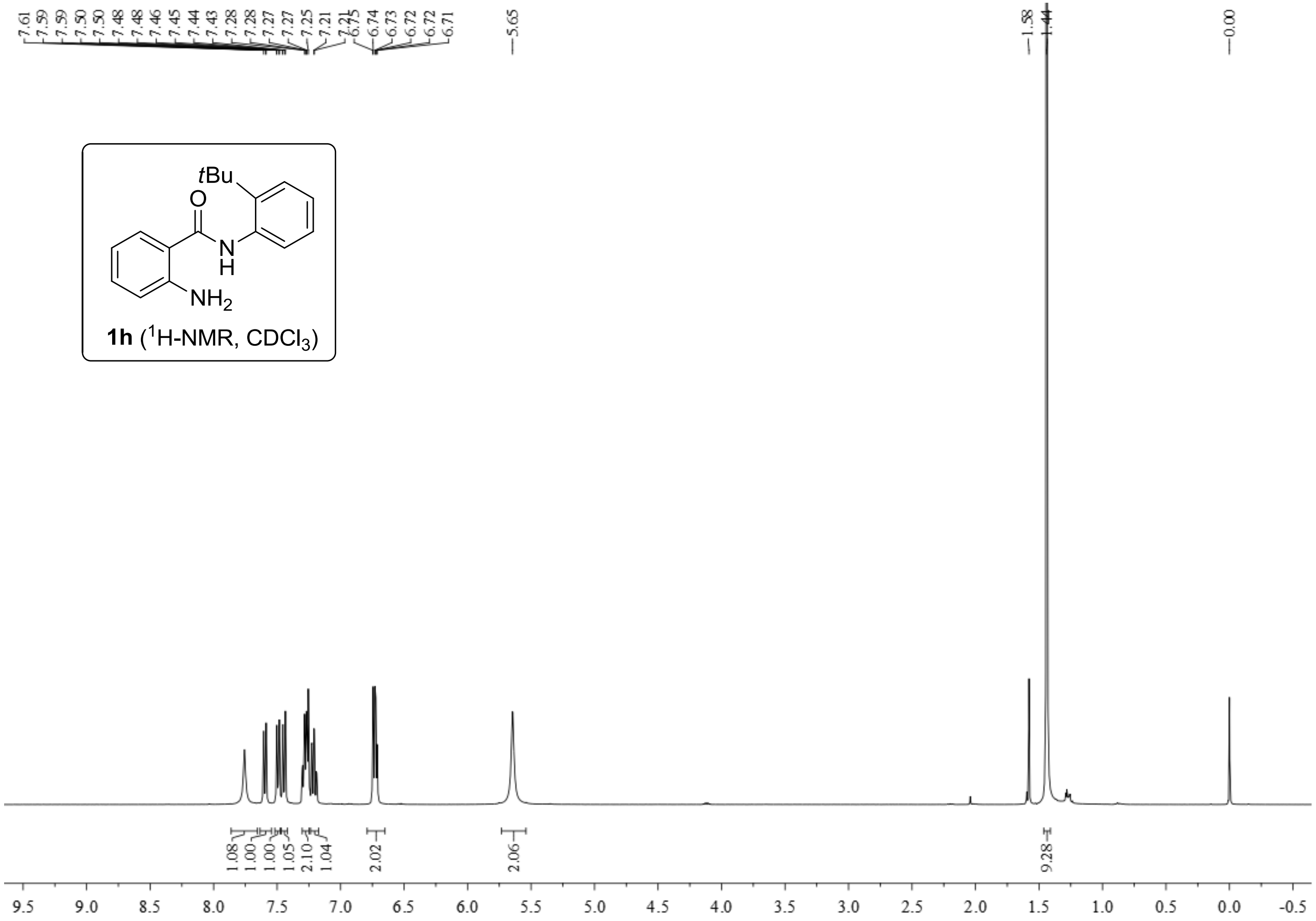

**Supplementary Figure 19.** $^{1}$H NMR of **1h**

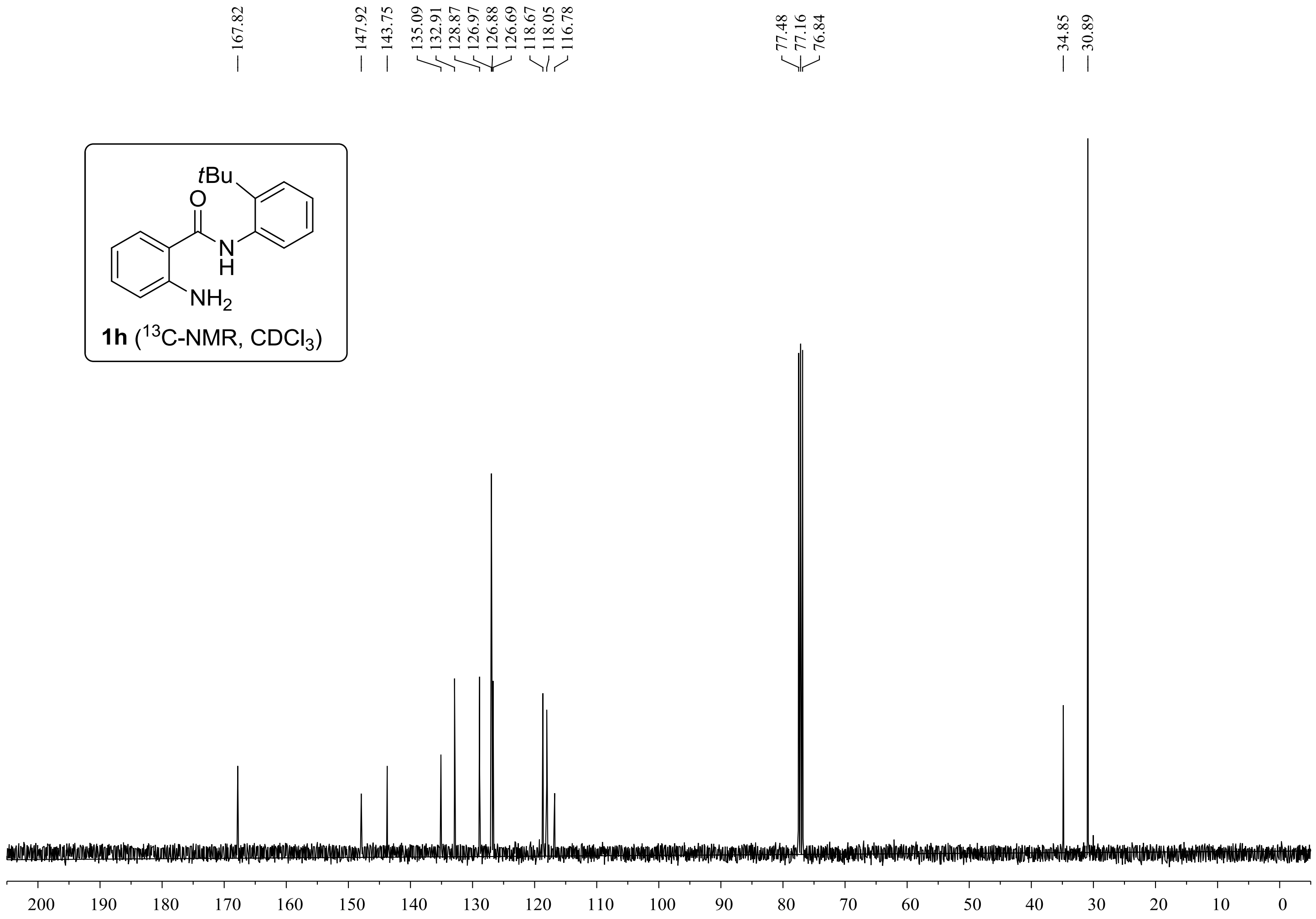


**Supplementary Figure 20.** $^{13}$C NMR of **1h**

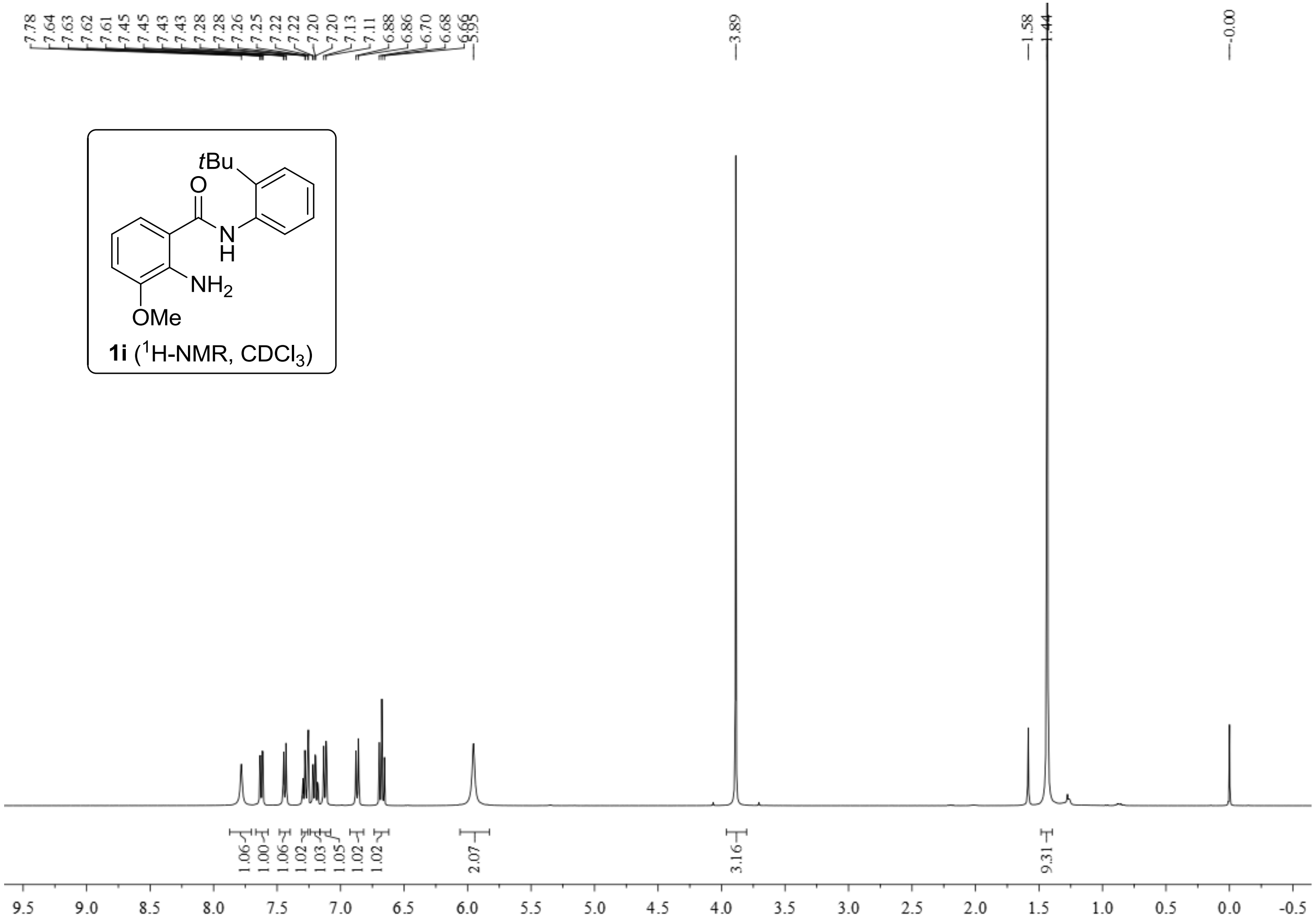


**Supplementary Figure 21.** $^1$H NMR of **1i**

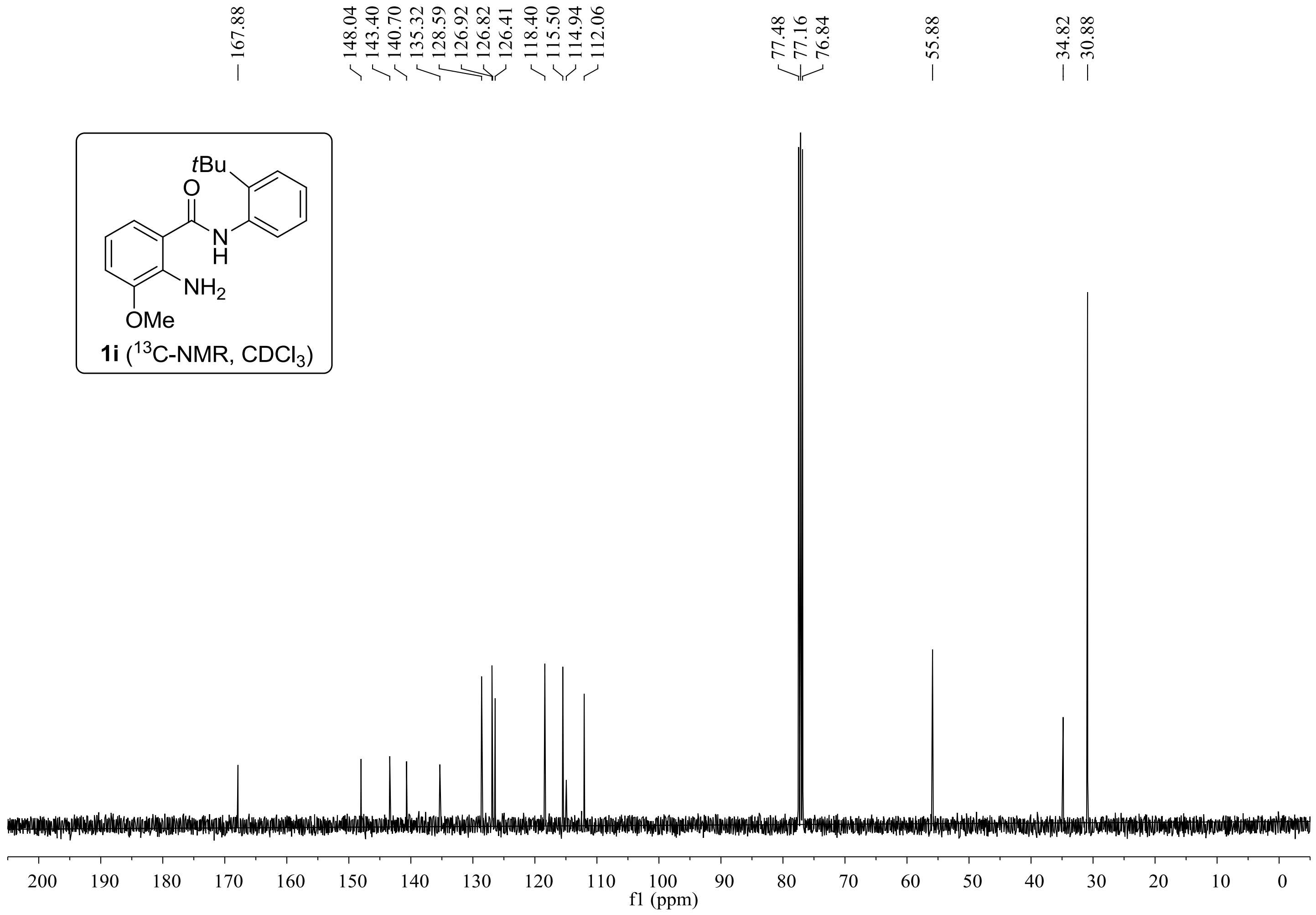


**Supplementary Figure 22.** $^{13}$C NMR of **1i**

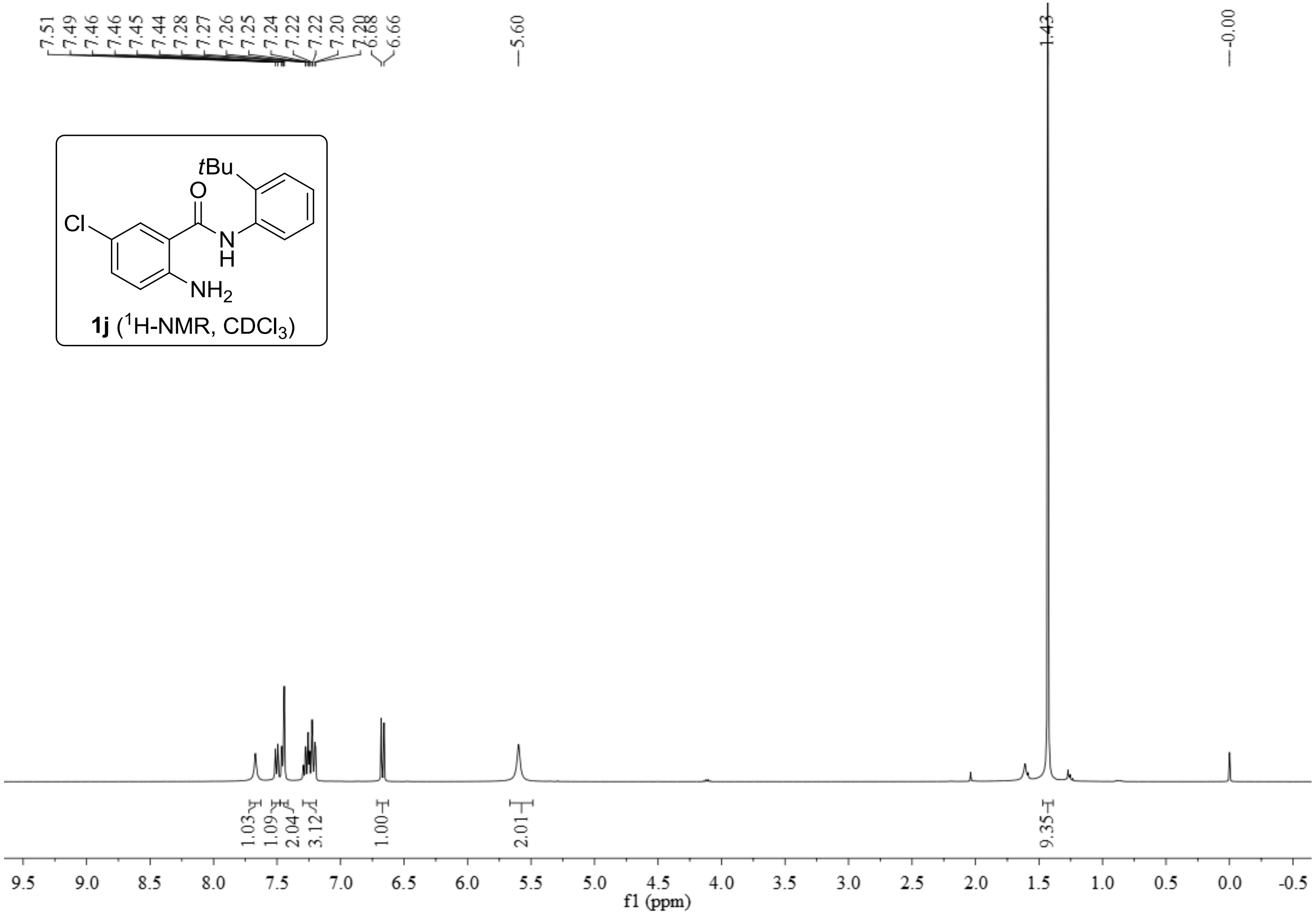


**Supplementary Figure 23.** $^{1}H$ NMR of **1j**

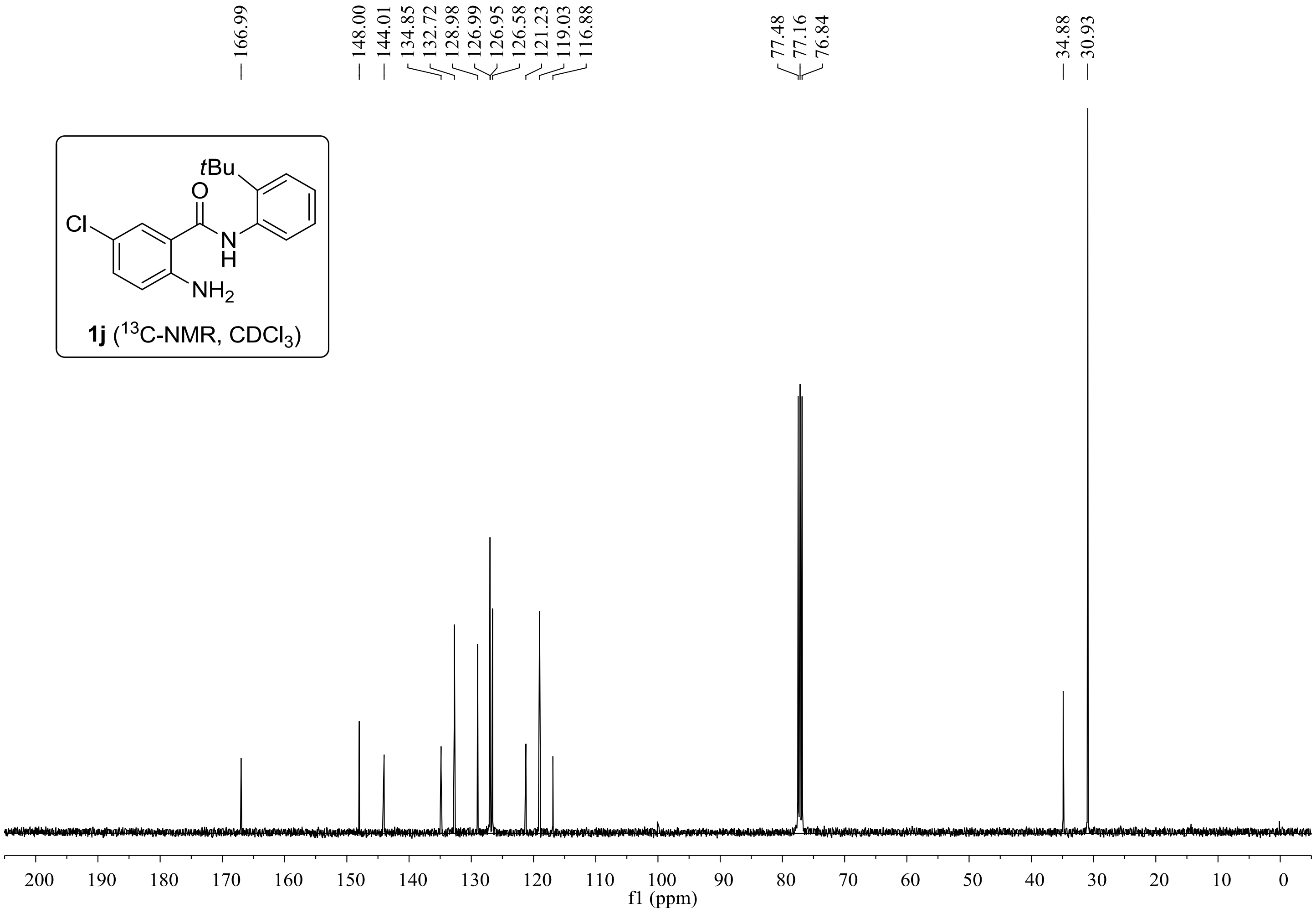


**Supplementary Figure 24.** $^{13}$C NMR of **1j**

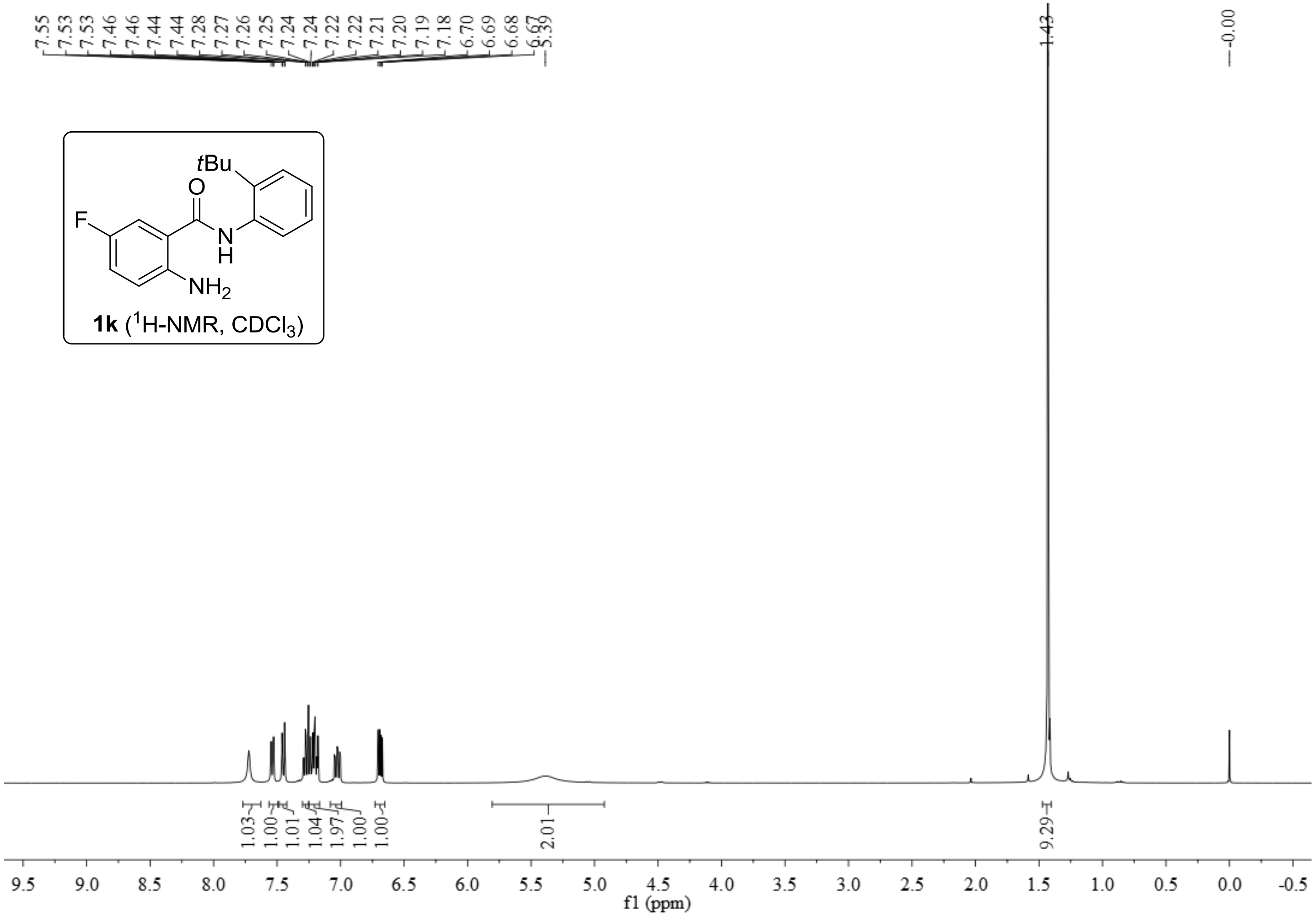


**Supplementary Figure 25.** $^1$H NMR of **1k**

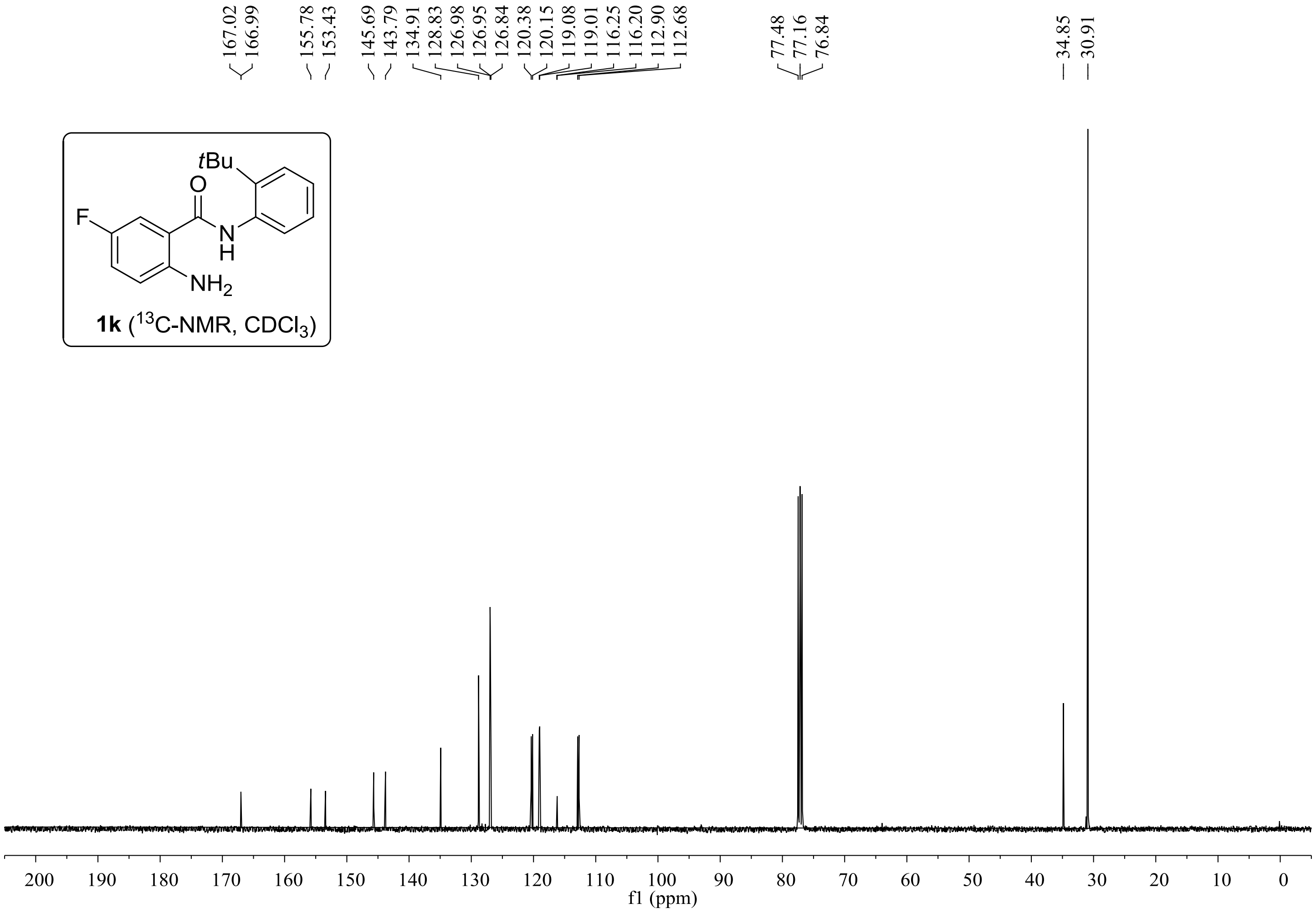


**Supplementary Figure 26.** $^{13}$C NMR of **1k**

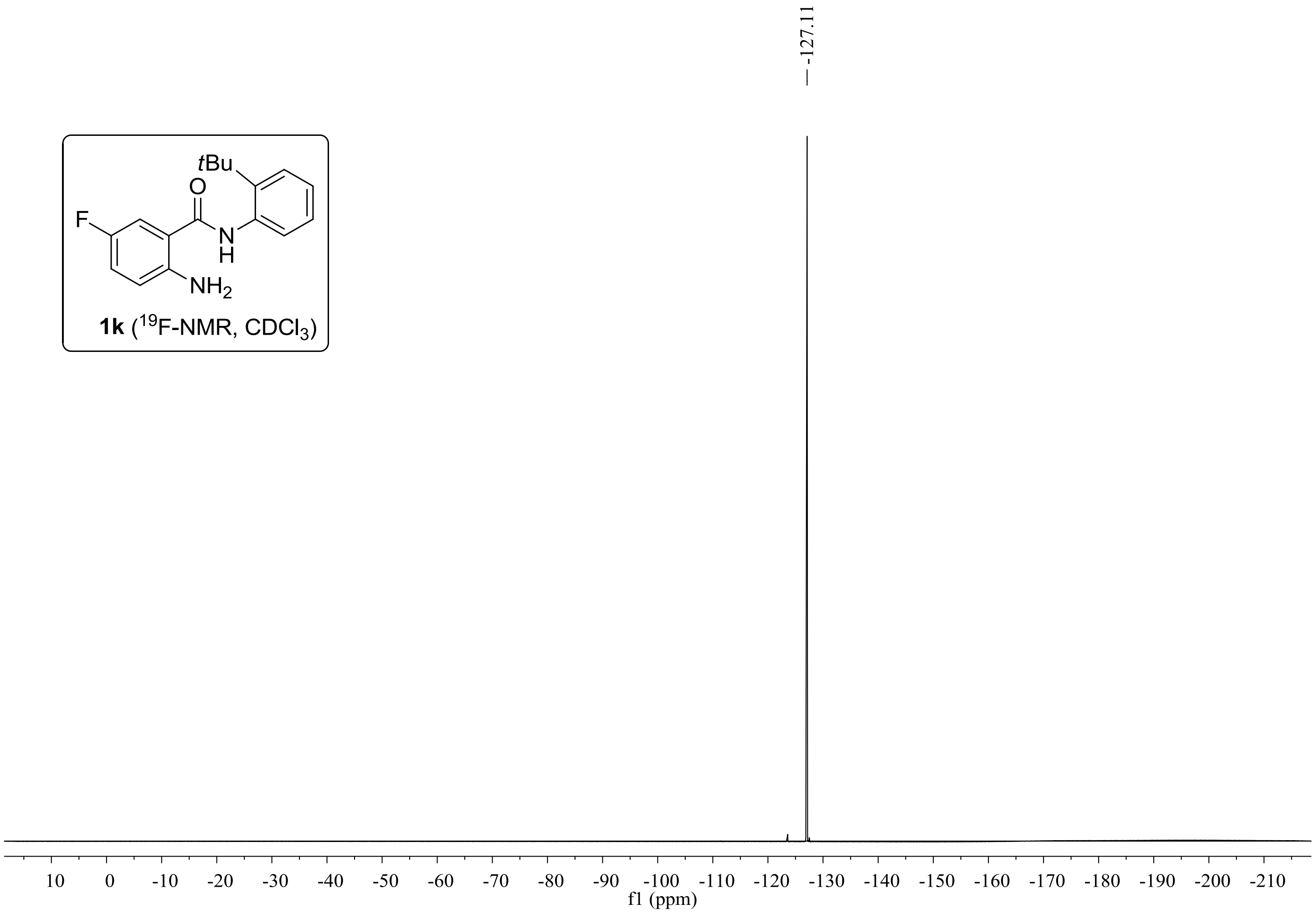


**Supplementary Figure 27.** $^{19}$F NMR of **1k**

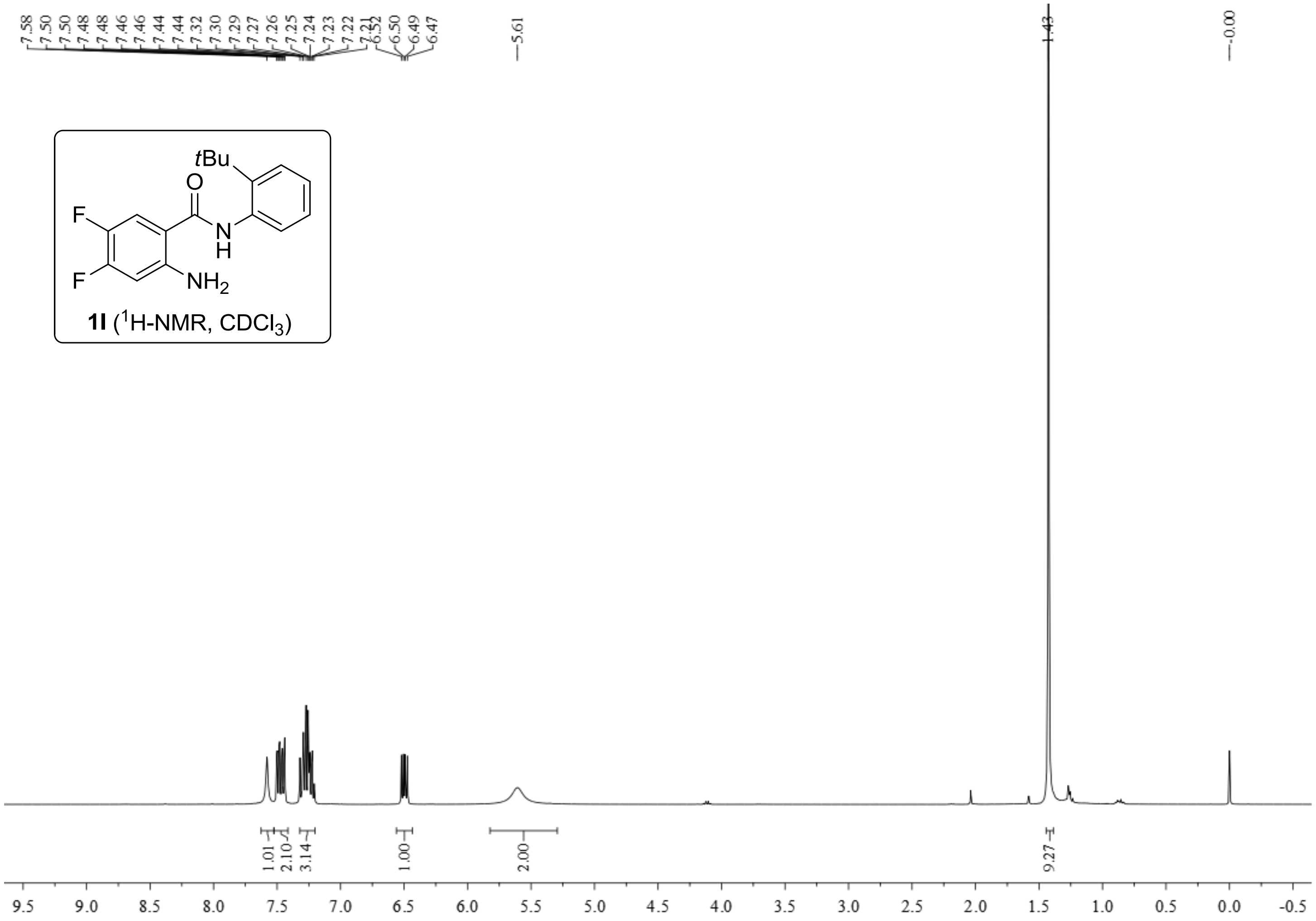


**Supplementary Figure 28.** $^{1}$H NMR of **1l**

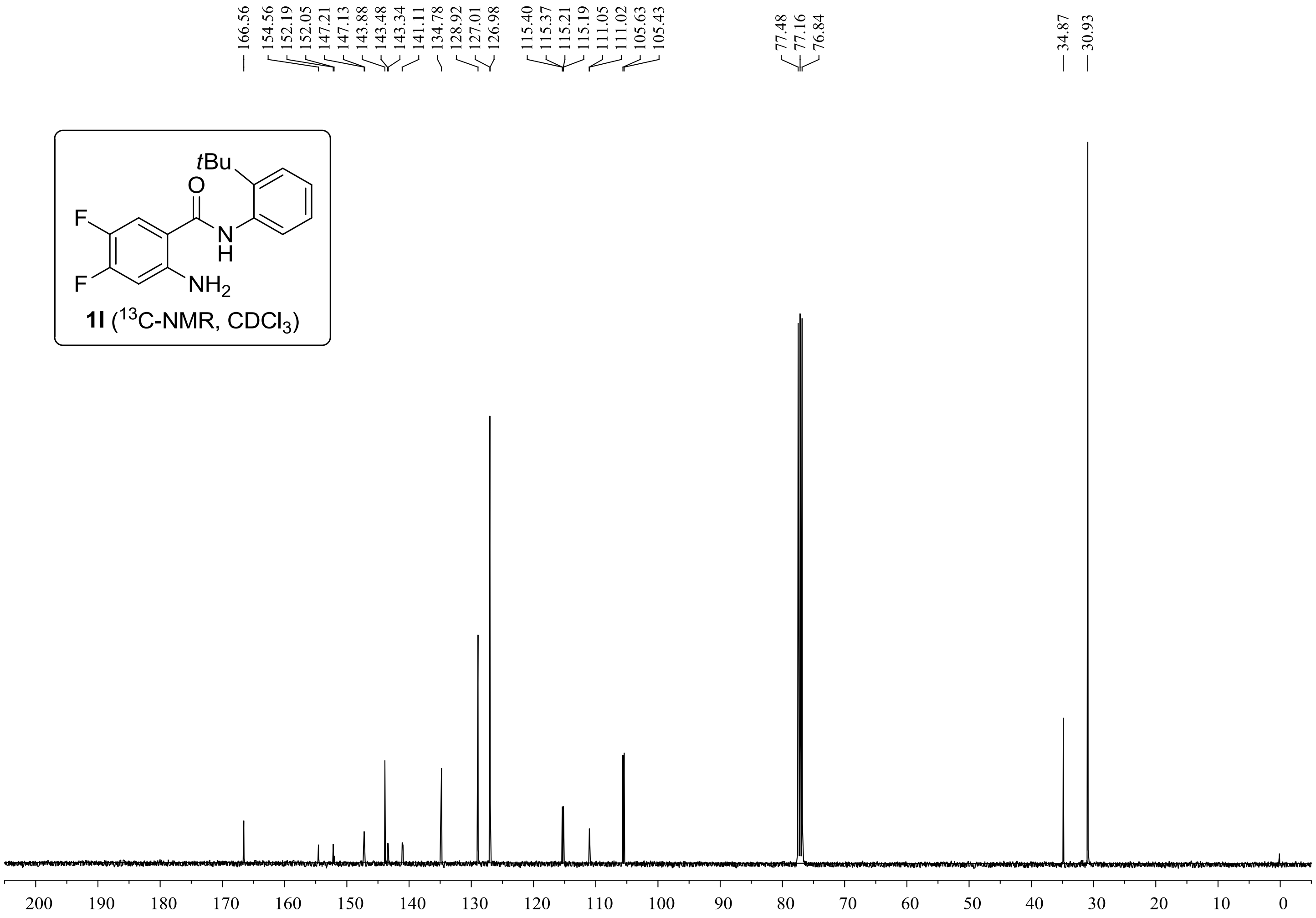


**Supplementary Figure 29.** $^{13}$C NMR of **1l**

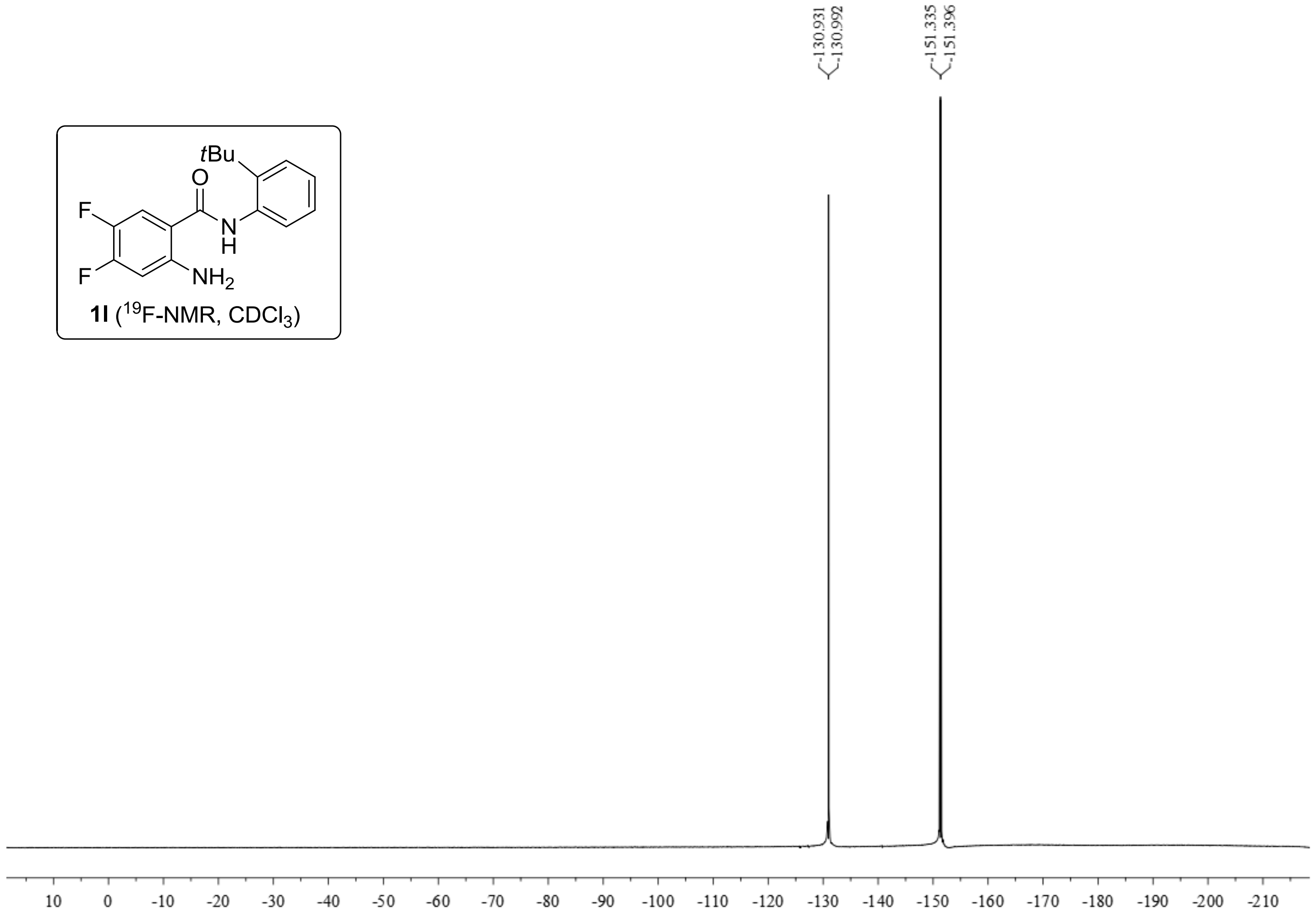


**Supplementary Figure 30.** $^{19}$F NMR of **1l**

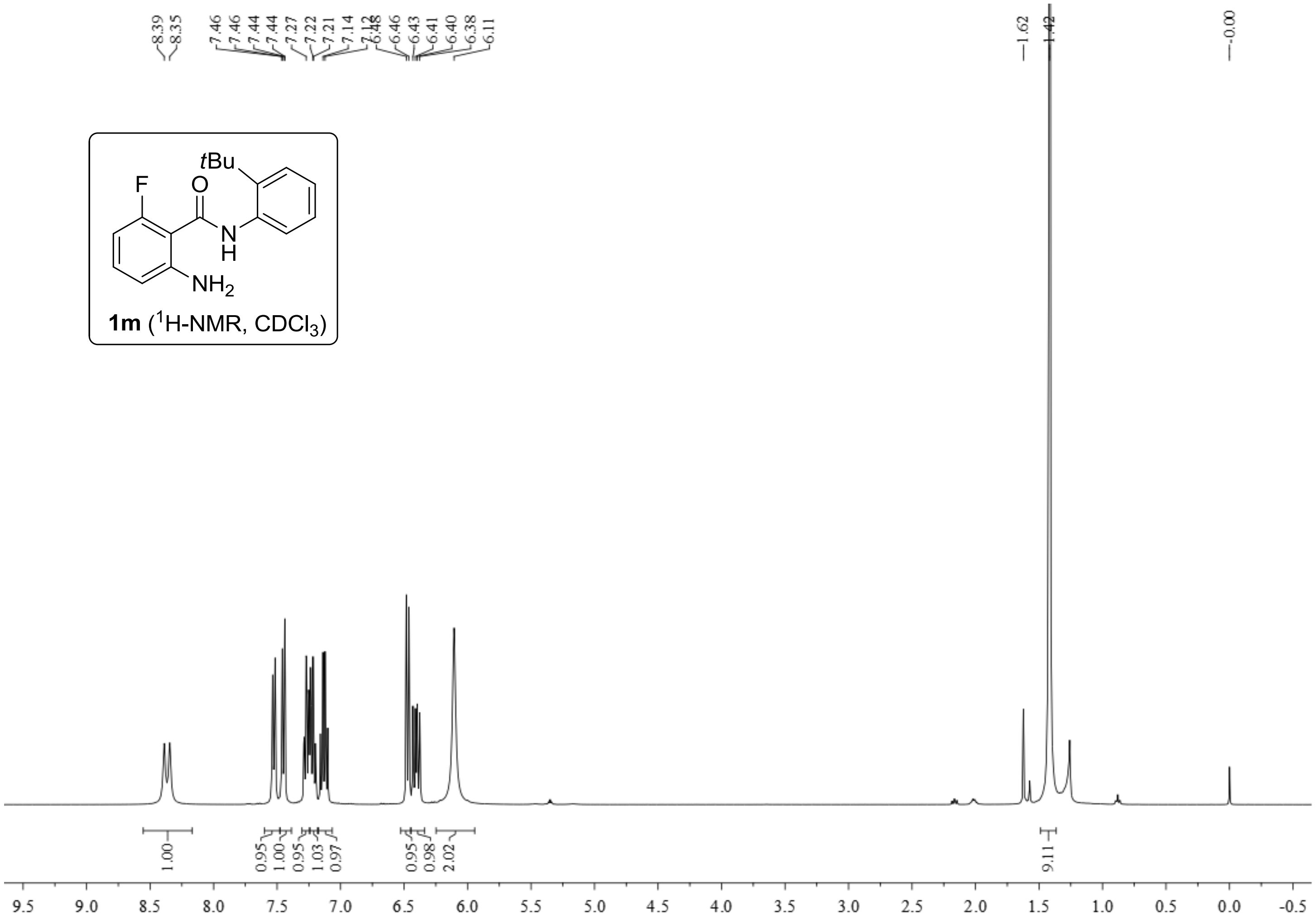


**Supplementary Figure 31.** $^{1}$H NMR of **1m**

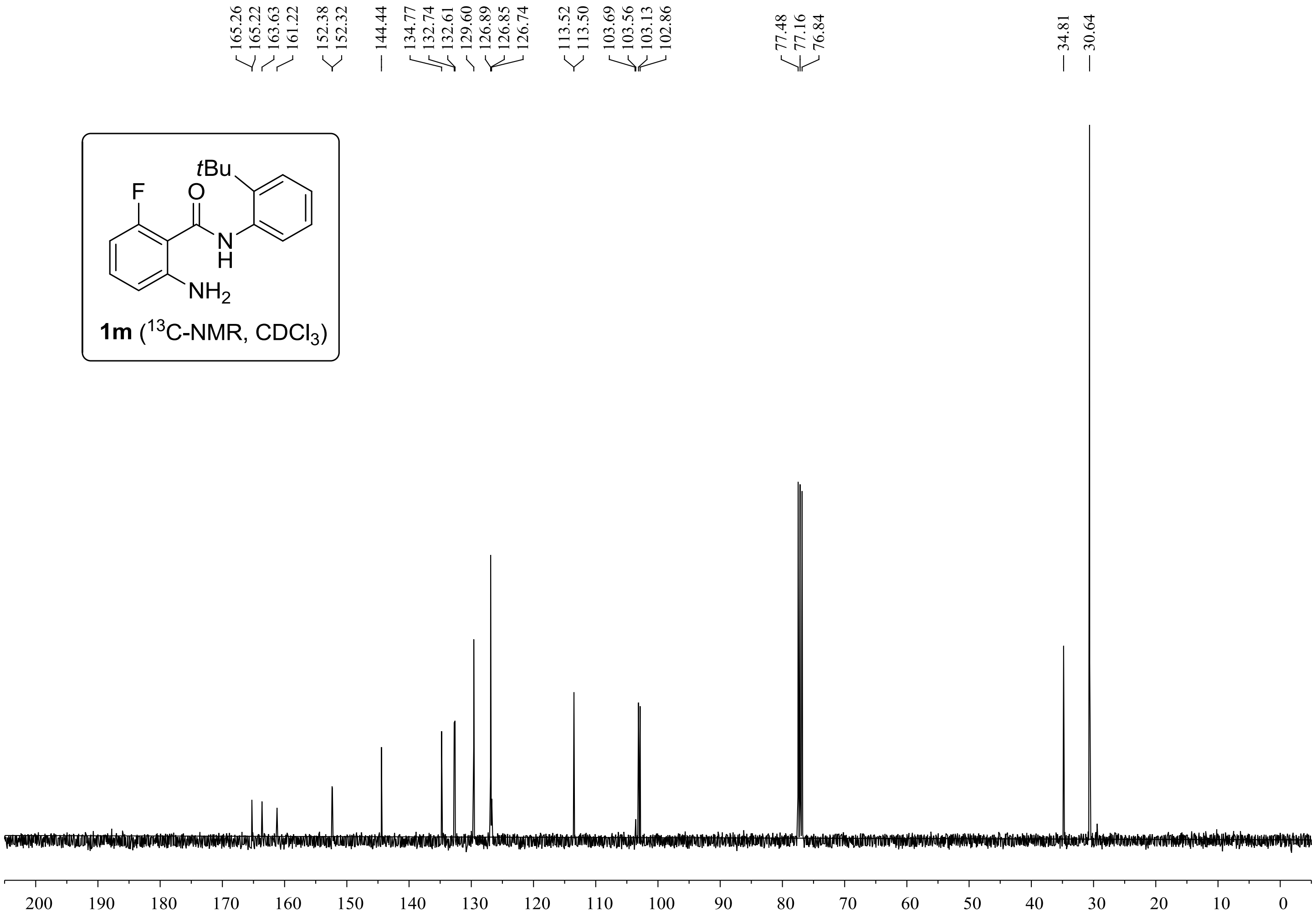


**Supplementary Figure 32.** $^{13}$C NMR of **1m**

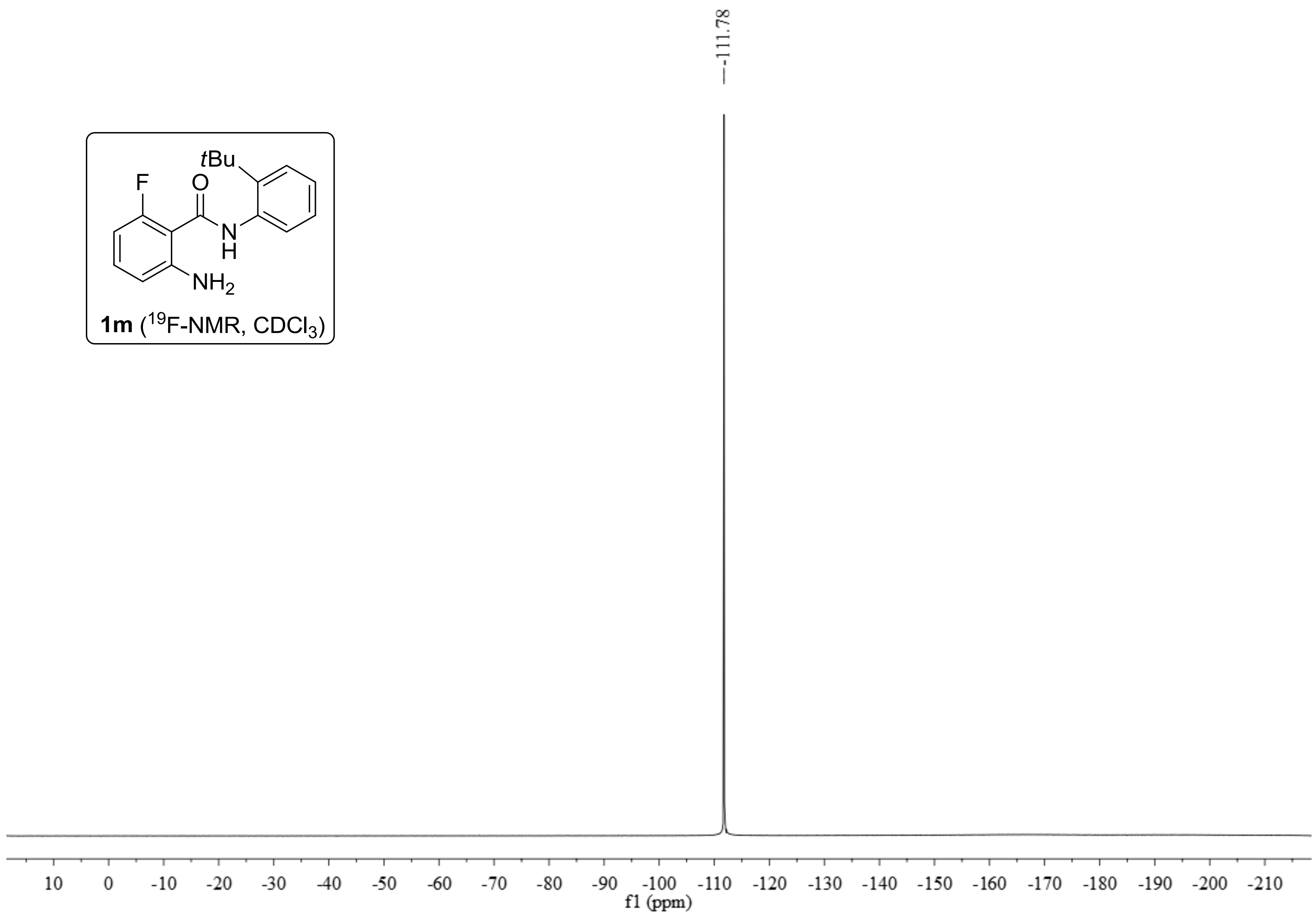


**Supplementary Figure 33.** $^{19}$F NMR of **1m**

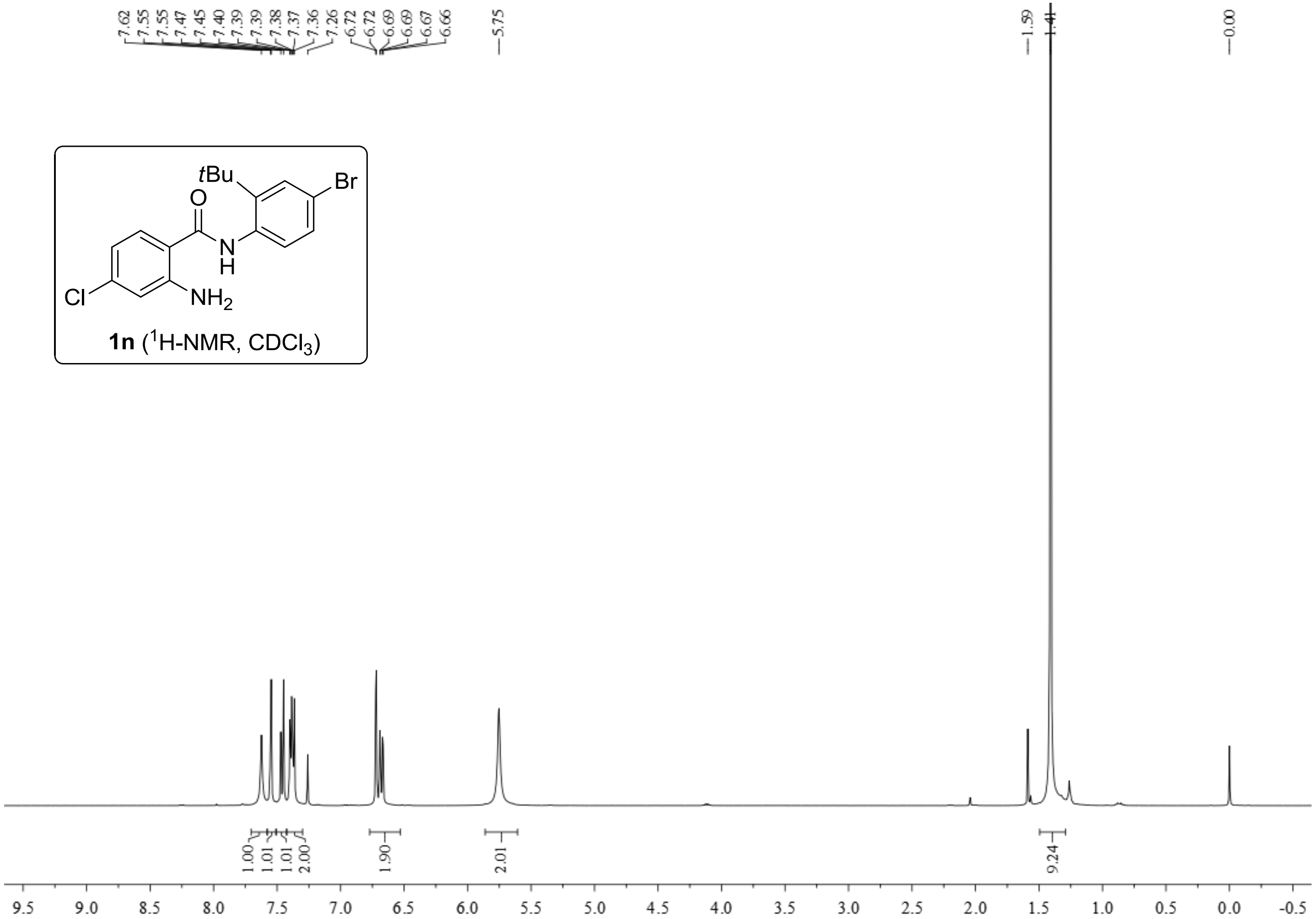


**Supplementary Figure 34.** $^{1}$H NMR of **1n**

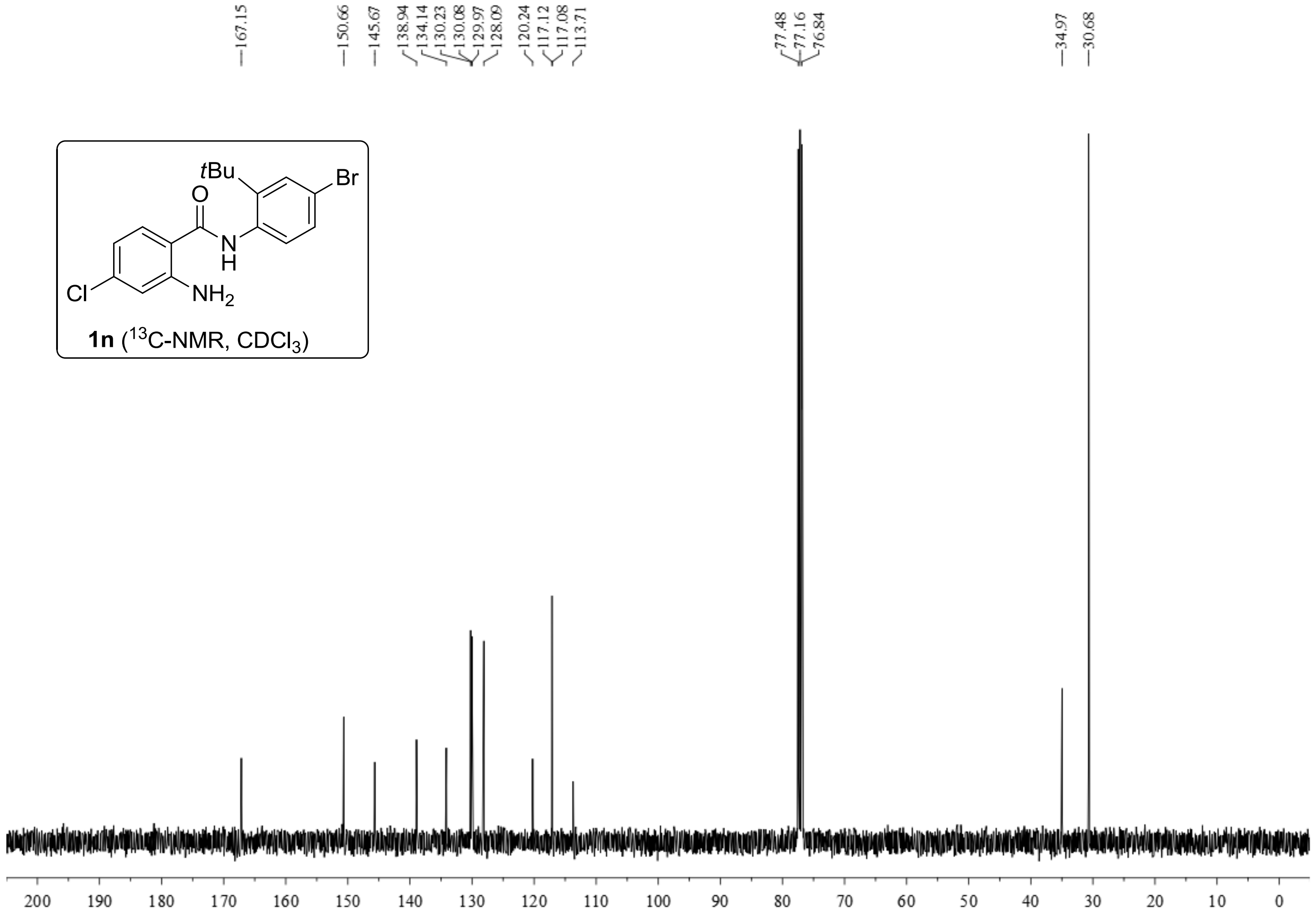


**Supplementary Figure 35.** $^{13}$C NMR of **1n**

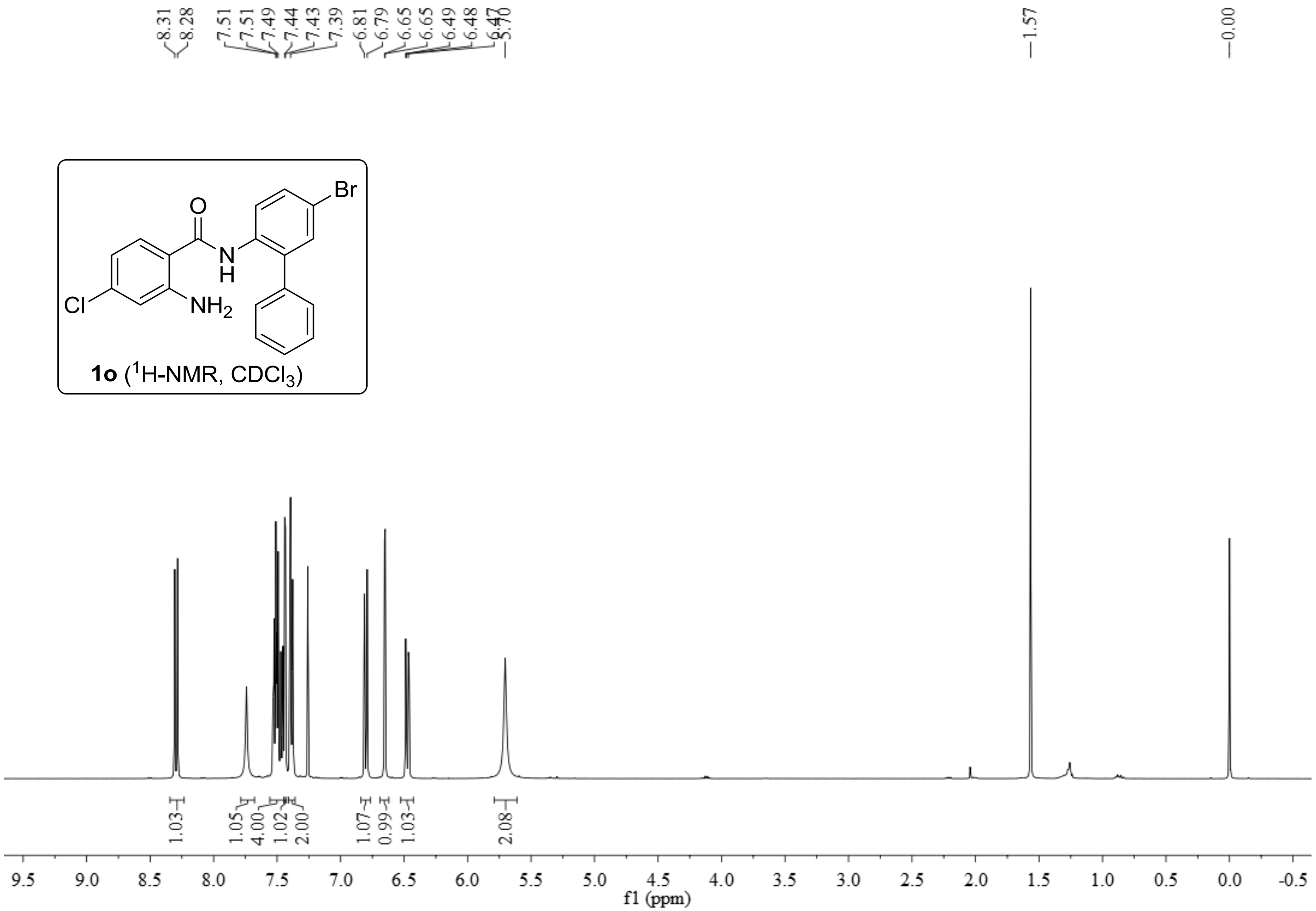


**Supplementary Figure 36.** $^{1}$H NMR of **1o**

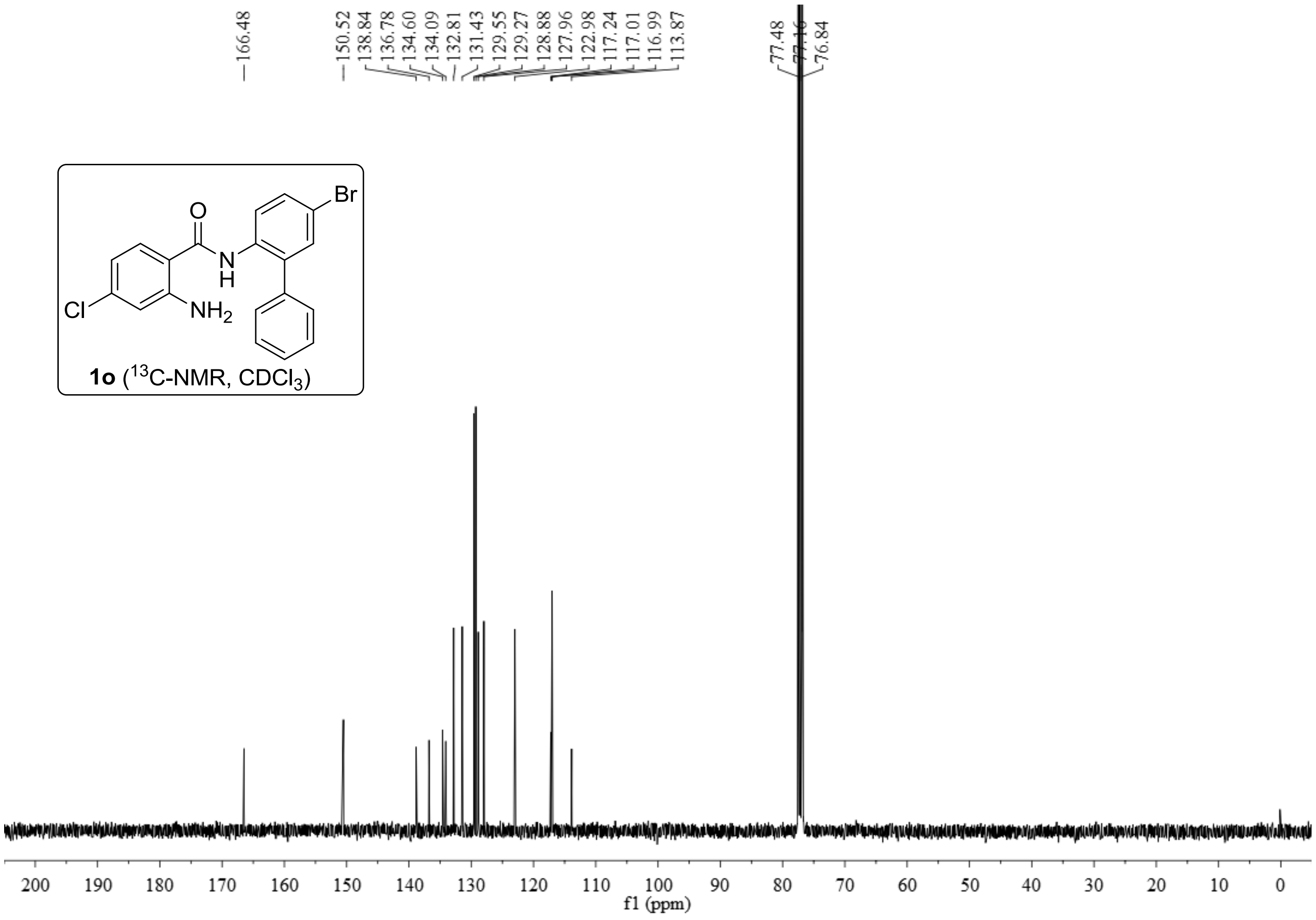


**Supplementary Figure 37.** $^{13}$C NMR of **1o**

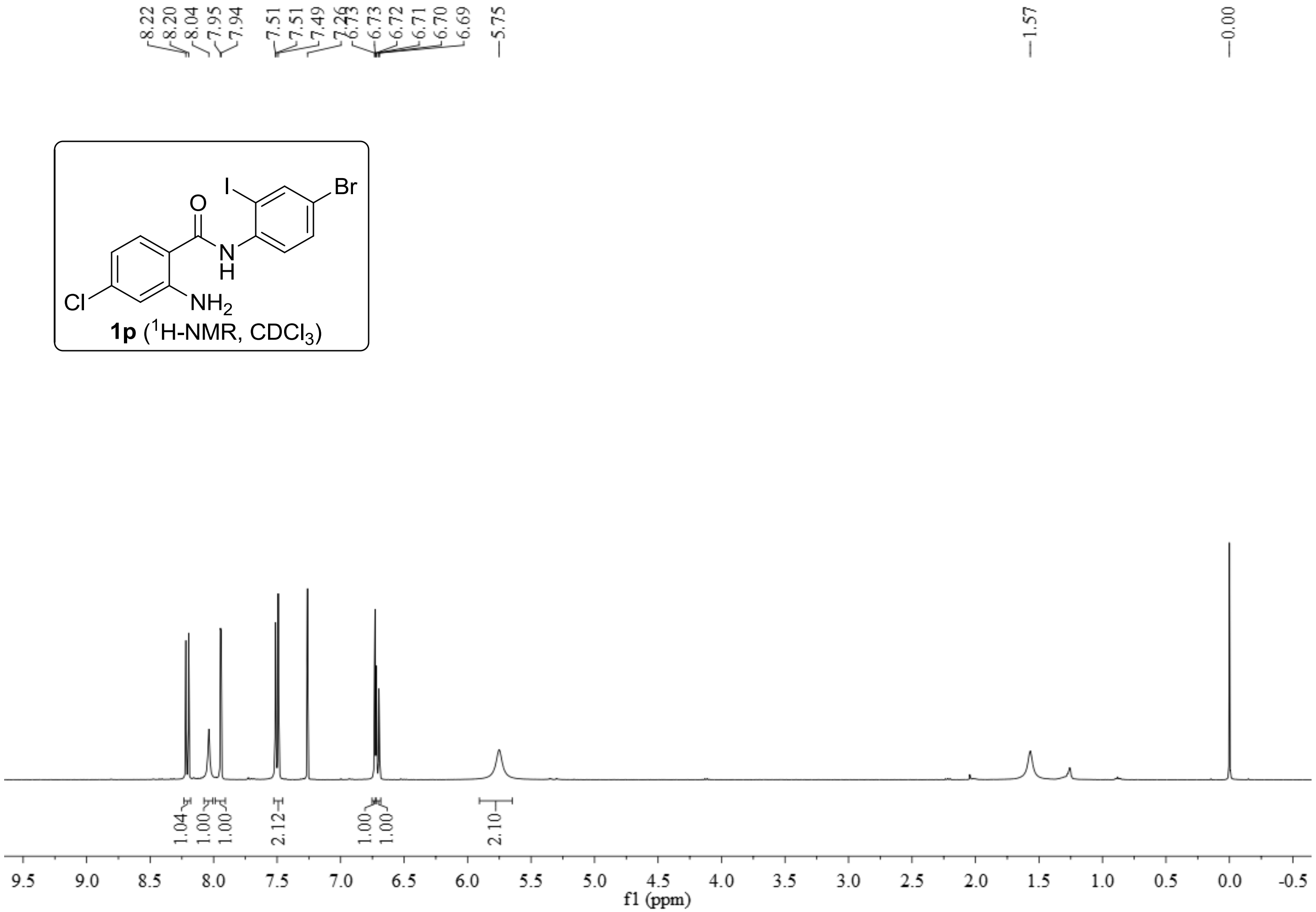


**Supplementary Figure 38.** $^1$H NMR of **1p**

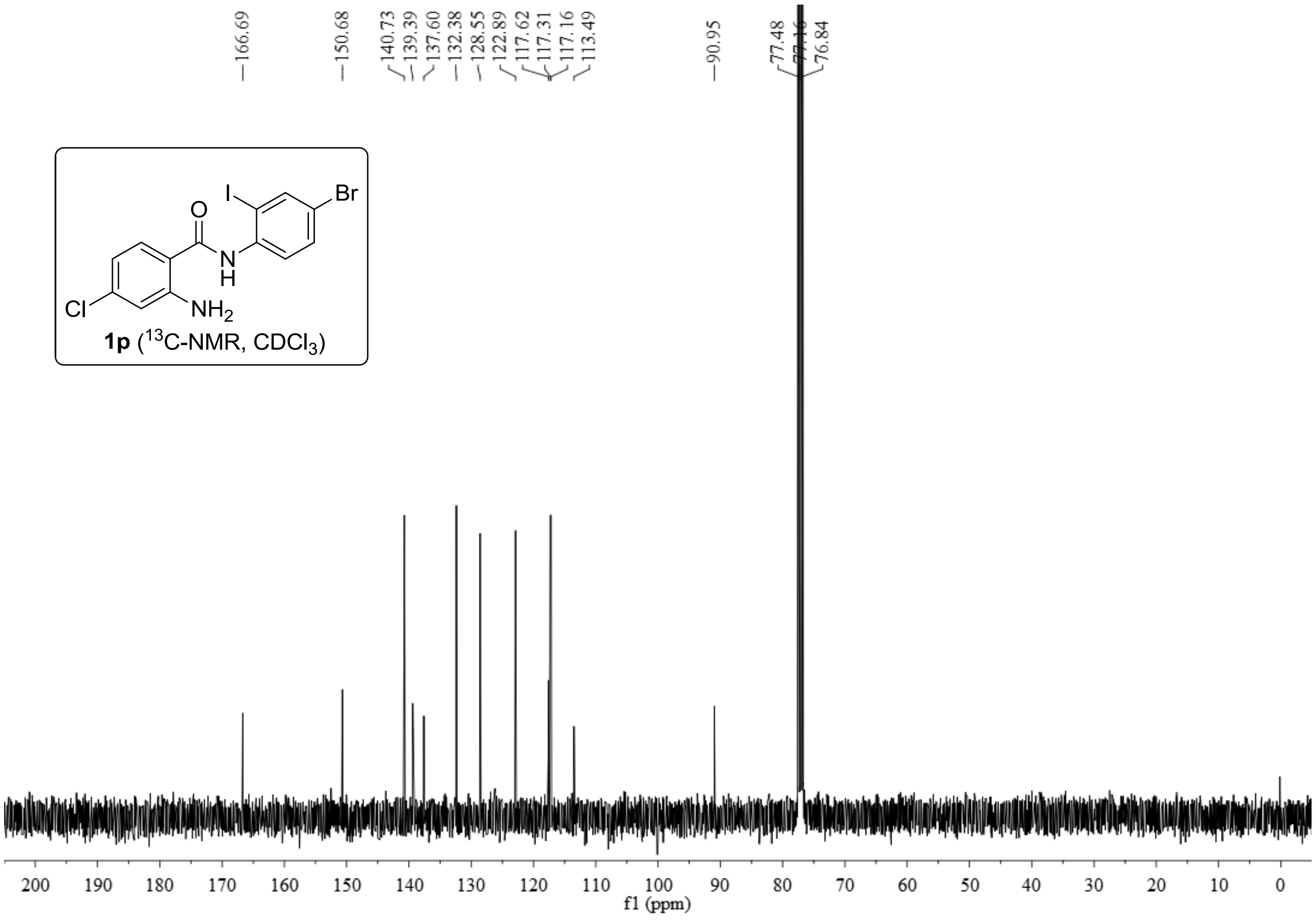


**Supplementary Figure 39.** $^{13}$C NMR of **1p**

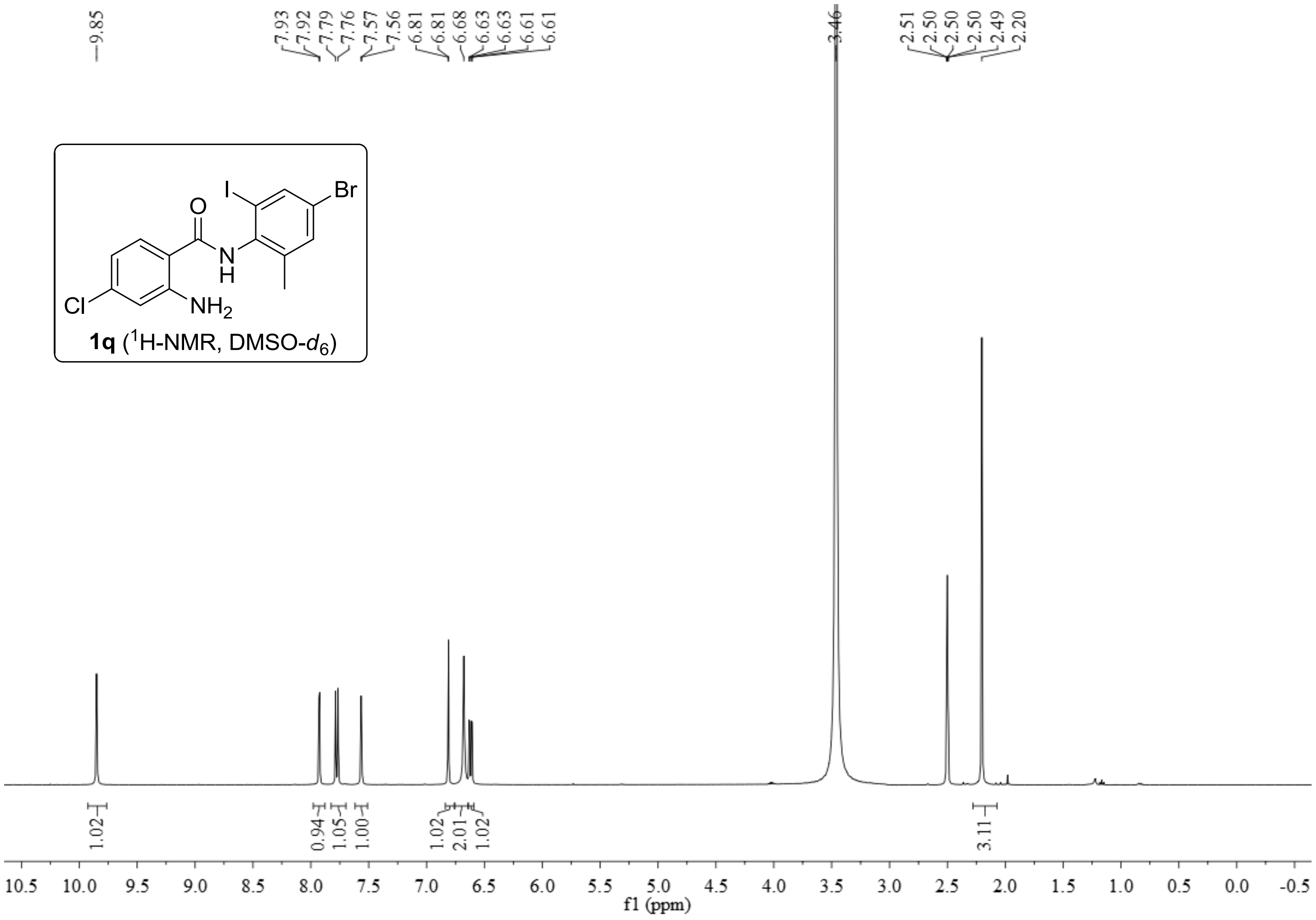


**Supplementary Figure 40.** $^{1}$H NMR of **1q**

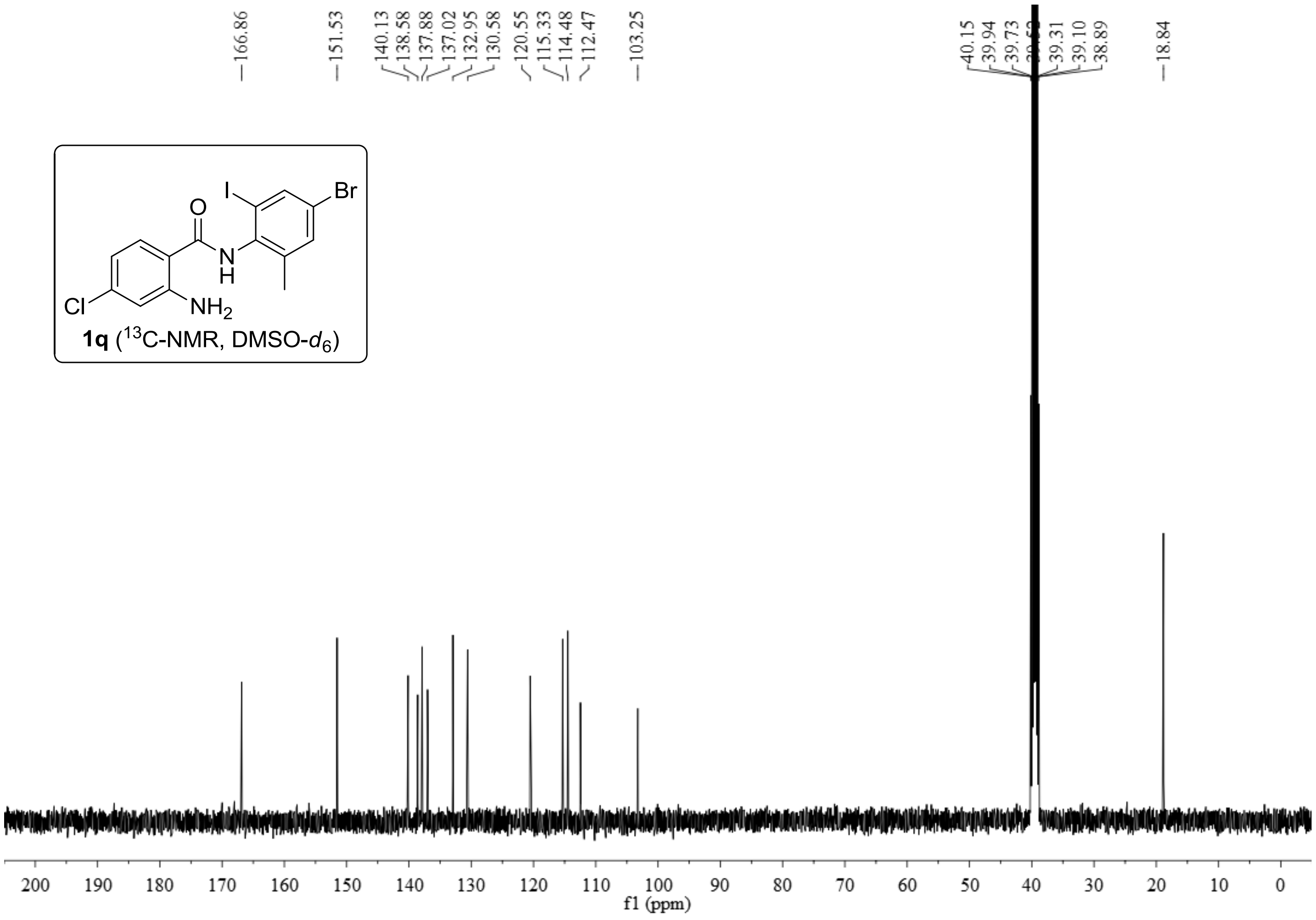


**Supplementary Figure 41.** $^{13}$C NMR of **1q**

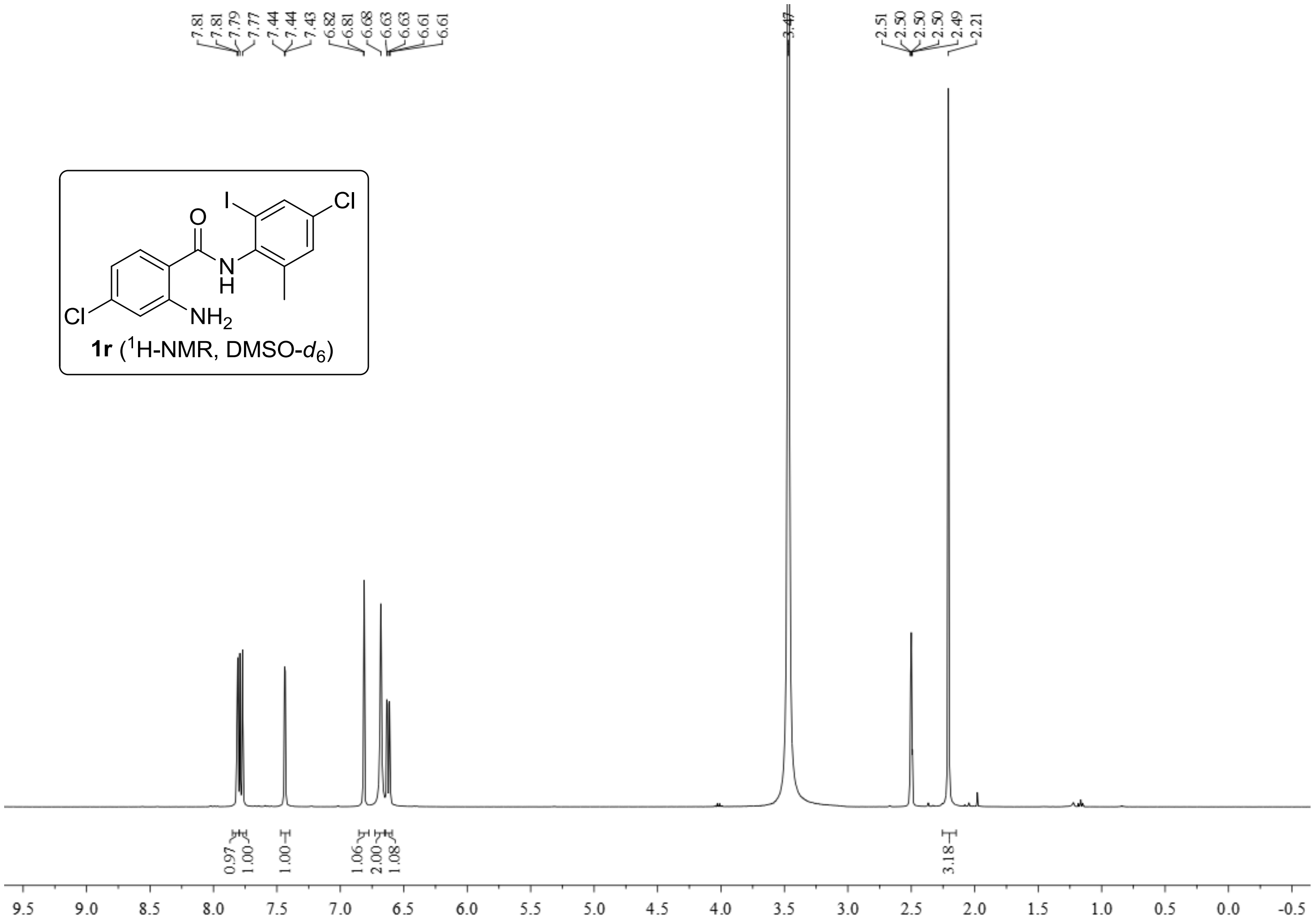


**Supplementary Figure 42.** $^{1}$H NMR of **1r**

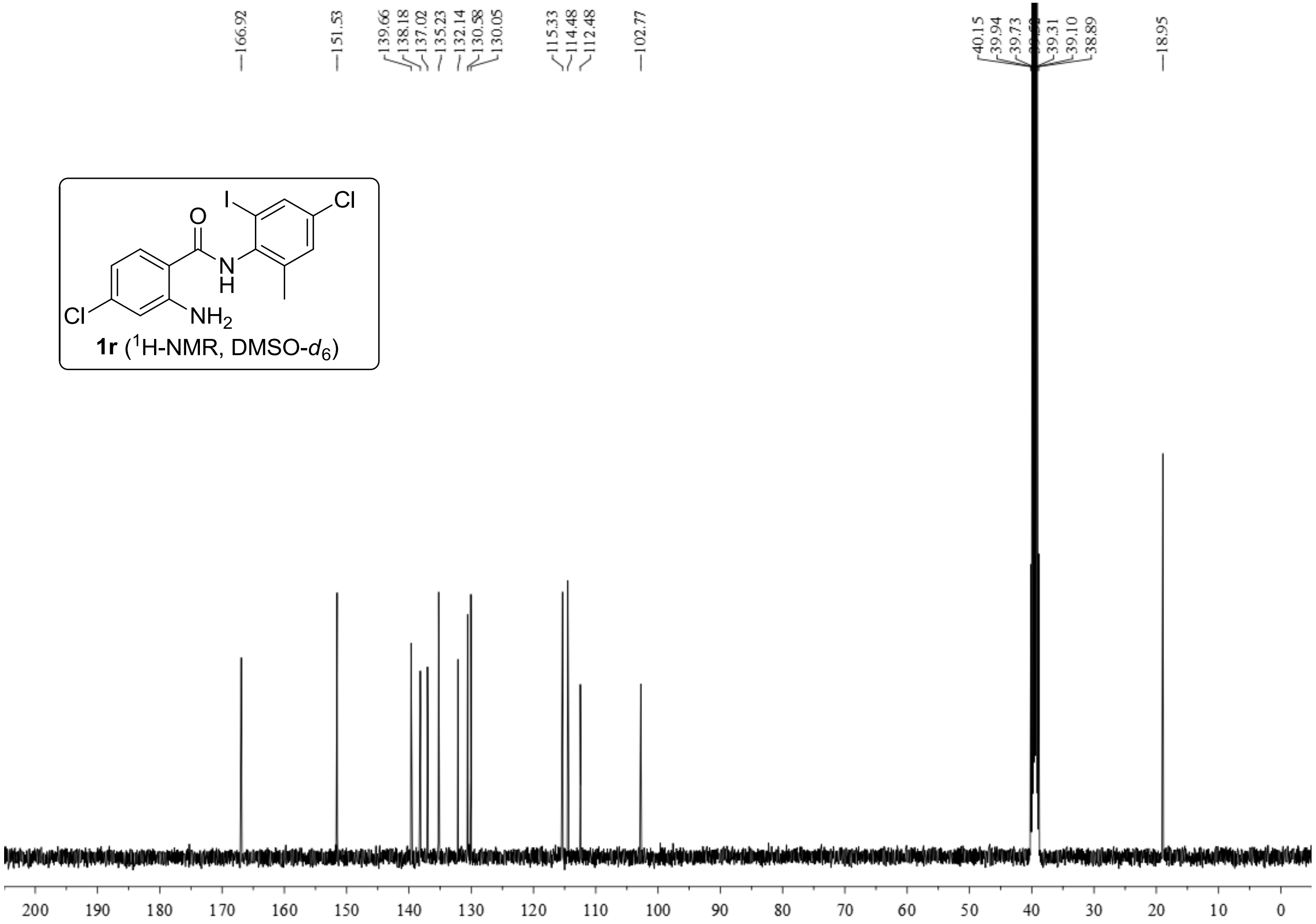


**Supplementary Figure 43.** $^{13}$C NMR of **1r**

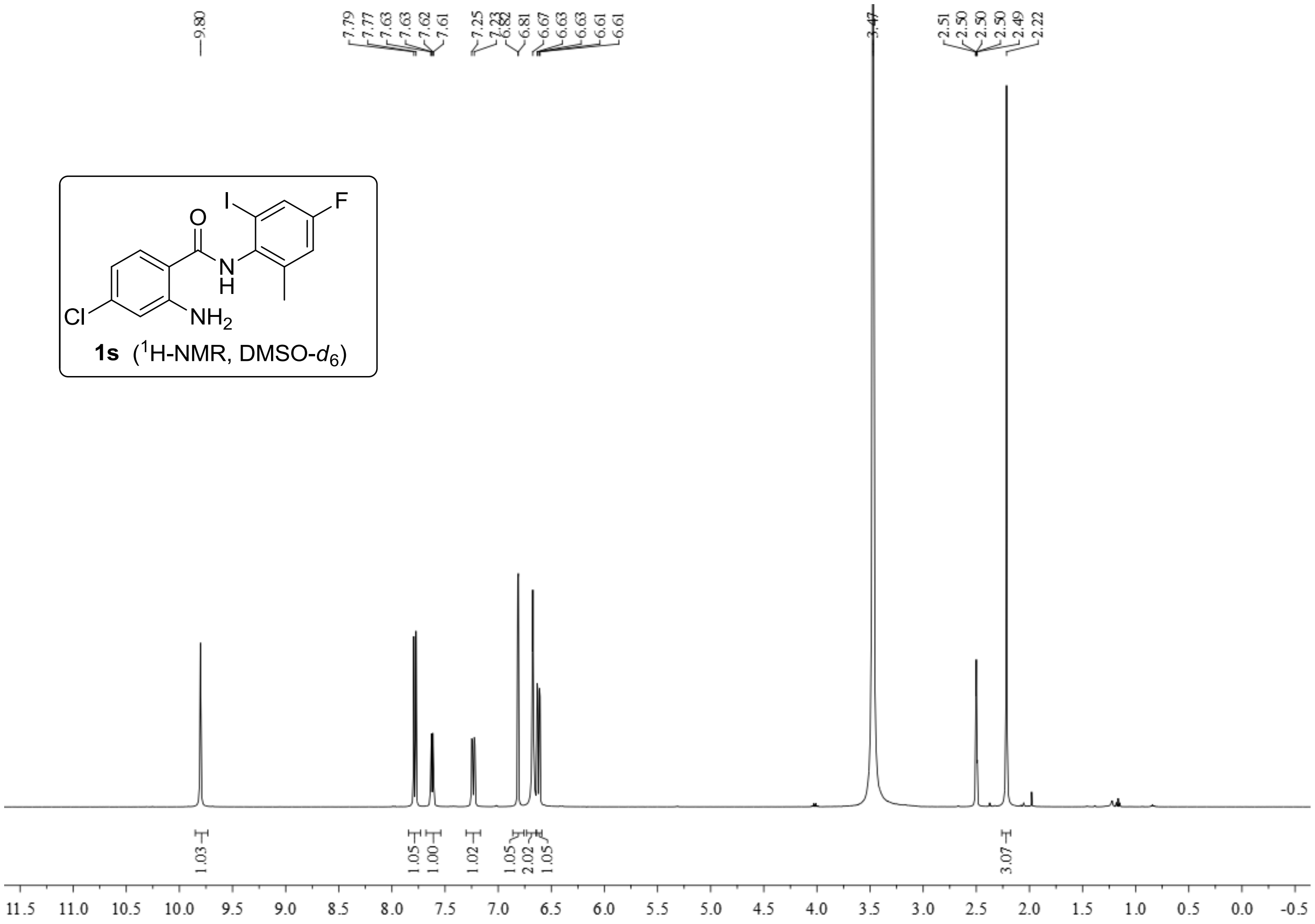


**Supplementary Figure 44.** $^1$H NMR of **1s**

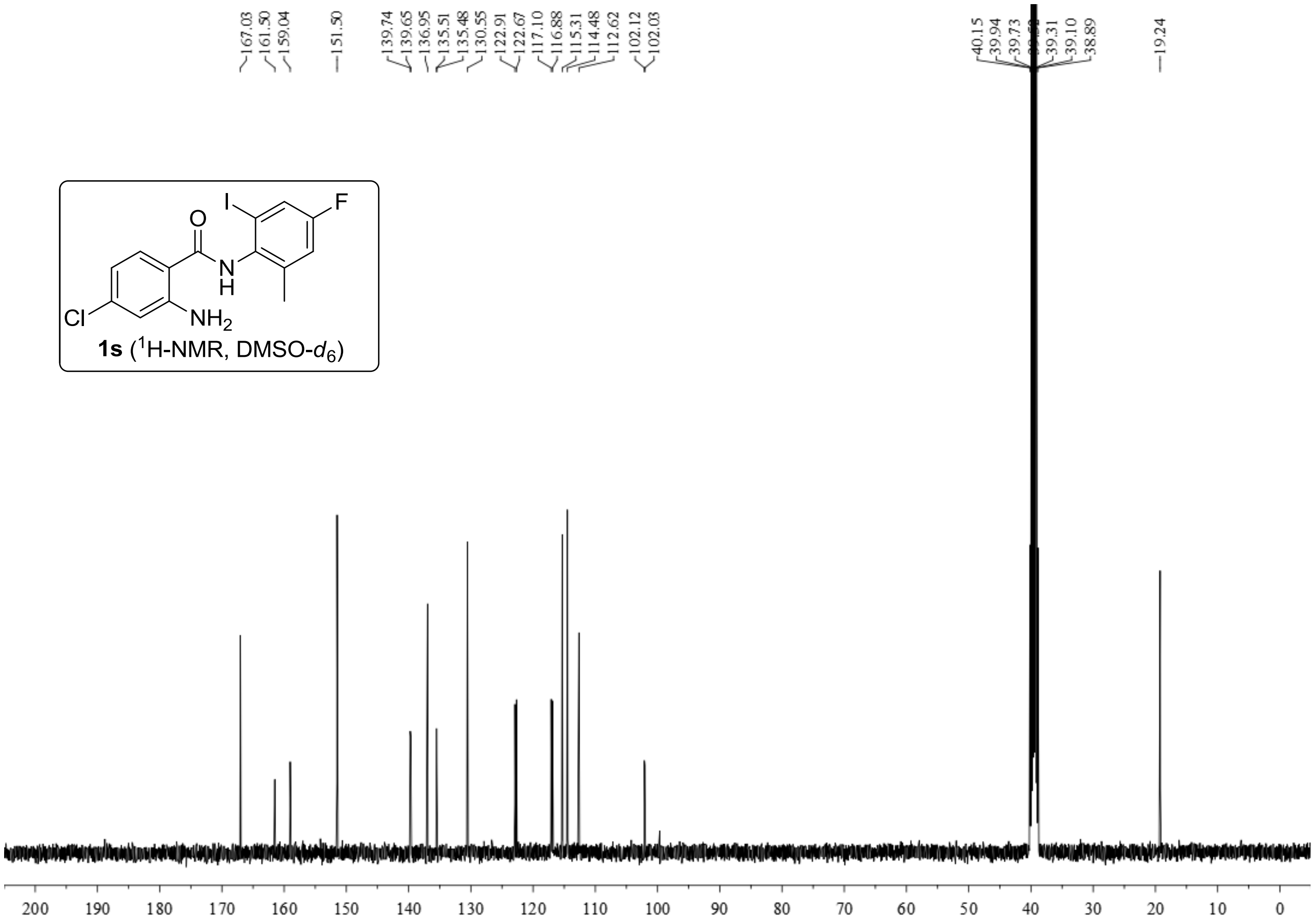


**Supplementary Figure 45.** $^{13}$C NMR of **1s**

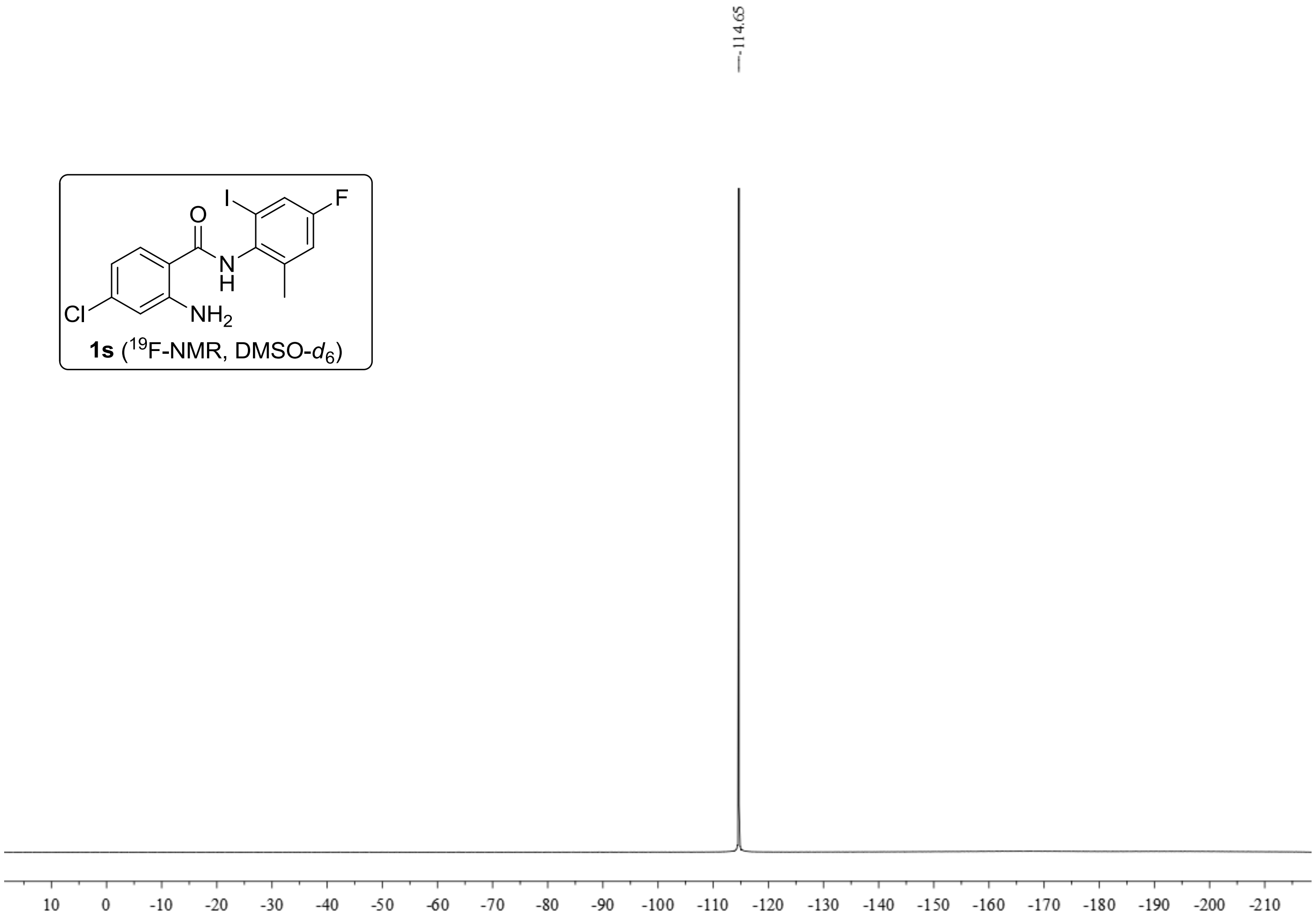


**Supplementary Figure 46.** $^{19}$F NMR of **1s**

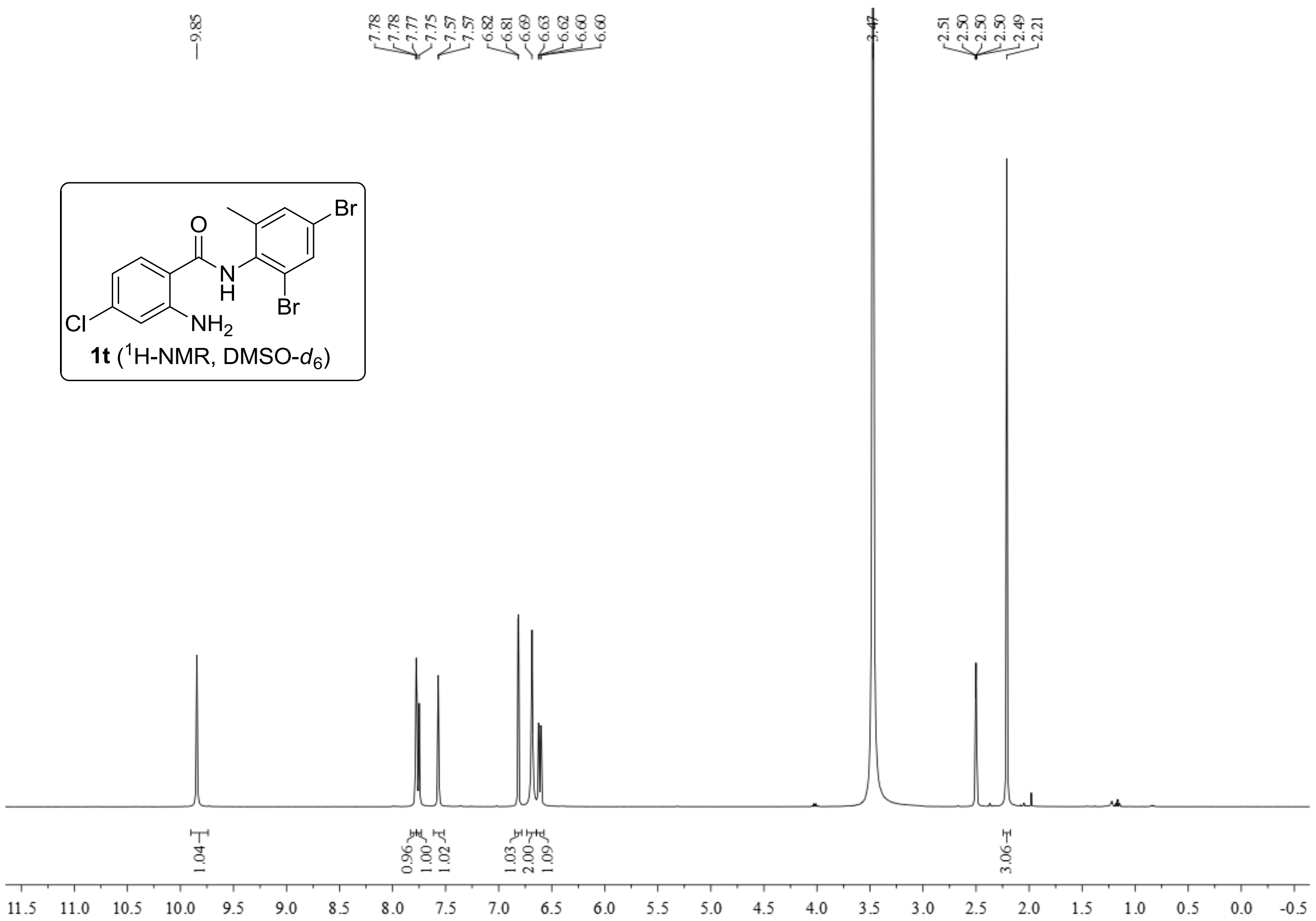


**Supplementary Figure 47.** $^1$H NMR of **1t**

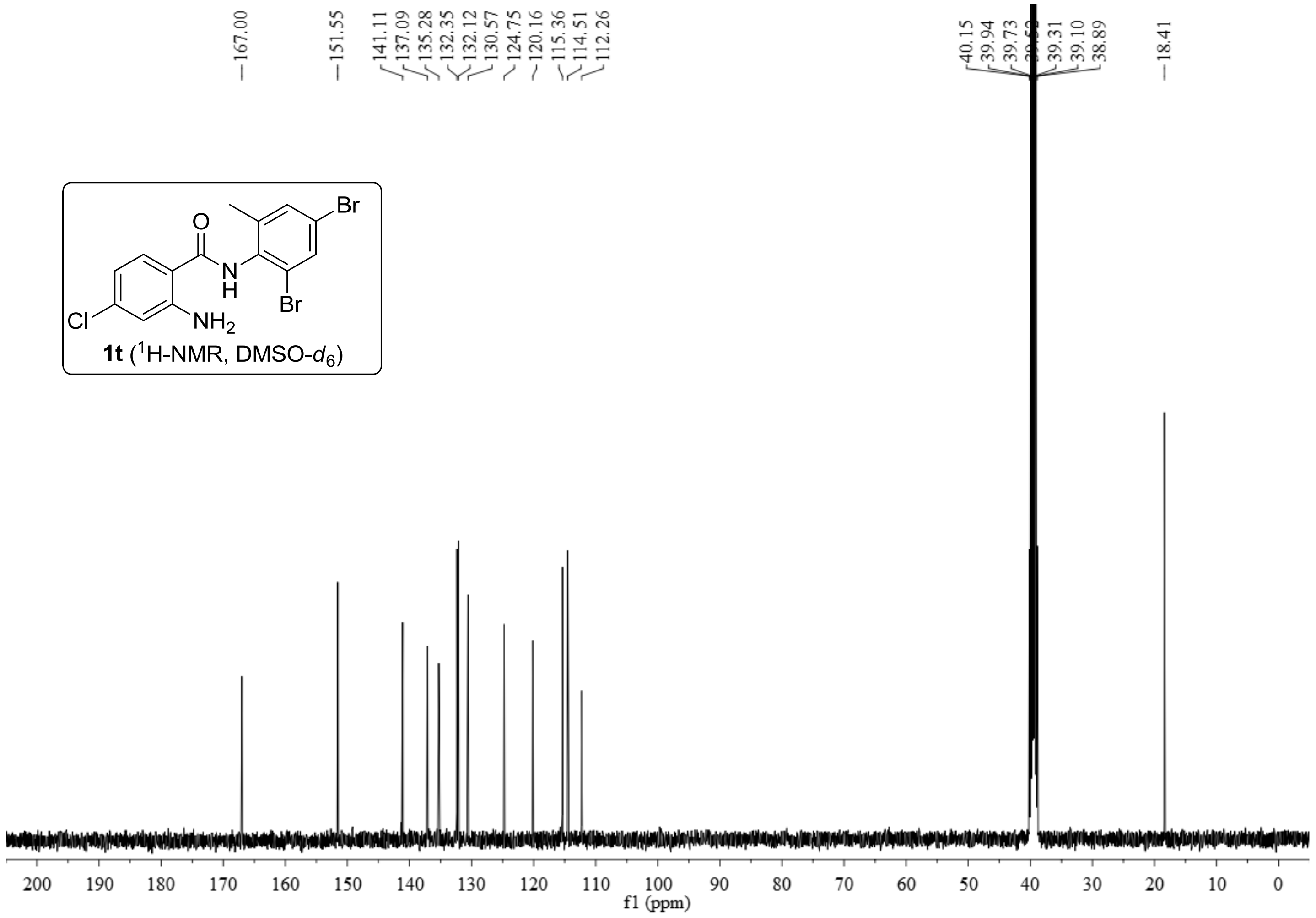


**Supplementary Figure 48.** $^{13}$C NMR of **1t**

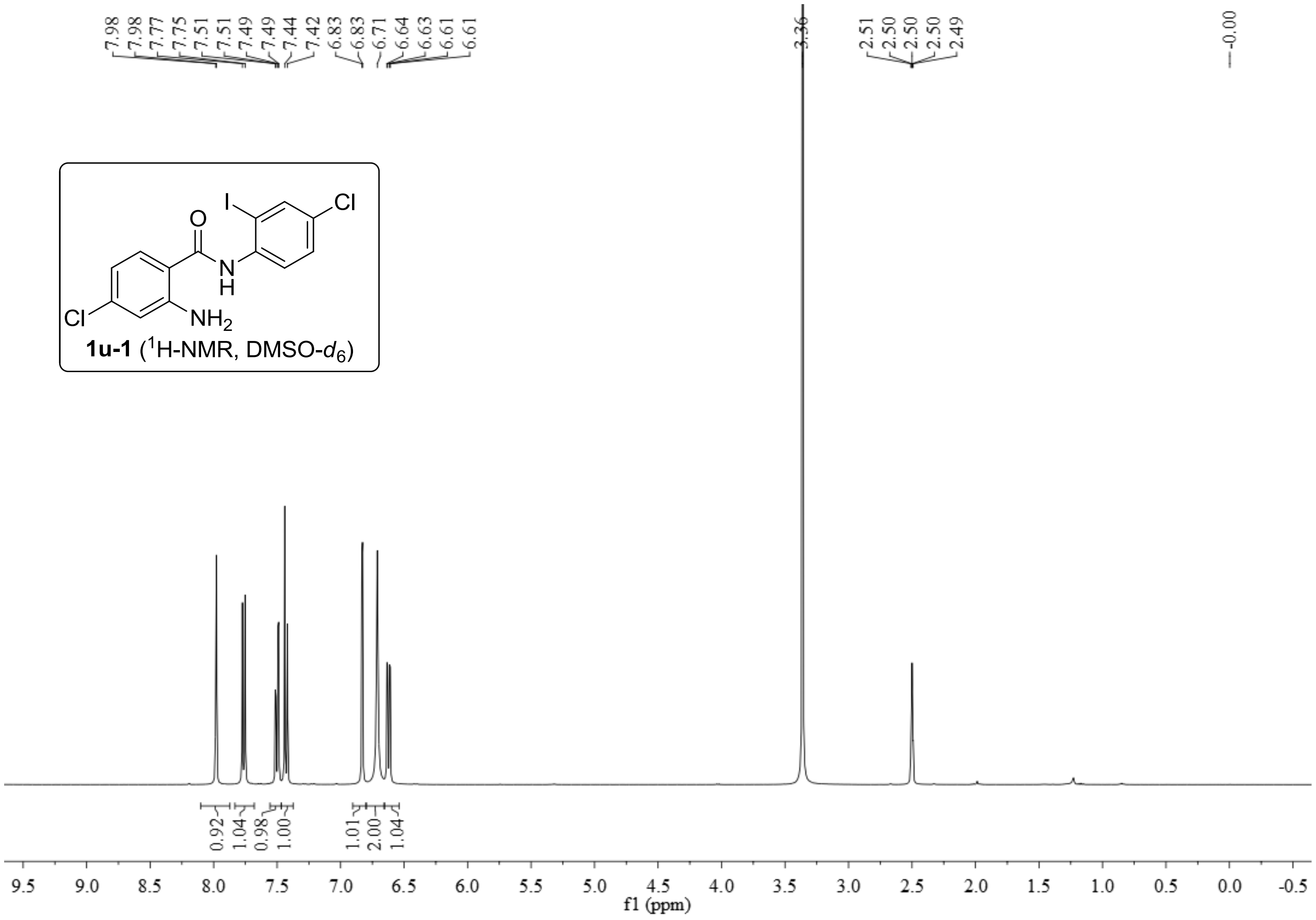


**Supplementary Figure 49.** $^{1}$H NMR of **1u-1**

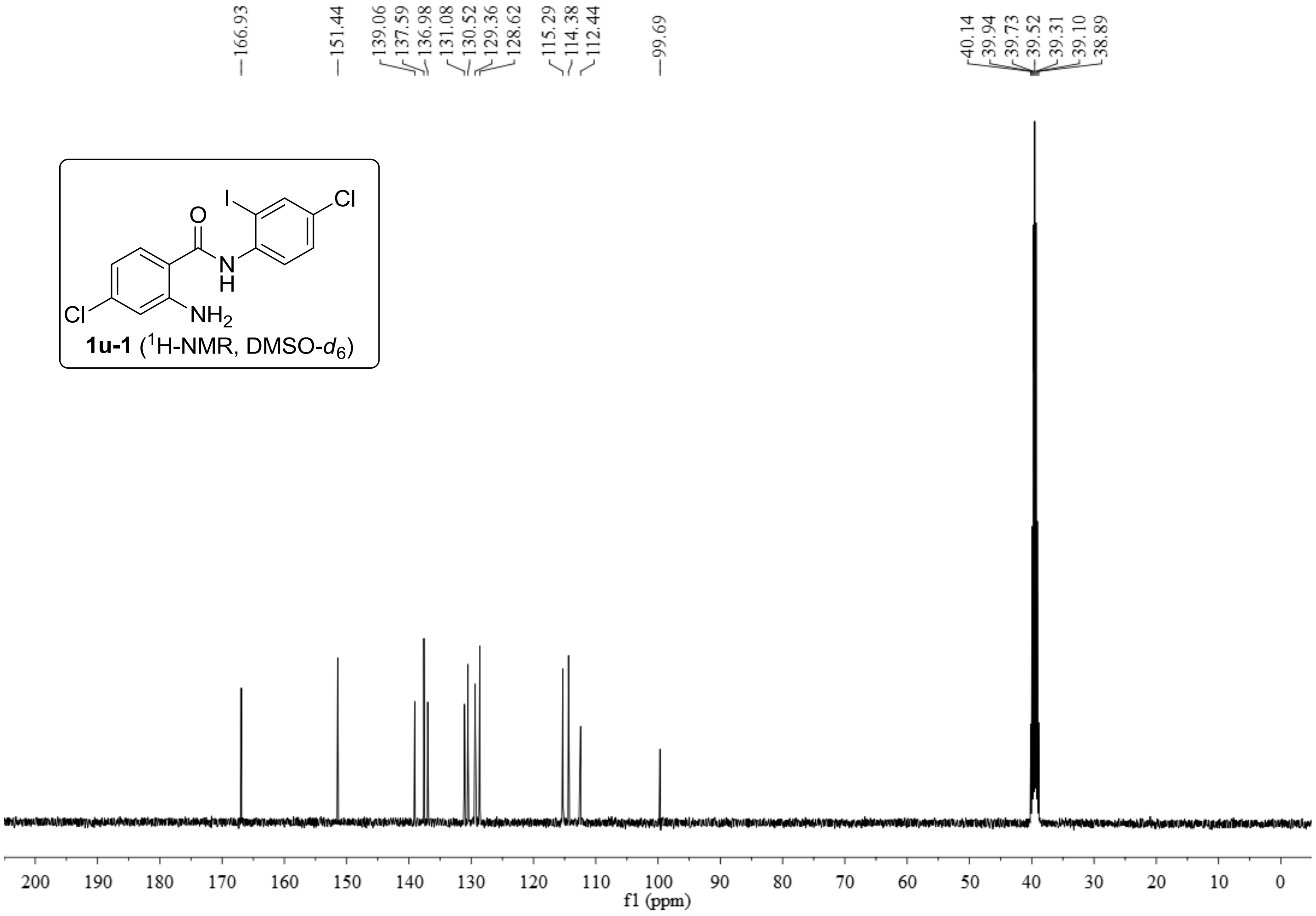


**Supplementary Figure 50.** $^{13}$C NMR of **1u-1**

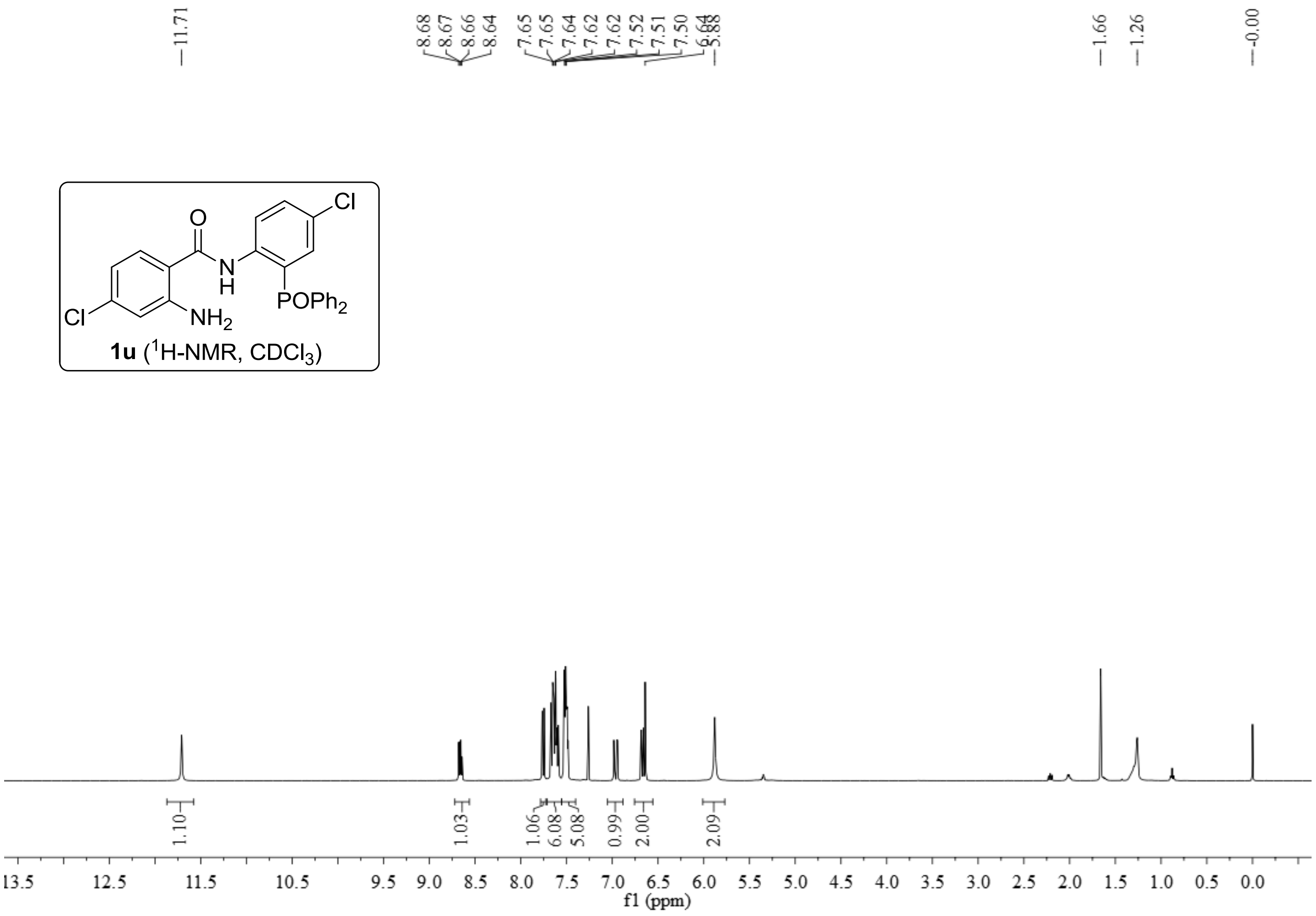


**Supplementary Figure 51.** $^{1}$H NMR of **1u**

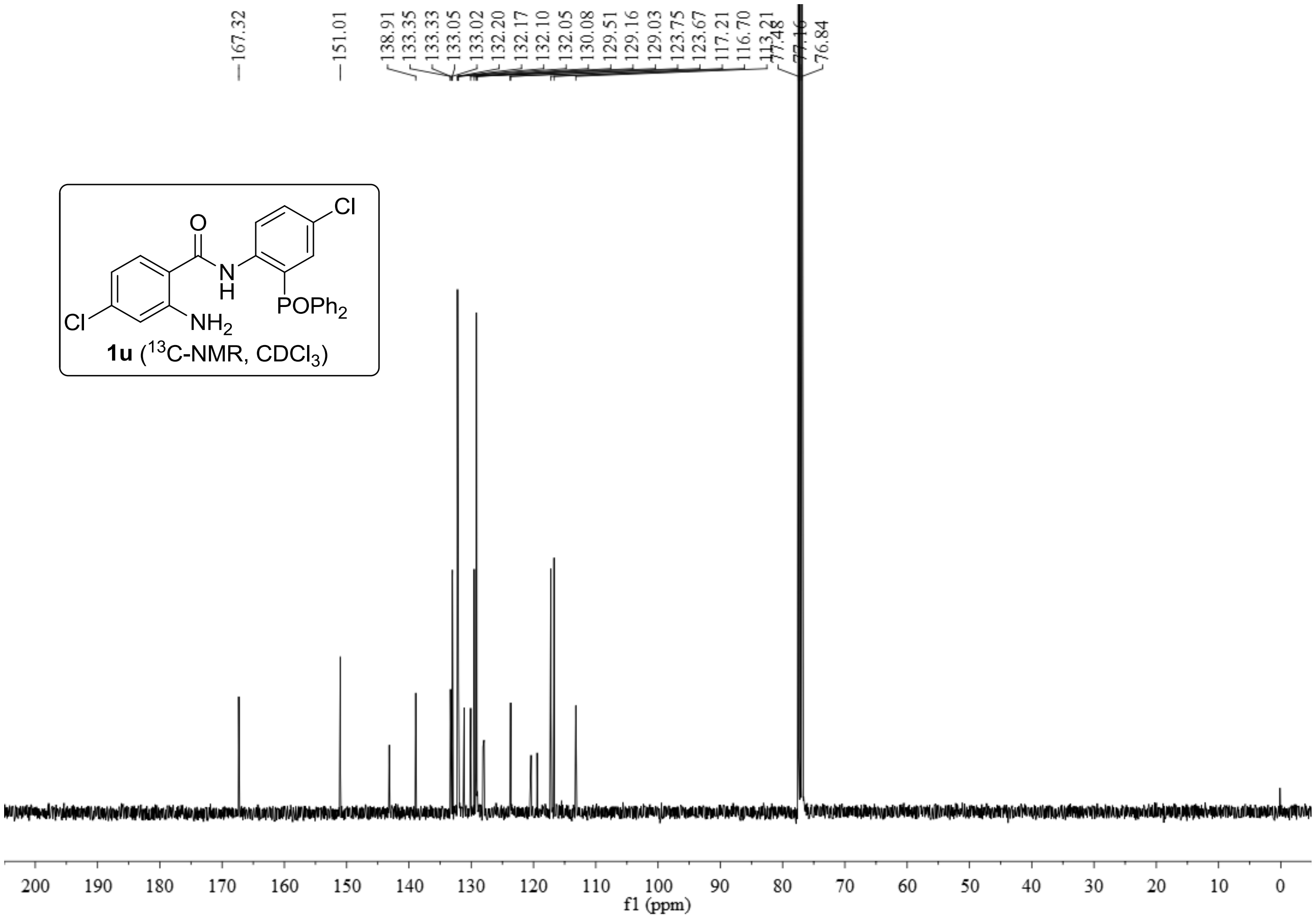


**Supplementary Figure 52.** $^{13}$C NMR of **1u**

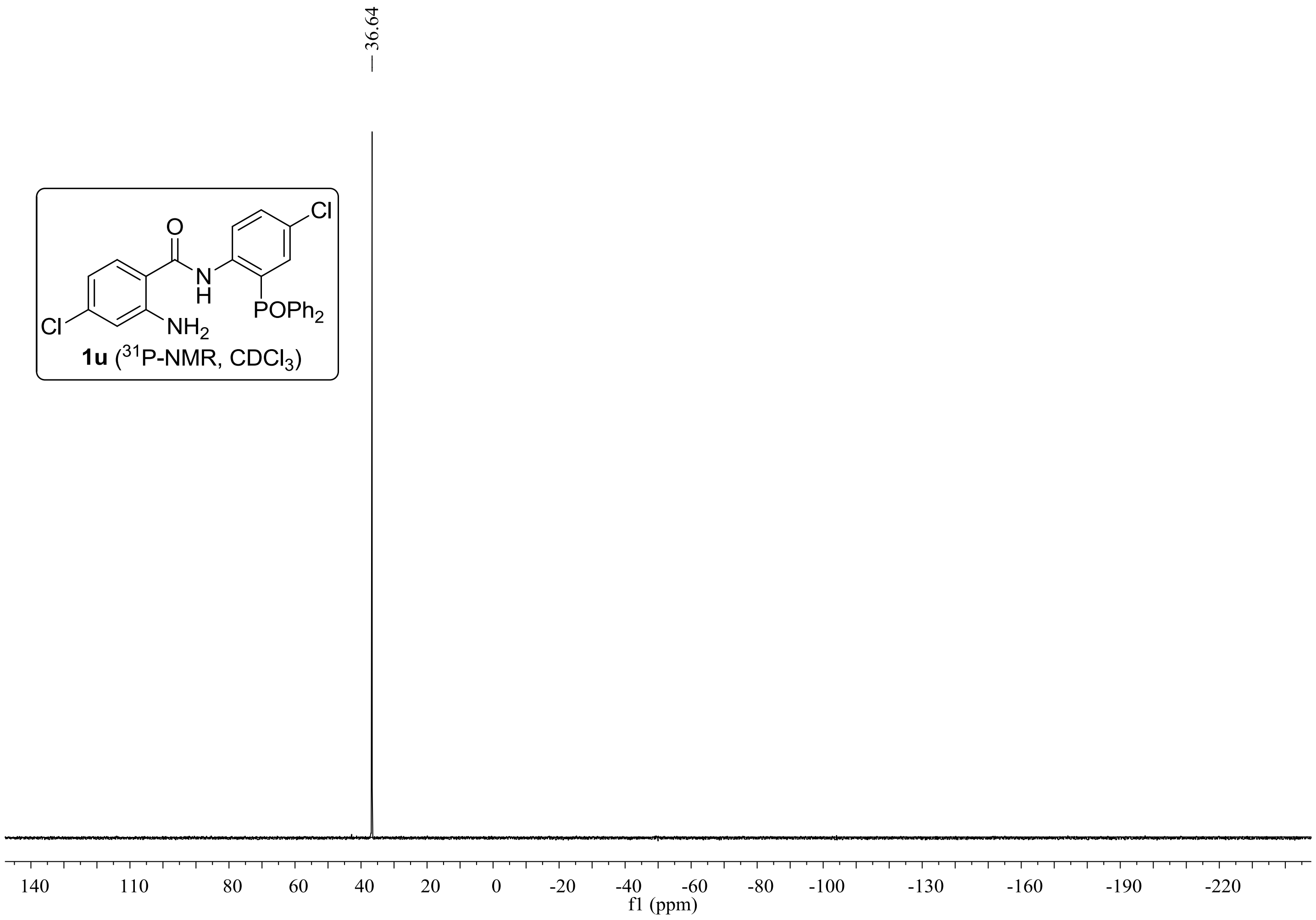


**Supplementary Figure 53.** $^{19}$F NMR of **1u**

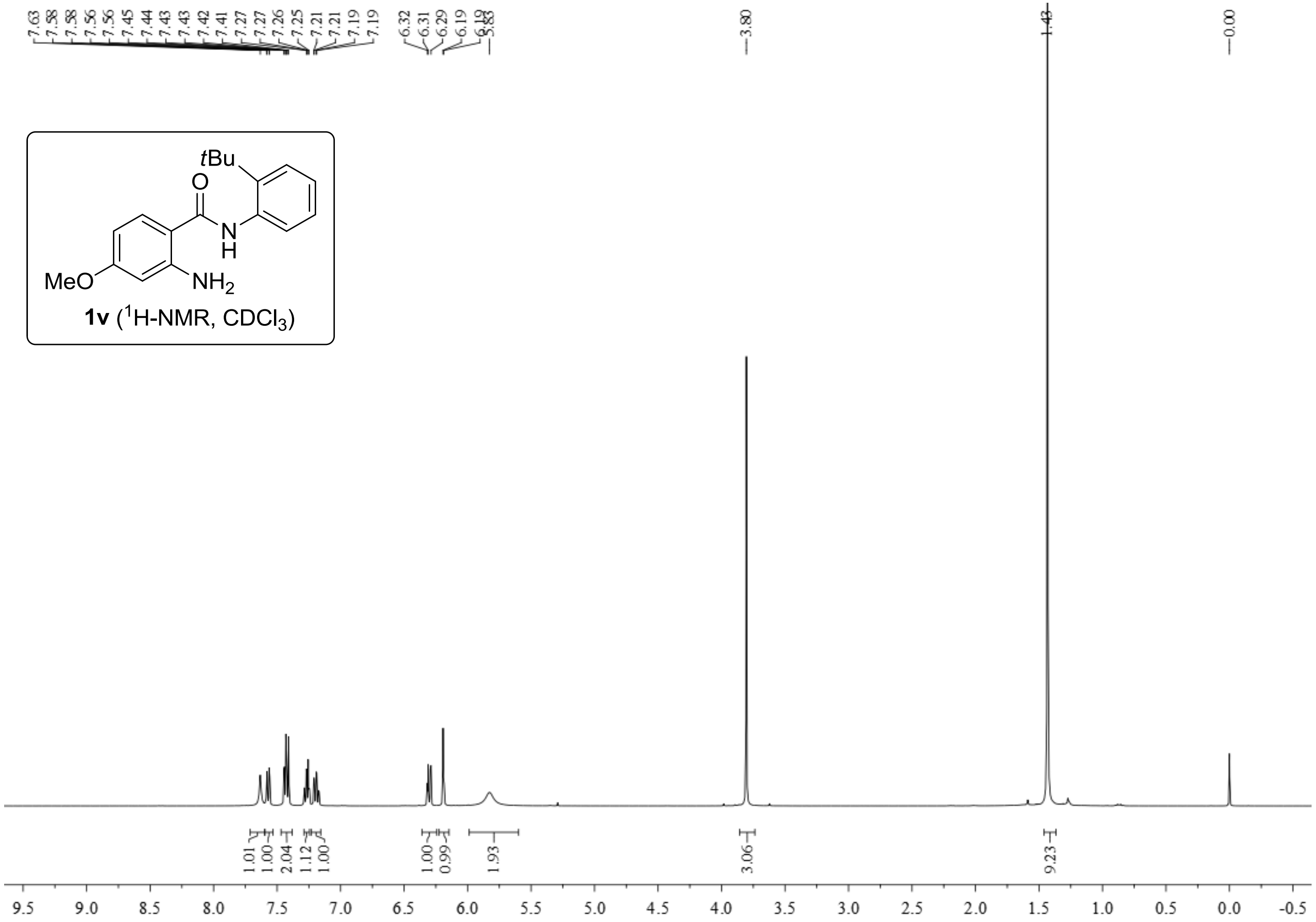


**Supplementary Figure 54.** $^{1}$H NMR of **1v**

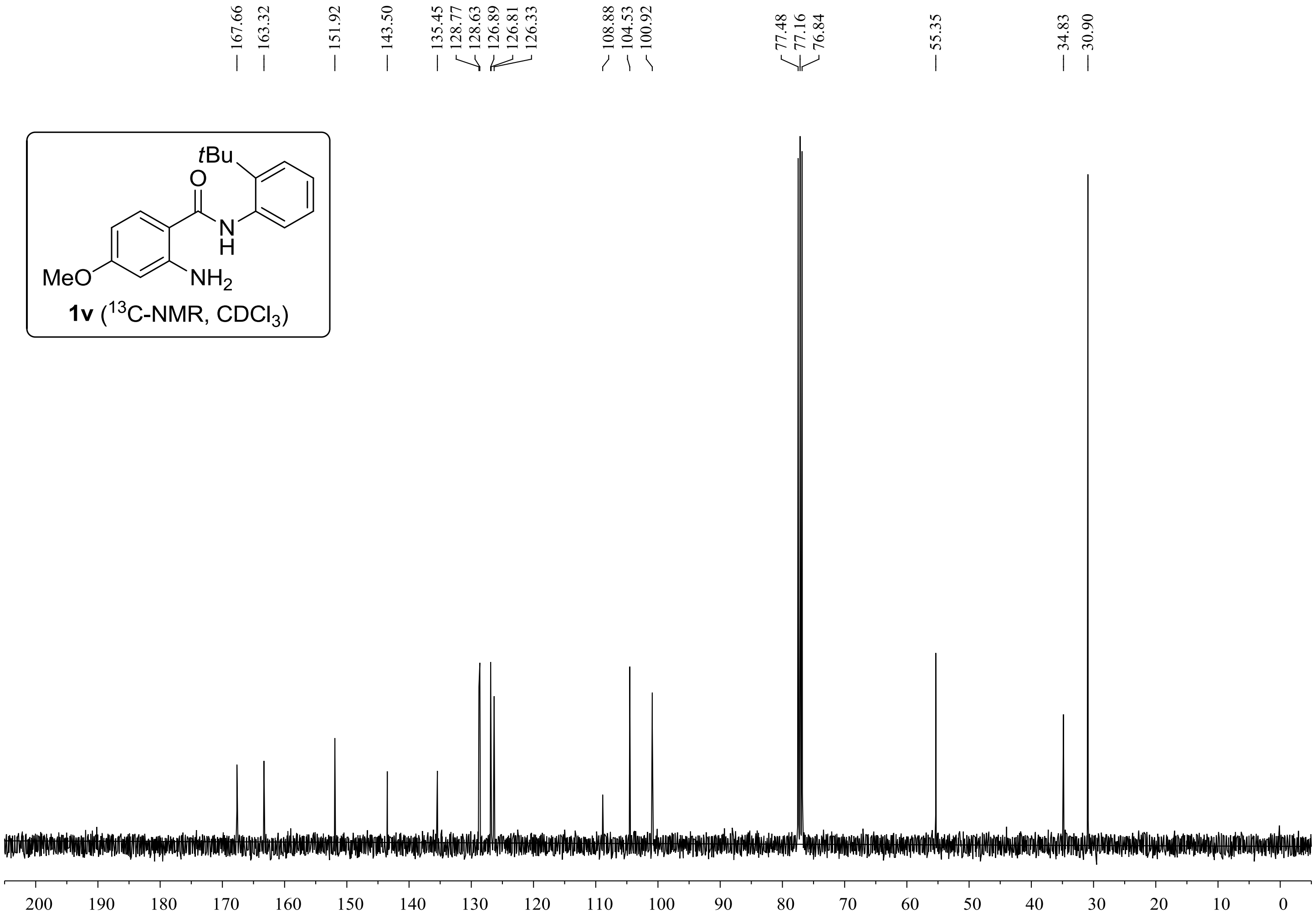


**Supplementary Figure 55.** $^{13}$C NMR of **1v**

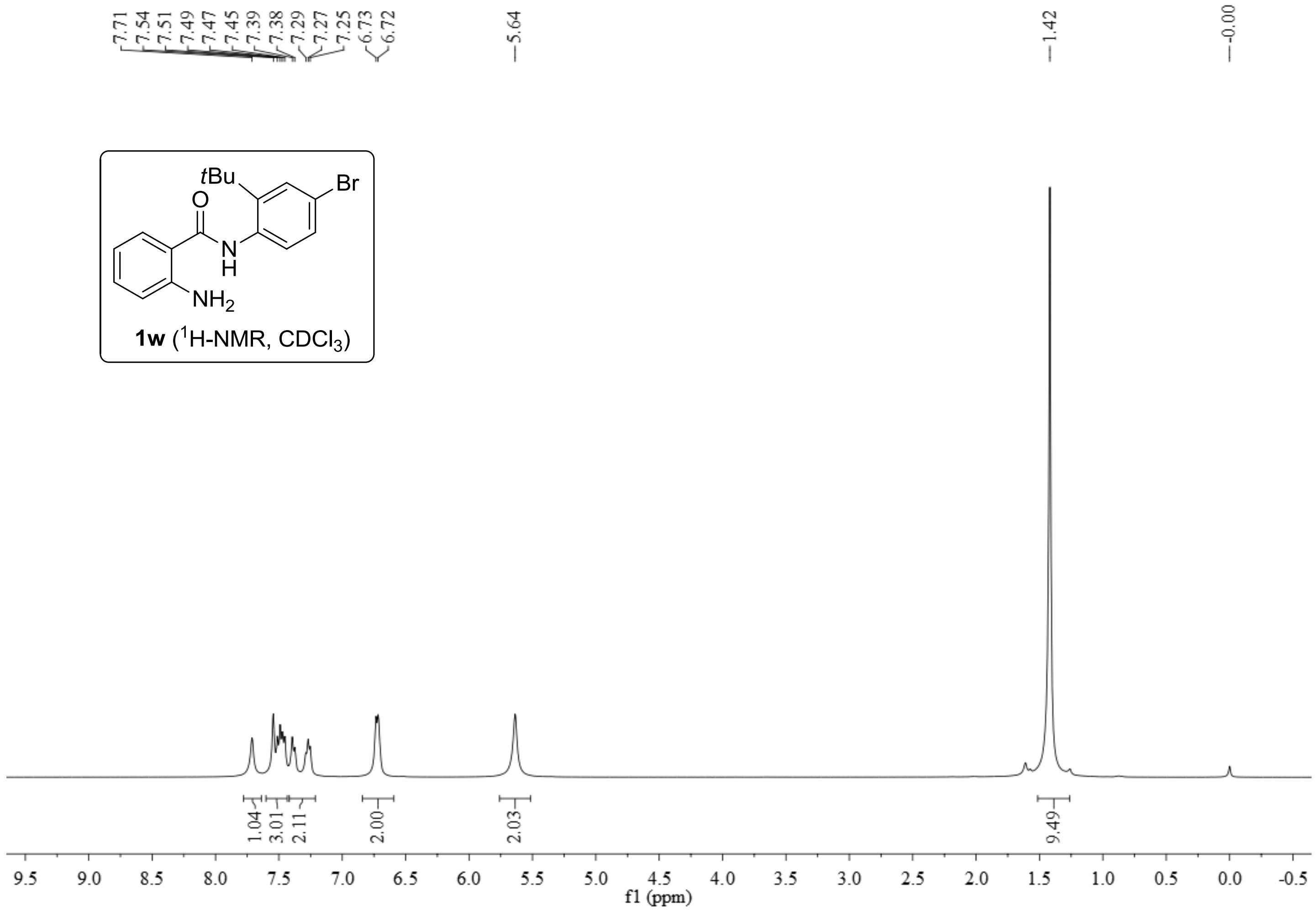


**Supplementary Figure 56.** $^{1}$H NMR of **1w**

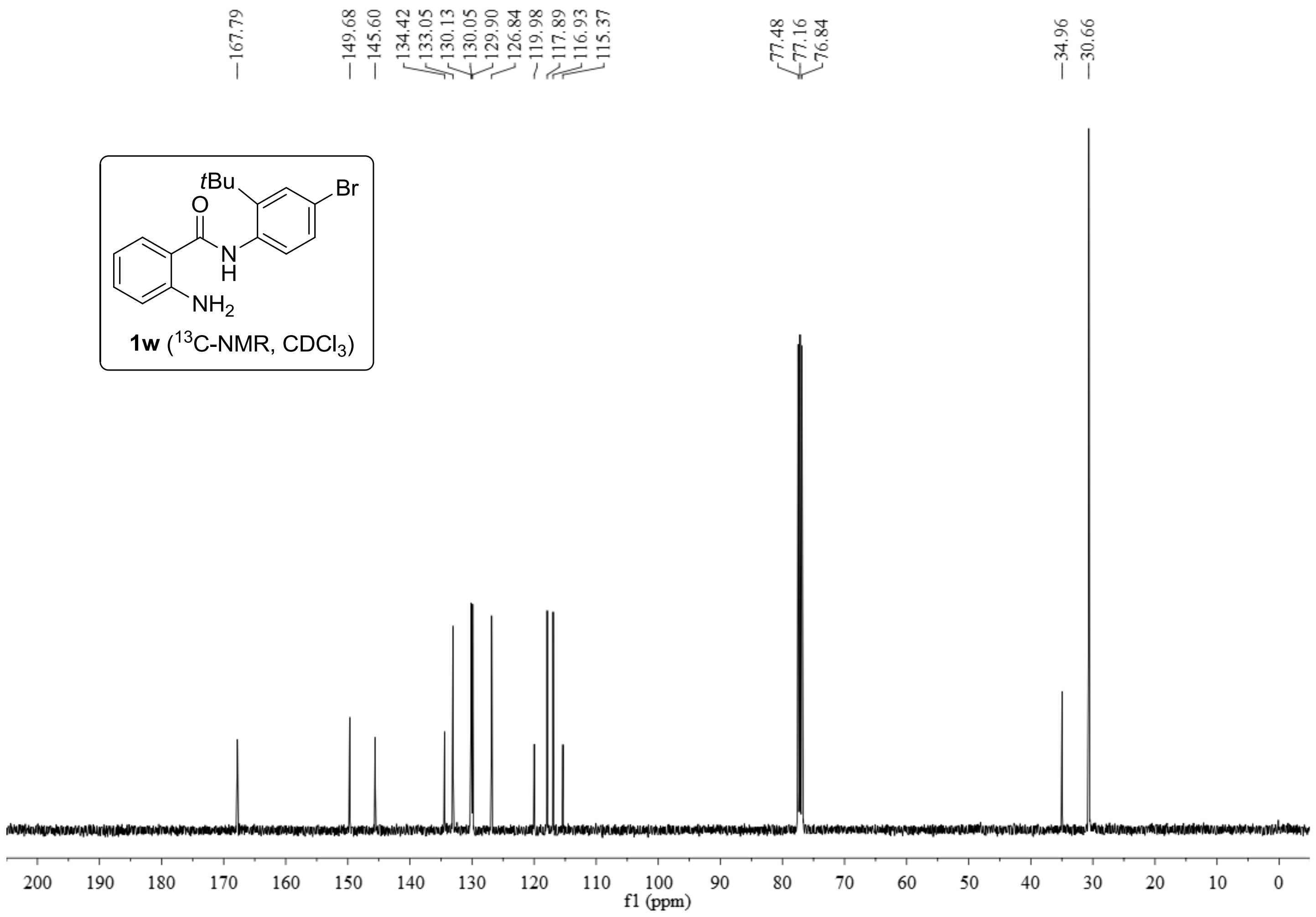


**Supplementary Figure 57.** $^{13}$C NMR of **1w**

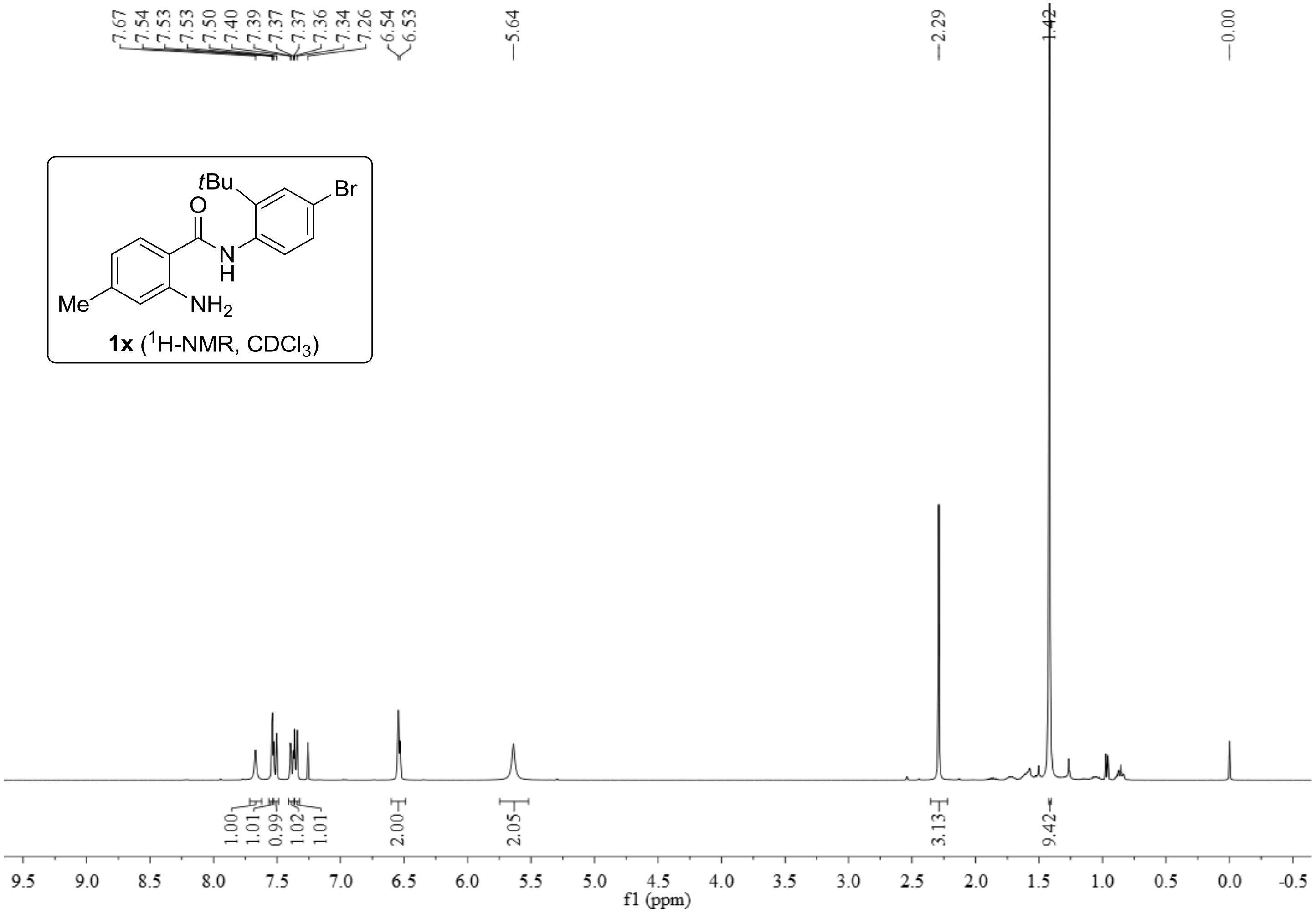


**Supplementary Figure 58.** $^{1}$H NMR of **1x**

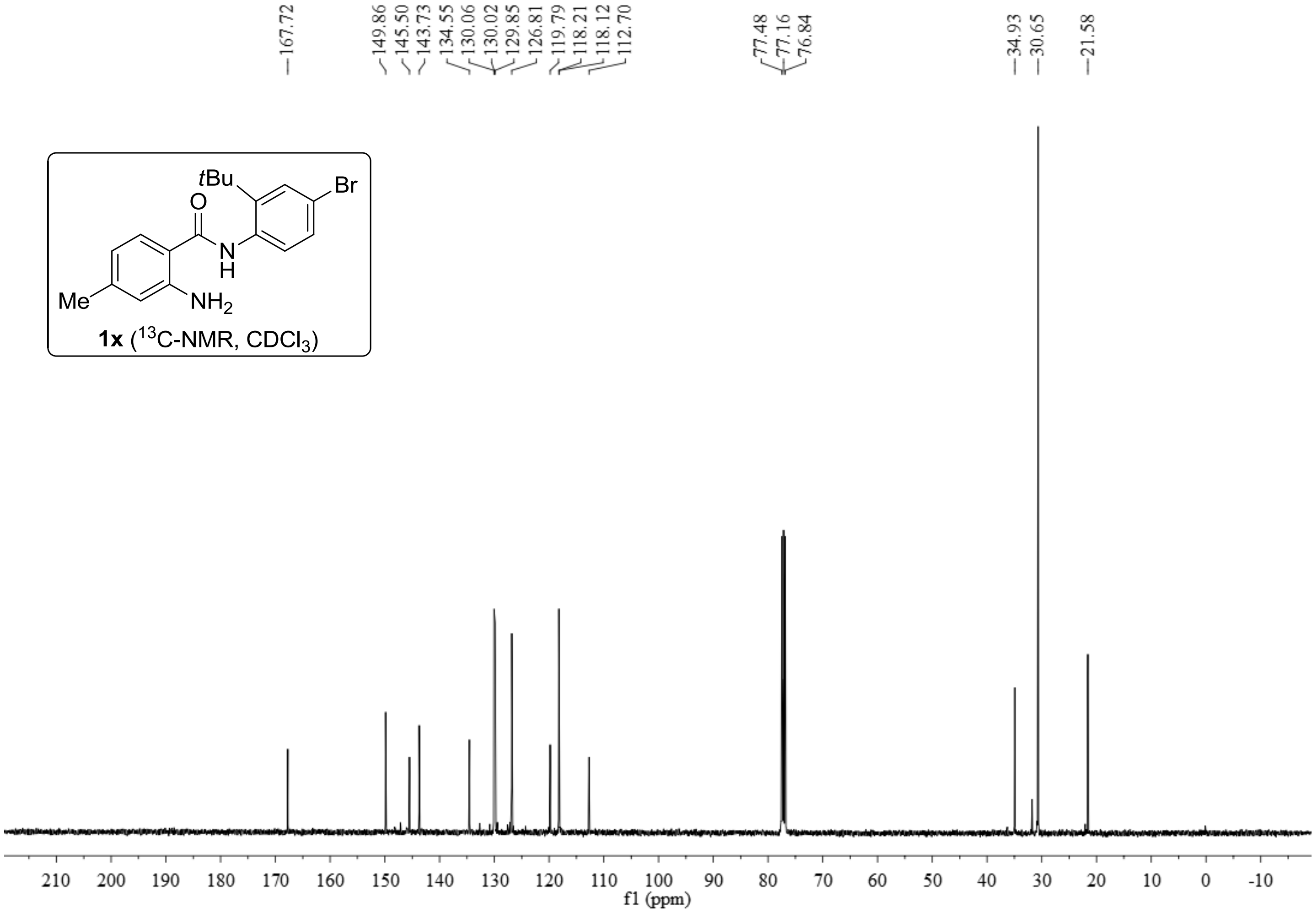


**Supplementary Figure 59.** $^{13}C$ NMR of **1x**

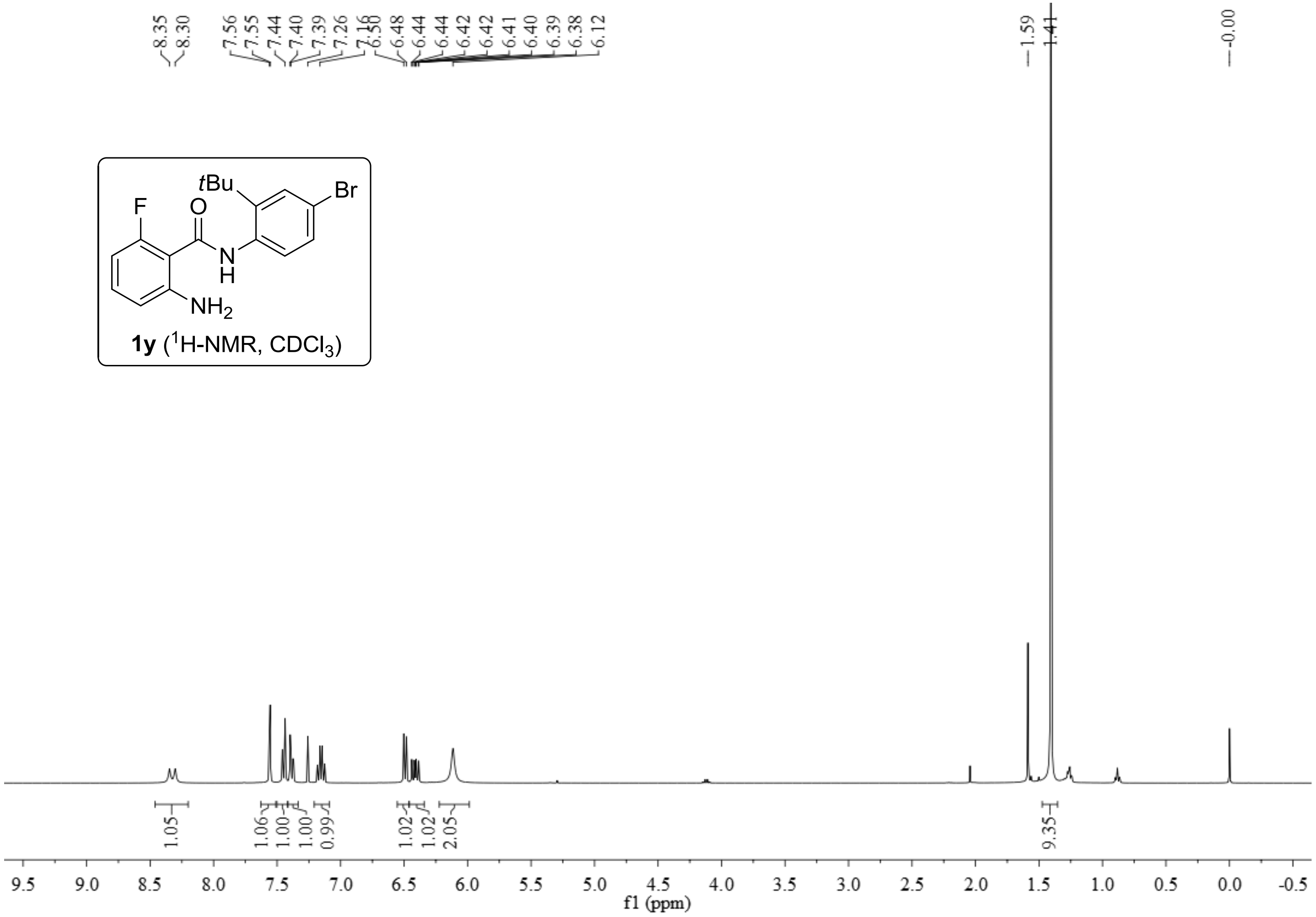


**Supplementary Figure 60.** $^1$H NMR of **1y**

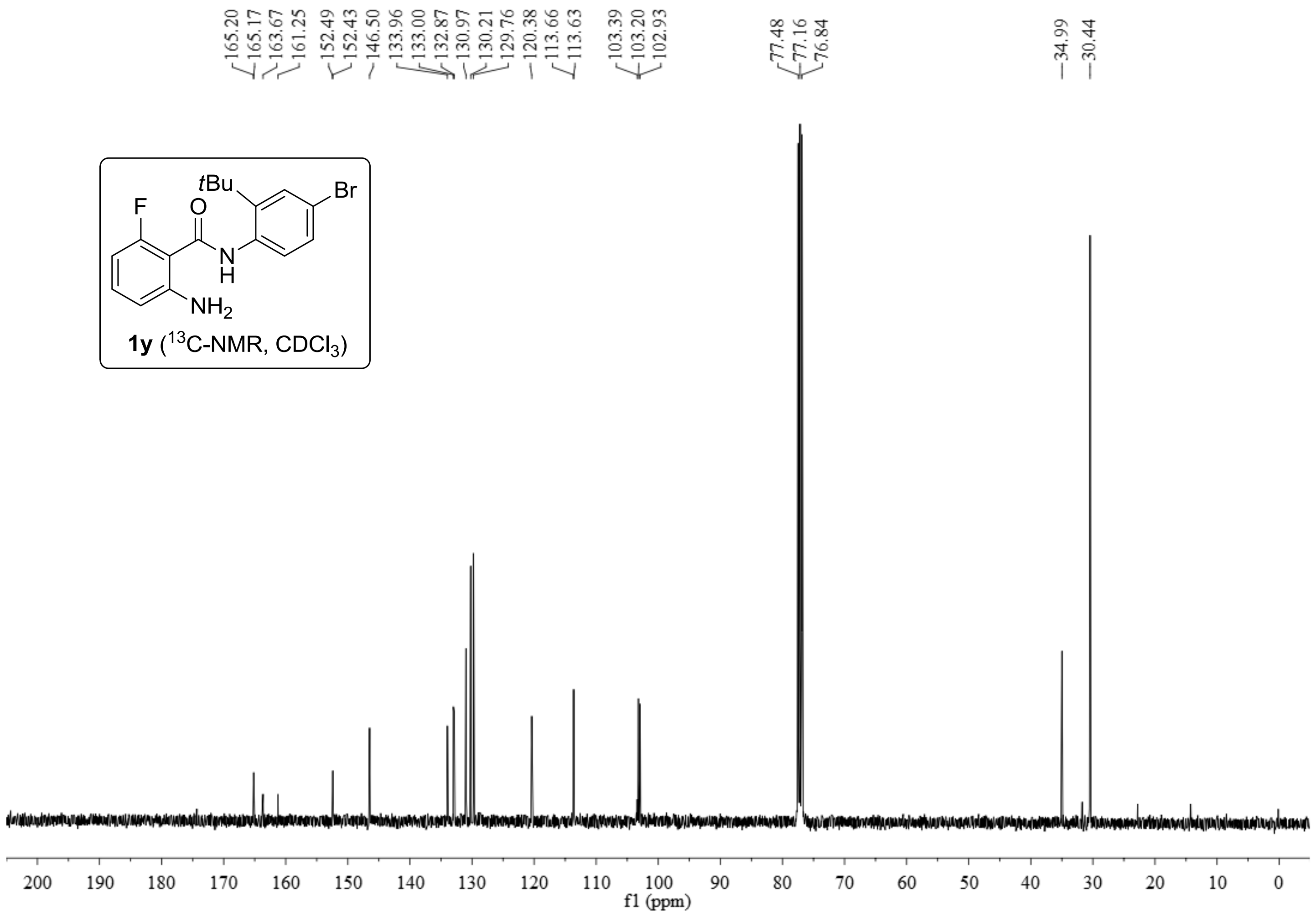


**Supplementary Figure 61.** $^{13}$C NMR of **1y**

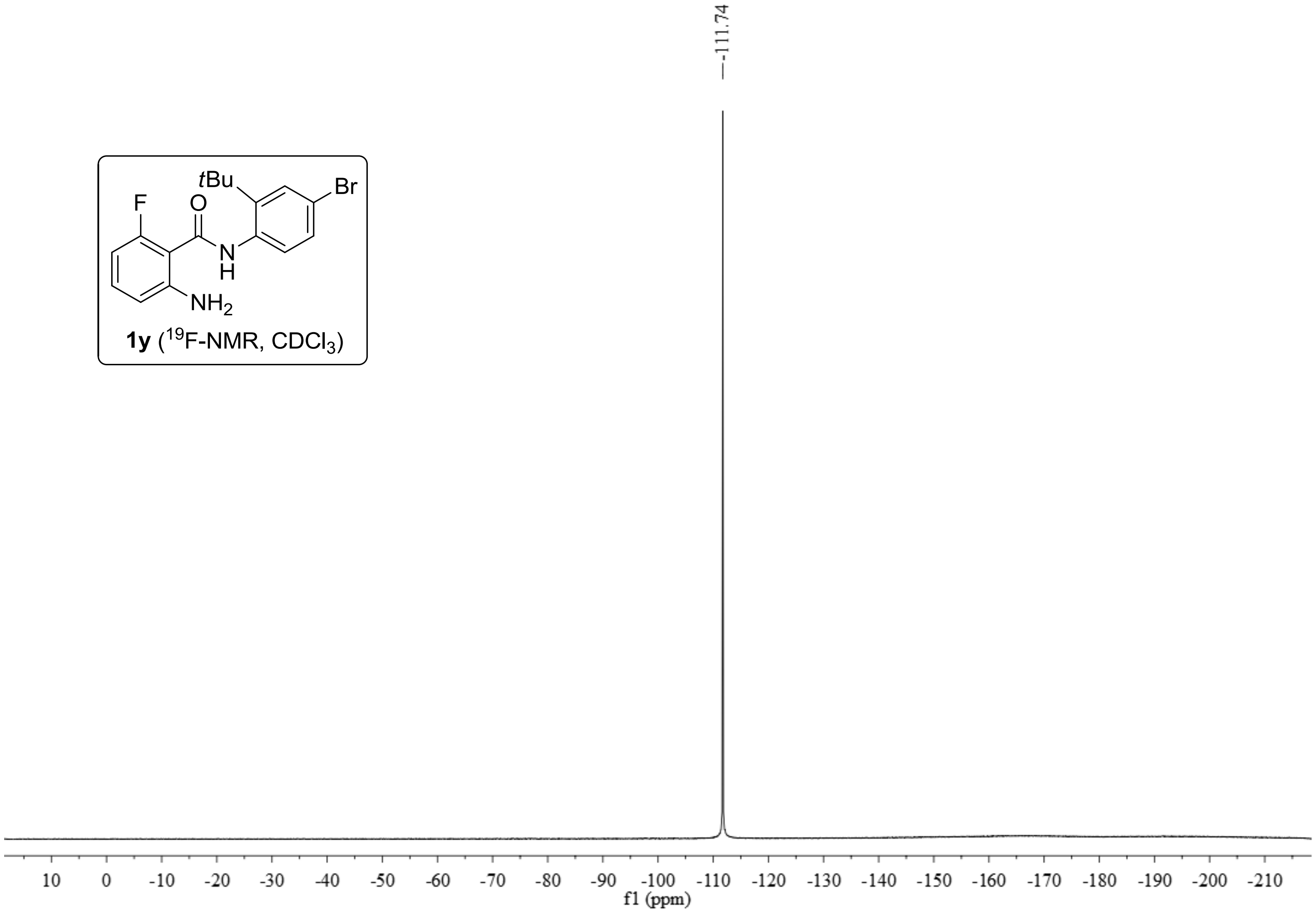


**Supplementary Figure 62.** $^{19}$F NMR of **1y**

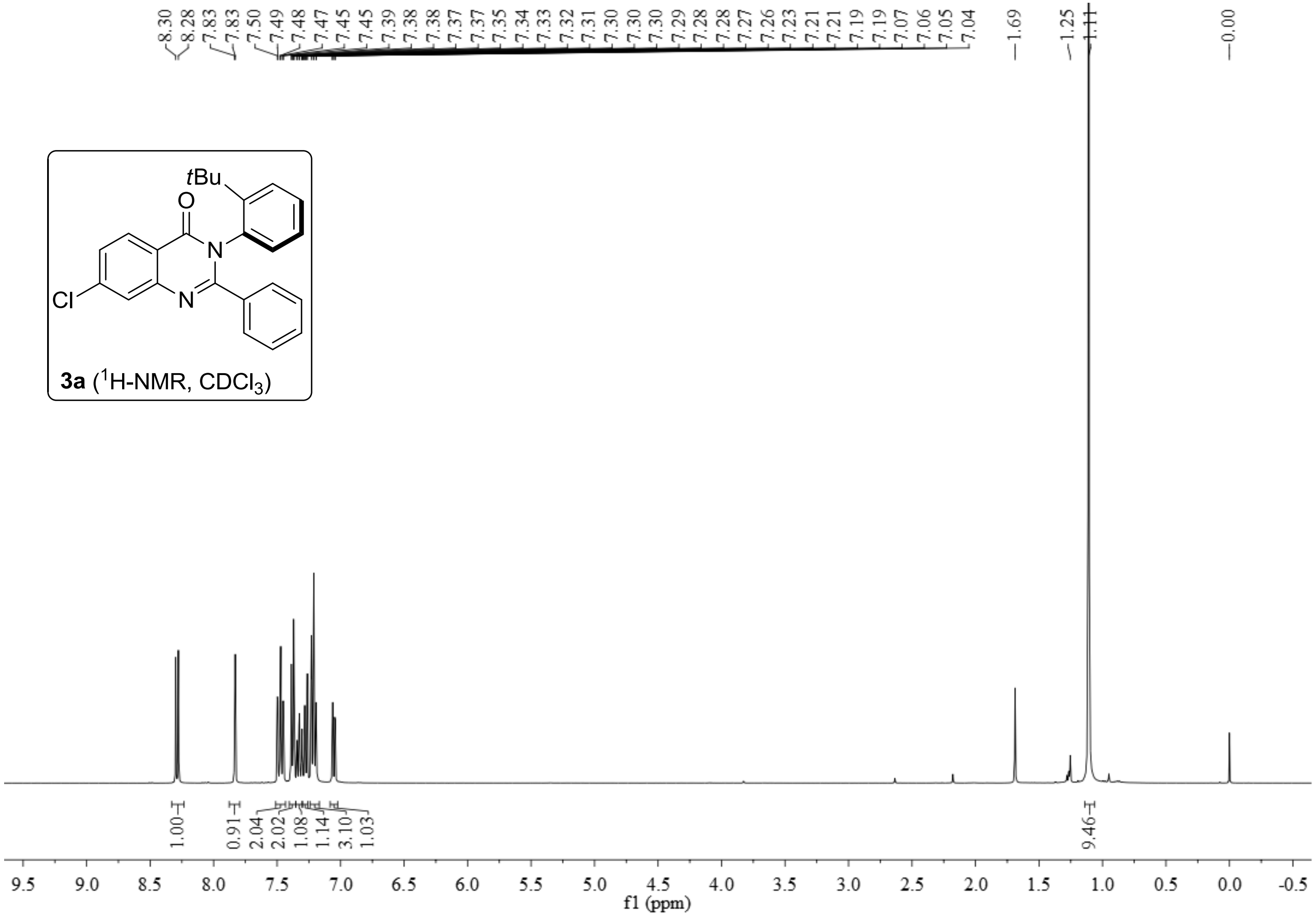


**Supplementary Figure 63.** ${}^{1}$H NMR of **3a**

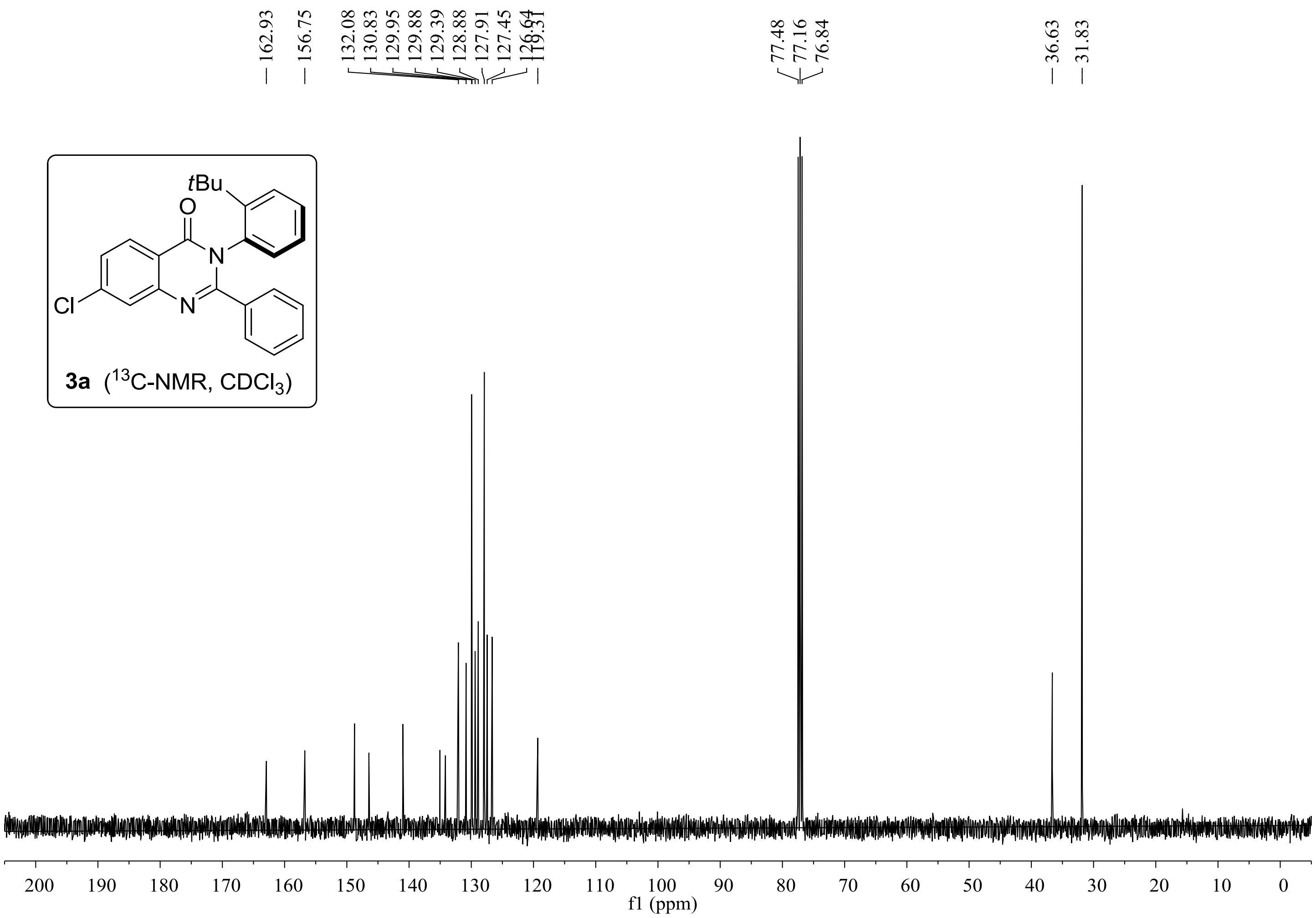


**Supplementary Figure 64.** $^{13}$C NMR of **3a**

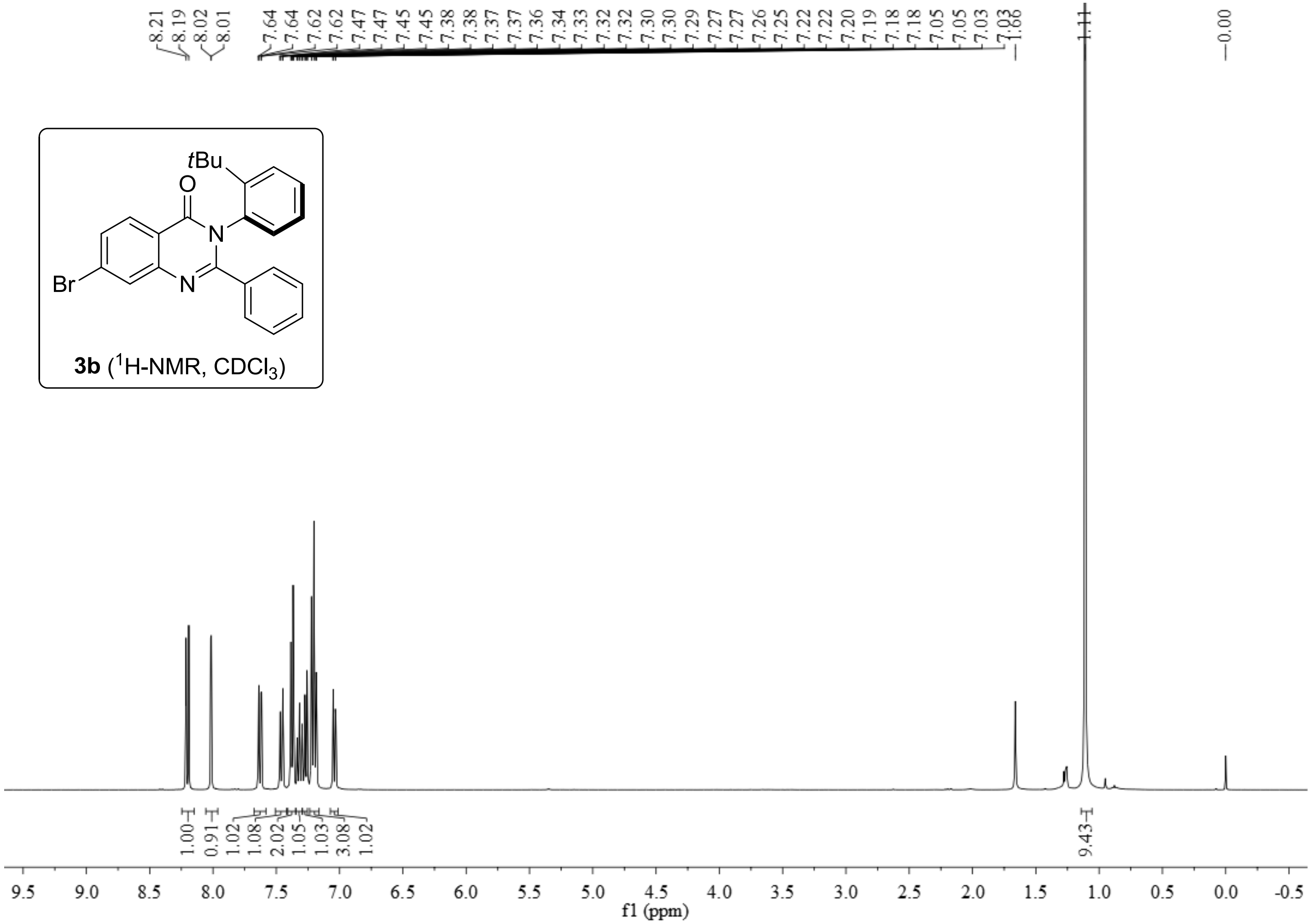


**Supplementary Figure 65.** $^{1}$H NMR of **3b**

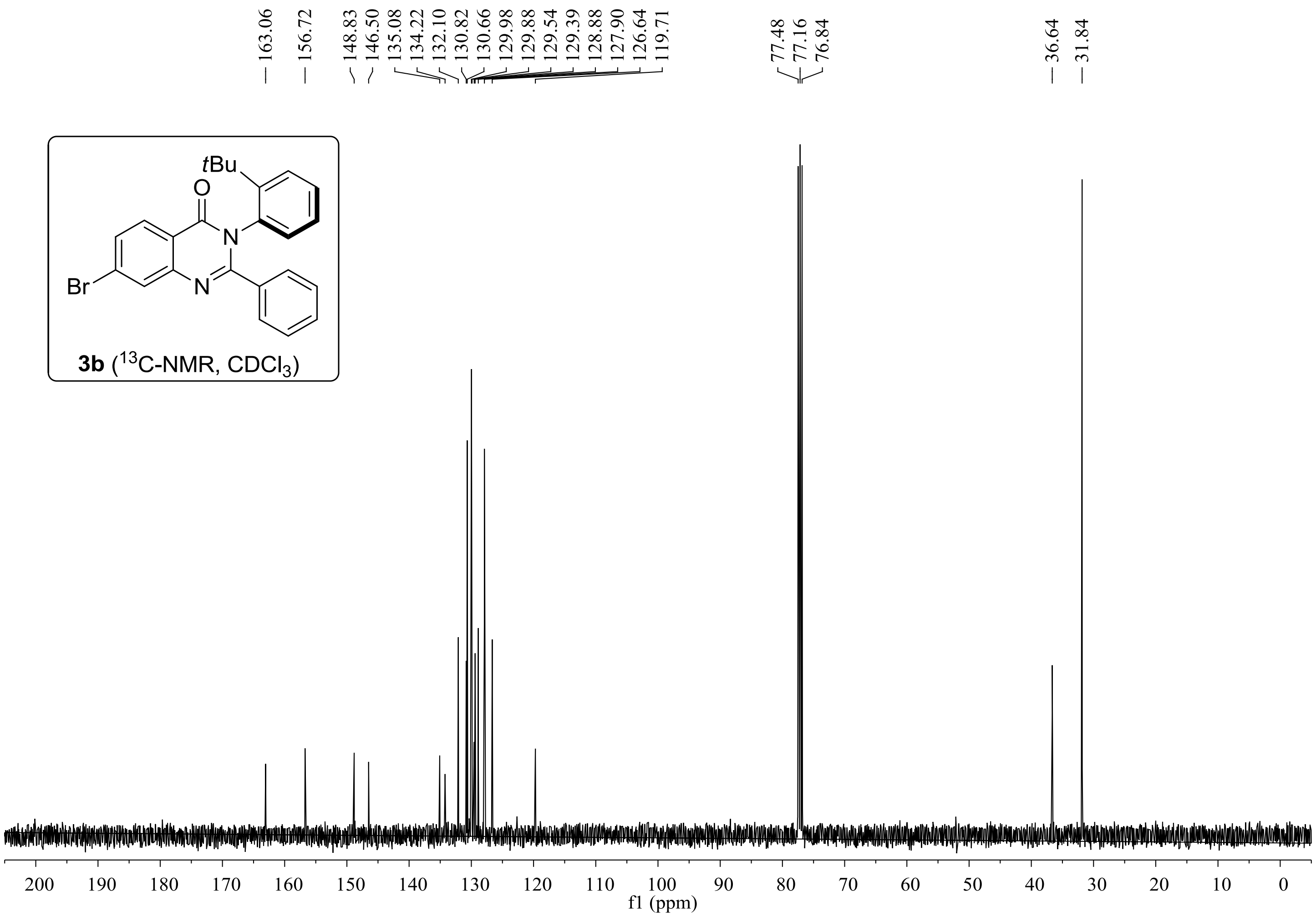


**Supplementary Figure 66.** $^{13}$C NMR of **3b**

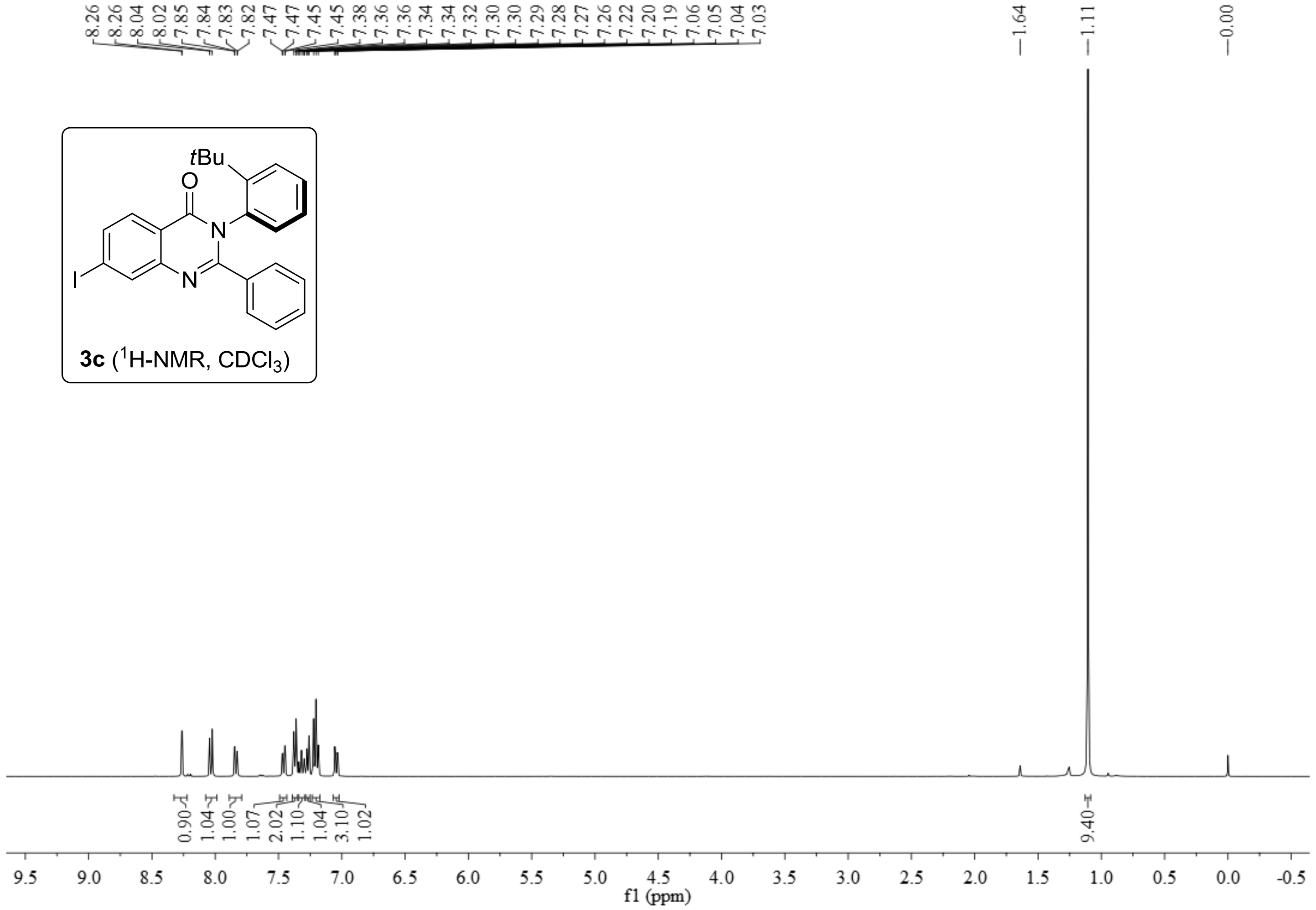


**Supplementary Figure 67.** $^1$H NMR of **3c**

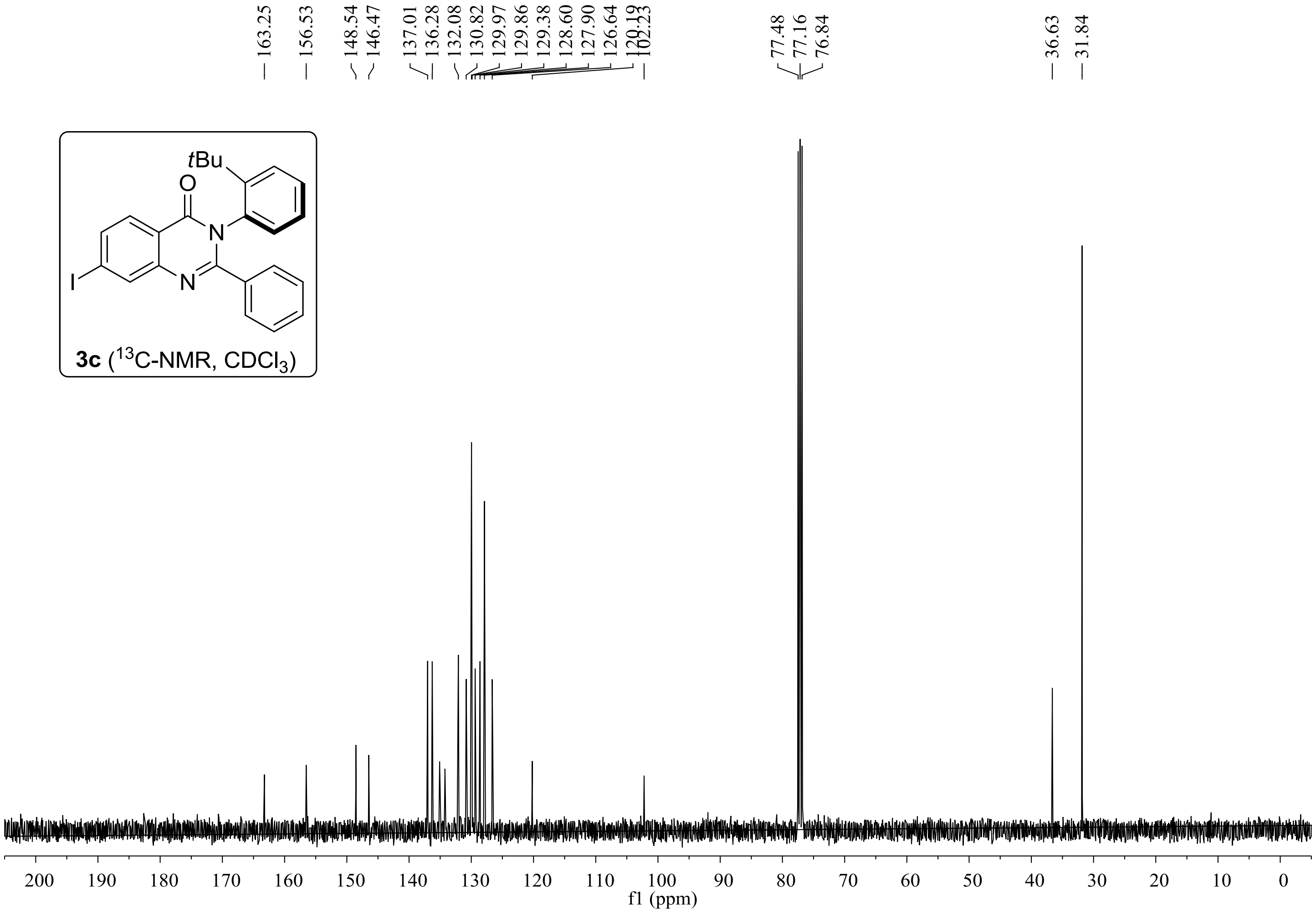


**Supplementary Figure 68.** $^{13}$C NMR of **3c**

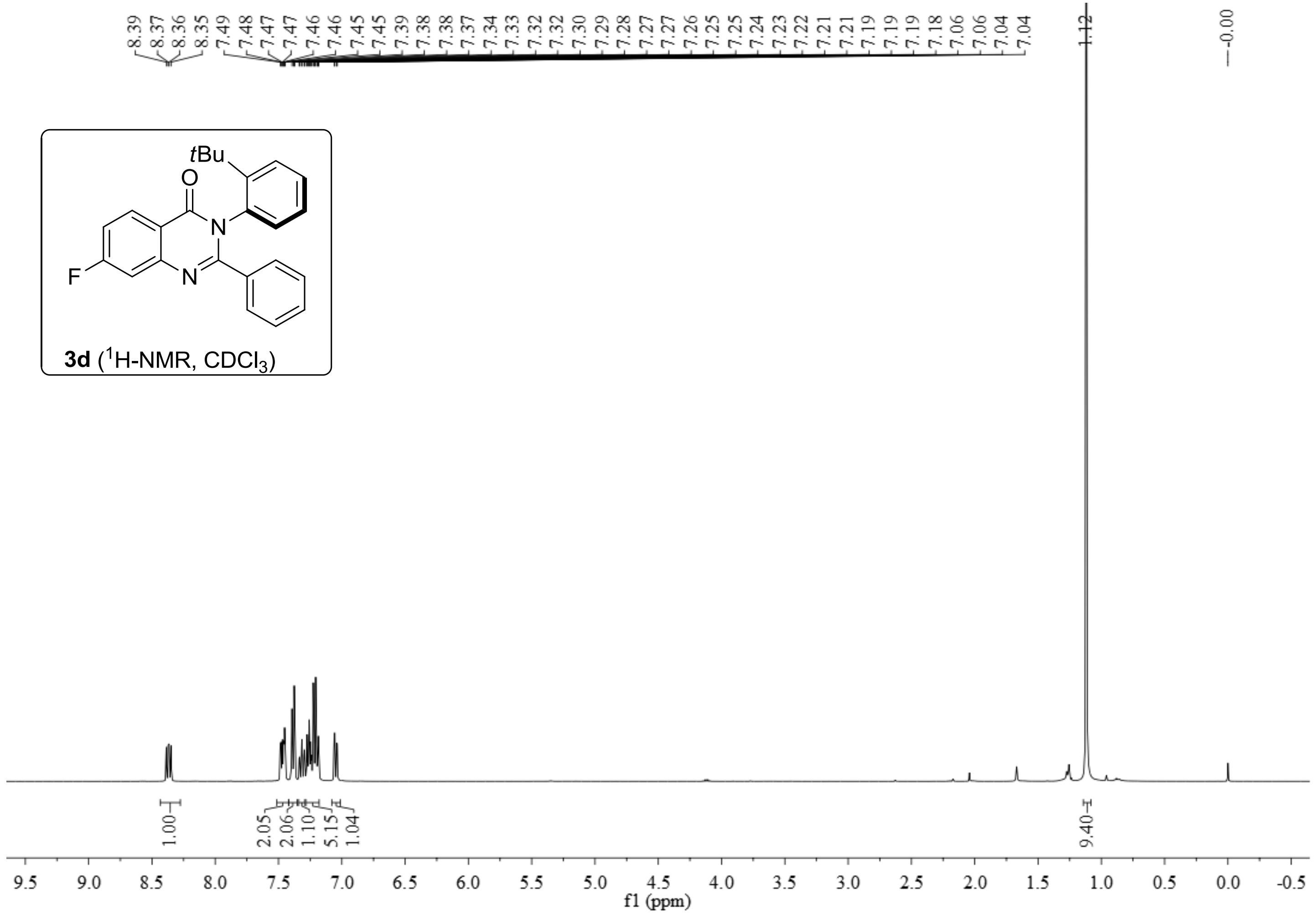


**Supplementary Figure 69.** $^{1}$H NMR of **3d**

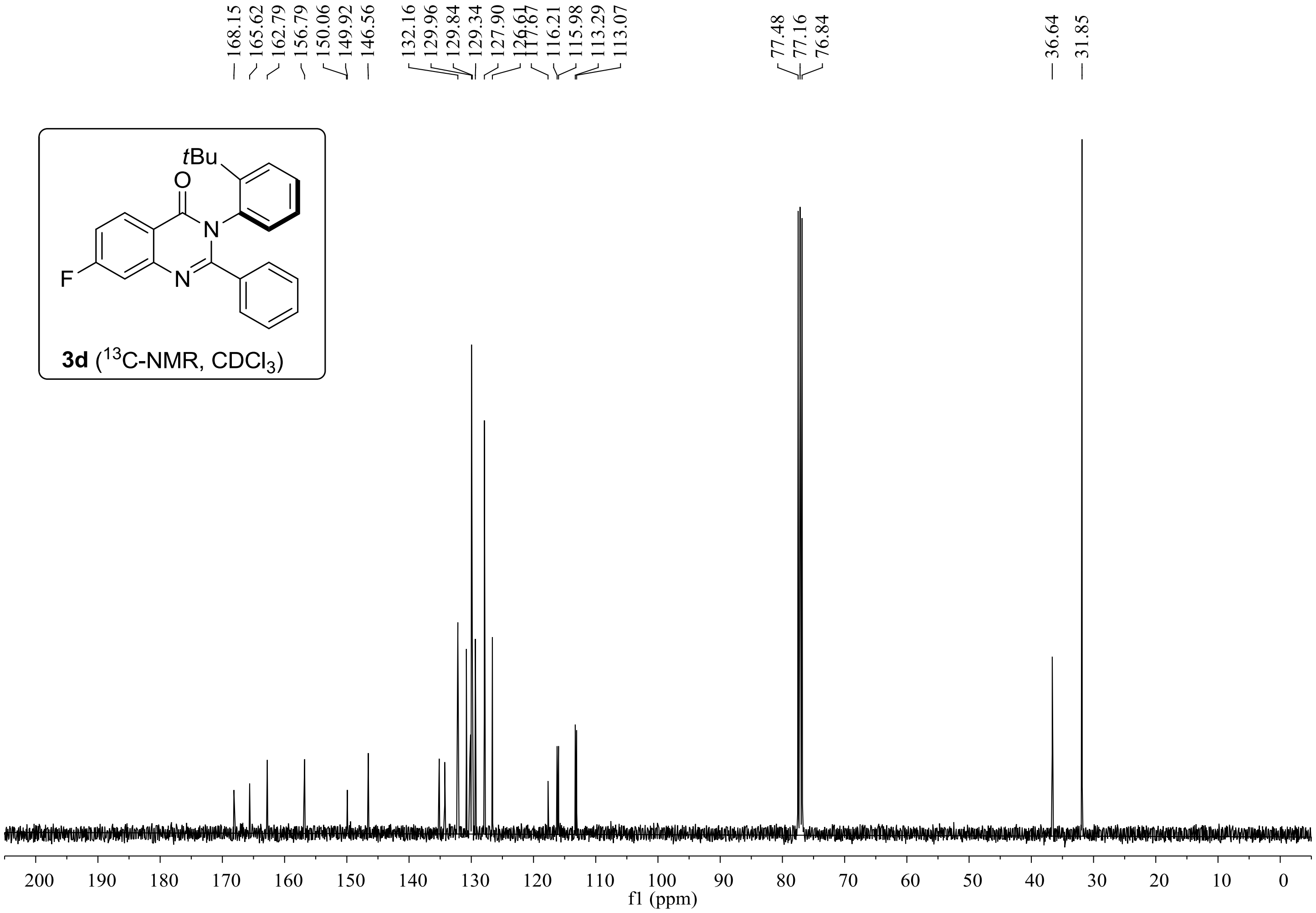


**Supplementary Figure 70.** $^{13}$C NMR of **3d**

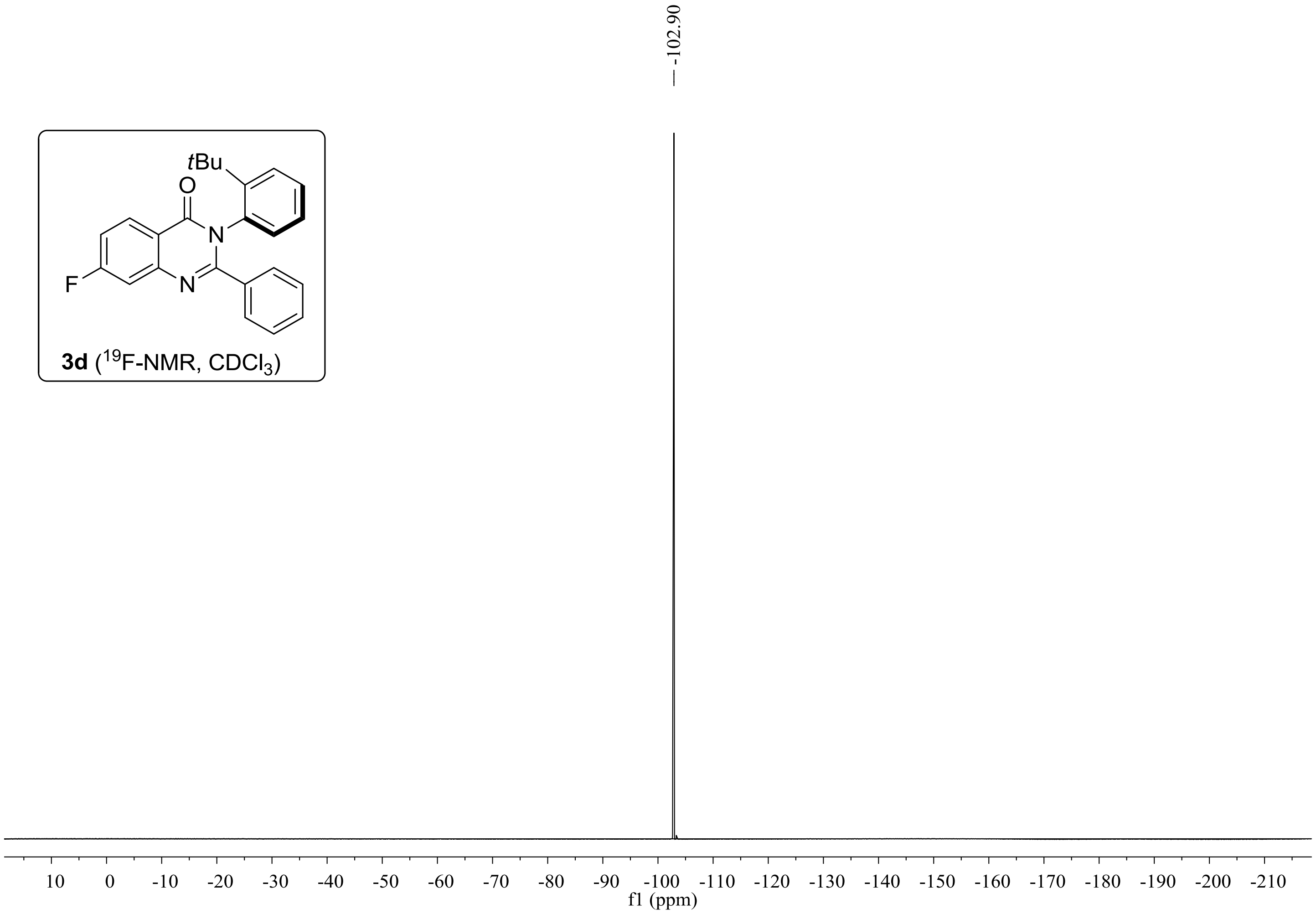


**Supplementary Figure 71.** $^{19}$F NMR of **3d**

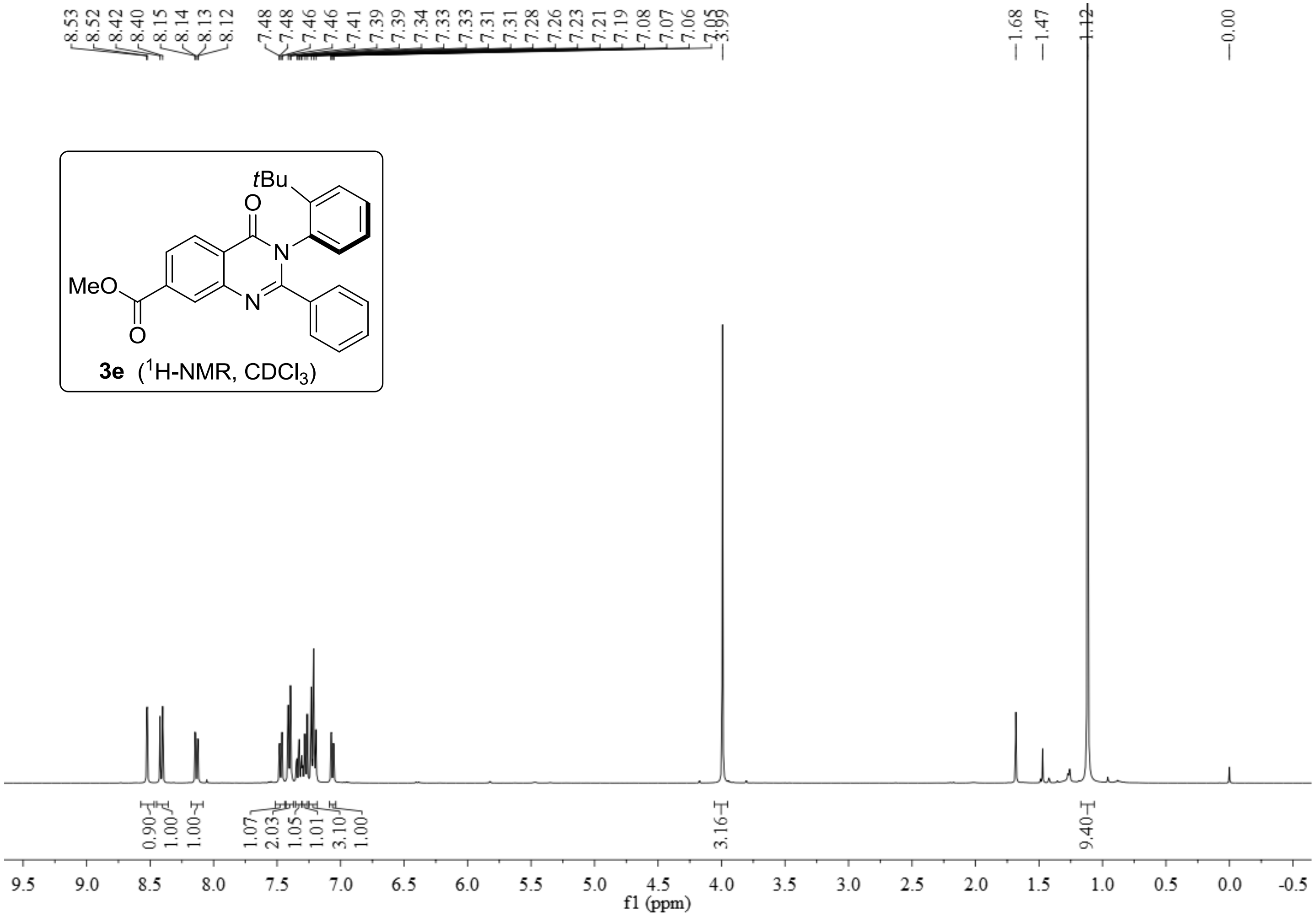


**Supplementary Figure 72.** $^{1}$H NMR of **3e**

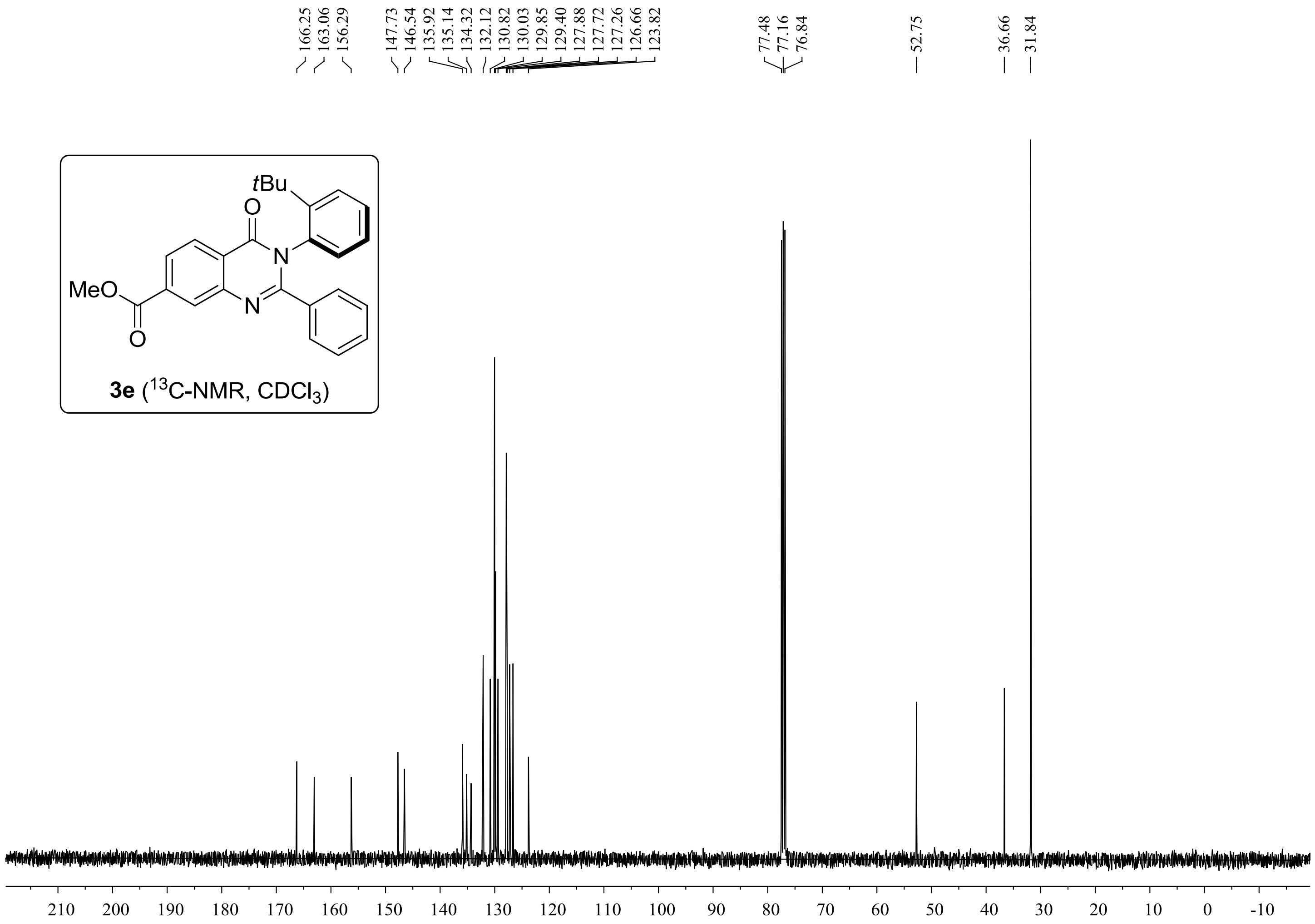


**Supplementary Figure 73.** $^{13}$C NMR of **1h**

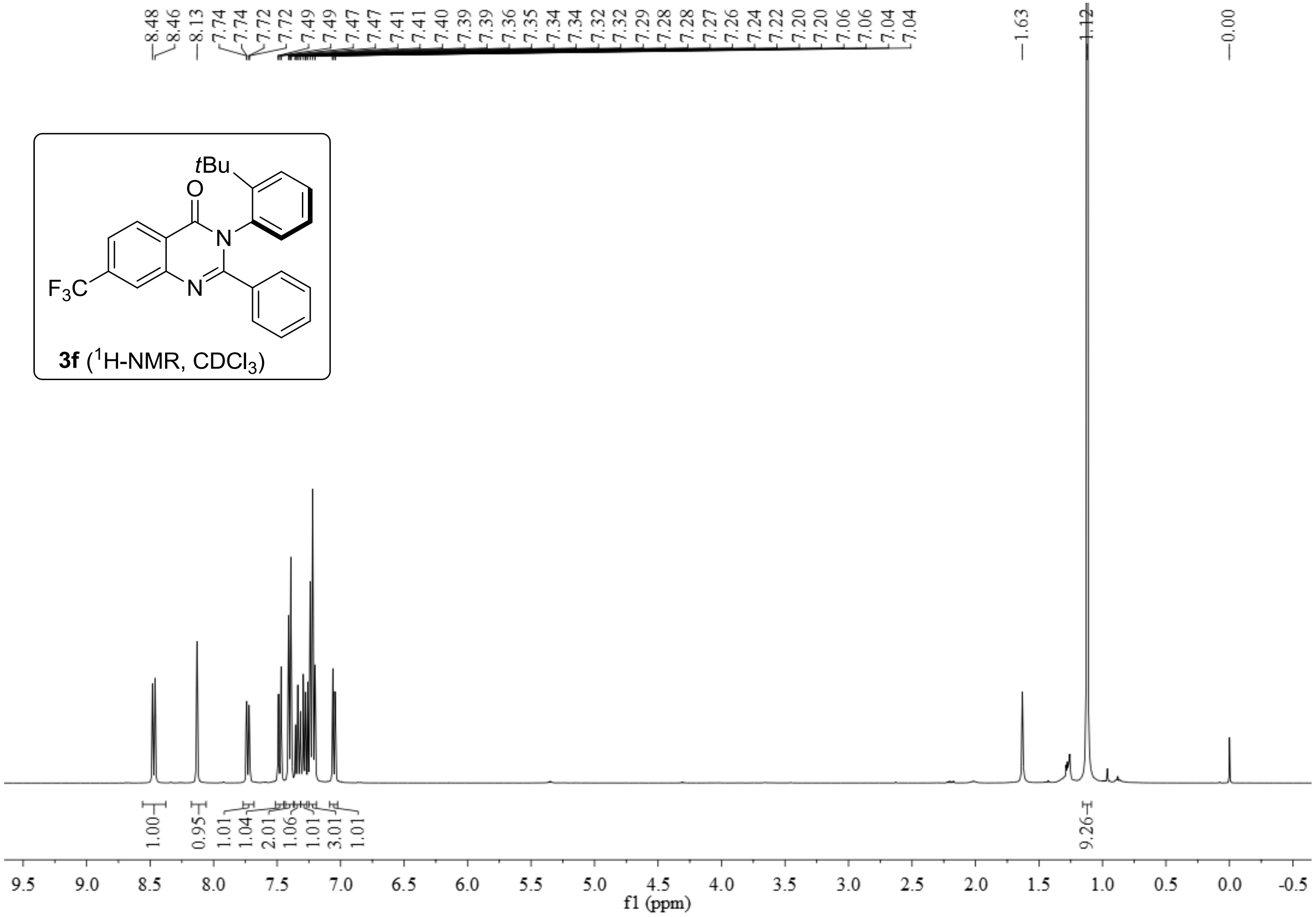


**Supplementary Figure 74.** $^1$H NMR of **3f**

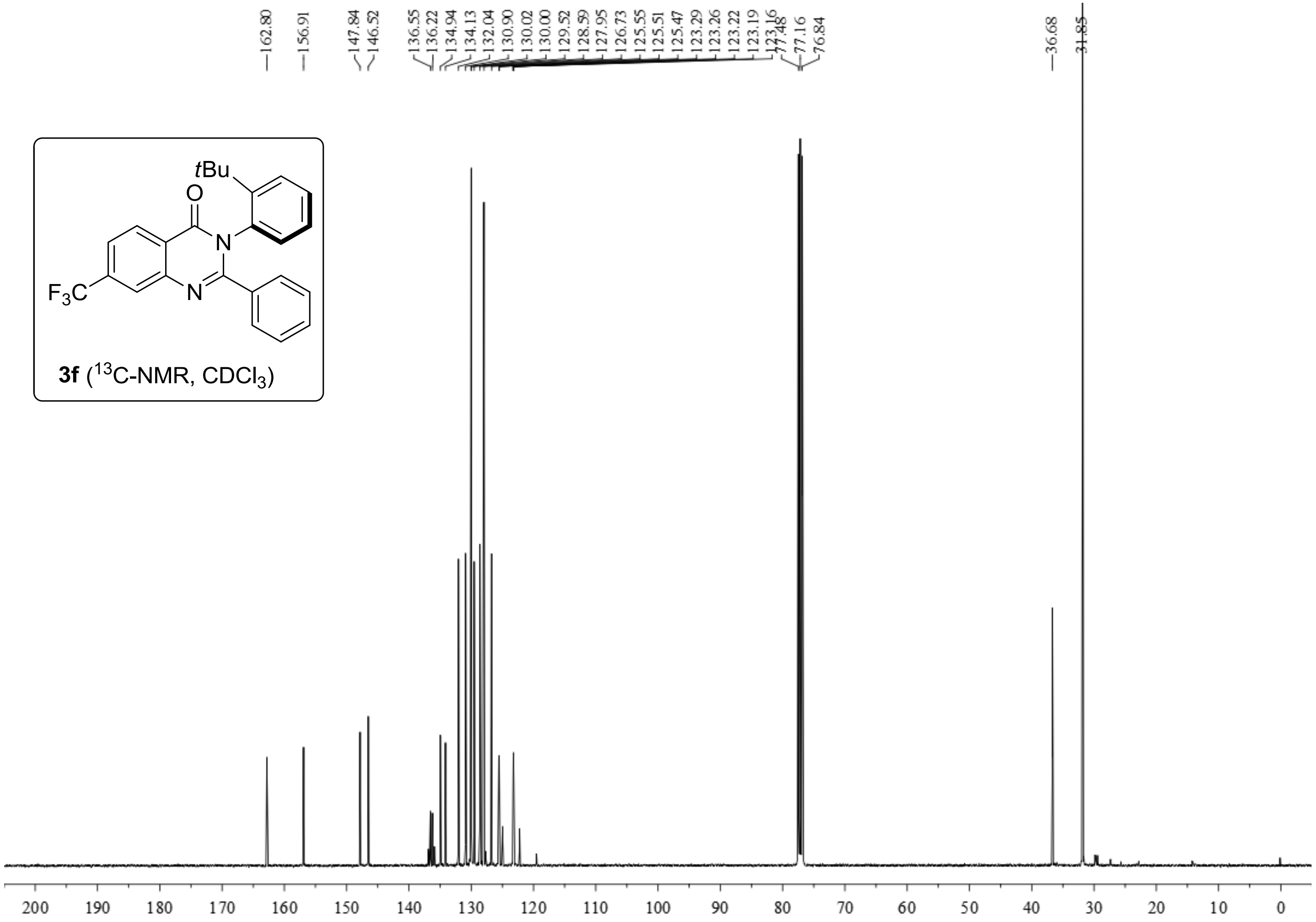


**Supplementary Figure 75.** $^{13}$C NMR of **3f**

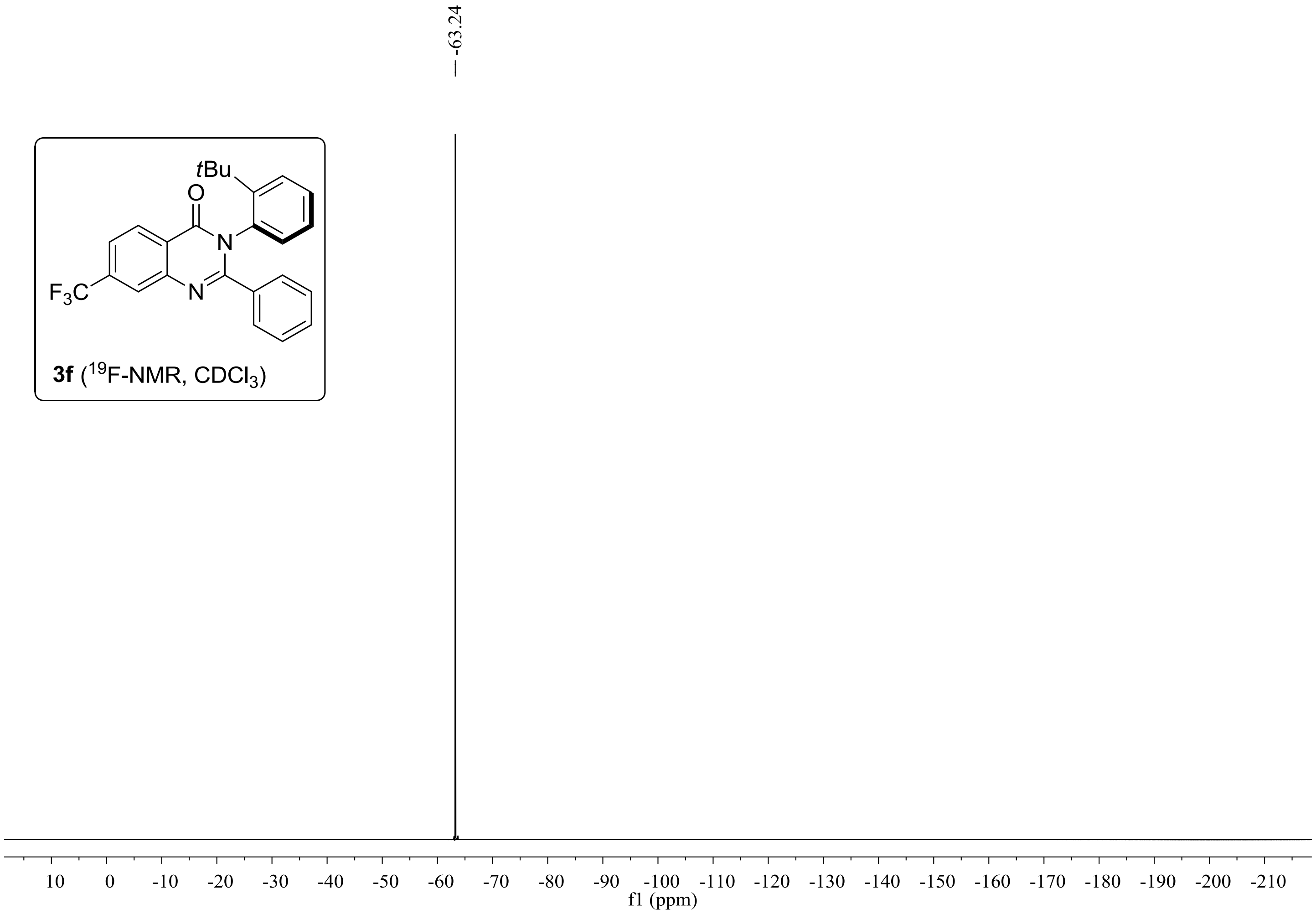


**Supplementary Figure 76.** ¹⁹F NMR of **3f**

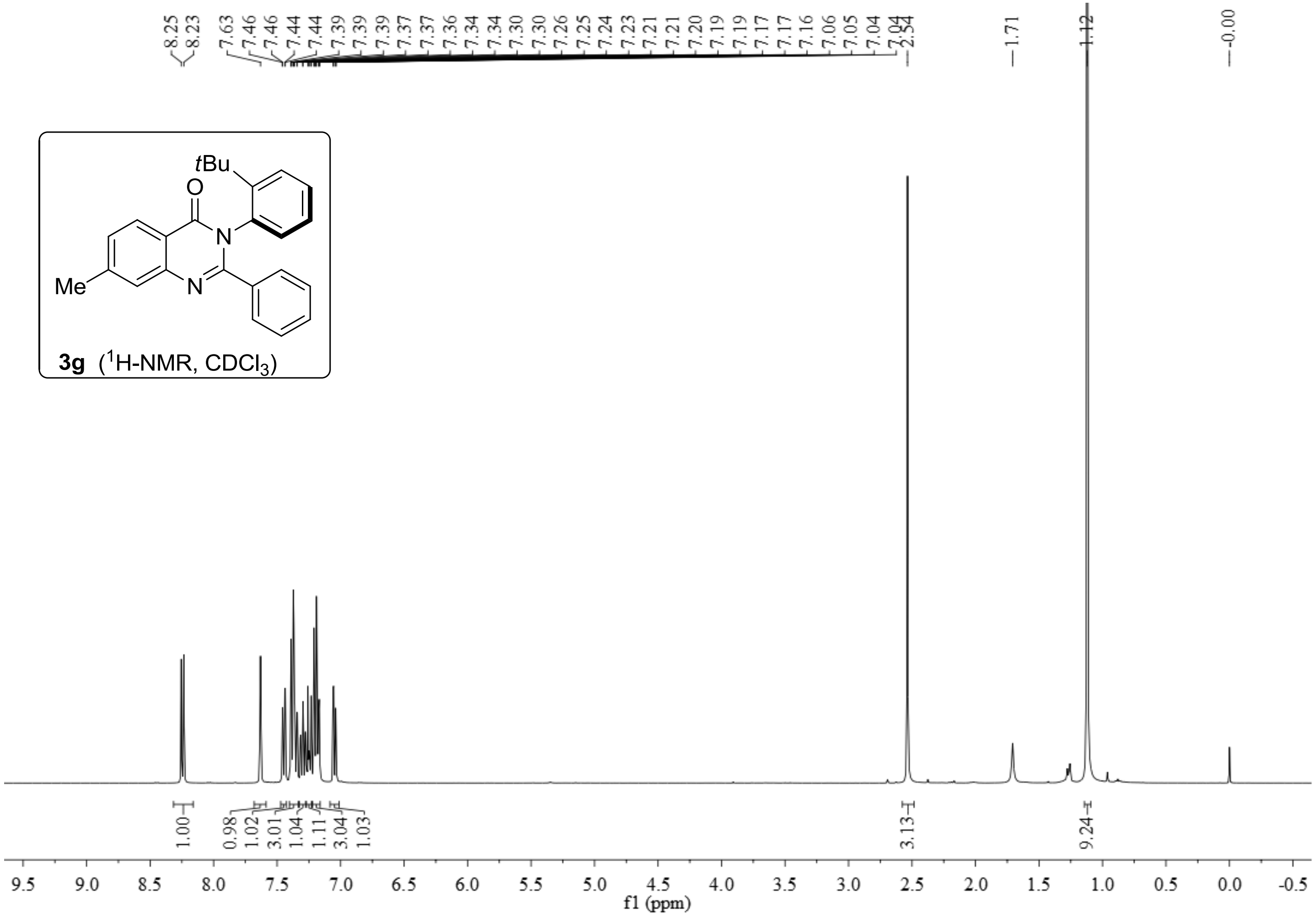


**Supplementary Figure 77.** $^{1}$H NMR of **3g**

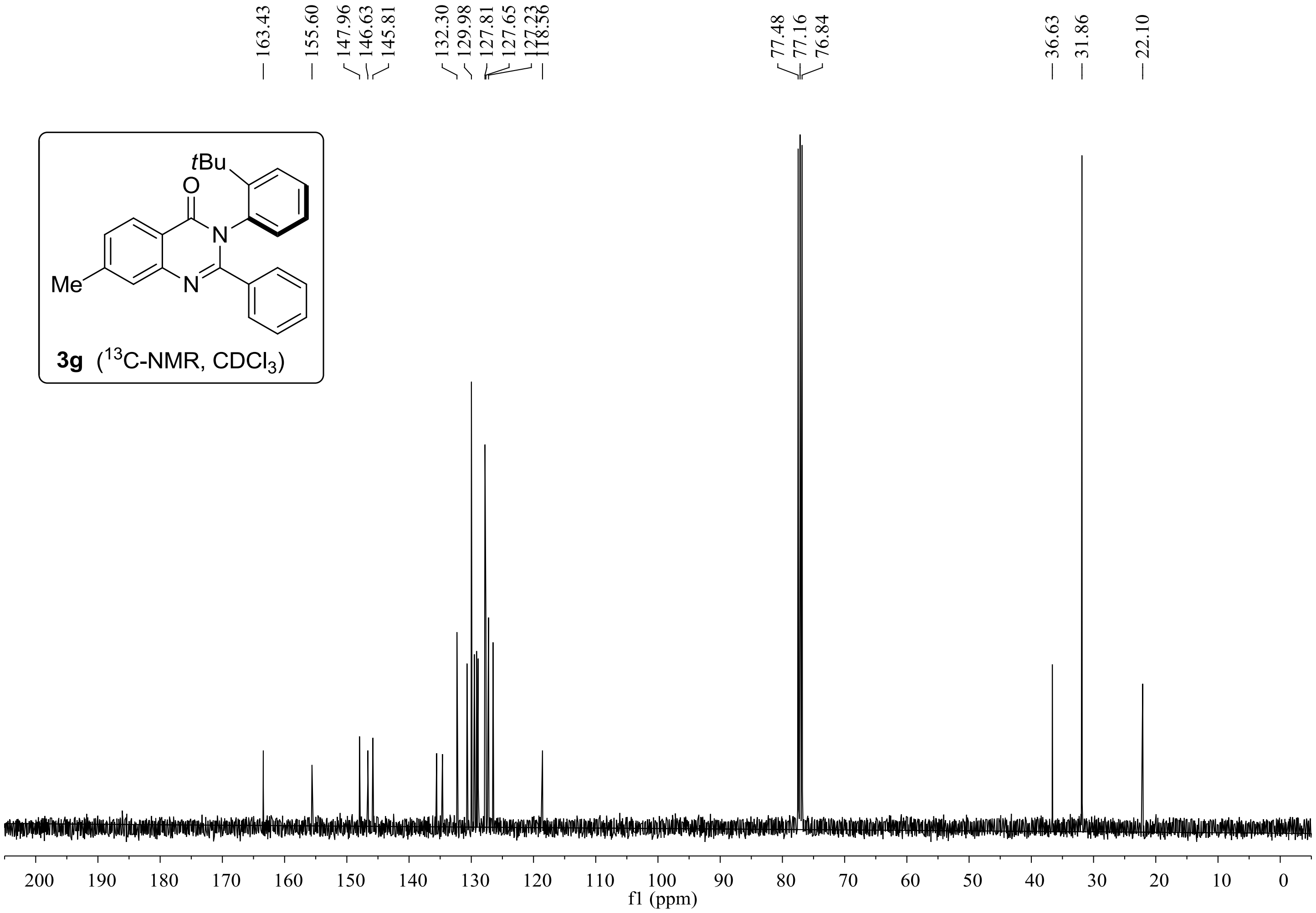


**Supplementary Figure 78.** $^{13}$C NMR of **3g**

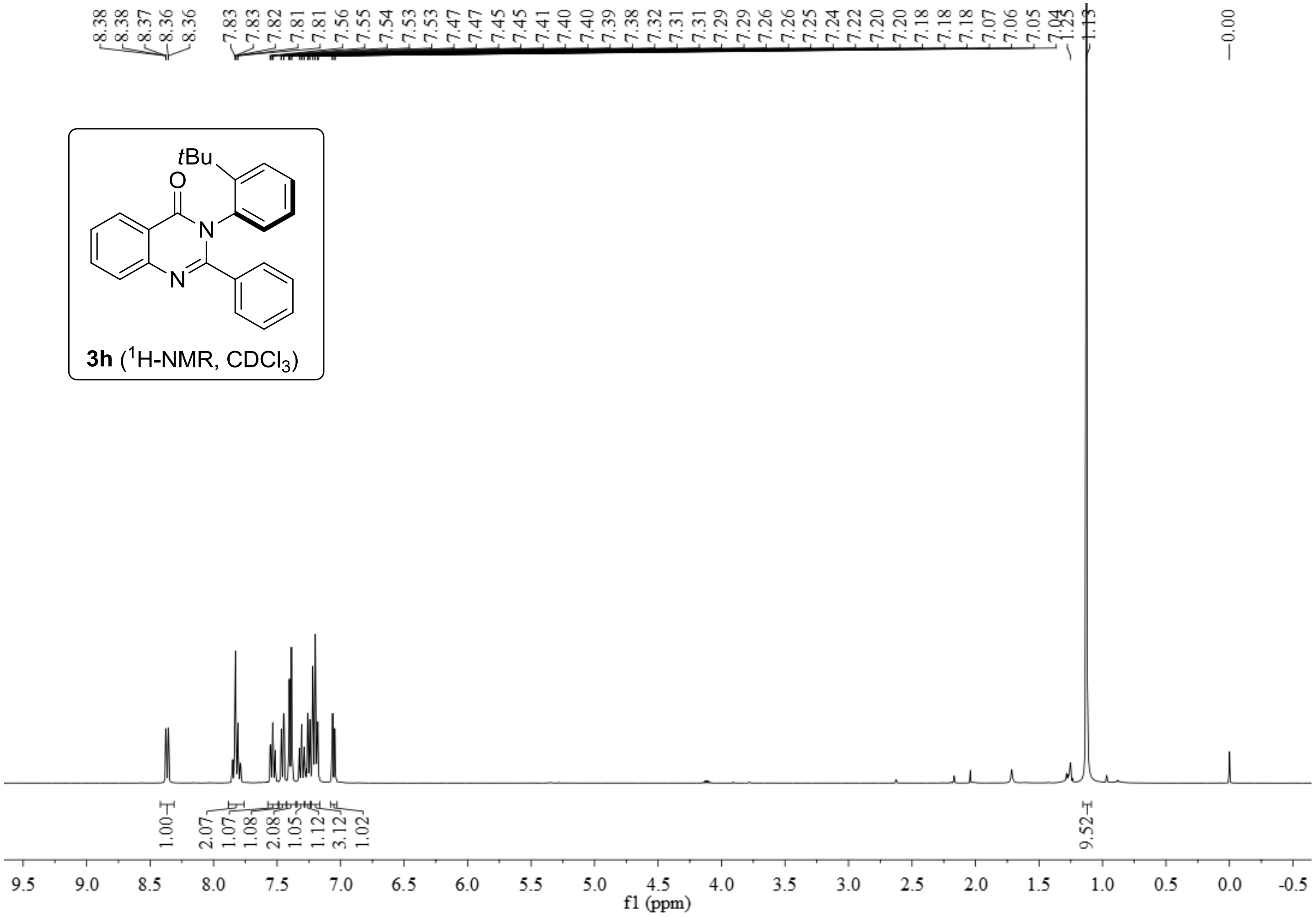


**Supplementary Figure 79.** $^{1}$H NMR of **3h**

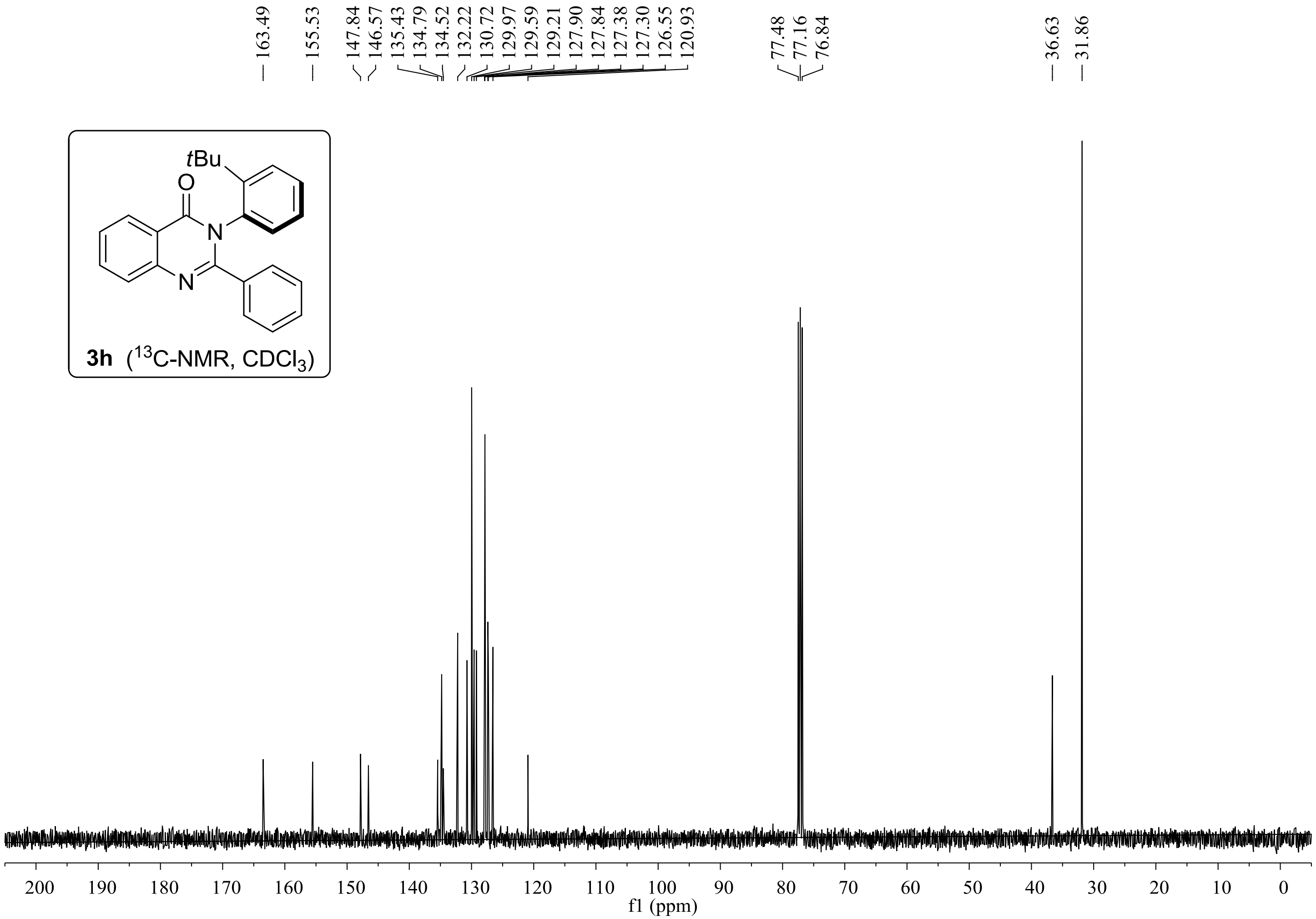


**Supplementary Figure 80.** $^{13}$C NMR of **3h**

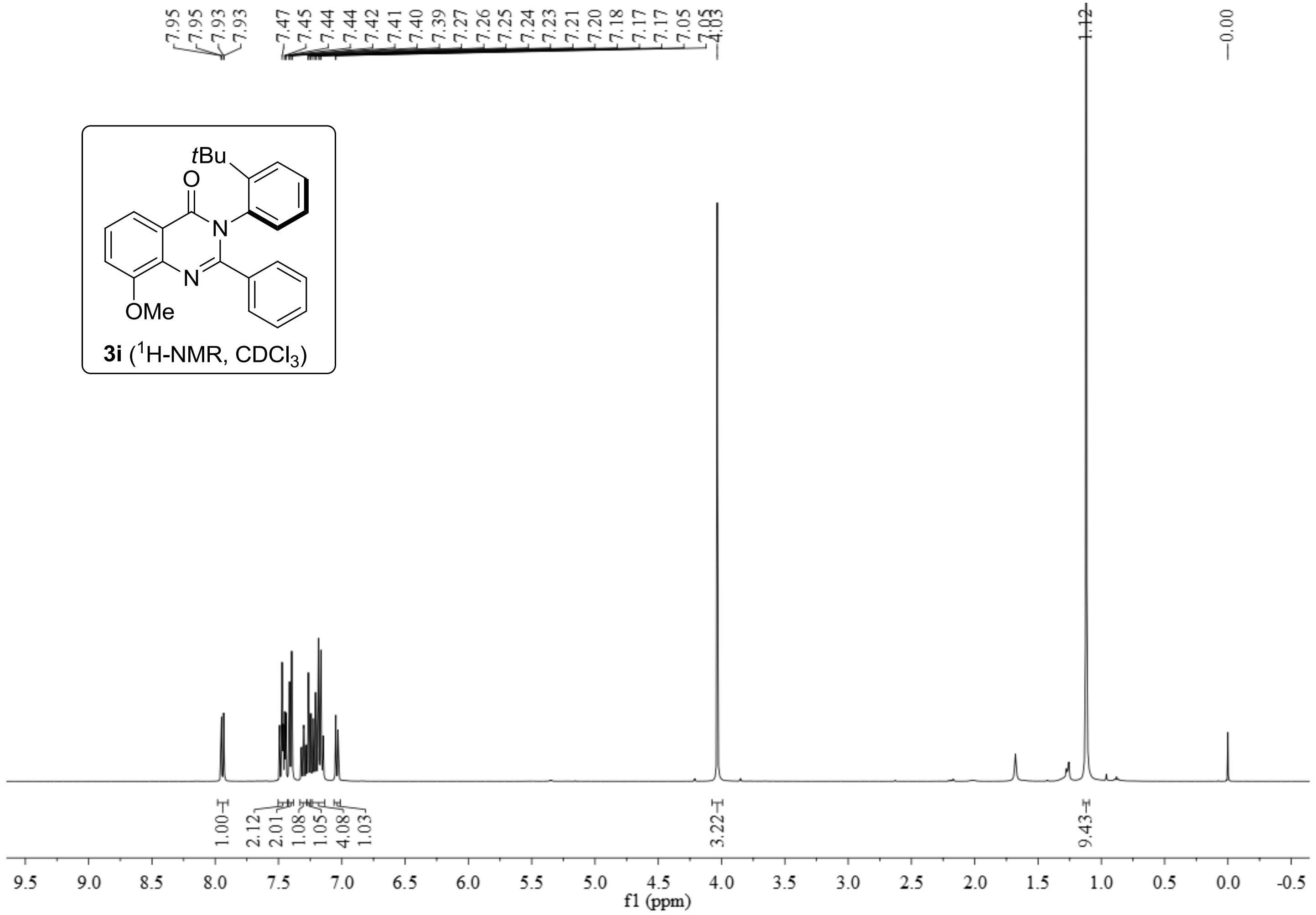


**Supplementary Figure 81.** $^{1}$H NMR of **3i**

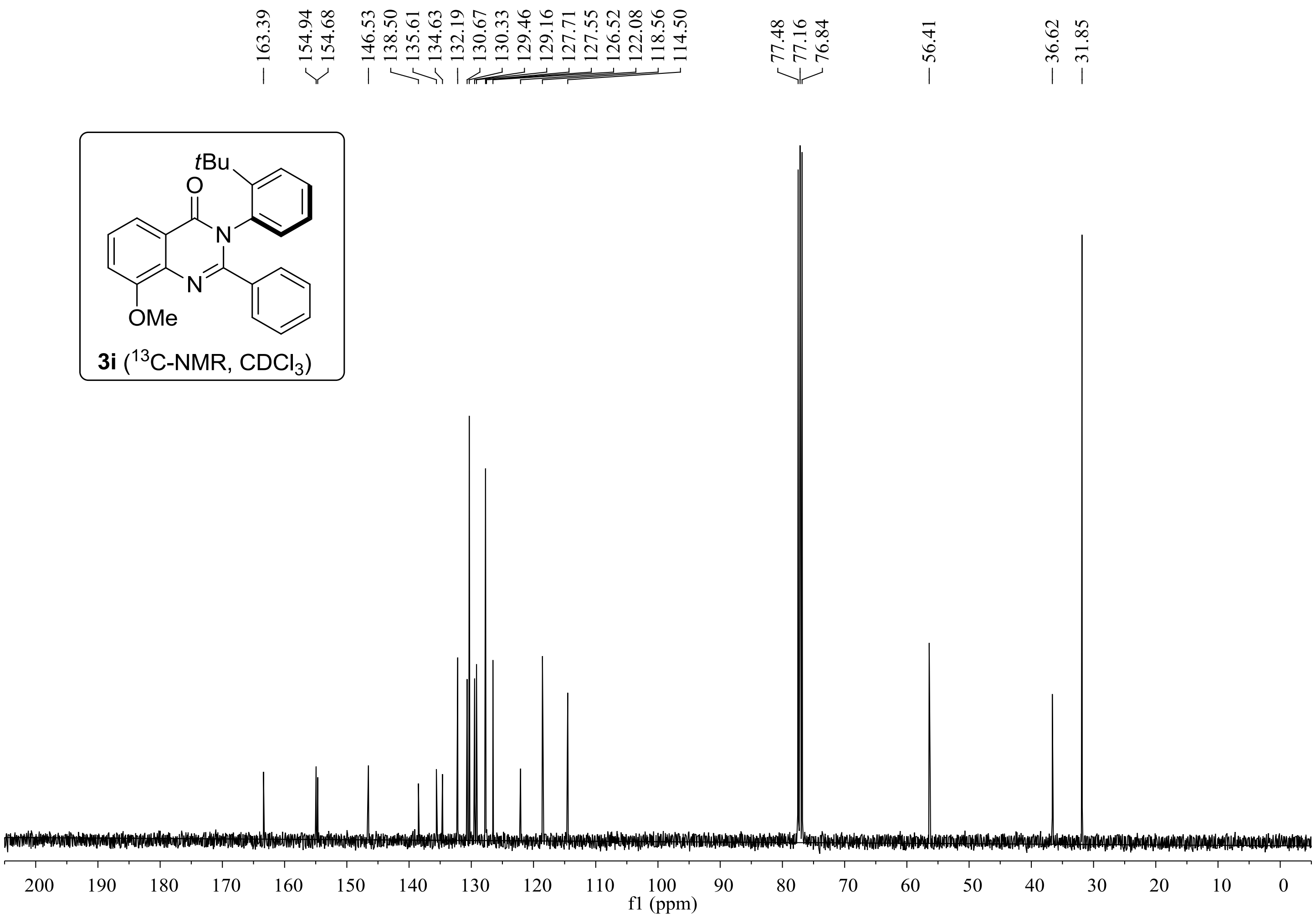


**Supplementary Figure 82.** $^{13}$C NMR of **3i**

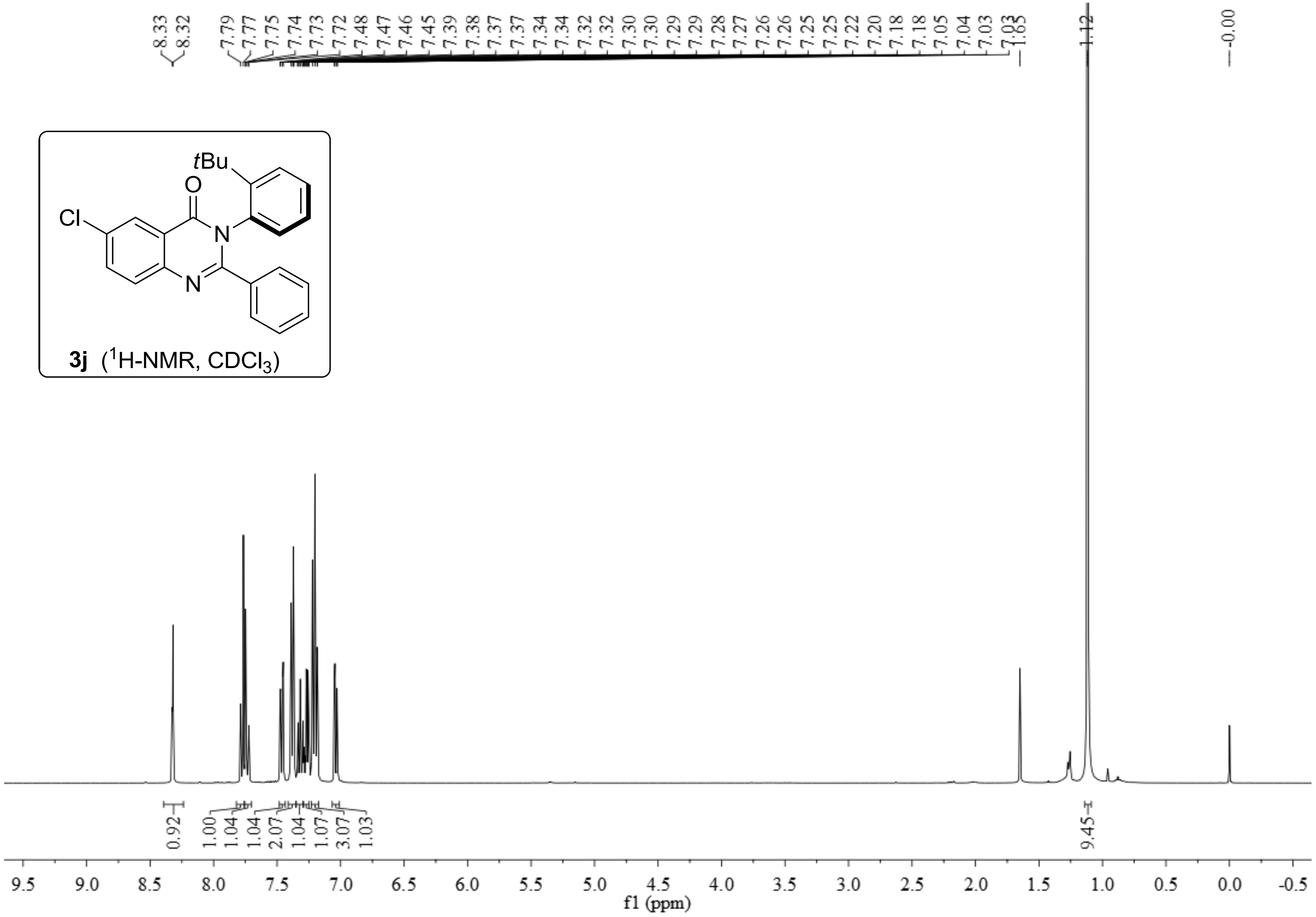


**Supplementary Figure 83.** $^1$H NMR of **3j**

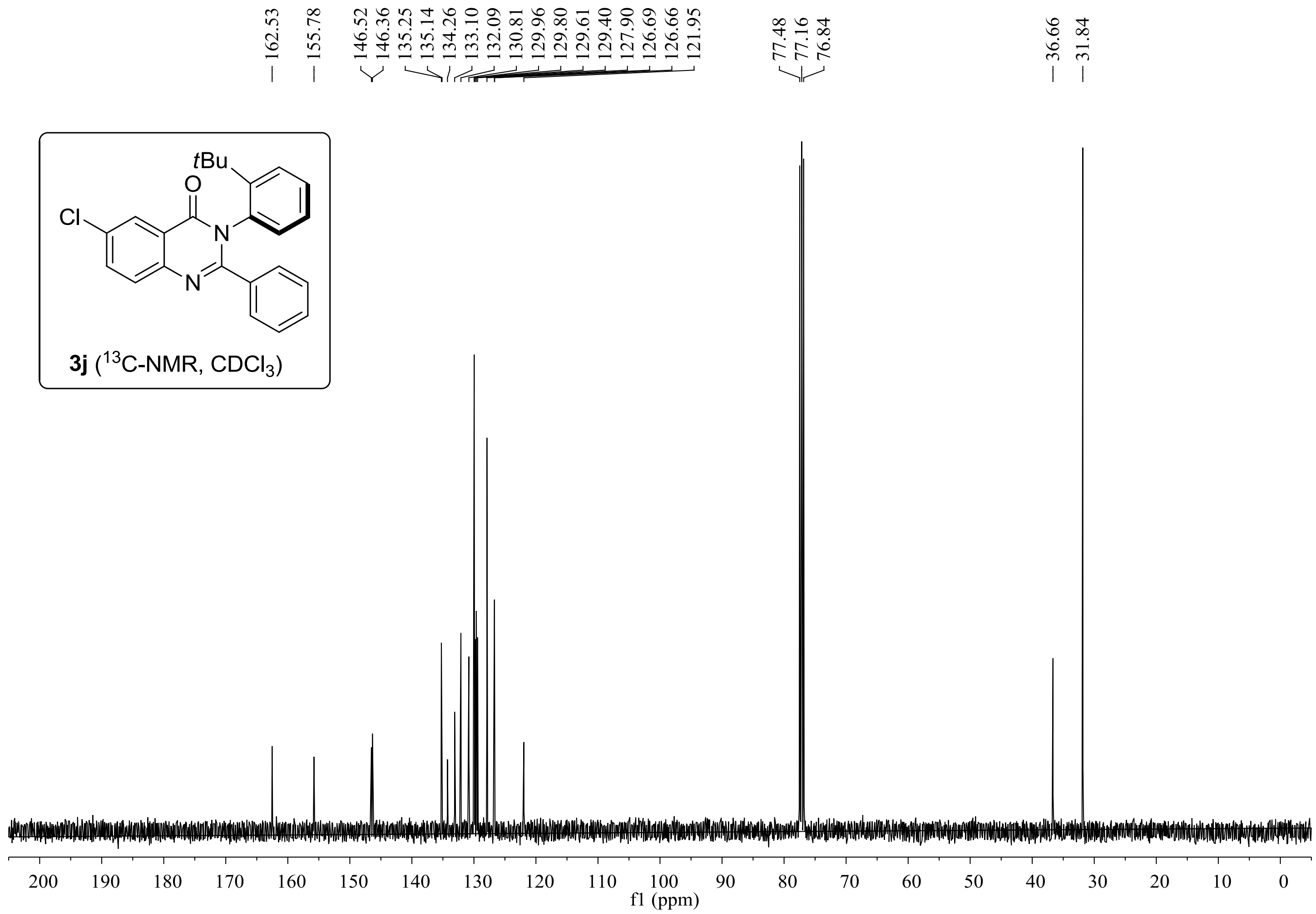


**Supplementary Figure 84.** $^{13}$C NMR of **3j**

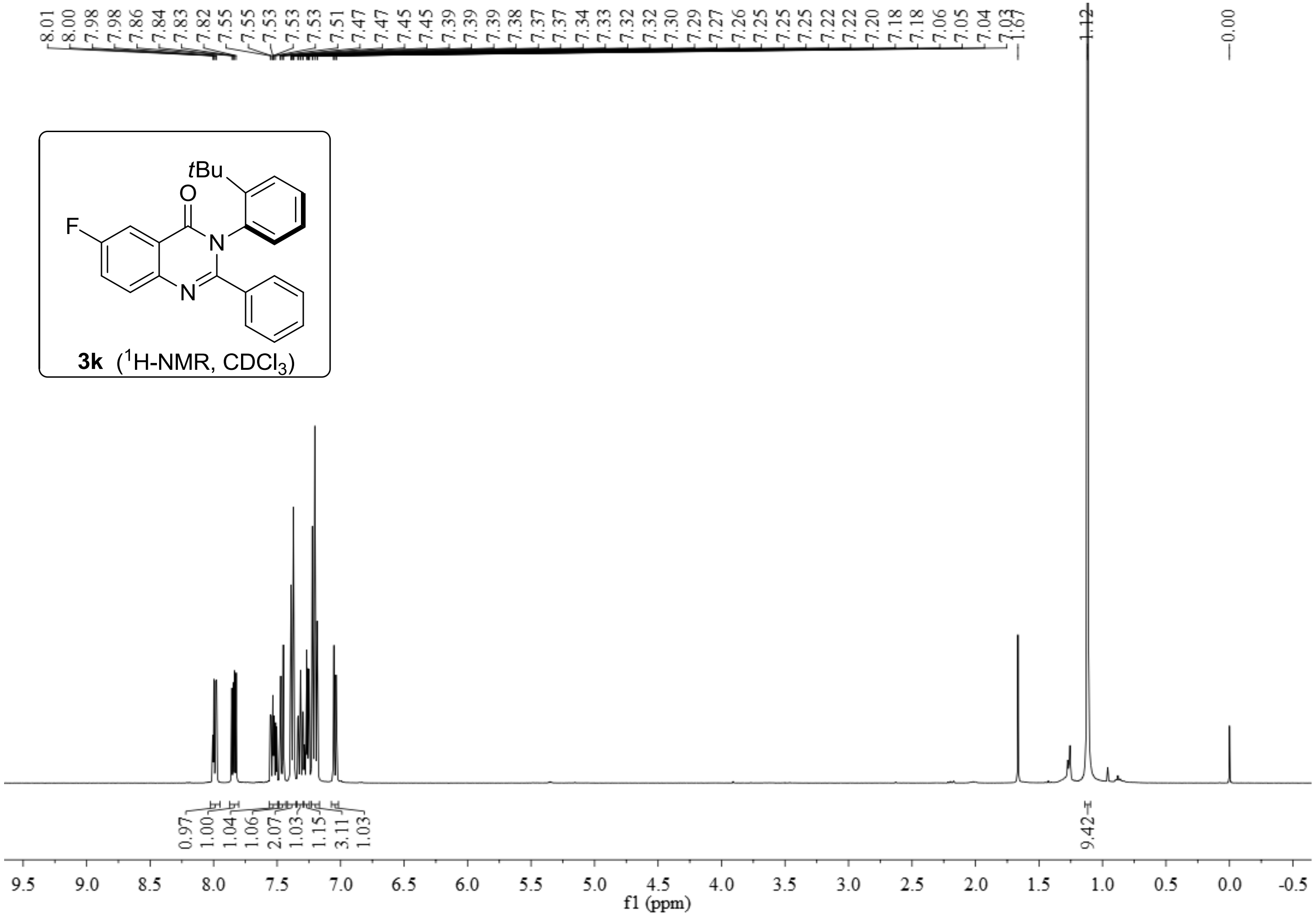


**Supplementary Figure 85.** $^{1}$H NMR of **3k**

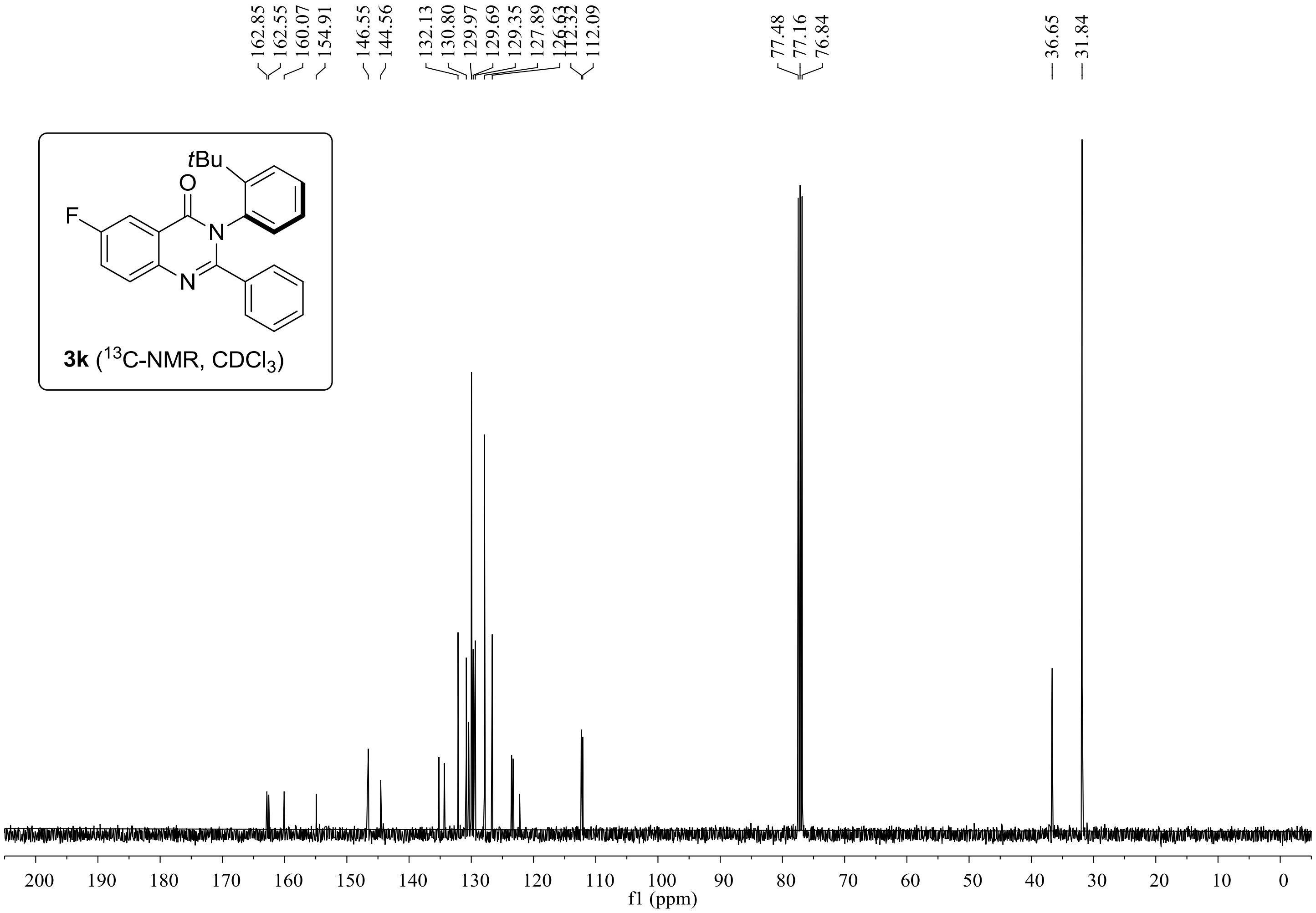


**Supplementary Figure 86.** $^{13}$C NMR of **3k**

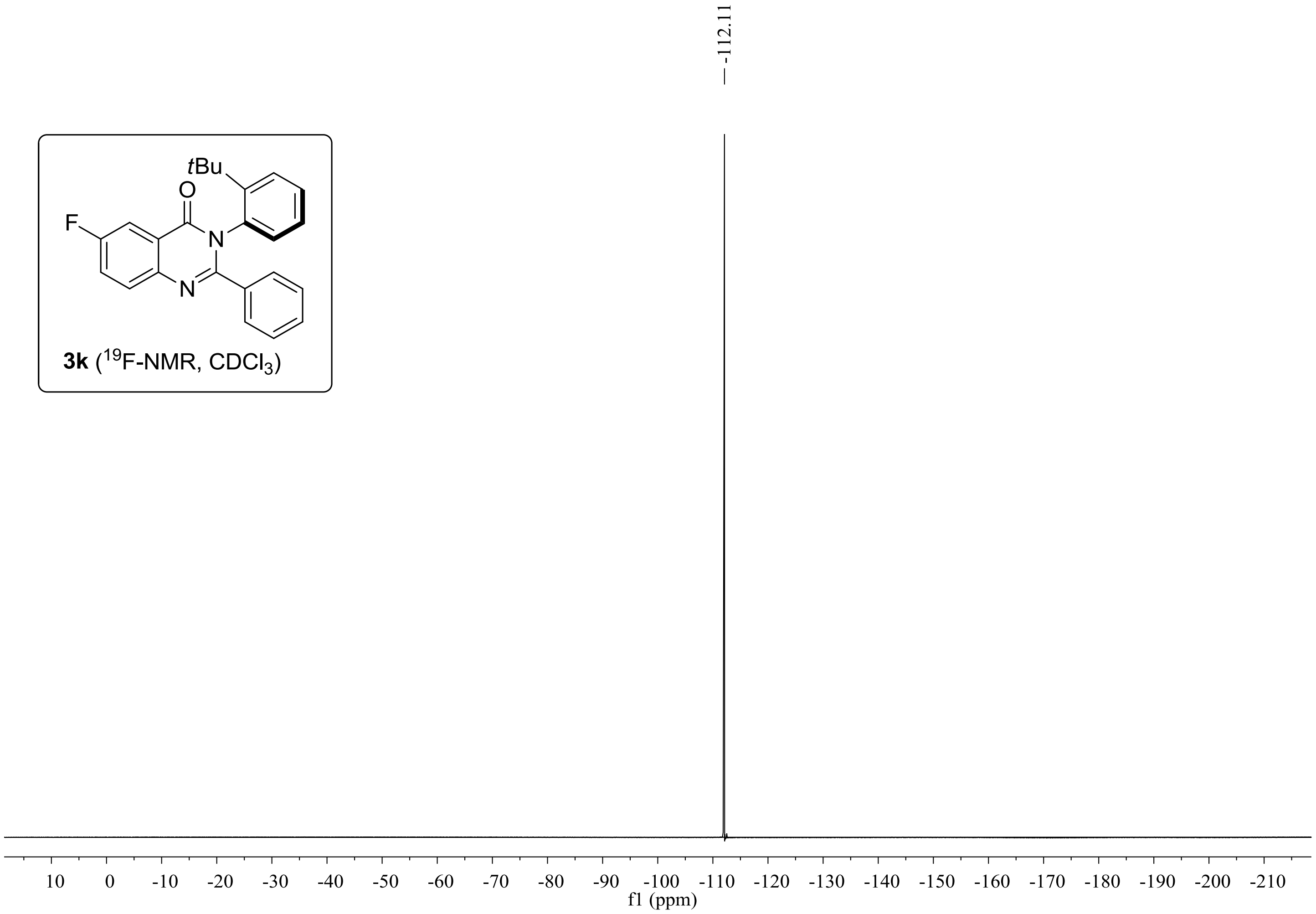


**Supplementary Figure 87.** $^{19}$F NMR of **3k**

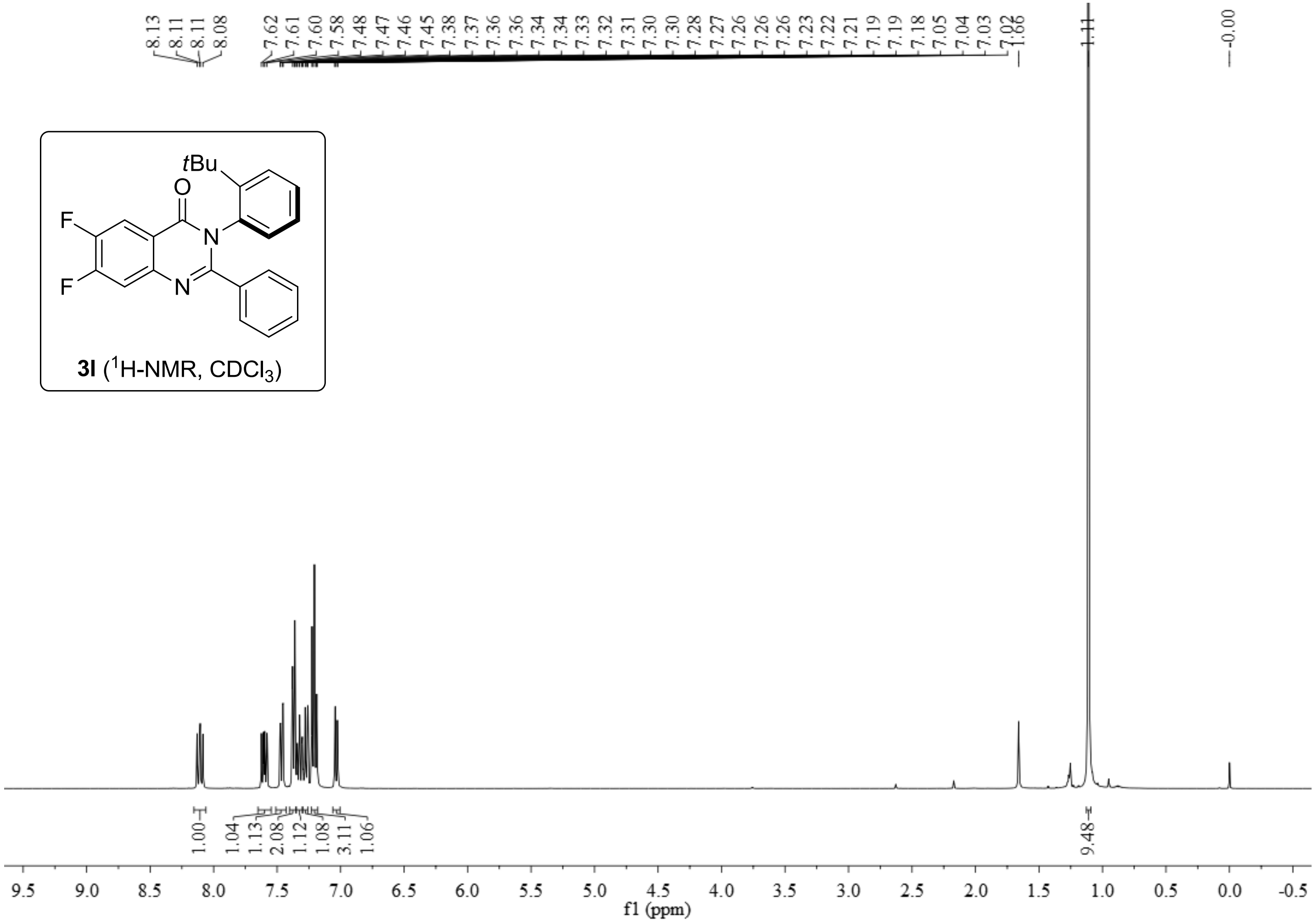


**Supplementary Figure 88.** $^{1}$H NMR of **3l**

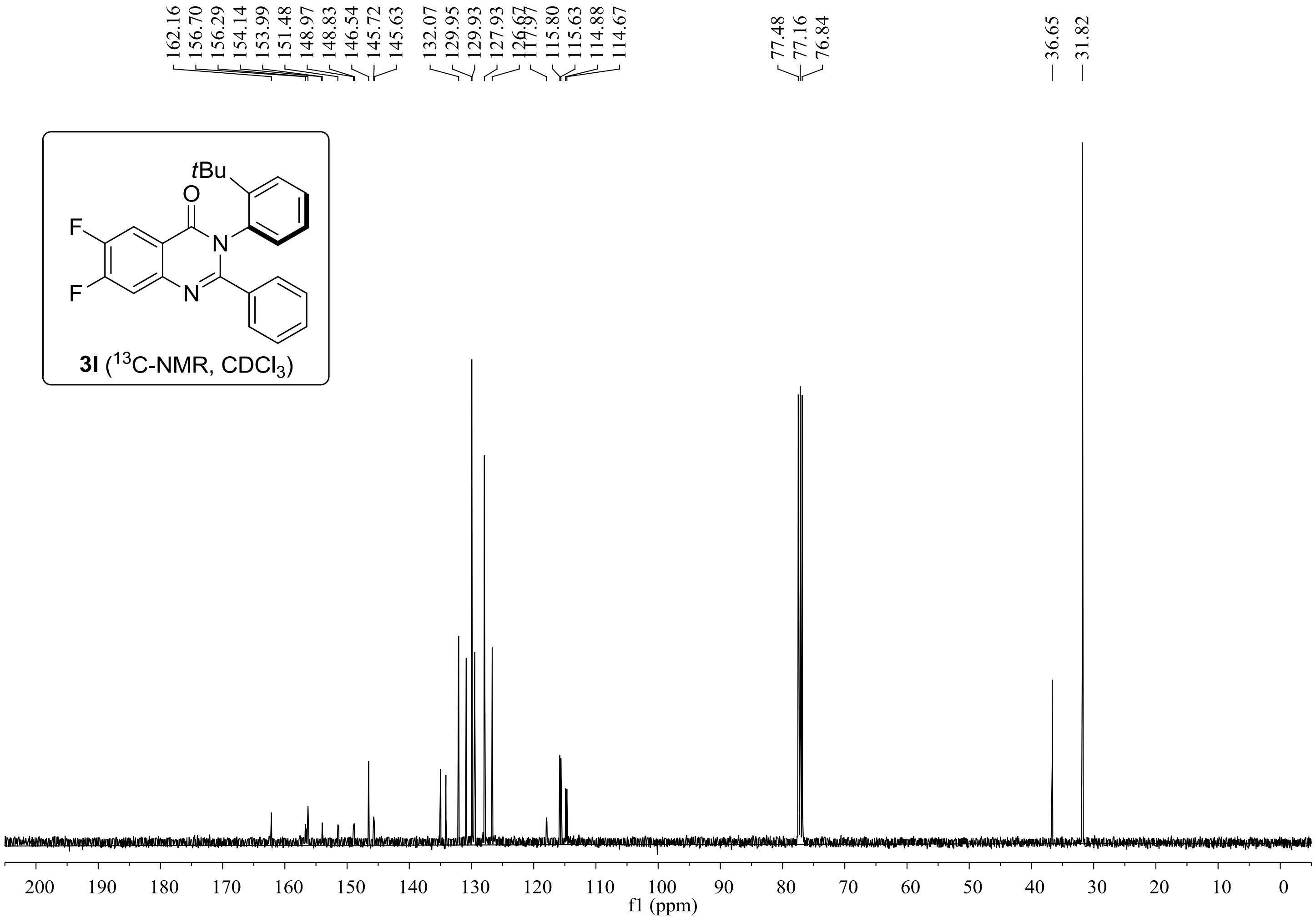


**Supplementary Figure 89.** $^{13}$C NMR of **3l**

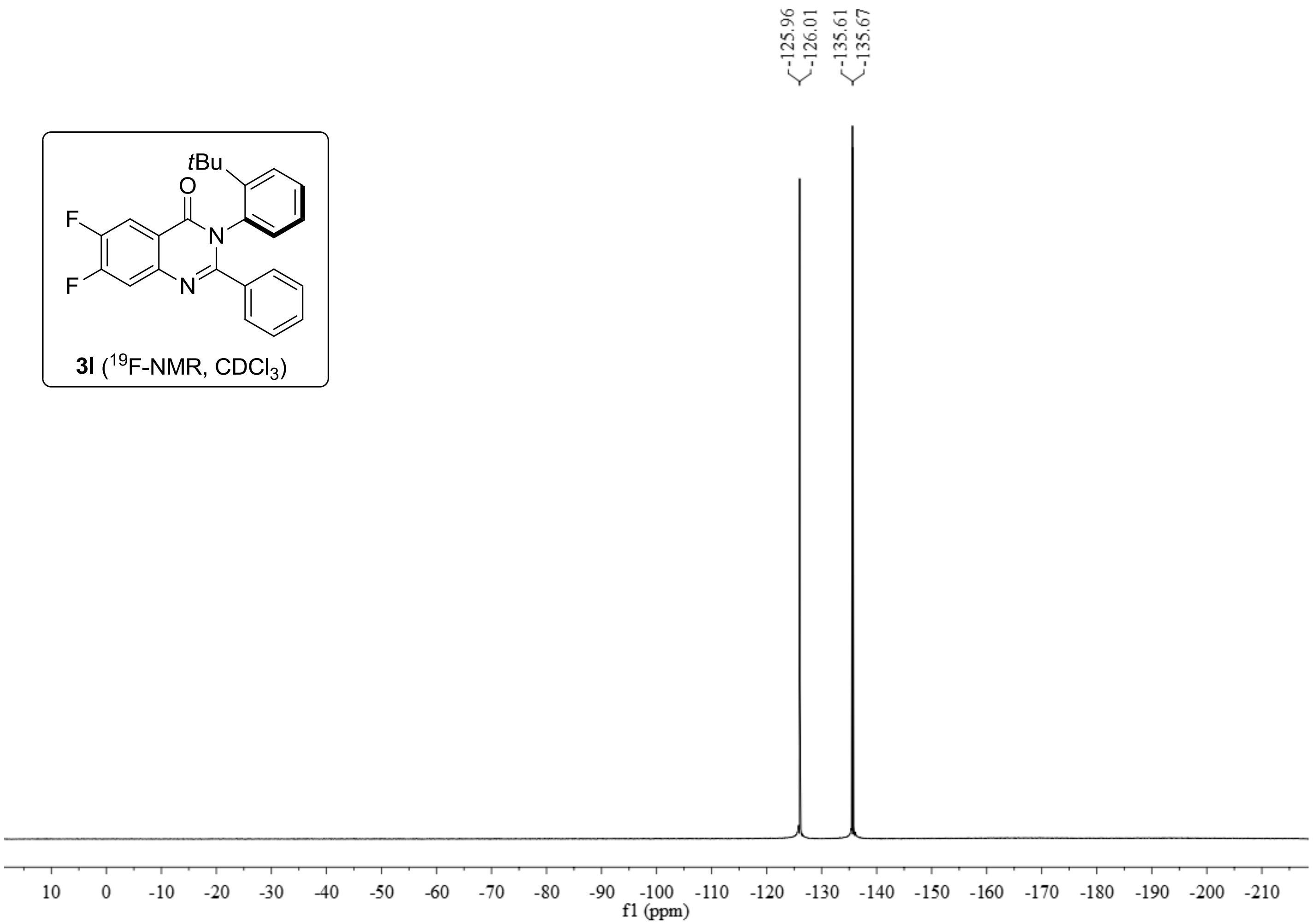


**Supplementary Figure 90.** $^{19}$F NMR of **3l**

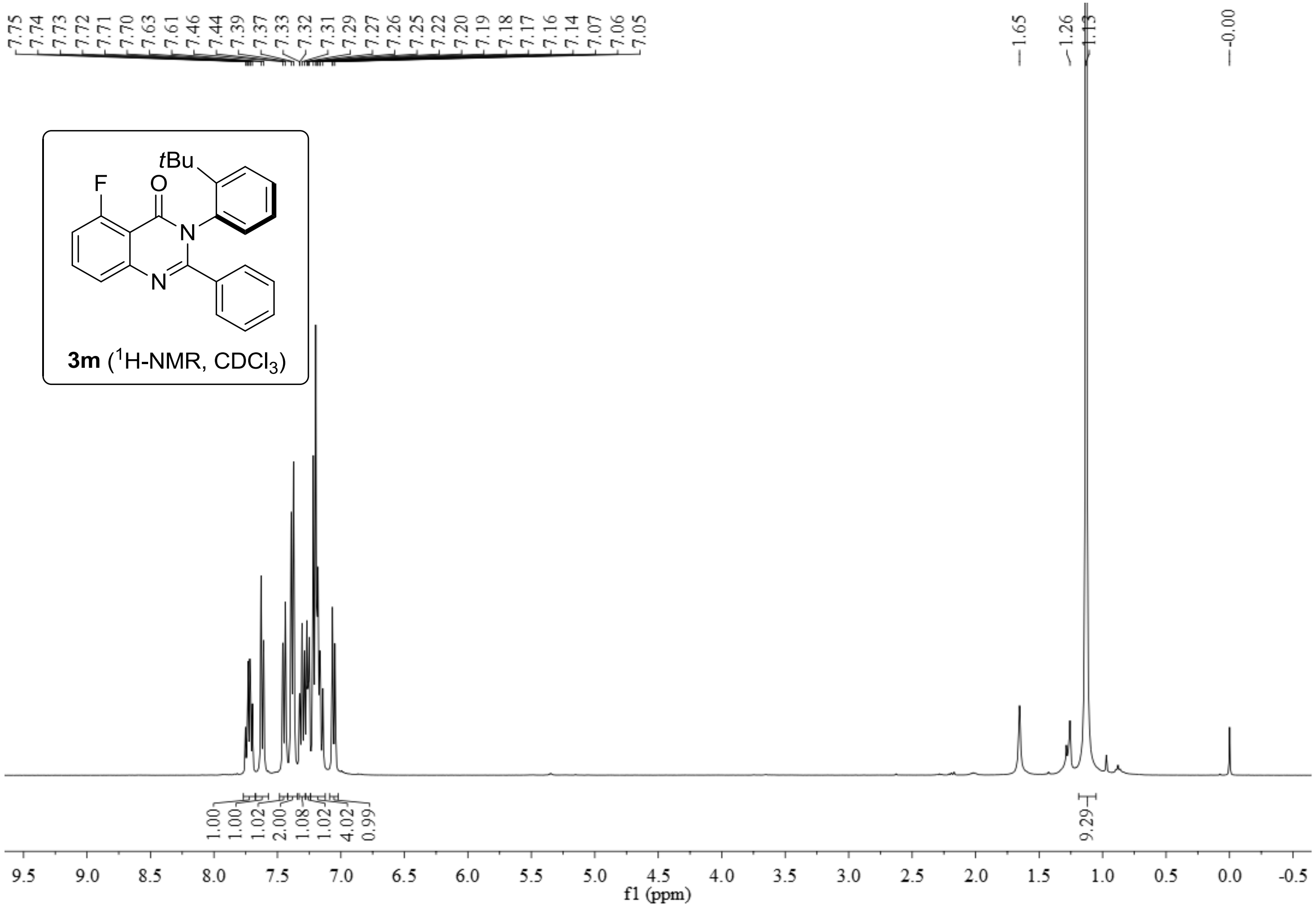


**Supplementary Figure 91.** $^{1}$H NMR of **3m**

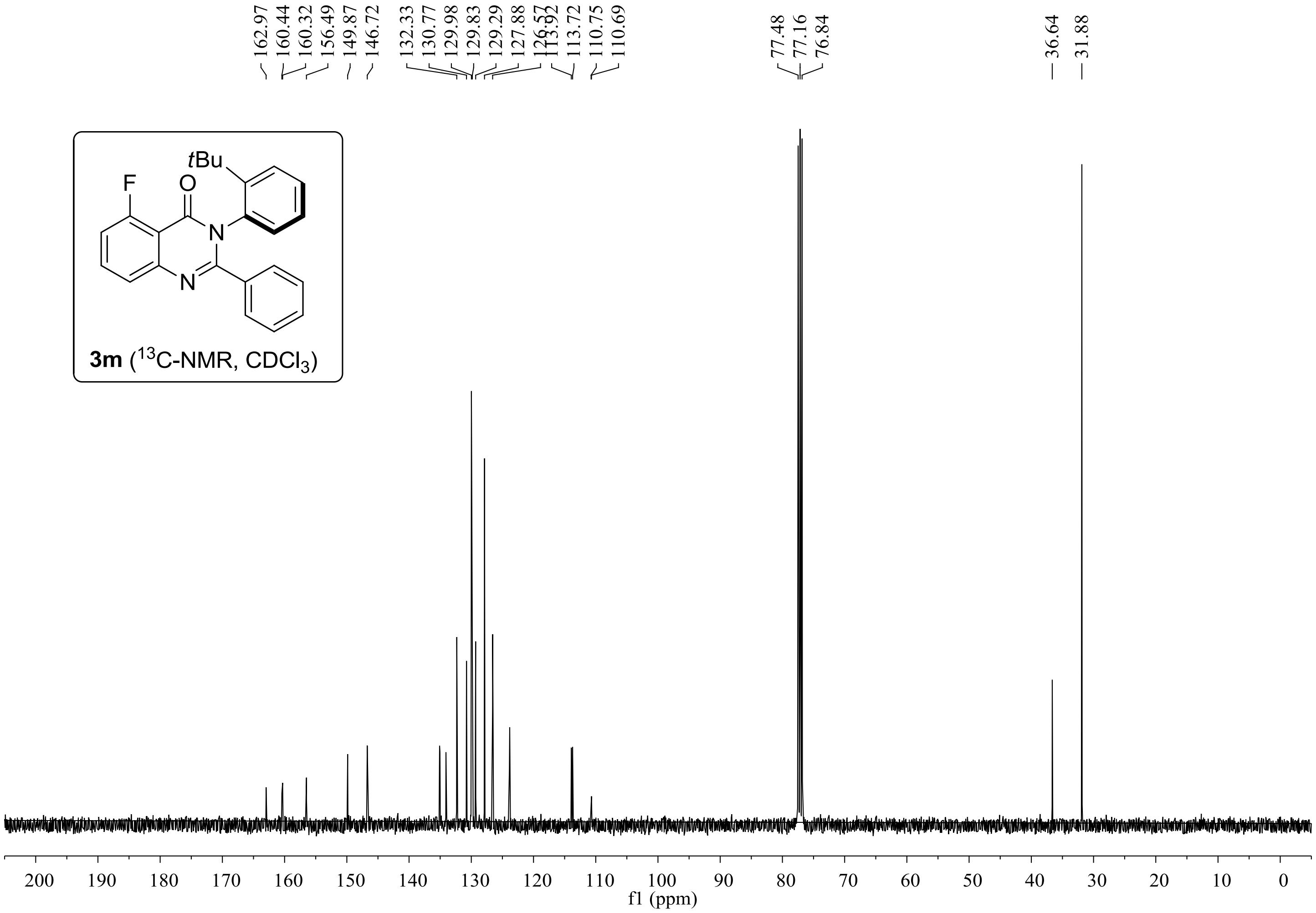


**Supplementary Figure 92.** $^{13}$C NMR of **3m**

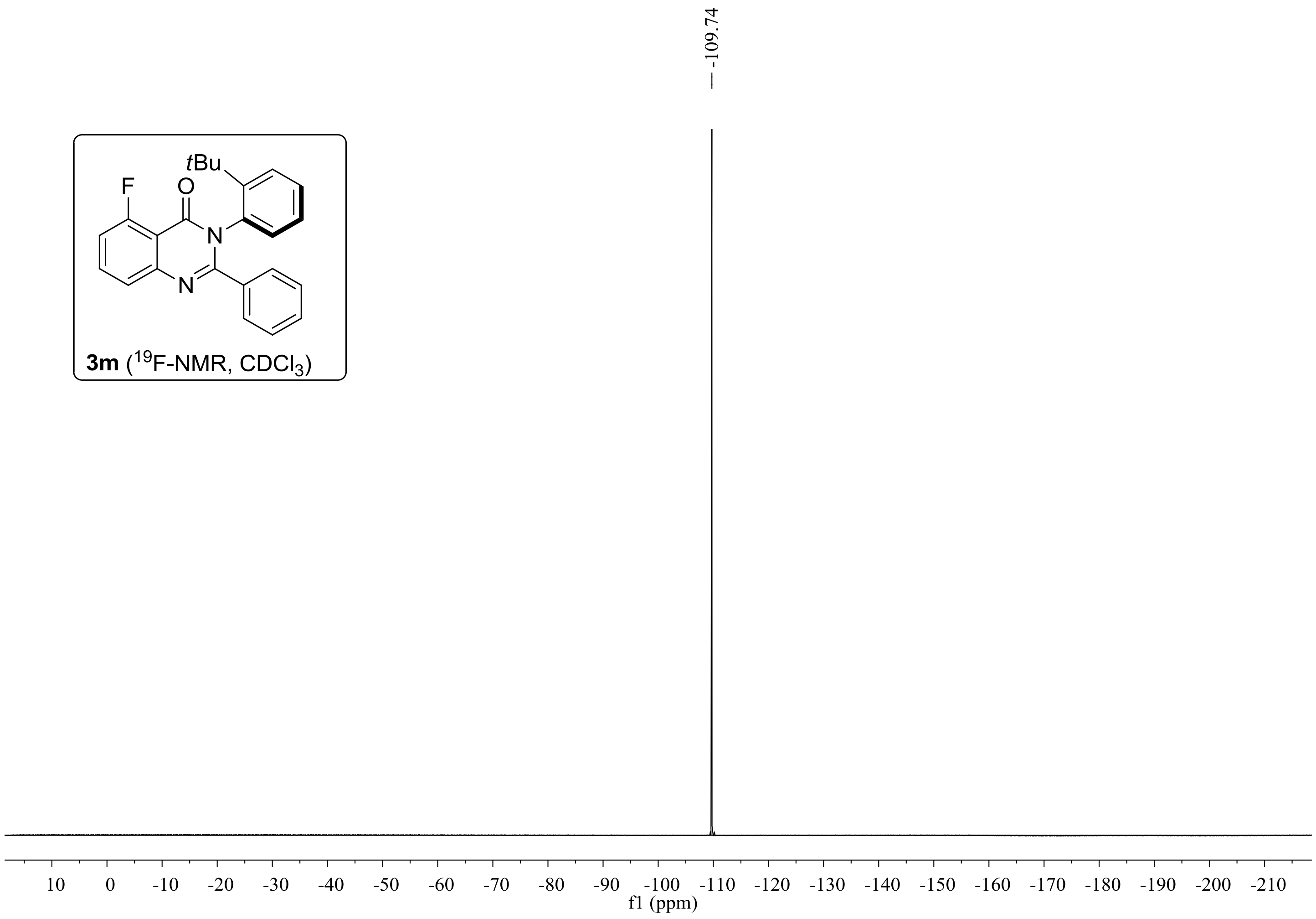


**Supplementary Figure 93.** $^{19}$F NMR of **3m**

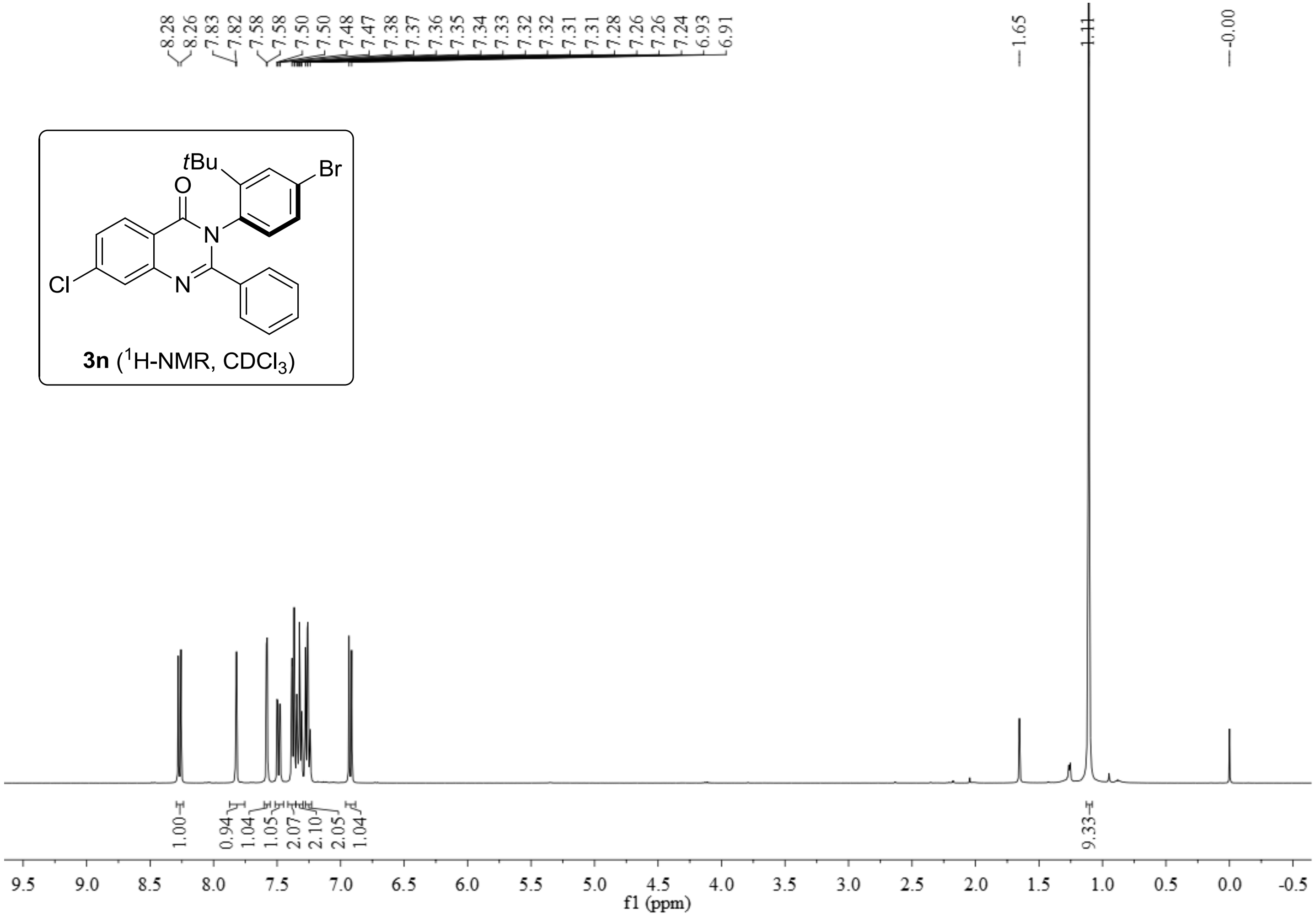


**Supplementary Figure 94.** $^{1}$H NMR of **3n**

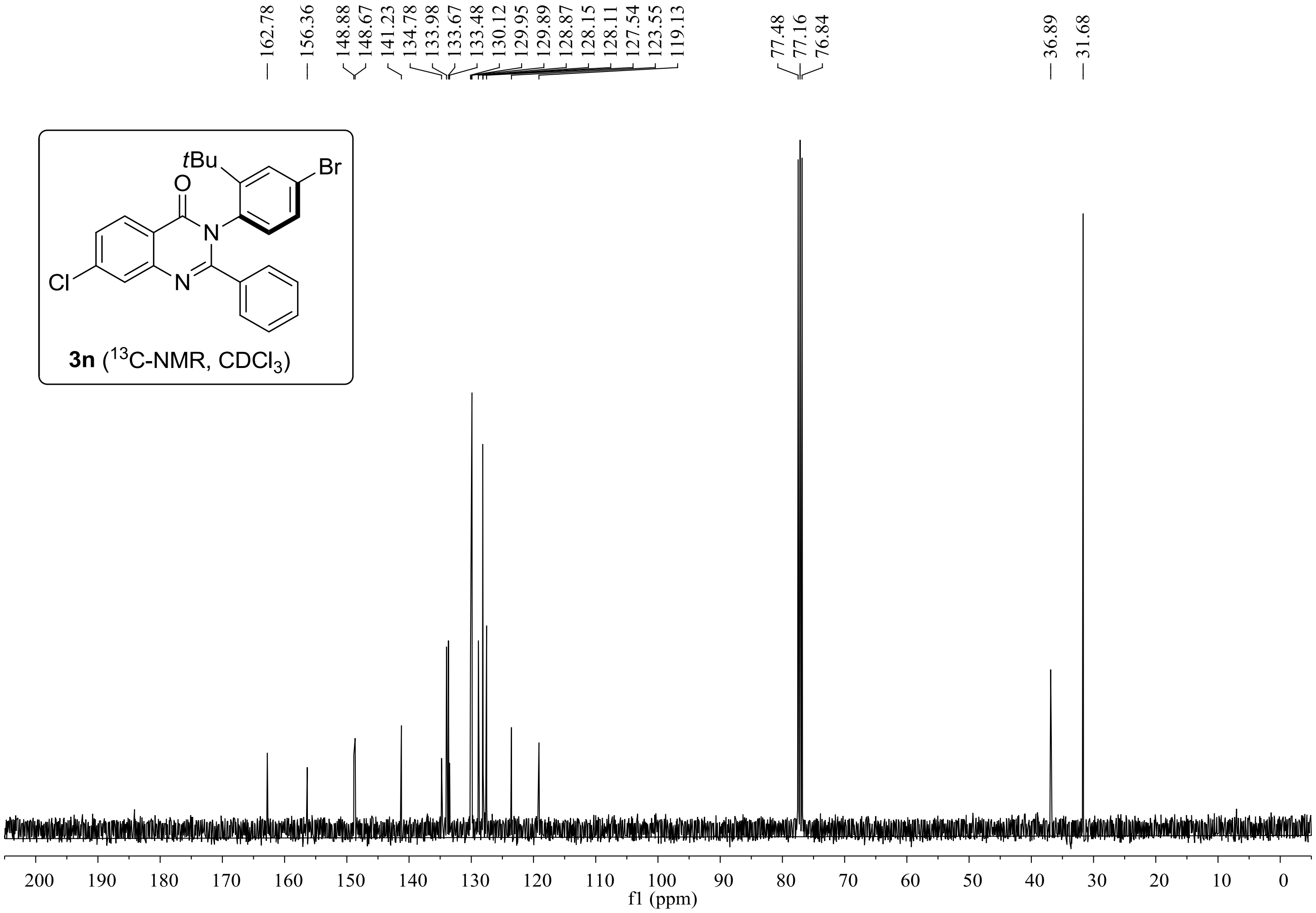


**Supplementary Figure 95.** $^{13}$C NMR of **3n**

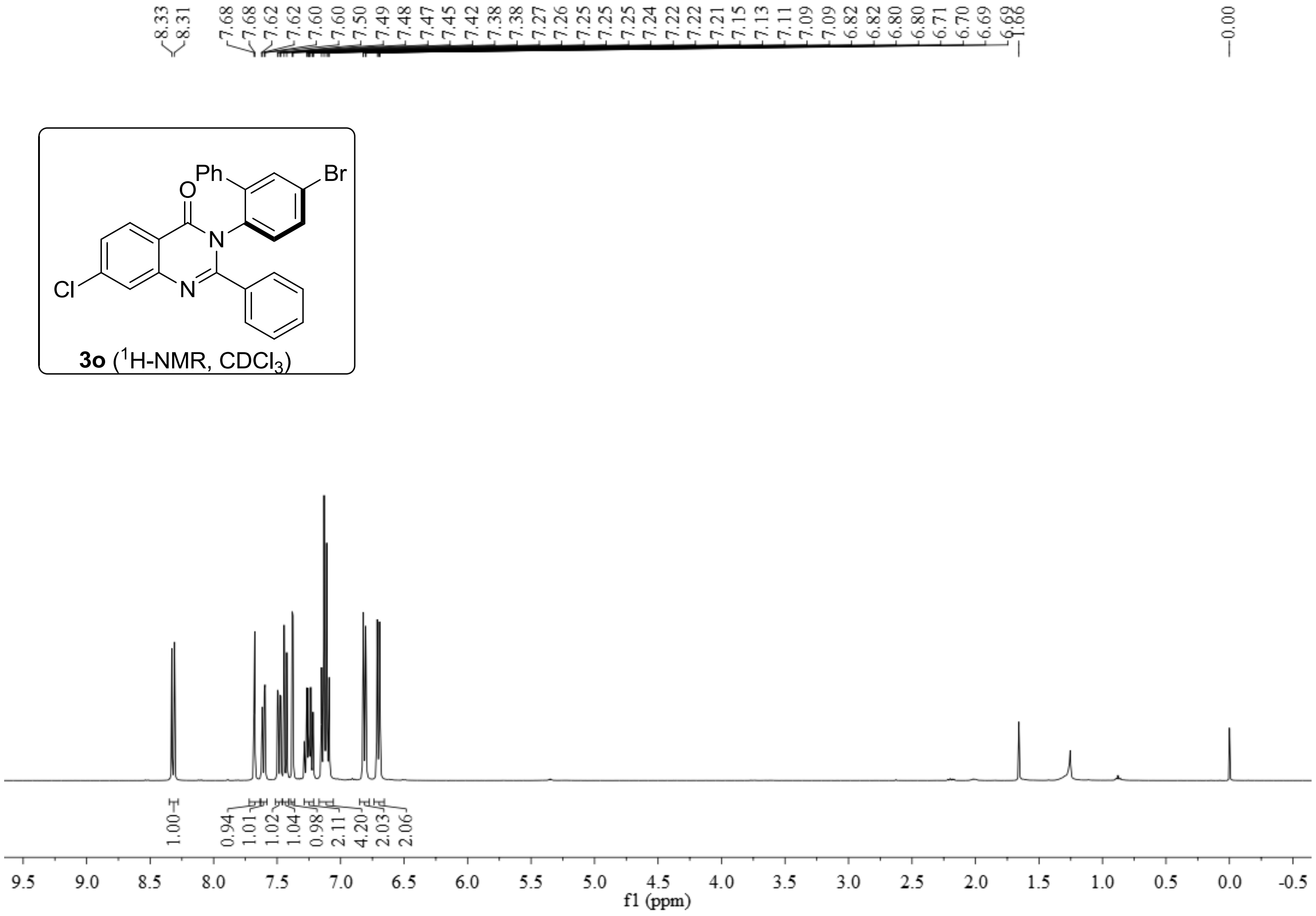


**Supplementary Figure 96.** $^1$H NMR of **3o**

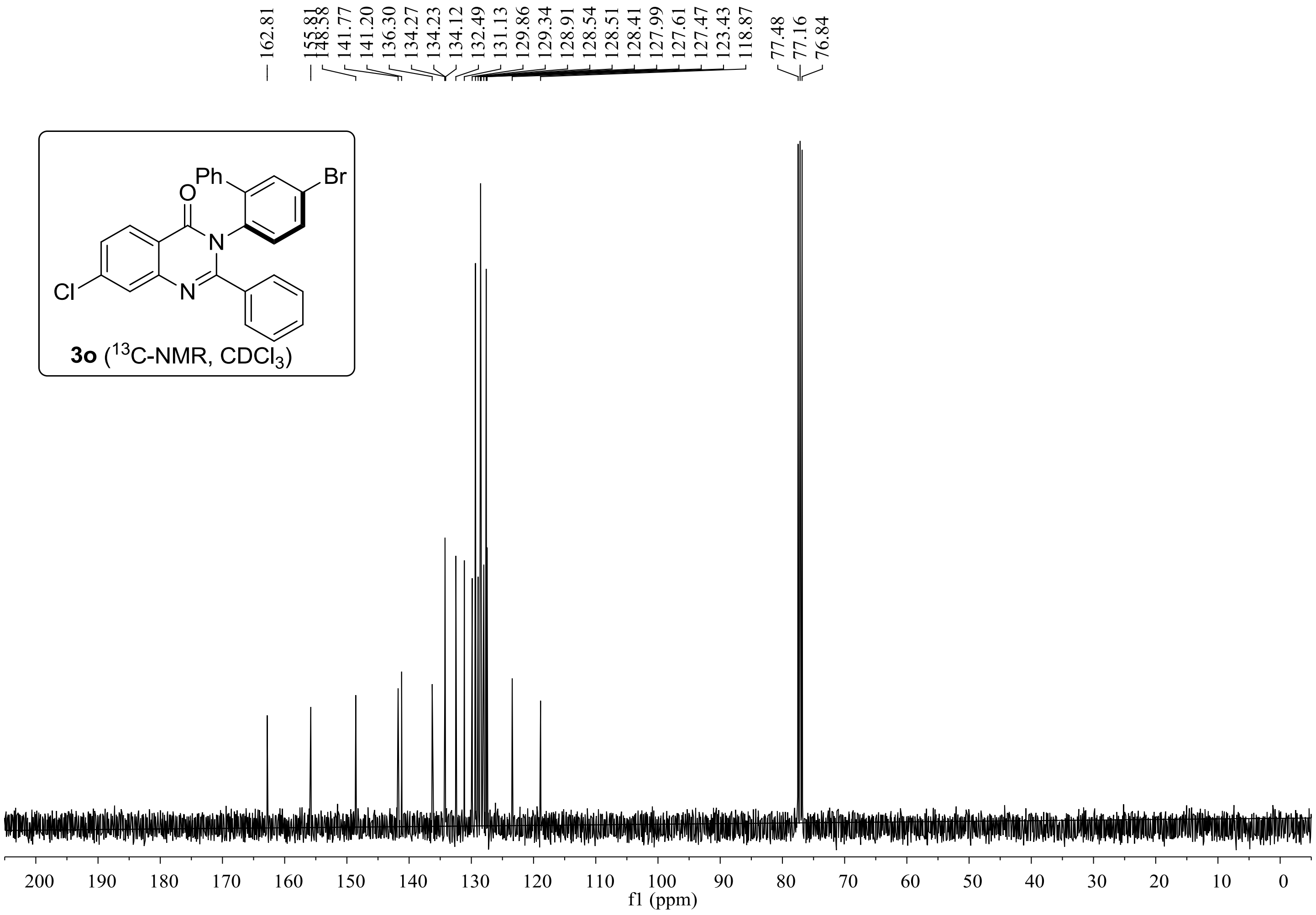


**Supplementary Figure 97.** $^{13}$C NMR of **3o**

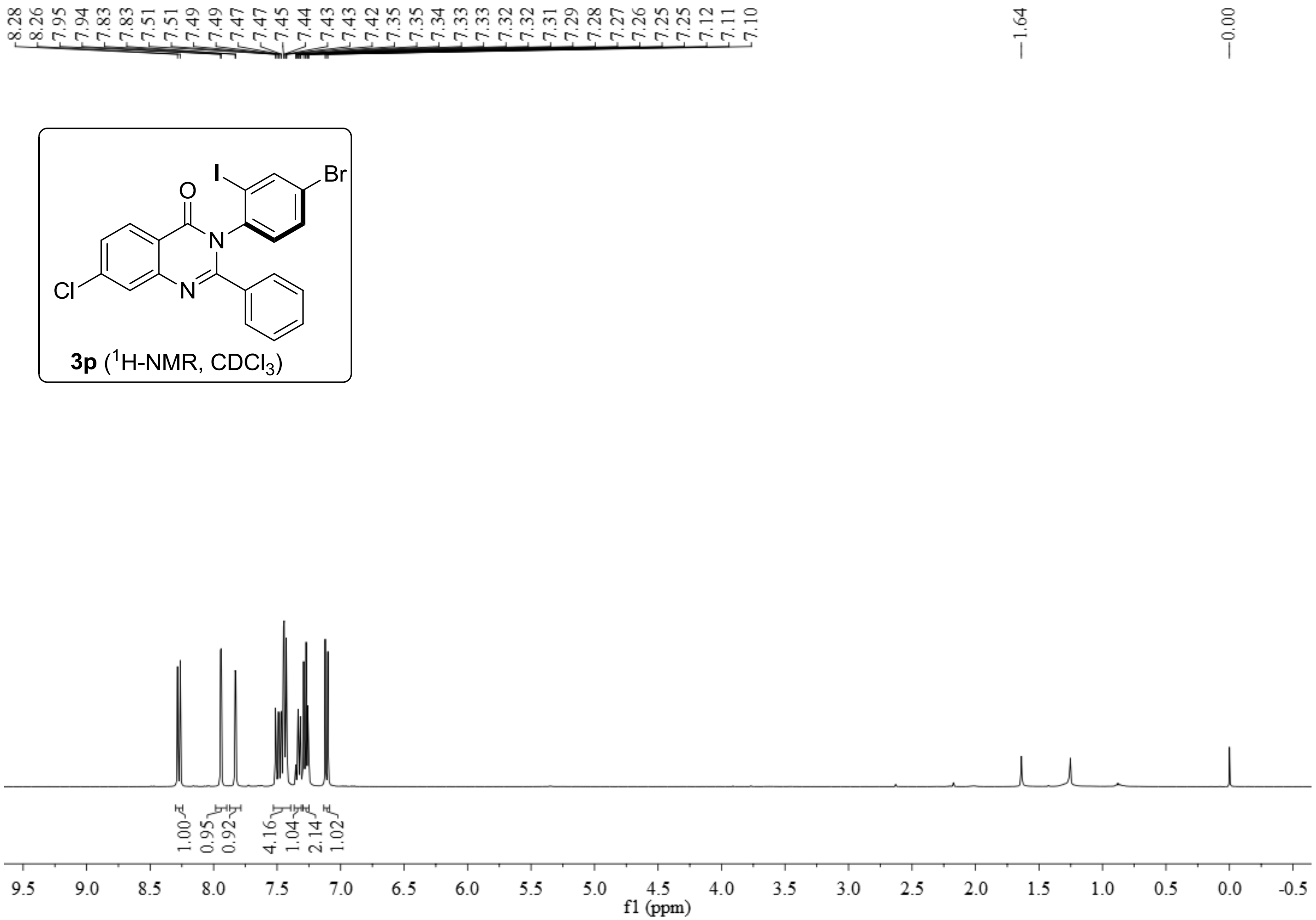


**Supplementary Figure 98.** $^1$H NMR of **3p**

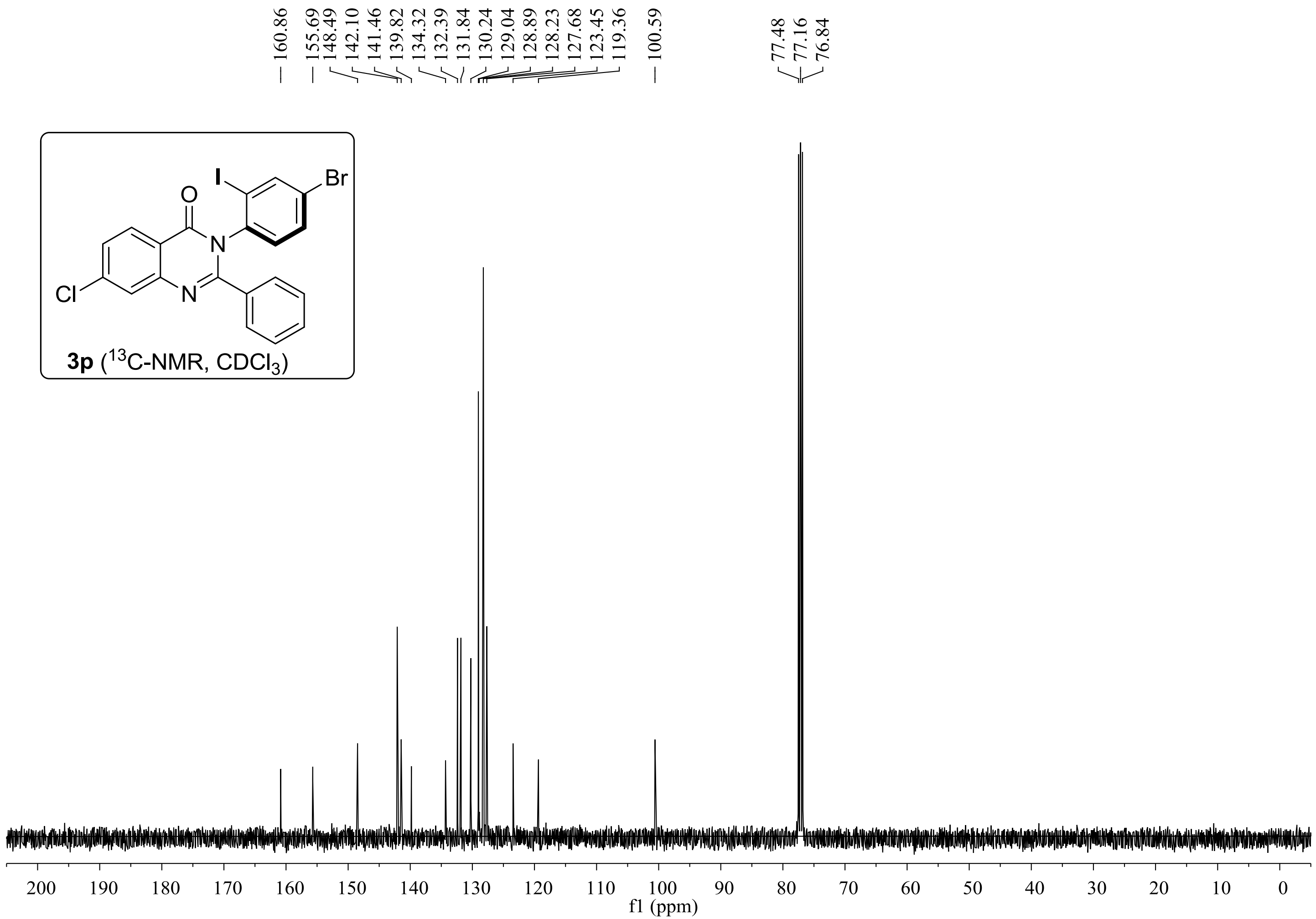


**Supplementary Figure 99.** $^{13}$C NMR of **3p**

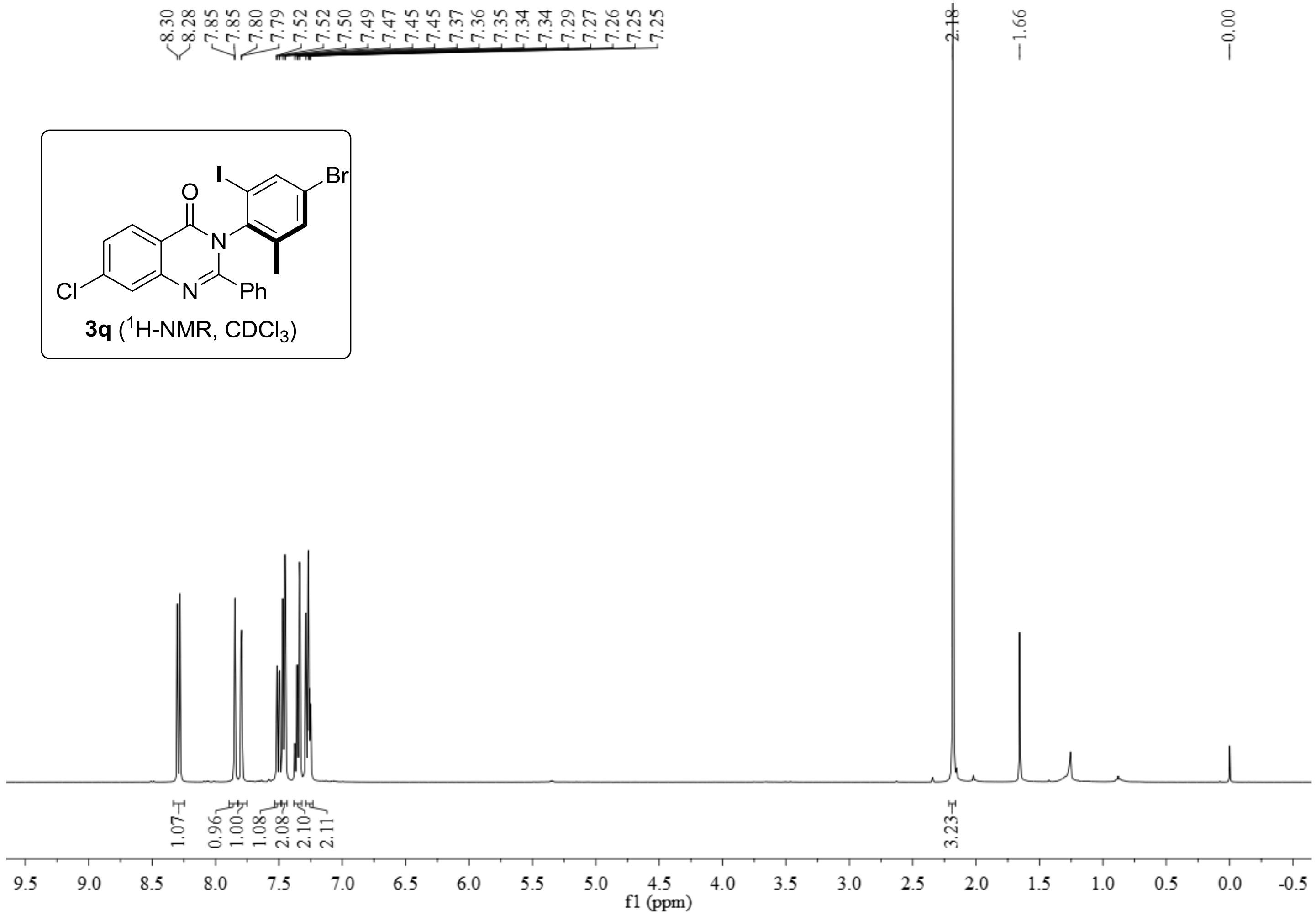


**Supplementary Figure 100.** $^1$H NMR of **3q**

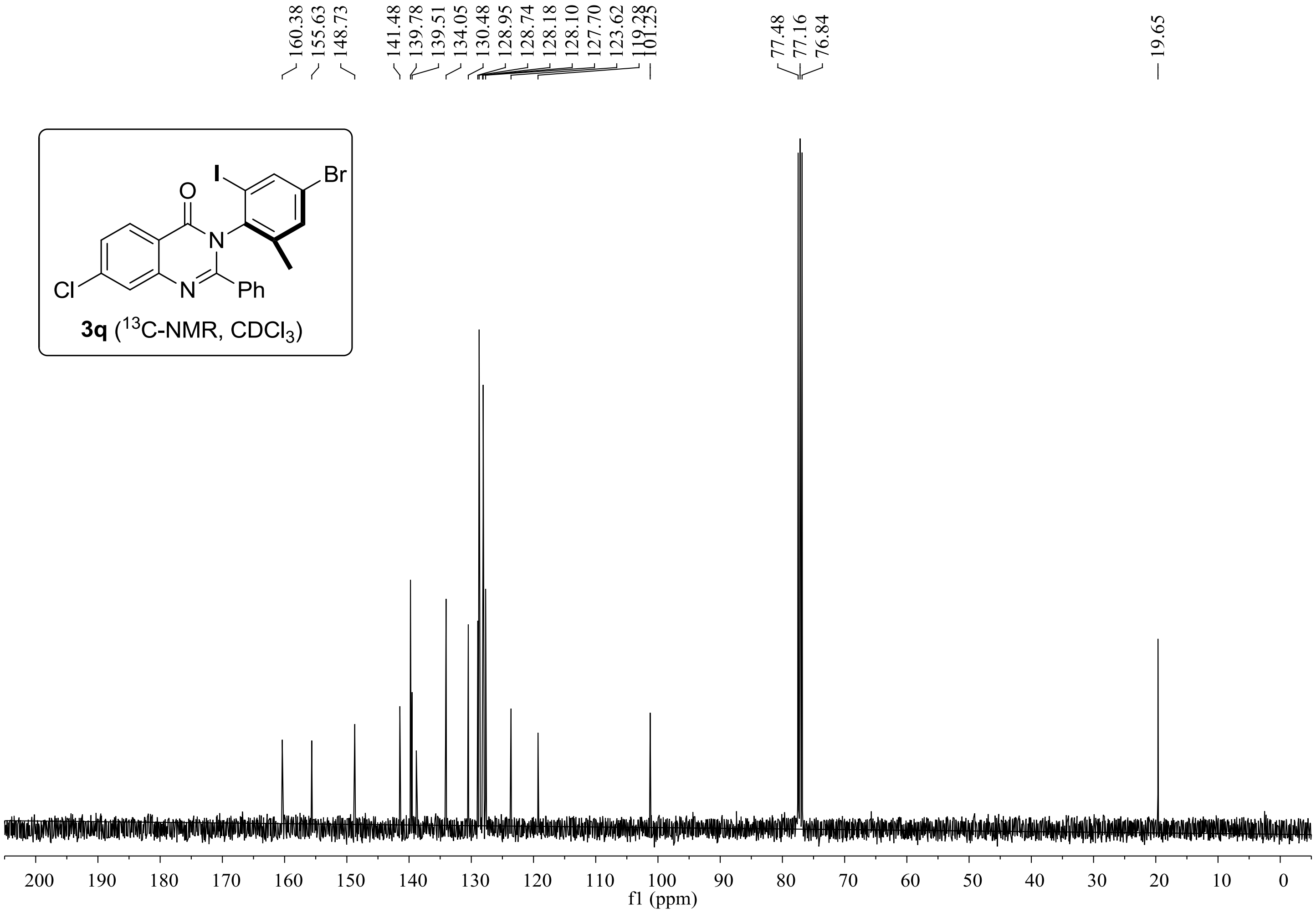


**Supplementary Figure 101.** $^{13}$C NMR of **3q**

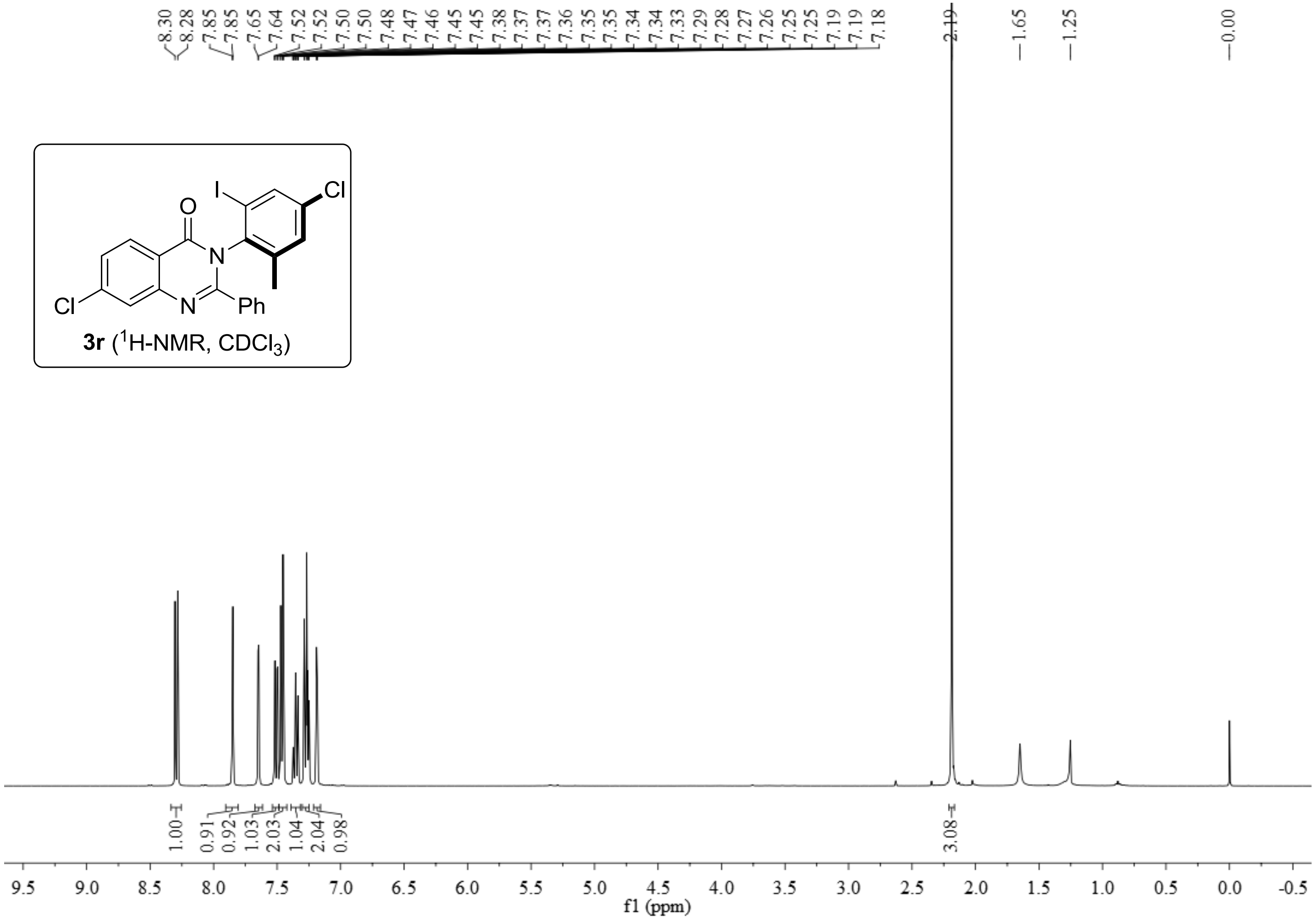


**Supplementary Figure 102.** $^1$H NMR of **3r**

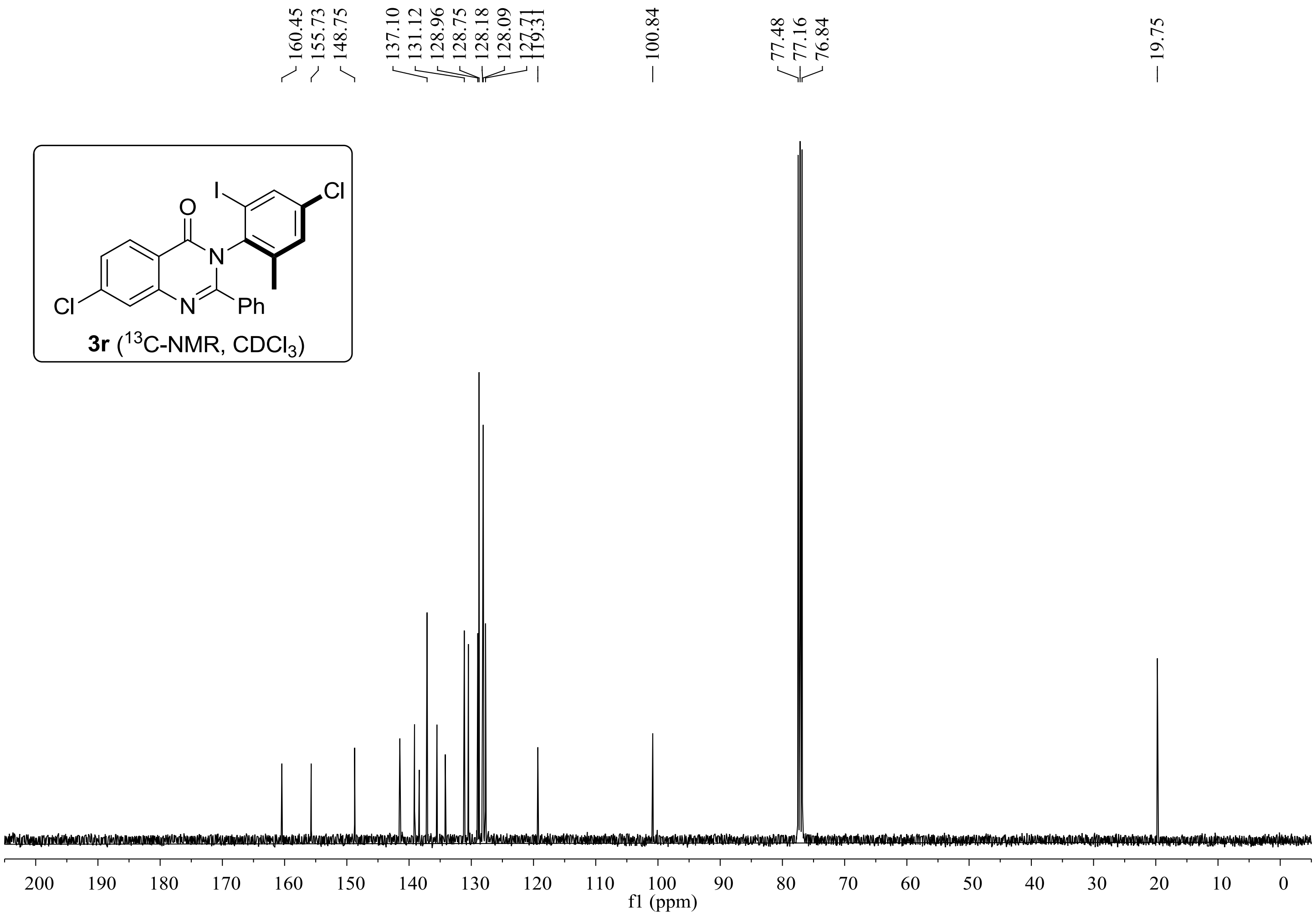


**Supplementary Figure 103.** $^{13}$C NMR of **3r**

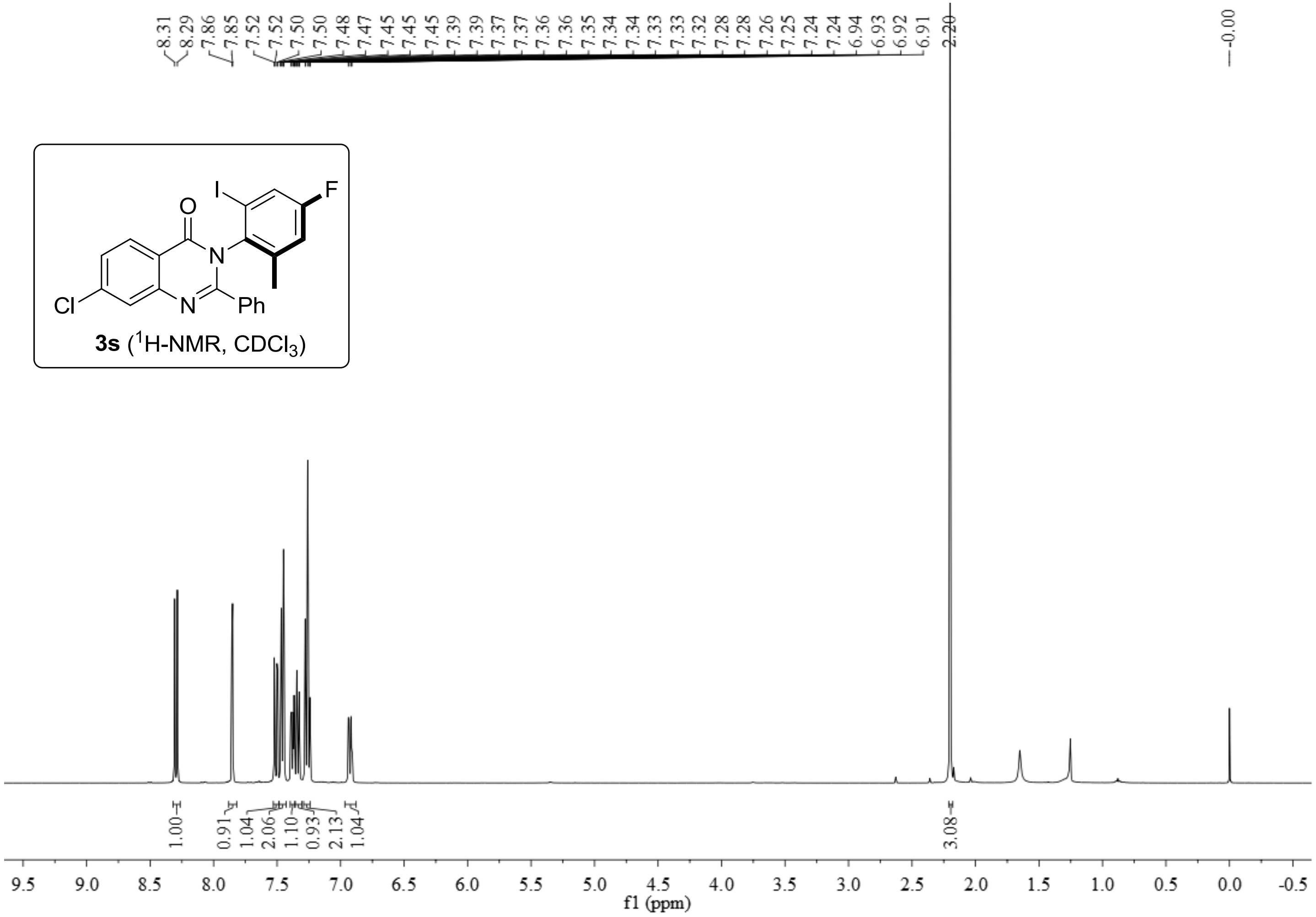


**Supplementary Figure 104.** $^{1}$H NMR of **3s**

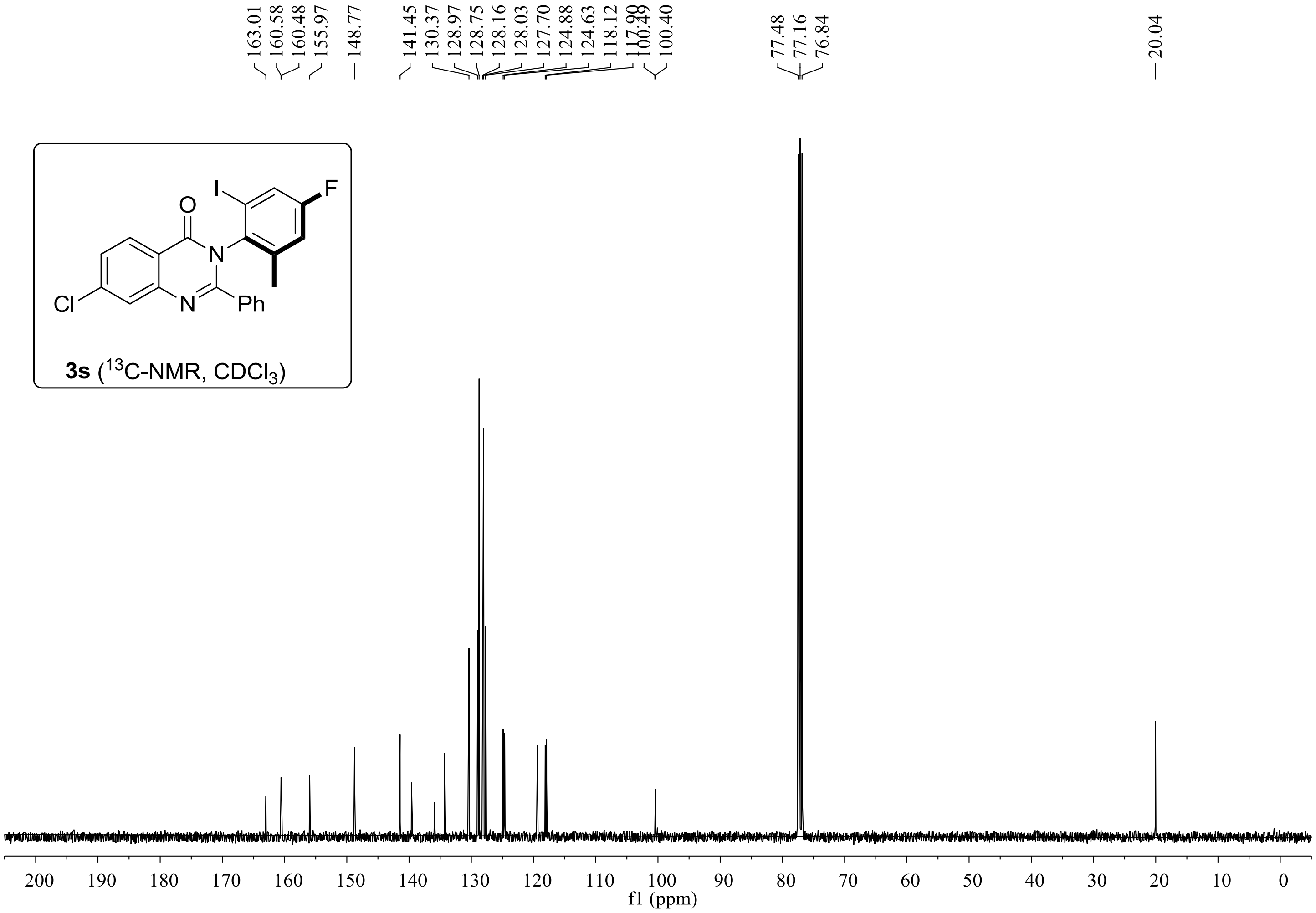


**Supplementary Figure 105.** $^{13}$C NMR of **3s**

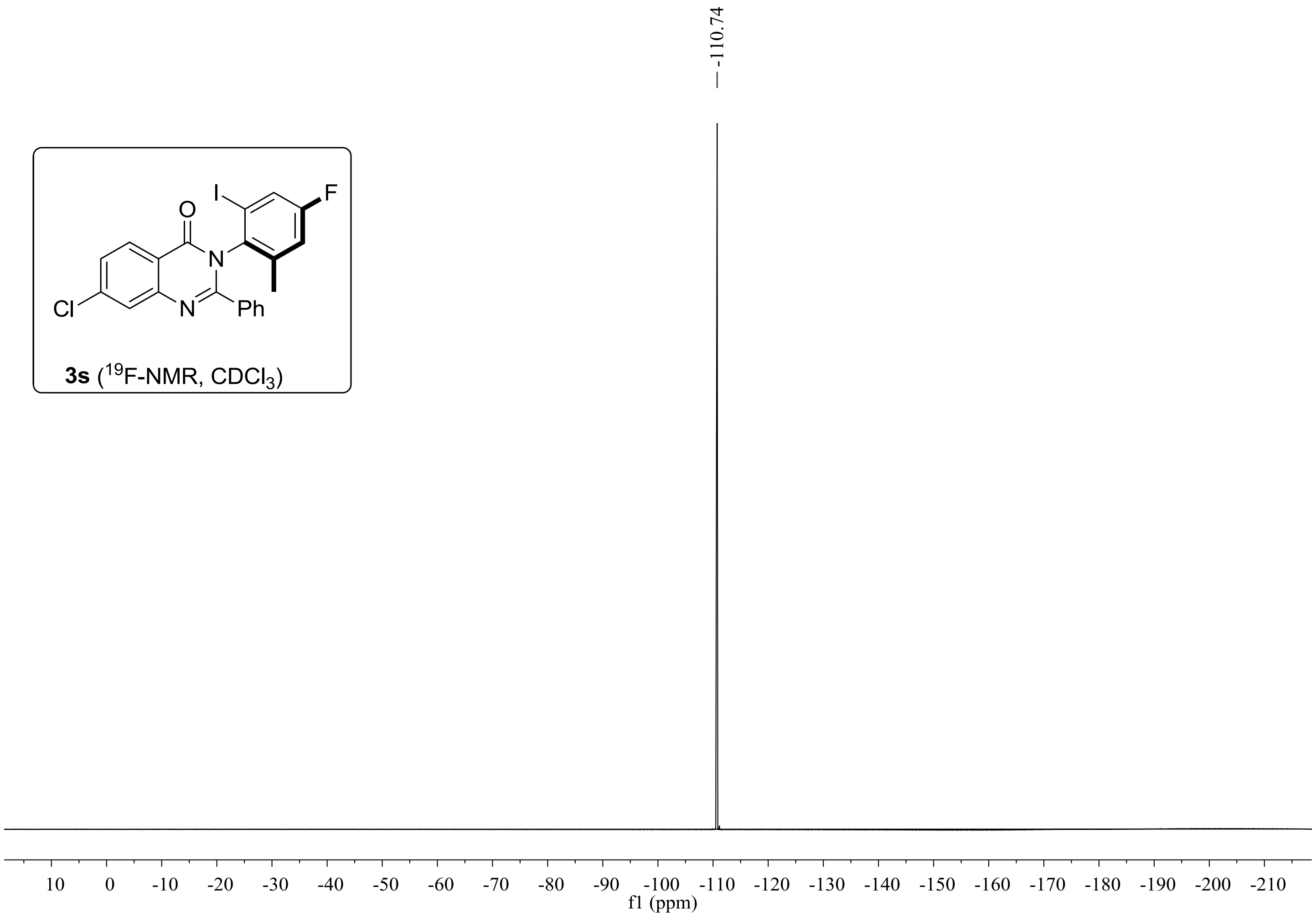


**Supplementary Figure 106.** $^{19}$F NMR of **3s**

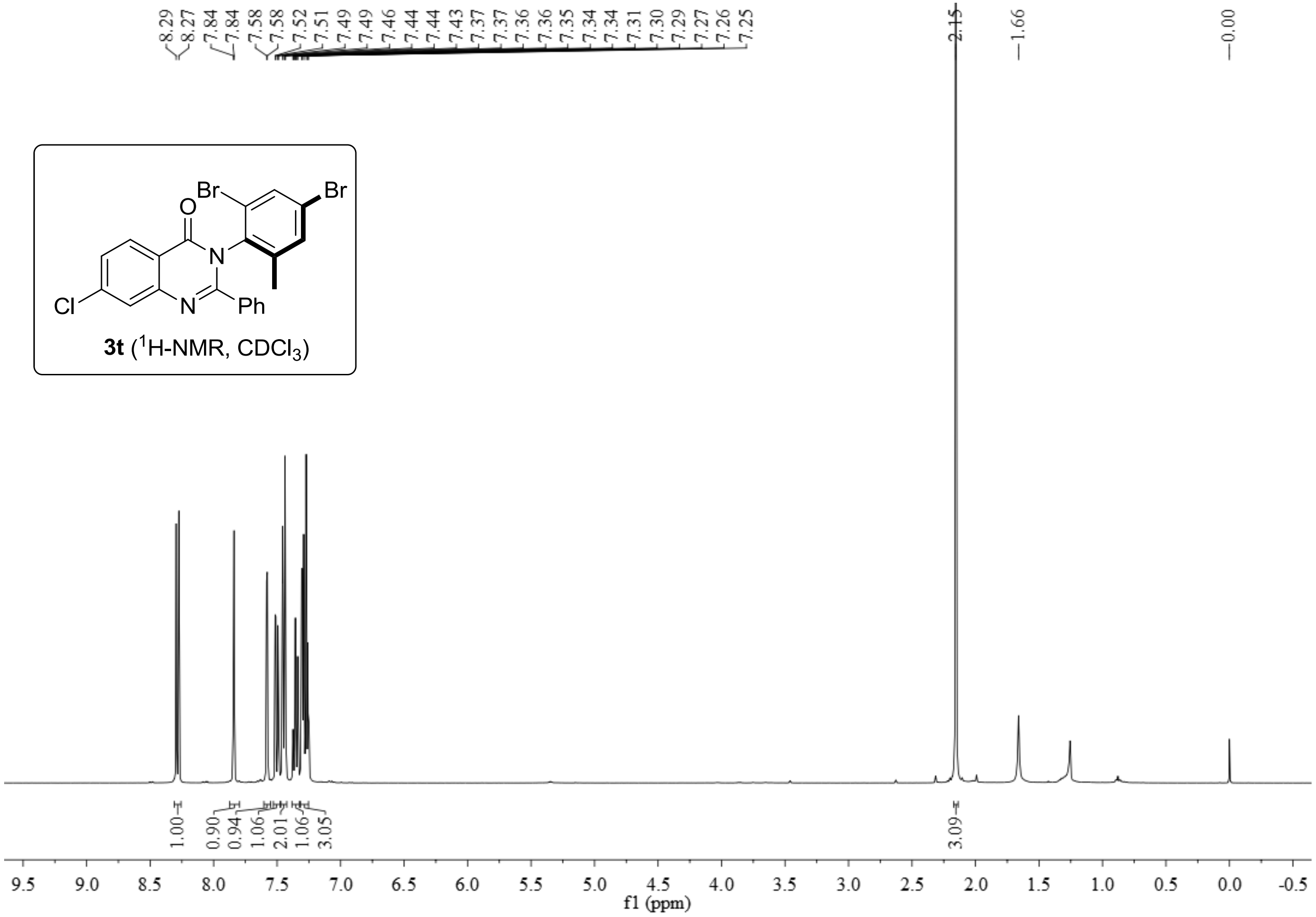


**Supplementary Figure 107.** $^{1}$H NMR of **3t**

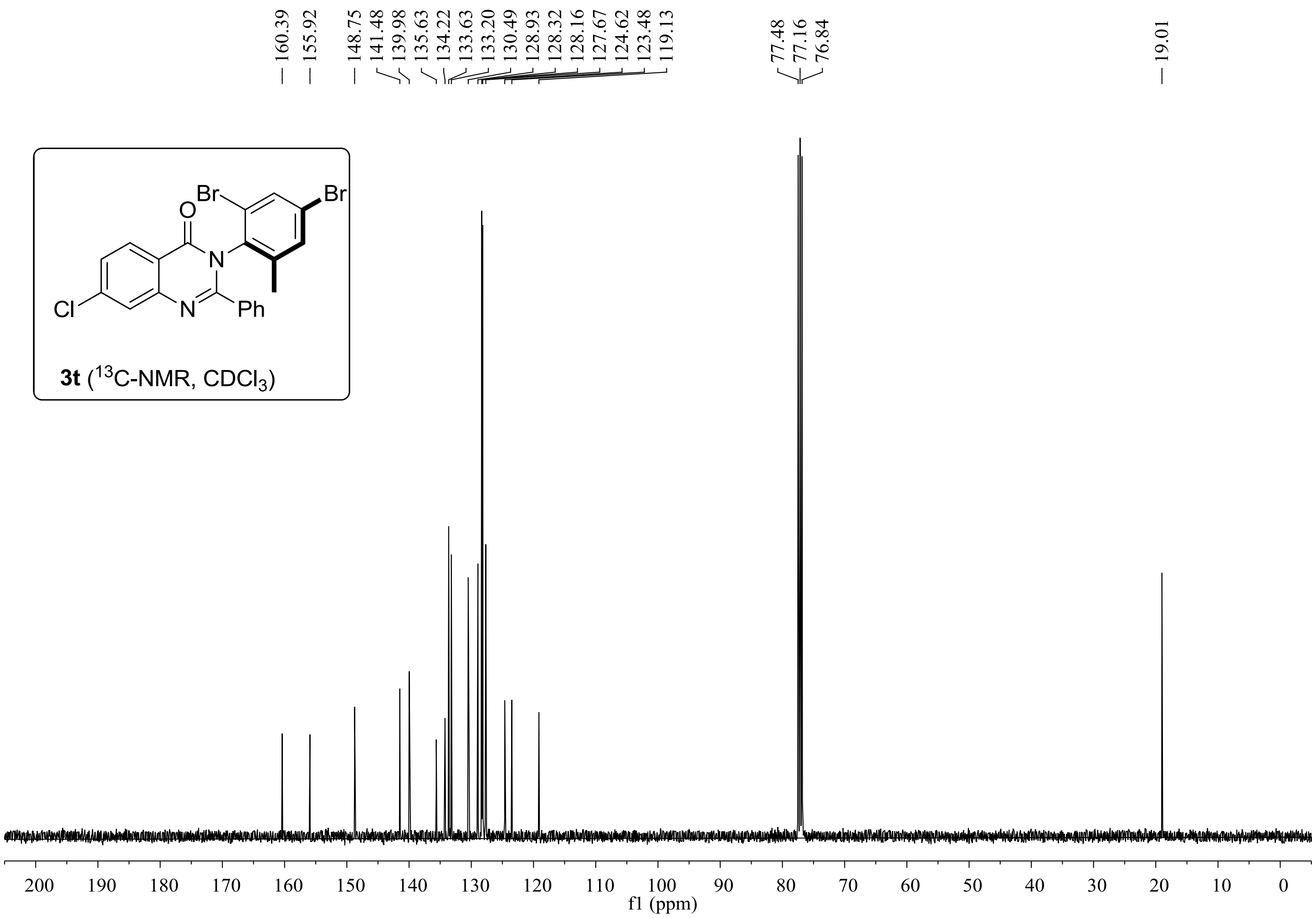


**Supplementary Figure 108.** $^{13}$C NMR of **3t**

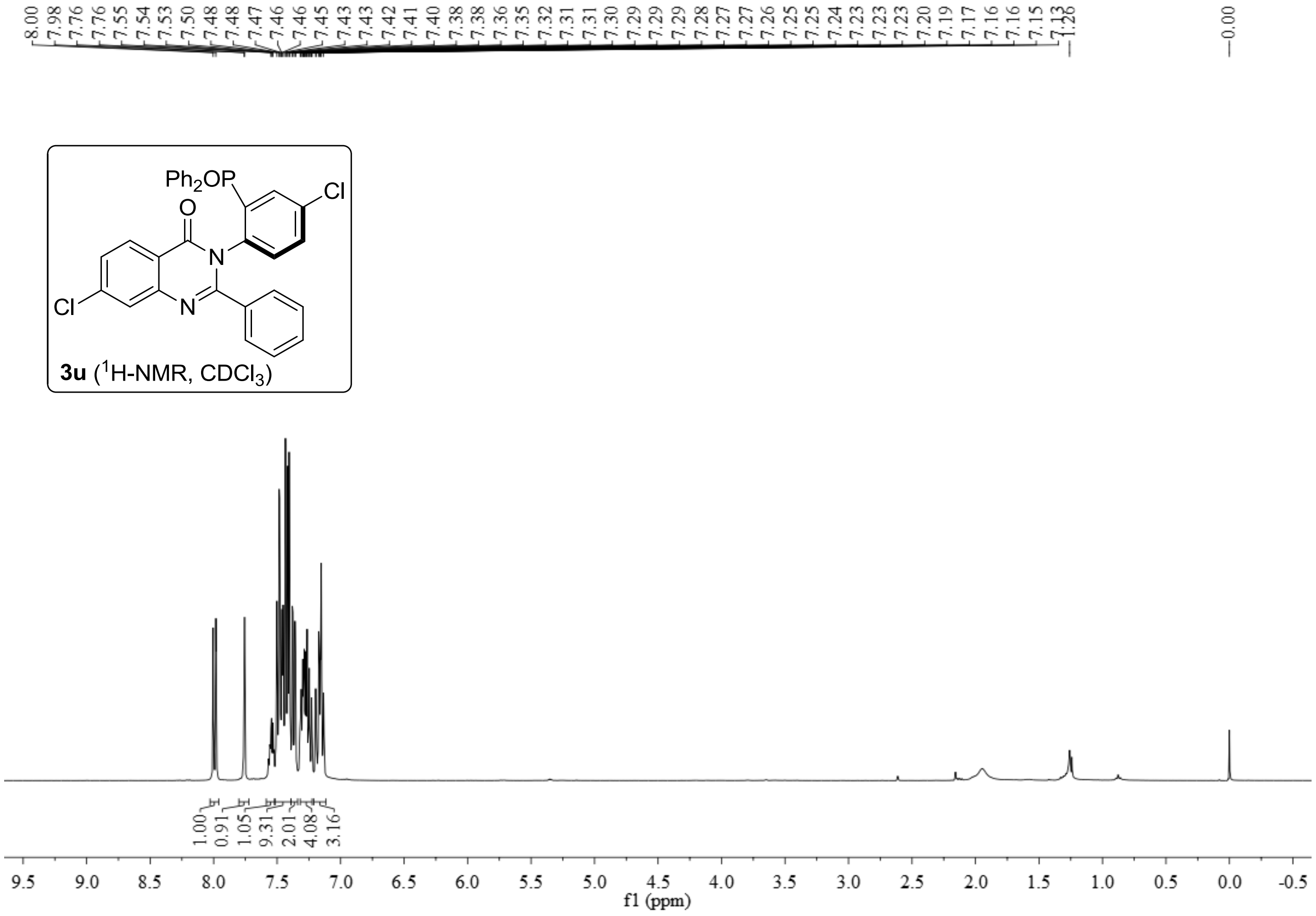

**Supplementary Figure 109.** $^1$H NMR of **3u**

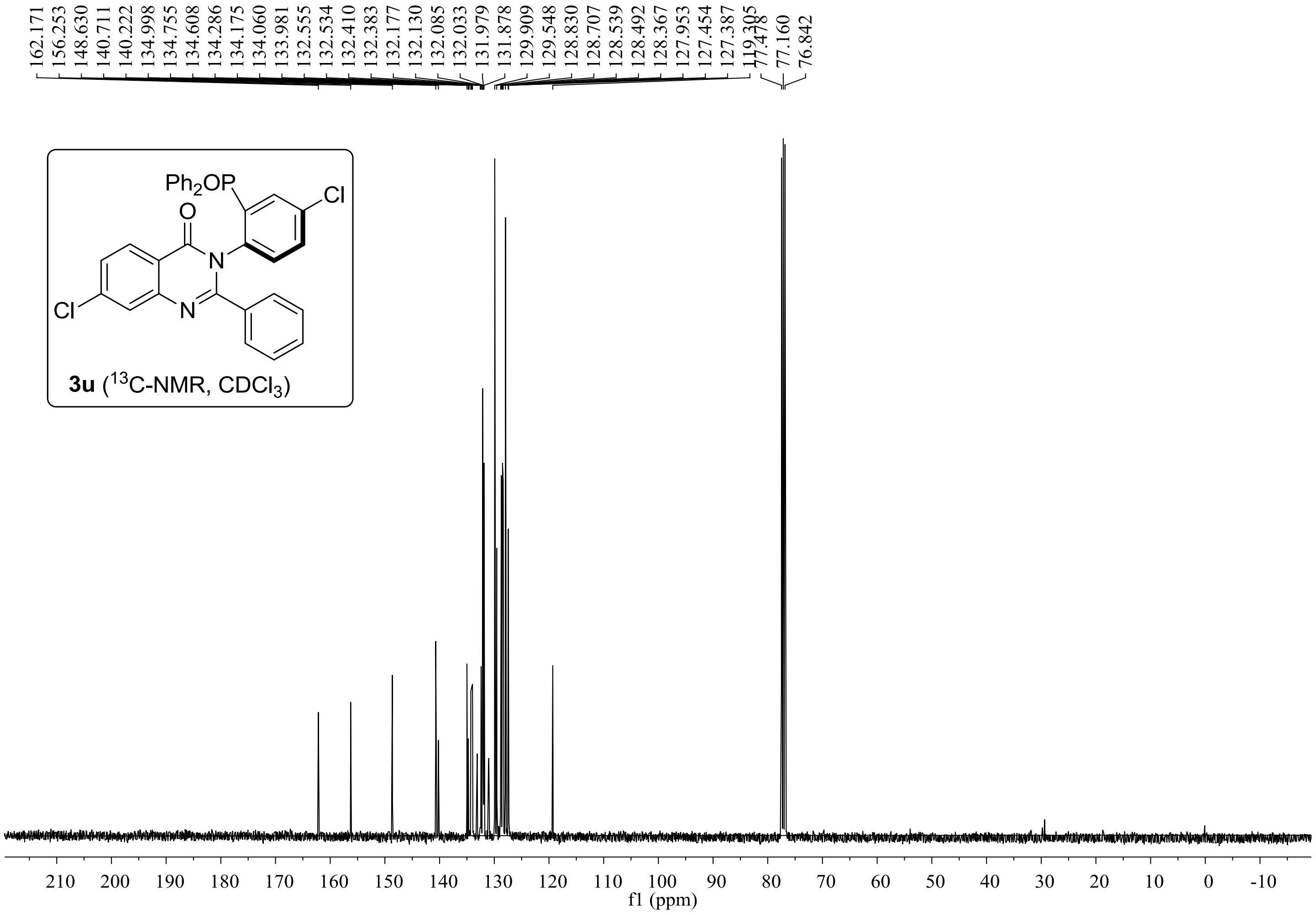


**Supplementary Figure 110.** $^{13}$C NMR of **3u**

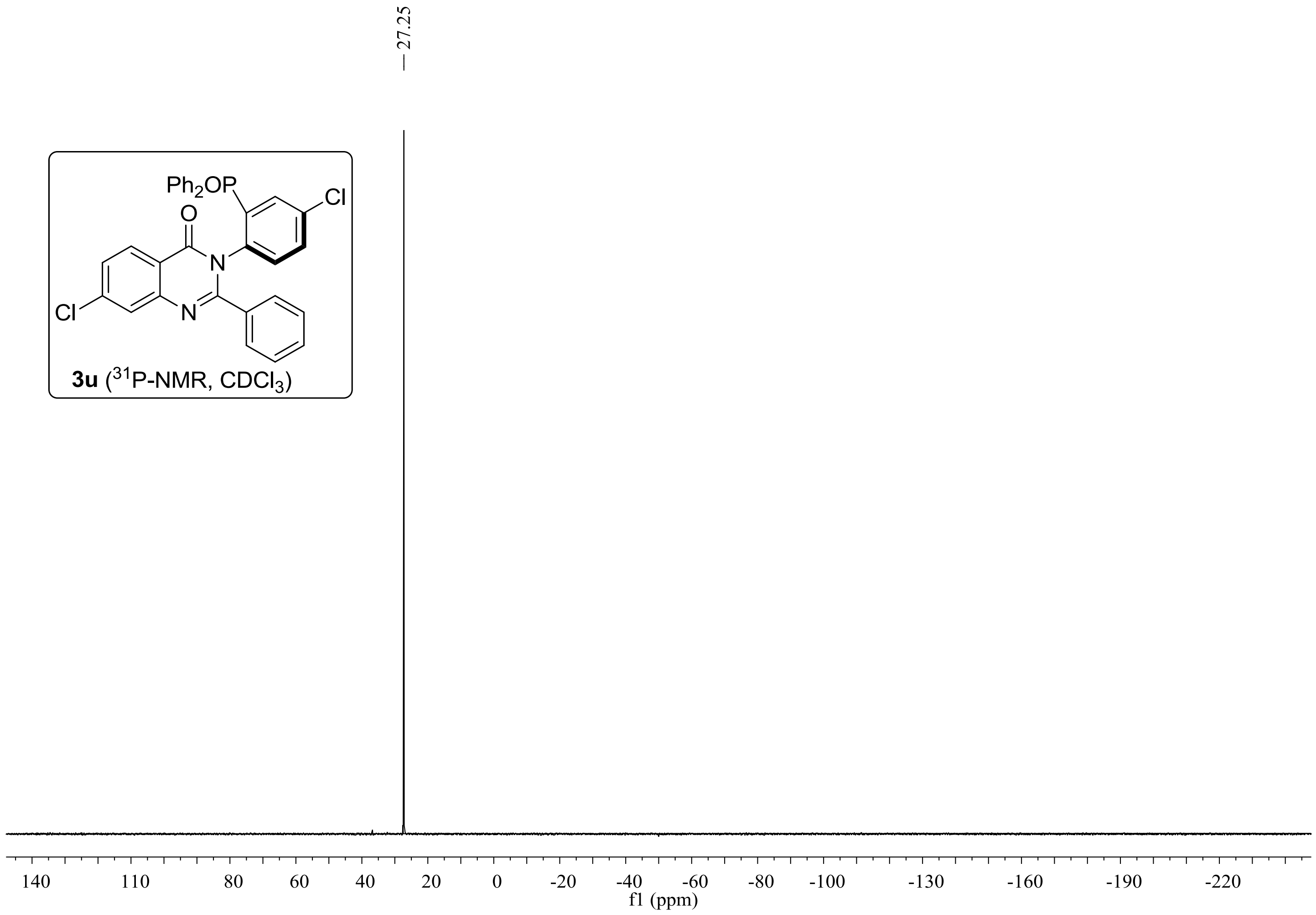


**Supplementary Figure 111.** $^{19}$F NMR of **3u**

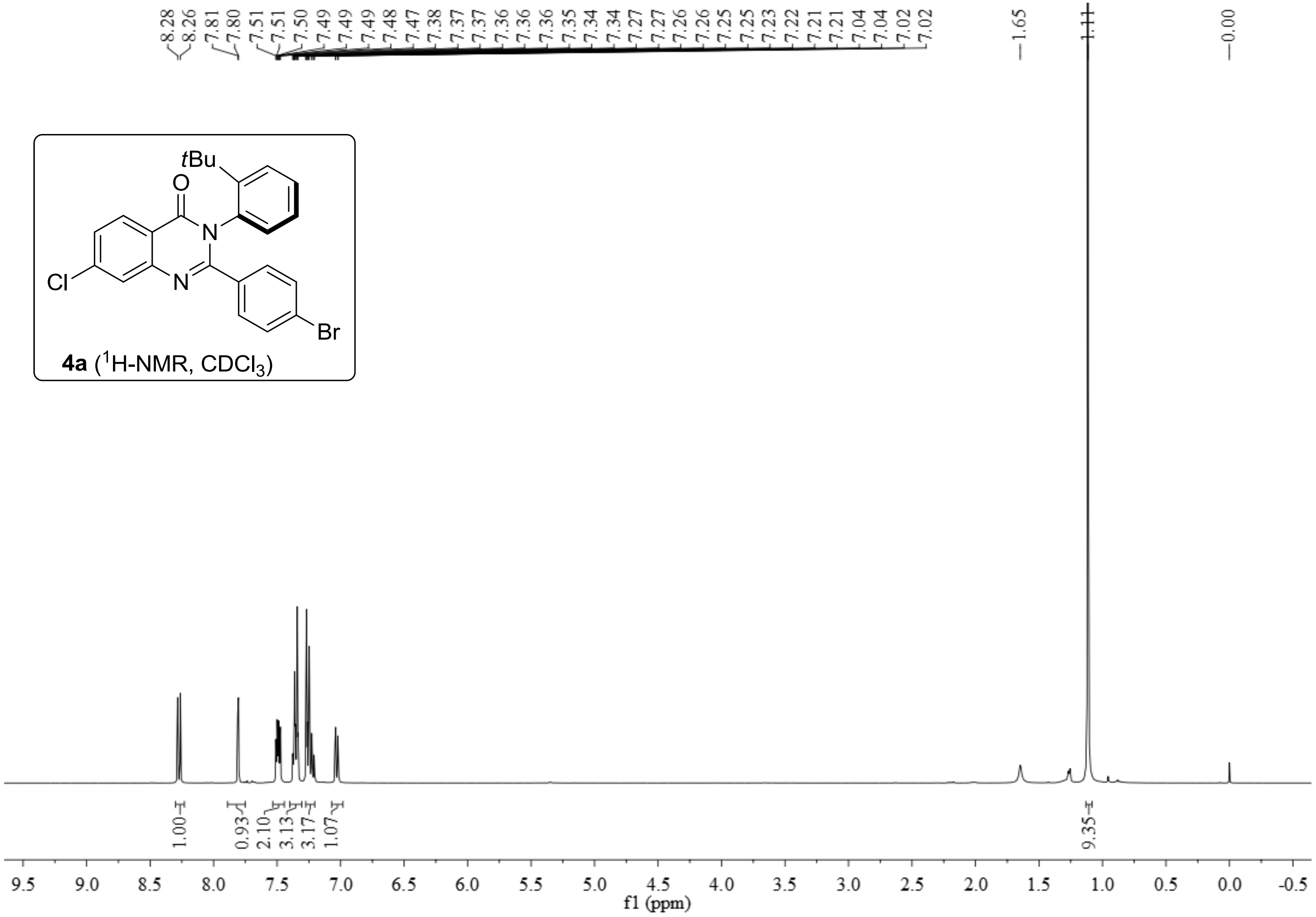


**Supplementary Figure 112.** $^1$H NMR of **4a**

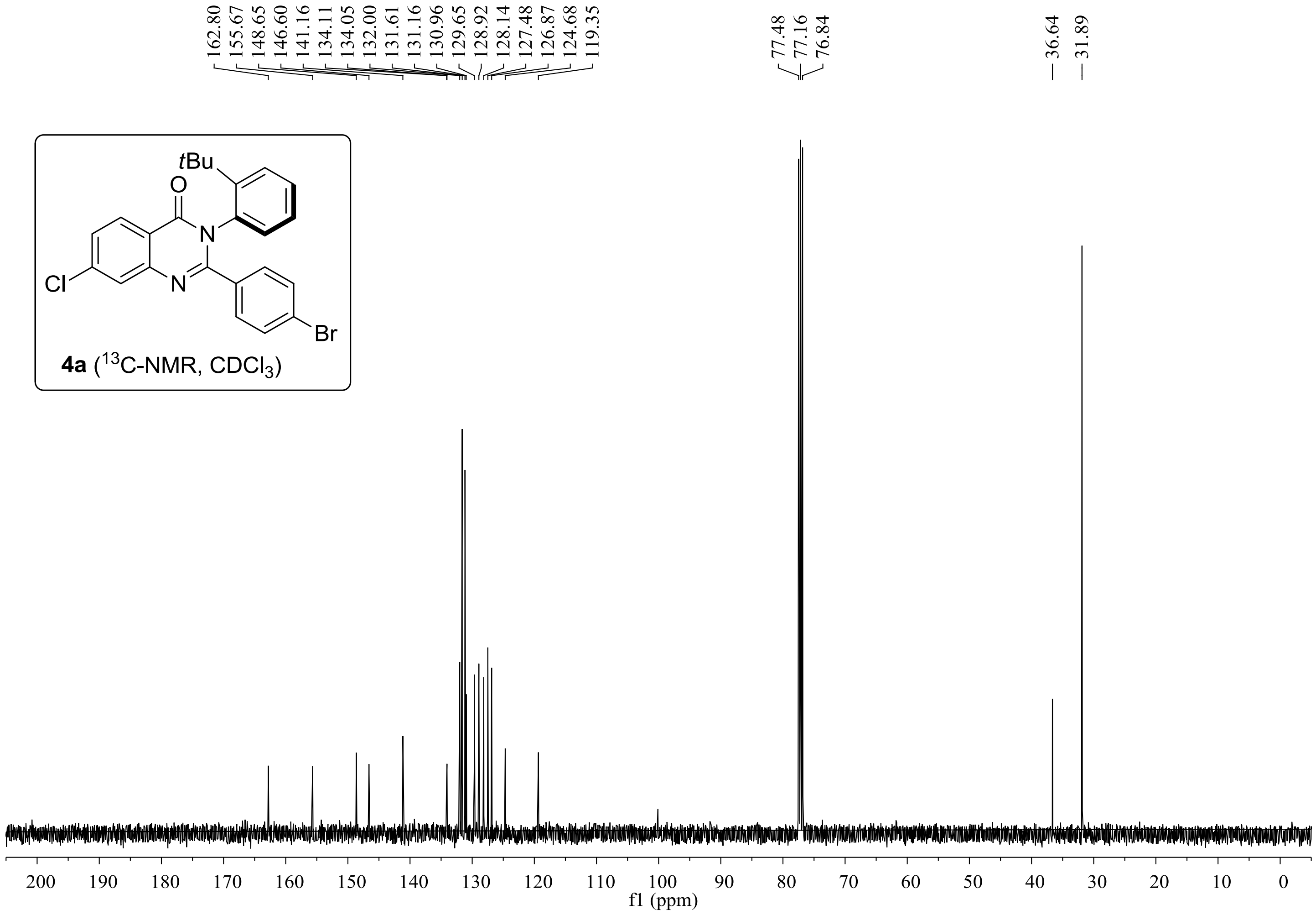


**Supplementary Figure 1113.** $^{13}$C NMR of **4a**

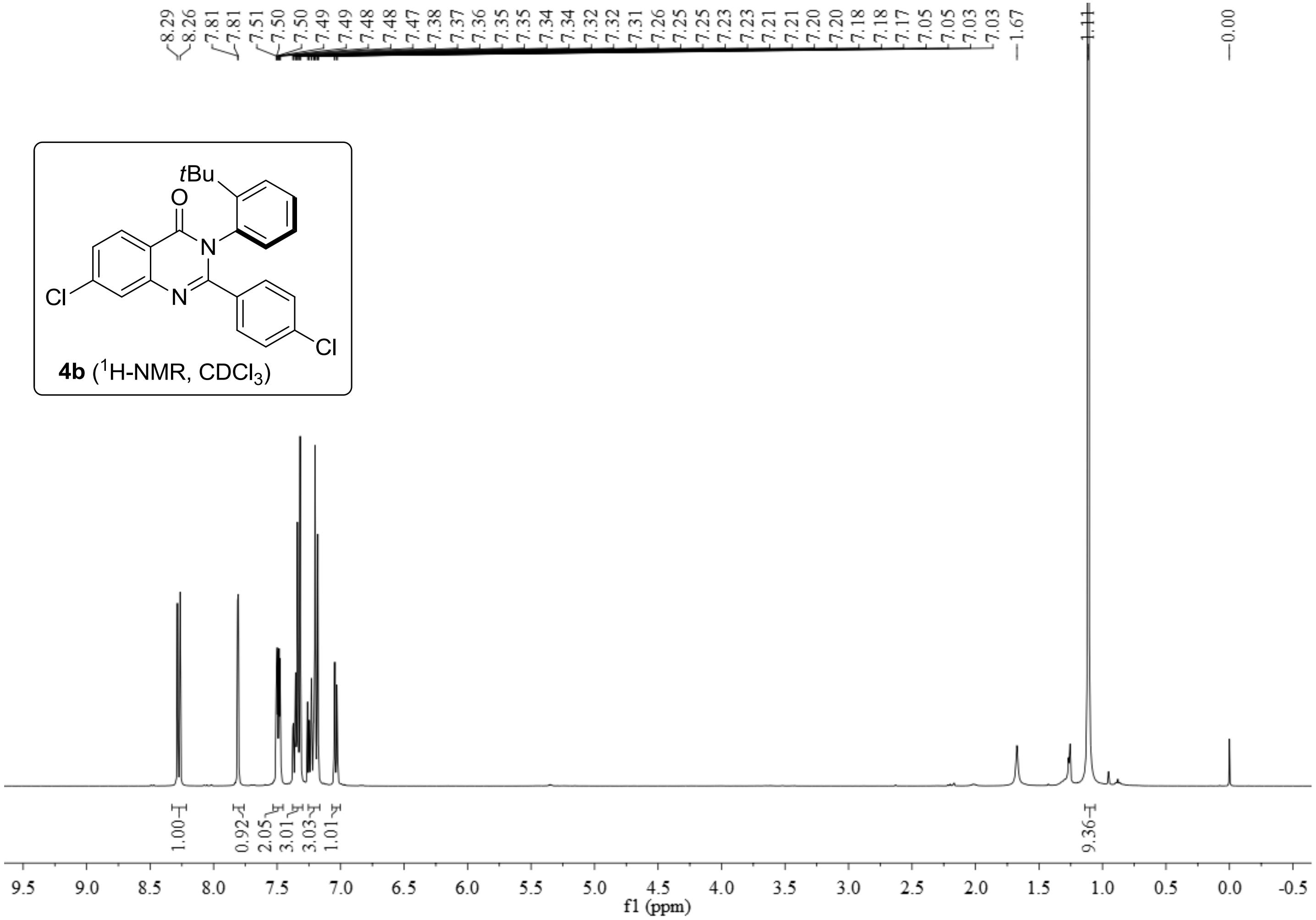


**Supplementary Figure 114.** $^{1}$H NMR of **4b**

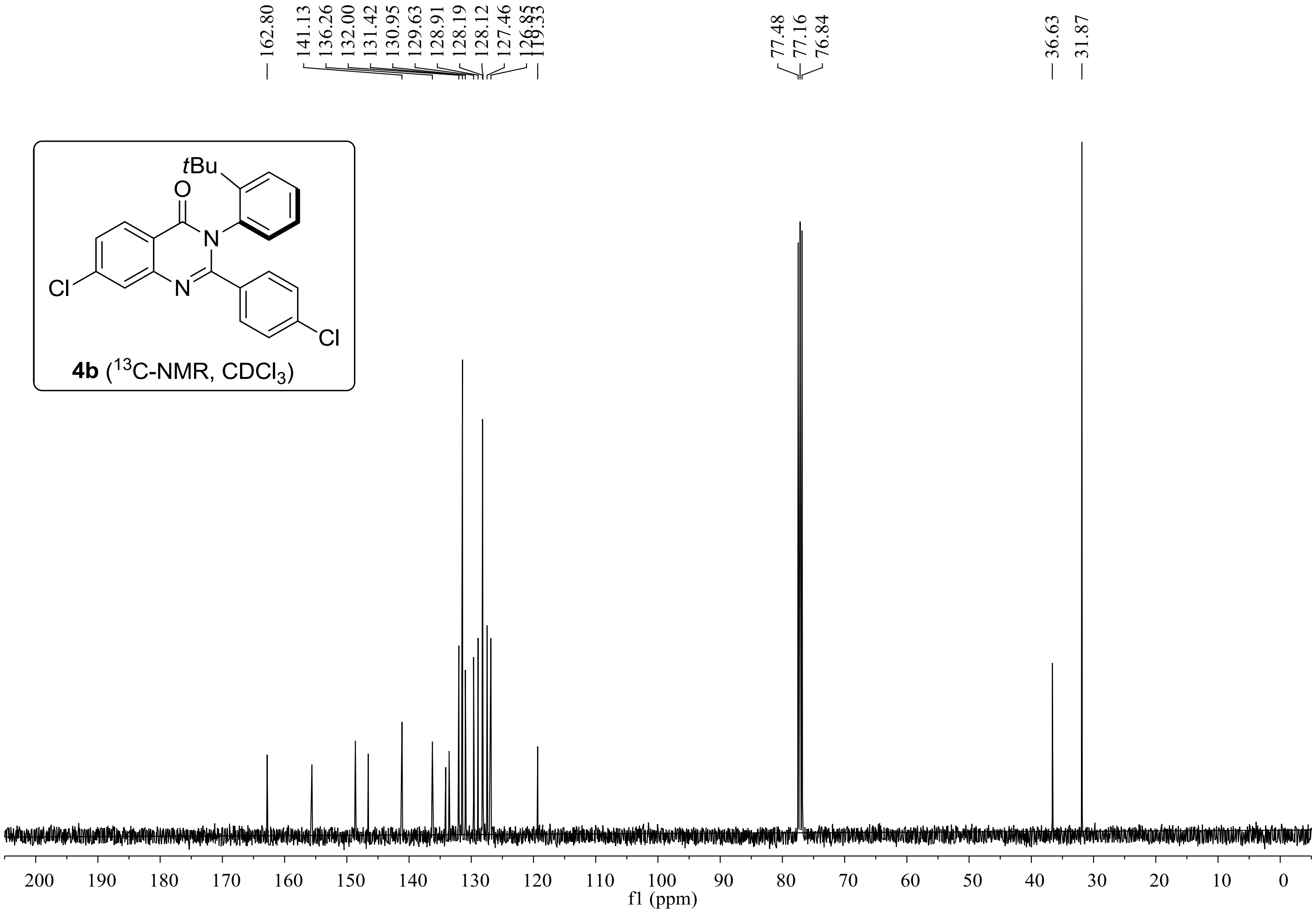


**Supplementary Figure 115.** $^{13}$C NMR of **4b**

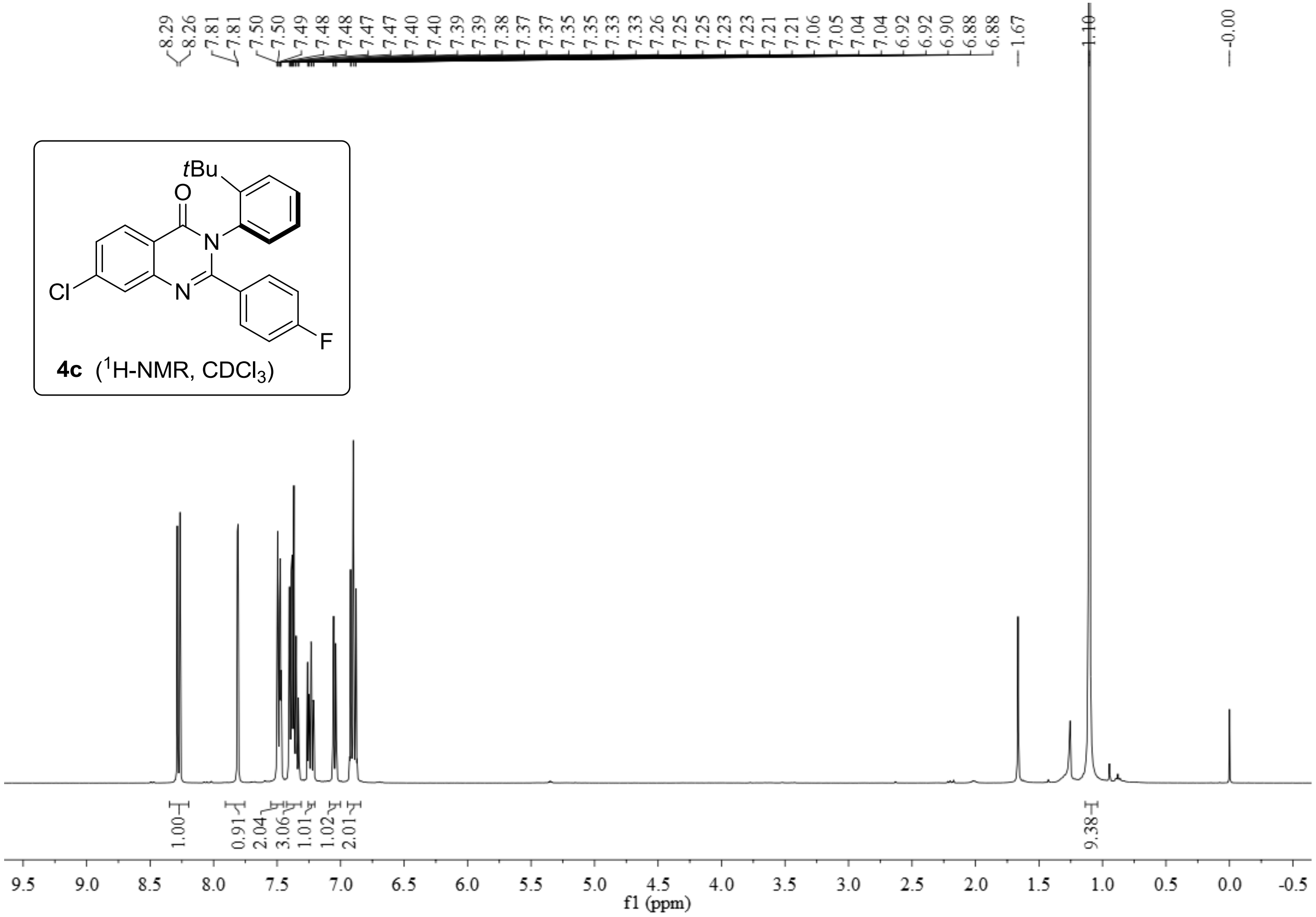


**Supplementary Figure 116.** $^{1}$H NMR of **4c**

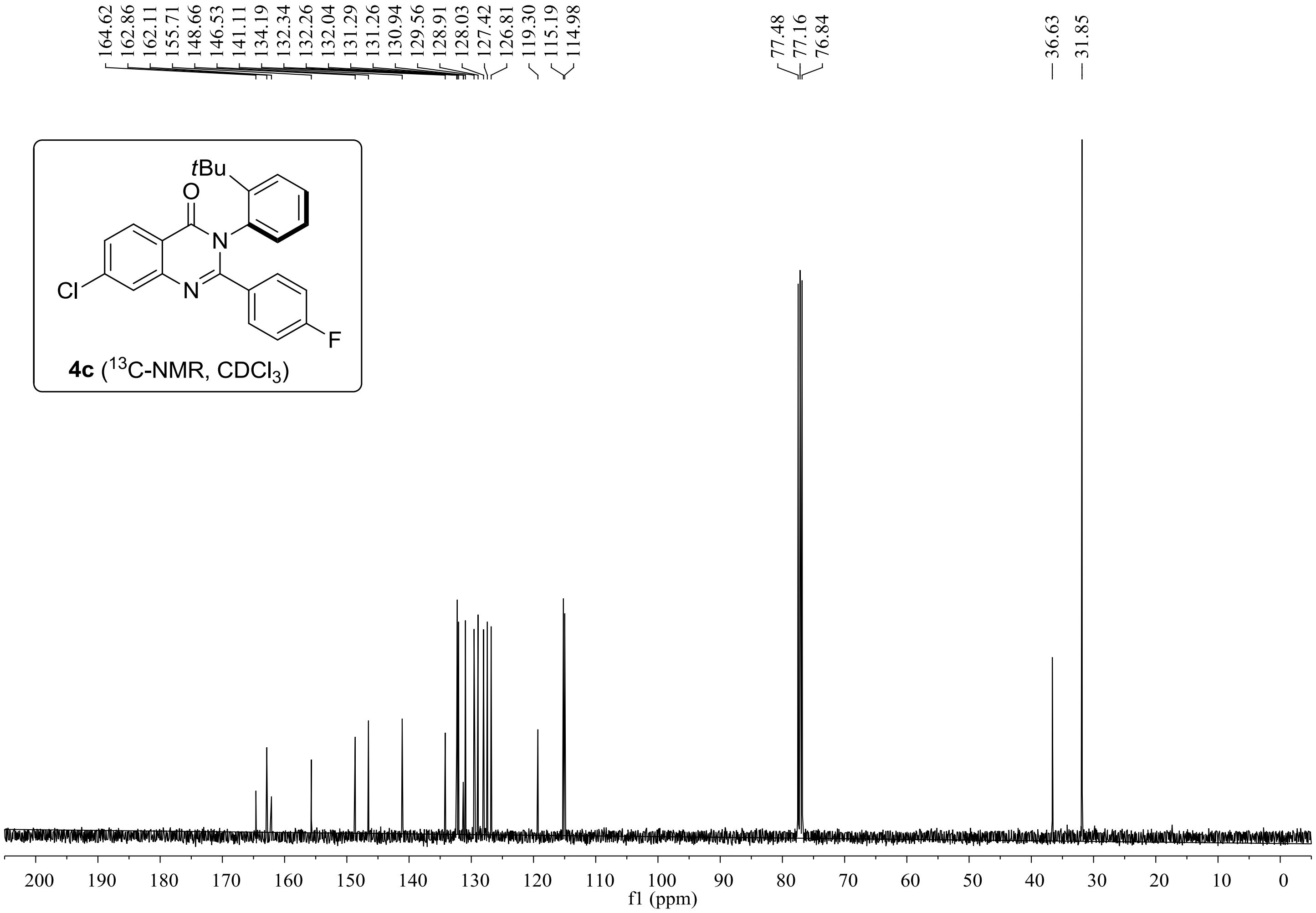


**Supplementary Figure 117.** $^{13}$C NMR of **4c**

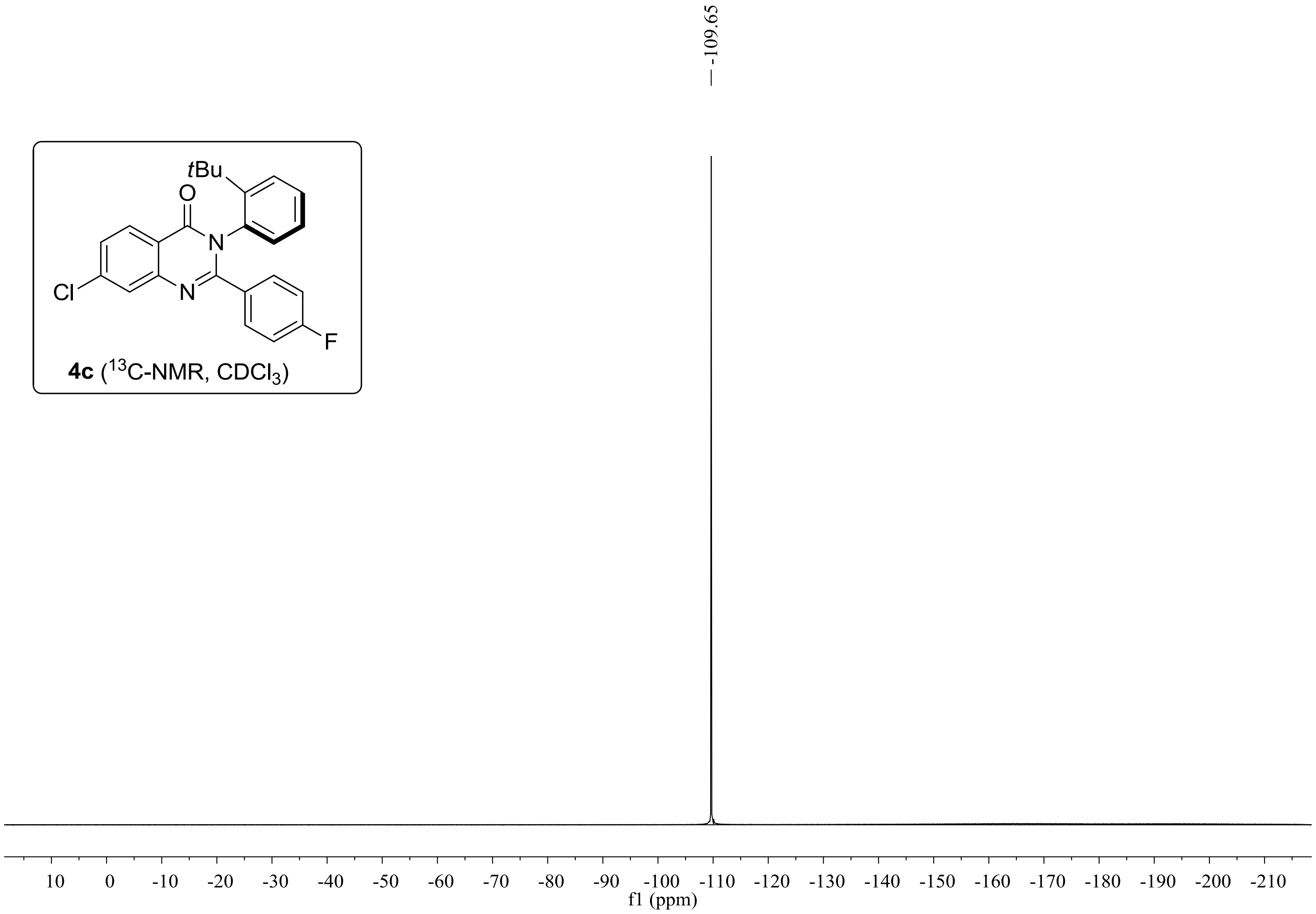


**Supplementary Figure 118.** $^{19}$F NMR of **4c**

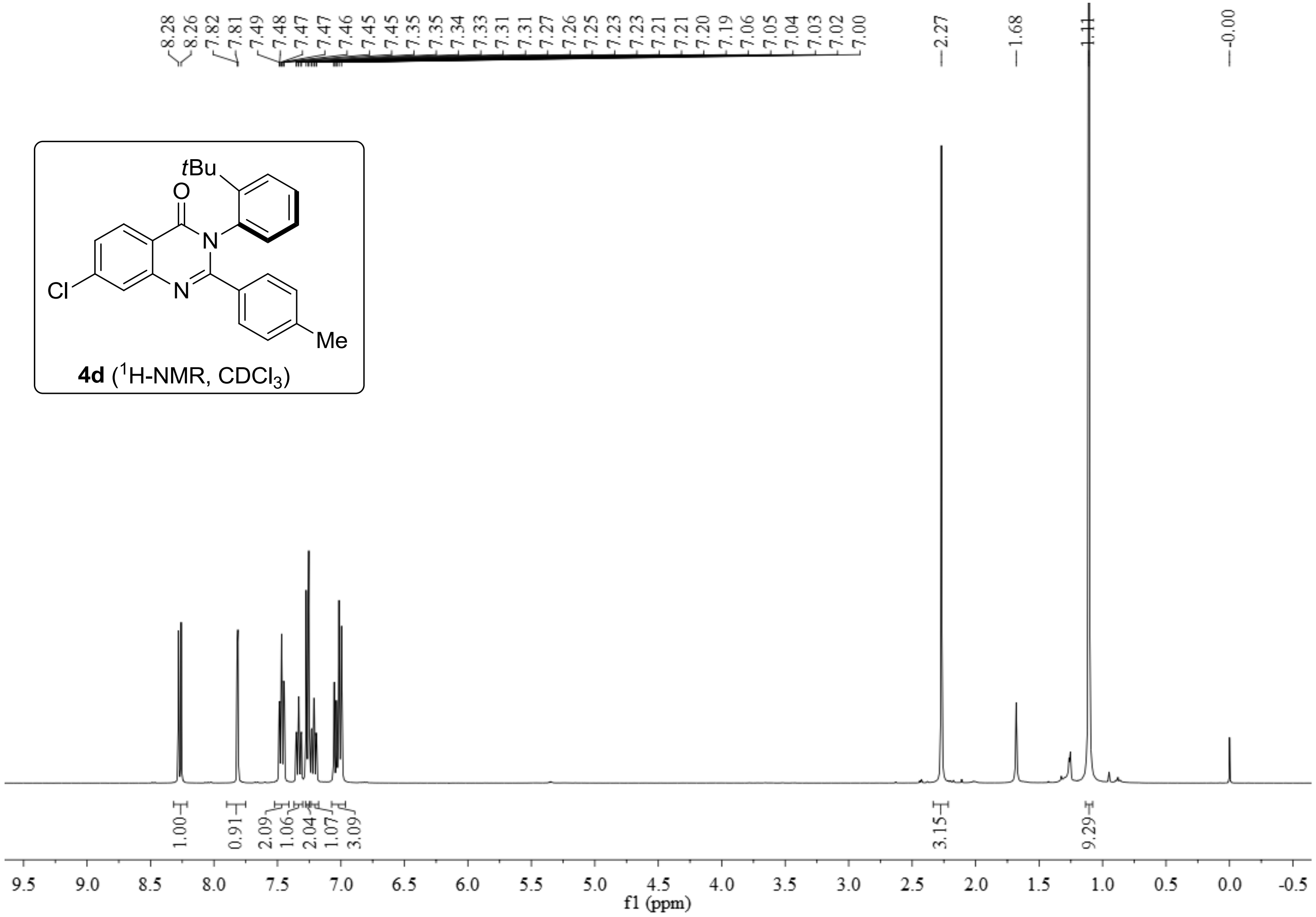


**Supplementary Figure 119.** $^1$H NMR of **4d**

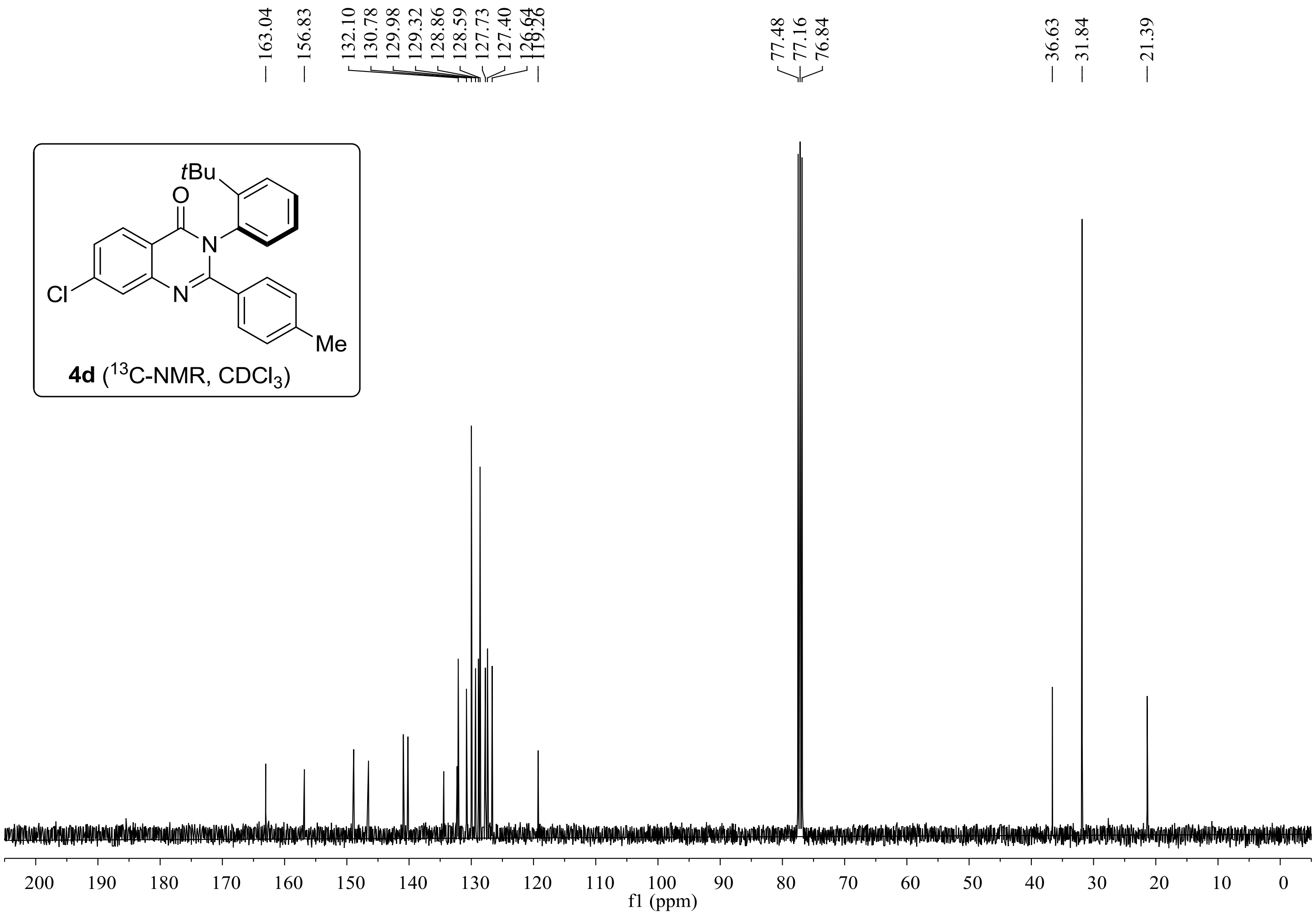


**Supplementary Figure 120.** $^{13}$C NMR of **4d**

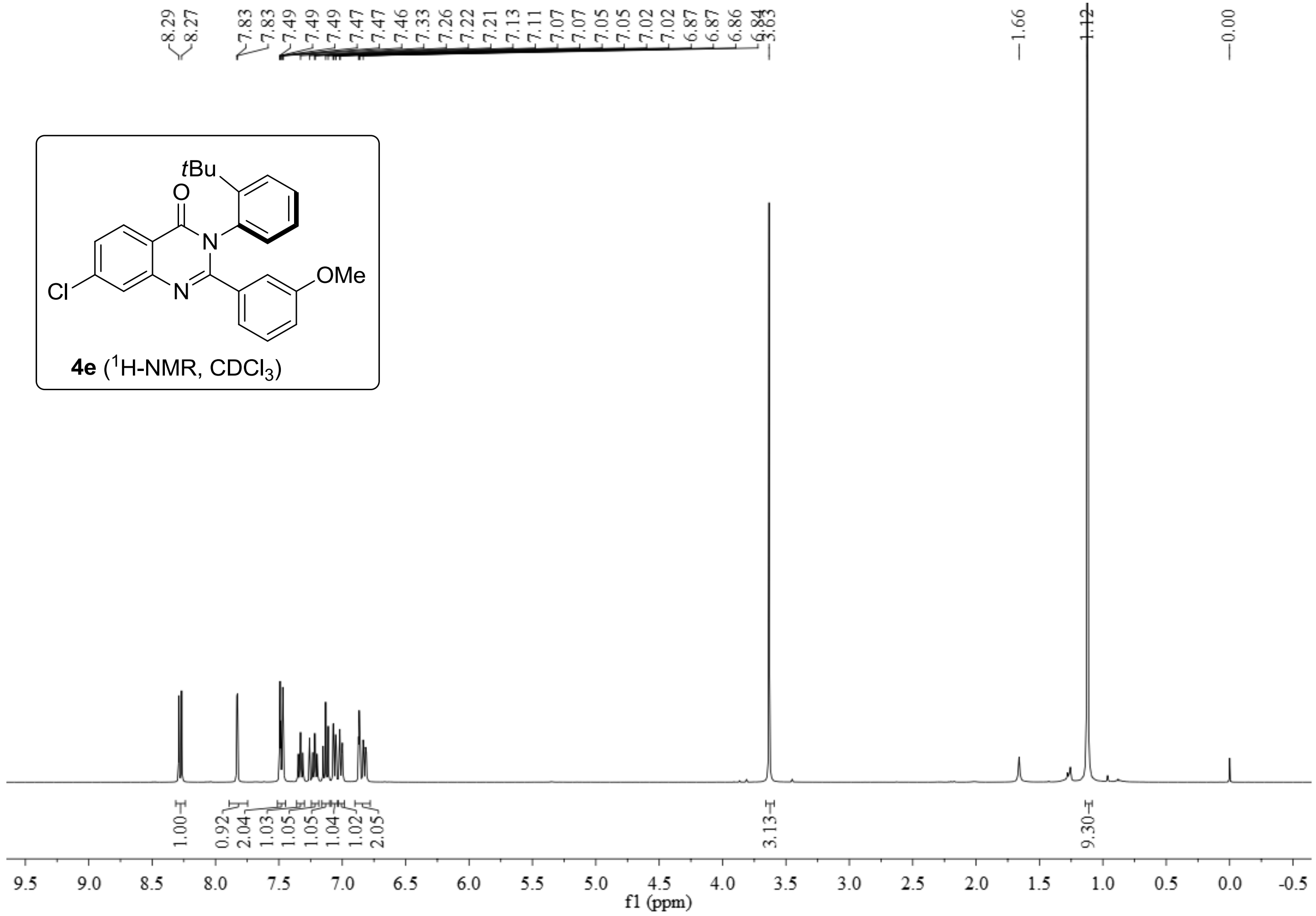


**Supplementary Figure 121.** $^{1}$H NMR of **4e**

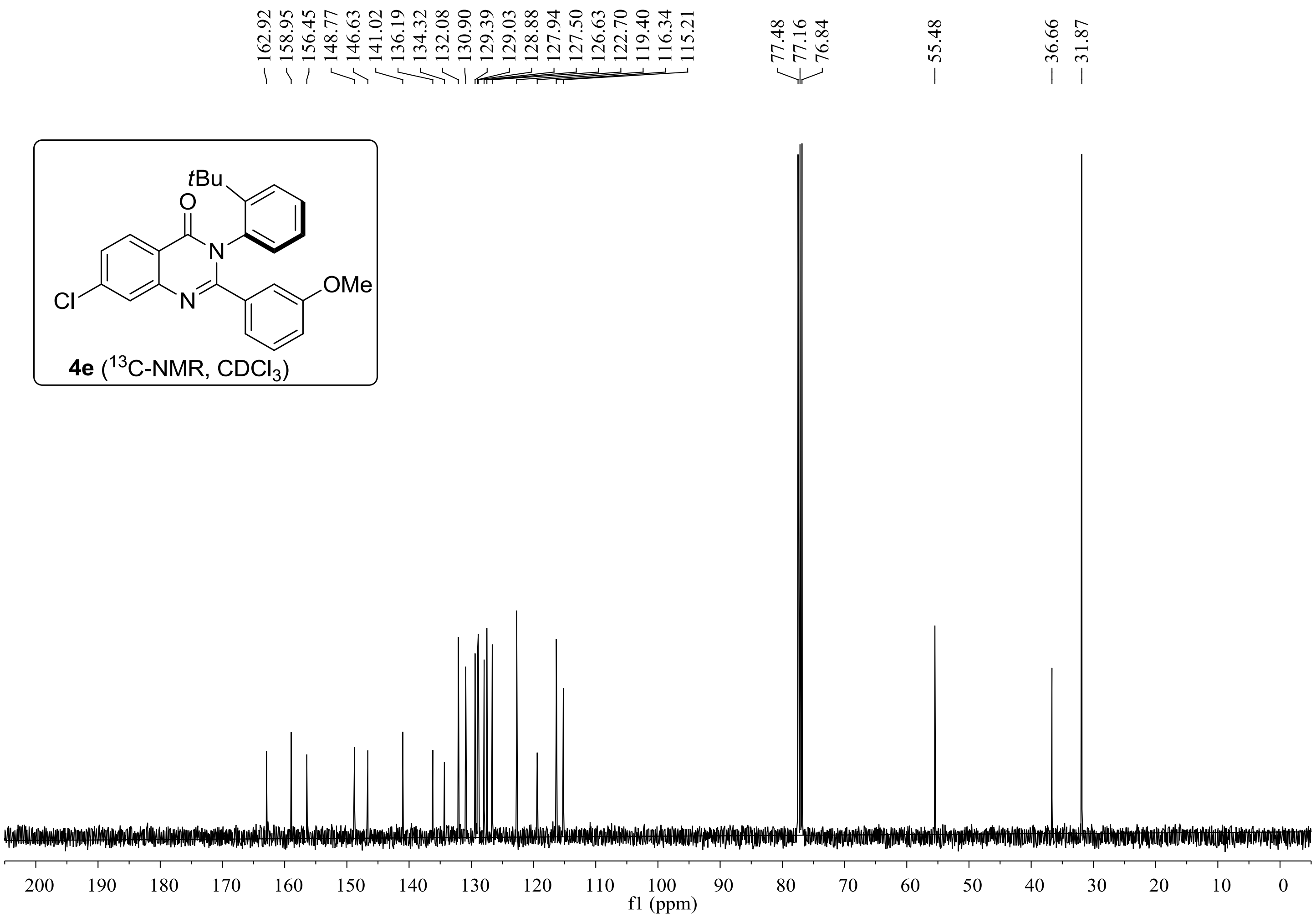


**Supplementary Figure 122.** $^{13}$C NMR of **4e**

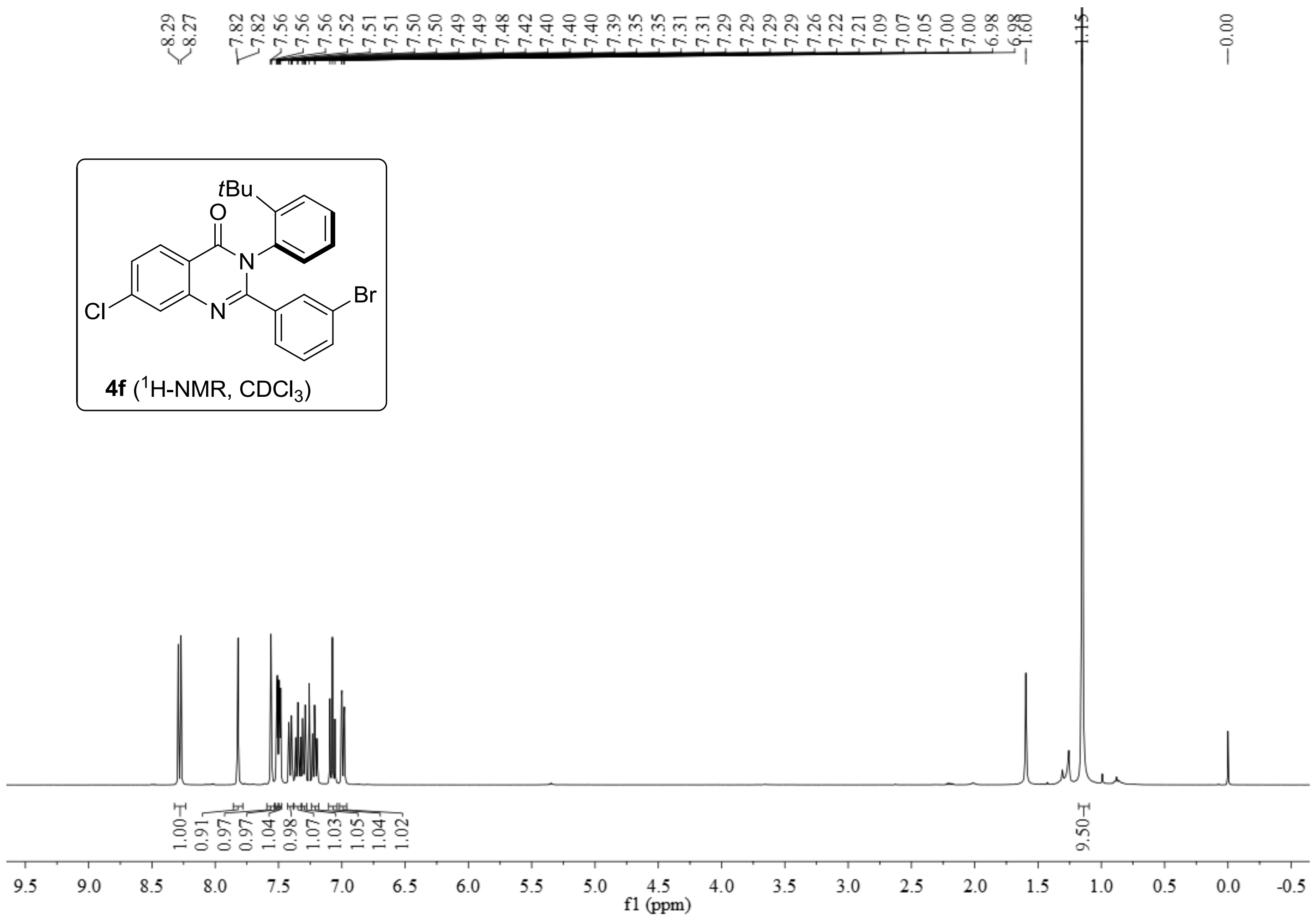


**Supplementary Figure 123.** $^{1}$H NMR of **4f**

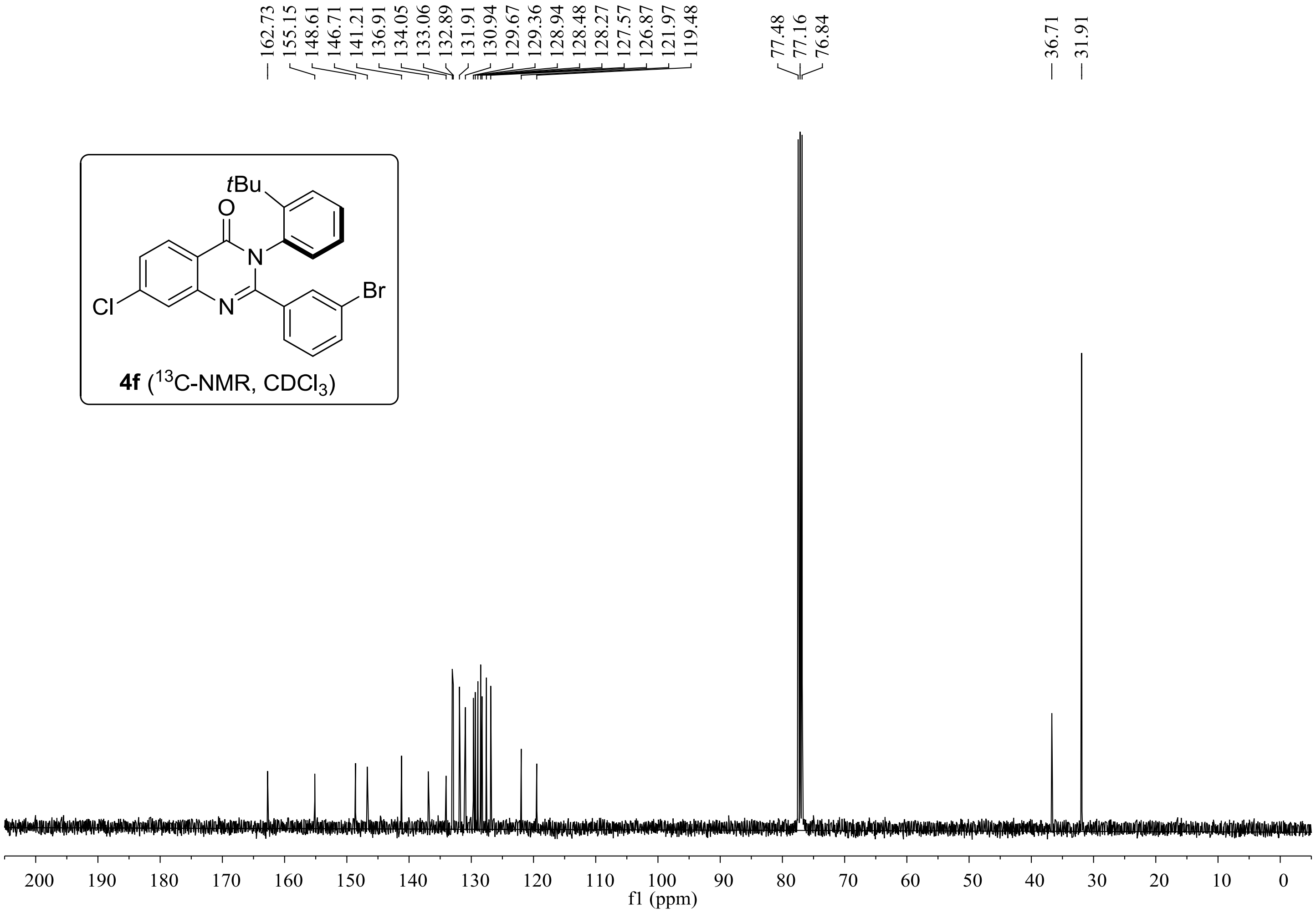


**Supplementary Figure 124.** $^{13}$C NMR of **4f**

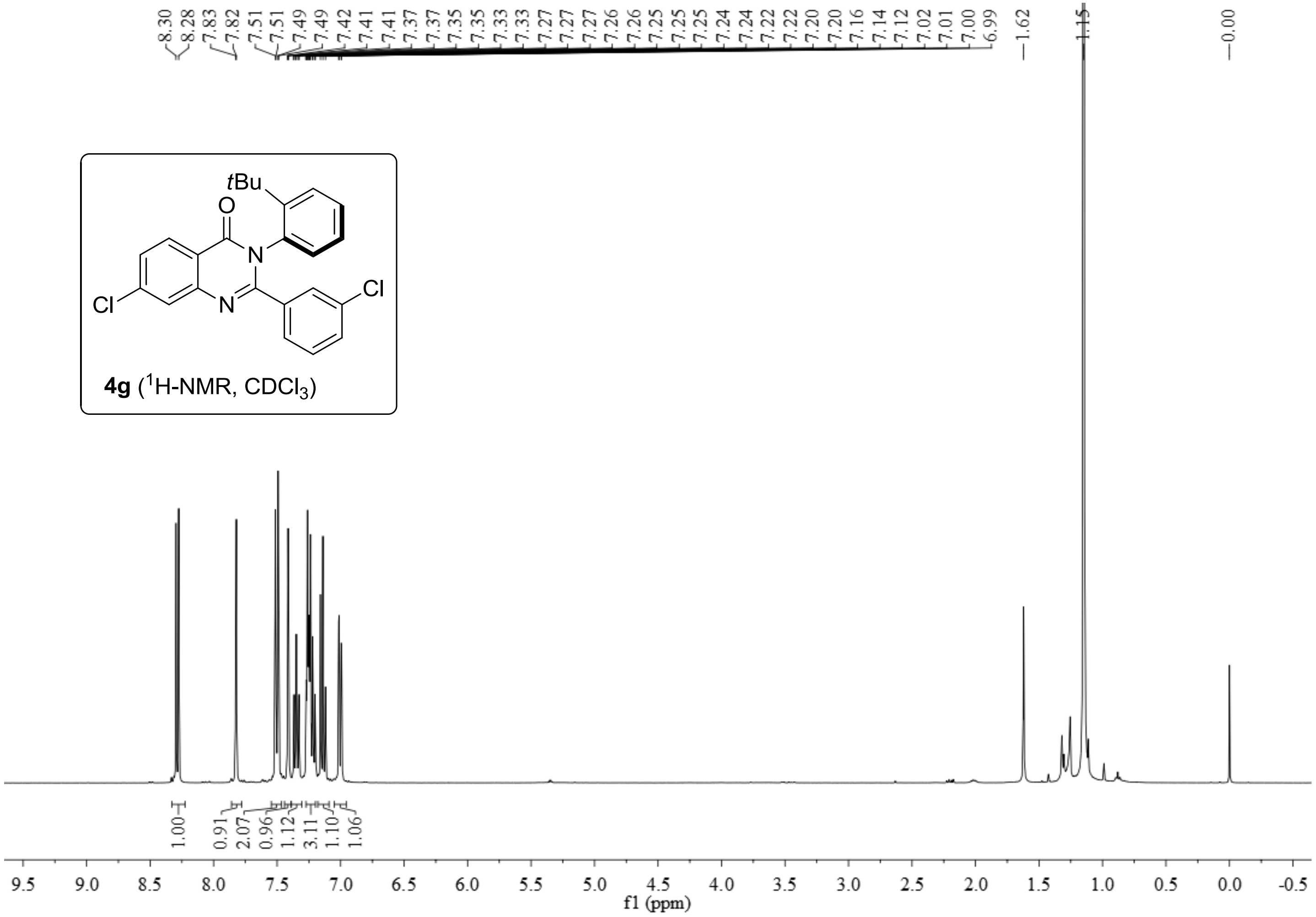


**Supplementary Figure 125.** $^{1}$H NMR of **4g**

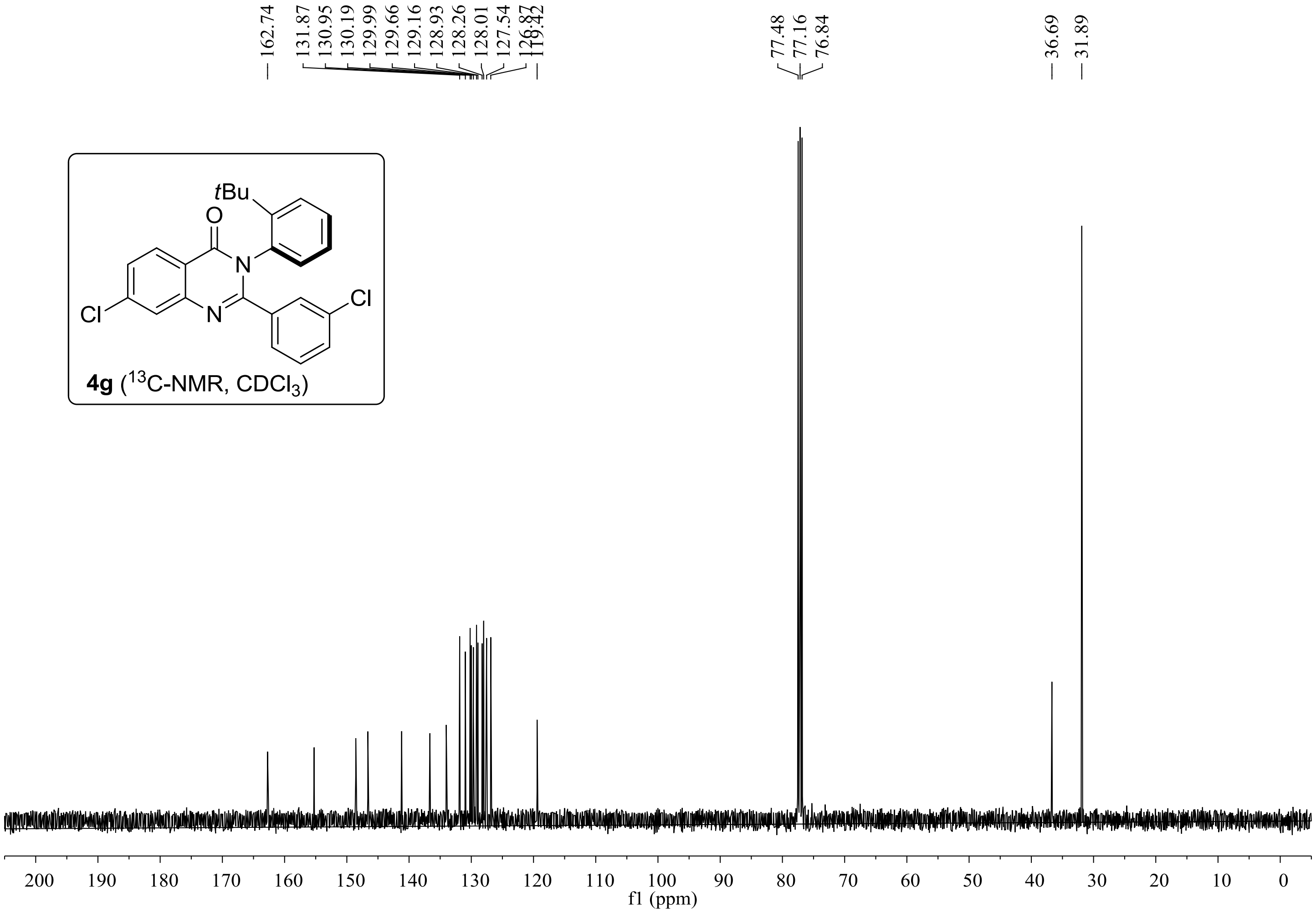


**Supplementary Figure 126.** $^{13}$C NMR of **4g**

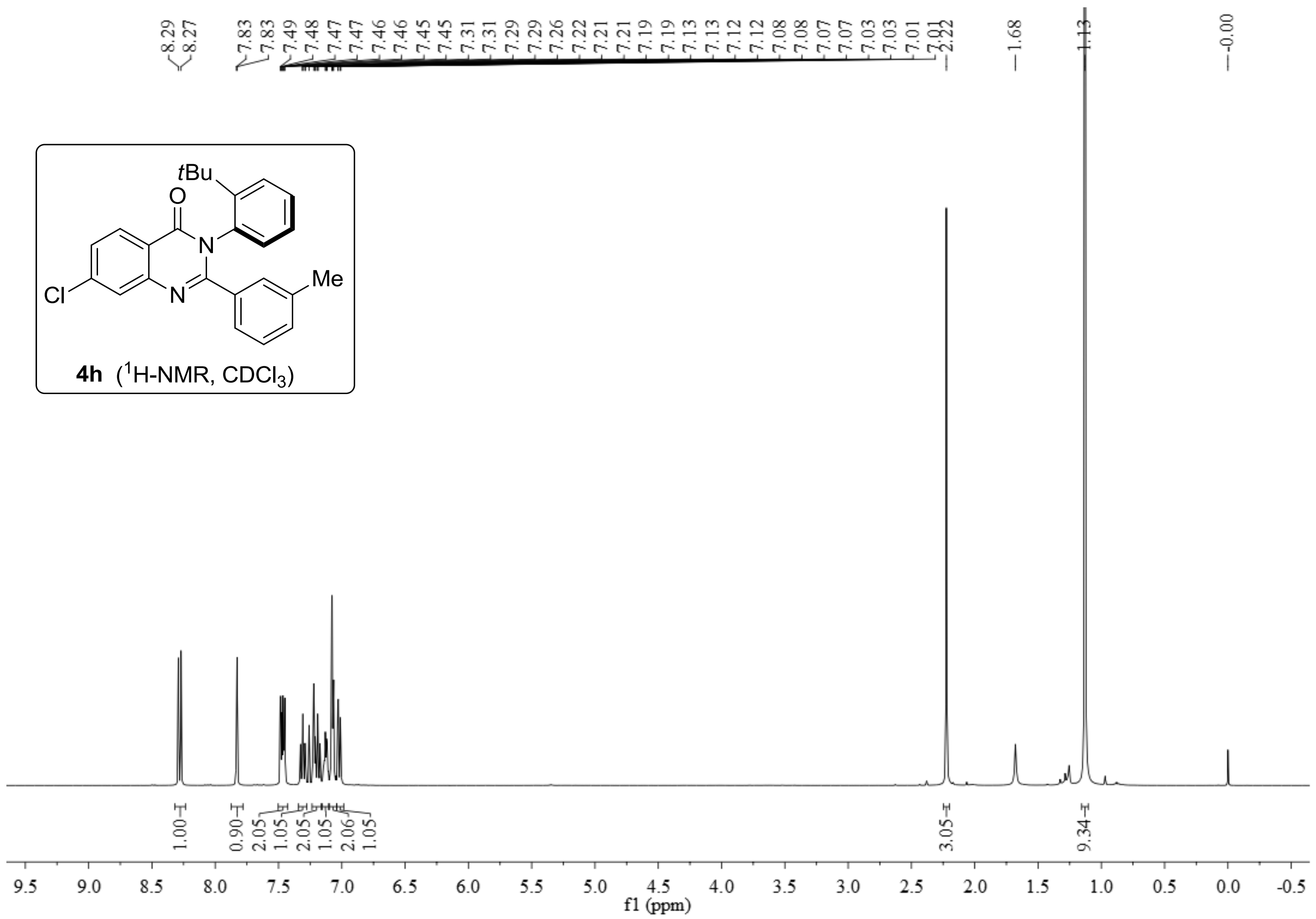


**Supplementary Figure 127.** $^{1}$H NMR of **4h**

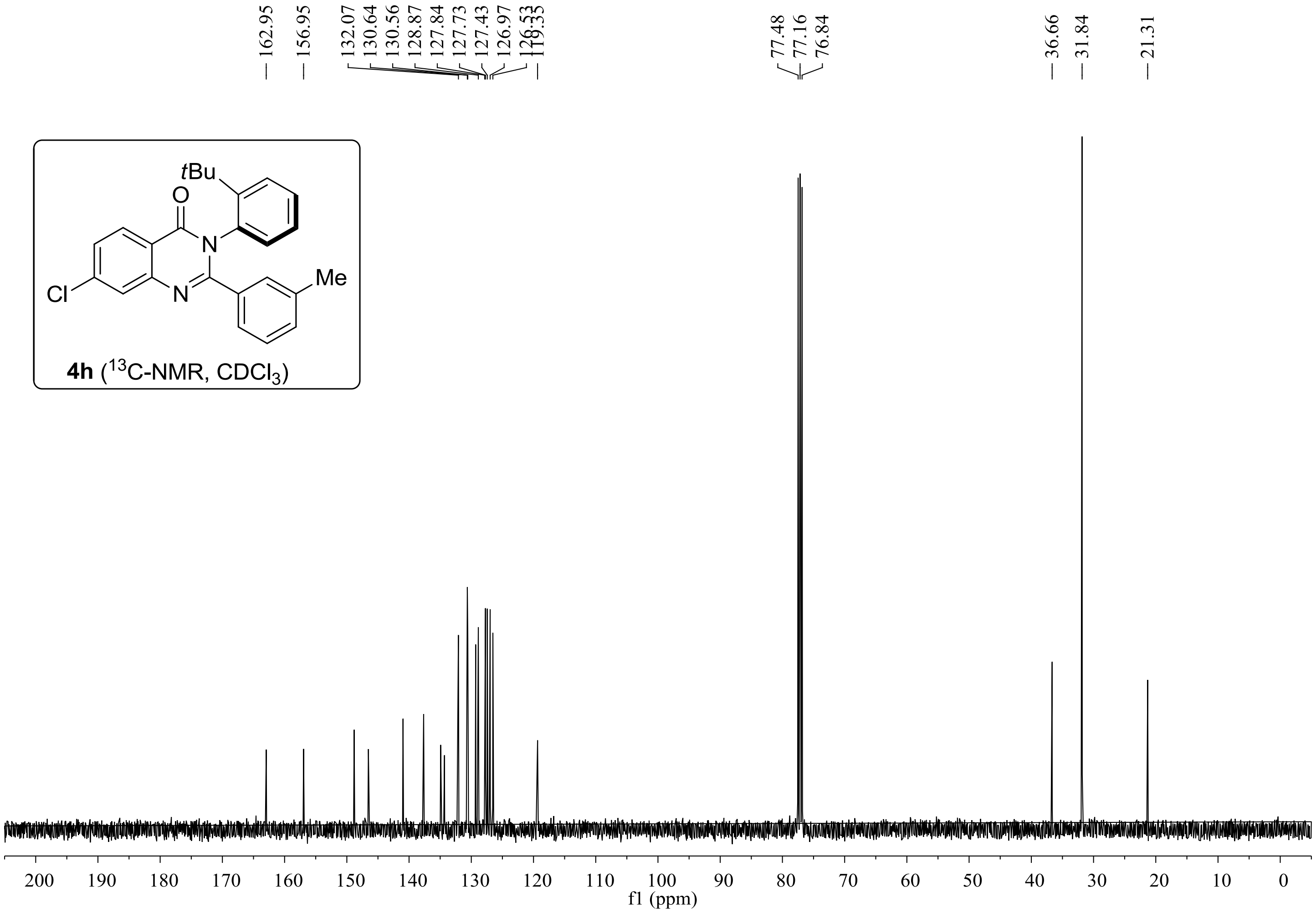


**Supplementary Figure 128.** $^{13}$C NMR of **4h**

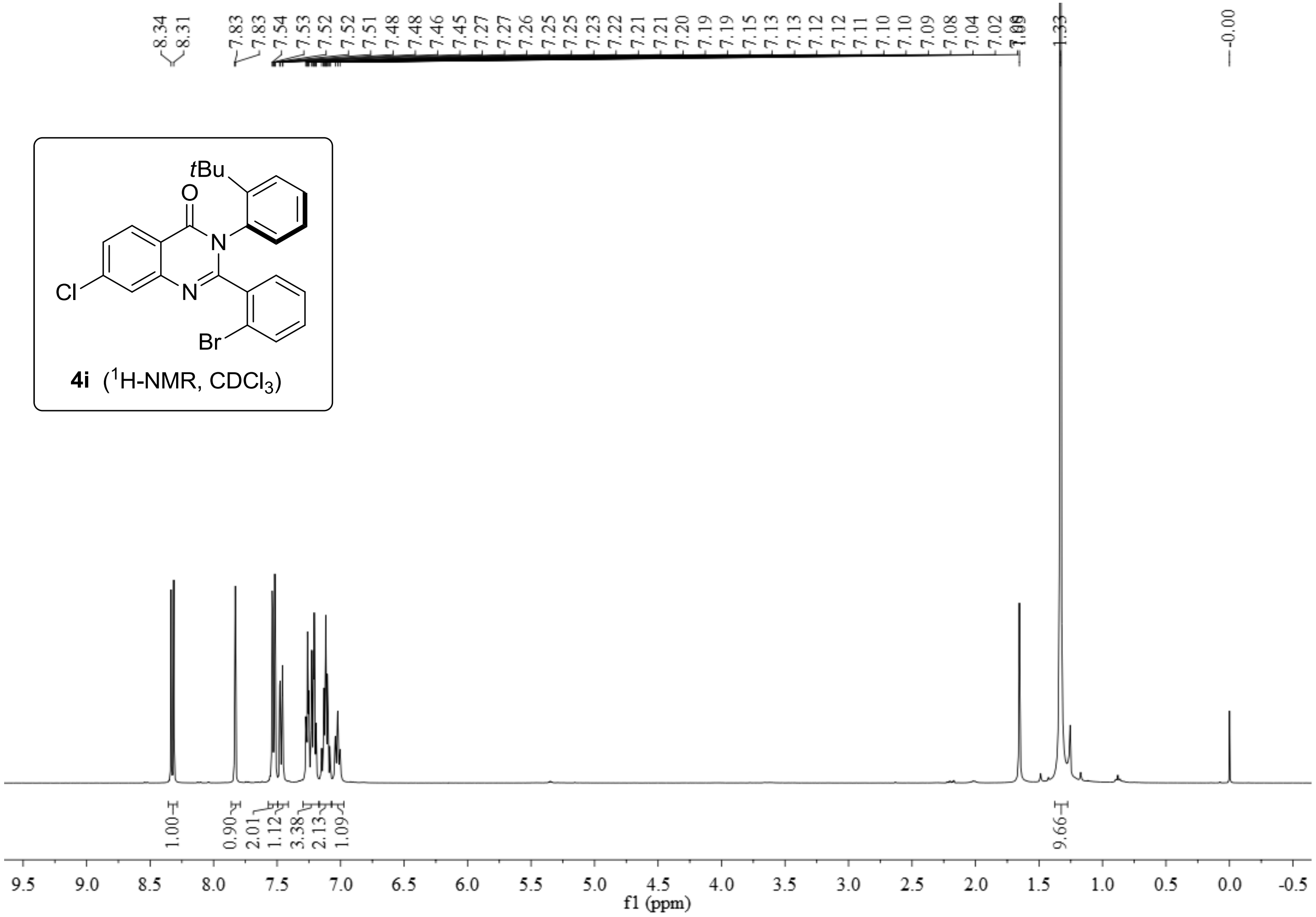


**Supplementary Figure 129.** $^1$H NMR of **4i**

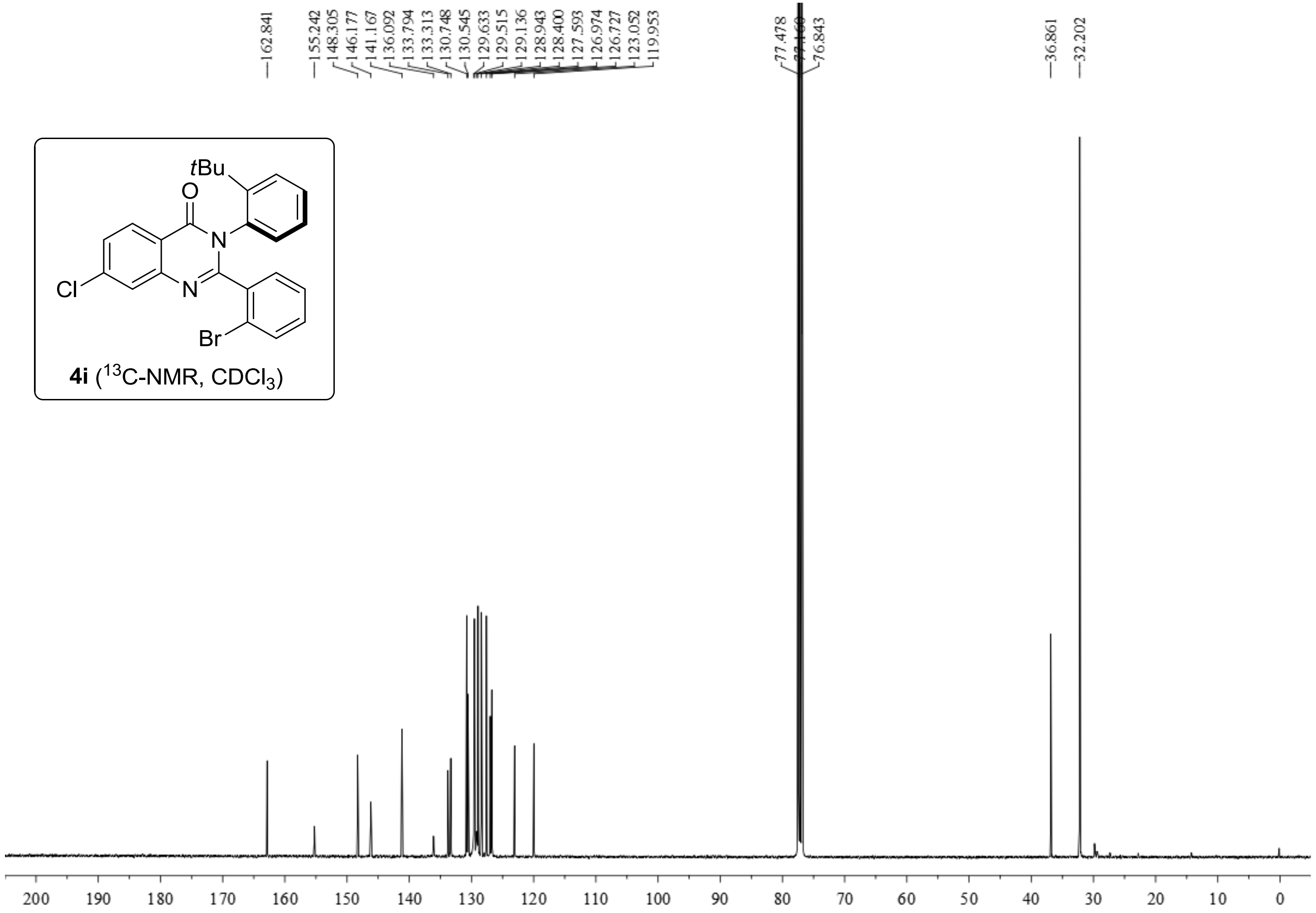


**Supplementary Figure 130.** $^{13}$C NMR of **4i**

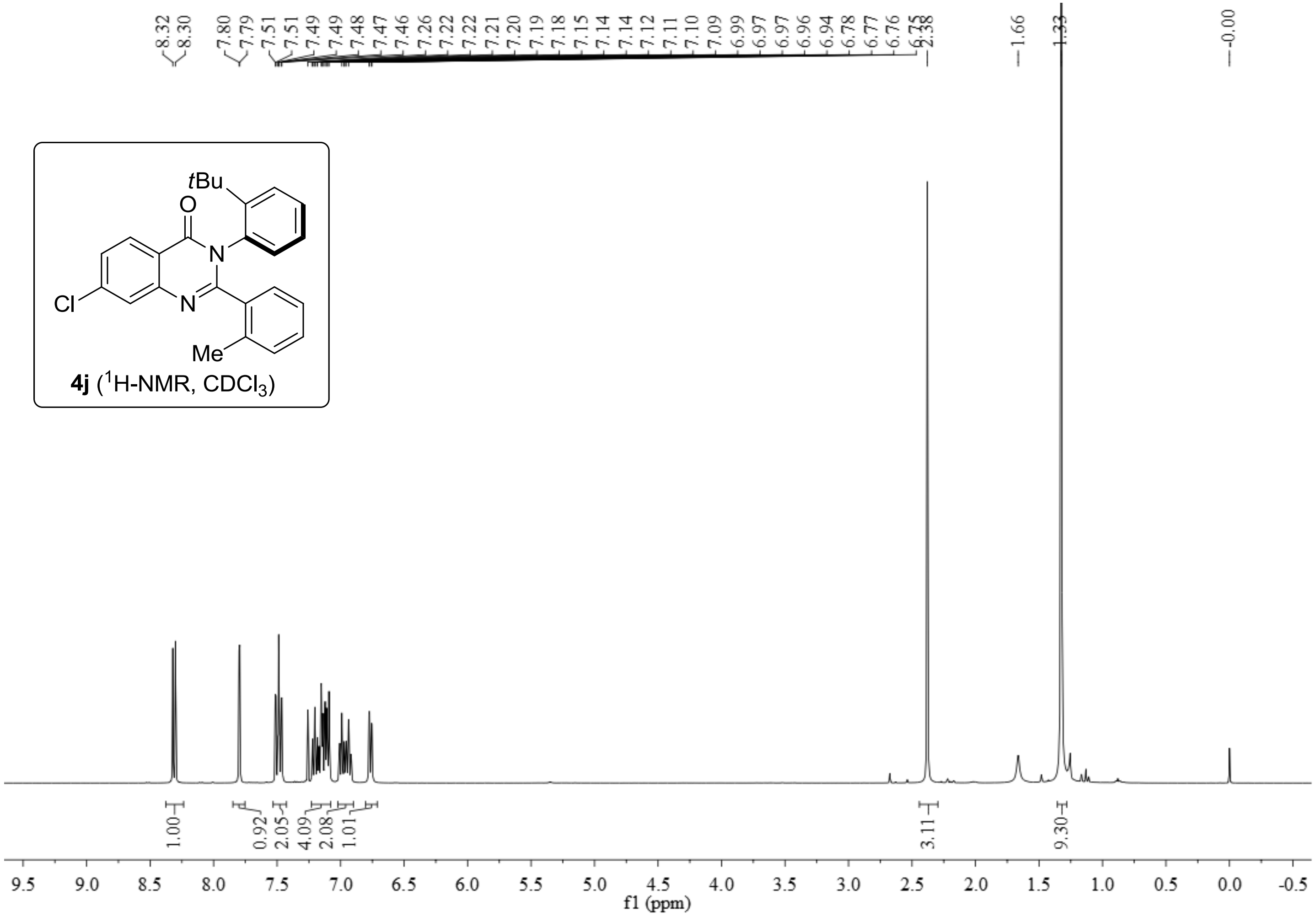


**Supplementary Figure 131.** $^{1}$H NMR of **4j**

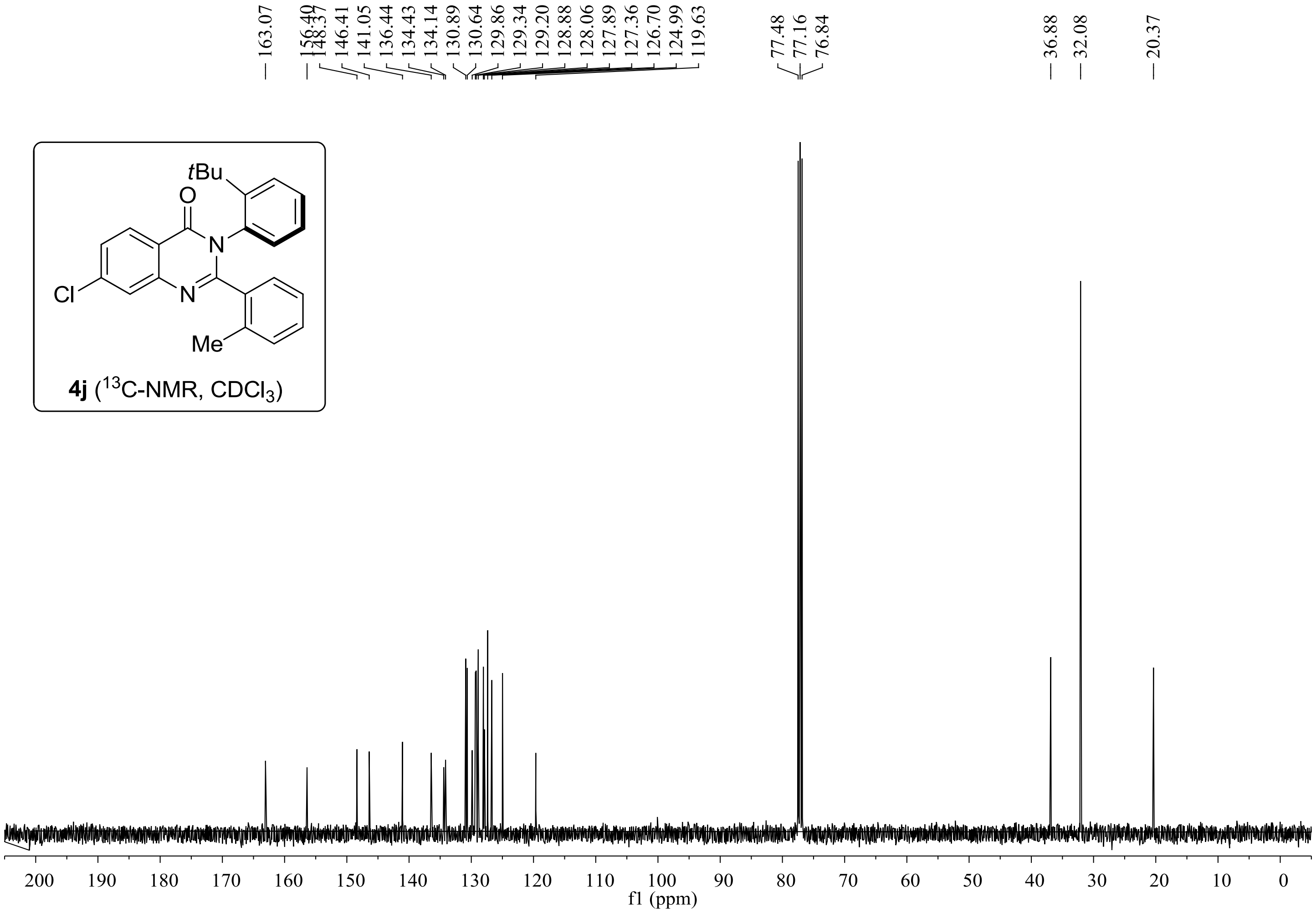


**Supplementary Figure 132.** $^{13}$C NMR of **4j**

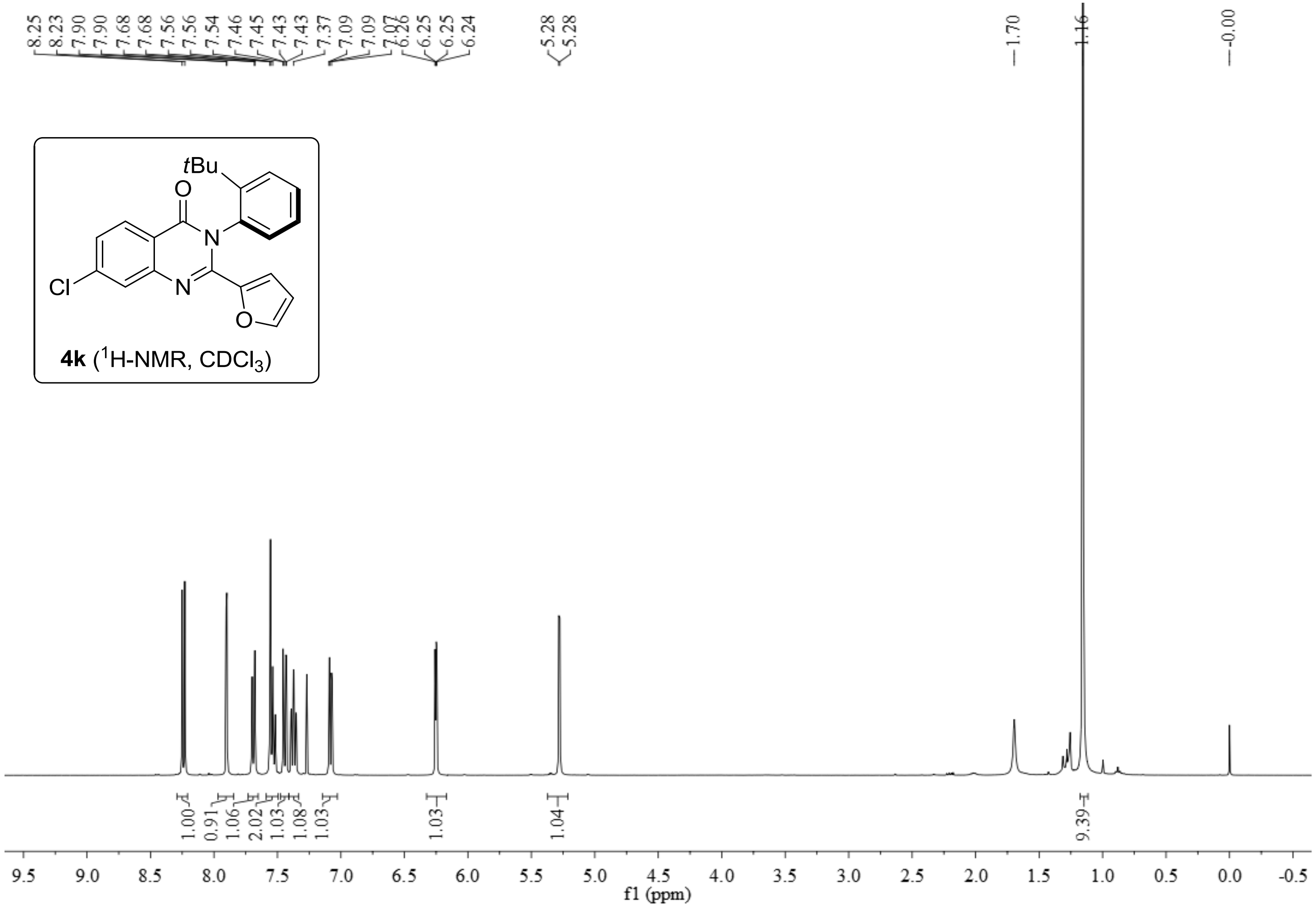


**Supplementary Figure 133.** $^1$H NMR of **4k**

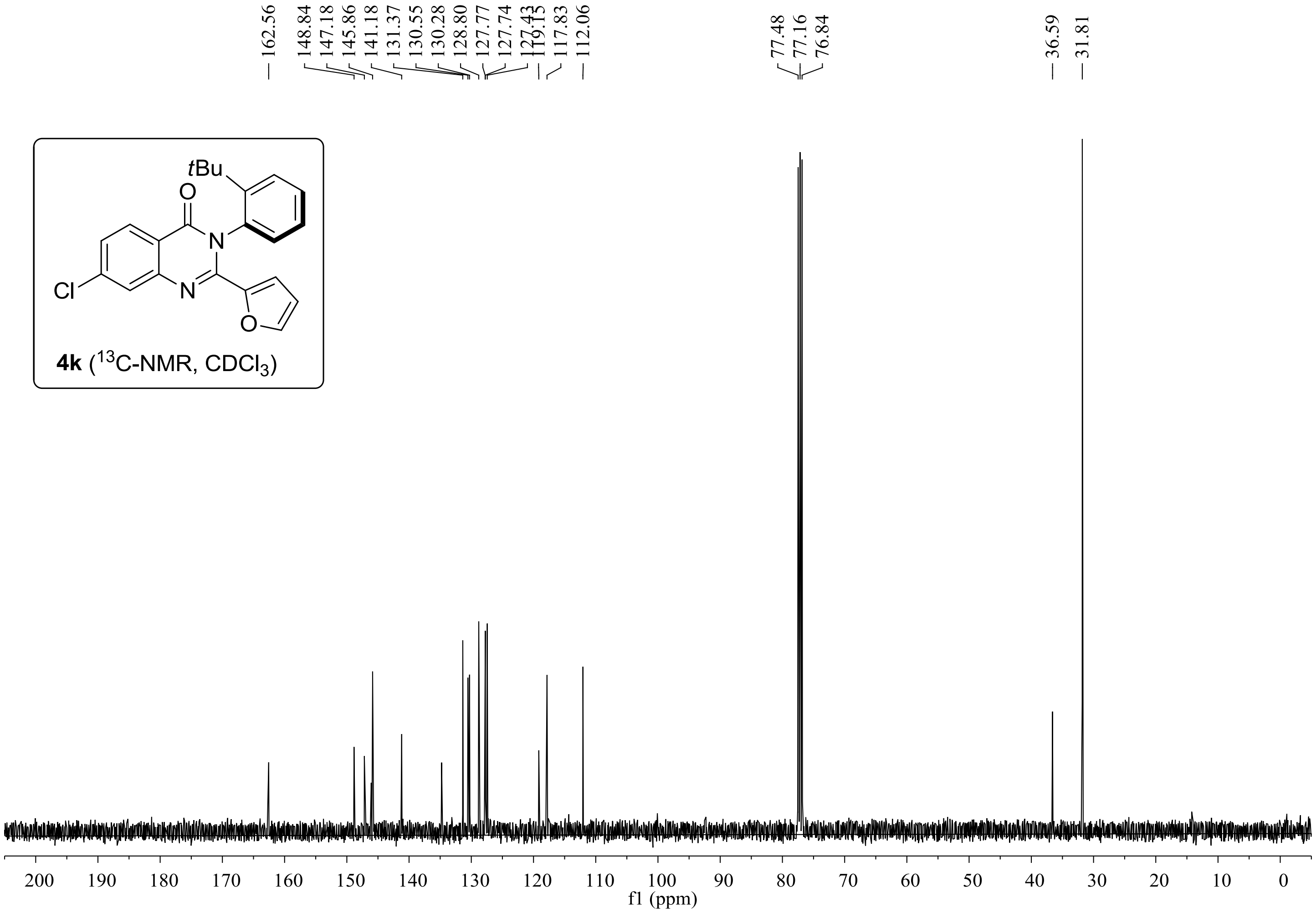


**Supplementary Figure 134.** $^{13}$C NMR of **4k**

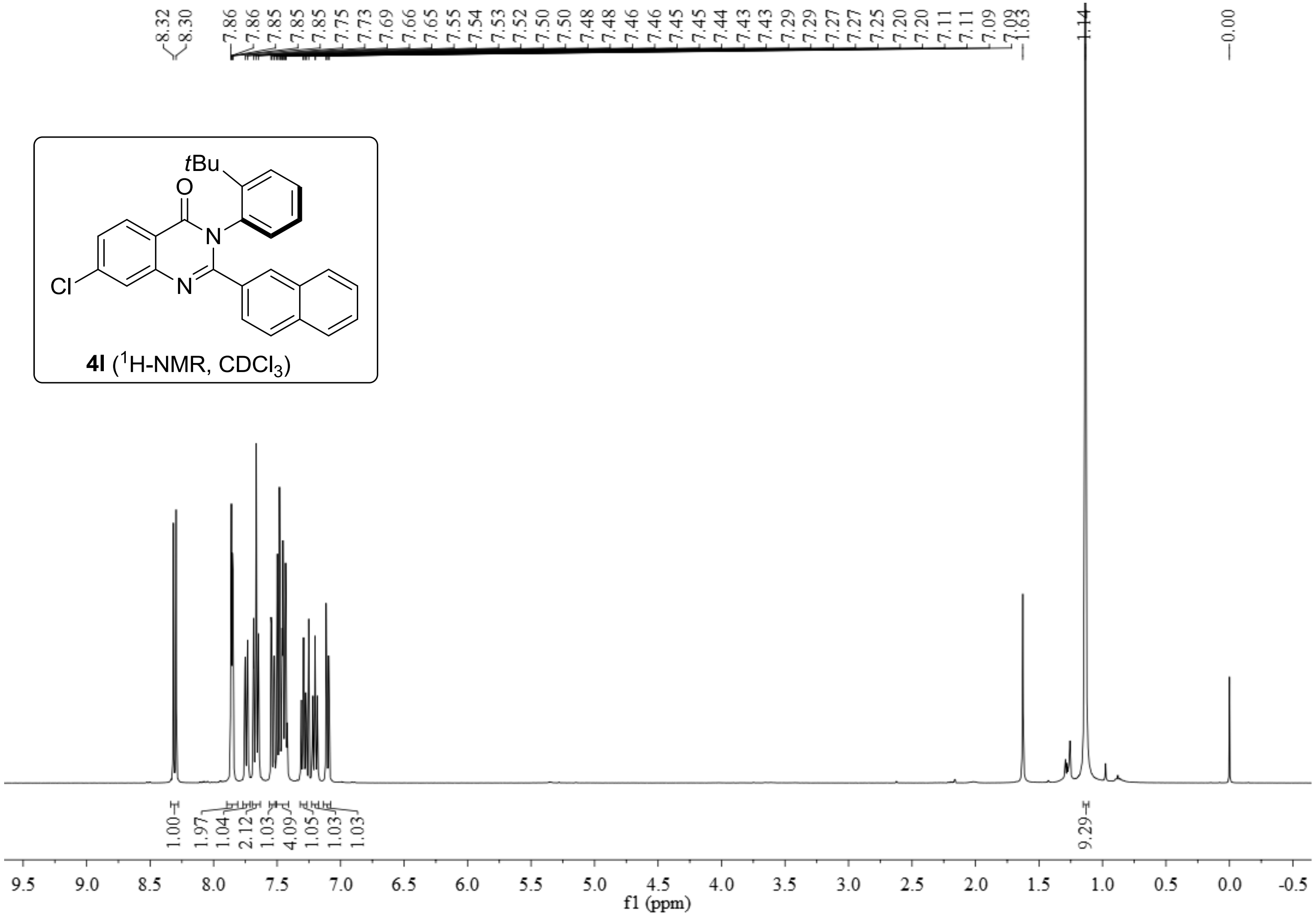


**Supplementary Figure 135.** $^{1}H$ NMR of **4l**

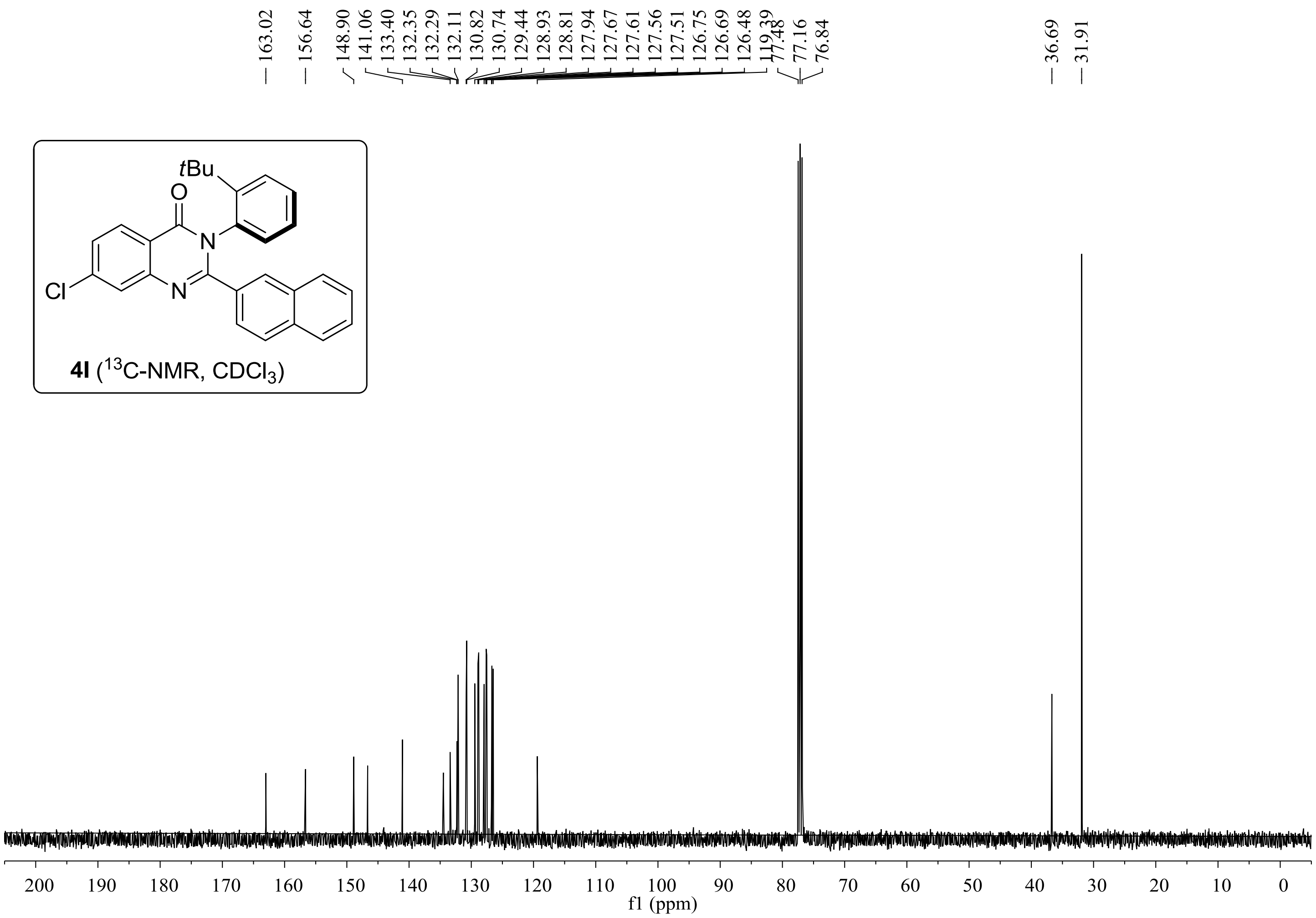


**Supplementary Figure 136.** $^{13}$C NMR of **4l**

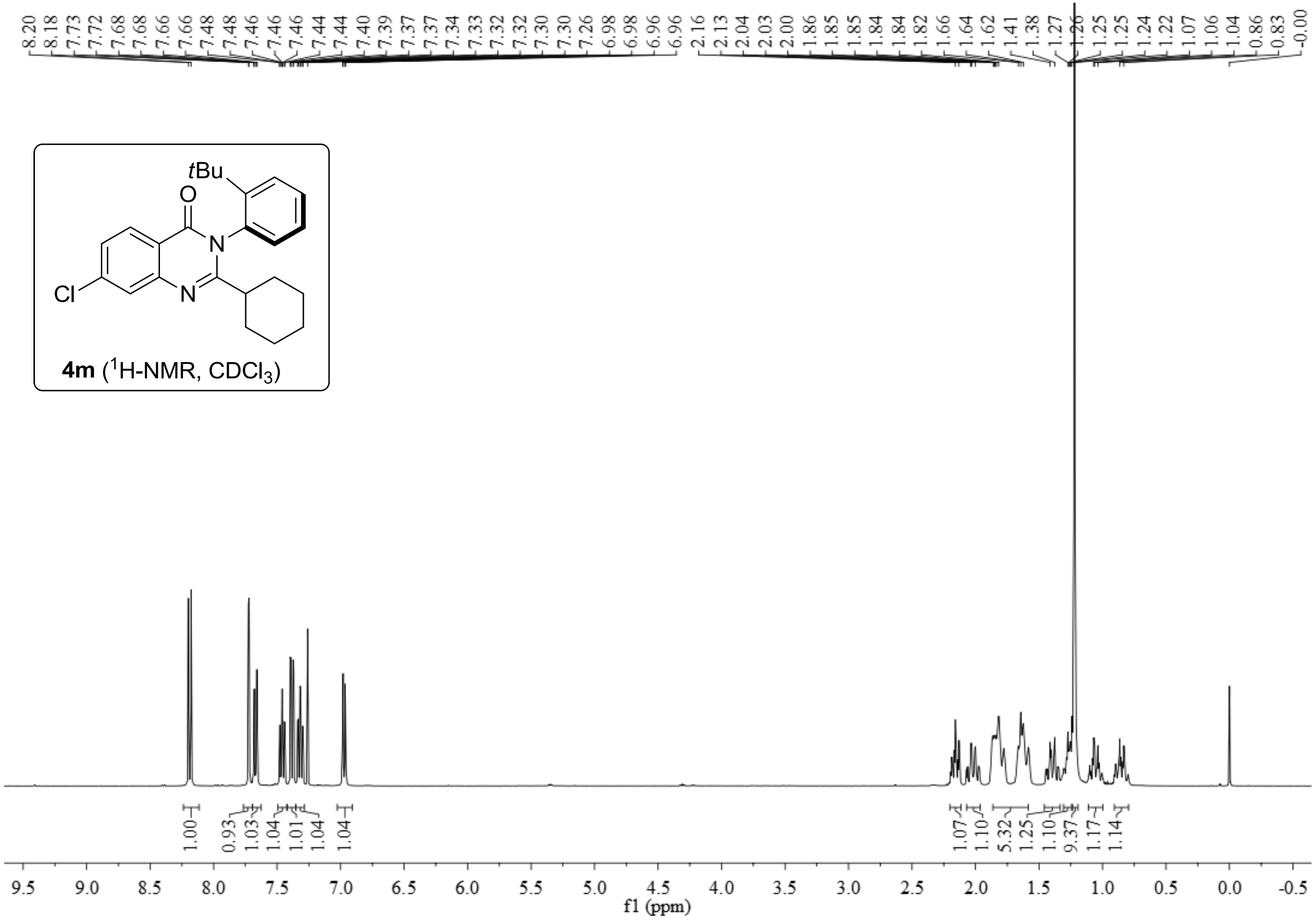


**Supplementary Figure 137.** $^{1}$H NMR of **4m**

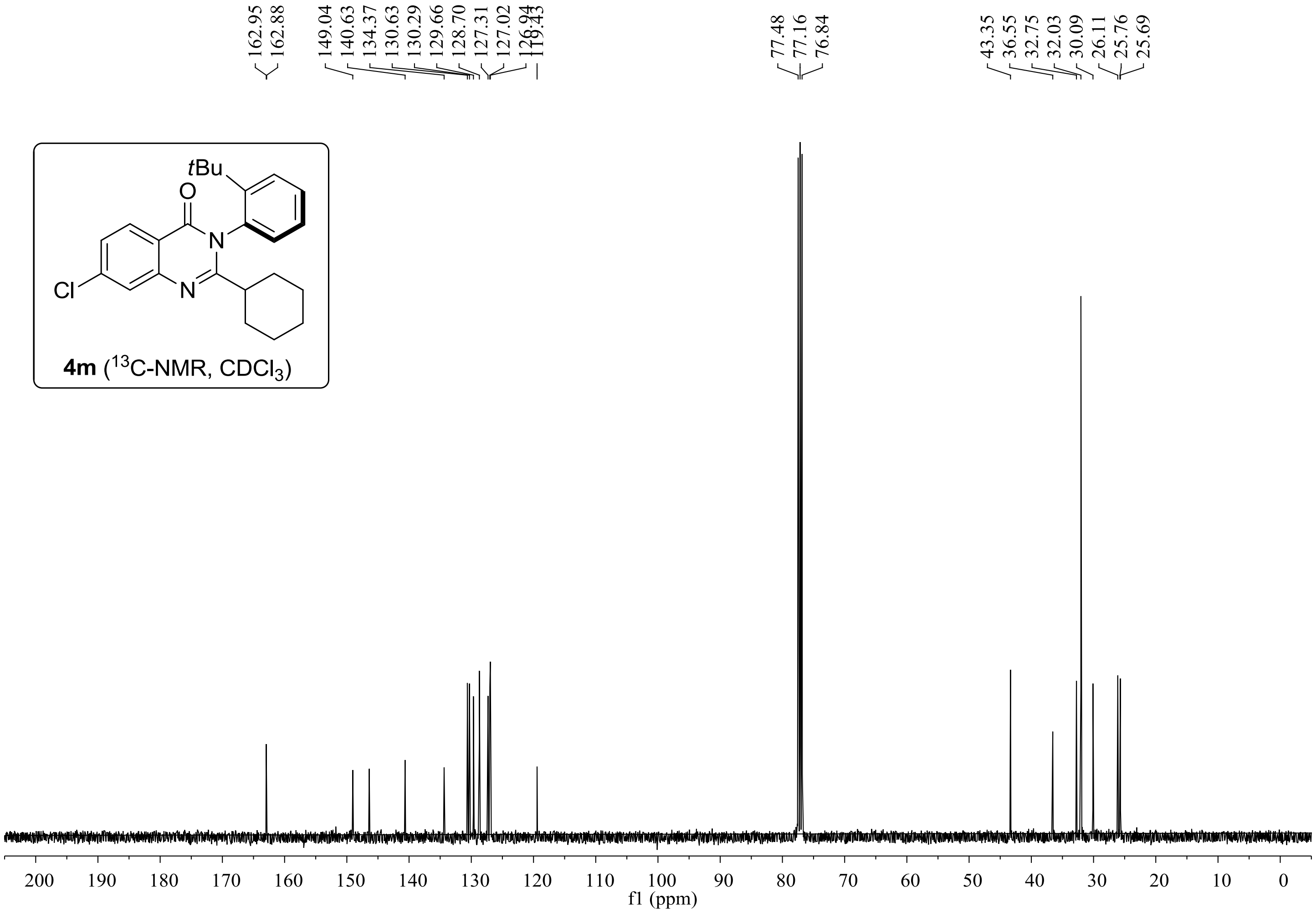


**Supplementary Figure 138.** $^{13}$C NMR of **4m**

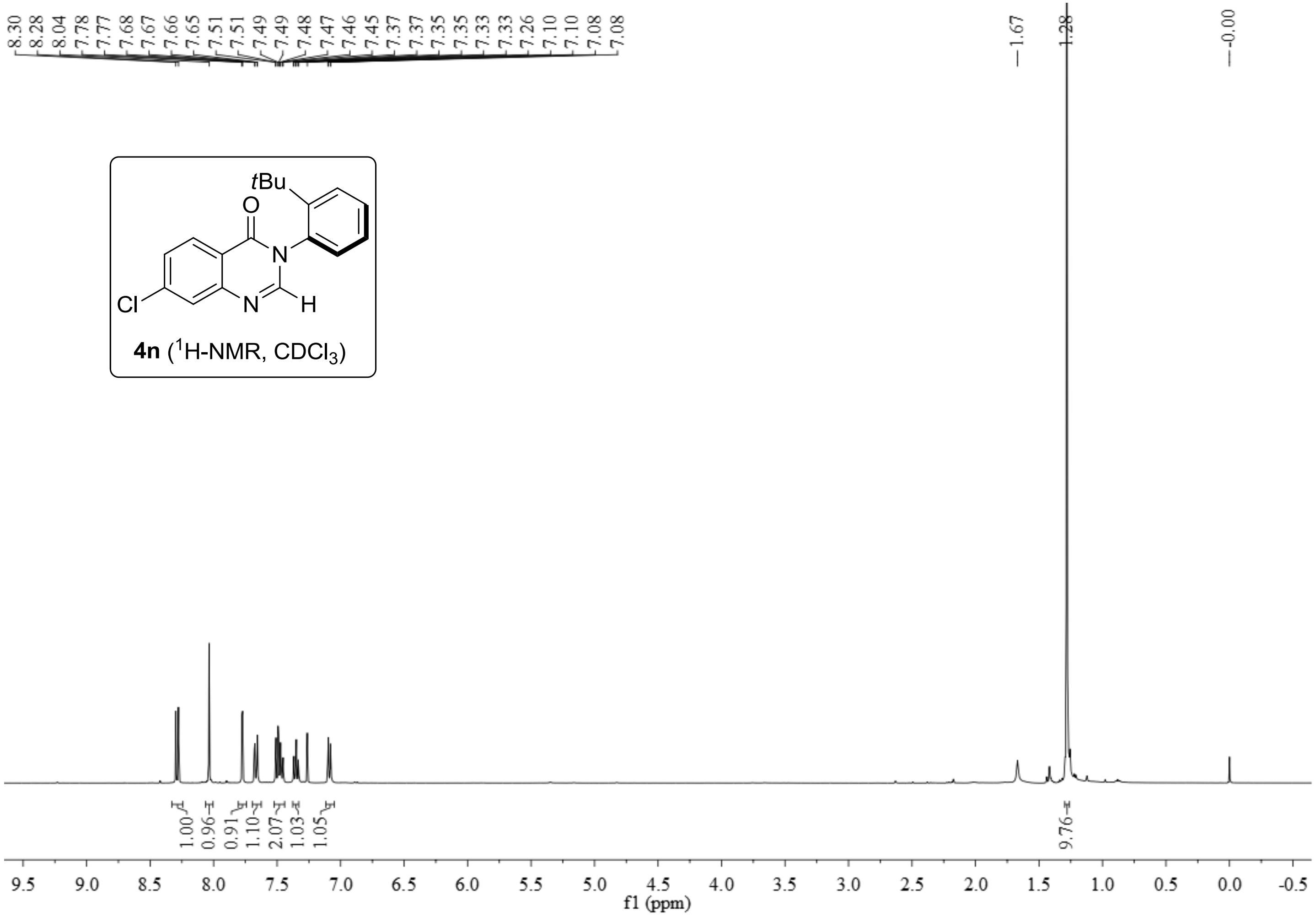


**Supplementary Figure 139.** $^{1}$H NMR of **4n**

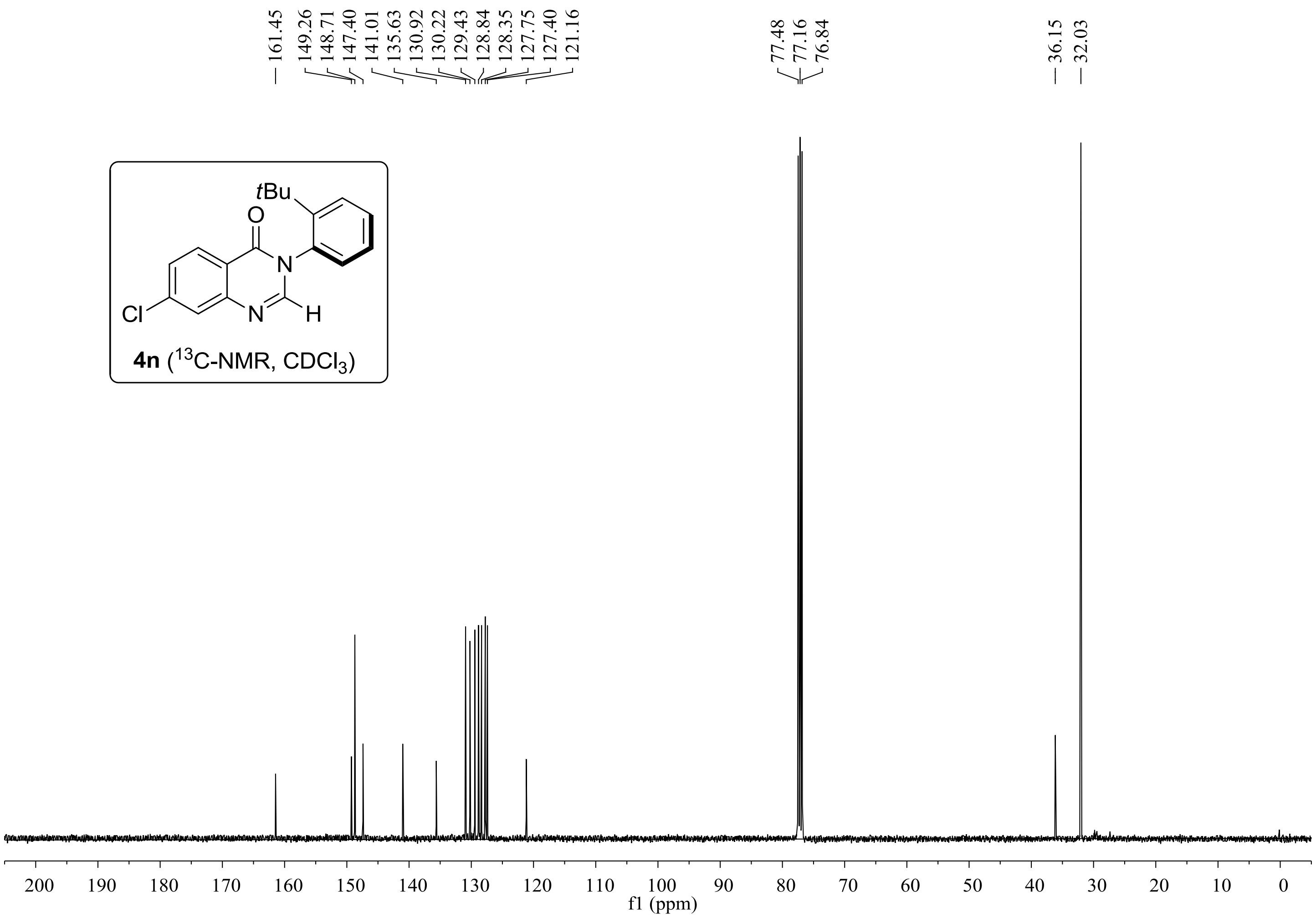


**Supplementary Figure 140.** $^{13}$C NMR of **4n**

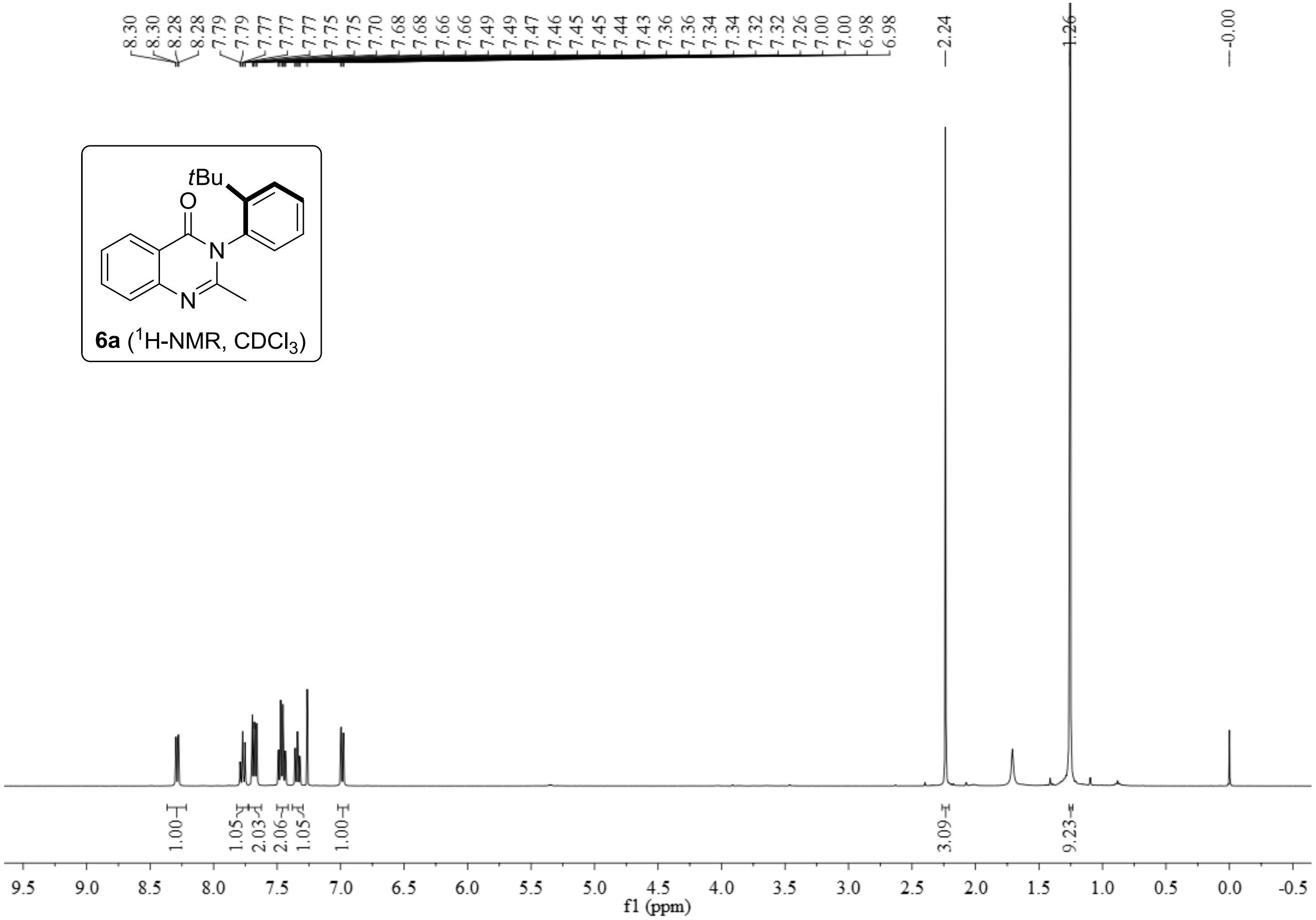


**Supplementary Figure 141.** $^{1}$H NMR of **6a**

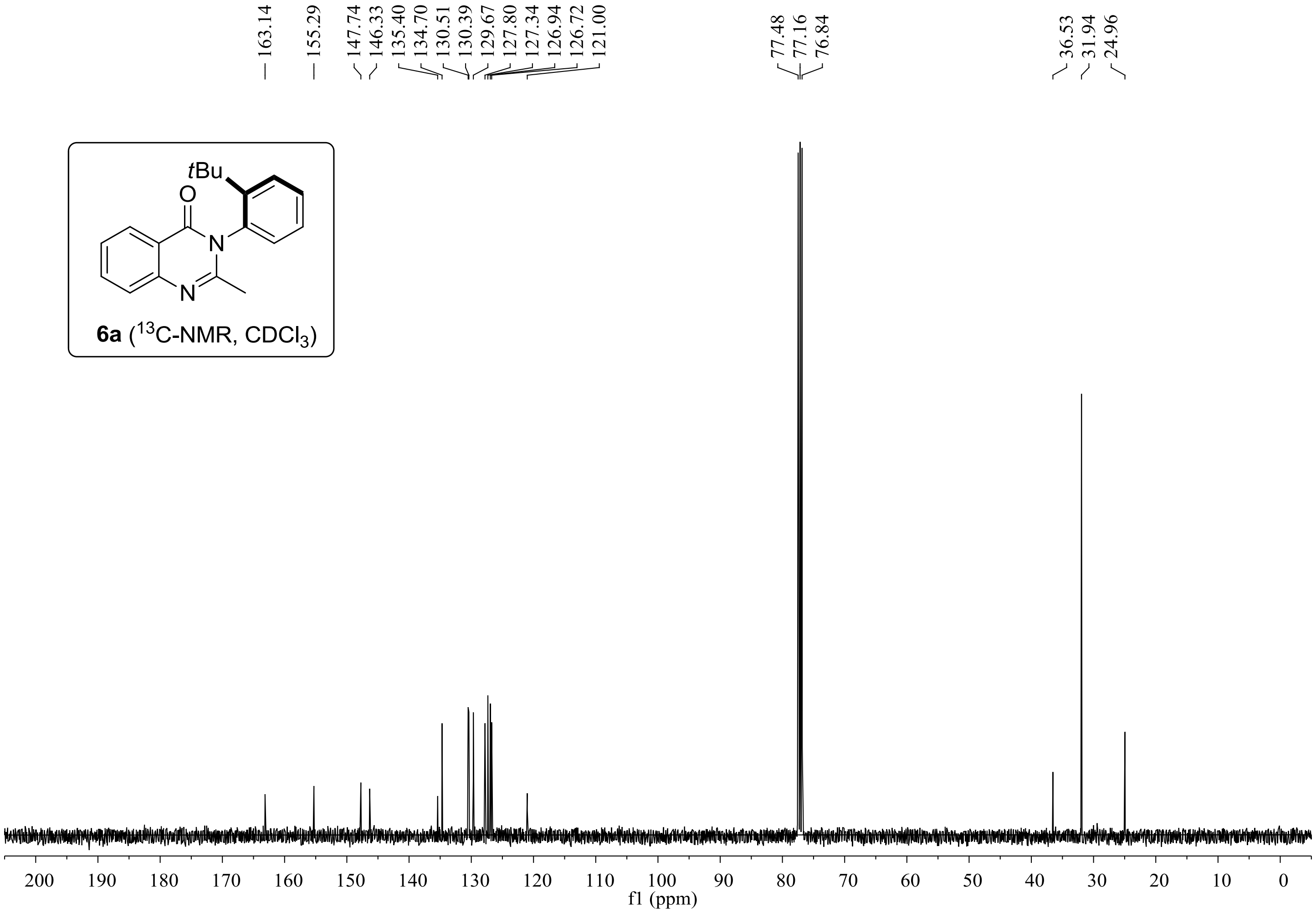


**Supplementary Figure 142.** $^{13}$C NMR of **6a**

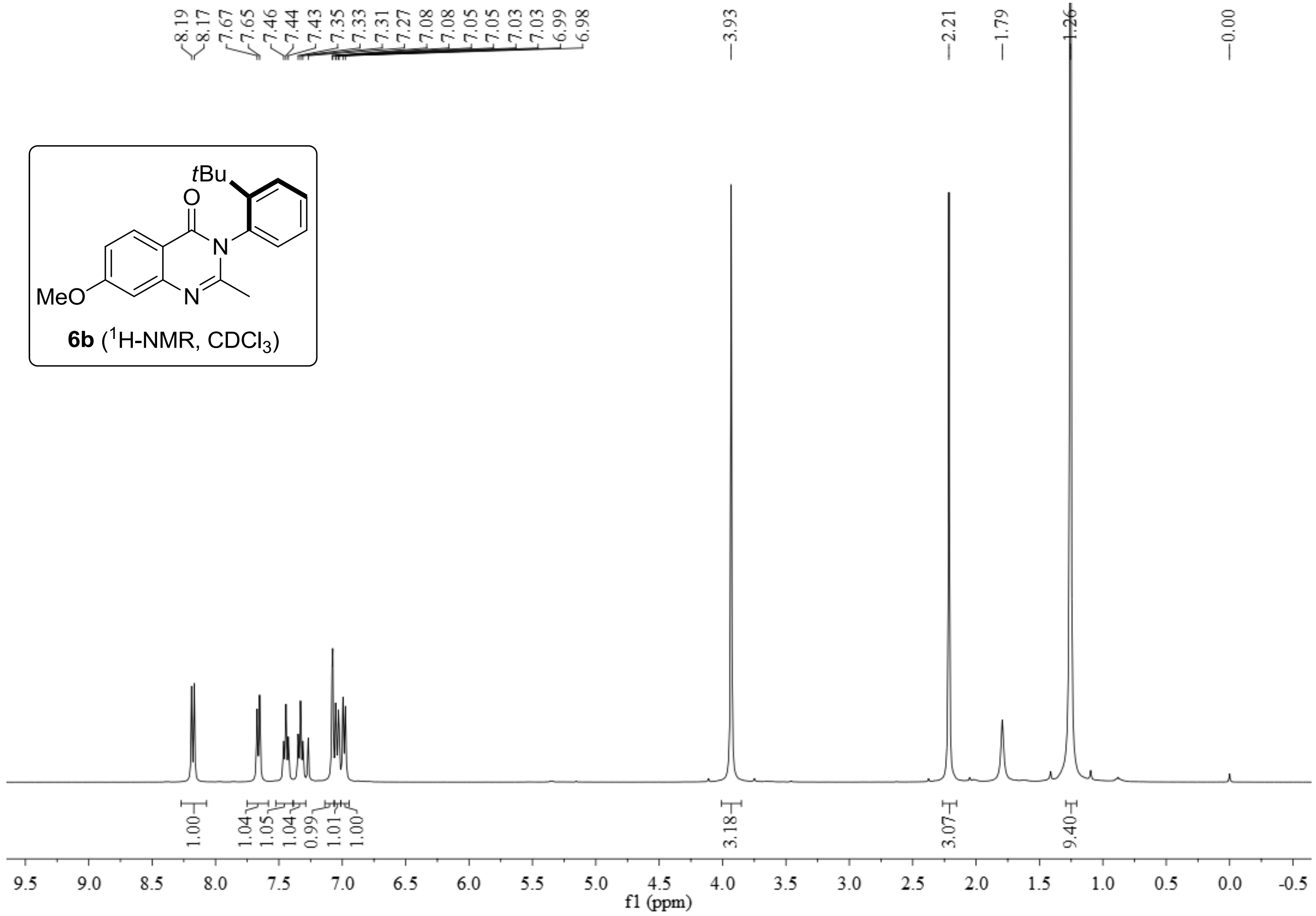


**Supplementary Figure 143.** $^1$H NMR of **6b**

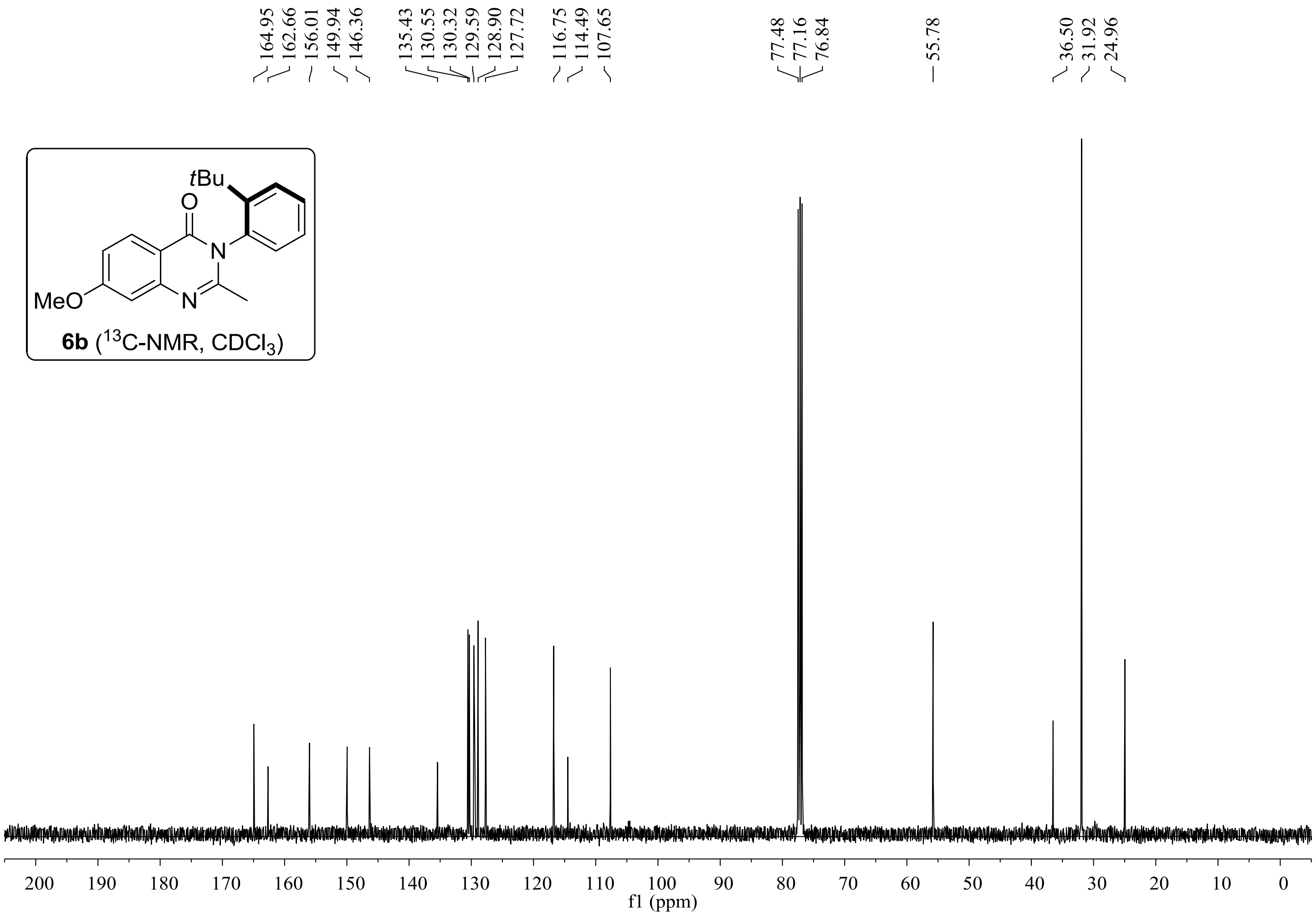


**Supplementary Figure 144.** $^{13}$C NMR of **6b**

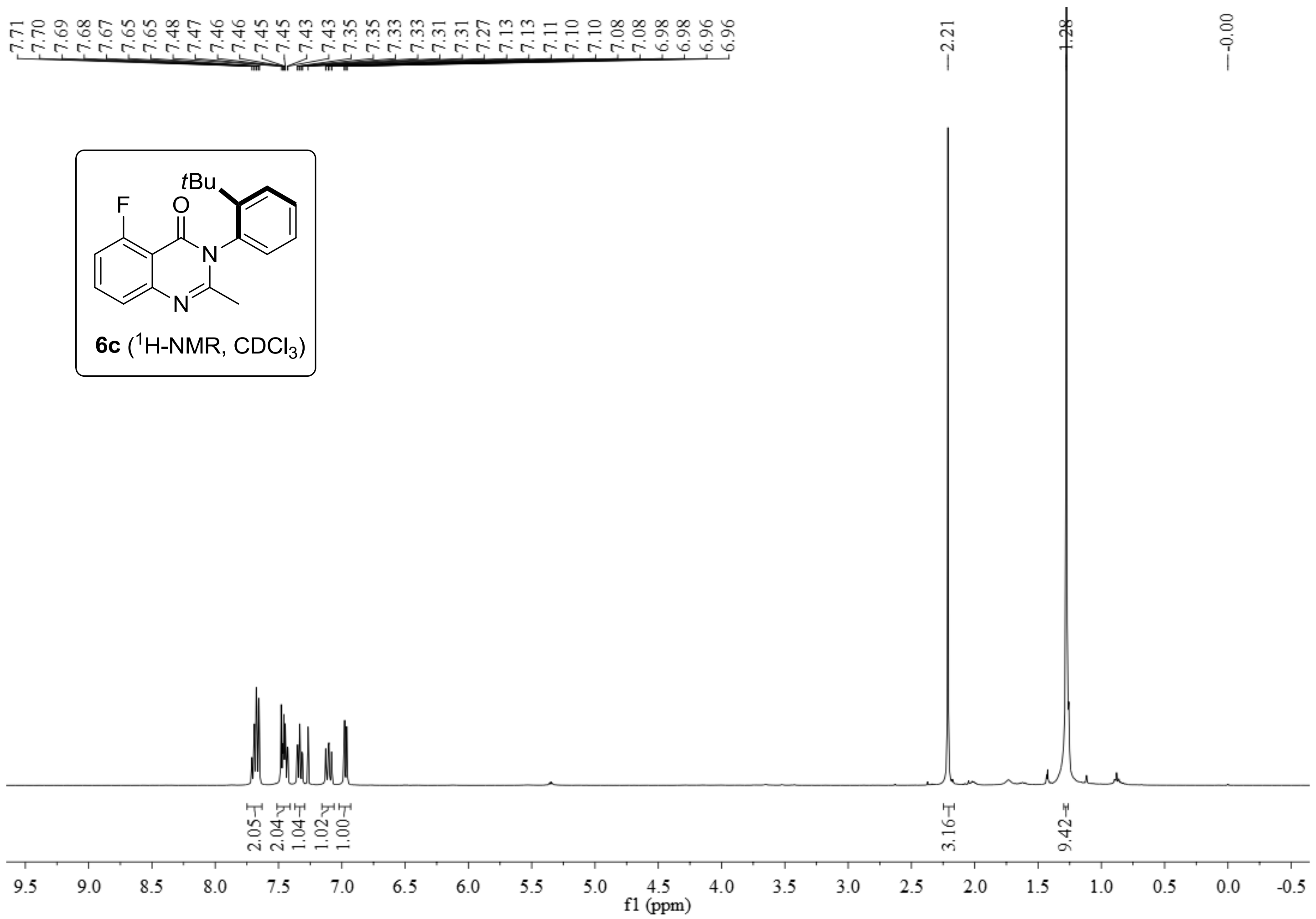


**Supplementary Figure 145.** $^{1}$H NMR of **6c**

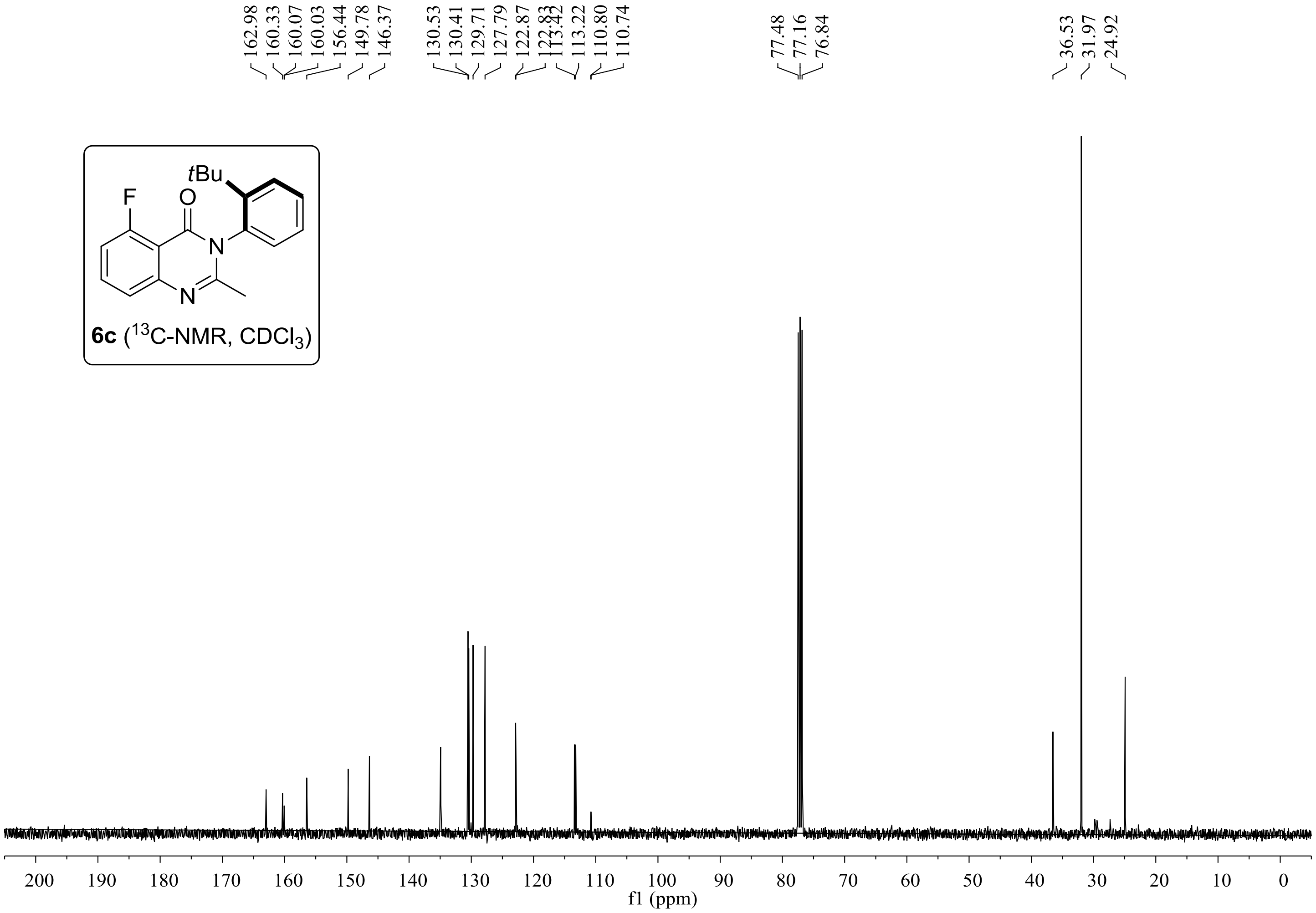


**Supplementary Figure 146.** $^{13}$C NMR of **6c**

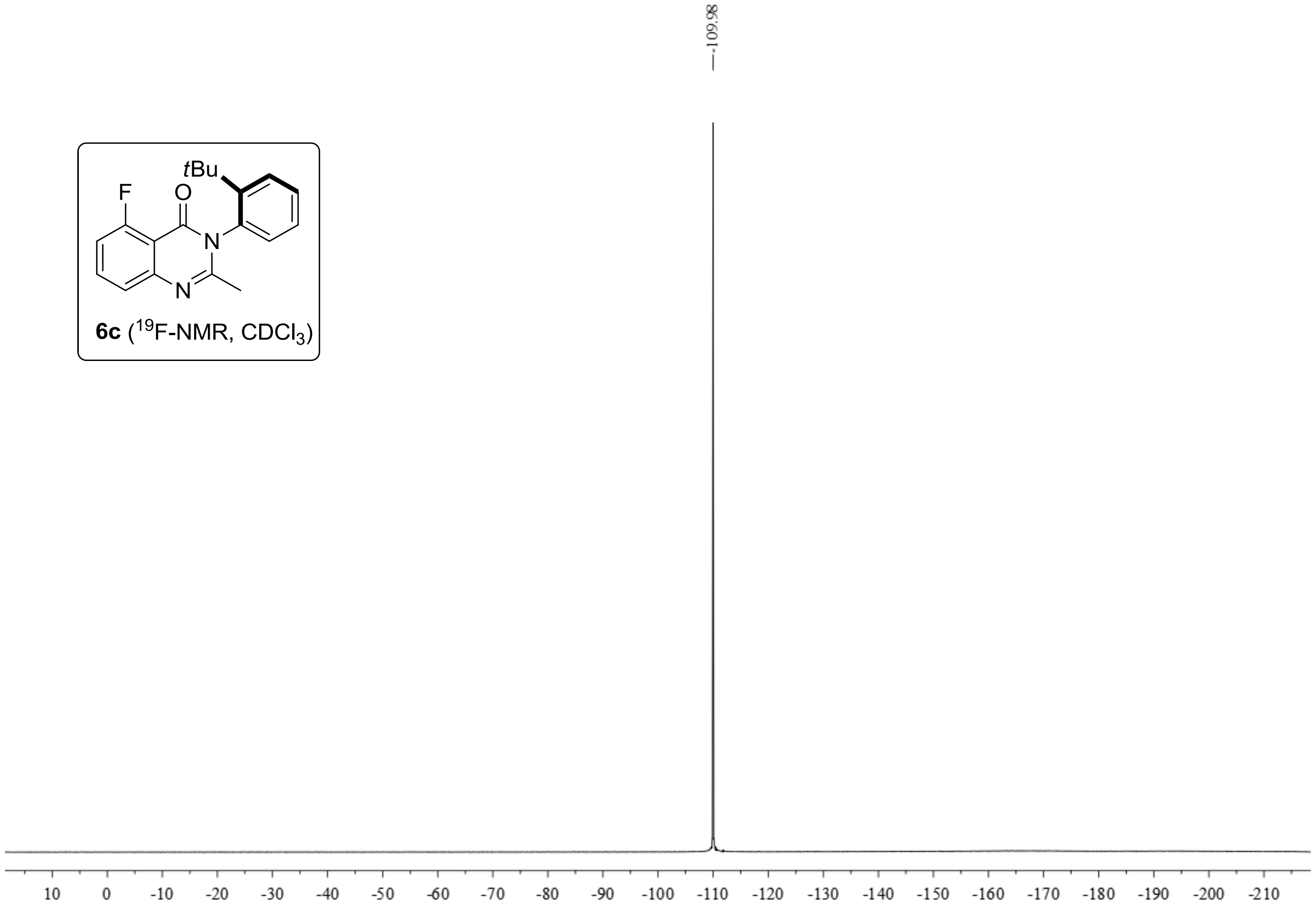


**Supplementary Figure 147.** $^{19}$F NMR of **6c**

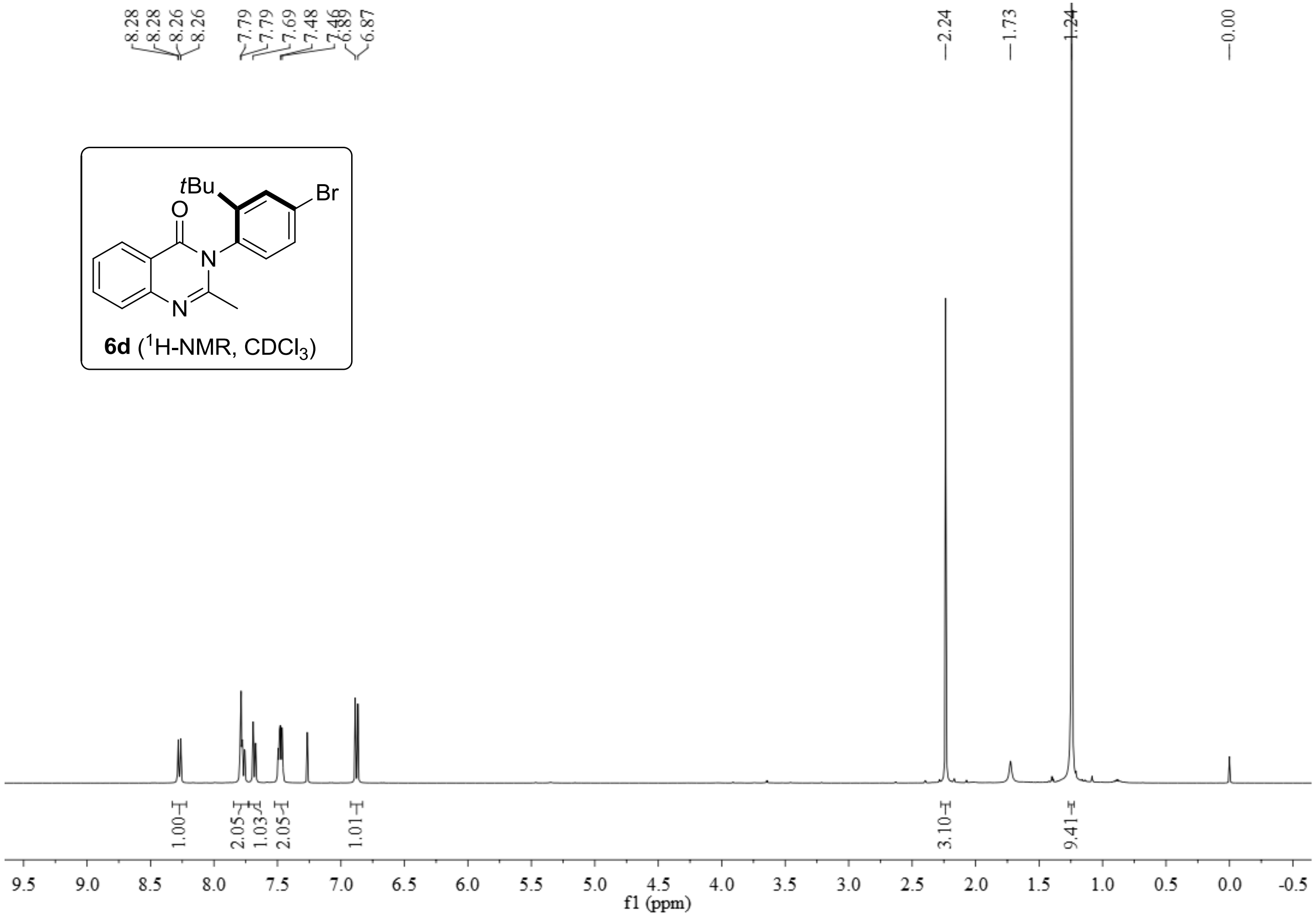


**Supplementary Figure 148.** $^{1}$H NMR of **6d**

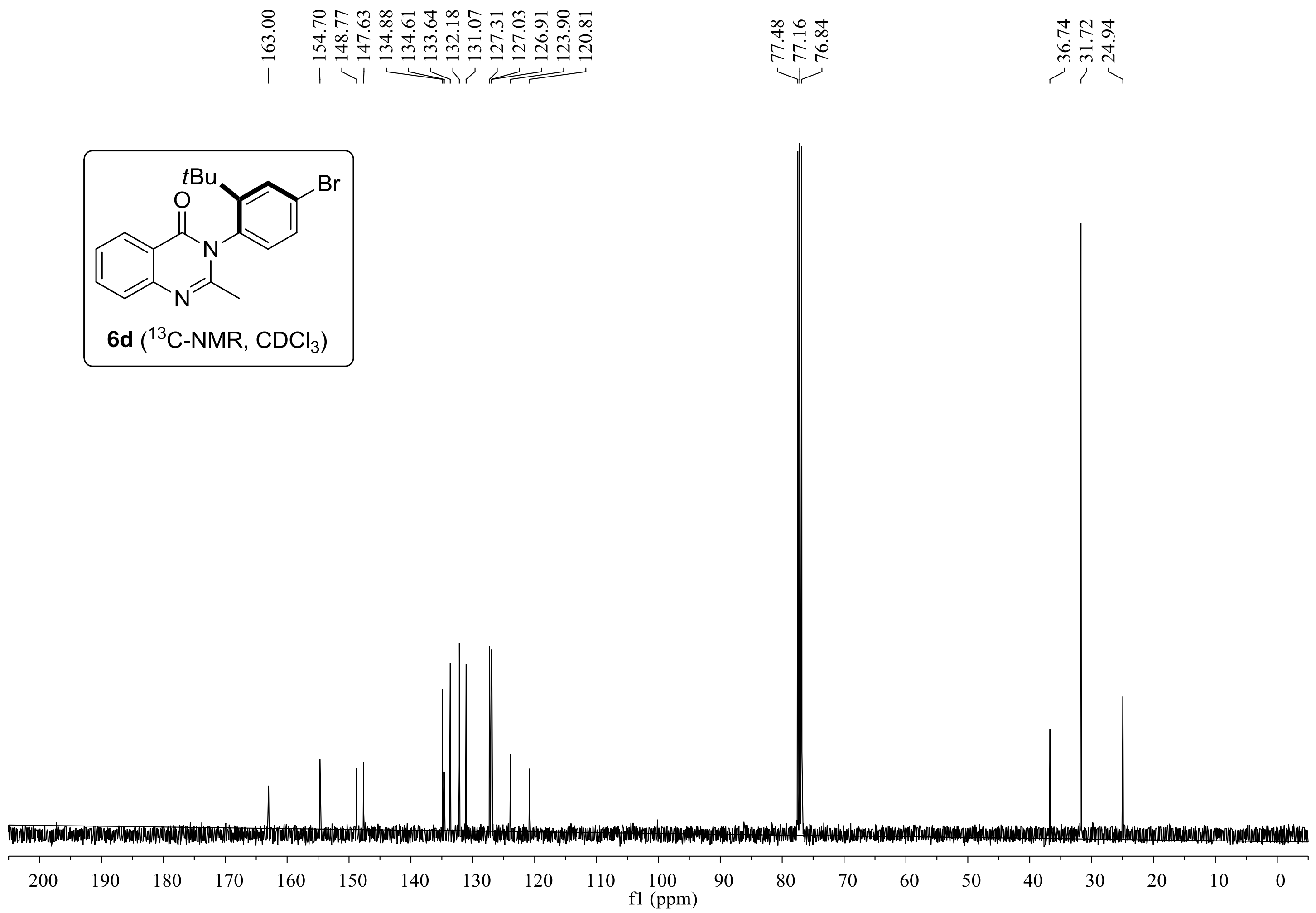


**Supplementary Figure 149.** $^{13}$C NMR of **6d**

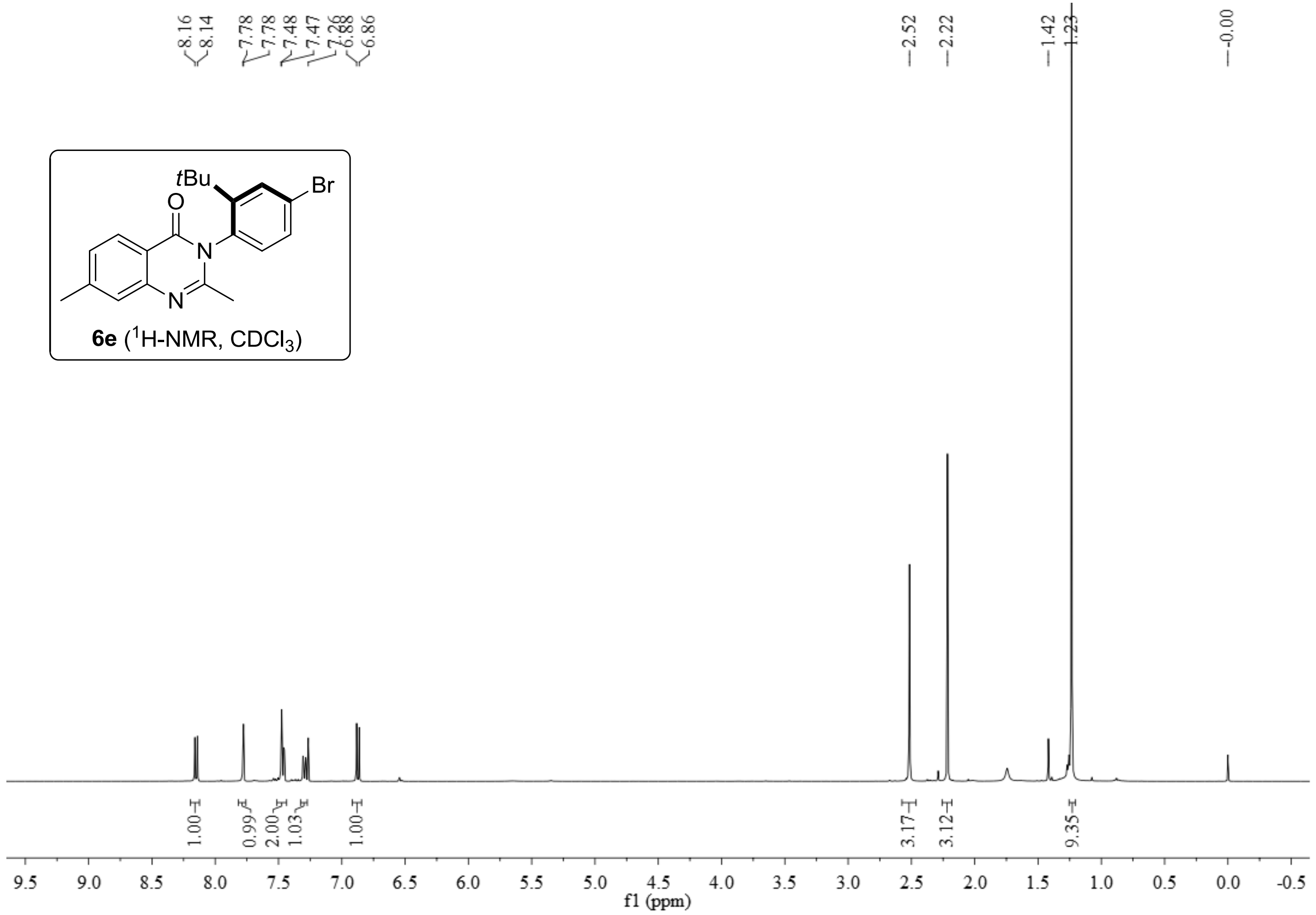


**Supplementary Figure 150.** $^{1}$H NMR of **6e**

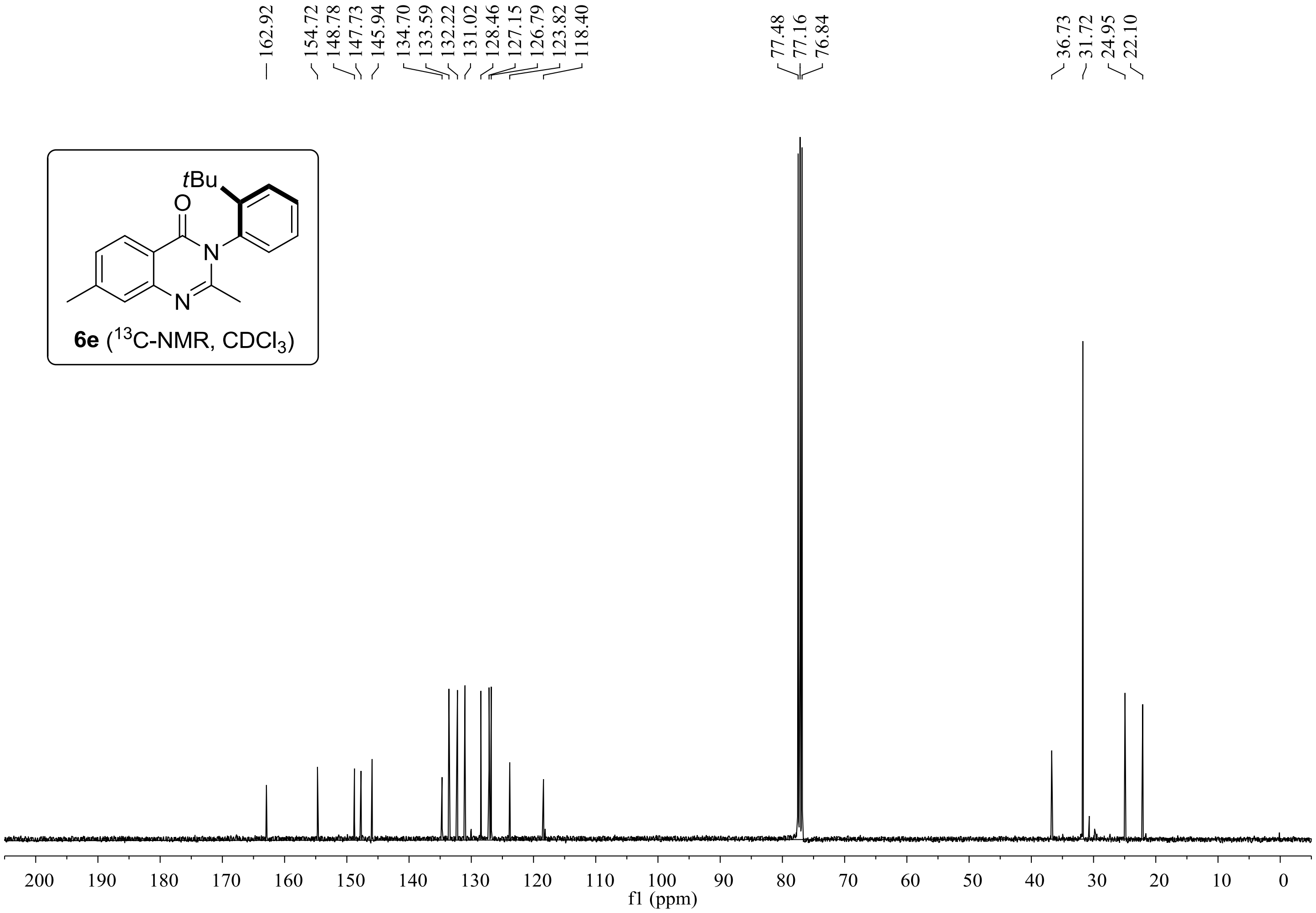


**Supplementary Figure 151.** $^{13}$C NMR of **6e**

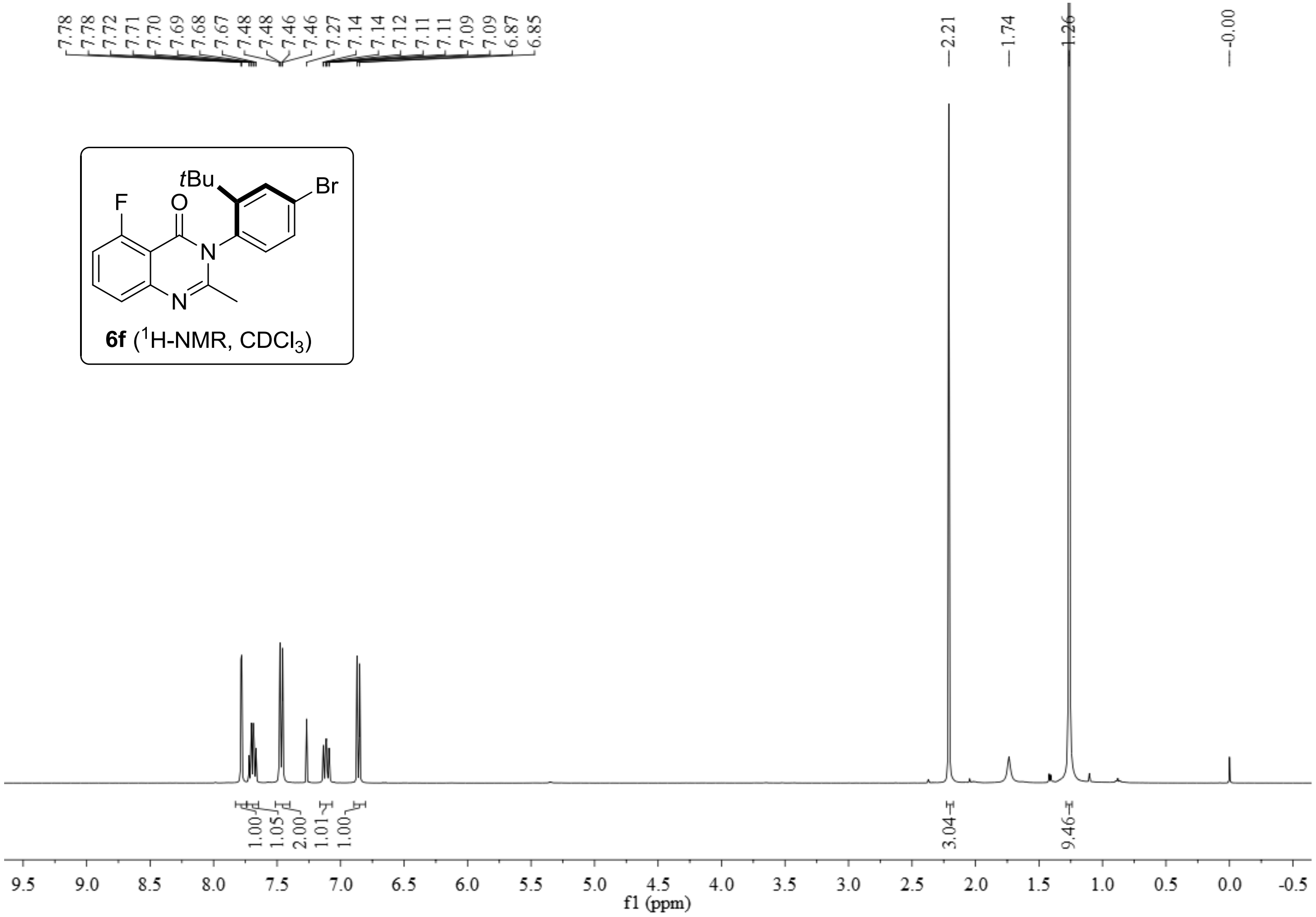


**Supplementary Figure 152.** $^{1}$H NMR of **6f**

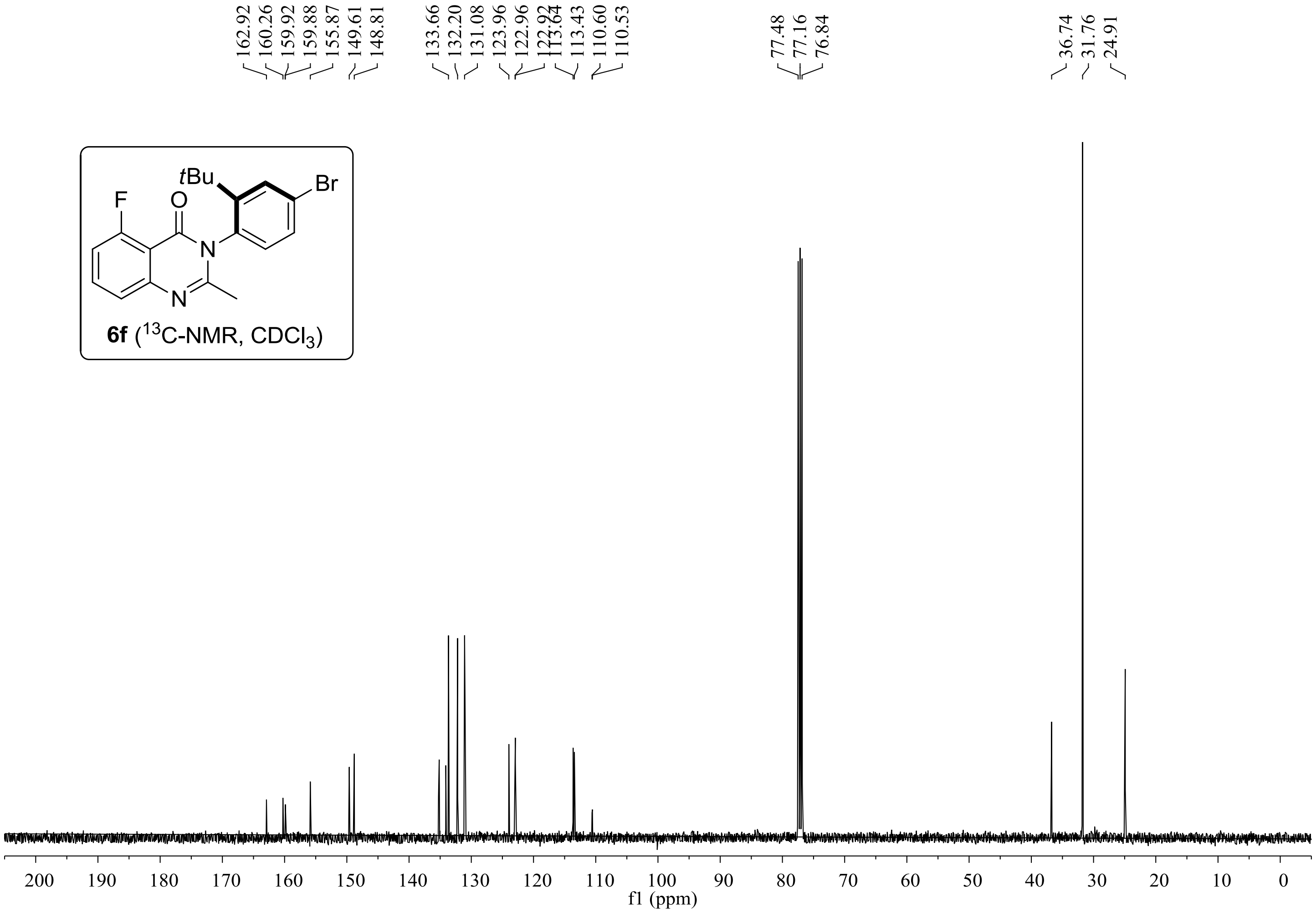


**Supplementary Figure 153.** $^{13}$C NMR of **6f**

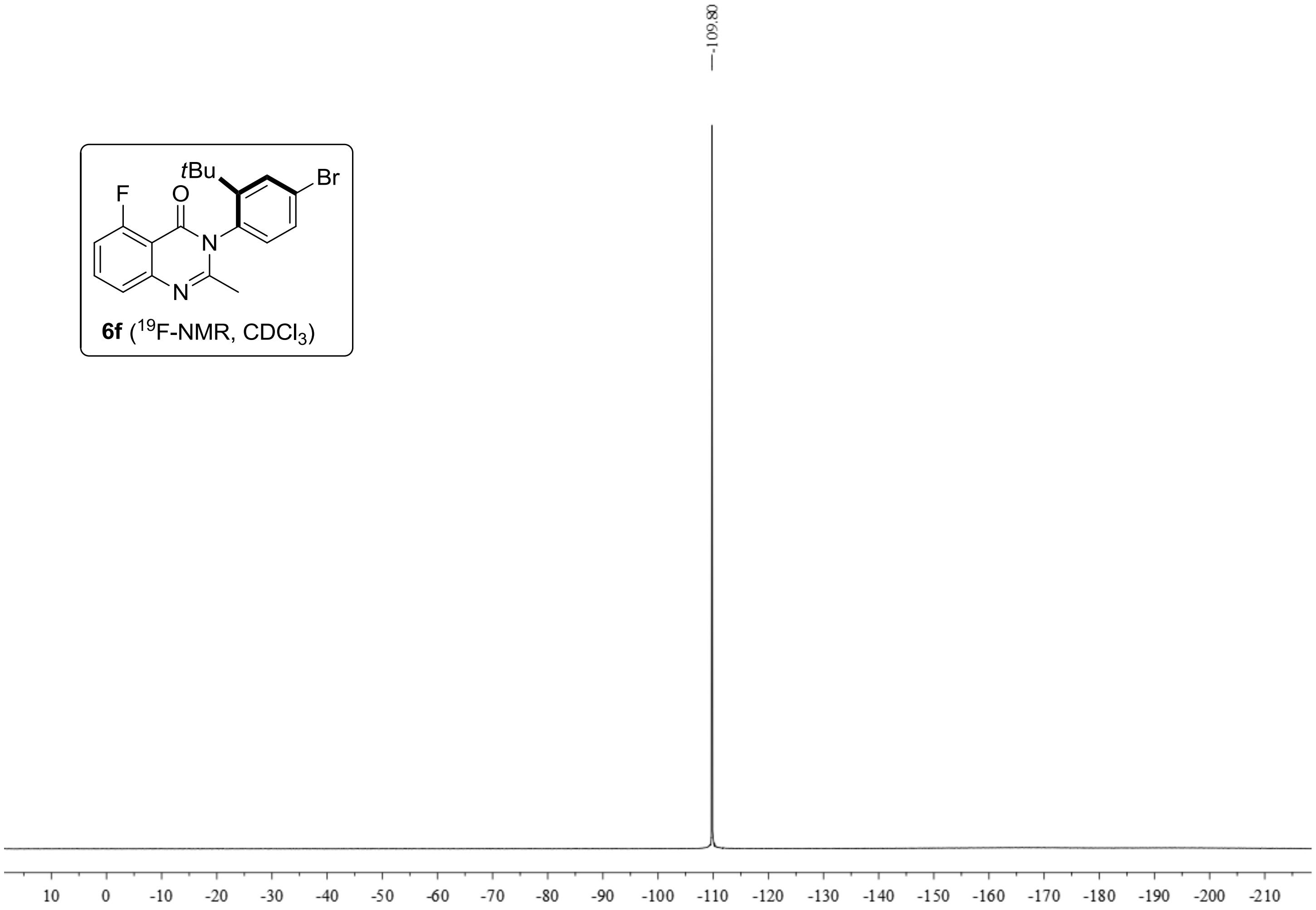


**Supplementary Figure 154.** $^{19}$F NMR of **6f**

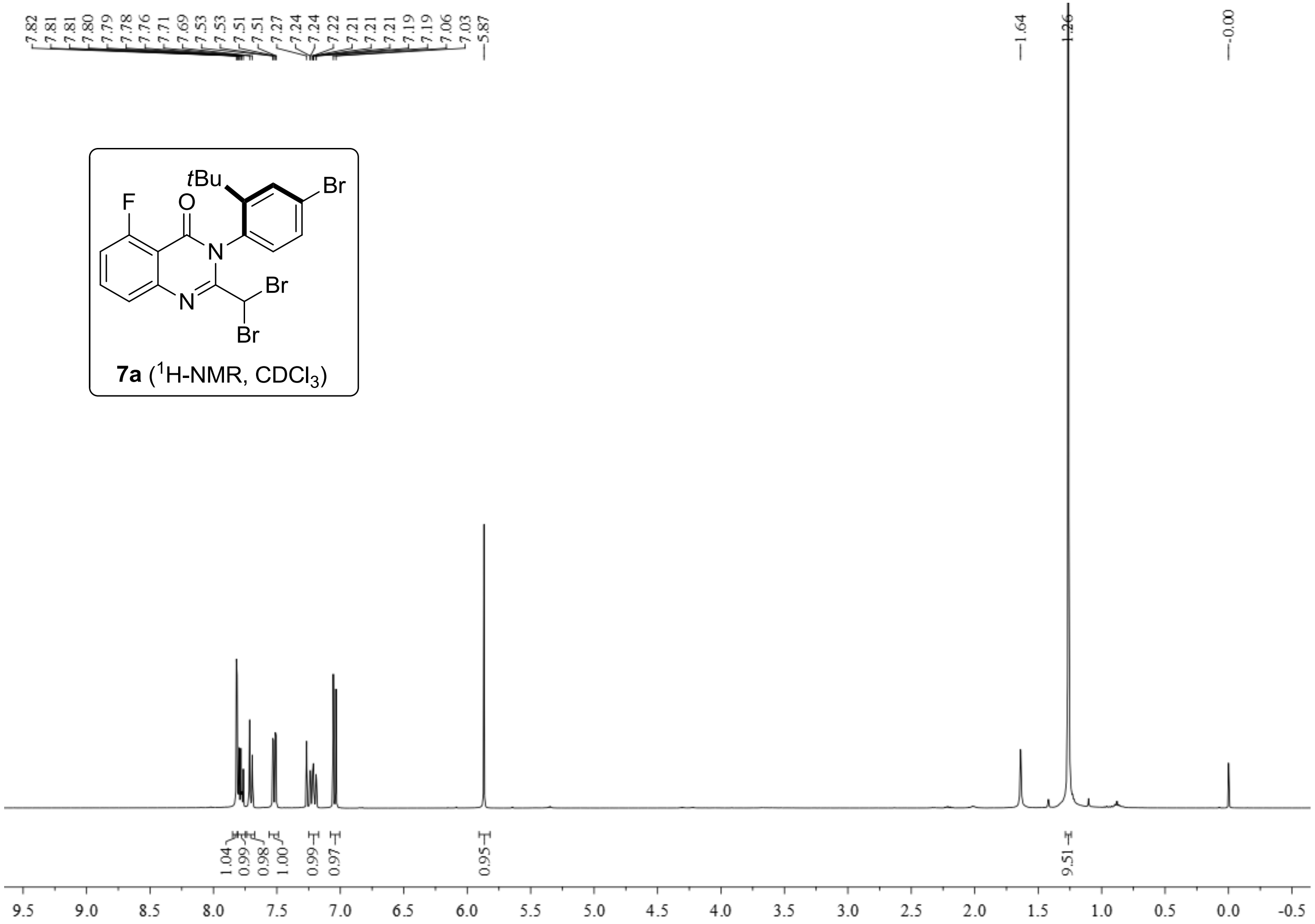


**Supplementary Figure 155.** $^1$H NMR of **7a**

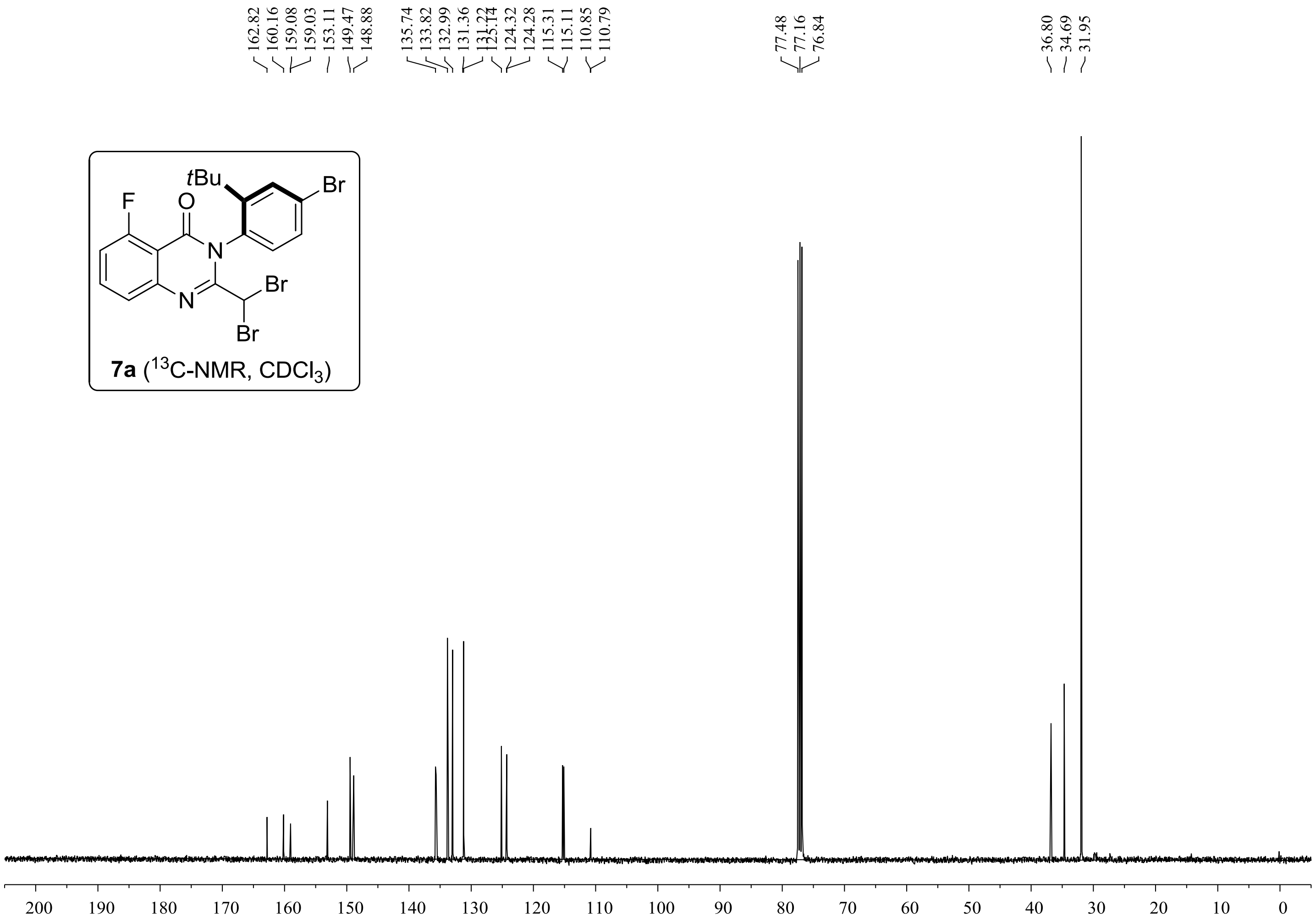


**Supplementary Figure 156.** $^{13}$C NMR of **7a**

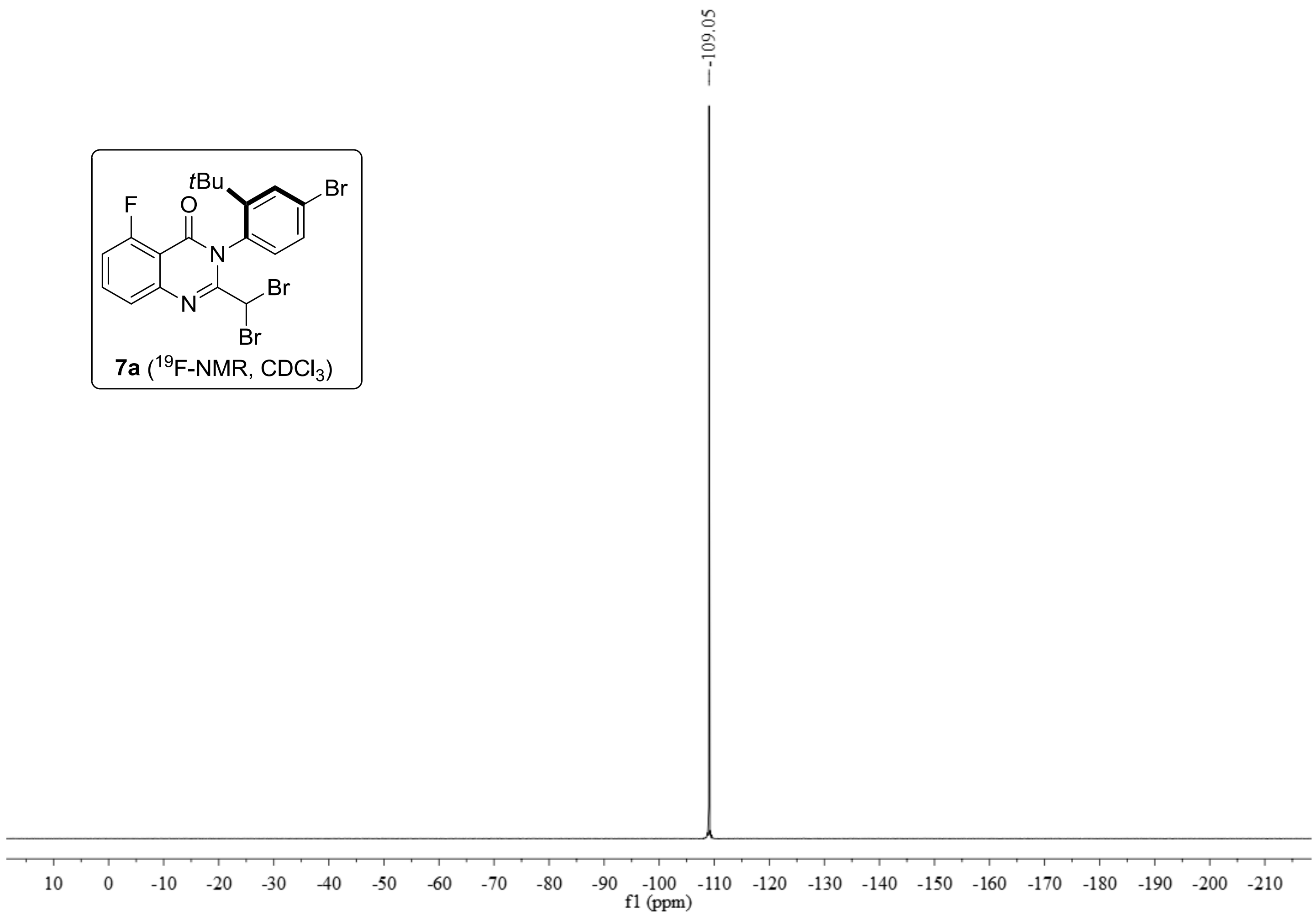


**Supplementary Figure 157.** $^{19}$F NMR of **7a**

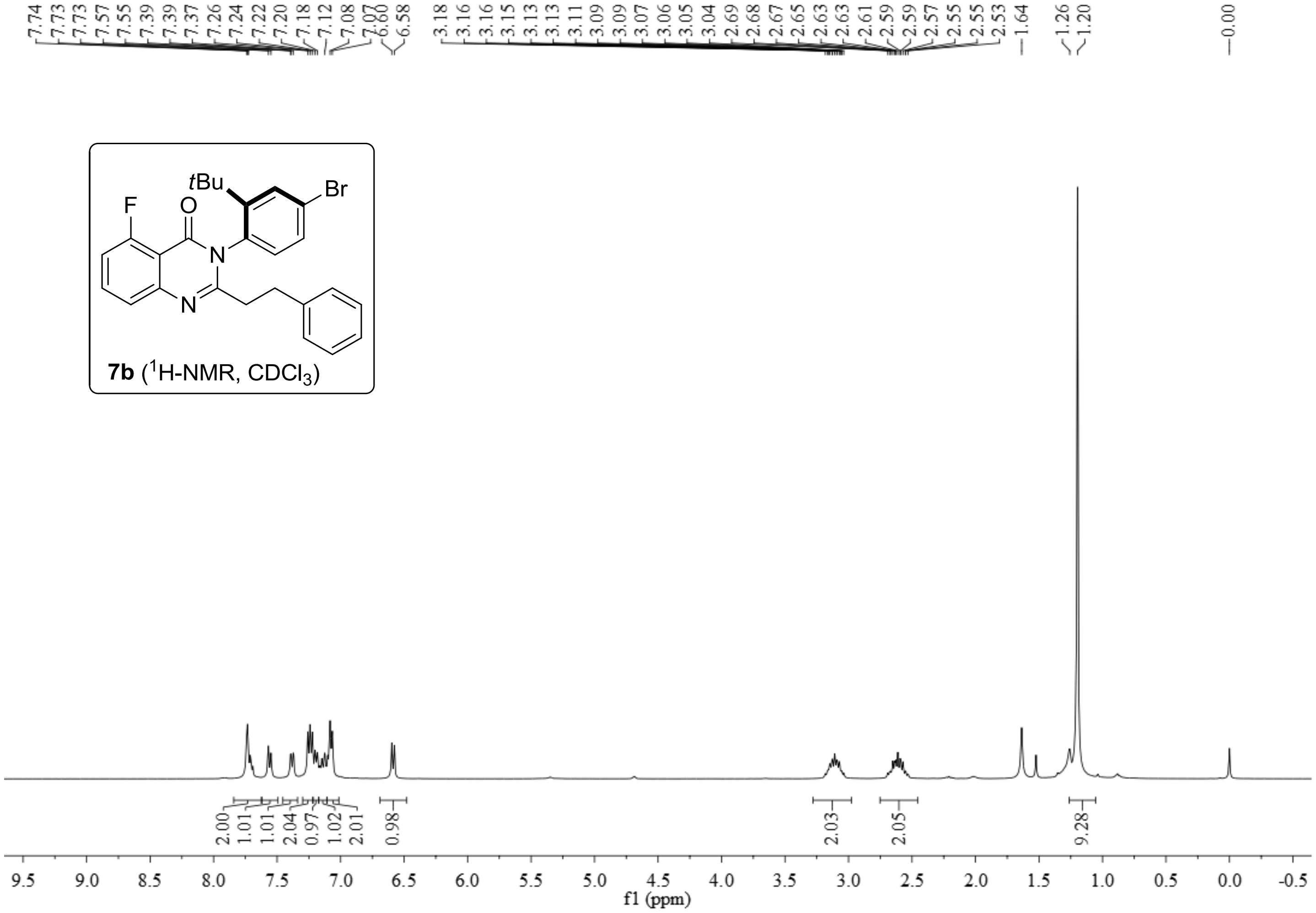


**Supplementary Figure 158.** $^1$H NMR of **7b**

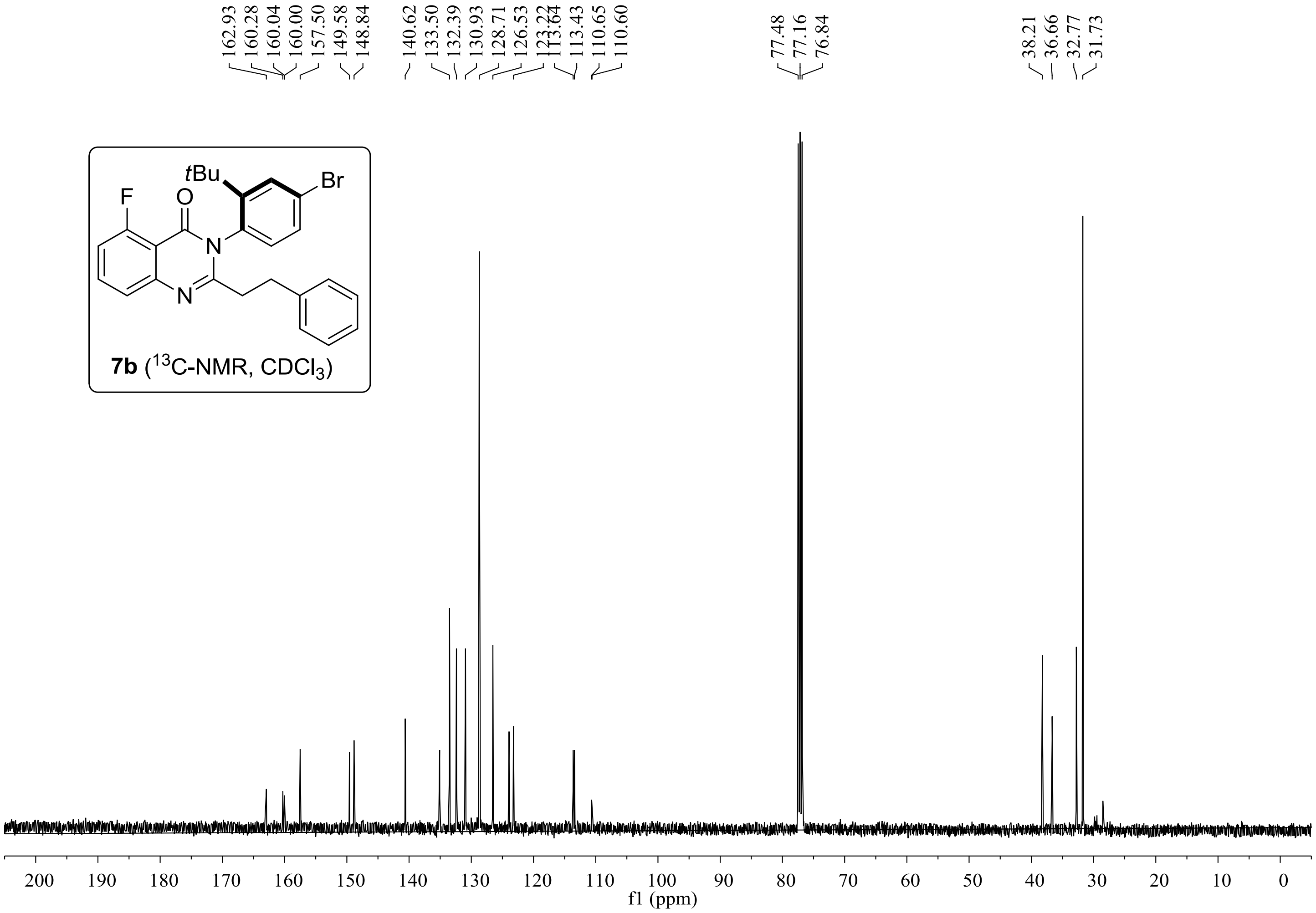


**Supplementary Figure 159.** $^{13}$C NMR of **7b**

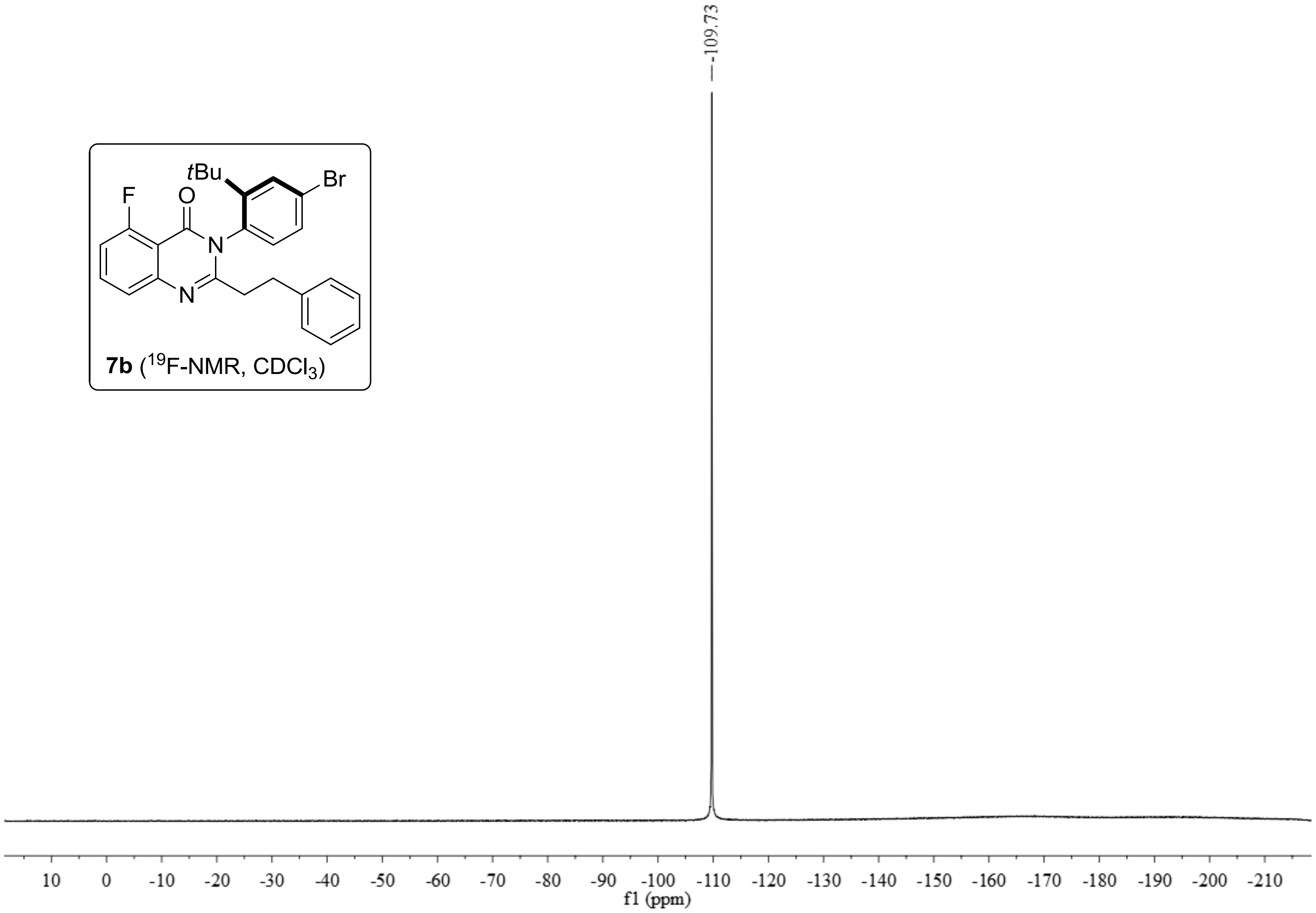


**Supplementary Figure 160.** $^{1}$H NMR of **7b**

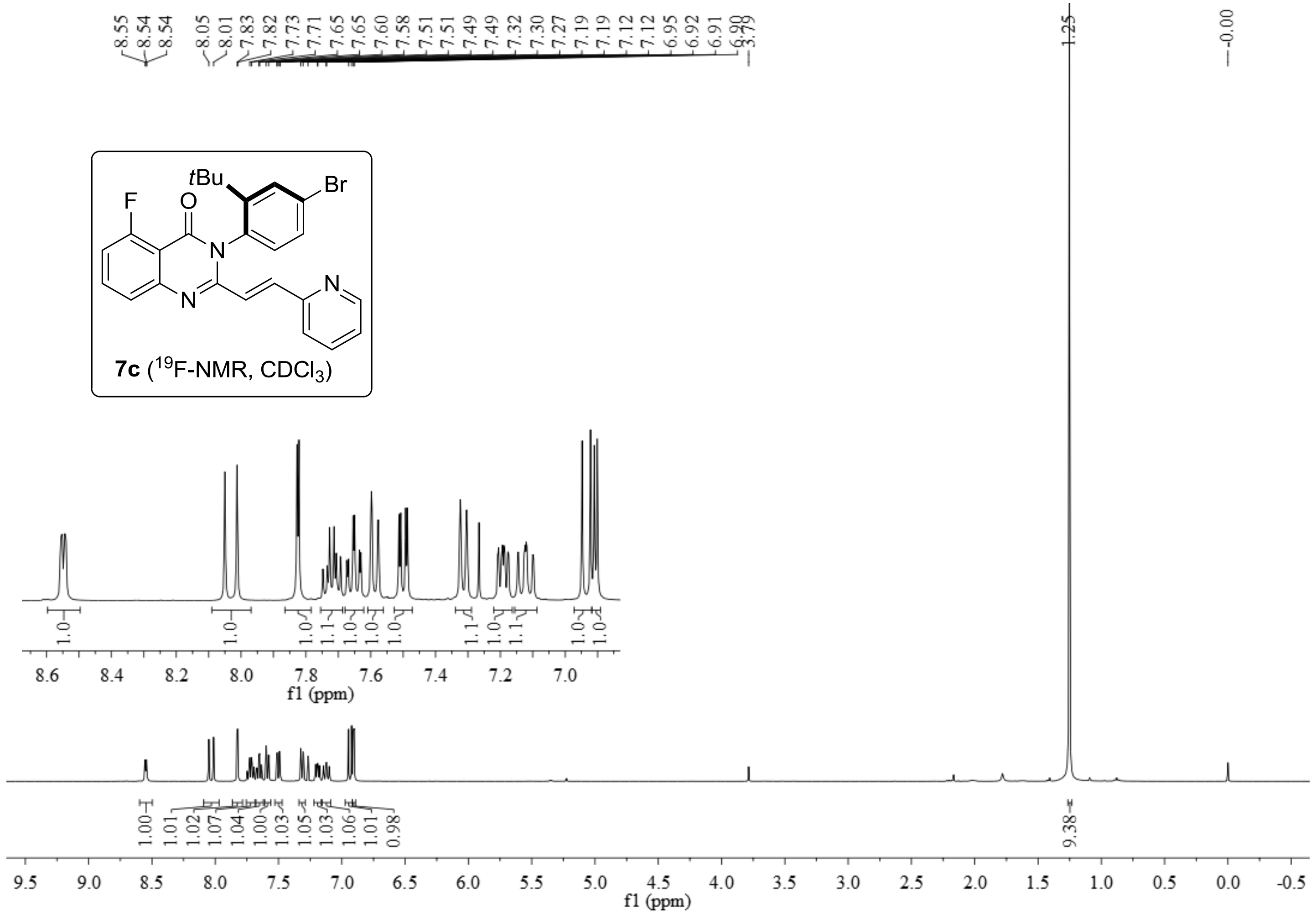


**Supplementary Figure 161.** $^1$H NMR of **7c**

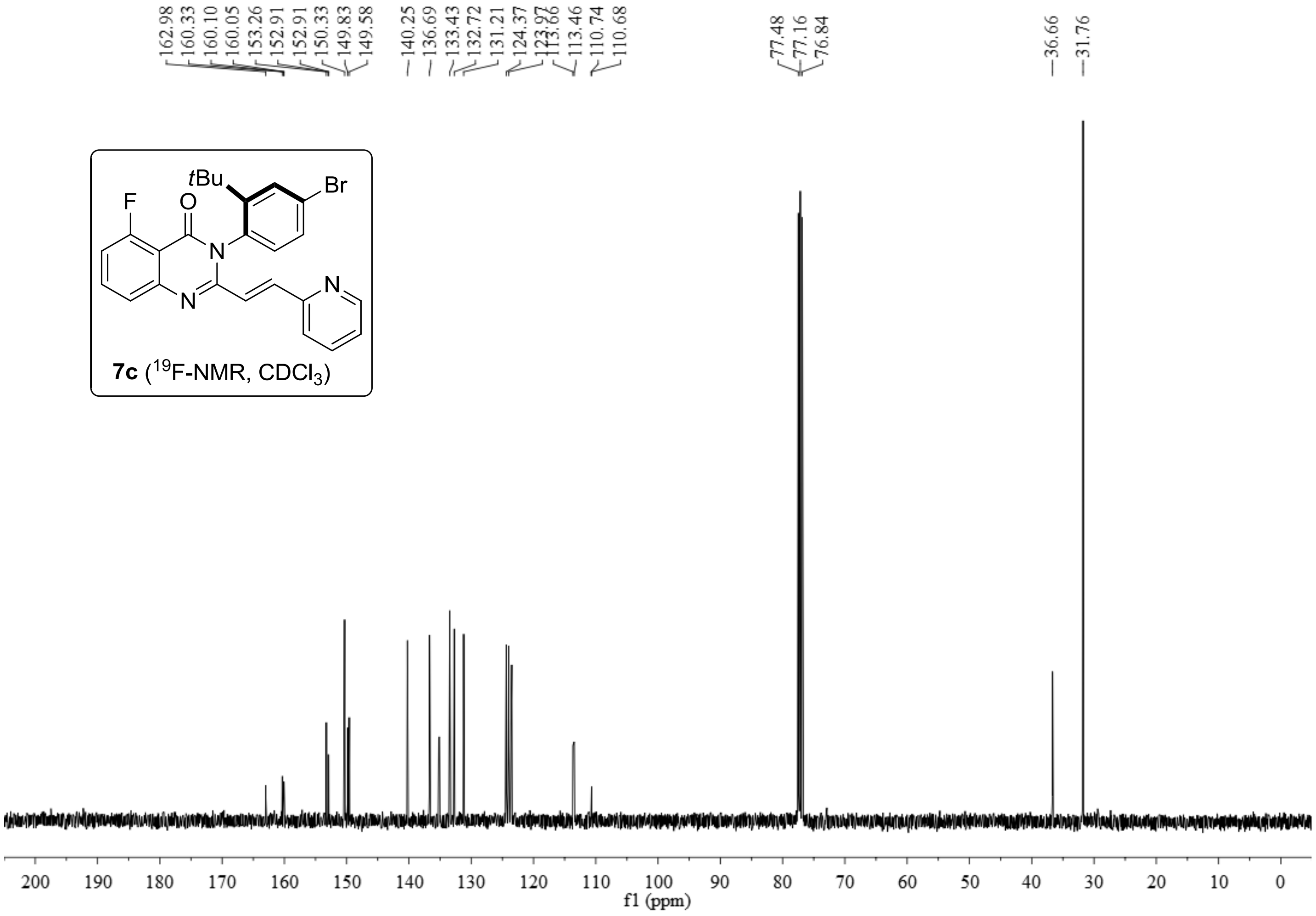


**Supplementary Figure 162.** $^{13}$C NMR of **7c**

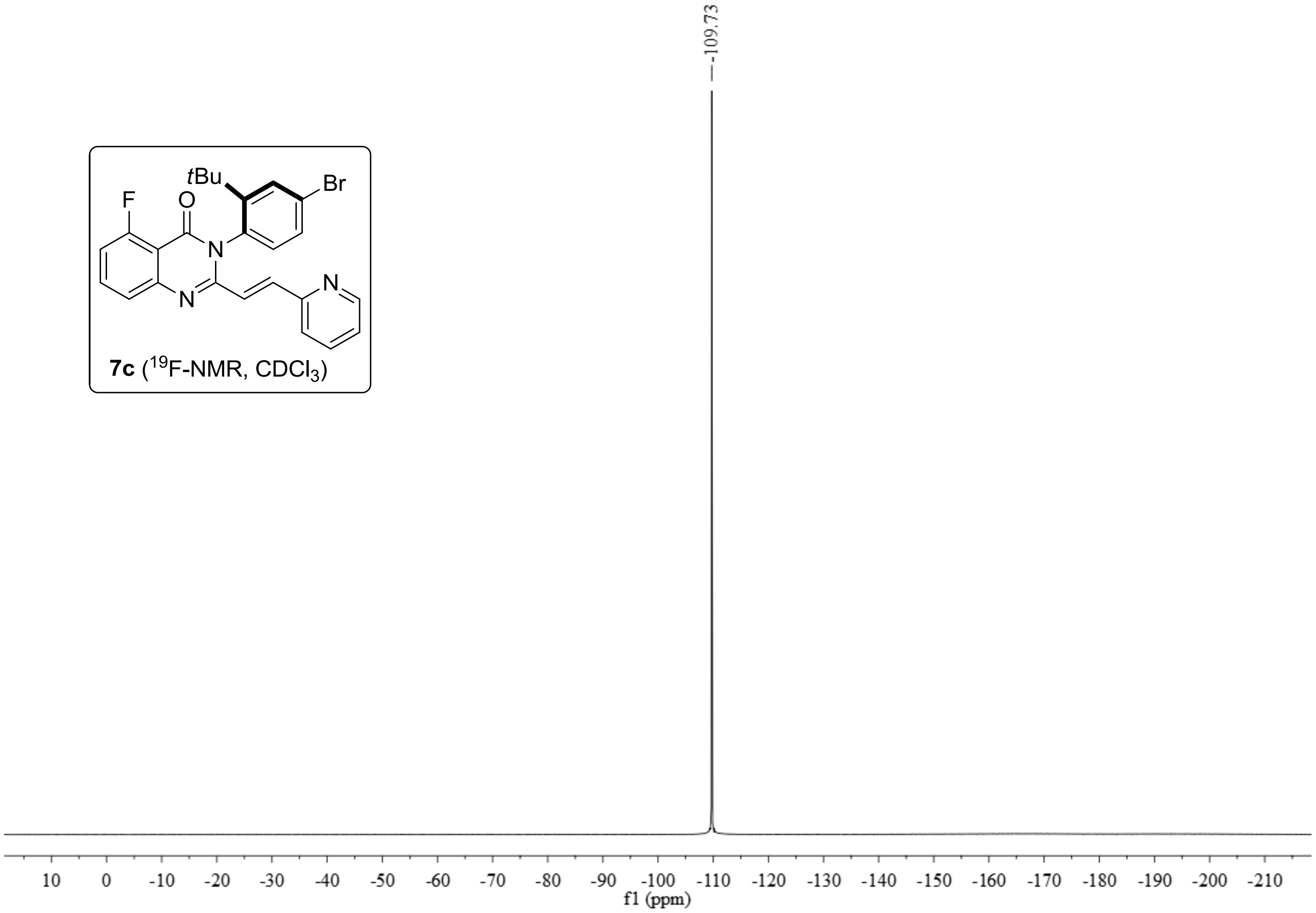


**Supplementary Figure 163.** $^{19}$F NMR of **7c**

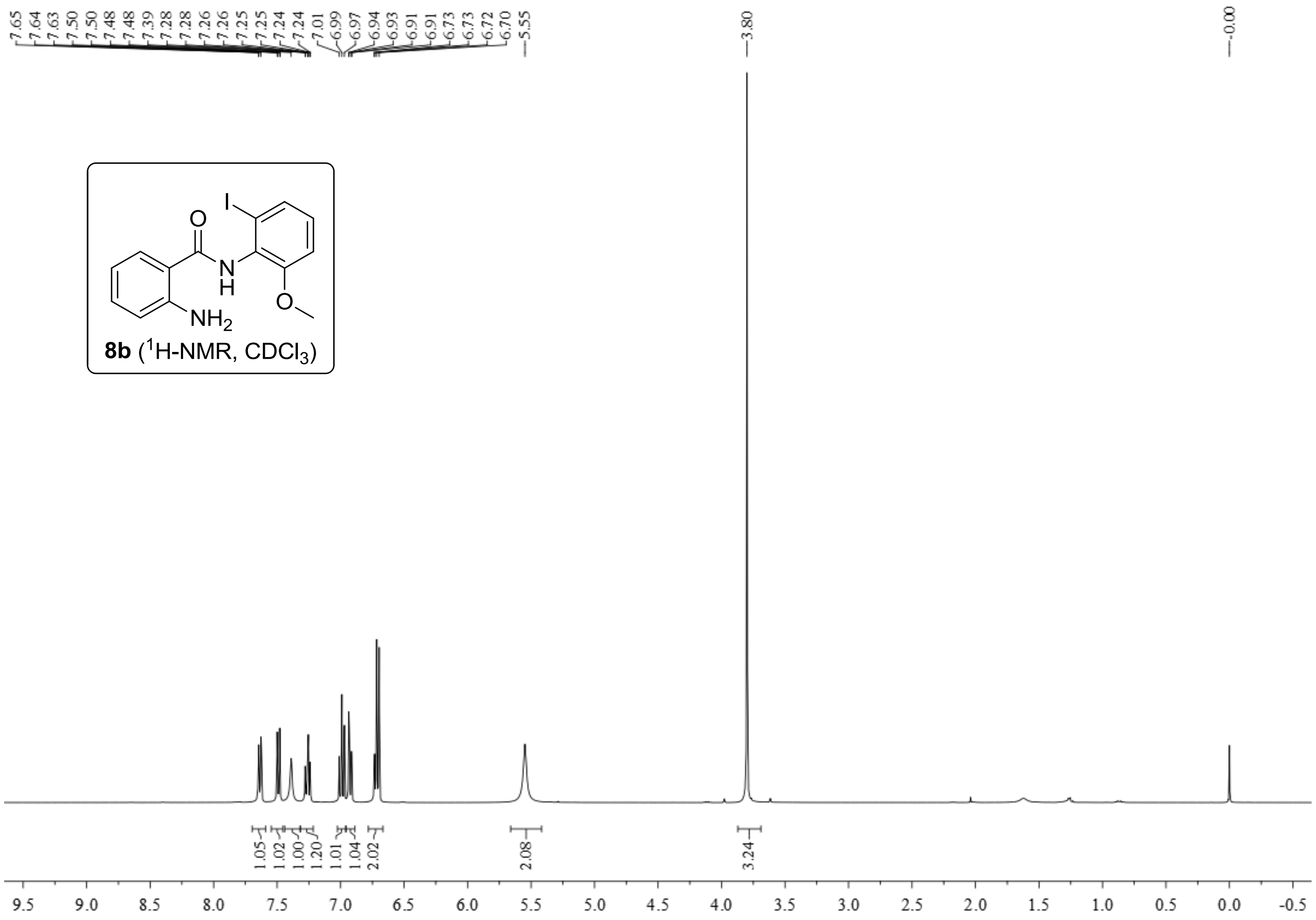


**Supplementary Figure 164.** $^{1}$H NMR of **8b**

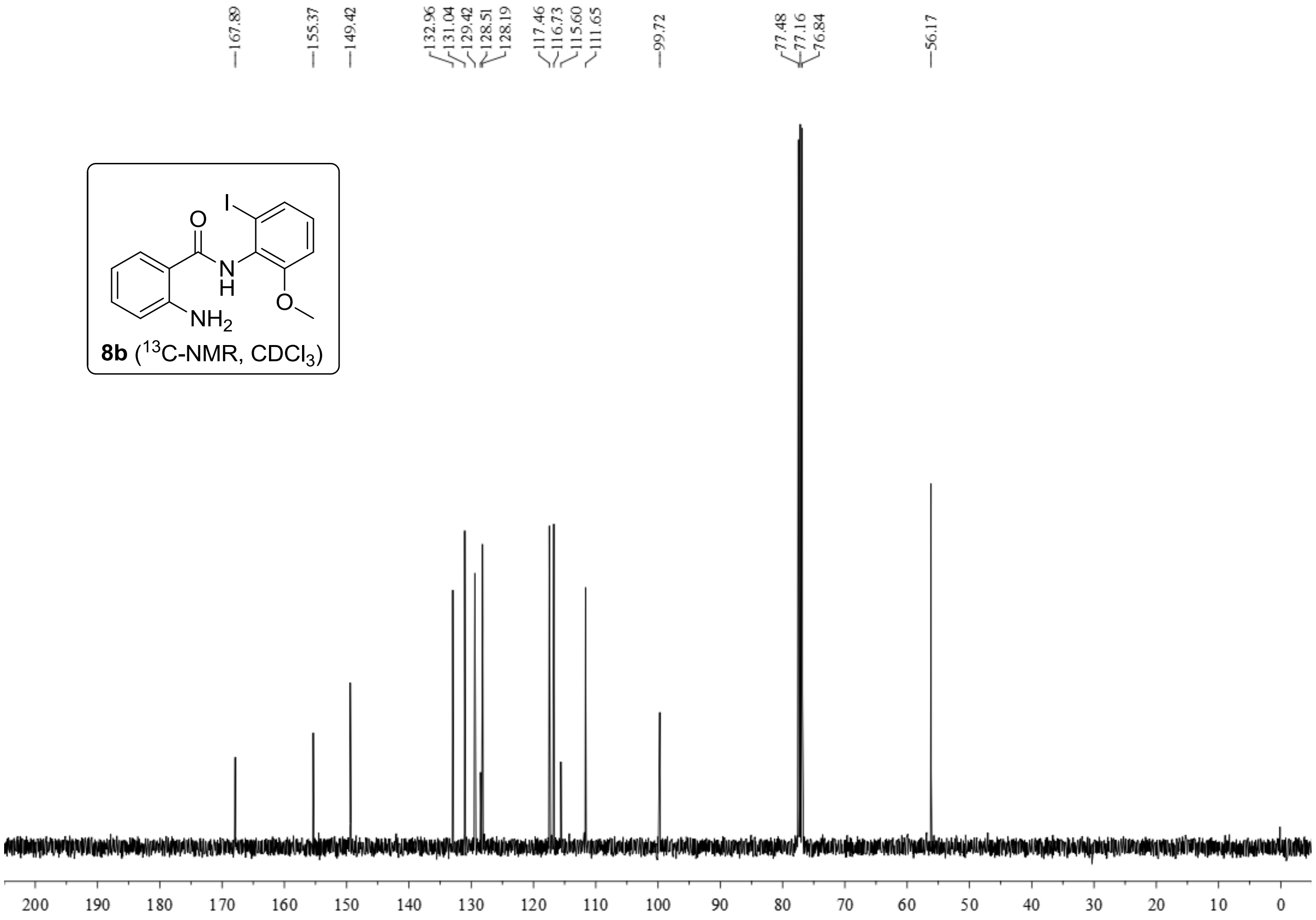


**Supplementary Figure 165.** $^{13}$C NMR of **8b**

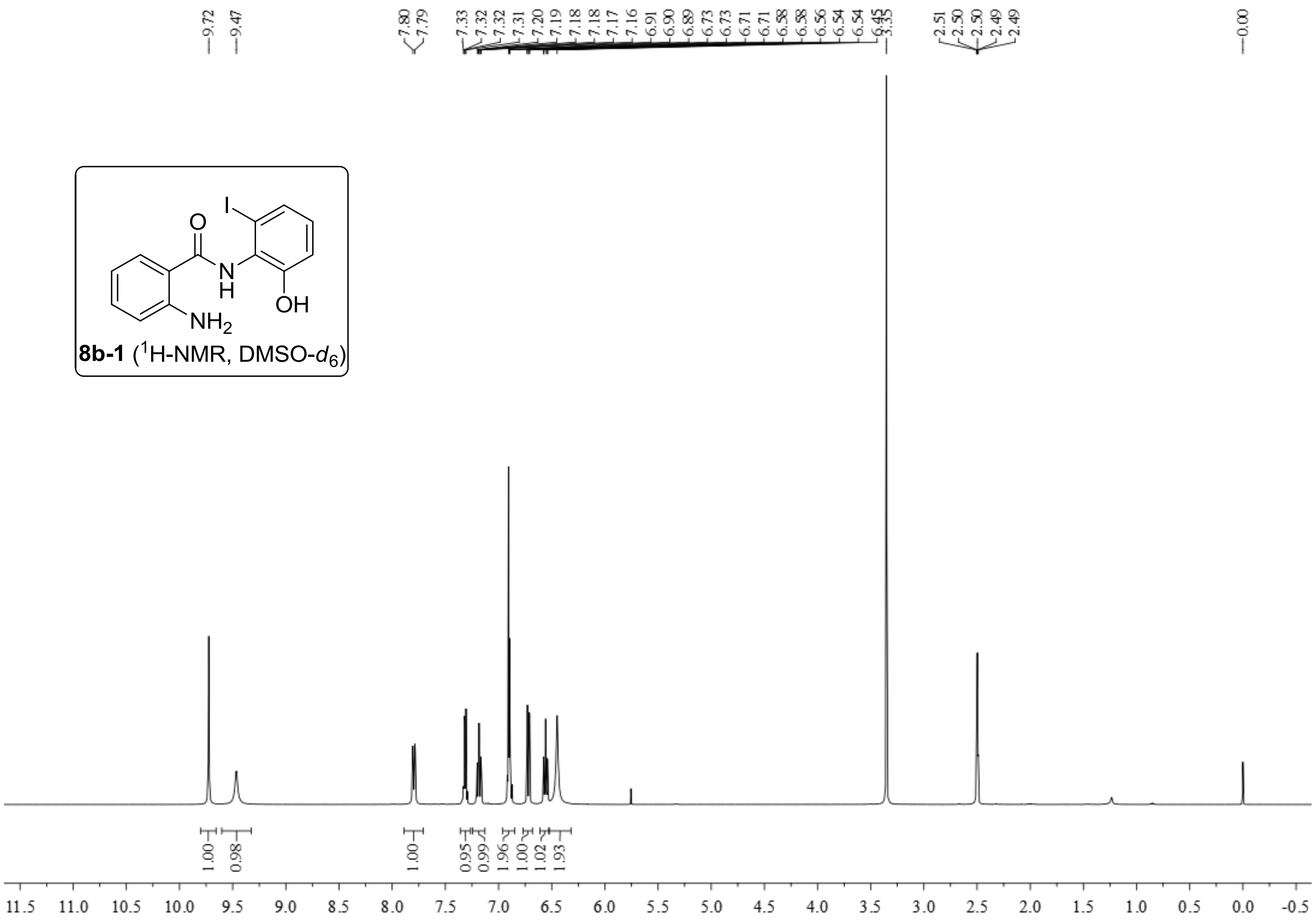


**Supplementary Figure 166.** ¹H NMR of **8b-1**

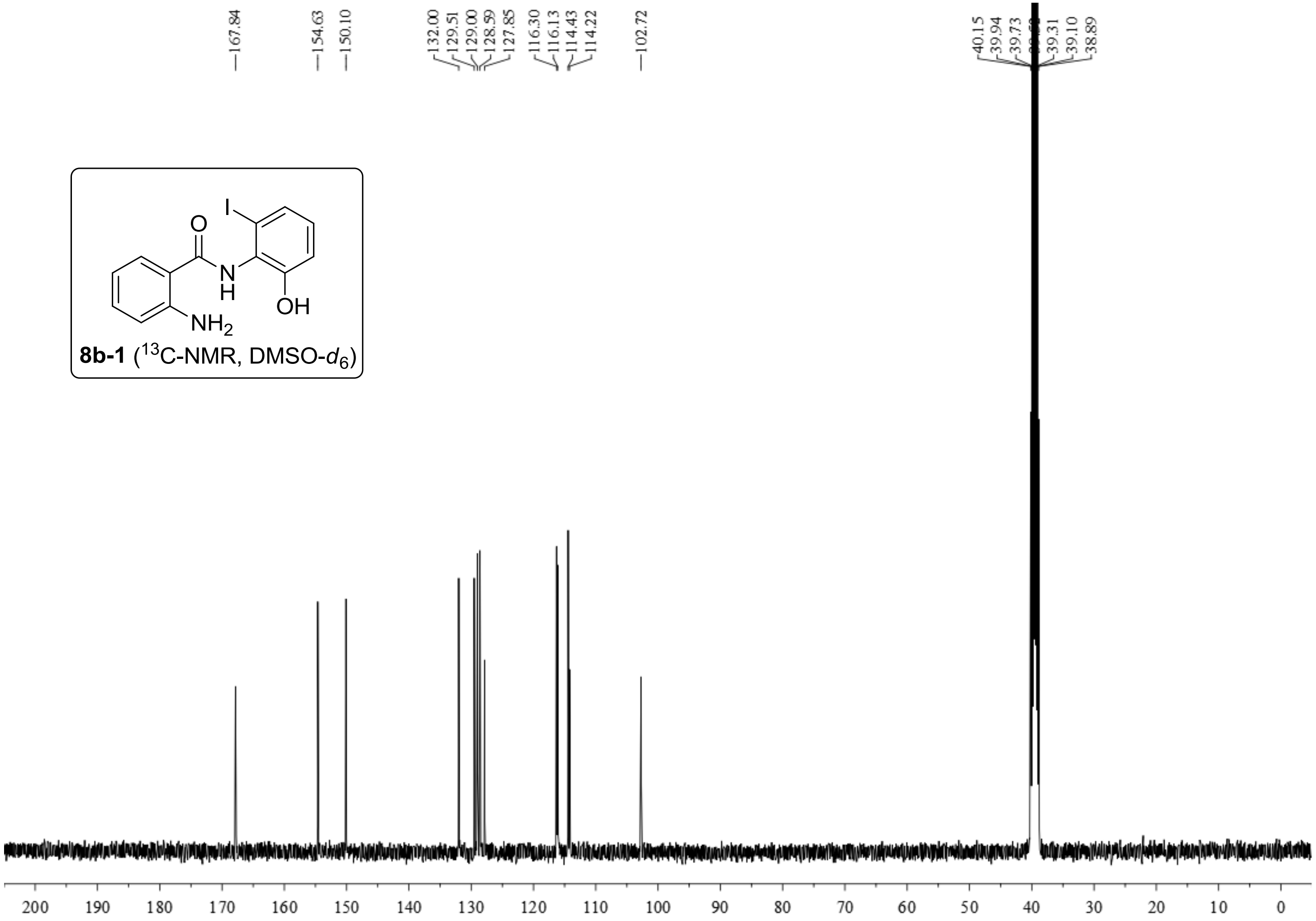


**Supplementary Figure 167.** $^{13}$C NMR of **8b-1**

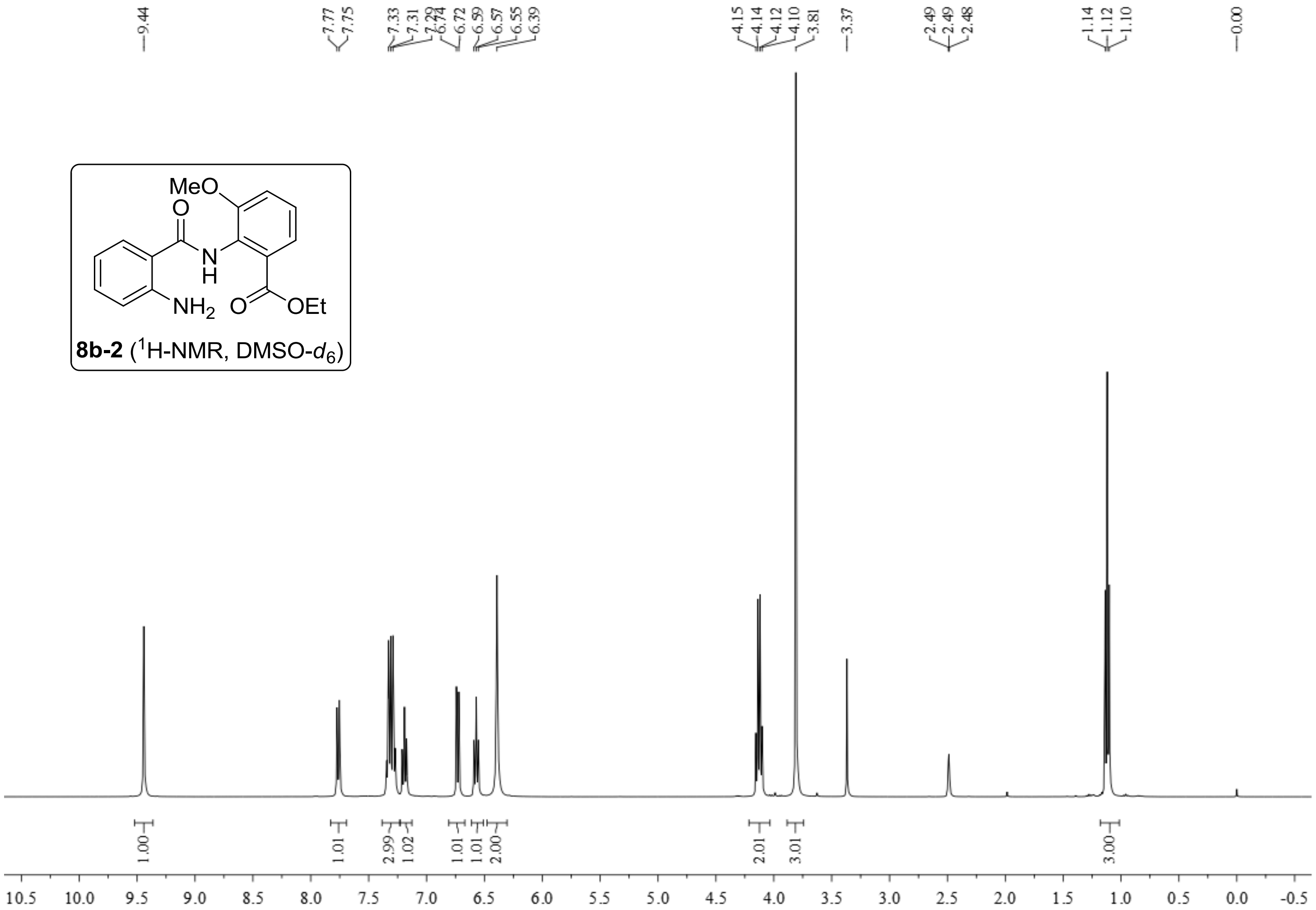


**Supplementary Figure 168.** $^{1}$H NMR of **8b-2**

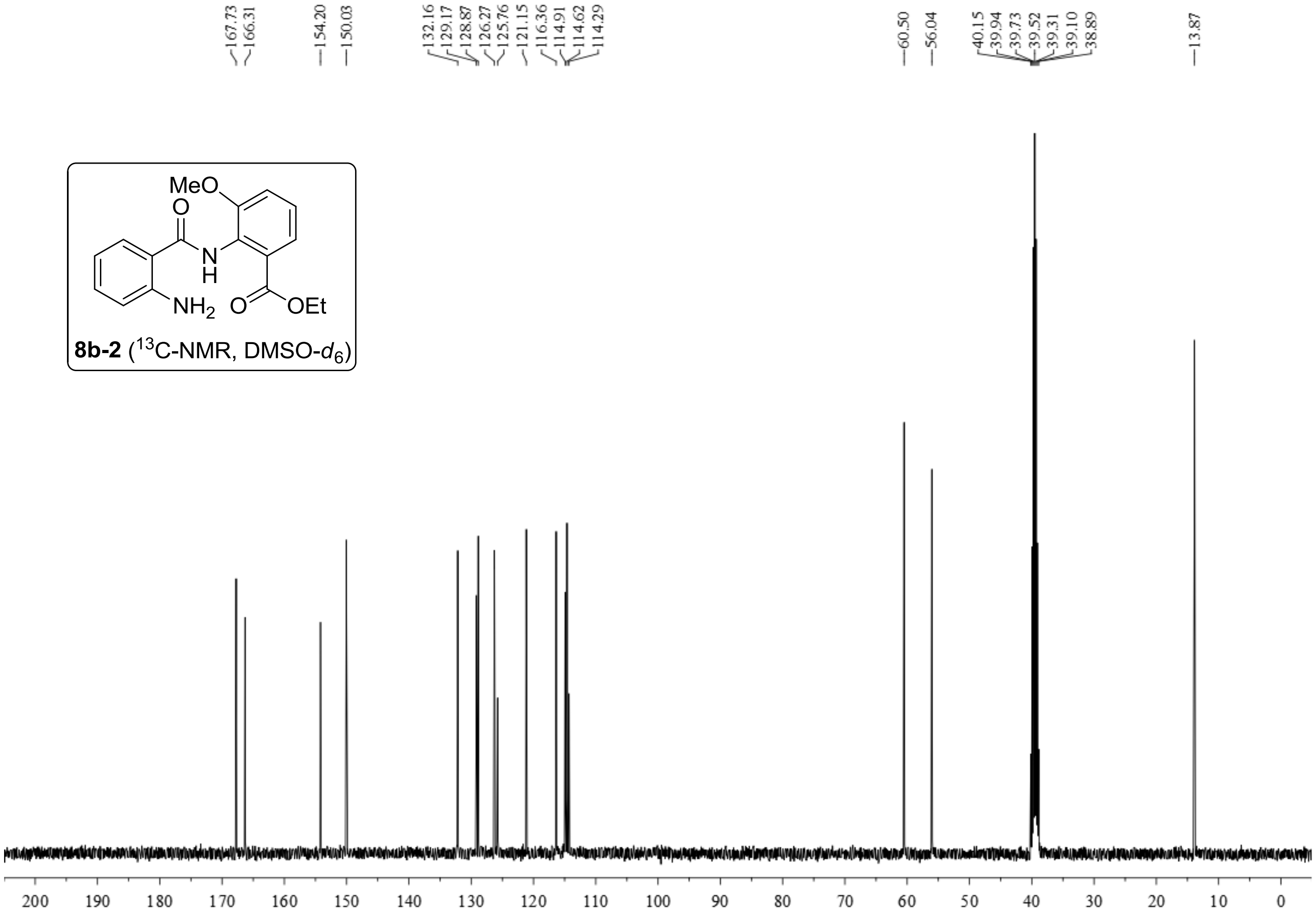


**Supplementary Figure 169.** $^{13}$C NMR of **8b-2**

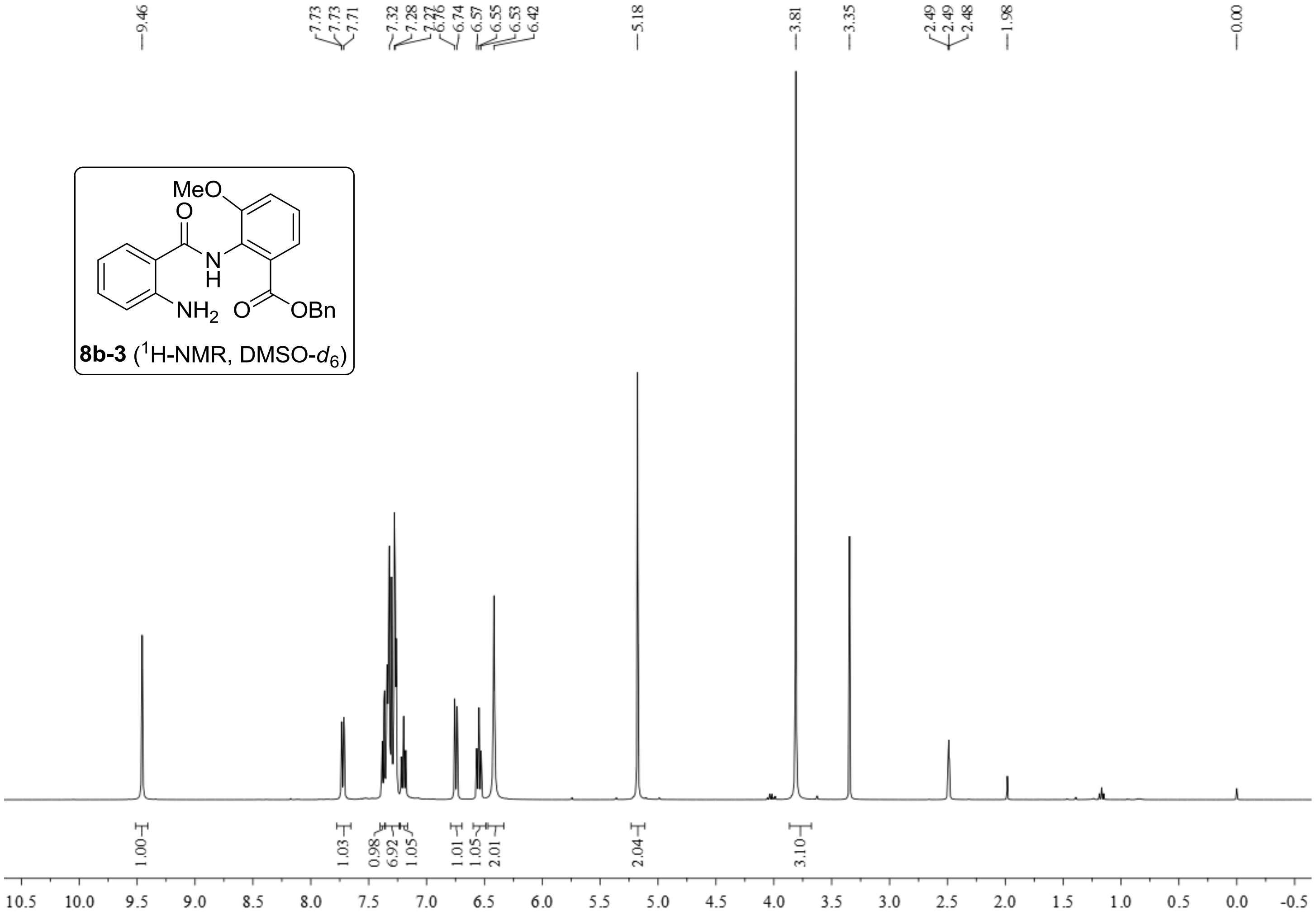


**Supplementary Figure 170.** $^{1}$H NMR of **8b-3**

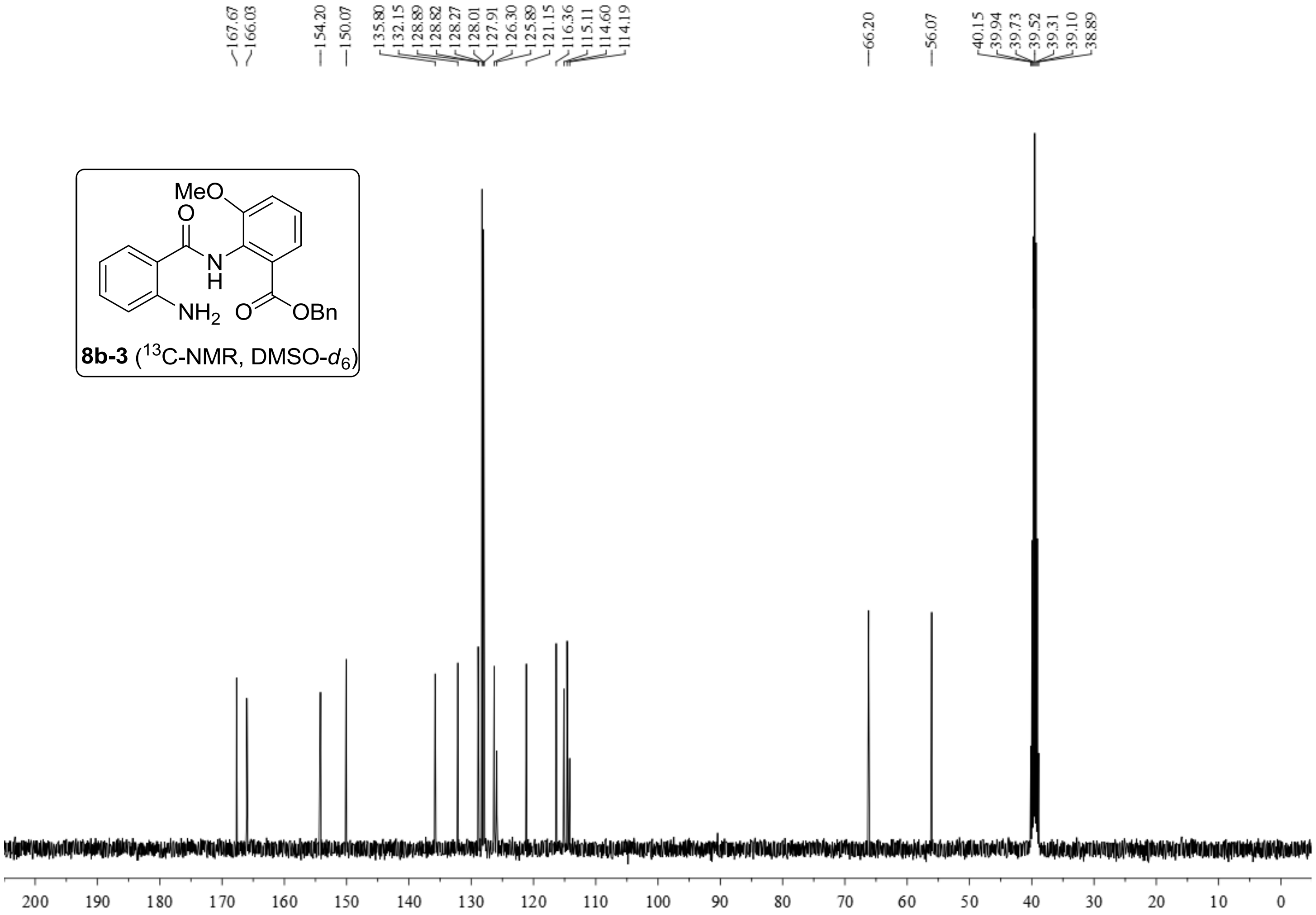


**Supplementary Figure 171.** $^{13}$C NMR of **8b-3**

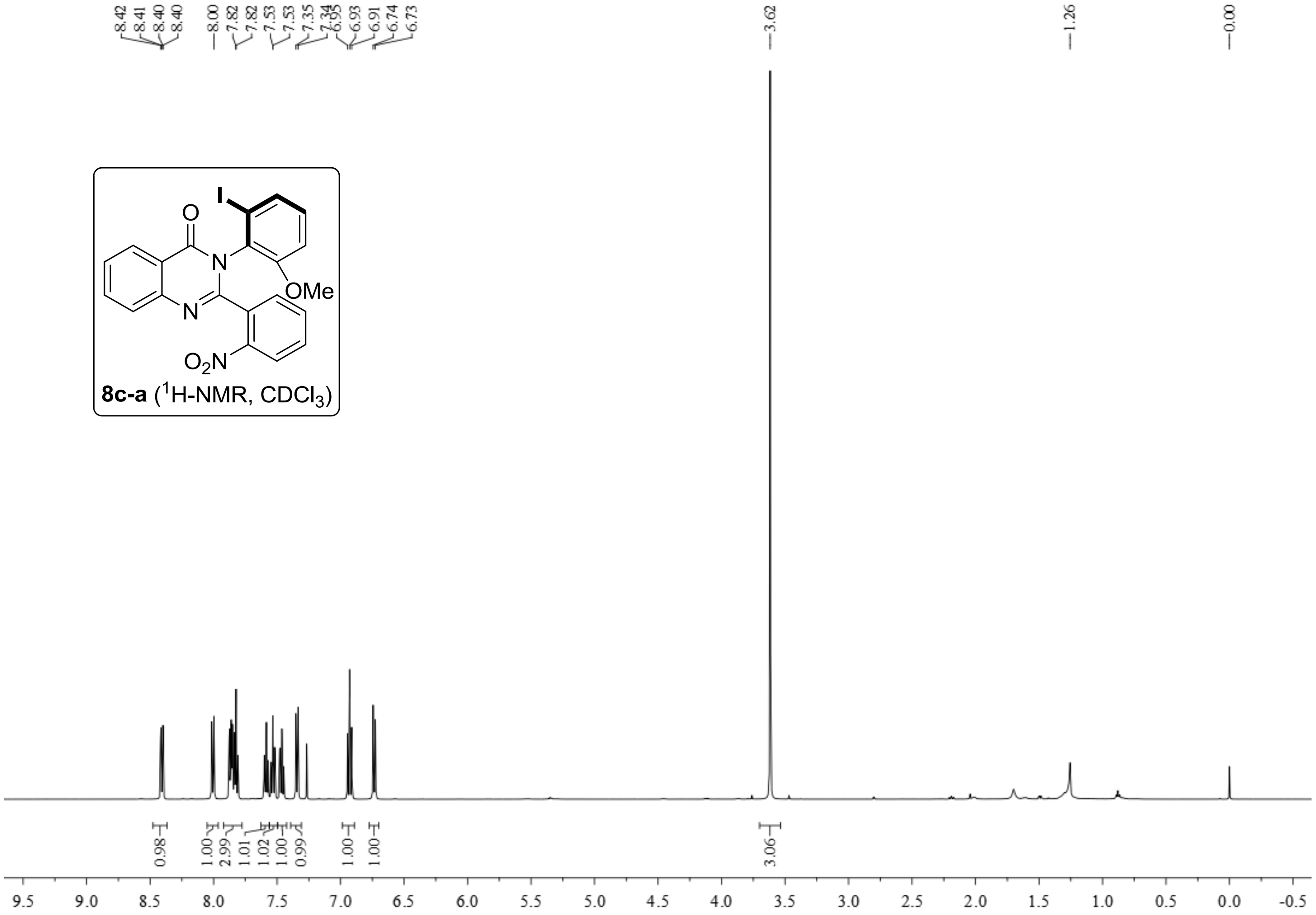


**Supplementary Figure 172.** $^1$H NMR of **8c-a**

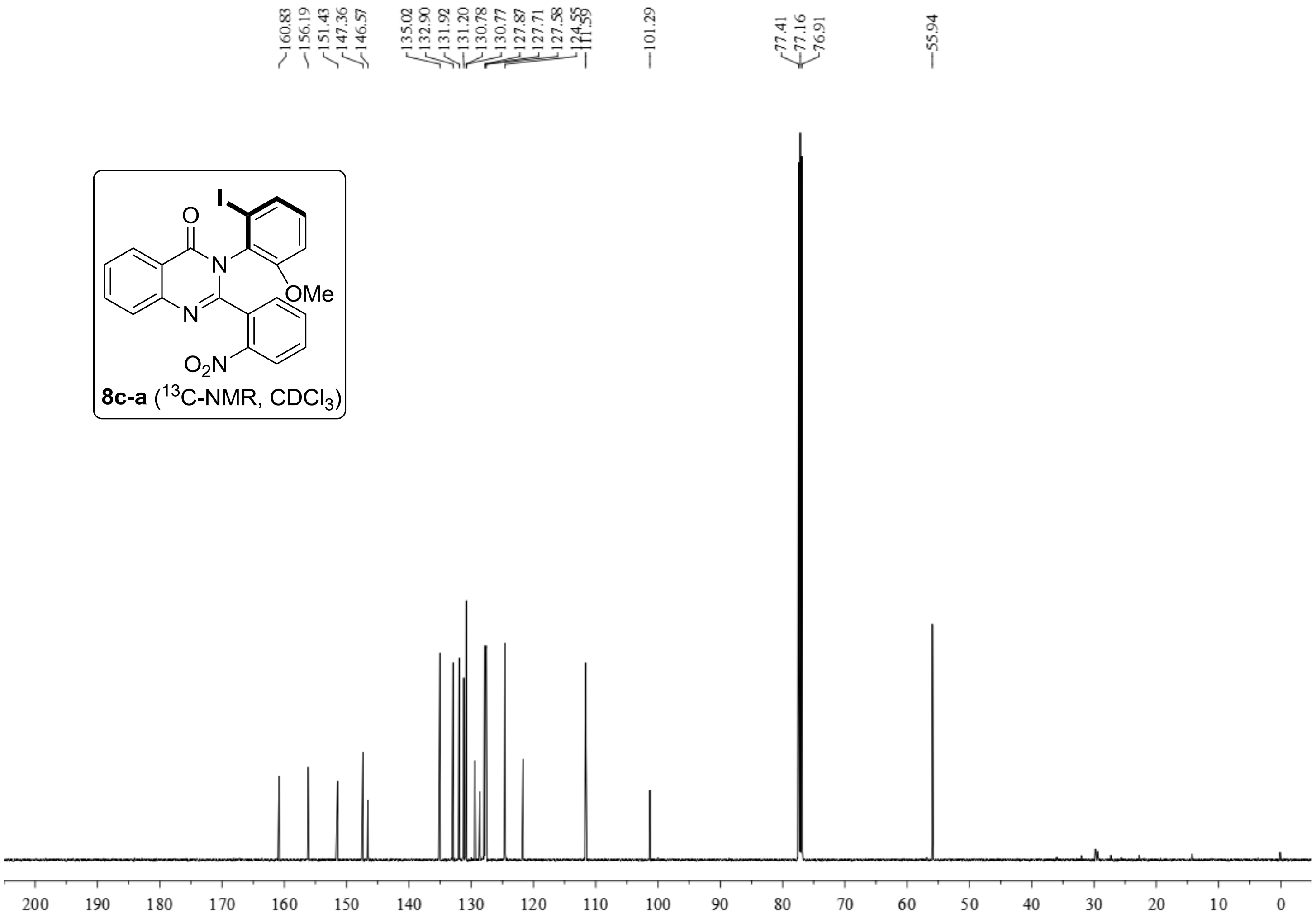


**Supplementary Figure 173.** $^{13}$C NMR of **8c-a**

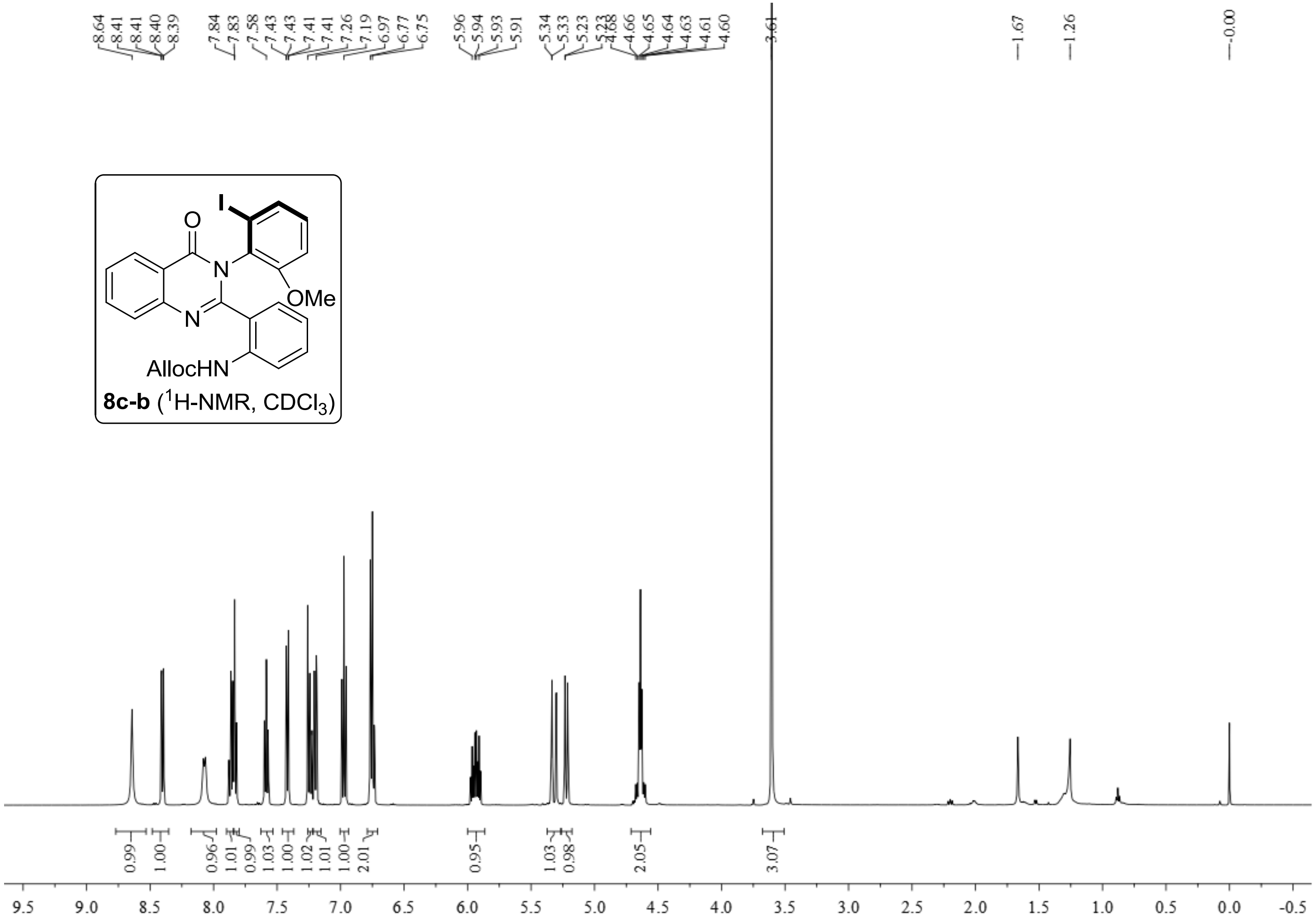


**Supplementary Figure 174.** $^1$H NMR of **8c-b**

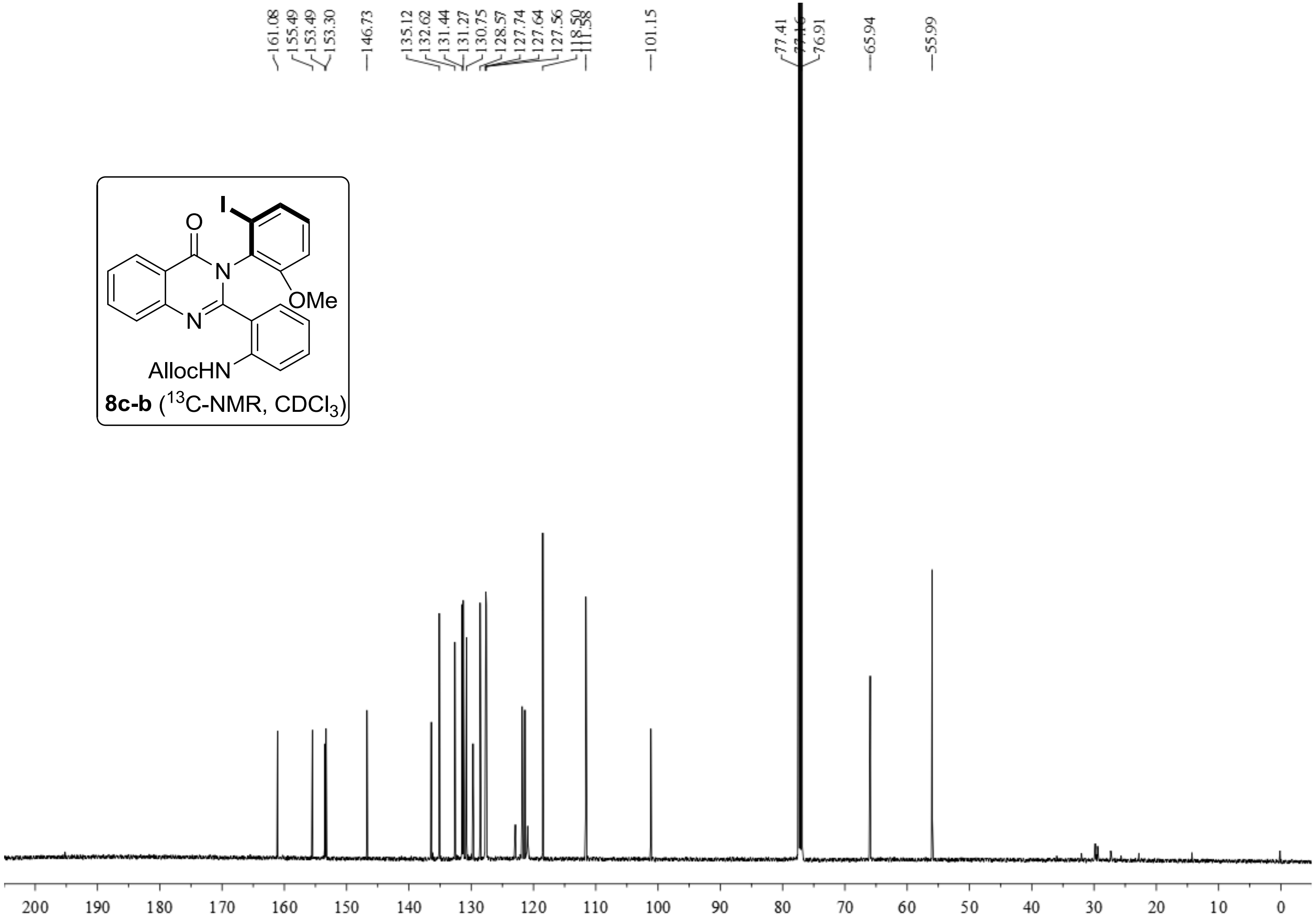


**Supplementary Figure 175.** $^{13}$C NMR of **8c-b**

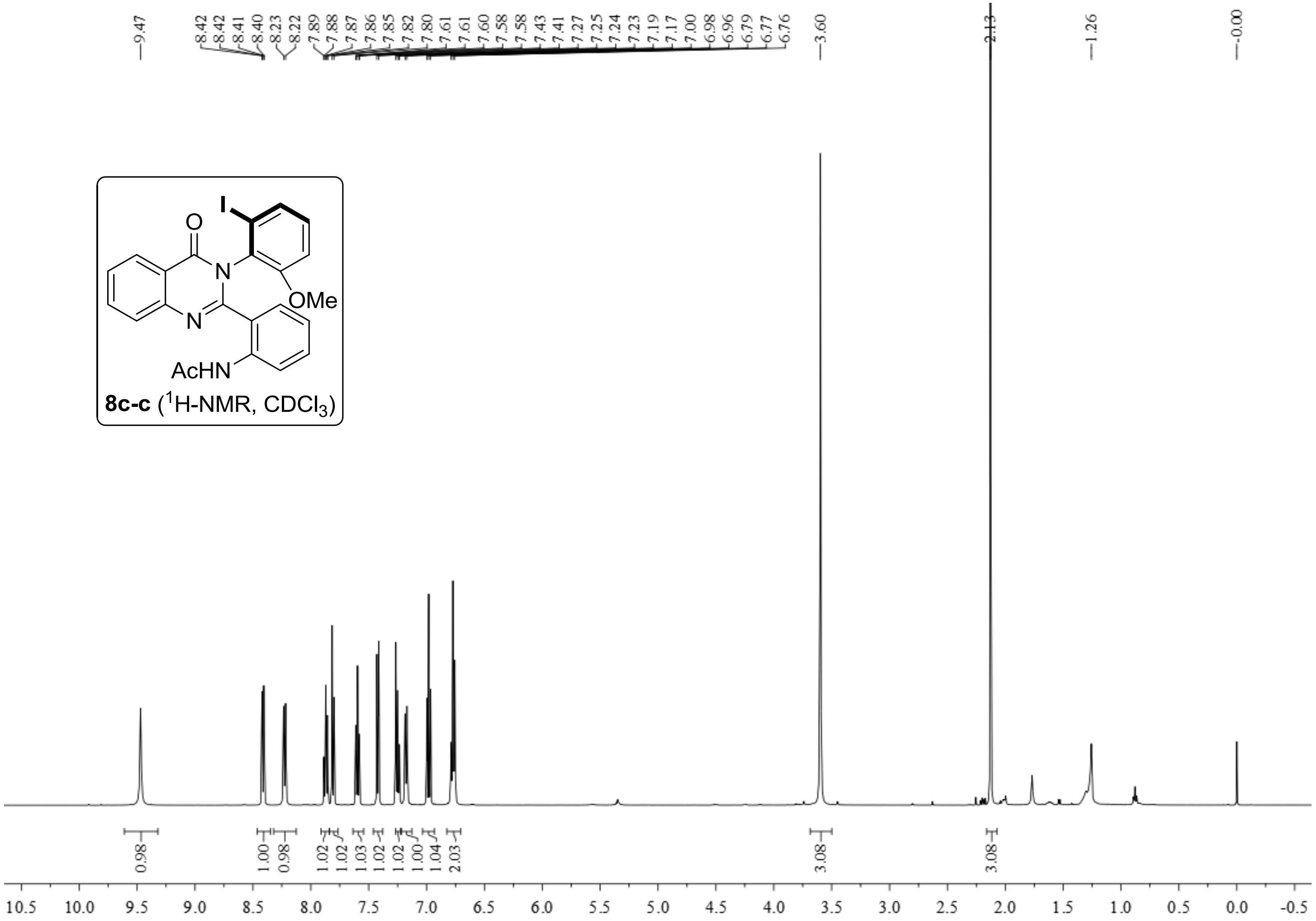


**Supplementary Figure 176.** $^{1}$H NMR of **8c-c**

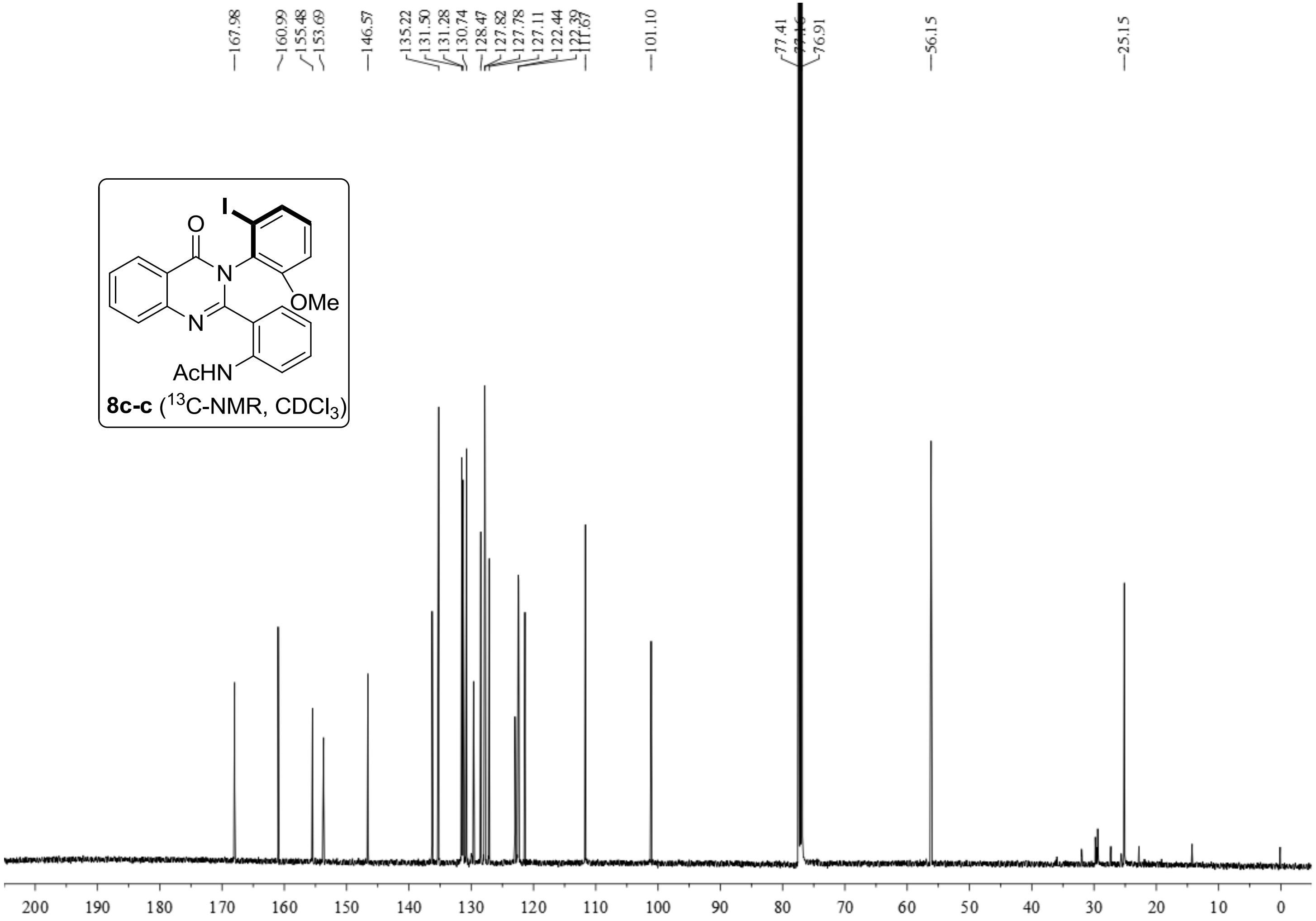


**Supplementary Figure 177.** $^{13}$C NMR of **8c-c**

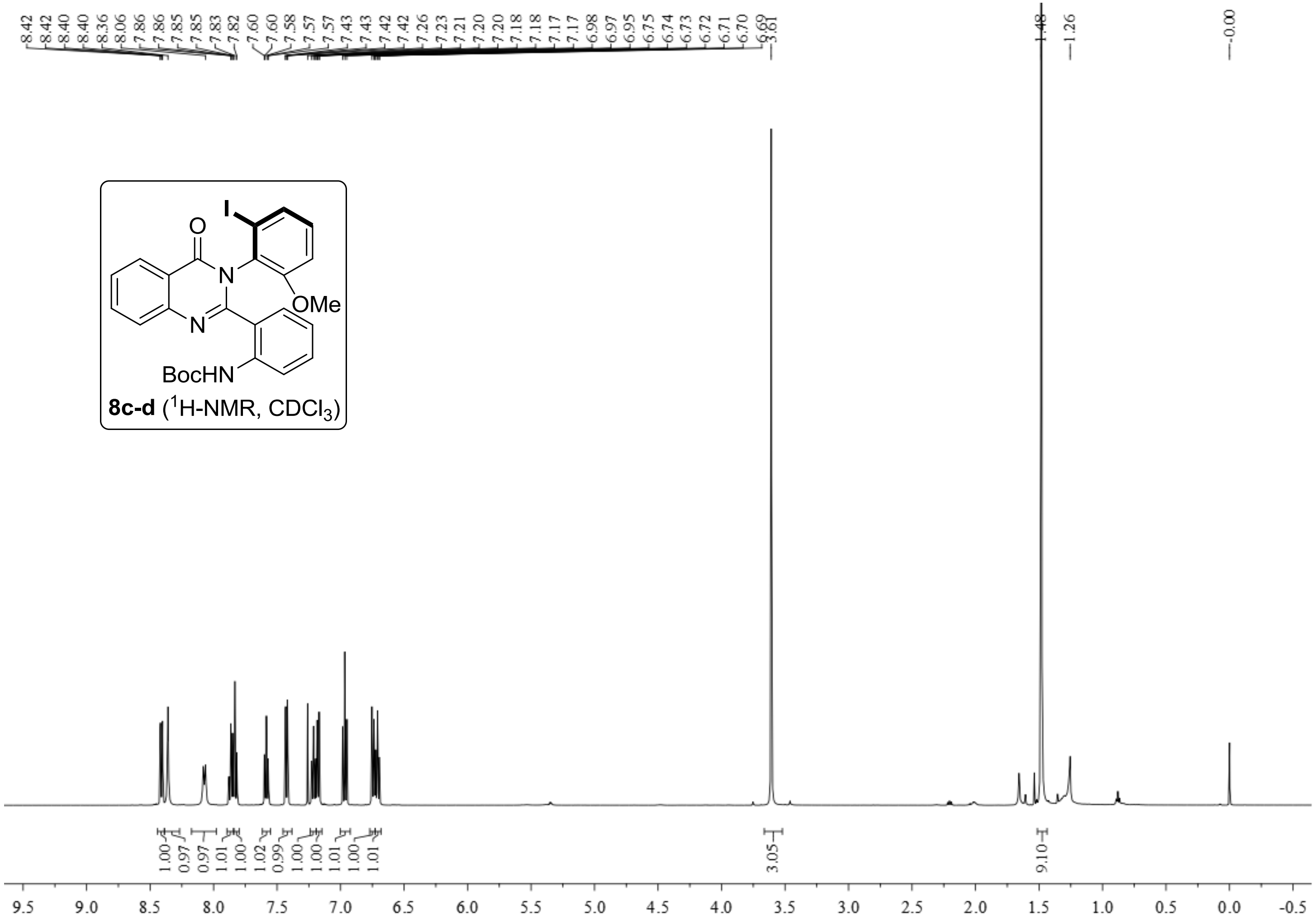


**Supplementary Figure 178.** $^{1}H$ NMR of **8c-d**

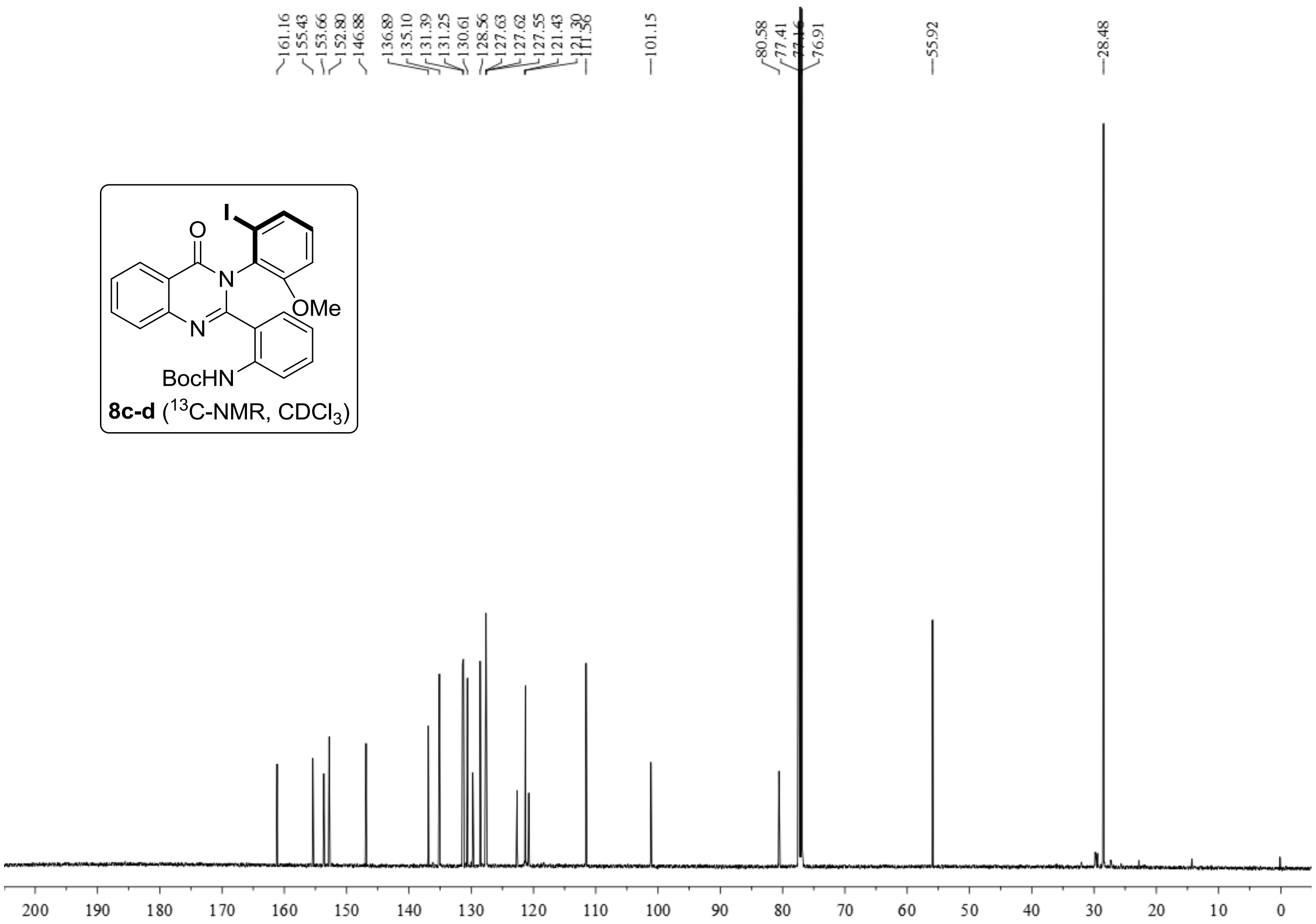


**Supplementary Figure 179.** $^{13}$C NMR of **8c-d**

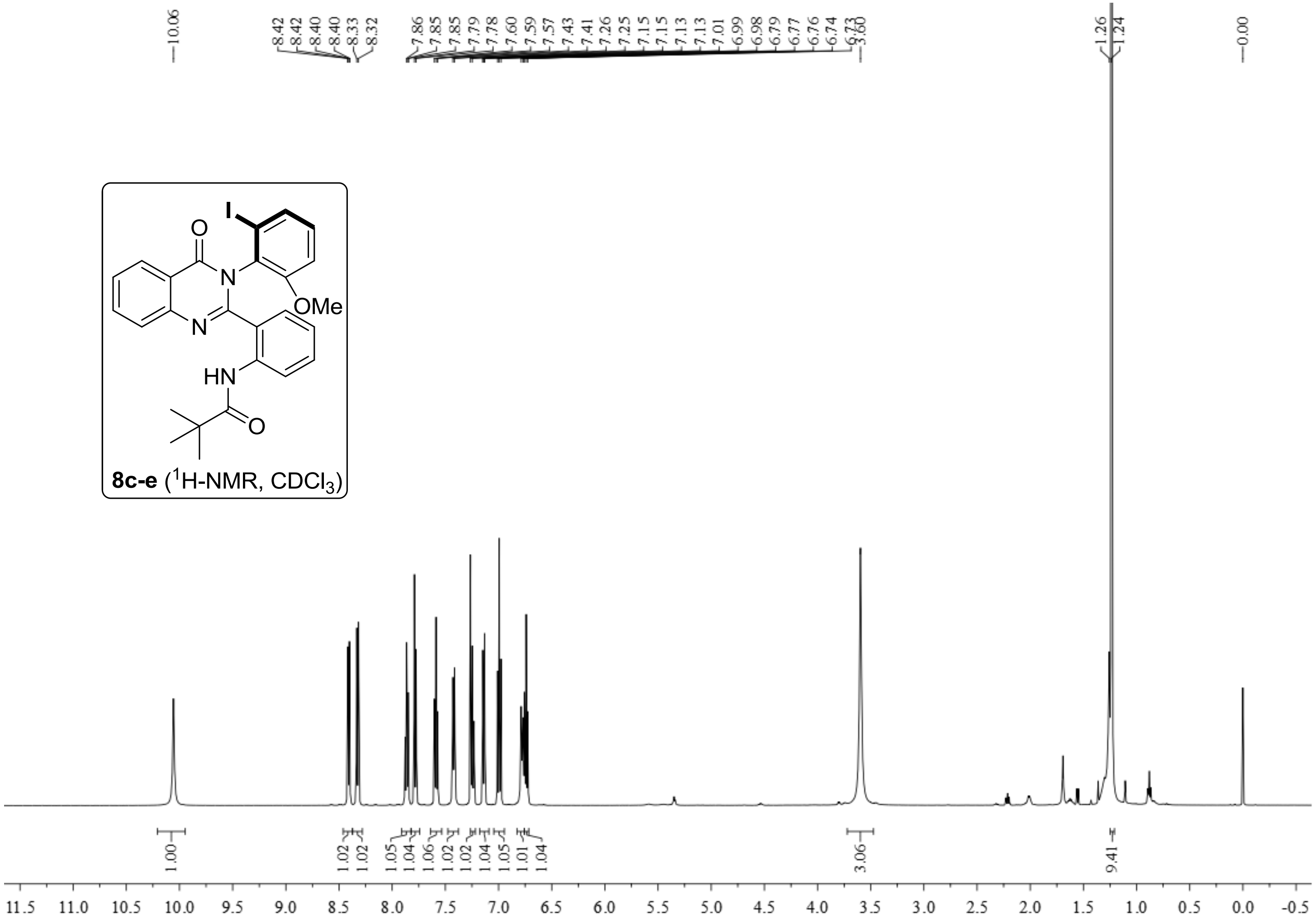


**Supplementary Figure 180.** $^{1}$H NMR of **8c-e**

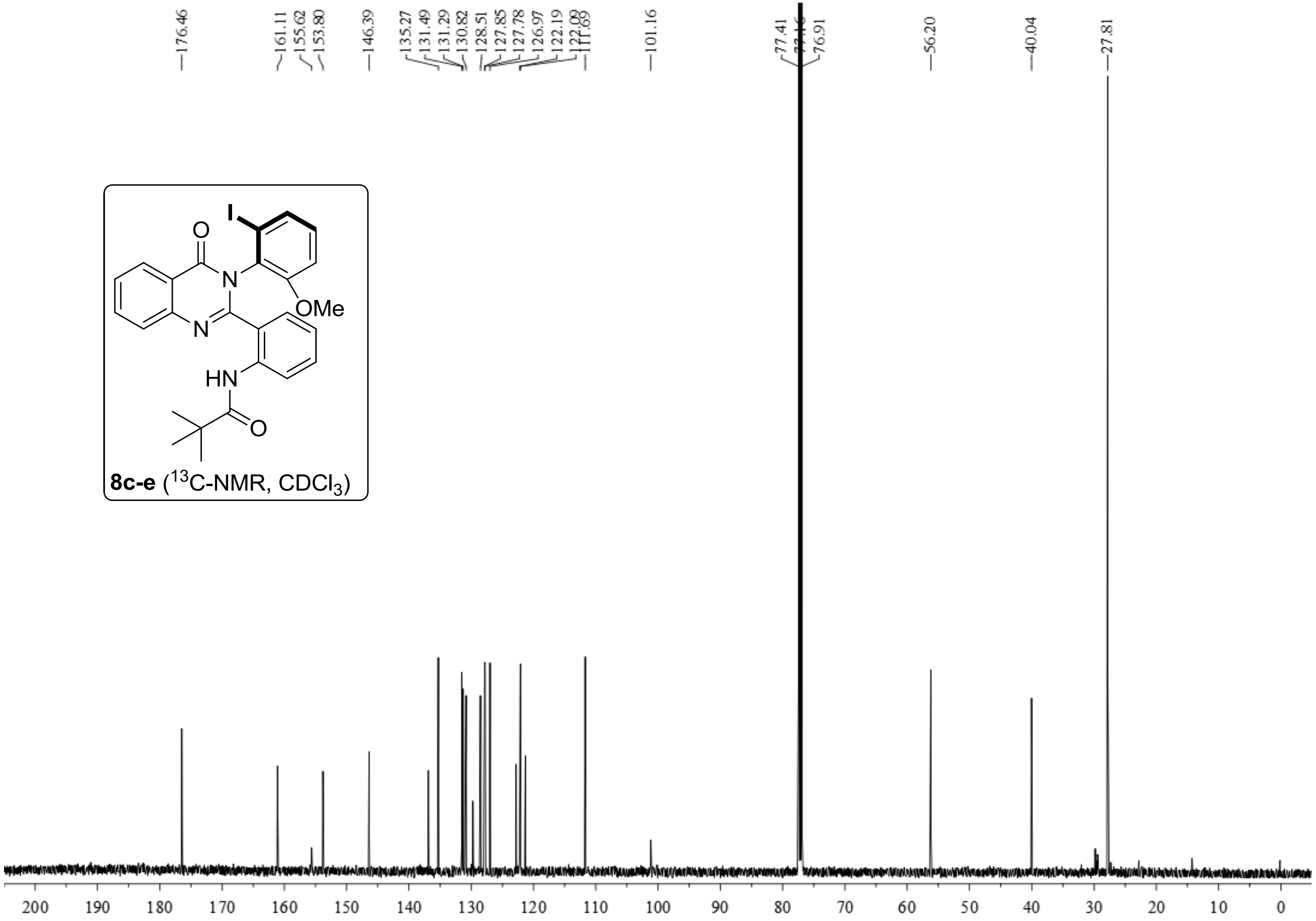


**Supplementary Figure 181.** $^{13}$C NMR of **8c-e**

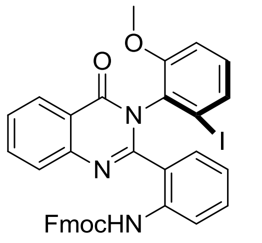


**8c** ($^{1}$H-NMR, $CDCl_3$)

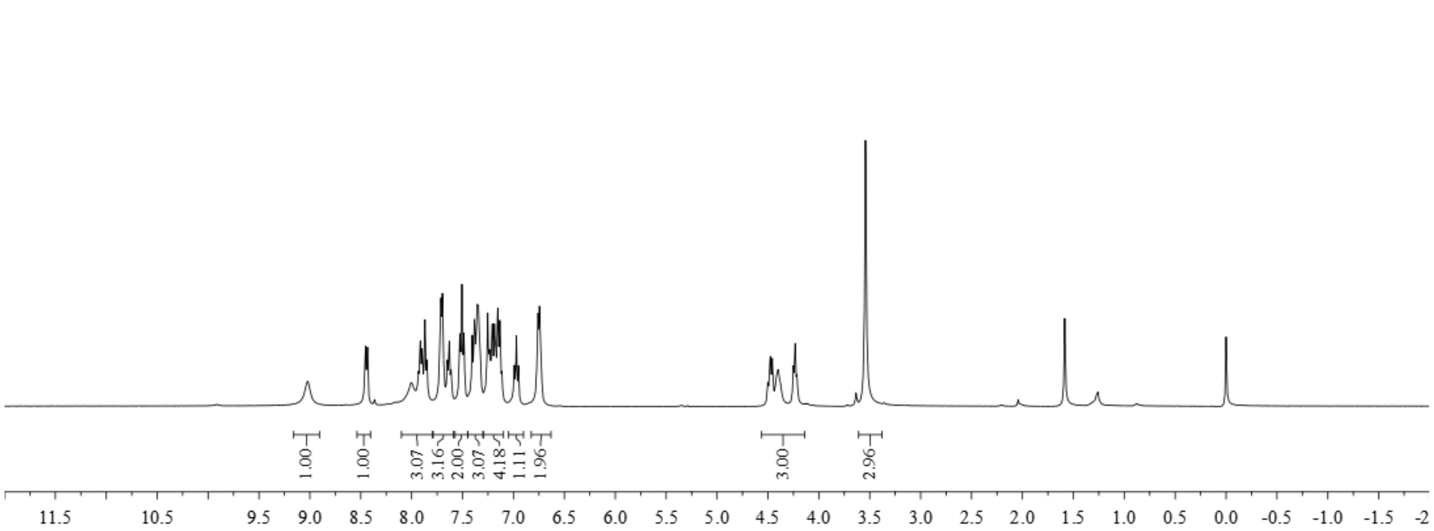

**Supplementary Figure 182.** $^1$H NMR of **8c**

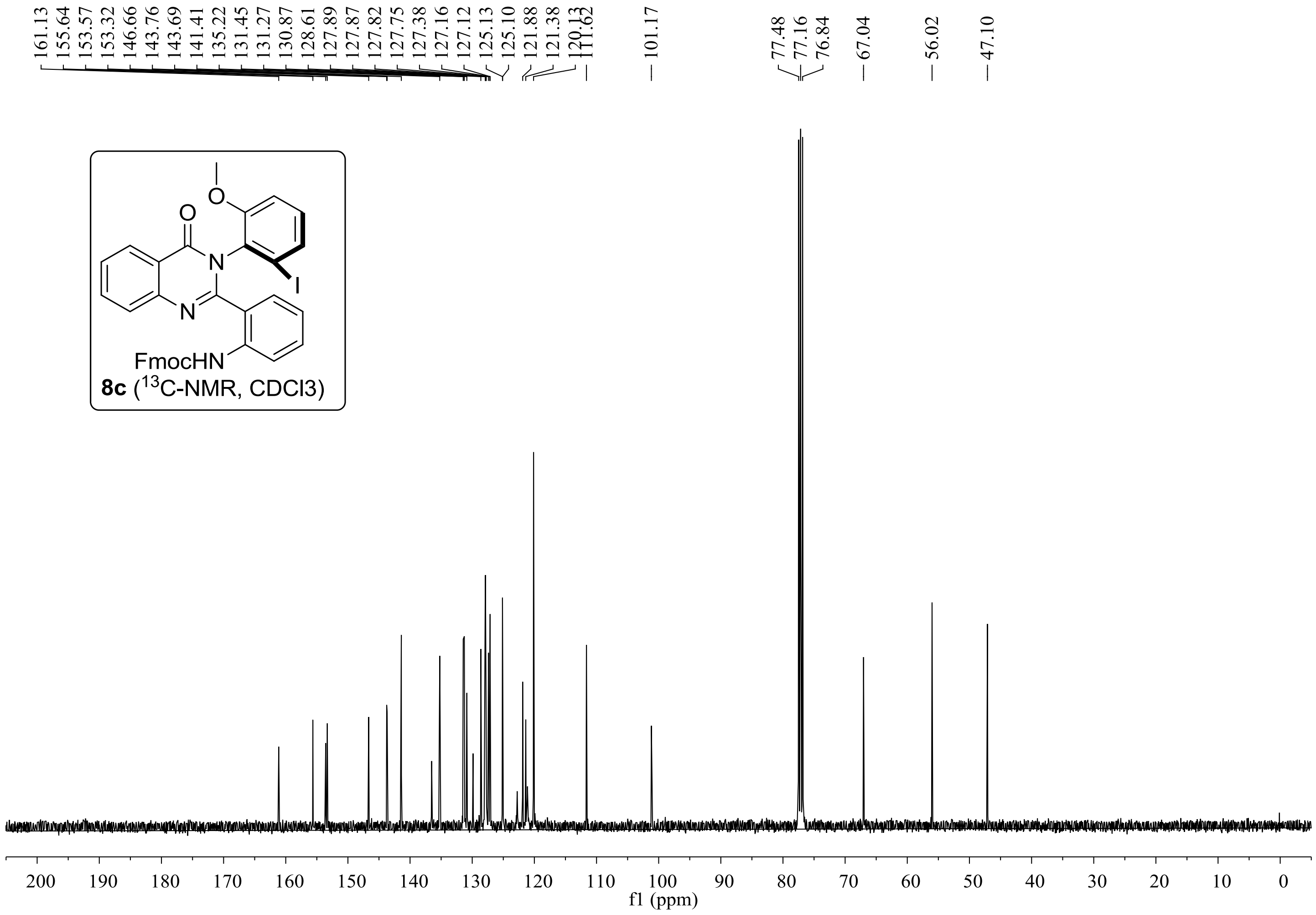


**Supplementary Figure 183.** $^{13}$C NMR of **8c**

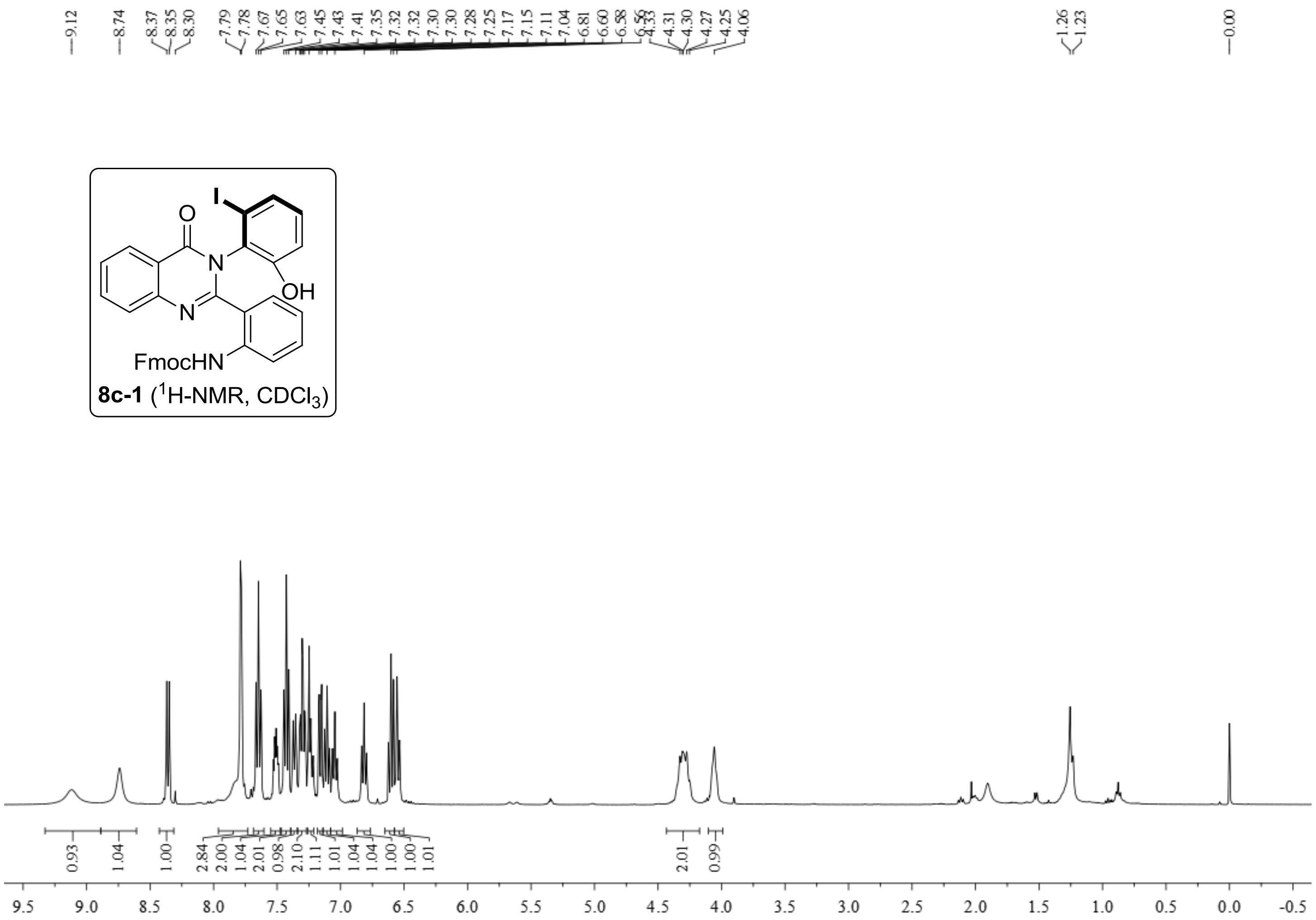


**Supplementary Figure 184.** $^1$H NMR of **8c-1**

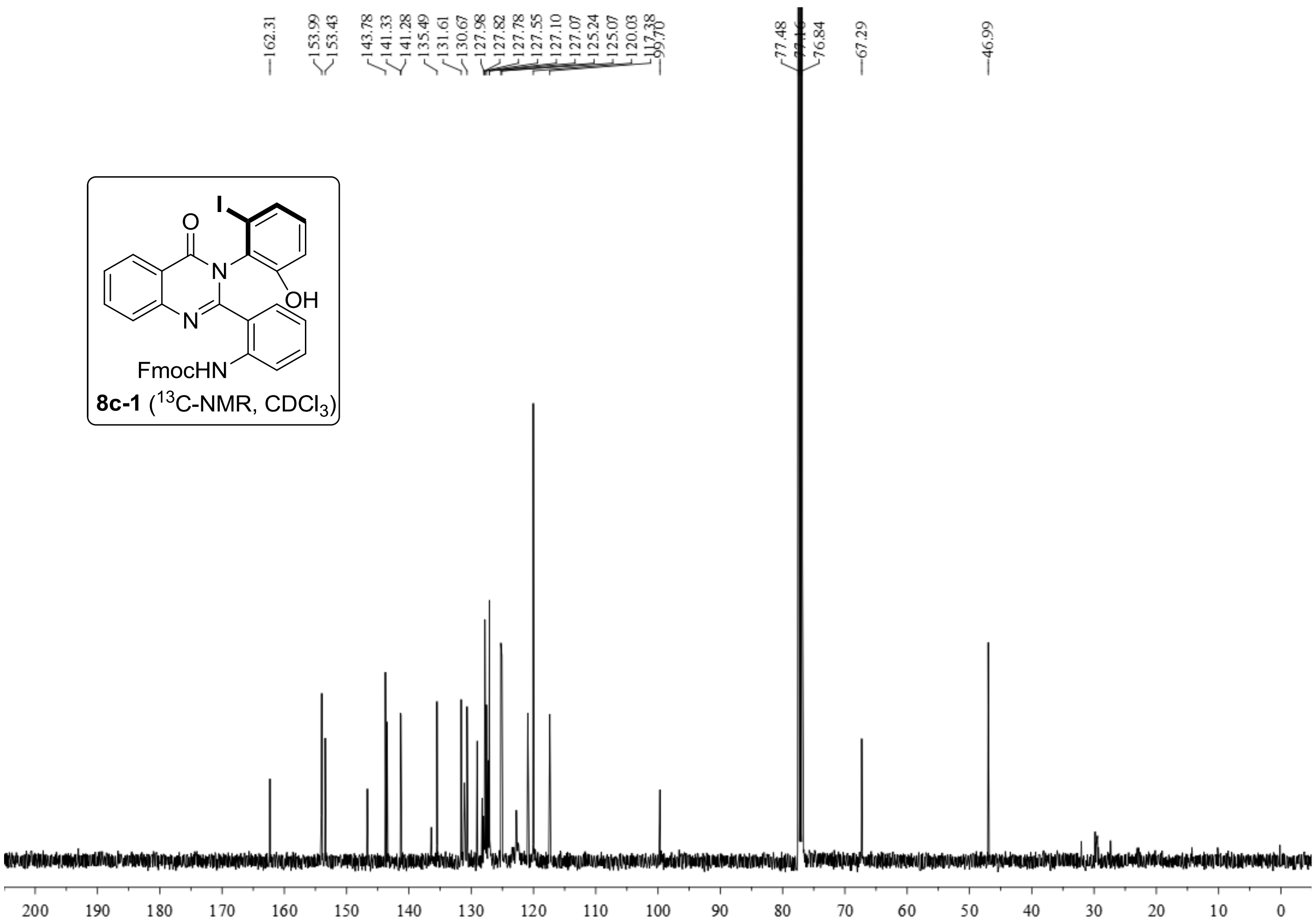


**Supplementary Figure 185.** $^{13}$C NMR of **8c-1**

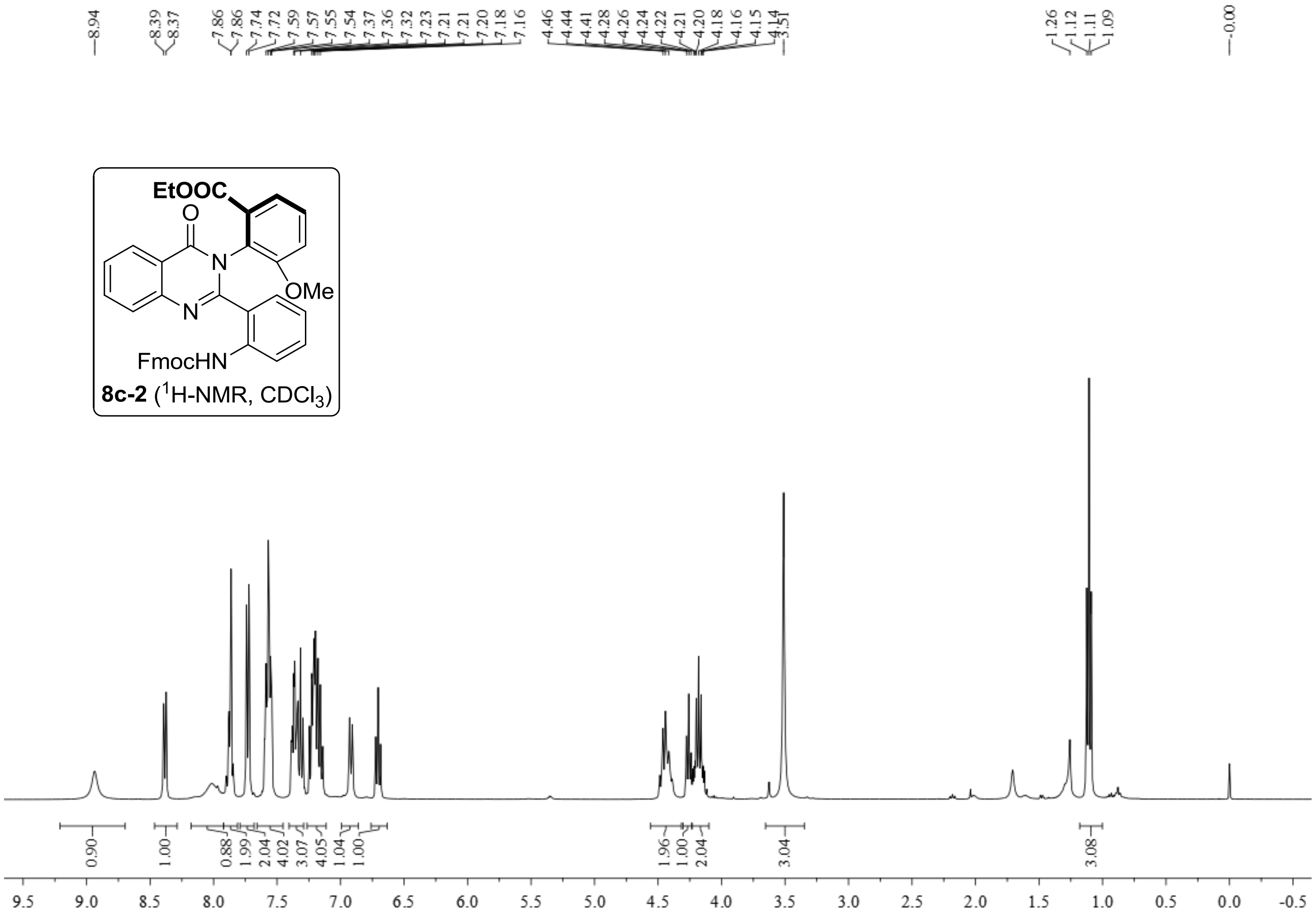


**Supplementary Figure 186.** $^1$H NMR of **8c-2**

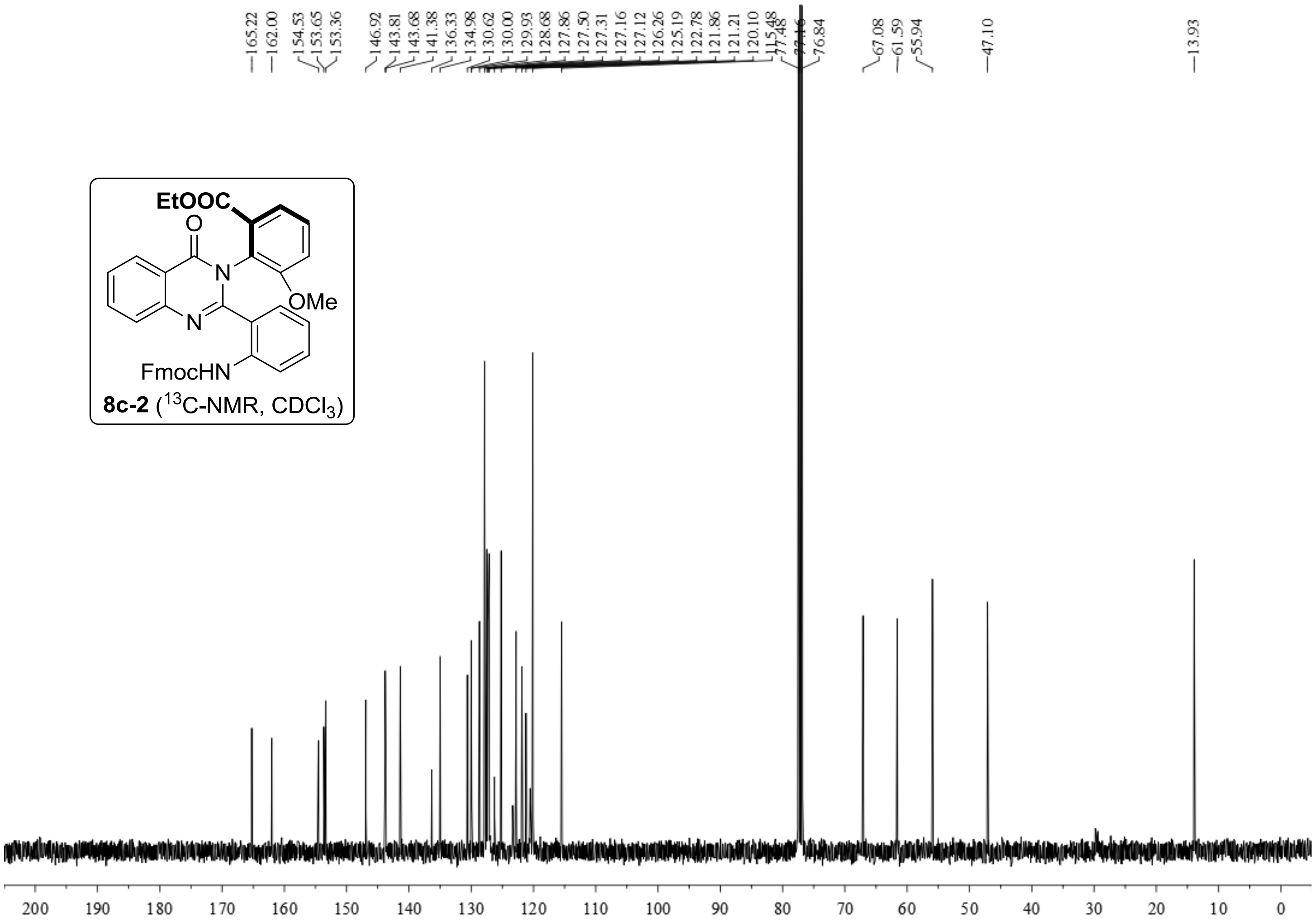


**Supplementary Figure 187.** $^{13}$C NMR of **8c-2**

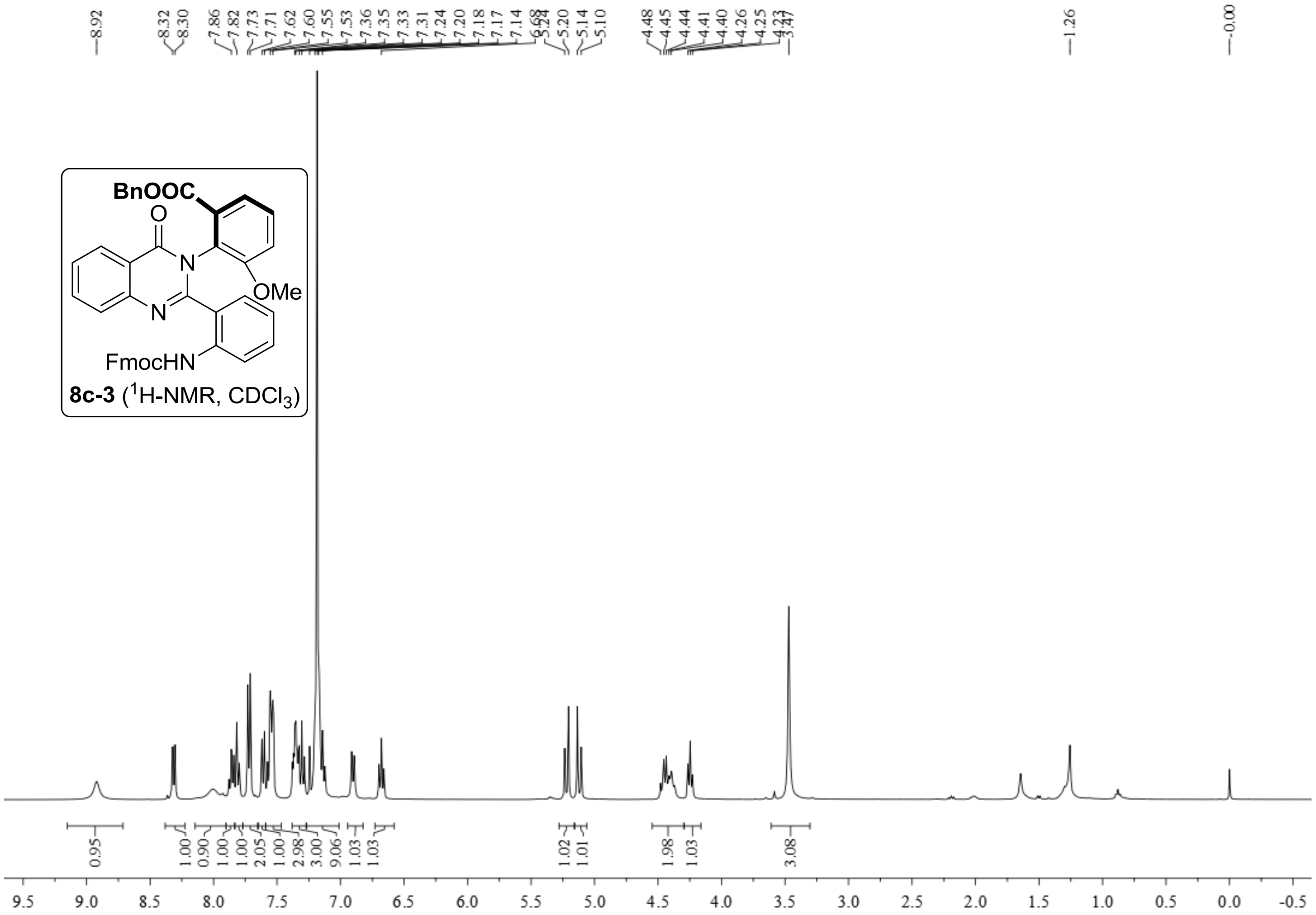


**Supplementary Figure 188.** $^1$H NMR of **8c-3**

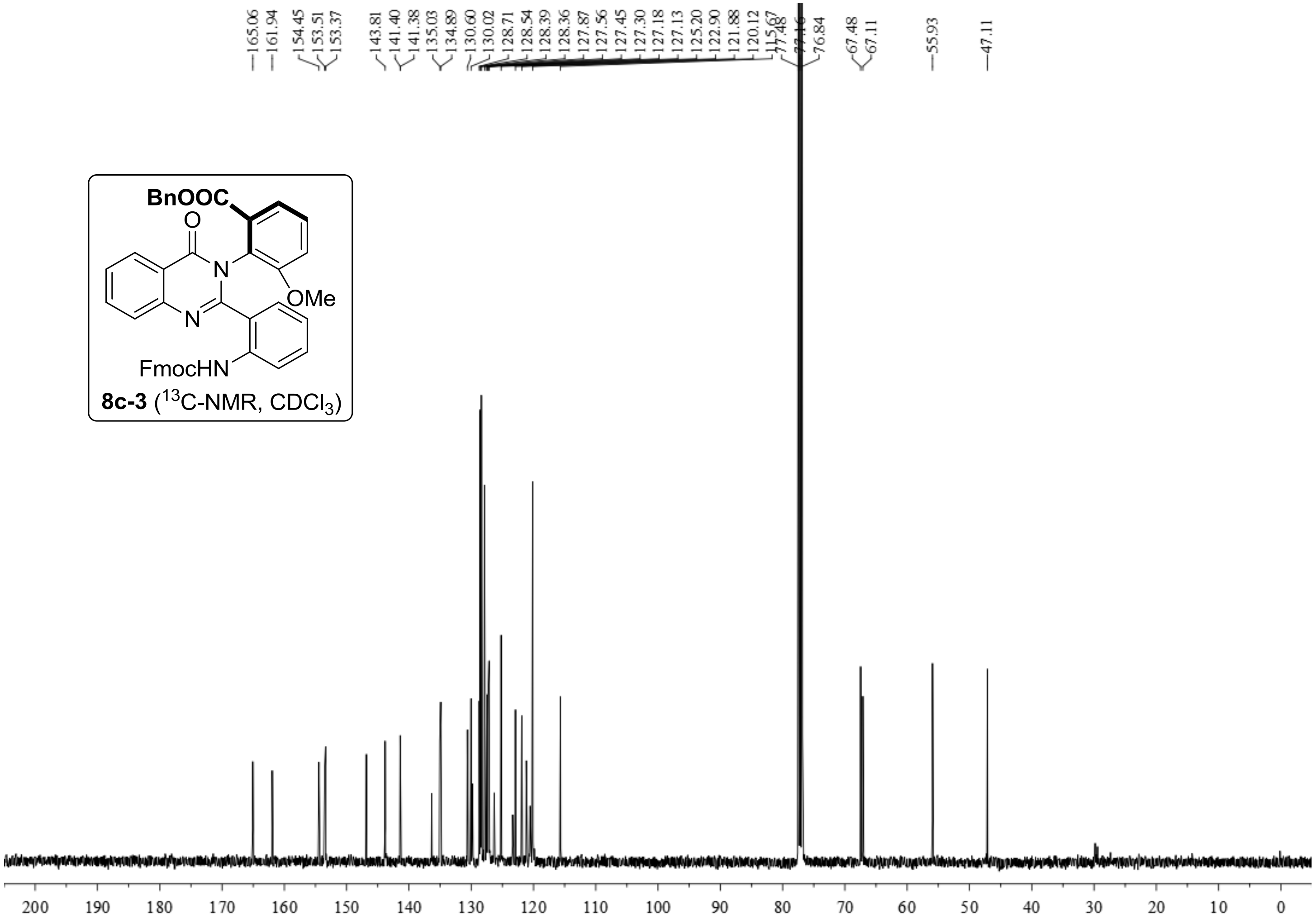


**Supplementary Figure 189.** $^{13}$C NMR of **8c-3**

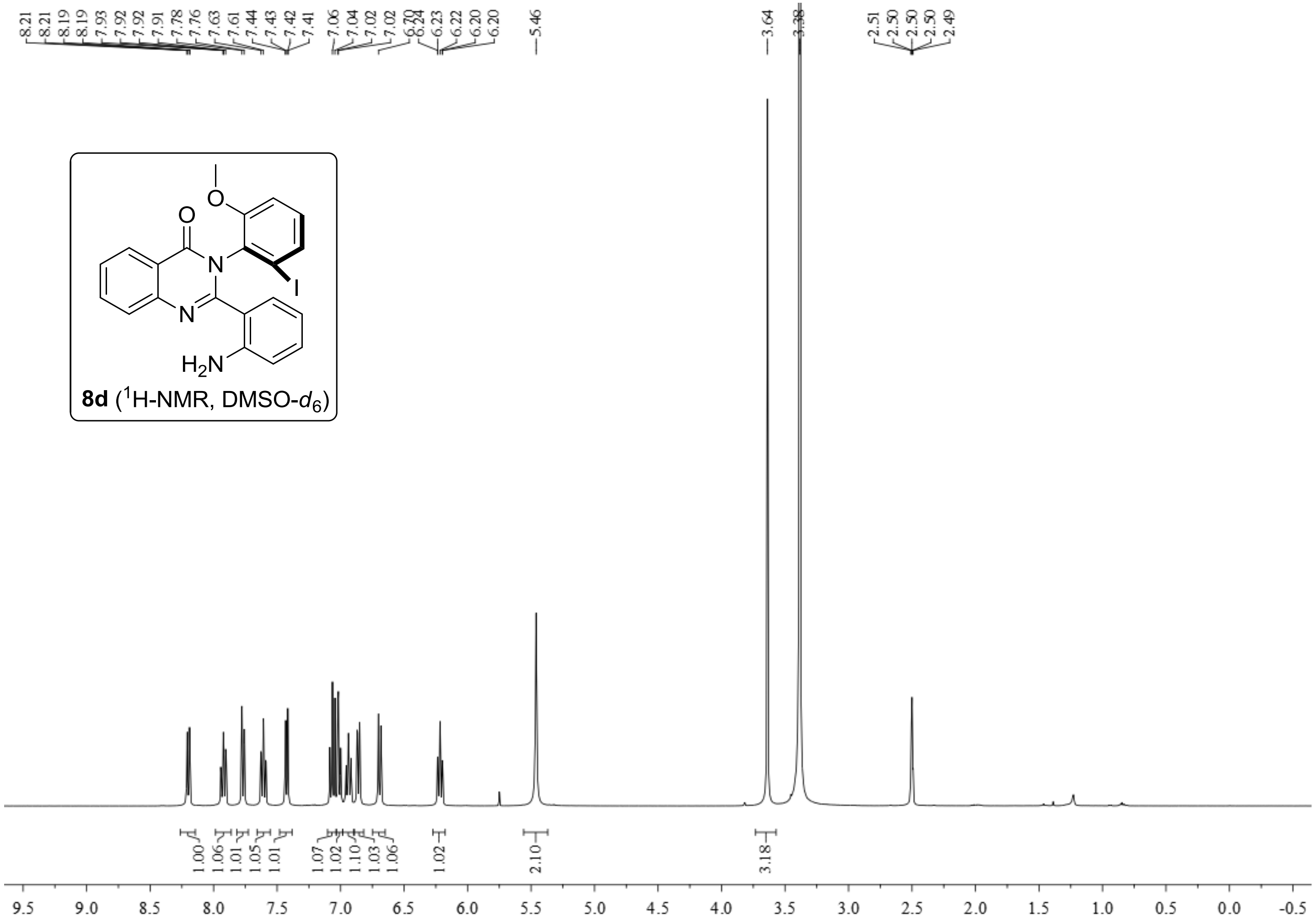


**Supplementary Figure 190.** $^1$H NMR of **8d**

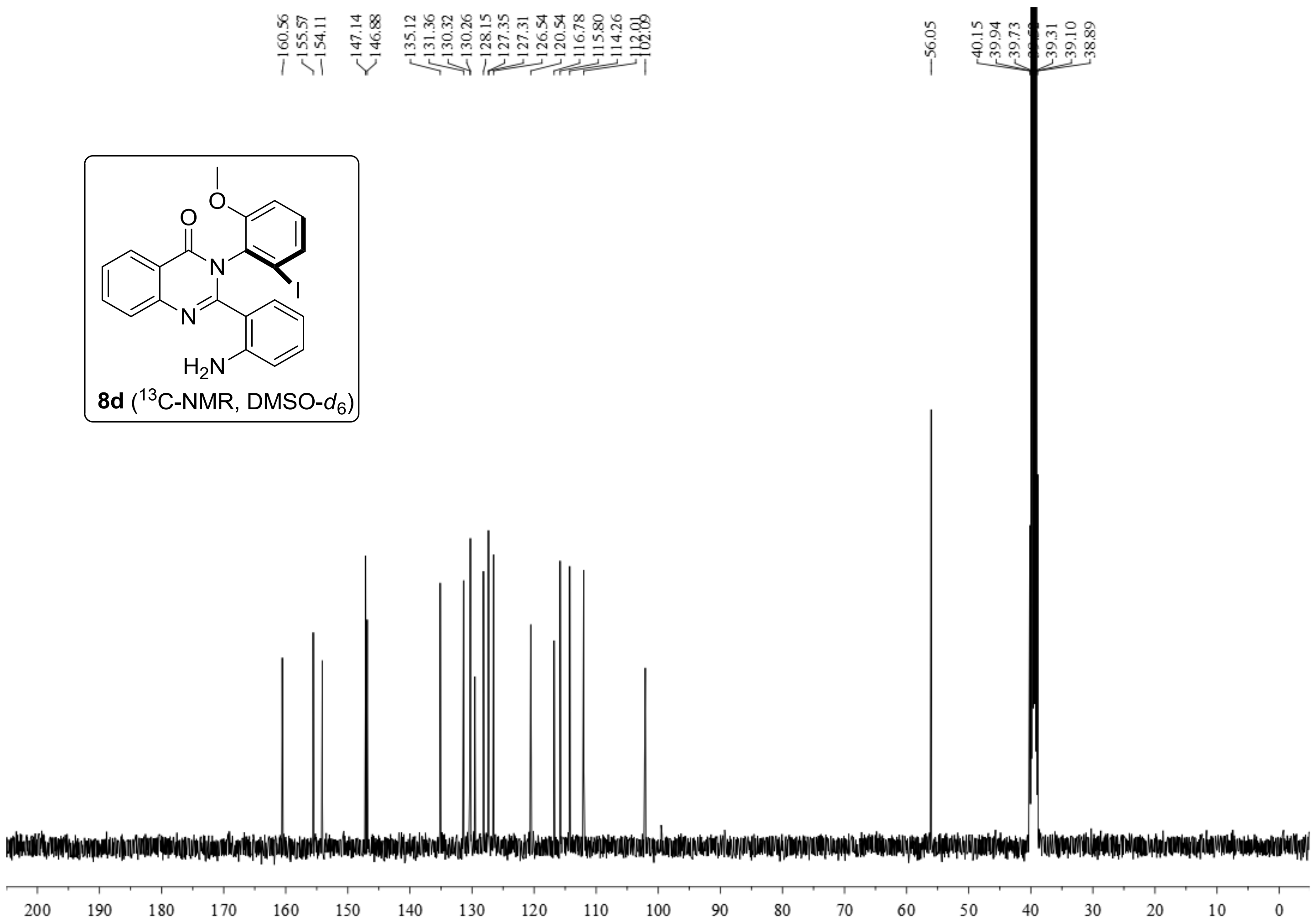


**Supplementary Figure 191.** $^{13}$C NMR of **8d**

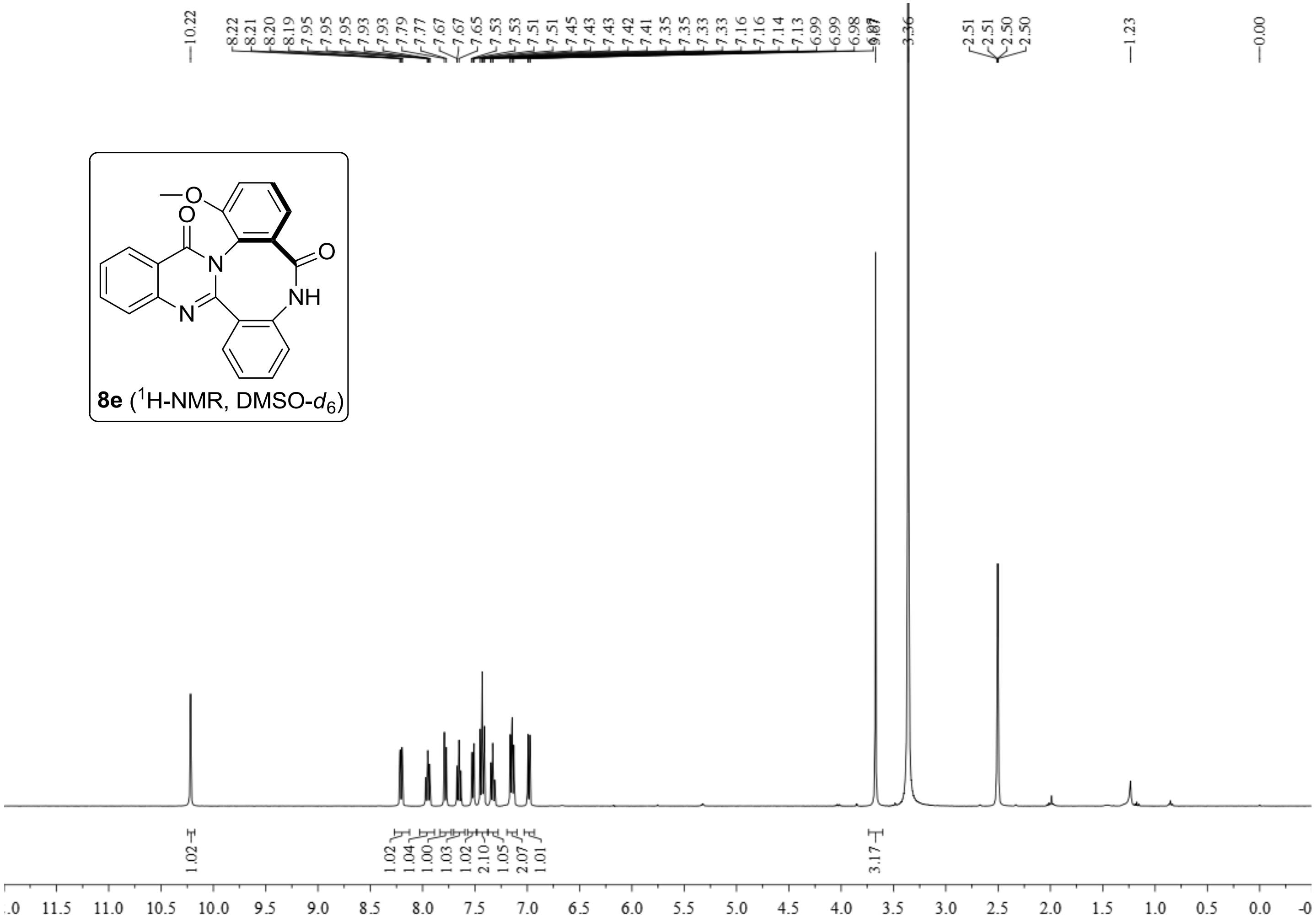


**Supplementary Figure 192.** $^1$H NMR of **8e**

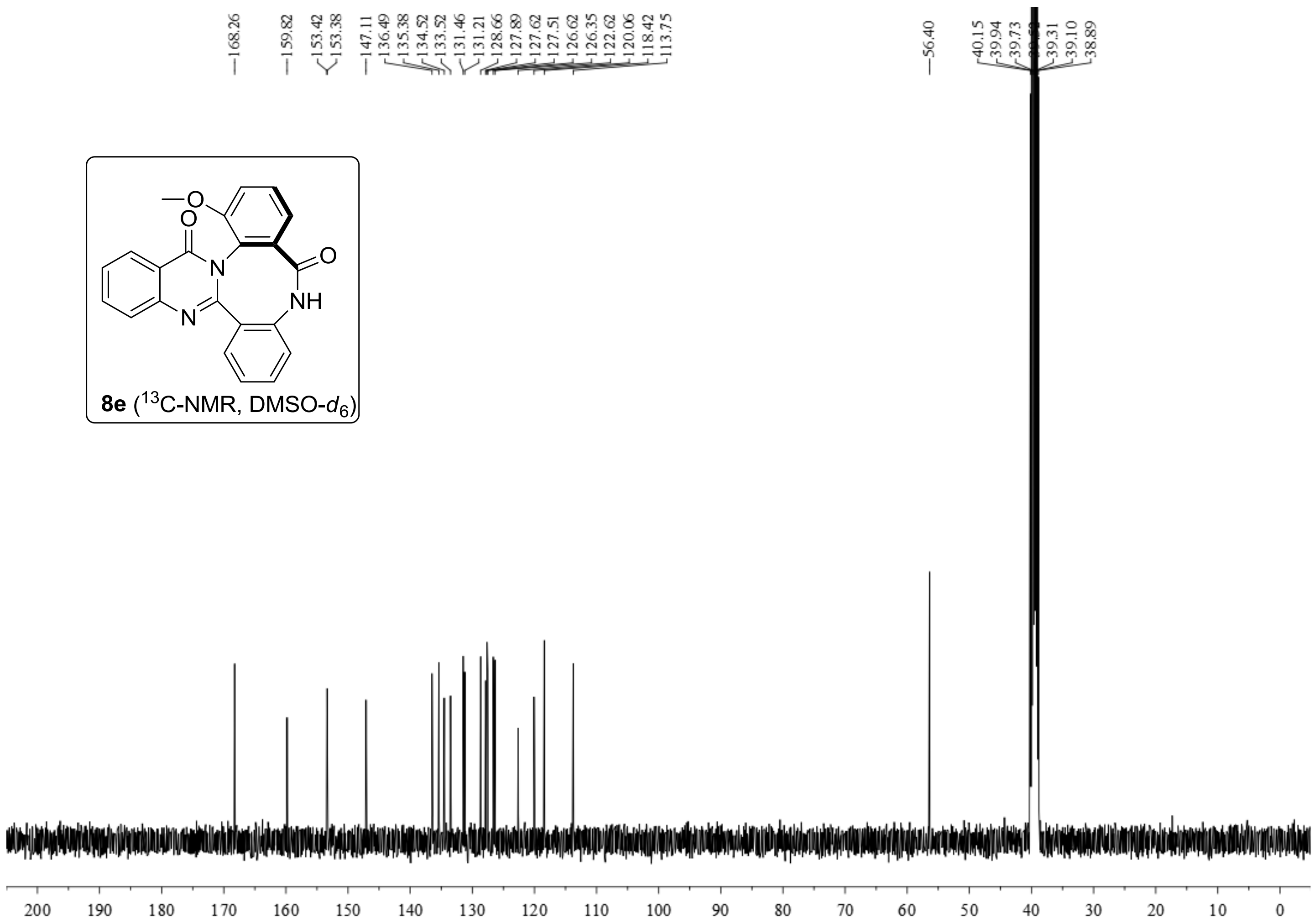


**Supplementary Figure 193.** $^{13}$C NMR of **8e**

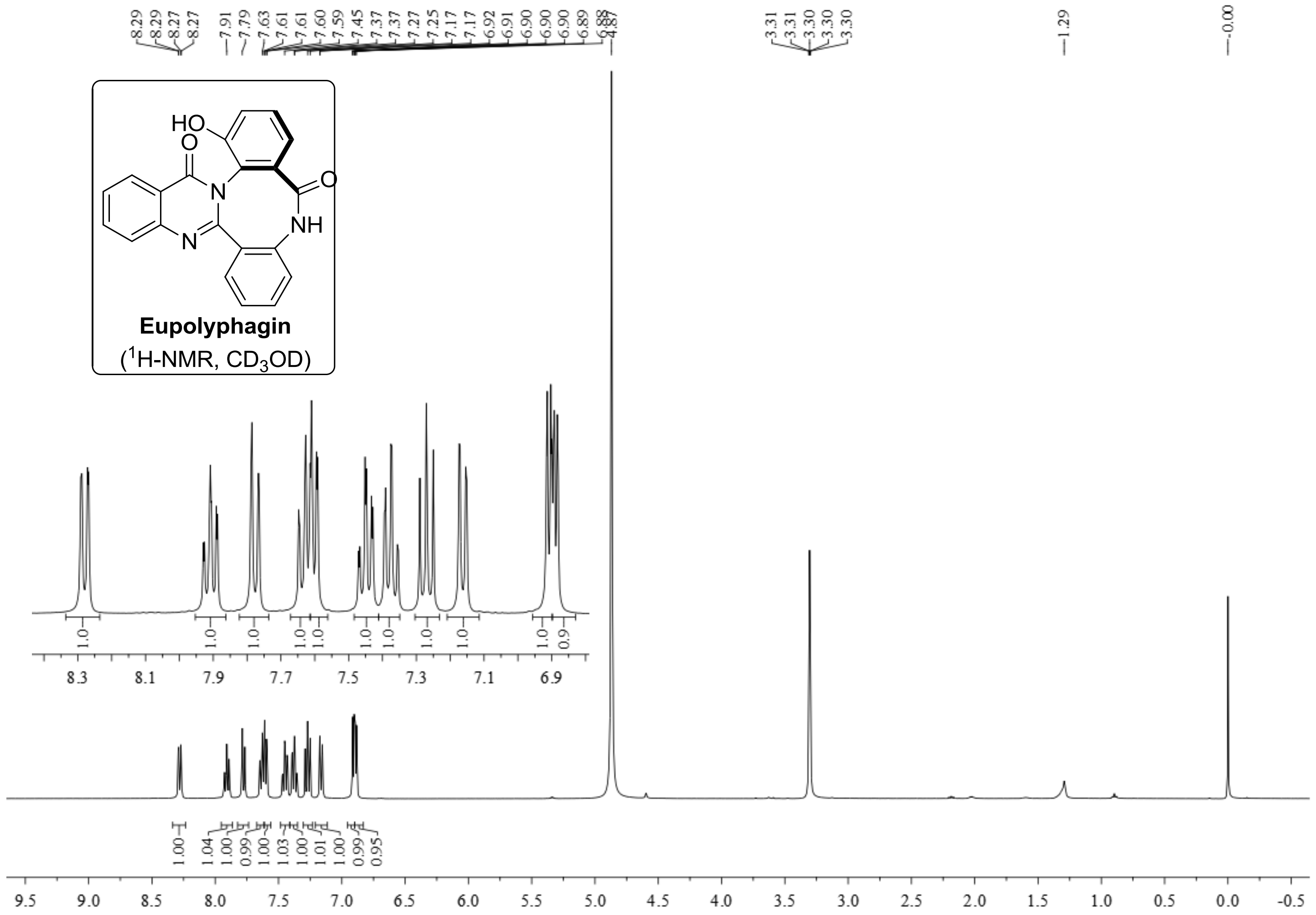


**Supplementary Figure 194.** $^1$H NMR of **Eupolyphagin**

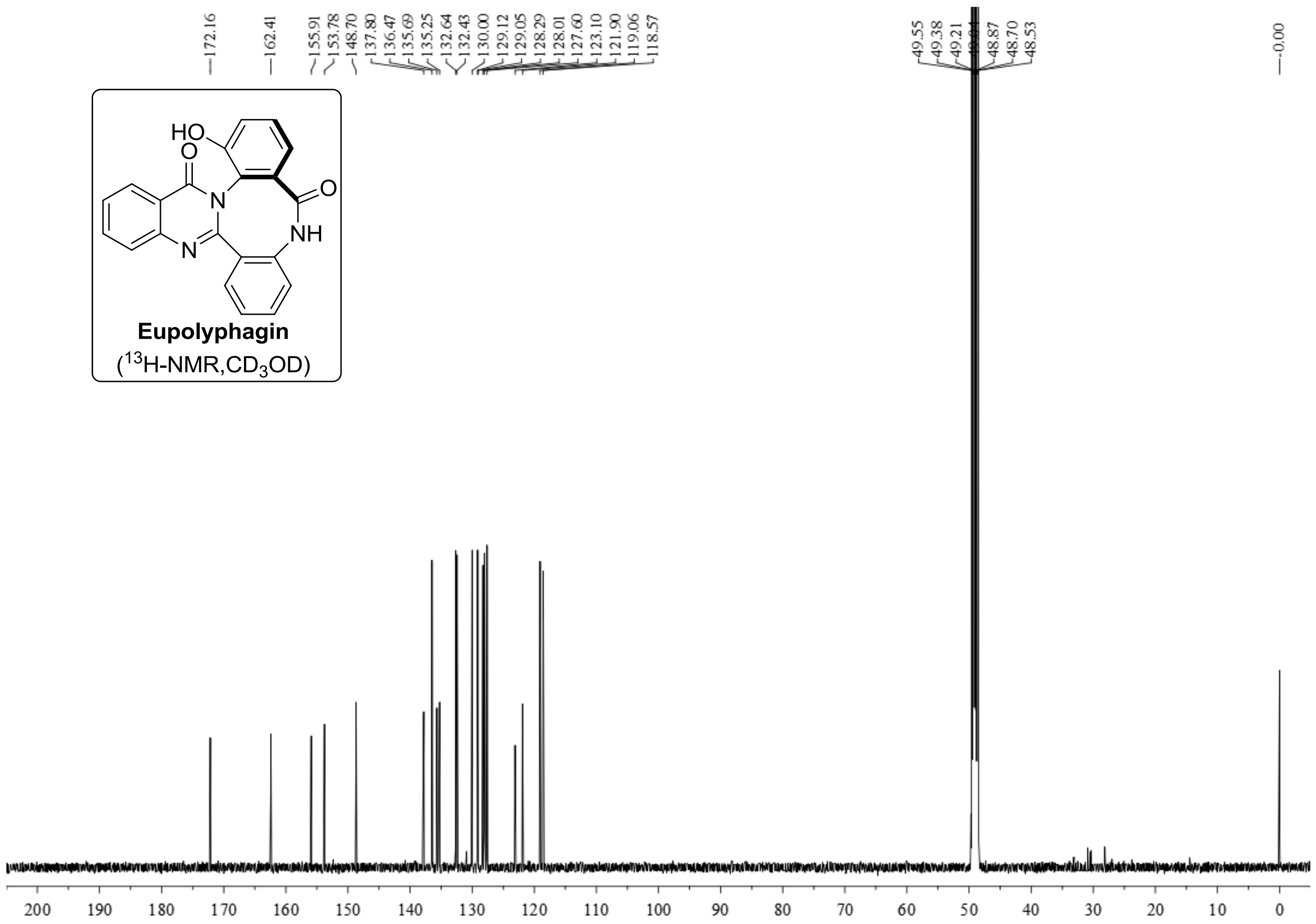


**Supplementary Figure 195.** $^{13}$C NMR of **Eupolyphagin**

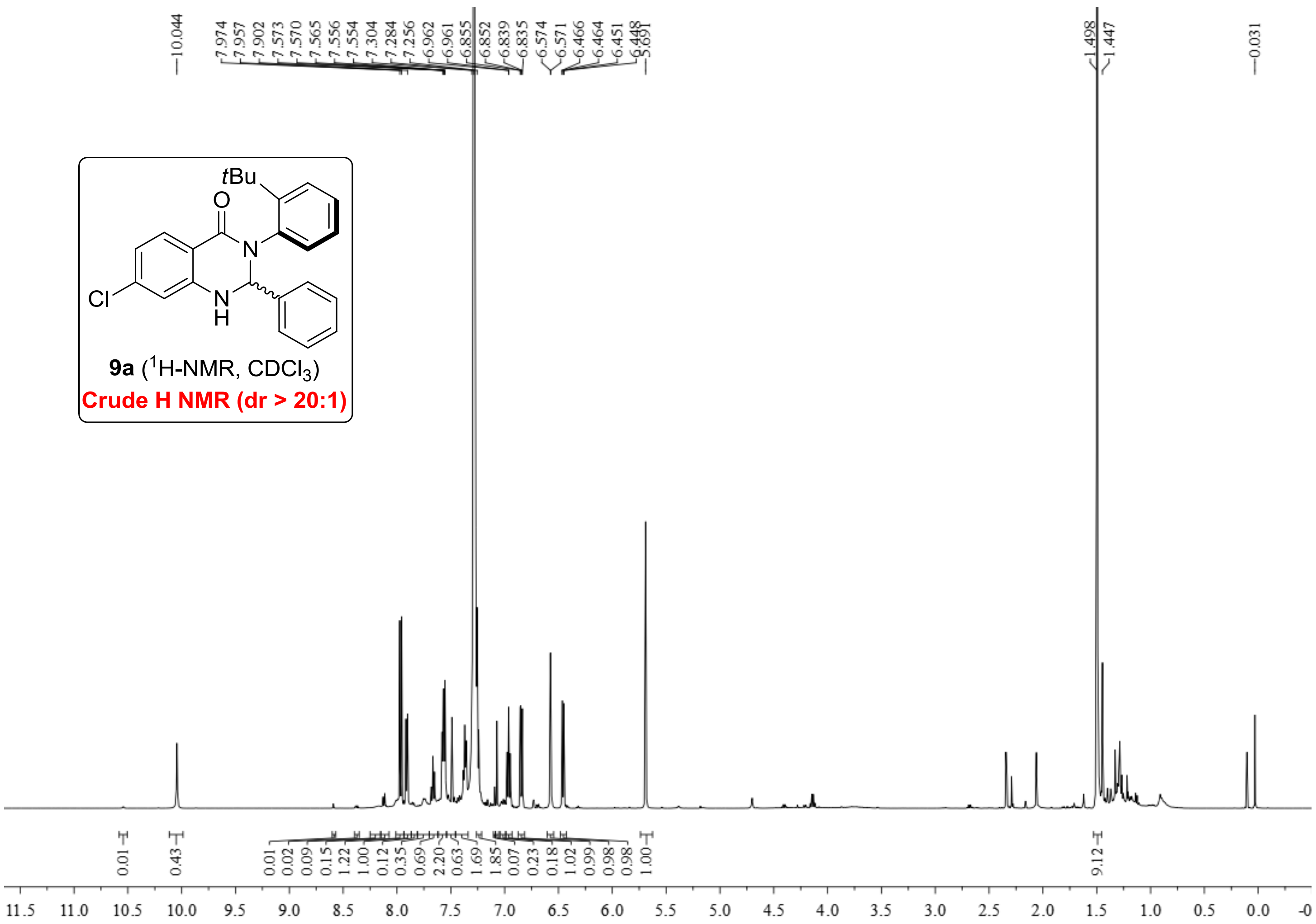


**Supplementary Figure 196.** Crude $^1$H NMR of **9a**

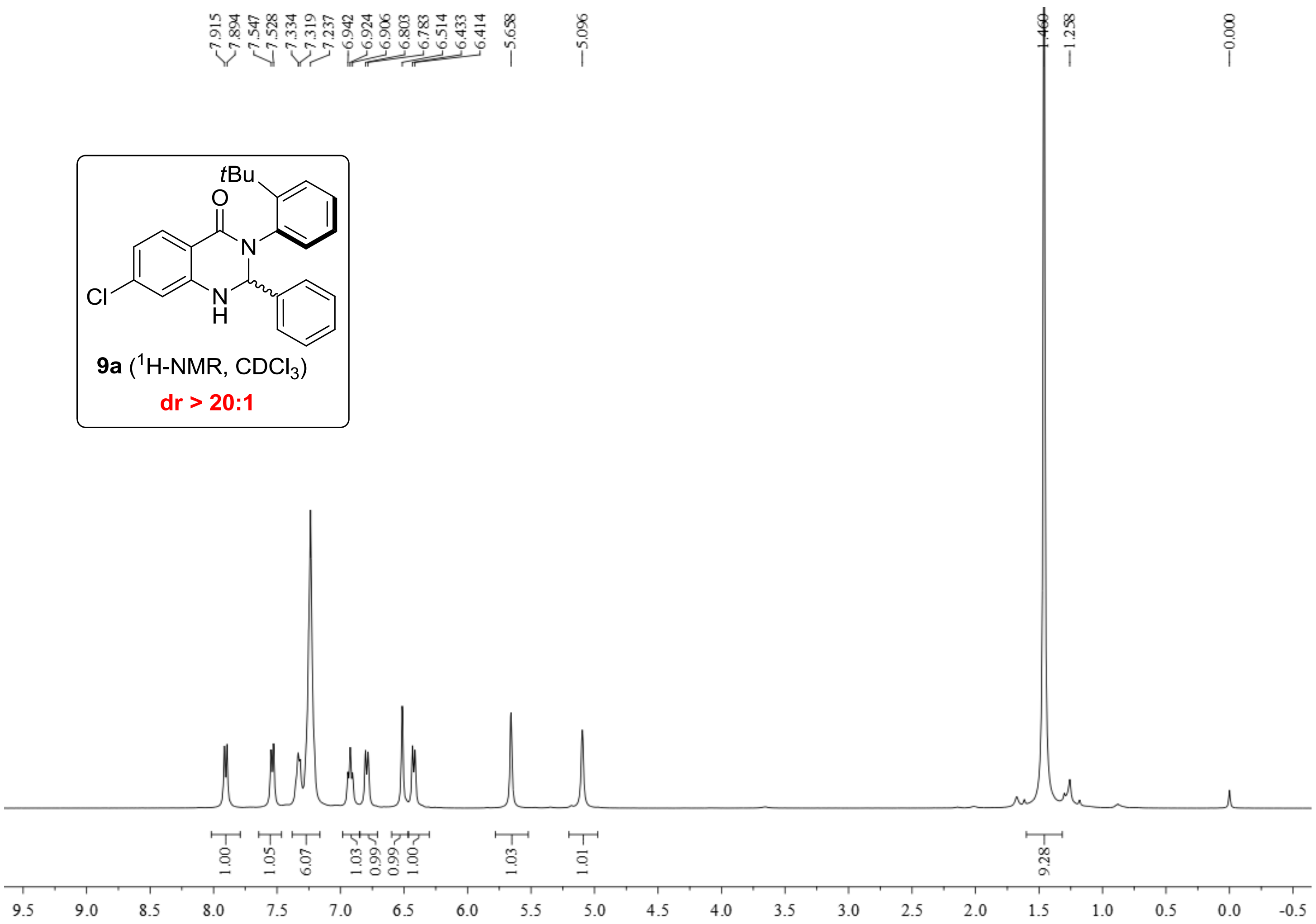


**Supplementary Figure 197.** $^1$H NMR of **9a**

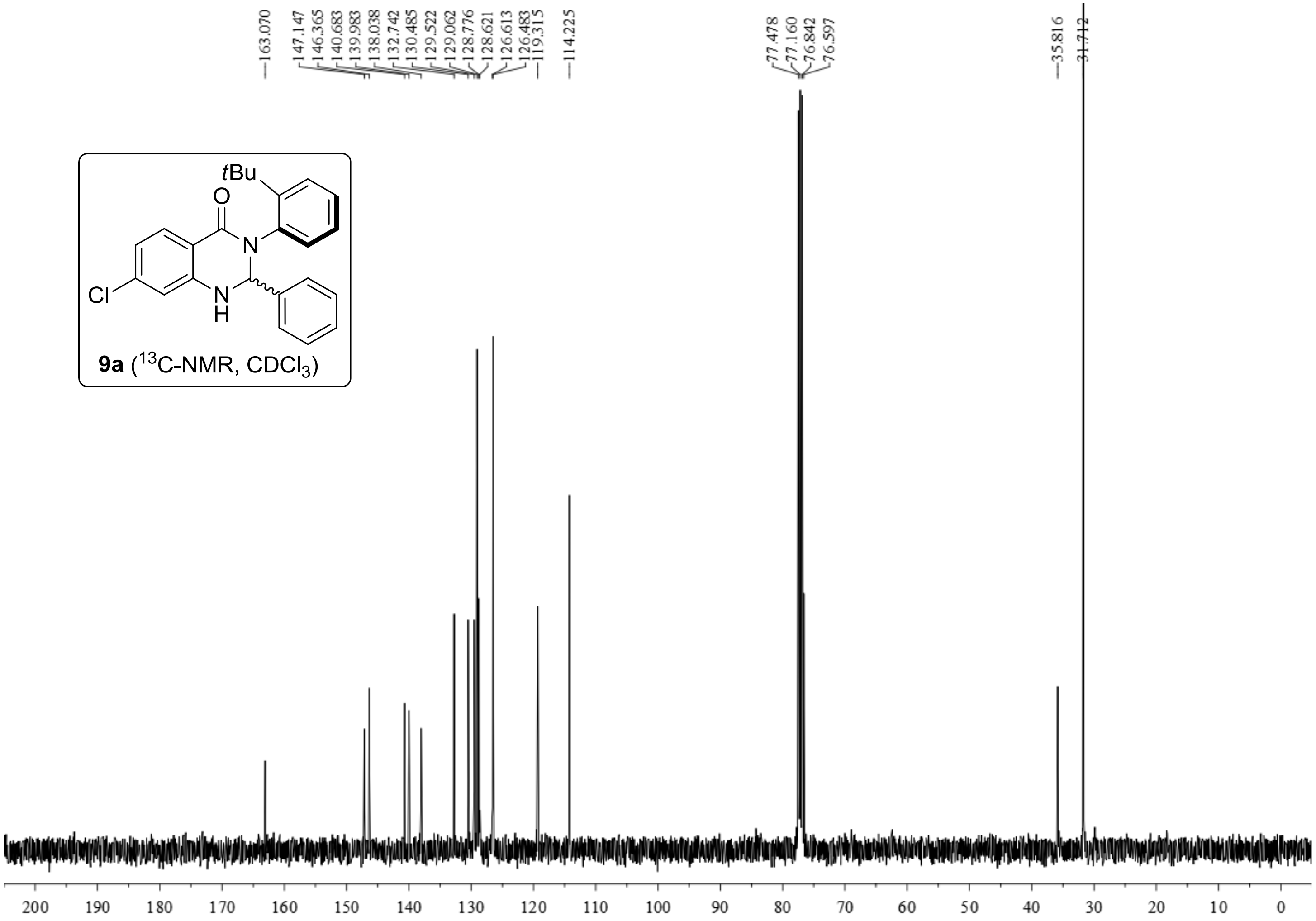


**Supplementary Figure 198.** $^{13}$C NMR of **9a**

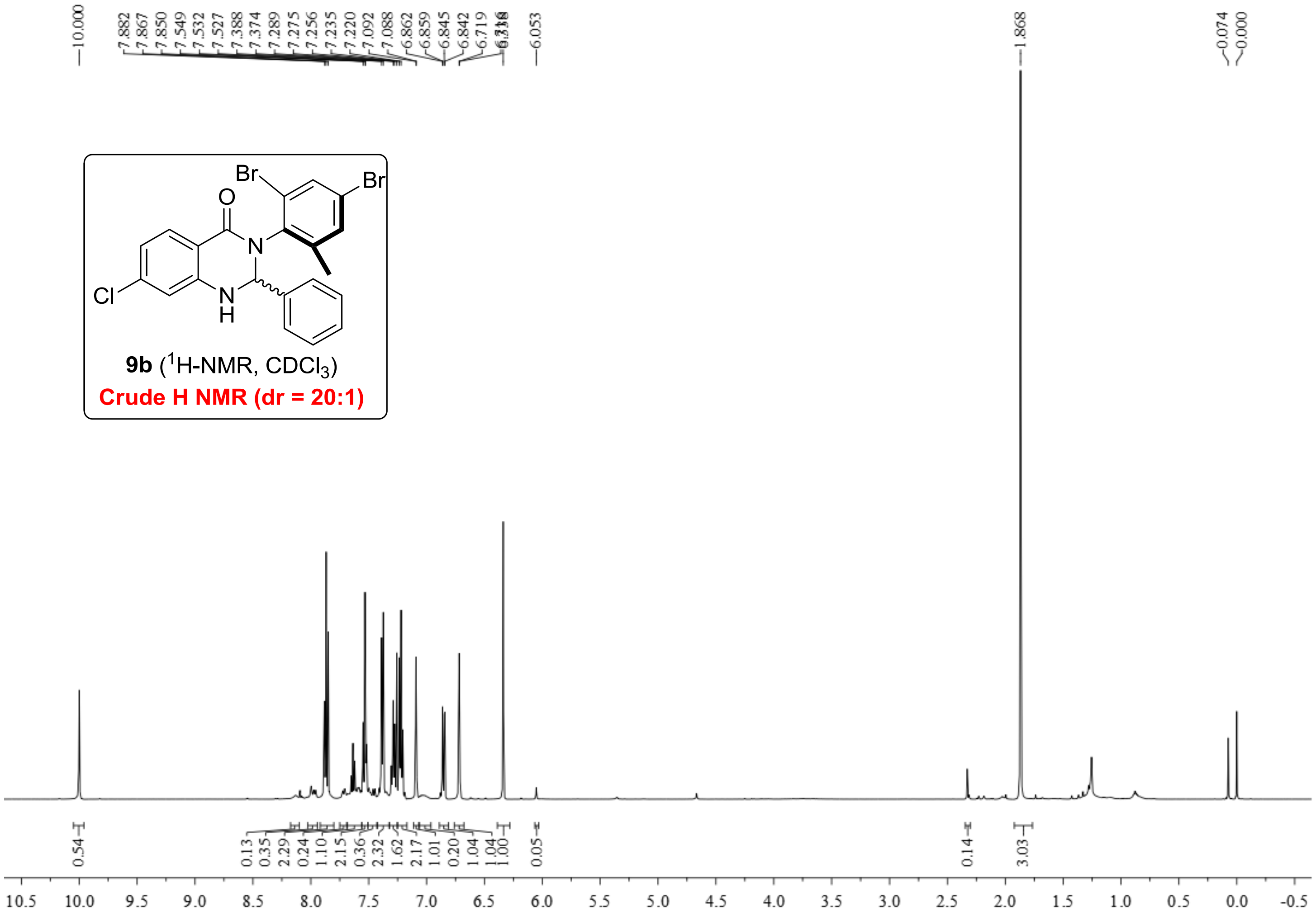


**Supplementary Figure 199.** Crude $^1$H NMR of **9b**

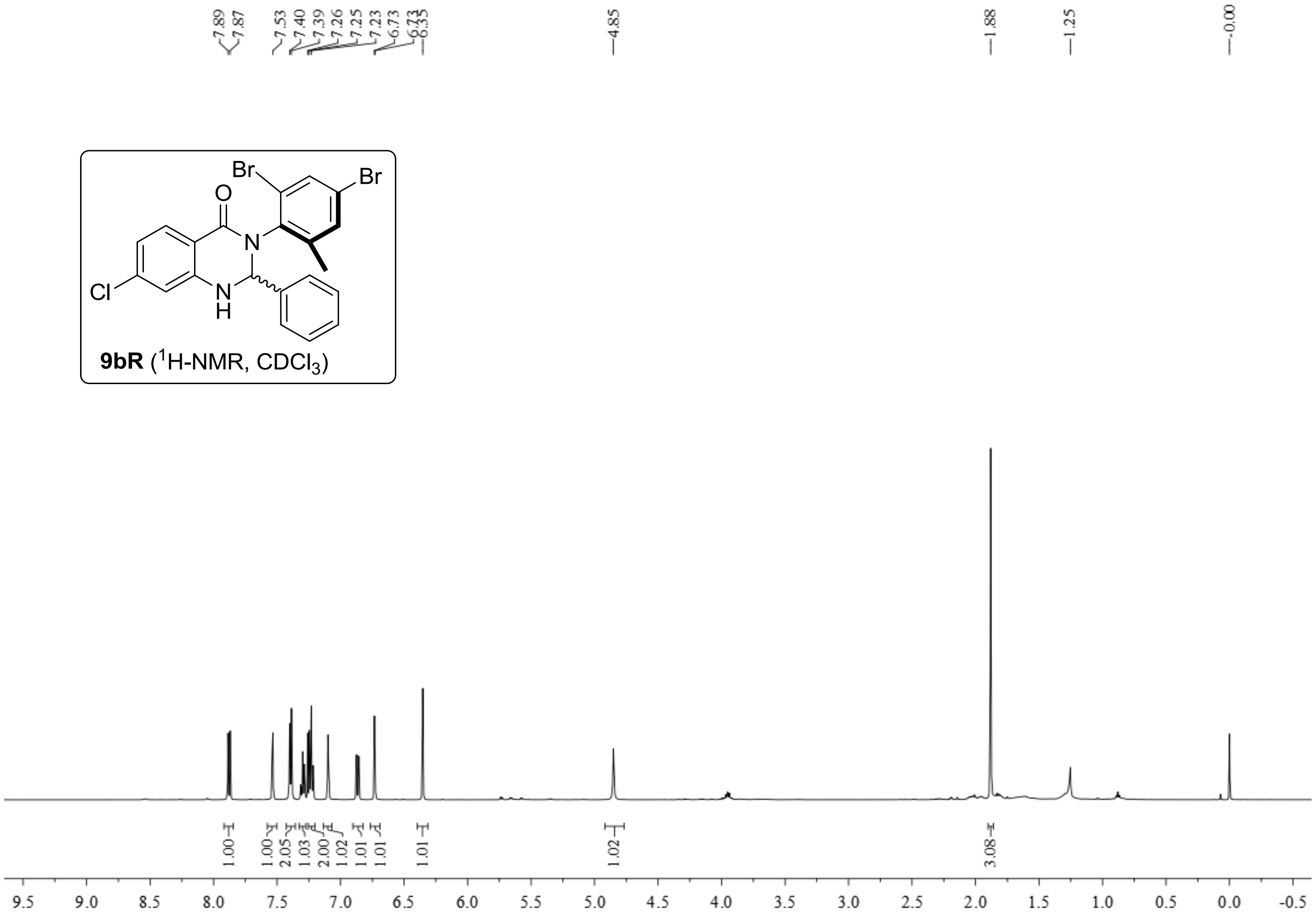


**Supplementary Figure 200.** $^1$H NMR of **9bR**

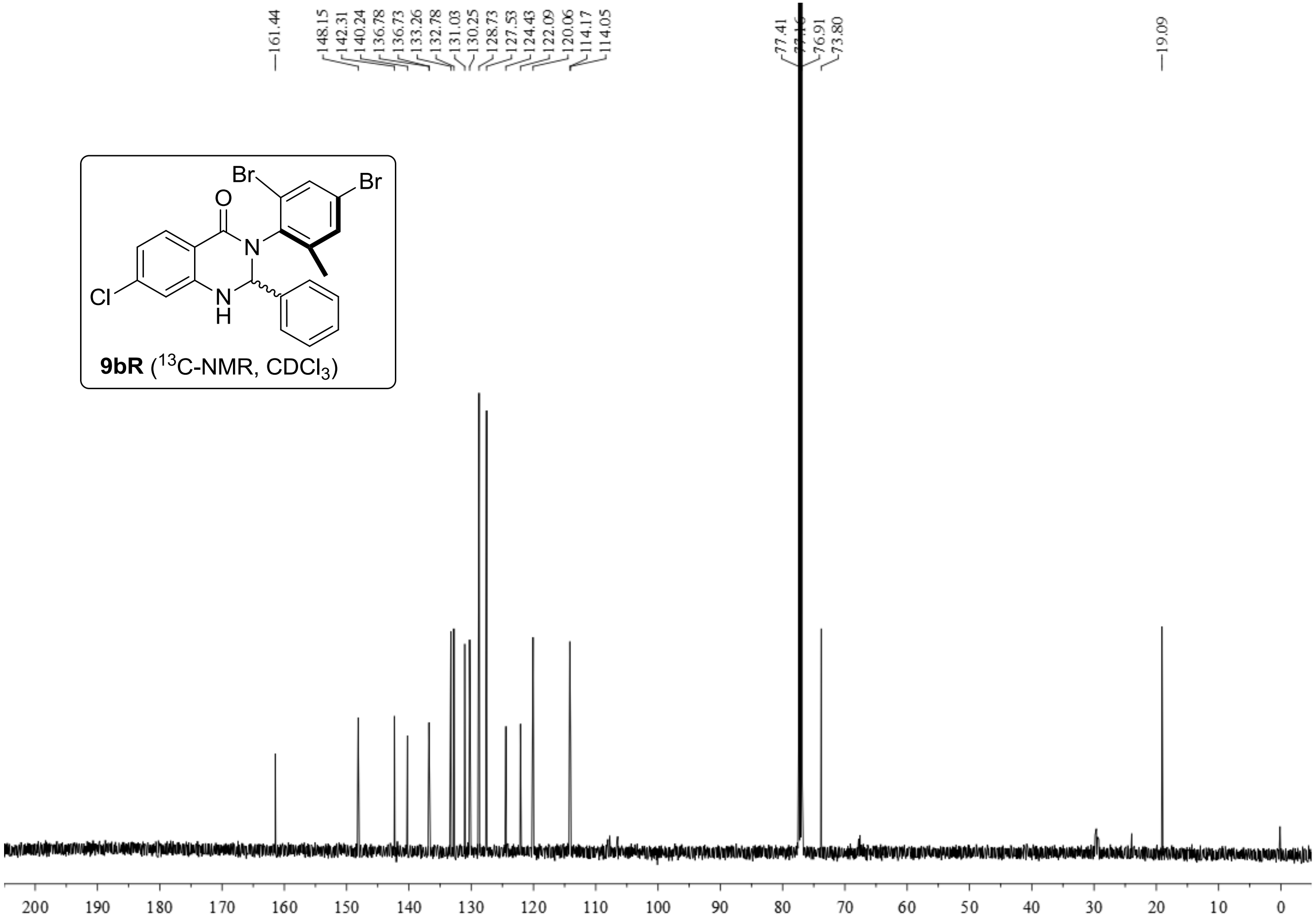


**Supplementary Figure 201.** $^{13}$C NMR of **9bR**

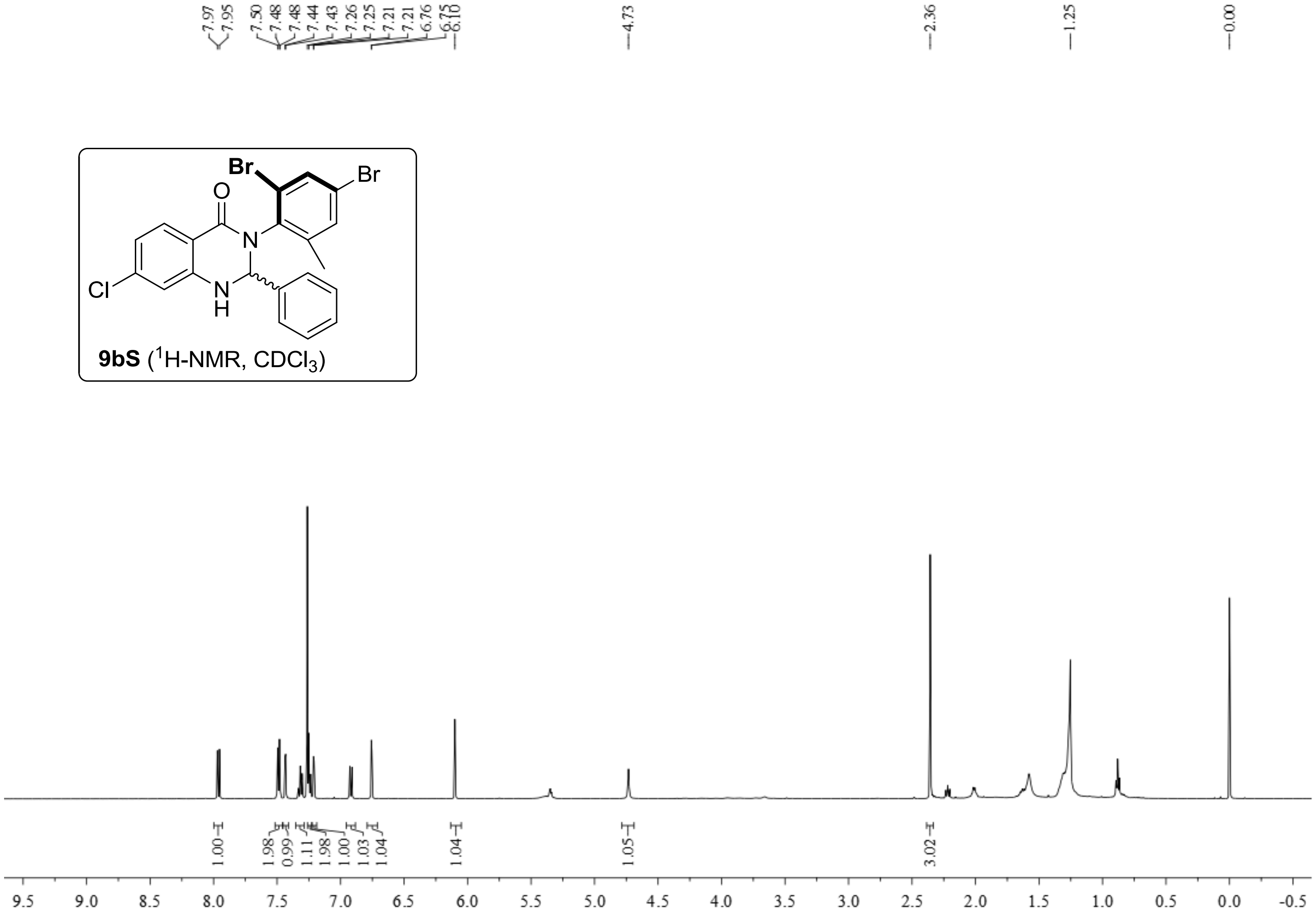


**Supplementary Figure 202.** $^{1}$H NMR of **9bS**

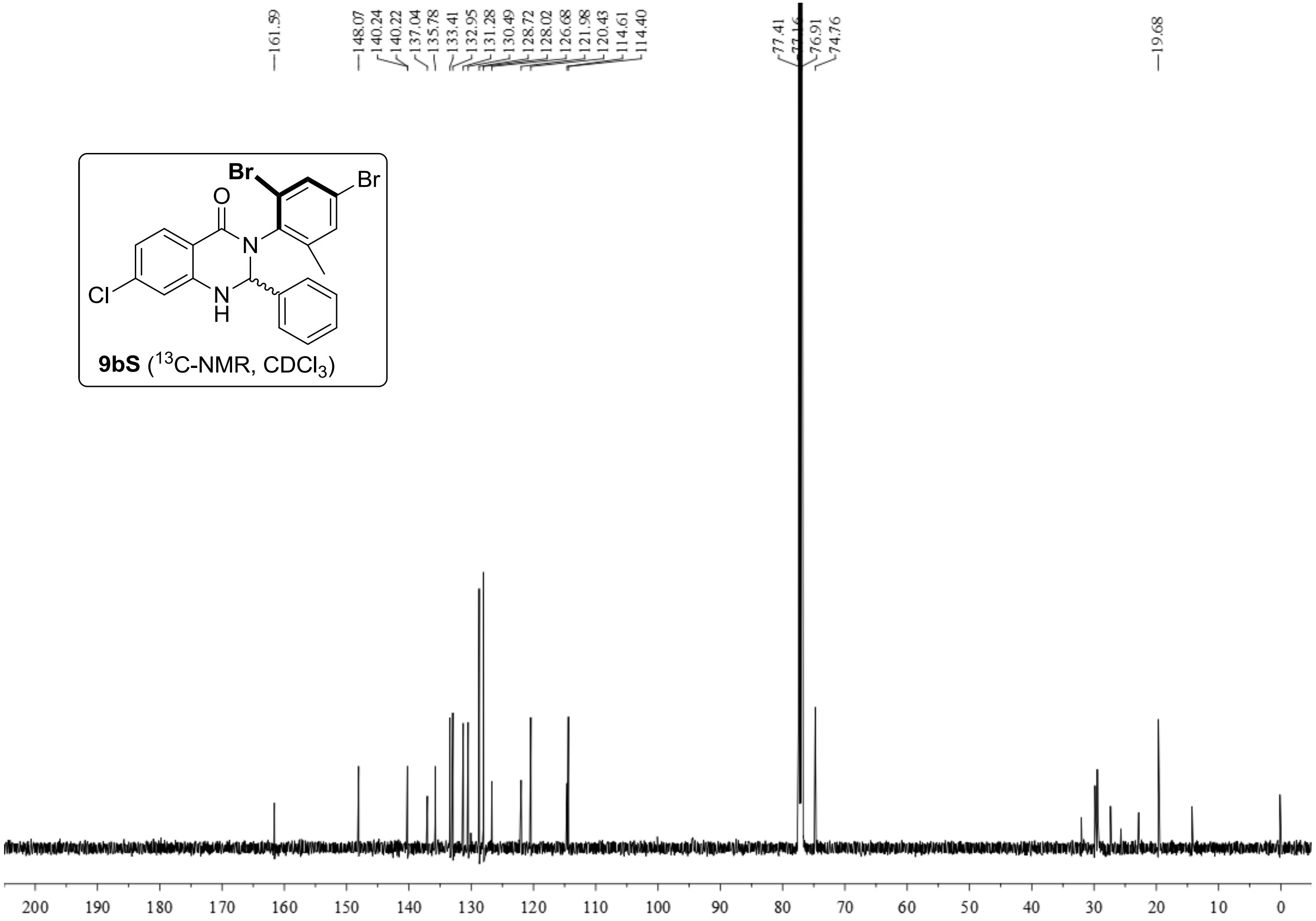


**Supplementary Figure 203.** $^{13}$C NMR of **1h9bS**

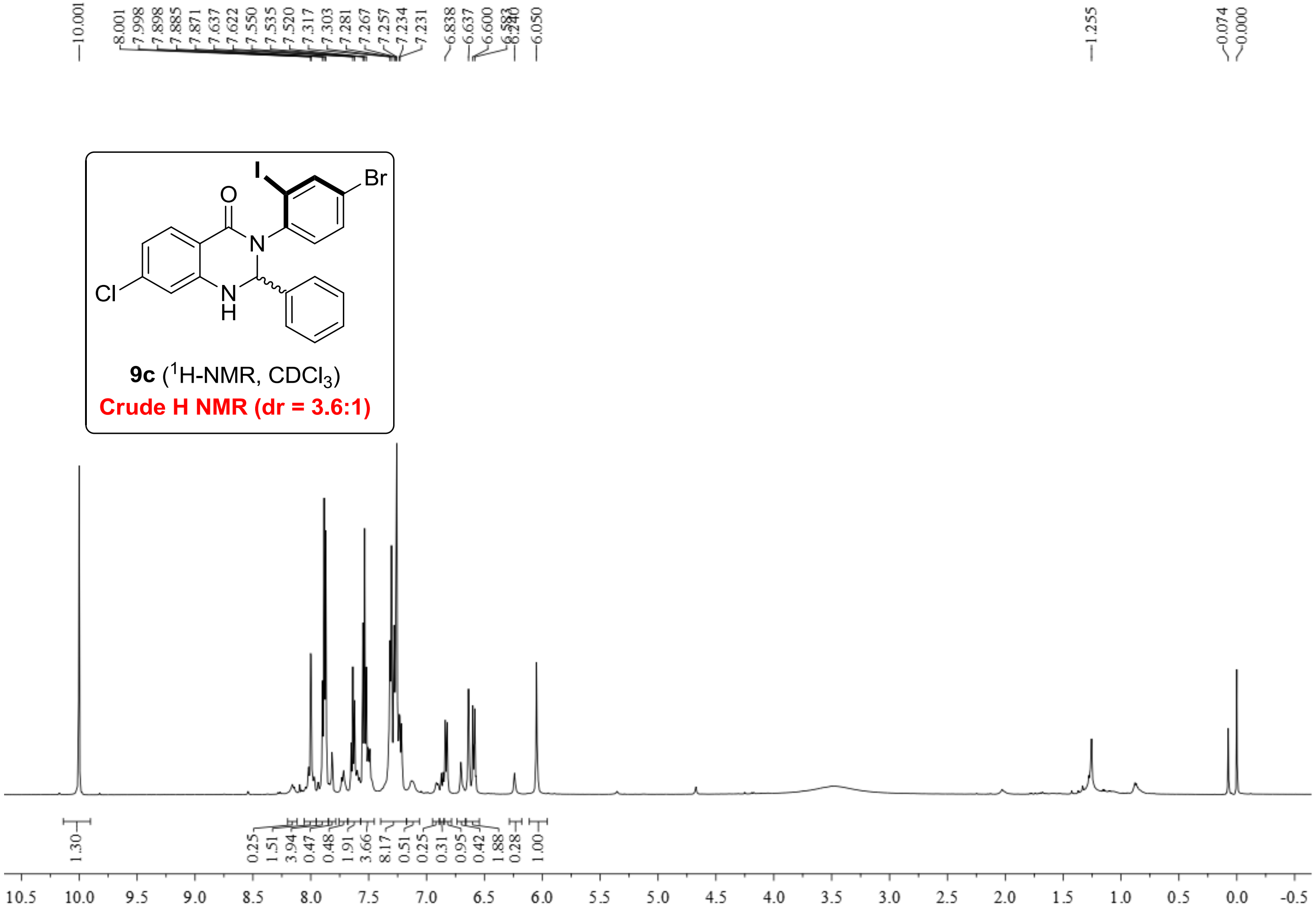


**Supplementary Figure 204.** Crude $^{1}$H NMR of **9c**

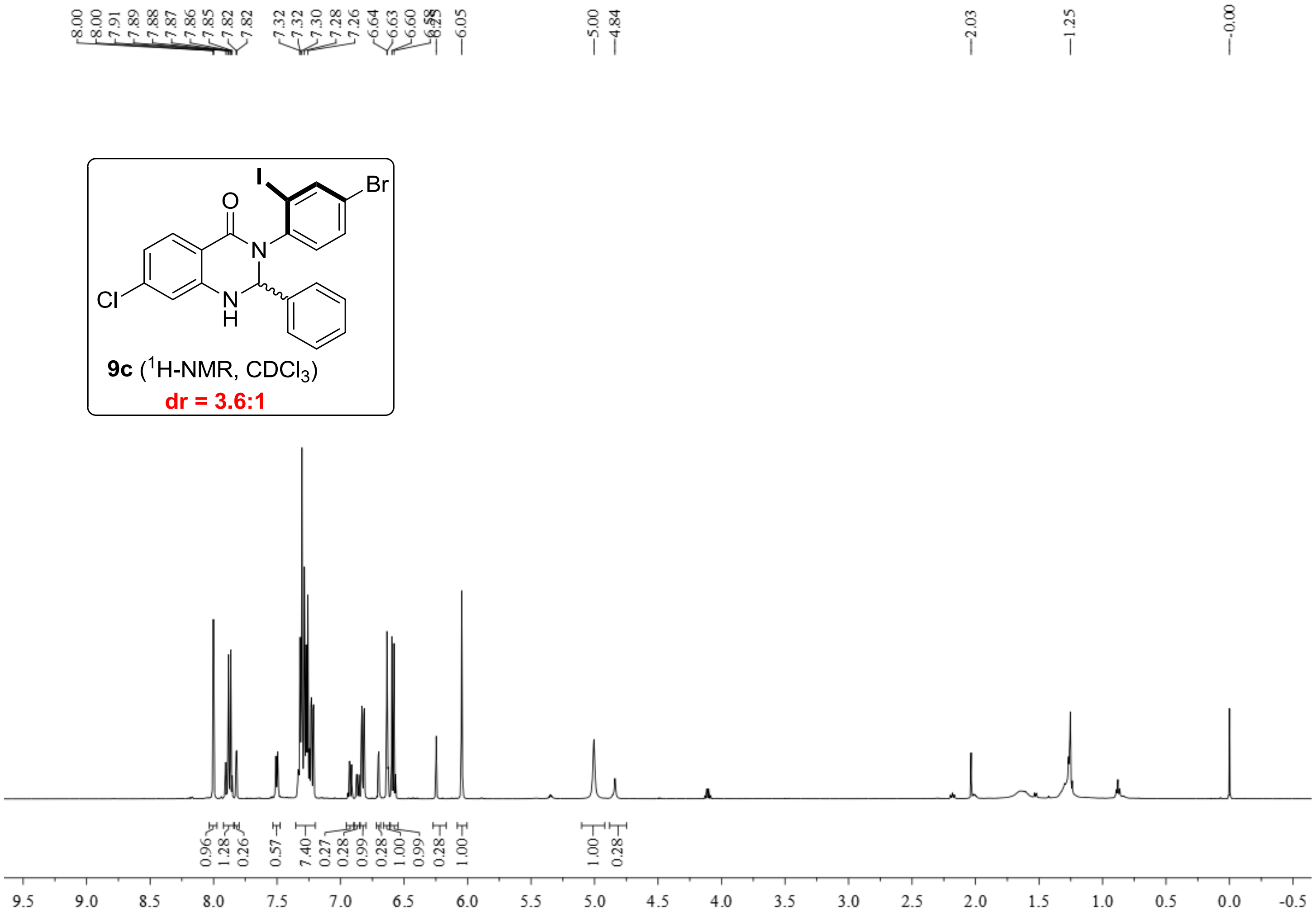


**Supplementary Figure 205.** $^1$H NMR of **9c**

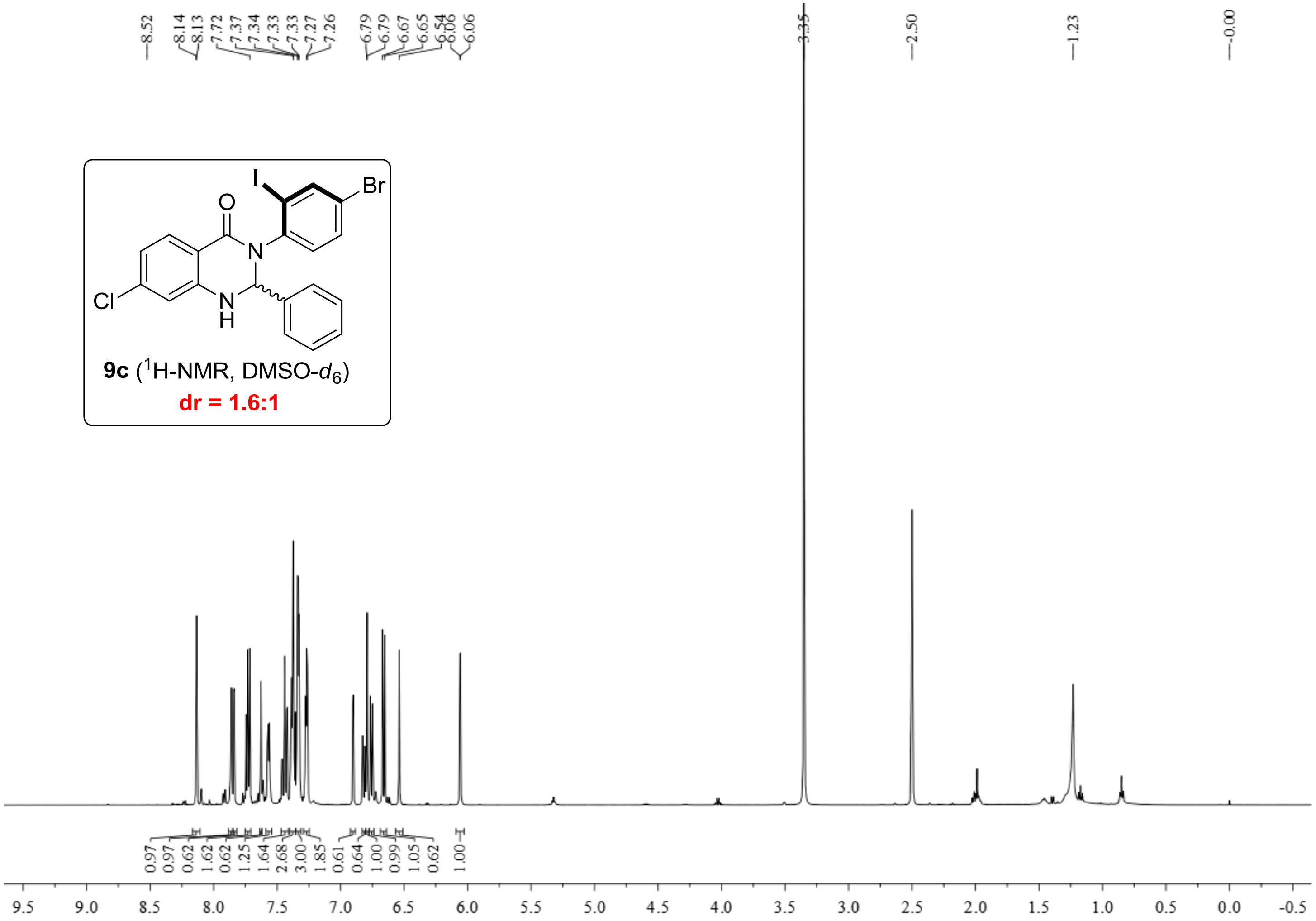


**Supplementary Figure 206.** $^{1}$H NMR of **9c**

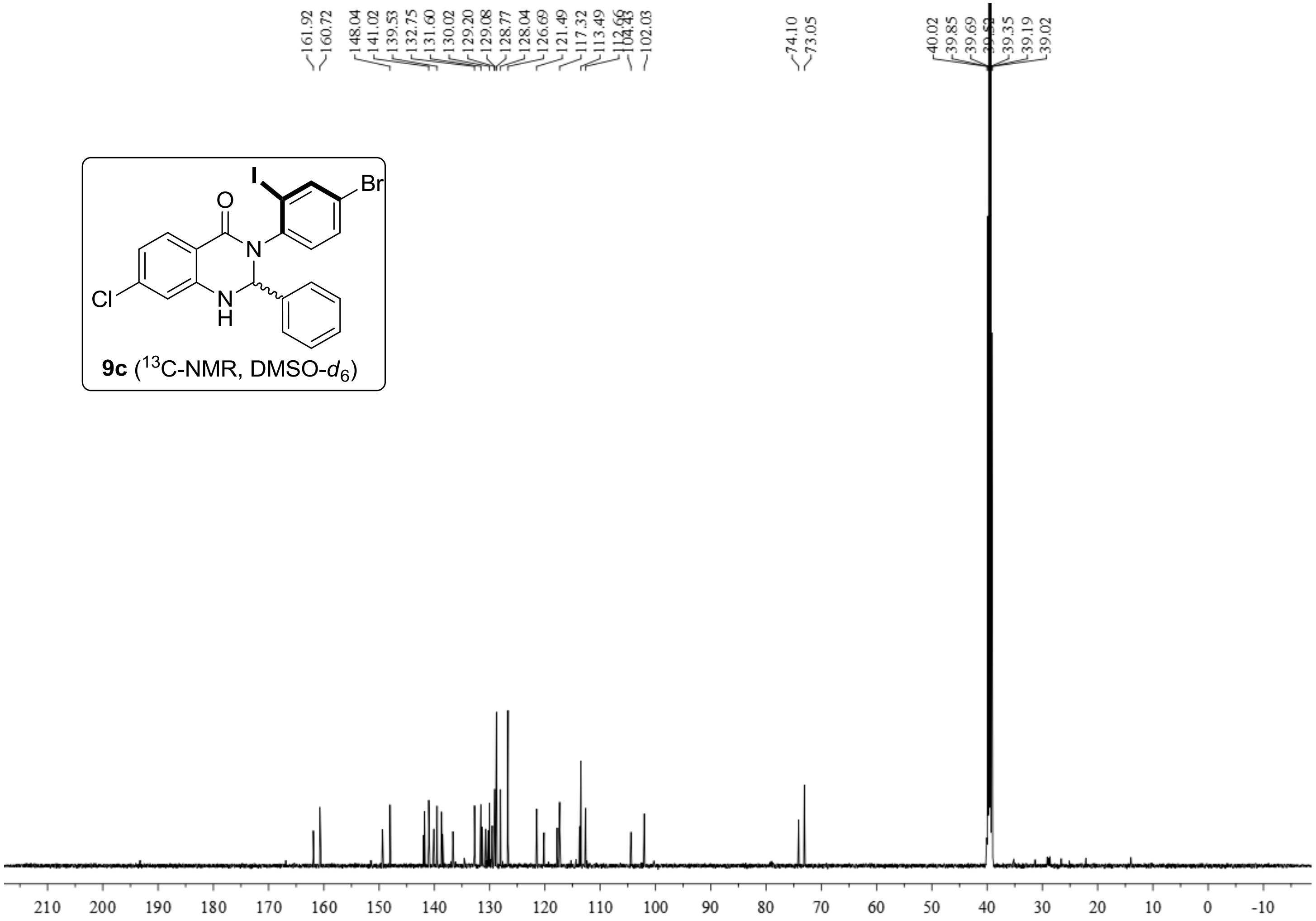


**Supplementary Figure 207.** $^{13}$C NMR of **9c**

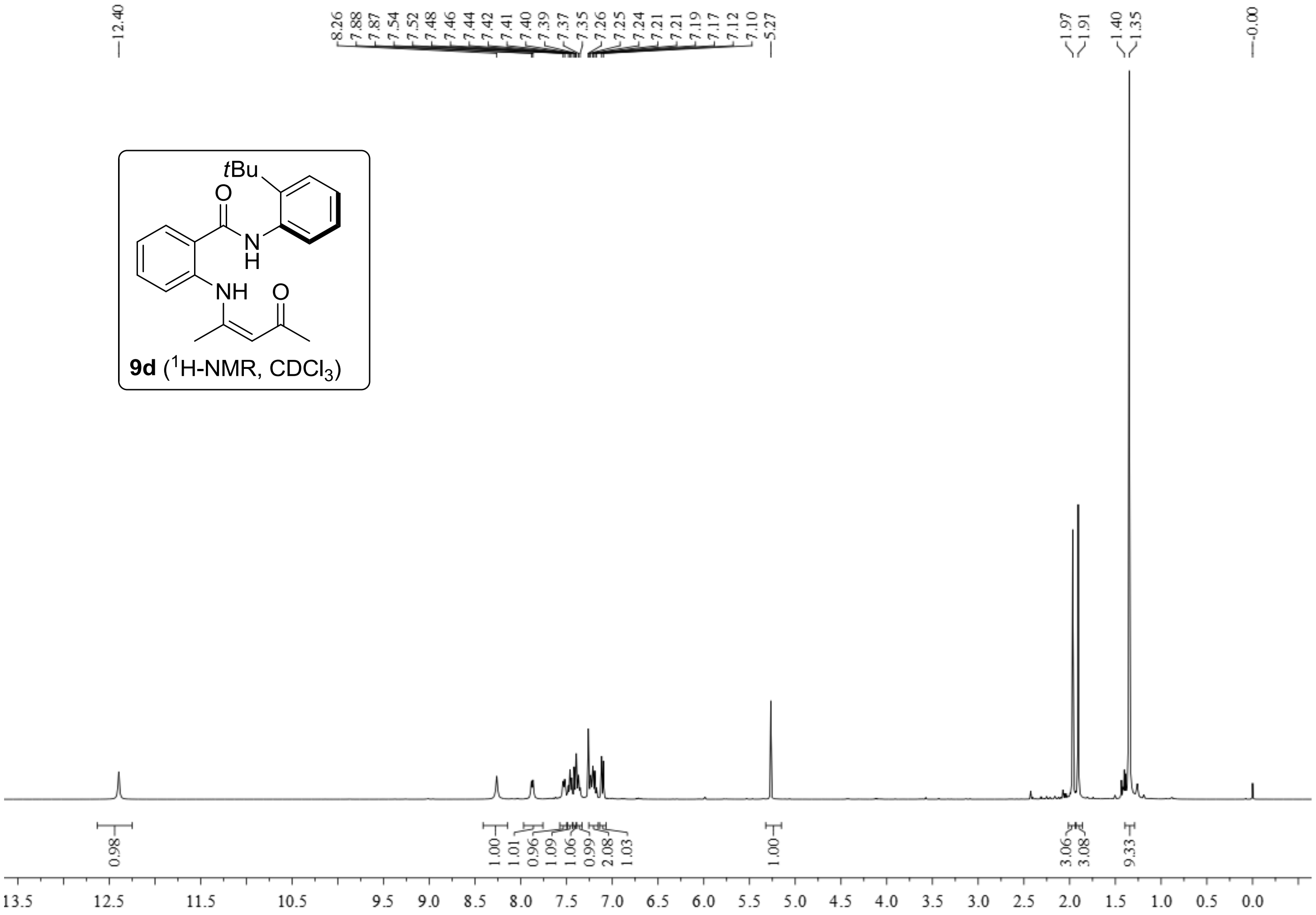


**Supplementary Figure 20.8** $^1$H NMR of **9d**

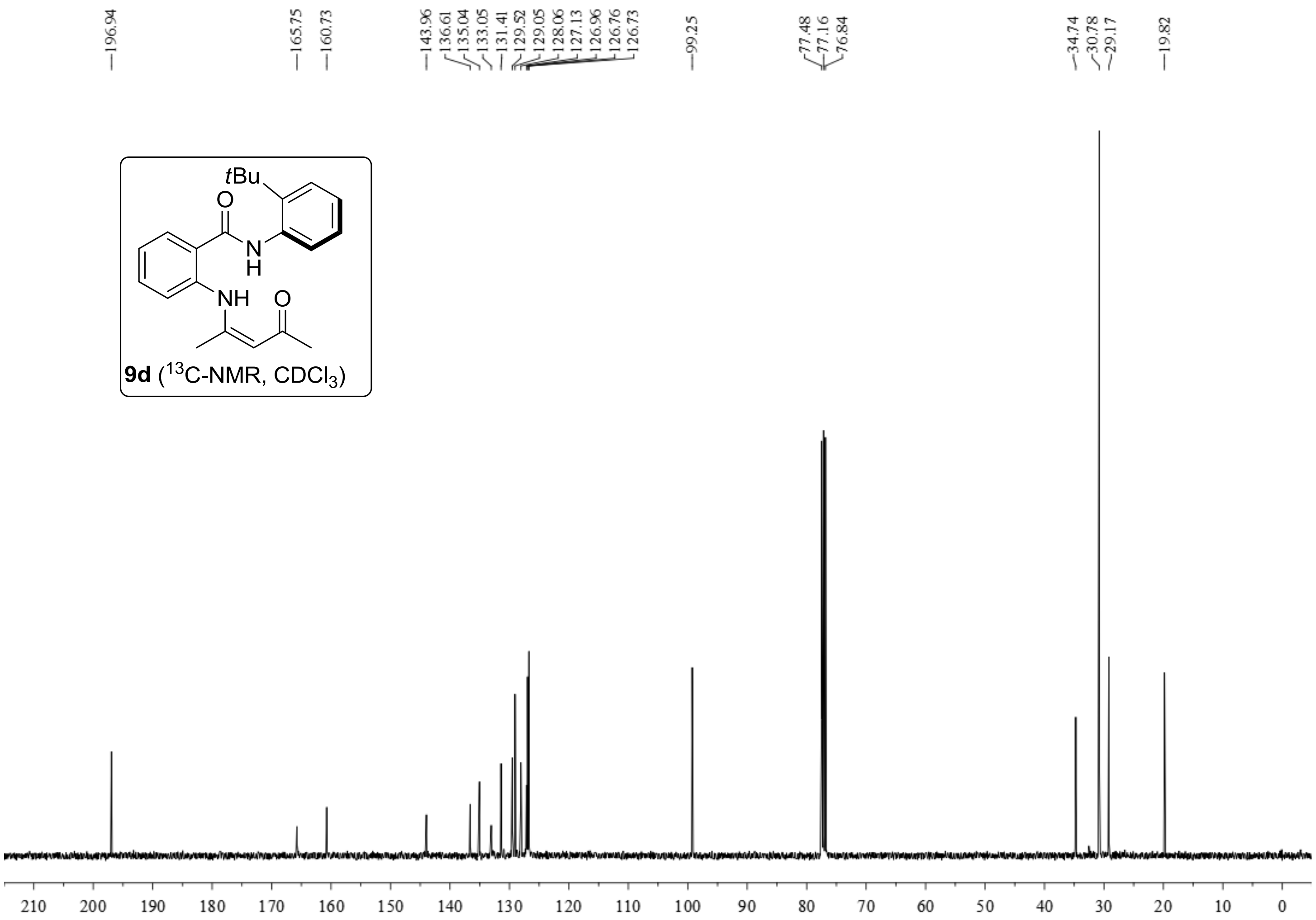


**Supplementary Figure 209.** $^{13}$C NMR of **9d**

# Supplementary Tables

## Supplementary Table 1.

## Optimization of the reaction conditions of using 1h with 4-methoxypent-3-en-2 –one [a]

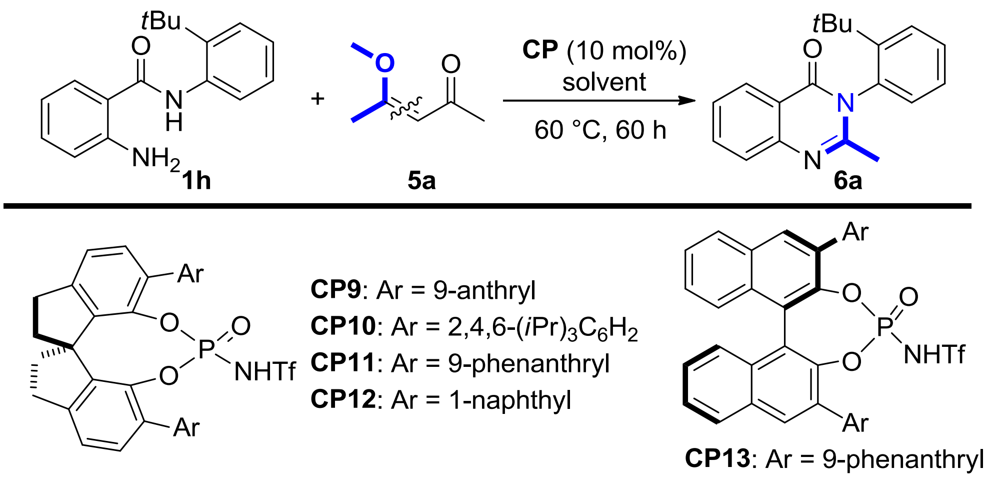


| entry | catalyst | solvent | $MgSO_4$ | yield (%)[b] | ee (%)[c] |
|---|---|---|---|---|---|
| 1 | **CP3** | $CHCl_3$ | -- | 69 | -34 |
| 2 | **CP7** | $CHCl_3$ | -- | 50 | -43 |
| 3 | **CP8** | $CHCl_3$ | -- | 84 | 33 |
| **4** | **CP9** | **$CHCl_3$** | -- | **57** | **78.4** |
| 5 | **CP10** | $CHCl_3$ | -- | 78 | 25 |
| 6 | **CP11** | $CHCl_3$ | -- | 61 | 43 |
| 7 | **CP12** | $CHCl_3$ | -- | 55 | 49 |
| 8 | **CP13** | $CHCl_3$ | -- | 48 | -40 |
| 9 [d] | **CP9** | $CHCl_3$ | -- | 11 | 86 |
| 10[e] | **CP9** | $CHCl_3$ | -- | 22 | 79 |
| 11 | **CP9** | $CHCl_3$ | -- | 60 | 61 |
| 12 | **CP9** | $CCl_4$ | -- | 96 | 72 |
| 13 | **CP9** | *c*-hexane | -- | 98 | 68 |
| 14 | **CP9** | toluene | -- | 88 | 75 |
| 15 | **CP9** | $CHCl_4$ (4.0 mL) | -- | 60 | 79 |
| 16 | **CP9** | *c*-hexane:$CHCl_3$ (1:1) | -- | 71 | 80 |
| 17 | **CP9** | *c*-hexane:$CHCl_3$ (2:1) | -- | 82 | 79 |
| 18 | **CP9** | $CCl_4$:$CHCl_3$ (1:1) | -- | 74 | 79 |
| 19 [f] | **CP9** | $CHCl_3$ | 50 mg | 0 | -- |
| 20 | **CP9** | $CHCl_3$ | 100 mg | 75 | 46 |
| 21 | **CP9** | $CHCl_3$ | 50 mg | 67 | 74 |
| **22** | **CP9** | **$CHCl_3$** | **0.6 mg** | **58** | **82** |
| 23 | **CP9** | *c*-hexane:$CHCl_3$ (1:1) | 0.6 mg | 68 | 82.6 |

| | | | | | |
|---|---|---|---|---|---|
| 24 | **CP9** | *c*-hexane:$CHCl_3$ (2:1) | 0.6 mg | 77 | 82 |
| **25** [g] | **CP9** | *c*-hexane:$CHCl_3$ (1:1) | 0.6 mg | 52 | 82.8 |
| **26** | **CP9** | ***c*-hexane:$CHCl_3$ (1:1)** | **3.0 mg** | **62** | **83.3** |
| 27 | **CP9** | *c*-hexane:$CHCl_3$ (1:1) | 12 mg | 62 | 83.3 |
| **28** [h] | **CP9** | ***c*-hexane:$CHCl_3$ (1:1)** | **6.0 mg** | **84 (83[i])** | **83** |

[a] Unless otherwise specified, the reaction of **1h** (13.4 mg, 0.05 mmol), **5a** (11.4 mg, 0.1 mmol), catalyst (10 mol %) and additive was carried out in 1.0 mL solvent (2.0 mL for mixture) at 60 °C for 60 h under Ar. [b] H-NMR yield with $CH_2Br_2$ as internal standard substance. [c] Determined by HPLC analysis. [d] At rt. [e] Acetylacetone was used instead of **5a**. [f] 4 Å MS was used instead of $MgSO_4$. [g] At 50 °C. [h] Reaction conditions: **1h** (26.8 mg, 0.1 mmol), **5a** (22.8 mg, 0.2 mmol), **CP9** (8.0 mg, 10 mol %) and additive (6.0 mg) was carried out in 4.0 mL solvent at 60 °C for 96 h. [i] Isolated yield.

## Supplementary Table 2.

## Screening the substrates scope of the atroposelective reaction for the total synthesis of eupolyphagin [a]

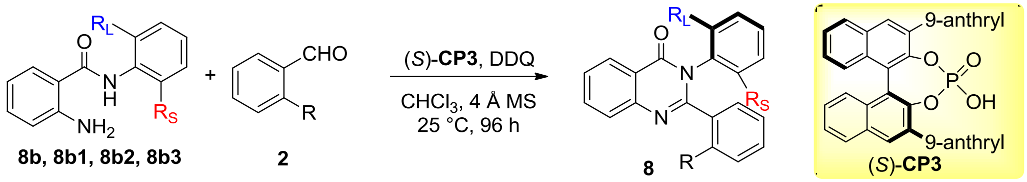


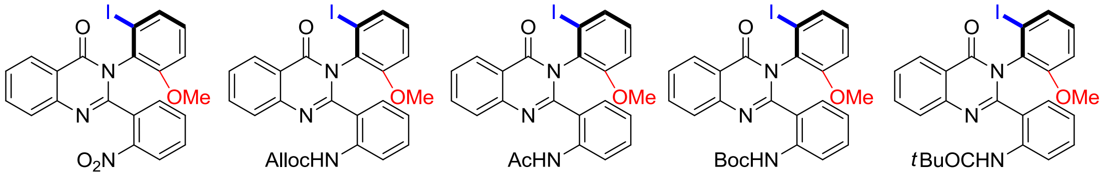


| 8c-a | 8c-b | 8c-c | 8c-d | 8c-e |
|---|---|---|---|---|
| 99% yield, 31% ee | 94%% yield, 56% ee | 97% yield, 50% ee | 91% yield, 52% ee | 87% yield, 42% ee |

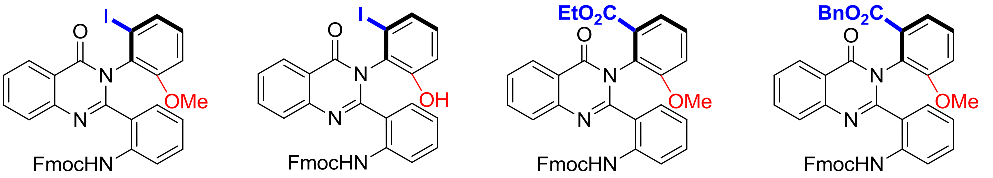


| 8c | 8c-1 | 8c-2 | 8c-3 |
|---|---|---|---|
| 96% yield, 58% ee | 94% yield, 5% ee [b] | 91% yield, 44% ee | 67% yield, 39% ee |

[a] Unless otherwise specified, the reaction of **8b** (0.1 mmol), **2** (0.2 mmol), DDQ (31.9 mg, 0.14 mmol), *S*-**CP3** (7.0 mg, 10 mol%) and 4 Å MS was carried out in 4.0 mL $CHCl_3$ at 25 °C for 96 h under Ar. [b] DDQ was added after 48 h and then the reaction was performed for additional 96 h.

## Supplementary Table 3.

## Optimization of the reaction conditions of palladium-catalyzed carbonylation of 8d [a]

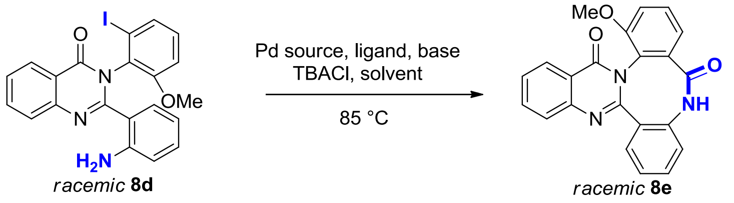


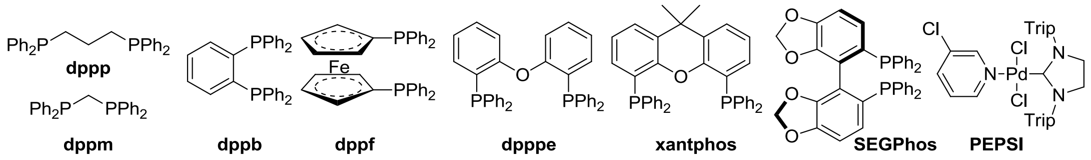


| entry | Pd source | ligand | solvent | time/h | yield (%)[b] |
|---|---|---|---|---|---|
| 1 | $Pd_2dba_3{\cdot}CHCl_3$ | *t*$Bu_3P$ | $CH_3CN$ | 96 | 49 |
| **2** | **$Pd_2dba_3{\cdot}CHCl_3$** | **dppp** | **$CH_3CN$** | **96** | **84** |
| 3 | $Pd_2dba_3{\cdot}CHCl_3$ | dppf | $CH_3CN$ | 96 | 57 |
| 4 | $Pd_2dba_3{\cdot}CHCl_3$ | dppm | $CH_3CN$ | 96 | 58 |
| 5 | $Pd_2dba_3{\cdot}CHCl_3$ | Xantphos | $CH_3CN$ | 96 | 25 |
| 6 | $Pd_2dba_3{\cdot}CHCl_3$ | dppb | $CH_3CN$ | 96 | 27 |
| 7 | $Pd_2dba_3{\cdot}CHCl_3$ | dpppe | $CH_3CN$ | 96 | 18 |
| 8 | $Pd_2dba_3{\cdot}CHCl_3$ | SEGPhos | $CH_3CN$ | 96 | 63 |
| 9[c] | PEPSI | -- | $CH_3CN$ | 96 | 35 |
| 10 | $Pd_2dba_3{\cdot}CHCl_3$ | dppp | DMF | 96 | 80 |
| 11[d] | $Pd_2dba_3{\cdot}CHCl_3$ | dppp | DMF | 96 | trace |
| **12** | **$Pd_2dba_3{\cdot}CHCl_3$** | **dppp** | **toluene** | **96** | **96** |
| 13 | $Pd_2dba_3{\cdot}CHCl_3$ | dppp | toluene | 72 | 93 |
| **14**[c] | **$Pddba_2$** | **dppp** | **toluene** | **72** | **96** |
| 15[c] | $Pddba_2$ | dppp | $CH_3CN$ | 120 | 97 |
| **16**[c, e] | **$Pddba_2$** | **dppp** | **toluene** | **72** | **95 (95%ee)** |

[a] Unless otherwise specified, the reaction of *racemic* **8d** (46.9 mg, 0.1 mmol), $Pd_2dba_3{\cdot}CHCl_3$ (7.8 mg, 0.0075 mmol), ligand (0.020 mmol), tetrabutylammonium chloride (77.9 mg, 0.3 mmol) and DIPEA (49.6 μL, 0.3 mmol) and CO (1 atm) was carried out in 2.0 mL solvent at 85 °C; [b] Isolated yield; [c] Pd source (0.015 mmol); [d] $K_3PO_4$ (42.5 mg, 0.2 mmol) was used instead of DIPEA; [e] **8d** (95% ee) was used instead of *racemic* **8d**.

# Supplementary Table 4.

## Crystal data and structure refinement for 3a

| | |
|---|---|
| Identification code | **cxy408_0m** |
| Empirical formula | $C_{24}H_{21}ClN_2O$ |
| Formula weight | 388.88 |
| Temperature | 100 K |
| Wavelength | 1.54178 Å |
| Crystal system | Monoclinic |
| Space group | $P2_1$ |
| Unit cell dimensions | a = 7.6488(3) Å α=90 º. |
| | b = 12.2911(4) Å β=90.419(1) º. |
| | c = 10.6841(4) Å γ=90 º. |
| Volume | 1004.41(6) $Å^3$ |
| Z | 2 |
| Density (calculated) | 1.286 $Mg/m^3$ |
| Absorption coefficient | 1.804 $mm^{-1}$ |
| F(000) | 408.0 |
| Crystal size | 0.2 × 0.2 × 0.15 |
| $2^{\Theta}$ range for data collection | 8.276 to 134.918° |
| Index ranges | -9 <= h <= 9, -14 <= k <= 13, -12 <= l <= 12 |
| Reflections collected | 12120 |
| Independent reflections | 3187 [Rint = 0.0461, Rsigma = 0.0429] |
| Completeness to theta = 67.459 ° | 1.67/0.88 |
| Absorption correction | Multi-Scan |
| Data / restraints / parameters | 3187 / 1 / 257 |
| Goodness-of-fit on $F^2$ | 1.049 |
| Final R indices [I>2sigma(I)] | R1 = 0.262, wR2 = 0.0674 |
| R indices (all data) | R1 = 0.262, wR2 = 0.0674 |
| Absolute structure parameter | 0.069(14) |
| Extinction coefficient | n/a |
| Largest diff. peak and hole | 0.16/-0.19 $Å^{-3}$ |

## Supplementary Table 5.

## Crystal data and structure refinement for 6f

| | |
|---|---|
| Identification code | **cxy510_0m** |
| Empirical formula | $C_{19}H_{18}BrFN_2O$ |
| Formula weight | 389.26 |
| Temperature | 100 K |
| Wavelength | 1.54178 Å |
| Crystal system | Orthorhombic |
| Space group | $P2_12_12_1$ |
| Unit cell dimensions | a = 8.5688(3) Å α=90 °. |
| | b = 12.4308(4) Å β=90 °. |
| | c = 16.2377(6) Å γ=90 °. |
| Volume | 1729.59(10) $Å^3$ |
| Z | 4 |
| Density (calculated) | 1.495 $Mg/m^3$ |
| Absorption coefficient | 3.392 $mm^{-1}$ |
| F(000) | 792.0 |
| Crystal size | 0.28 × 0.23 × 0.21 |
| $2^{\Theta}$ range for data collection | 8.958 to 133.828 ° |
| Index ranges | -10 <= h <= 7, -14 <= k <= 13, -19 <= l <= 17 |
| Reflections collected | 15543 |
| Independent reflections | 3021 [$R_{int}$ = 0.0386, $R_{sigma}$ = 0.0287] |
| Completeness to theta = 66.914 ° | 1.70/0.98 |
| Absorption correction | Multi-Scan |
| Data / restraints / parameters | 3021 / 0 / 221 |
| Goodness-of-fit on $F^2$ | 1.078 |
| Final R indices [I>2sigma(I)] | R1 = 0.0235, wR2 = 0.0606 |
| R indices (all data) | R1 = 0.235, wR2 = 0.0606 |
| Absolute structure parameter | -0.001(6) |
| Extinction coefficient | n/a |
| Largest diff. peak and hole | 0.24/-0.37 $Å^{-3}$ |

# Supplementary Note 1

## General information

All solvents and reagents were purchased at the highest commercial quality and used without further purification, unless otherwise stated. Analytical thin layer chromatography (TLC) was performed on precoated silica gel 60 F254 plates. Flash column chromatography was performed using Tsingdao silica gel (60, particle size 0.040-0.063 mm). Visualization on TLC was achieved by use of UV light (254, 365nm). NMR spectrums were recorded on a Bruker DPX 400 NMR spectrometer at 400 MHz for $^{1}$H NMR, 100 MHz for $^{13}$C NMR, 376MHz for $^{19}$F and 162 MHz for $^{31}$P NMR in DMSO-$d_6$, $CD_3OD$ or $CDCl_3$ with tetramethylsilane (TMS) as internal standard. Chemical shifts are reported in ppm and coupling constants are given in Hz. Data for $^{1}$H NMR, $^{13}$C NMR, $^{19}$F NMR and $^{31}$P NMR are recorded as follows: chemical shift (ppm), multiplicity (s, singlet; d, doublet; t, triplet; q, quarter; m, multiplet, brs, broad singlet; dd, double of doublet), coupling constant (Hz), integration. Enantioselectivities were recorded on Agilent HPLC using CHIRALPAK and CHIRALCEL columns.

# Supplementary Note 2

## Experimental procedure for the preparation of *N*-aryl-anthranilamides (1a-1y)

### Procedure A

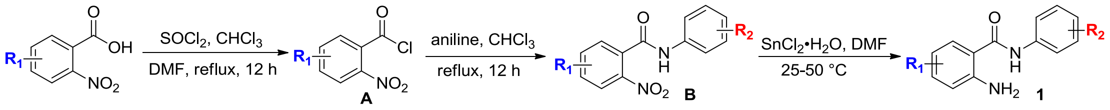


To a 100 mL round bottom flask, equipped with a magnetic stir bar, 2-nitrobenzoic acid derivative (8 mmol), a drop of DMF and $CHCl_3$ (40 mL) was added. $SOCl_2$ (1.74 mL, 2.88 g, 24 mmol) was added drop-wise to the mixture at 0 ºC. After the addition, the ice-water bath was removed and the mixture was heated to reflux for 12 h. The clear solution was concentrated under reduced pressure to remove the solvent and excess $SOCl_2$. The obtained crude product **A** was directly used in the next step without further purification. To the solution of **A** (8 mmol) in $CHCl_3$ (30 mL), aniline (8.8 mmol, 1.1 eq) was added slowly and then the solution was heated to reflux for 12 h. The reaction solution was diluted to 60 mL with DCM and then washed with saturated $NaHCO_3$ (2 x 20 mL), 1 M HCl (20 mL) and brine (20 mL). The organic layer was dried over $Na_2SO_4$ and concentrated under reduced pressure. The residue was recrystallized from PE/DCM to afford the desired product **B**. $SnCl_2{\cdot}2H_2O$ (9.0 g, 40 mmol) was added to the solution of **B** (4 mmol) in DMF (30 mL) in one portion at 0 ºC. After 15 min, the solution was warmed to room temperature and stirred for 4-24 h (monitored by TLC). Upon completion, the solution was slowly added to saturated $NaHCO_3$ solution (200 mL). The mixture was extracted with EA (2 x 45 mL). The combined organic layer was washed with $H_2O$ (5 x 50 mL) and brine (30 mL), dried over $Na_2SO_4$ and concentrated under reduced pressure. The residue was purified through flash chromatography on silica gel to yield the corresponding products **1a-1t** and **1v-1y.**

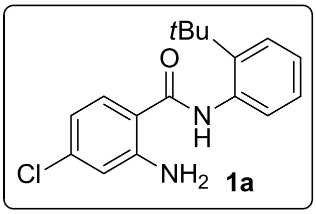


**2-Amino-*N*-(2-(*tert*-butyl)phenyl)-4-chlorobenzamide (1a)**

According to Procedure A, **1a** was obtained as a white solid. $^1$H NMR (400 MHz, $CDCl_3$) δ 7.69 (s, 1H), 7.54 (dd, J = 7.8, 1.5 Hz, 1H), 7.45 (dd, J = 7.9, 1.6 Hz, 1H), 7.40 (d, J = 8.4 Hz, 1H), 7.27 (td, J = 7.4, 1.6 Hz, 1H), 7.21 (td, J = 7.6, 1.6 Hz, 1H), 6.71 (d, J = 2.0 Hz, 1H), 6.67 (dd, J = 8.4, 2.0 Hz, 1H), 5.75 (s, 2H), 1.42 (s, 9H); $^{13}$C NMR (101 MHz, $CDCl_3$) δ 167.3, 150.6, 143.7, 138.7, 135.0, 128.8, 128.2, 126.98, 126.94, 126.8, 117.0, 117.0, 114.2, 34.8, 30.9; HRMS (m/z): $[M]^+$ calcd for $C_{17}H_{20}ON_2Cl$ 303.1259; found 303.1256.

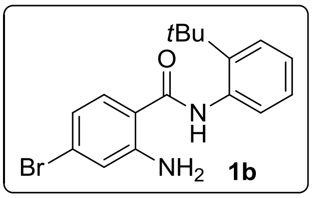


**2-Amino-4-bromo-*N*-(2-(*tert*-butyl)phenyl)benzamide (1b)**

According to Procedure A, **1b** was obtained as a white solid. $^1$H NMR (400 MHz, $CDCl_3$) δ 7.69 (s, 1H), 7.55 (dd, J = 7.8, 1.5 Hz, 1H), 7.45 (dd, J = 7.9, 1.6 Hz, 1H), 7.33 (d, J = 8.4 Hz, 1H), 7.28 (td, J = 7.4, 1.6 Hz, 1H), 7.22 (td, J = 7.6, 1.6 Hz, 1H), 5.74 (brs, 2H), 1.43 (s, 9H); $^{13}$C NMR (101 MHz, $CDCl_3$) δ 167.4, 150.7, 143.7, 135.0, 128.7, 128.2, 127.2, 127.00, 126.96, 126.8, 120.1, 119.9, 114.6, 34.9, 30.9; HRMS (m/z): $[M]^+$ calcd for $C_{17}H_{20}ON_2Br$ 347.0754; found 347.0752.

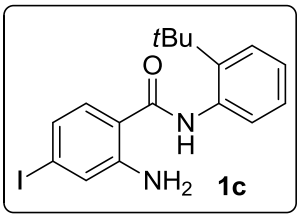


**2-Amino-*N*-(2-(*tert*-butyl)phenyl)-4-iodobenzamide (1c)**

According to Procedure A, **1c** was obtained as a yellowish solid. $^1$H NMR (400 MHz, $CDCl_3$) δ 7.69 (s, 1H), 7.55 (dd, J = 7.8, 1.4 Hz, 1H), 7.44 (dd, J = 7.9, 1.6 Hz, 1H), 7.30-7.26 (m, 1H), 7.22 (dd, J = 7.7, 1.5 Hz, 1H), 7.17 (d, J = 8.3 Hz, 1H), 7.12 (d, J = 1.6 Hz, 1H), 7.04 (dd, J = 8.3, 1.6 Hz, 1H), 5.67 (s, 2H), 1.42 (s, 9H); $^{13}$C NMR (101 MHz, $CDCl_3$) δ 167.5, 150.4, 143.6, 135.0, 128.7, 128.0, 126.99, 126.95, 126.8, 126.3, 125.8, 115.1, 99.7, 34.9, 30.9; HRMS (m/z): $[M]^+$ calcd for $C_{17}H_{20}ON_2I$ 395.0615; found 395.0612.

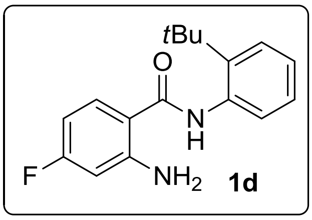


**2-Amino-*N*-(2-(*tert*-butyl)phenyl)-4-fluorobenzamide (1d)**

According to Procedure A, **1d** was obtained as a white solid. $^1$H NMR (400 MHz, $CDCl_3$) δ 7.64 (s, 1H), 7.54 (dd, J = 7.8, 1.4 Hz, 1H), 7.49-7.43 (m, 2H), 7.31-7.25 (m, 1H), 7.27 (td, J = 7.4, 1.6 Hz, 1H), 7.21 (td, J = 7.6, 1.6 Hz, 1H), 6.44-6.37 (m, 2H), 5.84 (brs, 2H), 1.43 (s, 9H); $^{13}$C NMR (101 MHz, $CDCl_3$) δ 167.4, 165.7 (d, $^1J_{CF}$ = 250.1 Hz), 152.0 (d, $^3J_{CF}$ = 12.2 Hz), 143.7, 135.1, 129.2 (d, $^3J_{CF}$ = 11.2 Hz), 128.9, 126.97, 126.93, 126.71, 112.2 (d, $^4J_{CF}$ = 1.9 Hz), 104.4 (d, $^2J_{CF}$ = 22.9 Hz), 103.4 (d, $^2J_{CF}$ = 24.2 Hz), 34.9, 30.9; $^{19}$F NMR (376 MHz, $CDCl_3$) δ -107.4; HRMS (m/z): $[M]^+$ calcd for $C_{17}H_{20}ON_2F$ 287.1551; found 287.1554.

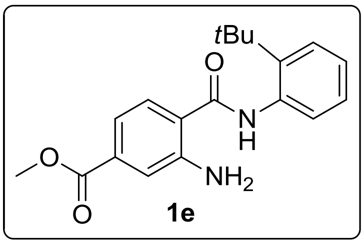


**2-Amino-*N*-(2-(*tert*-butyl)phenyl)-4-(methoxycarbonyl)benzamide (1e)**

According to Procedure A, **1e** was obtained as a yellowish solid. $^1$H NMR (400 MHz, $CDCl_3$) δ 7.81 (s, 1H), 7.58 (d, J = 7.8 Hz, 1H), 7.54 (d, J = 8.2 Hz, 1H), 7.45 (d, J = 7.8 Hz, 1H), 7.40 (s, 1H), 7.35 (dd, J = 8.2, 0.8 Hz, 1H), 7.29 (t, J = 7.6 Hz, 1H), 7.23 (dd, J = 14.1, 6.7 Hz, 1H), 5.70 (s, 2H), 3.92 (s, 3H), 1.43 (s, 9H); $^{13}$C NMR (101 MHz, $CDCl_3$) δ 167.3, 166.6, 149.3, 143.7, 134.9, 133.8, 128.7, 127.1, 127.0, 126.97, 126.86, 119.2, 118.8, 117.4, 52.5, 34.9, 30.9; HRMS (m/z): $[M]^+$ calcd for $C_{19}H_{23}O_3N_2$ 327.1703; found 327.1700.

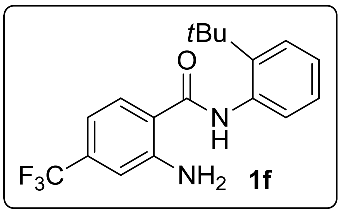


**2-Amino-*N*-(2-(*tert*-butyl)phenyl)-4-(trifluoromethyl)benzamide (1f)**

According to Procedure A, **1f** was obtained as a yellowish solid when the reduction reaction with $SnCl_2{\cdot}H_2O$ was performed at 50 ºC. $^1$H NMR (400 MHz, CDCl3) δ 7.77 (s, 1H), 7.57 (t, J = 6.9 Hz, 2H), 7.46 (dd, J = 7.8, 1.5 Hz, 1H), 7.29 (td, J = 7.5, 1.5 Hz, 1H), 7.23 (td, J = 7.4, 1.4 Hz, 1H), 6.96 (s, 1H), 6.94 (d, J = 8.3 Hz, 1H), 5.80 (s, 2H), 1.43 (s, 9H); $^{13}$C NMR (101 MHz, $CDCl_3$) δ 167.1, 149.5, 143.7, 134.8, 134.5 (q, $^2J_{CF}$ = 32.5 Hz), 128.7, 127.7, 127.04, 127.02 (s), 126.97, 123.7 (q, $^1J_{CF}$ = 272.7 Hz), 118.3, 114.3 (q, $^3J_{CF}$ = 3.9 Hz), 113.0 (q, $^3J_{CF}$ = 3.6 Hz), 34.9, 30.9; $^{19}$F NMR (376 MHz, $CDCl_3$) δ -63.7. HRMS (m/z): $[M]^+$ calcd for $C_{18}H_{20}ON_2F_3$ 337.1522; found 337.1518.

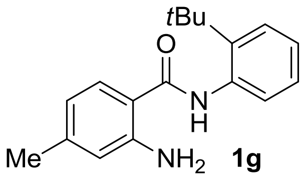

1g

**2-Amino-*N*-(2-(*tert*-butyl)phenyl)-4-methylbenzamide (1g)**

According to Procedure A, **1g** was obtained as a yellowish crystal. $^{1}$H NMR (400 MHz, $CDCl_3$) δ 7.73 (s, 1H), 7.59 (dd, J = 7.8, 1.4 Hz, 1H), 7.44 (dd, J = 7.9, 1.5 Hz, 1H), 7.38 (d, J = 8.4 Hz, 1H), 7.29-7.25 (m, 1H), 7.19 (td, J = 7.7, 1.5 Hz, 1H), 6.54 (dd, J = 3.6, 3.1 Hz, 2H), 5.65 (brs, 2H), 2.29 (s, 3H), 1.43 (s, 9H); $^{13}$C NMR (101 MHz, $CDCl_3$) δ 167.9, 149.8, 143.5, 135.4, 128.7, 126.9, 126.8, 126.4, 118.2, 118.1, 34.8, 30.9, 21.6; HRMS (m/z): $[M]^+$ calcd for $C_{18}H_{23}ON_2$ 283.1805; found 283.1802.

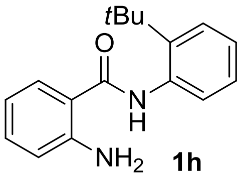

1h

**2-Amino-*N*-(2-(*tert*-butyl)phenyl)benzamide (1h)**

According to Procedure A, **1h** was obtained as a white solid. $^{1}$H NMR (400 MHz, $CDCl_3$) δ 7.76 (s, 1H), 7.60 (dd, J = 7.8, 1.2 Hz, 1H), 7.49 (dd, J = 8.1, 0.9 Hz, 1H), 7.45 (dd, J = 7.9, 1.4 Hz, 1H), 7.30-7.25 (m, 2H), 7.21 (td, J = 7.7, 1.4 Hz, 1H), 6.75-6.71 (m, 2H), 5.65 (s, 2H), 1.44 (s, 9H); $^{13}$C NMR (101 MHz, $CDCl_3$) δ 167.8, 147.9, 143.8, 135.1, 132.9, 128.9, 126.97, 126.88, 126.69, 118.7, 118.1, 116.8, 34.9, 30.9; HRMS (m/z): $[M]^+$ calcd for $C_{17}H_{21}ON_2$ 269.1648; found 269.1645.

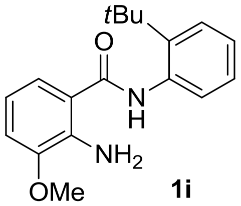

1i

**2-Amino-*N*-(2-(*tert*-butyl)phenyl)-3-Methoxybenzamide (1i)**

According to Procedure A, **1i** was obtained as a white solid. $^{1}$H NMR (400 MHz, $CDCl_3$) δ 7.78 (s, 1H), 7.62 (dd, J = 7.8, 1.3 Hz, 1H), 7.44 (dd, J = 7.9, 1.5 Hz, 1H), 7.28 (td, J = 7.7, 1.5 Hz, 1H), 7.20 (td, J = 7.7, 1.5 Hz, 1H), 7.12 (d, J = 7.6 Hz, 1H), 6.87 (d, J = 7.7 Hz, 1H), 6.68 (t, J = 8.0 Hz, 1H), 5.95 (s, 2H), 3.89 (s, 3H), 1.44 (s, 9H); $^{13}$C NMR (101 MHz, $CDCl_3$) δ 167.9, 148.0, 143.4, 140.7, 135.3, 128.6, 126.9, 126.8, 126.4, 118.4, 115.5, 114.9, 112.1, 55.9, 34.8, 30.9; HRMS (m/z): $[M]^+$ calcd for $C_{18}H_{23}O_2N_2$ 299.1754; found 299.1753.

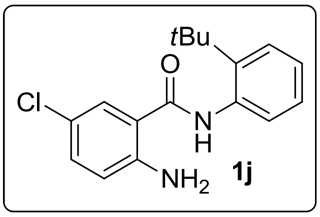


**2-Amino-*N*-(2-(*tert*-butyl)phenyl)-5-chlorobenzamide (1j)**

According to Procedure A, **1j** was obtained as a white solid. $^1$H NMR (400 MHz, $CDCl_3$) δ 7.67 (s, 1H), 7.50 (d, J = 7.7 Hz, 1H), 7.46-7.44 (m, 1H), 7.30-7.19 (m, 3H), 6.67 (d, J = 8.8 Hz, 1H), 5.60 (s, 2H), 1.43 (s, 9H); $^{13}$C NMR (101 MHz, $CDCl_3$) δ 167.0, 148.0, 144.0, 134.9, 132.7, 129.0, 126.99, 126.95, 126.6, 121.2, 119.0, 116.9, 34.9, 30.9; HRMS (m/z): $[M]^+$ calcd for $C_{17}H_{20}ON_2Cl$ 303.1259; found 303.1256.

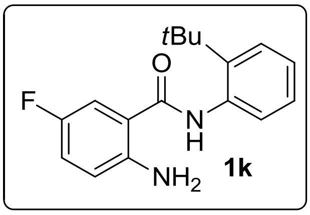


**2-Amino-*N*-(2-(*tert*-butyl)phenyl)-5-fluorobenzamide (1k)**

According to Procedure A, **1k** was obtained as a white solid. $^1$H NMR (400 MHz, $CDCl_3$) δ 7.71 (d, J = 13.8 Hz, 1H), 7.54 (dd, J = 7.8, 1.0 Hz, 1H), 7.45 (dd, J = 7.8, 1.7 Hz, 1H), 7.27 (dd, J = 7.4, 1.6 Hz, 1H), 7.24-7.18 (m, 2H), 7.05-7.00 (m, 1H), 6.69 (dd, J = 9.0, 4.7 Hz, 1H), 5.39 (brs, 2H), 1.42 (d, J = 6.5 Hz, 9H); $^{13}$C NMR (101 MHz, $CDCl_3$) δ 167.0 (d, $^4J_{CF}$ = 2.3 Hz), 154.6 (d, $^1J_{CF}$ = 236.3 Hz), 145.7, 143.8, 134.9, 128.8, 126.98, 126.95, 126.84, 120.3 (d, $^2J_{CF}$ = 22.7 Hz), 119.0 (d, $^3J_{CF}$ = 7.1 Hz), 116.2 (d, $^3J_{CF}$ = 5.2 Hz), 112.8 (d, $^2J_{CF}$ = 22.9 Hz), 34.9, 30.9; $^{19}$F NMR (376 MHz, $CDCl_3$) δ -127.1; HRMS (m/z): $[M]^+$ calcd for $C_{17}H_{20}ON_2F$ 287.1554; found 287.1552.

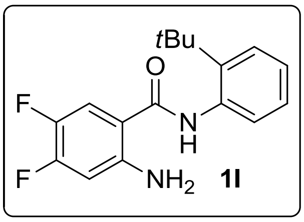


**2-Amino-*N*-(2-(*tert*-butyl)phenyl)-4,5-difluorobenzamide (1l)**

According to Procedure A, **1l** was obtained as a light brown solid. $^1$H NMR (400 MHz, $CDCl_3$) δ 7.58 (s, 1H), 7.49 (dd, J = 7.7, 1.2 Hz, 1H), 7.45 (dd, J = 7.8, 1.5 Hz, 1H), 7.32-7.25 (m, 2H), 7.22 (td, J = 7.6, 1.6 Hz, 1H), 6.50 (dd, J = 12.0, 6.8 Hz, 1H), 5.61 (brs, 2H), 1.43 (s, 9H); $^{13}$C NMR (101 MHz, $CDCl_3$) δ 166.6 (d, $^4J_{CF}$ = 1.5 Hz), 153.4 (dd, $^{1,2}J_{CF}$ = 252.6, 13.8 Hz), 147.2 (dd, $^{3,4}J_{CF}$ = 9.9, 1.3 Hz), 143.9, 142.2 (dd, $^{1,2}J_{CF}$ = 238.8, 13.7 Hz), 134.8, 128.9, 127.0, 127.0, 115.3 (dd, $^{2,3}J_{CF}$ = 18.3, 2.8 Hz), 111.023 (dd, $^3J_{CF}$ = 6.0Hz), 110.023, 105.5 (d, $^2J_{CF}$ = 19.9 Hz), 34.8, 30.9; $^{19}$F NMR (376 MHz, $CDCl_3$) δ -131.0 (d, $^3J_{FF}$ = 22.8 Hz), -151.4 (d, $^3J_{FF}$ = 22.8 Hz); HRMS (m/z): $[M]^+$ calcd for $C_{17}H_{19}ON_2F_2$ 305.1460; found 305.1456.

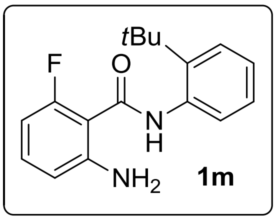


**2-Amino-*N*-(2-(*tert*-butyl)phenyl)-6-fluorobenzamide (1m)**

According to Procedure A, **1m** was obtained as a white solid. $^{1}$H NMR (400 MHz, $CDCl_3$) δ 8.37 (d, J = 17.3 Hz, 1H), 7.51 (t, J = 11.0 Hz, 1H), 7.45 (dd, J = 7.8, 1.5 Hz, 1H), 7.27 (td, J = 7.5, 1.5 Hz, 1H), 7.22 (td, J = 7.6, 1.5 Hz, 1H), 7.13 (td, J = 8.2, 6.7 Hz, 1H), 6.47 (d, J = 8.3 Hz, 1H), 6.40 (dd, J = 13.4, 8.1 Hz, 1H), 6.11 (s, 2H), 1.42 (s, 9H); $^{13}$C NMR (101 MHz, $CDCl_3$) δ 165.2 (d, $^{3}J_{CF}$ = 3.3 Hz), 162.4 (d, $^{1}J_{CF}$ = 242.7 Hz), 152.3 (d, $^{3}J_{CF}$ = 5.9 Hz), 144.4, 134.8, 132.7 (d, $^{3}J_{CF}$ = 13.5 Hz), 129.6, 126.89, 126.85, 126.7, 113.5 (d, $^{4}J_{CF}$ = 2.3 Hz), 103.6 (d, $^{2}J_{CF}$ = 12.9 Hz), 103.0 (d, $^{2}J_{CF}$ = 26.8 Hz), 34.8, 30.6; $^{19}$F NMR (376 MHz, $CDCl_3$) δ -111.8; HRMS (m/z): $[M]^+$ calcd for $C_{17}H_{20}ON_2F$ 287.1554; found 287.1551.

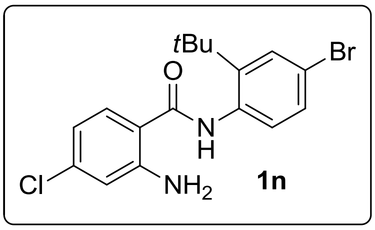


**2-Amino-N-(4-bromo-2-(*tert*-butyl)phenyl)-4-chlorobenzamide (1n)**

According to Procedure A, **1n** was obtained as a white solid. $^{1}$H NMR (400 MHz, $CDCl_3$) δ 7.62 (s, 1H), 7.55 (d, J = 2.1 Hz, 1H), 7.46 (d, J = 8.5 Hz, 1H), 7.40-7.36 (m, 1H), 6.77-6.53 (m, 2H), 5.75 (s, 2H), 1.41 (s, 9H); $^{13}$C NMR (101 MHz, $CDCl_3$) δ 167.2, 150.7, 145.7, 138.9, 134.1, 130.2, 130.1, 130.0, 128.1, 120.2, 117.12, 117.08, 113.7, 35.0, 30.7; HRMS (m/z): $[M]^+$ calcd for $C_{17}H_{19}ON_2BrCl$ 381.0364; found 381.0362.

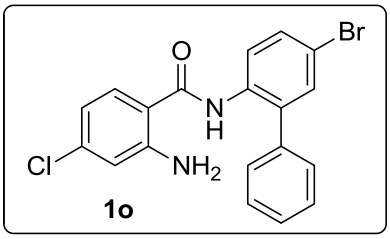


**2-Amino-*N*-(5-bromo-[1,1'-biphenyl]-2-yl)-4-chlorobenzamide (1o)**

According to Procedure A, **1o** was obtained as a white solid. $^{1}$H NMR (400 MHz, $CDCl_3$) δ 8.29 (d, J = 8.8 Hz, 1H), 7.74 (s, 1H), 7.53-7.45 (m, 4H), 7.44 (d, J = 2.4 Hz, 1H), 7.40-7.37 (m, 1H), 6.80 (d, J = 8.5 Hz, 1H), 6.65 (d, J = 2.0 Hz, 1H), 6.47 (dd, J = 8.5, 2.0 Hz, 1H), 5.70 (s, 2H); $^{13}$C NMR (101 MHz, $CDCl_3$) δ 166.5, 150.5, 138.8, 136.8, 134.6, 134.1, 132.8, 131.4, 129.6, 129.3, 128.9, 128.0, 123.0, 117.2, 117.0, 117.0, 113.9; HRMS (m/z): $[M]^+$ calcd for $C_{19}H_{15}ON_2BrCl$ 401.0051; found 401.0053.

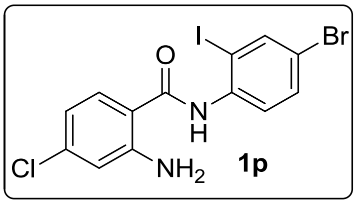

**1p**

**2-Amino-*N*-(4-bromo-2-iodophenyl)-4-chlorobenzamide (1p)**

According to Procedure A, **1p** was obtained as a light brown solid. $^{1}$H NMR (400 MHz, $CDCl_3$) δ 8.21 (d, J = 8.8 Hz, 1H), 8.04 (s, 1H), 7.94 (d, J = 2.2 Hz, 1H), 7.51-7.49 (m, 1H), 6.73 (d, J = 1.9 Hz, 1H), 6.71 (dd, J = 8.4, 2.0 Hz, 1H), 5.75 (brs, 2H); $^{13}$C NMR (101 MHz, $CDCl_3$) δ 166.7, 150.7, 140.7, 139.4, 137.6, 132.4, 128.6, 122.9, 117.6, 117.3, 117.2, 113.5, 91.0; HRMS (m/z): $[M]^+$ calcd for $C_{13}H_{10}ON_2BrCl$ 450.8704; found 450.8701.

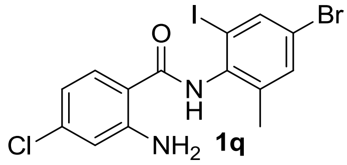

**1q**

**2-Amino-*N*-(4-bromo-2-iodo-6-methylphenyl)-4-chlorobenzamide (1q)**

According to Procedure A, **1q** was obtained as a white solid. $^{1}$H NMR (400 MHz, DMSO-$d_6$) δ 9.85 (s, 1H), 7.93 (d, J = 2.0 Hz, 1H), 7.78 (d, J = 8.6 Hz, 1H), 7.56 (d, J = 1.6 Hz, 1H), 6.81 (d, J = 2.1 Hz, 1H), 6.68 (s, 2H), 6.62 (dd, J = 8.5, 2.1 Hz, 1H), 2.20 (s, 3H); $^{13}$C NMR (101 MHz, DMSO-$d_6$) δ 166.9, 151.5, 140.1, 138.6, 137.9, 137.0, 133.0, 130.6, 120.6, 115.3, 114.5, 112.5, 103.3, 18.8; HRMS (m/z): $[M]^+$ calcd for $C_{14}H_{12}ON_2BrCl$ 464.8861; found 464.8859.

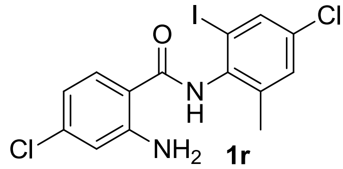

**1r**

**2-Amino-4-chloro-N-(4-chloro-2-iodo-6-methylphenyl)benzamide (1r)**

According to Procedure A, **1r** was obtained as a white solid. $^{1}$H NMR (400 MHz, DMSO-$d_6$) δ 9.86 (s, 1H), 7.81 (d, J = 2.2 Hz, 1H), 7.78 (d, J = 8.6 Hz, 1H), 7.44-7.43 (m, 1H), 6.81 (d, J = 2.1 Hz, 1H), 6.68 (s, 2H), 6.62 (dd, J = 8.5, 2.1 Hz, 1H), 2.21 (s, 3H); $^{13}$C NMR (101 MHz, DMSO-$d_6$) δ 166.9, 151.5, 139.7, 138.2, 137.0, 135.2, 132.1, 130.6, 130.1, 115.3, 114.5, 112.5, 102.8, 19.0; HRMS (m/z): $[M]^+$ calcd for $C_{14}H_{12}ON_2Cl_2I$ 420.9366; found 420.9362.

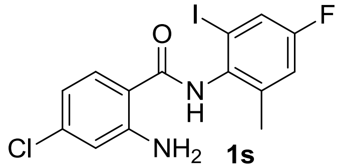

**1s**

**2-Amino-4-chloro-N-(4-fluoro-2-iodo-6-methylphenyl)benzamide (1s)**

According to Procedure A, **1s** was obtained as a white solid. $^{1}$H NMR (400 MHz, DMSO-$d_6$) δ 9.80 (s, 1H), 7.78 (d, J = 8.5 Hz, 1H), 7.62 (dd, J = 7.9, 2.9 Hz, 1H), 7.23 (dd, J = 9.4, 2.7 Hz, 1H), 6.81 (d, J =

2.1 Hz, 1H), 6.67 (s, 2H), 6.62 (dd, J = 8.5, 2.1 Hz, 1H), 2.22 (s, 3H); $^{13}$C NMR (101 MHz, DMSO-$d_6$) δ 167.0, 160.3 (d, $^1J_{CF}$ = 248.0 Hz), 151.5, 139.7 (d, $^3J_{CF}$ = 8.7 Hz), 137.0, 135.5 (d, $^4J_{CF}$ = 3.1 Hz), 130.6, 122.8 (d, $^2J_{CF}$ = 24.1 Hz), 117.0 (d, $^2J_{CF}$ = 21.8 Hz), 115.3, 114.5, 112.6, 102.1 (d, $^3J_{CF}$ = 8.9 Hz), 19.2; $^{19}$F NMR (376 MHz, DMSO-$d_6$) δ -114.7; HRMS (m/z): $[M]^+$ calcd for $C_{14}H_{12}ON_2ClFI$ 404.9661; found 404.9658.

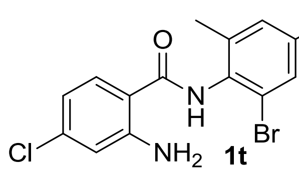

**1t**

**2-Amino-4-chloro-N-(2,4-dibromo-6-methylphenyl)benzamide (1t)**

According to Procedure A, **1t** was obtained as a white solid. $^1$H NMR (400 MHz, DMSO-$d_6$) δ 9.85 (s, 1H), 7.78 (d, J = 2.1 Hz, 1H), 7.76 (d, J = 8.6 Hz, 1H), 7.57 (d, J = 1.6 Hz, 1H), 6.82 (d, J = 2.1 Hz, 1H), 6.69 (s, 2H), 6.61 (dd, J = 8.5, 2.1 Hz, 1H), 2.21 (s, 3H); $^{13}$C NMR (101 MHz, DMSO-$d_6$) δ 167.0, 151.6, 141.1, 137.1, 135.3, 132.4, 132.1, 130.6, 124.8, 120.2, 115.4, 114.5, 112.3, 18.4; HRMS (m/z): $[M]^+$ calcd for $C_{14}H_{12}ON_2Br_2Cl$ 416.8999; found 416.8998.

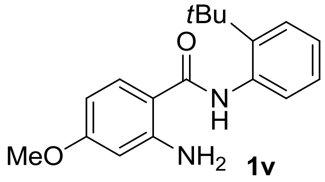

**1v**

**2-Amino-*N*-(2-(*tert*-butyl)phenyl)-4-Methoxybenzamide (1v)**

According to Procedure A, **1v** was obtained as a light brown solid. $^1$H NMR (400 MHz, $CDCl_3$) δ 7.63 (s, 1H), 7.57 (dd, J = 7.8, 1.3 Hz, 1H), 7.45-7.41 (m, 2H), 7.29-7.25 (m, 1H), 7.19 (td, J = 7.7, 1.5 Hz, 1H), 6.30 (dd, J = 8.8, 2.5 Hz, 1H), 6.19 (d, J = 2.5 Hz, 1H), 5.83 (brs, 2H), 3.80 (s, 3H), 1.43 (s, 9H); $^{13}$C NMR (101 MHz, $CDCl_3$) δ 167.7, 163.3, 151.9, 143.50, 135.45, 128.8, 128.6, 126.9, 126.8, 126.3, 108.9, 104.5, 100.9, 55.4, 34.8, 30.9; HRMS (m/z): $[M]^+$ calcd for $C_{18}H_{23}O_2N_2$ 299.1754; found 299.1752.

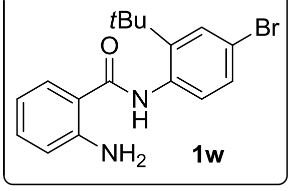

**1w**

**2-Amino-*N*-(4-bromo-2-(*tert*-butyl)phenyl)bezamide (1w)**

According to Procedure A, **1w** was obtained as a white solid. $^1$H NMR (400 MHz, $CDCl_3$) δ 7.71 (s, 1H), 7.60-7.43 (m, 3H), 7.38 (d, J = 7.5 Hz, 1H), 7.29-7.25 (m, 1H), 6.73 (d, J = 6.3 Hz, 2H), 5.64 (s, 2H), 1.42 (s, 9H); $^{13}$C NMR (101 MHz, $CDCl_3$) δ 167.8, 149.7, 145.6, 134.4, 133.1, 130.13, 130.05, 129.9, 126.8, 120.0, 117.9, 116.9, 115.4, 35.0, 30.7; HRMS (m/z): $[M]^+$ calcd for $C_{17}H_{20}ON_2Br$ 347.0754; found 347.0753.

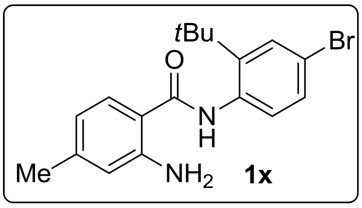


**2-Amino-*N*-(4-bromo-2-(*tert*-butyl)phenyl)-7 benzamide (1x)**

According to Procedure A, **1x** was obtained as a white solid. $^1$H NMR (400 MHz, $CDCl_3$) δ 7.67 (s, 1H), 7.54 (d, J = 2.2 Hz, 1H), 7.52 (d, J = 8.6 Hz, 1H), 7.38 (dd, J = 8.5, 2.2 Hz, 1H), 7.35 (d, J = 8.5 Hz, 1H), 6.54-6.53 (m, 2H), 5.64 (s, 2H), 2.29 (s, 3H), 1.42 (s, 9H); $^{13}$C NMR (101 MHz, $CDCl_3$) δ 167.7, 149.9, 145.5, 143.7, 134.6, 130.06, 130.02, 129.9, 126.8, 119.8, 118.21, 118.12, 112.7, 34.9, 30.7, 21.6; HRMS (m/z): $[M]^+$ calcd for $C_{18}H_{22}ON_2Br$ 361.0910; found 361.0905.

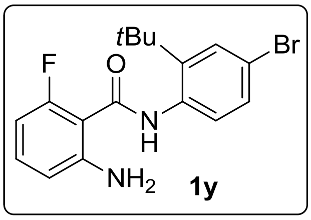


**2-Amino-*N*-(4-bromo-2-(*tert*-butyl)phenyl)-6-fluorobenzamide (1y)**

According to Procedure A, **1y** was obtained as a white solid. $^1$H NMR (400 MHz, $CDCl_3$) δ 8.33 (d, J = 17.9 Hz, 1H), 7.56 (d, J = 2.2 Hz, 1H), 7.45 (d, J = 8.5 Hz, 1H), 7.38 (dd, J = 8.5, 2.2 Hz, 1H), 7.15 (td, J = 8.2, 6.6 Hz, 1H), 6.49 (d, J = 8.3 Hz, 1H), 6.41 (ddd, J = 13.5, 8.1, 0.8 Hz, 1H), 6.12 (brs, 2H), 1.41 (s, 9H); $^{13}$C NMR (101 MHz, $CDCl_3$) δ 165.2 (d, $^3J_{CF}$ = 3.3 Hz), 162.5 (d, $^1J_{CF}$ = 242.8 Hz), 152.5 (d, $^3J_{CF}$ = 5.9 Hz), 146.5, 134.0, 132.9 (d, $^3J_{CF}$ = 13.5 Hz), 131.0, 130.2, 129.8, 120.4, 113.7 (d, $^4J_{CF}$ = 2.3 Hz), 103.3 (d, $^2J_{CF}$ = 14.6 Hz), 103.1 (d, $^2J_{CF}$ = 26.8 Hz), 35.0, 30.4; $^{19}$F NMR (376 MHz, $CDCl_3$) δ -111.7; HRMS (m/z): $[M]^+$ calcd for $C_{17}H_{19}ON_2BrF$ 365.06593; found 365.06589.

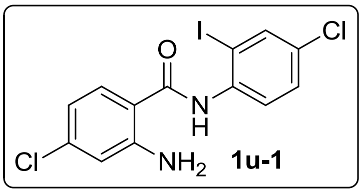


**2-Amino-4-chloro-N-(4-chloro-2-iodophenyl)benzamide (1u-1)**

According to Procedure A, **1u-1** was obtained as a white solid. $^1$H NMR (400 MHz, DMSO-$d_6$) δ 9.86 (s, 1H), 7.98 (d, J = 2.3 Hz, 1H), 7.76 (d, J = 8.5 Hz, 1H), 7.50 (dd, J = 8.5, 2.3 Hz, 1H), 7.43 (d, J = 8.5 Hz, 1H), 6.83 (d, J = 2.1 Hz, 1H), 6.71 (s, 2H), 6.62 (dd, J = 8.5, 2.1 Hz, 1H); $^{13}$C NMR (101 MHz, DMSO-$d_6$) δ 166.9, 151.4, 139.1, 137.6, 137.0, 131.1, 130.5, 129.4, 128.6, 115.3, 114.4, 112.4, 99.7; HRMS (m/z): $[M]^+$ calcd for $C_{13}H_{10}ON_2Cl_2I$ 406.9209; found 406.9210.

## Procedure B

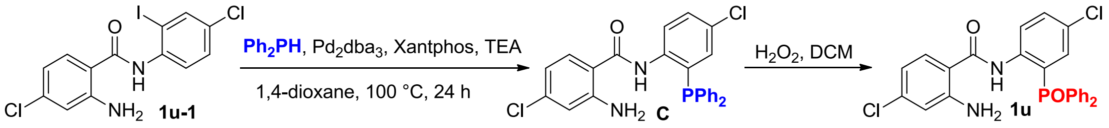

To a 25 mL Schlenk tube, **1u-1** (407 mg, 1.0 mmol), $Pd_2dba_3$ (92 mg, 0.1 mmol), Xantphos (135 mg, 0.2 mmol) and degased 1,4-dioxane (10 mL) was added under Ar. The mixture was degased for 10 min with Ar bubble, and then $Ph_2PH$ (280 mg, 1.5 mmol) and TEA (280 μL, 2.0 mmol) was added. The tube was sealed and the mixture was stirred at 100 ºC for 12 h. The reaction was concentrated under reduced pressure and the residue was purified through flash chromatography on silica gel to yield crude **C** contained about 20% oxidation byproduct **1u**. The above obtained mixture was directly dissolved in DCM, and then 30% $H_2O_2$ (227 mg, 2.0 mmol) was added. After stirred for 1 h, the mixture was washed with $H_2O$ (2 x 30 mL), saturated $Na_2S_2O_3$ solution (20 mL) and brine (20 mL). The organic layer was dried over $Na_2SO_4$ and concentrated under reduced pressure. The residue was purified through flash chromatography on silica gel to afford the desired product **1u** as a white solid.

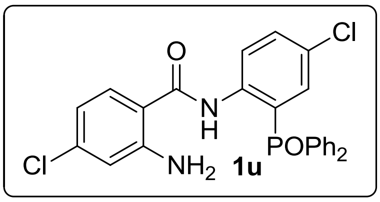


**2-Amino-4-chloro-N-(4-chloro-2-(diphenylphosphoryl)phenyl)benzamide (1u)**

$^1$H NMR (400 MHz, $CDCl_3$) δ 11.71 (s, 1H), 8.66 (dd, J = 9.0, 4.8 Hz, 1H), 7.76 (d, J = 8.6 Hz, 1H), 7.67-7.59 (m, 6H), 7.53-7.48 (m, 5H), 6.96 (dd, J = 14.1, 2.5 Hz, 1H), 6.67 (dd, J = 8.6, 2.0 Hz, 1H), 6.64 (d, J = 2.0 Hz, 1H), 5.88 (s, 2H); $^{13}$C NMR (101 MHz, $CDCl_3$) δ 167.3, 151.0, 143.2 (d, $^2J_{CP}$ = 2.8 Hz), 138.9, 133.3 (d, $^4J_{CP}$ = 2.1 Hz), 133.0 (d, $^4J_{CP}$ = 2.8 Hz), 132.2 (d, $^2J_{CP}$ = 10.3 Hz), 132.1 (d, $^2J_{CP}$ = 11.9 Hz), 130.6 (d, $^1J_{CP}$ = 106.3 Hz), 129.5, 129.1 (d, $^3J_{CP}$ = 12.5 Hz), 128.0 (d, $^3J_{CP}$ = 16.2 Hz), 123.7 (d, $^3J_{CP}$ = 8.0 Hz), 119.9 (d, $^1J_{CP}$ = 98.0 Hz), 117.2, 116.7, 113.2; $^{31}$P NMR (162 MHz, $CDCl_3$) δ 36.6; HRMS (m/z): $[M]^+$ calcd for $C_{25}H_{20}O_2N_2Cl_2P$ 481.0634; found 481.0633.

## Supplementary Note 3

### General experimental procedures for the Brønsted acid catalyzed reactions

**Experimental procedure for the synthesis of *racemic* 3, 4a-4l**

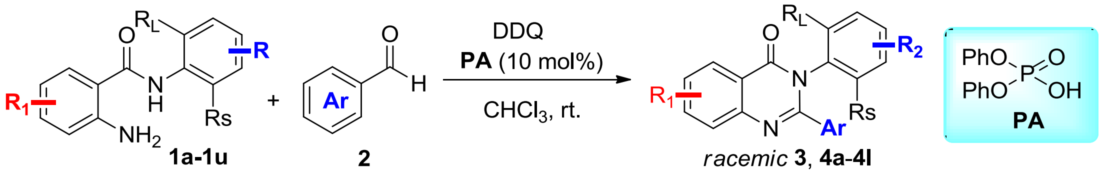


To a 2.0 mL vail, **1a-1u** (0.02 mmol) substrate, ArCHO (0.03 mmol), DDQ (0.03 mmol), diphenyl phosphate (0.50 mg, 0.002 mmol) and 0.5 mL $CHCl_3$ was added. The reaction mixture was stirred at room temperature and monitored by TLC. After completion, the mixture was purified by preparative TLC on silica gel to yield the corresponding product *racemic* **3**, **4a-4l**.

**Experimental procedure for the asymmetric synthesis of 3, 4a-4l**

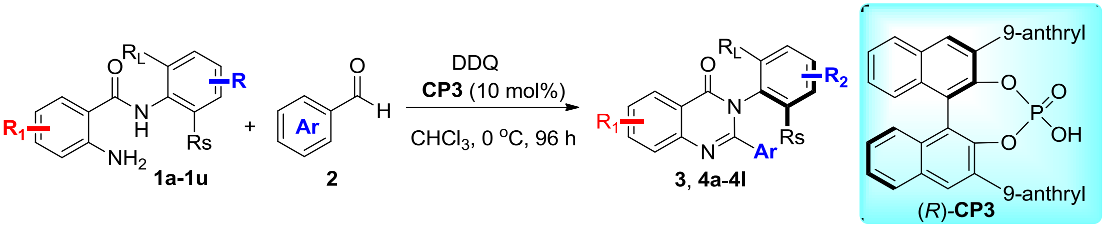


To a dry Schlenk tube (10 mL), 400 mg activated 4 Å MS (molecular sieves) was added and then the MS was reactivated at 300 ºC under reduced pressure for 15 min. After the tube was cooled down, **1a-1u** (0.10 mmol), **CP3** (7.0 mg, 0.01 mmol), DDQ (31.8 mg, 0.14 mmol) and anhydrous $CHCl_3$ (4.0 mL) was added under Ar. The resulting mixture was stirred for 10 min at 0 °C, and then aromatic aldehyde **2** (0.2 mmol) was added in one portion. After stirred for 96 h at 0 °C, the mixture was directly purified through flash column chromatography on silica gel (gradient elution with PE/EA) to give the pure product.

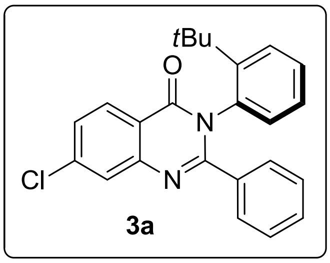


**3a**

**(*aR*)-3-(2-(*tert*-Butyl)phenyl)-7-chloro-2-phenylquinazolin-4(*3H*)-one (3a)**

According to Procedure D, **3a** was obtained in 96% yield and 96% ee as a white solid. The ee was determined by chiral stationary phase HPLC analysis [Daicel CHIRALCEL OD-3, hexane/isopropanol = 90/10, 1.0 mL/min, T = 25 °C, λ = 254 nm, $t_R$ (major) = 13.9 min, $t_R$ (minor) = 19.5 min]. $^1$H NMR (400 MHz, $CDCl_3$) δ 8.29 (d, J = 8.5 Hz, 1H), 7.83 (d, J = 1.9 Hz, 1H), 7.50-7.45 (m, 2H), 7.40-7.37 (m, 2H), 7.35-7.30 (m, 1H), 7.30-7.26 (m, 1H), 7.23-7.19 (m, 2H), 7.05 (dd, J = 7.9, 1.4 Hz, 1H), 1.11 (s, 9H); $^{13}$C NMR (101 MHz, $CDCl_3$) δ 162.9, 156.8, 148.8, 146.5, 141.0, 135.0, 134.2, 132.1, 130.8, 129.95, 129.88, 129.4, 128.9, 127.9, 127.4, 126.6, 119.3, 36.6, 31.8; HRMS (m/z): $[M]^+$ calcd for

$C_{24}H_{22}ON_2Cl$ 389.1415; found 389.1403.

*Chiral HPLC spectrum of racemic **3a***

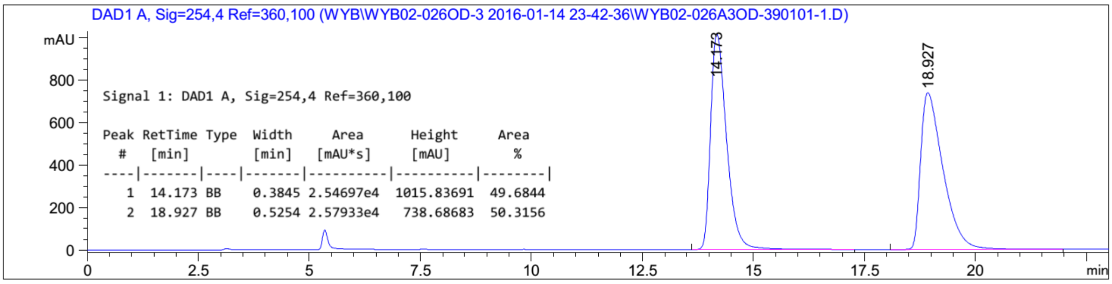


*Chiral HPLC spectrum of **3a***

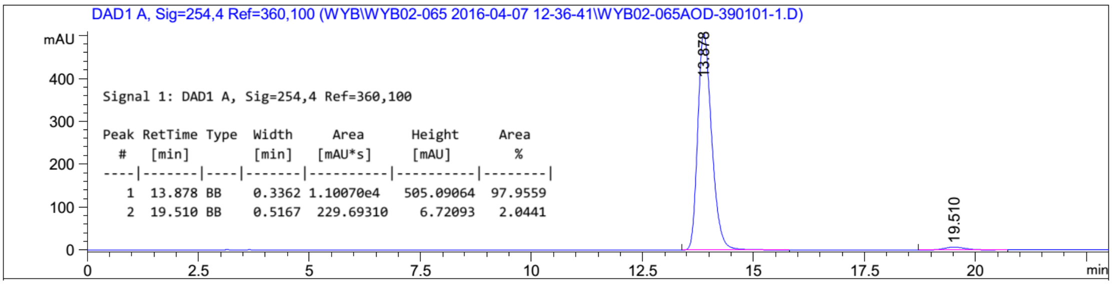


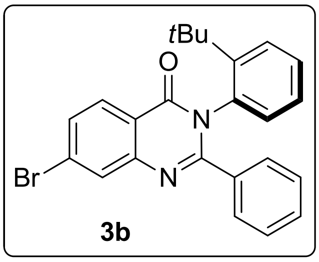


**(*aR*)-7-Bromo-3-(2-(*tert*-butyl)phenyl)-2-phenylquinazolin-4(*3H*)-one (3b)**

According to Procedure D, **3b** was obtained in 95% yield and 97% ee as a white solid. The ee was determined by chiral stationary phase HPLC analysis [Daicel CHIRALCEL OD-3, hexane/isopropanol = 90/10, 1.0 mL/min, T = 25 °C, λ = 254 nm, $t_R$ (major) = 14.8 min, $t_R$ (minor) = 19.5 min]. $^1$H NMR (400 MHz, $CDCl_3$) δ 8.20 (d, J = 8.5 Hz, 1H), 8.02 (d, J = 1.8 Hz, 1H), 7.63 (dd, J = 8.5, 1.9 Hz, 1H), 7.46 (dd, J = 8.2, 1.4 Hz, 1H), 7.38-7.36 (m, 2H), 7.32 (dd, J = 7.2, 1.5 Hz, 1H), 7.30-7.26 (m, 1H), 7.23-7.19 (m, 3H), 7.05 (dd, J = 7.9, 1.4 Hz, 1H), 1.11 (s, 9H); $^{13}$C NMR (101 MHz, $CDCl_3$) δ 163.1, 156.7, 148.8, 146.5, 135.1, 134.2, 132.1, 130.8, 130.7, 130.0, 129.9, 129.5, 129.4, 128.9, 127.9, 126.6, 119.7, 36.6, 31.8; HRMS (m/z): $[M]^+$ calcd for $C_{24}H_{22}ON_2Br$ 433.0910; found 433.0898.

*Chiral HPLC spectrum of racemic **3b***

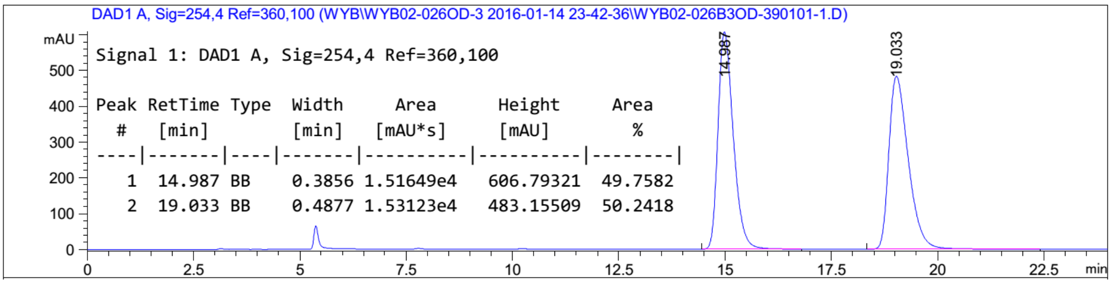


*Chiral HPLC spectrum of **3b***

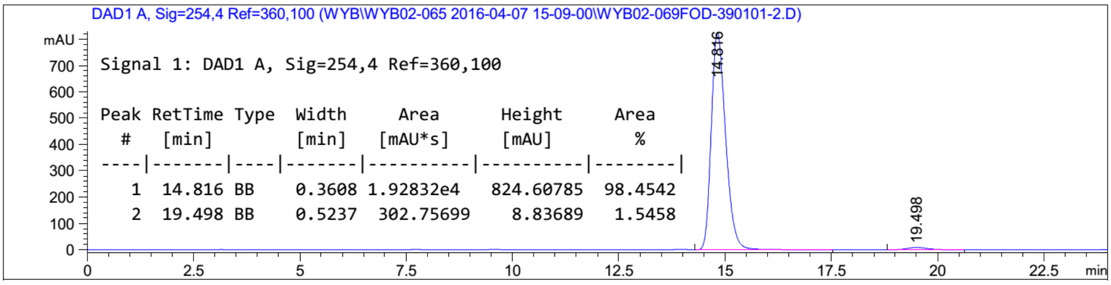


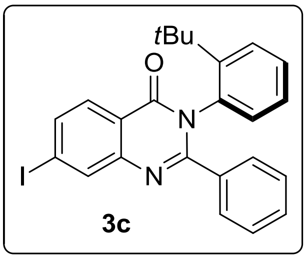


**(*aR*)-3-(2-(*tert*-Butyl)phenyl)-7-iodo-2-phenylquinazolin-4(*3H*)-one (3c)**

According to Procedure D, **3c** was obtained in 95% yield and 94% ee as a white solid. The ee was determined by chiral stationary phase HPLC analysis [Daicel CHIRALCEL OD-3, hexane/isopropanol = 90/10, 1.0 mL/min, T = 25 °C, λ = 254 nm, $t_R$ (major) = 14.9 min, $t_R$ (minor) = 18.6 min]. $^1$H NMR (400 MHz, $CDCl_3$) δ 8.26 (d, J = 1.5 Hz, 1H), 8.03 (d, J = 8.4 Hz, 1H), 7.83 (dd, J = 8.4, 1.5 Hz, 1H), 7.46 (dd, J = 8.2, 1.2 Hz, 1H), 7.38-7.36 (m, 2H), 7.34-7.29 (m, 1H), 7.27-7.25 (m, 1H), 7.22-7.18 (m, 3H), 7.04 (dd, J = 7.9, 1.3 Hz, 1H), 1.11 (s, 9H); $^{13}$C NMR (101 MHz, $CDCl_3$) δ 163.3, 156.5, 148.6, 146.5, 137.0, 136.3, 135.1, 134.3, 132.1, 130.8, 130.0, 129.9, 129.4, 128.6, 127.9, 126.6, 120.2, 102.2, 36.6, 31.9; HRMS (m/z): $[M]^+$ calcd for $C_{24}H_{22}ON_2I$ 481.0771; found 481.0756.

*Chiral HPLC spectrum of racemic **3c***

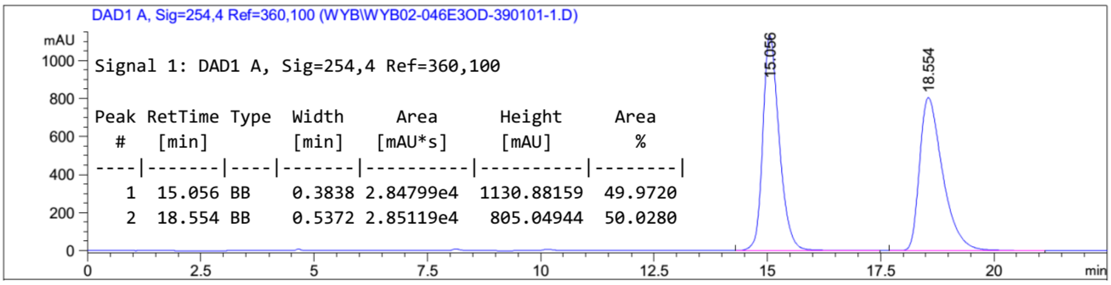


*Chiral HPLC spectrum of **3c***

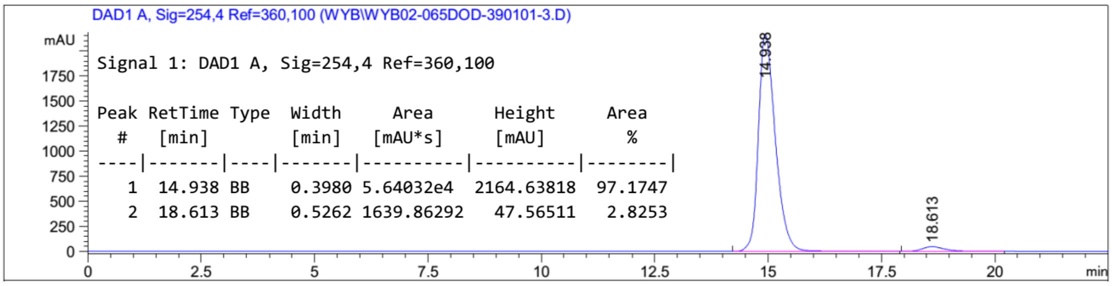

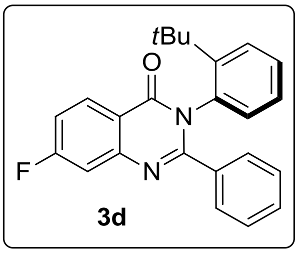


**(*aR*)-3-(2-(*tert*-butyl)phenyl)-7-fluoro-2-phenylquinazolin-4(3*H*)-one (3d)**

According to Procedure D, **3d** was obtained in 99% yield and 94% ee as a colorless oil. The ee was determined by chiral stationary phase HPLC analysis [Daicel CHIRALCEL OD-3, hexane/isopropanol = 90/10, 1.0 mL/min, T = 25 °C, λ = 254 nm, $t_R$ (major) = 12.3 min, $t_R$ (minor) = 20.8 min]. $^1$H NMR (400 MHz, $CDCl_3$) δ 8.37 (dd, J = 8.8, 6.1 Hz, 1H), 7.49-7.45 (m, 2H), 7.39-7.37 (m, 2H), 7.34-7.29 (m, 1H), 7.28-7.18 (m, 5H), 7.05 (dd, J = 7.8, 1.4 Hz, 1H), 1.12 (s, 9H); $^{13}$C NMR (101 MHz, $CDCl_3$) δ 166.9 (d, $^1J_{CF}$ = 255.5 Hz), 162.8, 156.8, 150.0 (d, $^3J_{CF}$ = 13.1 Hz), 146.6, 135.2, 134.3, 132.2, 130.8, 130.2 (d, $^3J$ = 10.6 Hz), 130.0, 129.8, 129.3, 127.9, 126.6, 117.7(d, $^4J_{CF}$ = 1.9 Hz), 116.1(d, $^2J_{CF}$ = 23.7 Hz), 113.2(d, $^2J_{CF}$ = 22.0 Hz), 36.6, 31.9; $^{19}$F NMR (376 MHz, $CDCl_3$) δ -102.9. HRMS (m/z): $[M]^+$ calcd for $C_{24}H_{22}ON_2F$ 373.1711; found 536. 373.1700.

*Chiral HPLC spectrum of racemic **3d***

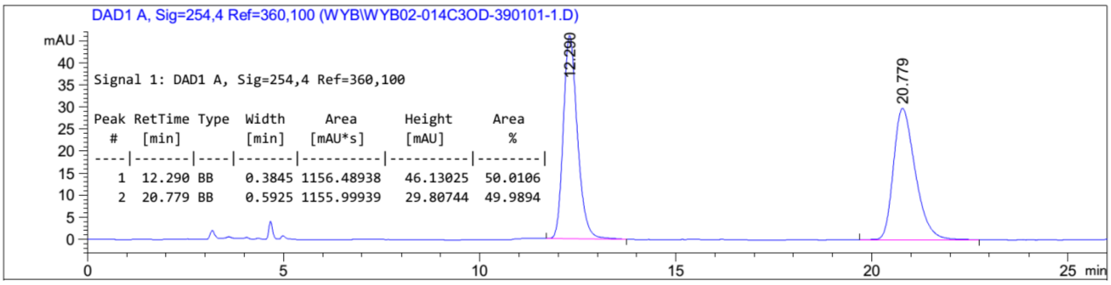


*Chiral HPLC spectrum of **3d***

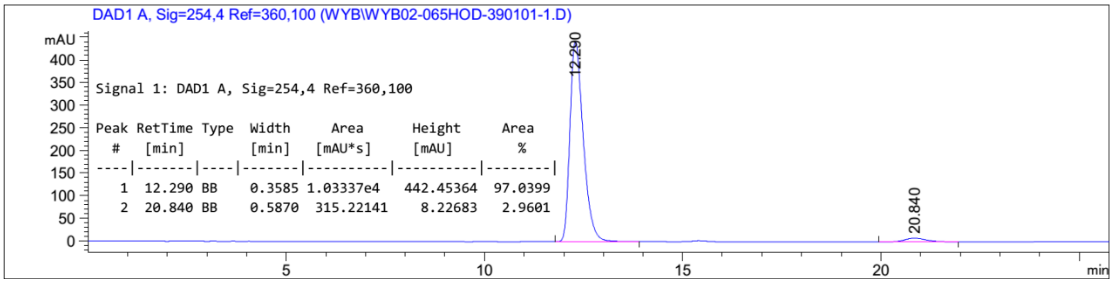


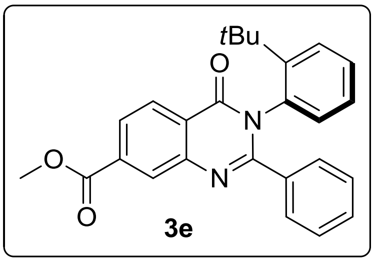


**(*aR*)-Methyl 3-(2-(*tert*-butyl)phenyl)-4-oxo-2-phenyl-3,4-dihydroquinazoline-7-carboxylate (3e)**

According to Procedure D, **3e** was obtained in 90% yield and 91% ee as a white solid. The ee was determined by chiral stationary phase HPLC analysis [Daicel CHIRALCEL AD-3, hexane/isopropanol

= 90/10, 1.0 mL/min, T = 25 °C, λ = 254 nm, $t_R$ (major) = 16.5 min, $t_R$ (minor) = 21.7 min]. $^1$H NMR (400 MHz, $CDCl_3$) δ 8.52 (d, J = 1.3 Hz, 1H), 8.41 (d, J = 8.3 Hz, 1H), 8.13 (dd, J = 8.3, 1.5 Hz, 1H), 7.47 (dd, J = 8.2, 1.3 Hz, 1H), 7.41-7.39 (m, 2H), 7.35-7.31 (m, 1H), 7.30-7.26 (m, 1H), 7.23 (s, 1H), 7.20 (d, J = 7.1 Hz, 2H), 7.06 (dd, J = 7.9, 1.3 Hz, 1H), 3.99 (s, 3H), 1.12 (s, 9H); $^{13}$C NMR (101 MHz, $CDCl_3$) δ 166.2, 163.1, 156.3, 147.7, 146.5, 135.9, 135.1, 134.3, 132.1, 130.8, 130.0, 129.9, 129.4, 127.9, 127.7, 127.3, 126.7, 123.8, 52.7, 36.7, 31.8; HRMS (m/z): $[M]^+$ calcd for $C_{26}H_{25}O_3N_2$ 413.1860; found 413.1846.

*Chiral HPLC spectrum of racemic **3e***

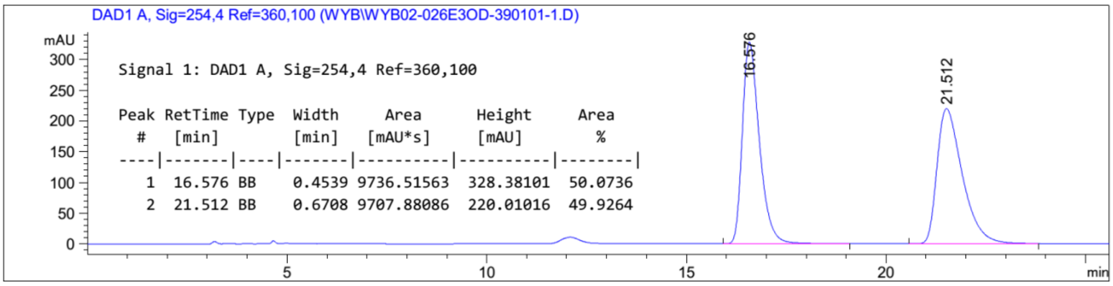


*Chiral HPLC spectrum of **3e***

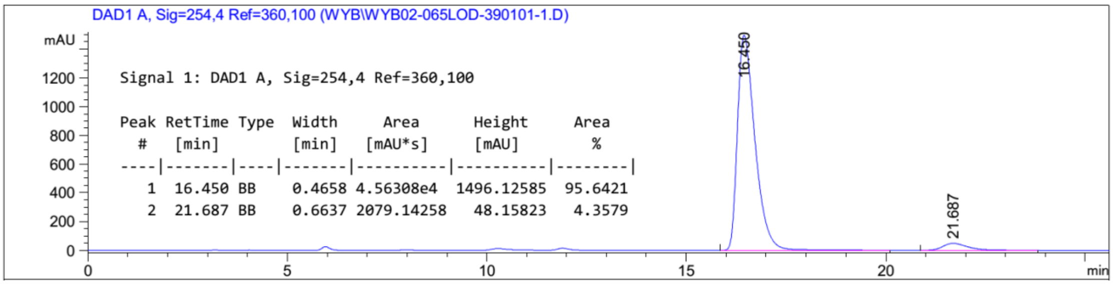


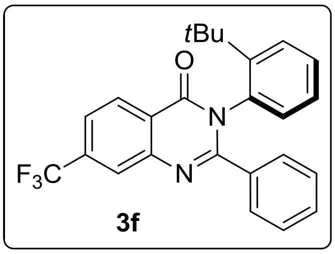


**(*aR*)-3-(2-(*tert*-Butyl)phenyl)-2-phenyl-7-(trifluoromethyl)quinazolin-4(3*H*)-one (3f)**

According to Procedure D at r.t, **3f** was obtained in 93% yield and 85% ee as a colorless oil. The ee was determined by chiral stationary phase HPLC analysis [Daicel CHIRALCEL OD-3, hexane/isopropanol = 90/10, 1.0 mL/min, T = 25 °C, λ = 254 nm, $t_R$ (minor) = 6.7 min, $t_R$ (major) = 8.2 min]. $^1$H NMR (400 MHz, $CDCl_3$) δ 8.47 (d, J = 8.3 Hz, 1H), 8.13 (s, 1H), 7.73 (dd, J = 8.3, 1.4 Hz, 1H), 7.48 (dd, J = 8.2, 1.4 Hz, 1H), 7.42-7.39 (m, 2H), 7.36-7.32 (m, 1H), 7.31-7.27 (m, 1H), 7.24-7.20 (m, 2H), 7.05 (dd, J = 7.9, 1.4 Hz, 1H), 1.12 (s, 9H); $^{13}$C NMR (101 MHz, $CDCl_3$) δ 162.8, 156.9, 147.8, 146.5, 136.4 (q, $^2J_{CF}$ = 32.9 Hz), 134.9, 134.1, 132.0, 130.9, 130.02, 130.00, 129.5, 128.6, 128.0, 126.7, 125.5 (q, $^3J_{CF}$ = 4.0 Hz), 123.6 (q, $^1J_{CF}$ = 273.1 Hz), 123.24 (q, $^3J_{CF}$ = 3.3 Hz), 123.16, 36.7, 31.8; $^{19}$F NMR (376 MHz, $CDCl_3$) δ -63.2; HRMS (m/z): $[M]^+$ calcd for $C_{25}H_{22}ON_2F_3$ 423.1679; found 423.1664.

*Chiral HPLC spectrum of racemic **3f***

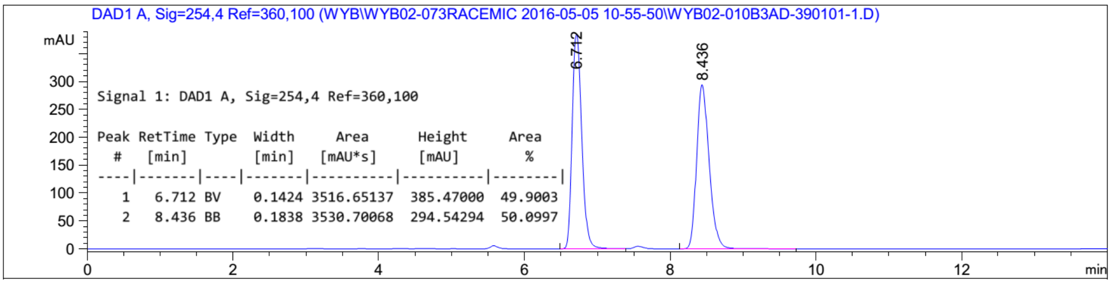


*Chiral HPLC spectrum of **3f***

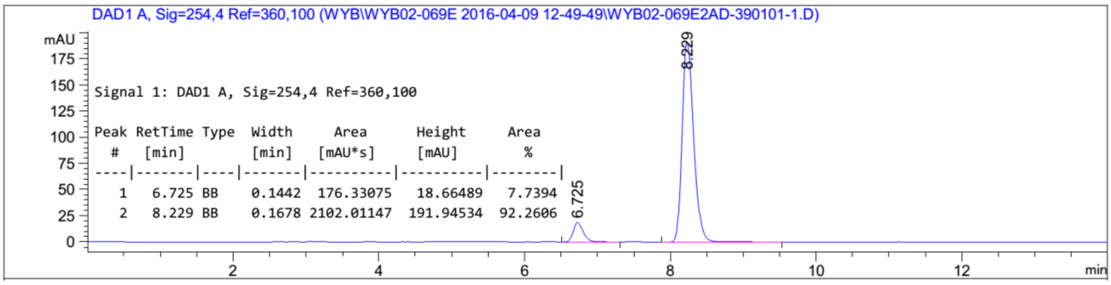


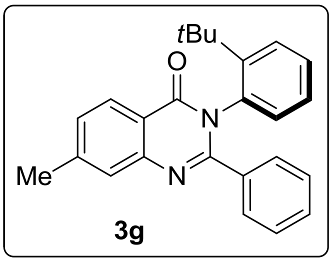


**(*aR*)-3-(2-(*tert*-Butyl)phenyl)-7-methyl-2-phenylquinazolin-4(*3H*)-one (3g)**

According to Procedure D with anhydrous *n*-Hexane/$CHCl_3$ = 2:1 as solvent, **3g** was obtained in 98% yield and 88% ee as a colorless oil. The ee was determined by chiral stationary phase HPLC analysis [Daicel CHIRALCEL OD-3, hexane/isopropanol = 90/10, 1.0 mL/min, T = 25 °C, λ = 254 nm, $t_R$ (major) = 12.1 min, $t_R$ (minor) = 20.2 min]. $^1$H NMR (400 MHz, $CDCl_3$) δ 8.24 (d, J = 8.1 Hz, 1H), 7.63 (s, 1H), 7.45 (dd, J = 8.2, 1.4 Hz, 1H), 7.40-7.34 (m, 3H), 7.32-7.27 (m, 1H), 7.27-7.23 (m, 1H), 7.21-7.16 (m, 3H), 7.05 (dd, J = 7.8, 1.4 Hz, 1H), 2.54 (s, 3H), 1.12 (s, 9H); $^{13}$C NMR (101 MHz, $CDCl_3$) δ 163.4, 155.6, 148.0, 146.6, 145.8, 135.6, 134.6, 132.3, 130.7, 130.0, 129.5, 129.2, 128.9, 127.8, 127.7, 127.2, 126.5, 118.6, 36.6, 31.9, 22.1. HRMS (m/z): $[M]^+$ calcd for $C_{25}H_{25}ON_2$ 369.1961; found 369.1950.

*Chiral HPLC spectrum of racemic **3g***

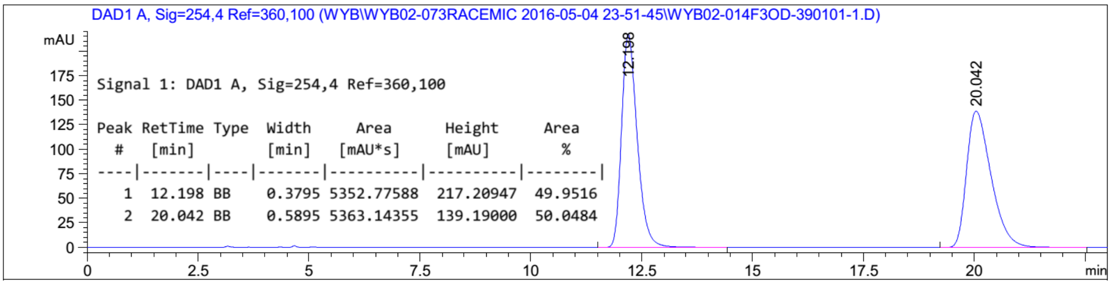


*Chiral HPLC spectrum of **3g***

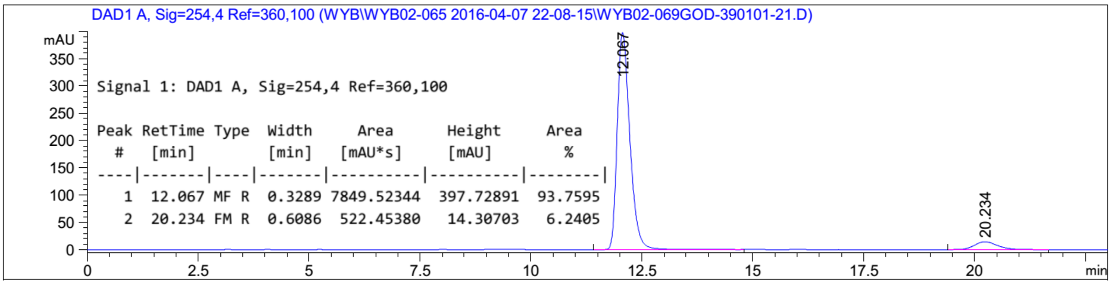


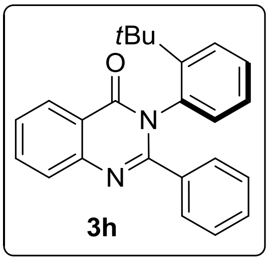


**(*aR*)-3-(2-(*tert*-Butyl)phenyl)-2-phenylquinazolin-4(*3H*)-one (3h)**

According to Procedure D with anhydrous *n*-Hexane/$CHCl_3$ = 2:1 as solvent, **3h** was obtained in 96% yield and 85% ee as a white solid. The product was recrystallized from PE and the filtrate afforded **3h** in 65% yield and 97% ee as a white solid. The ee was determined by chiral stationary phase HPLC analysis [Daicel CHIRALCEL OD-3, hexane/isopropanol = 90/10, 1.0 mL/min, T = 25 °C, $\lambda$ = 254 nm, $t_R$ (major) = 11.2 min, $t_R$ (minor) = 17.3 min]. $^1$H NMR (400 MHz, $CDCl_3$) $\delta$ 8.38-8.35 (m, 1H), 7.85-7.79 (m, 2H), 7.53 (ddd, J = 8.2, 6.5, 1.8 Hz, 1H), 7.46 (dd, J = 8.2, 1.4 Hz, 1H), 7.41-7.38 (m, 2H), 7.33-7.29 (m, 1H), 7.28-7.24 (m, 1H), 7.22-7.17 (m, 2H), 7.05 (dd, J = 7.8, 1.4 Hz, 1H), 1.13 (s, 9H); $^{13}$C NMR (101 MHz, $CDCl_3$) $\delta$ 163.5, 155.5, 147.8, 146.6, 135.4, 134.8, 134.6, 132.2, 130.7, 130.0, 129.6, 129.2, 127.9, 127.8, 127.4, 127.3, 126.6, 120.9, 36.6, 31.9; HRMS (m/z): $[M]^+$ calcd for $C_{24}H_{23}ON_2$ 355.1805; found 355.1804.

*Chiral HPLC spectrum of racemic **3h***

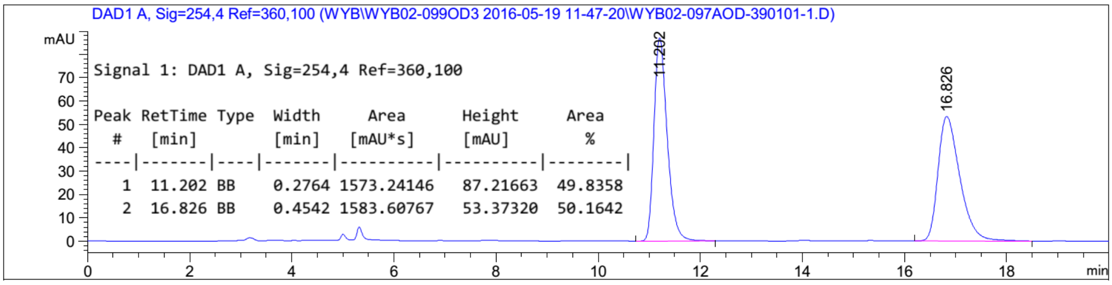


*Chiral HPLC spectrum of **3h***

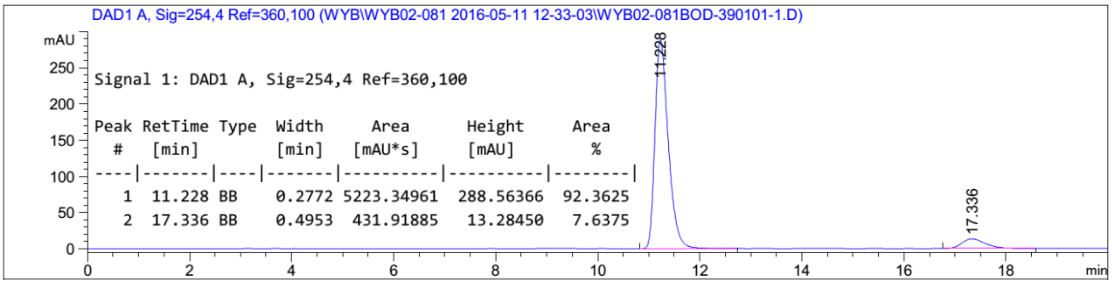


*Chiral HPLC spectrum of **3h***

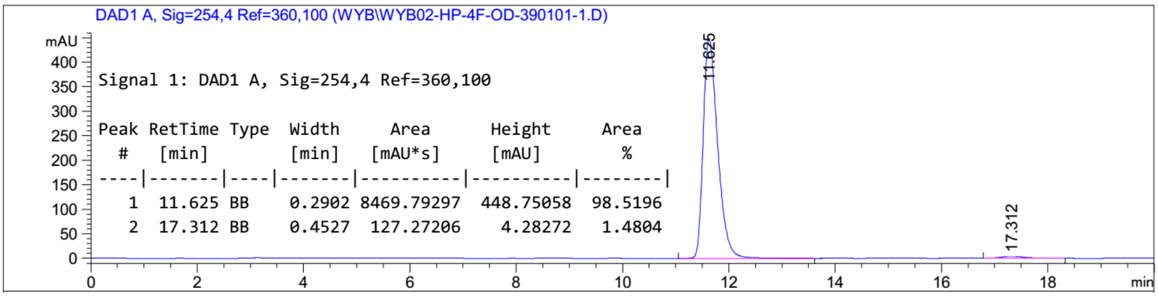


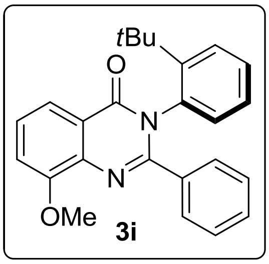


**(*aR*)-3-(2-(*tert*-Butyl)phenyl)-8-methoxy-2-phenylquinazolin-4(3*H*)-one (3i)**

According to Procedure D, **3i** was obtained in 97% yield and 90% ee as a colorless oil. The ee was determined by chiral stationary phase HPLC analysis [Daicel CHIRALCEL OD-3, hexane/isopropanol = 90/10, 1.0 mL/min, T = 25 °C, $\lambda$ = 254 nm, $t_R$ (major) = 15.9 min, $t_R$ (minor) = 21.5 min]. $^1$H NMR (400 MHz, $CDCl_3$) $\delta$ 7.94 (dd, J = 8.0, 1.2 Hz, 1H), 7.50-7.43 (m, 2H), 7.42-7.38 (m, 2H), 7.33-7.28 (m, 1H), 7.27-7.25 (m, 1H), 7.23-7.14 (m, 4H), 7.04 (dd, J = 7.9, 1.5 Hz, 1H), 4.03 (s, 3H), 1.12 (s, 9H); $^{13}$C NMR (101 MHz, $CDCl_3$) $\delta$ 163.4, 154.9, 154.7, 146.5, 138.5, 135.6, 134.6, 132.2, 130.7, 130.3, 129.5, 129.2, 127.7, 127.6, 126.5, 122.1, 118.6, 114.5, 56.4, 36.6, 31.9; HRMS (m/z): $[M]^+$ calcd for $C_{25}H_{25}O_2N_2$ 385.1911; found 385.1910.

*Chiral HPLC spectrum of racemic **3i***

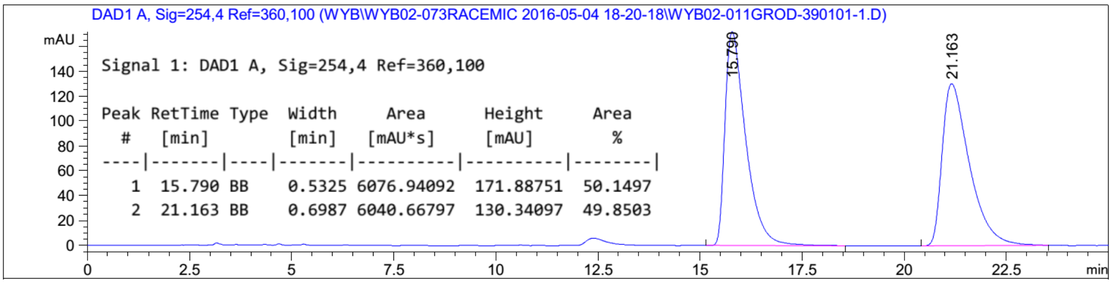


*Chiral HPLC spectrum of **3i***

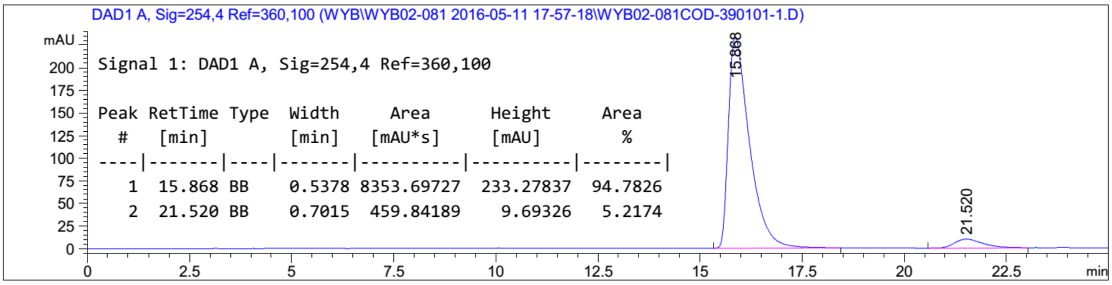

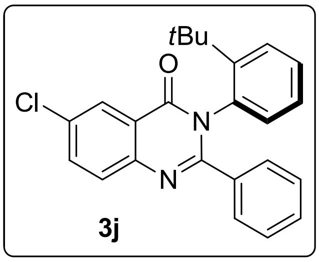


**(*aR*)-3-(2-(*tert*-butyl)phenyl)-6-chloro-2-phenylquinazolin-4(3*H*)-one (3j)**

According to Procedure D, **3j** was obtained in 94% yield and 90% ee as a colorless oil. The ee was determined by chiral stationary phase HPLC analysis [Daicel CHIRALCEL OD-3, hexane/isopropanol = 90/10, 1.0 mL/min, T = 25 °C, λ = 254 nm, $t_R$ (major) = 8.4 min, $t_R$ (minor) = 10.3 min]. $^1$H NMR (400 MHz, $CDCl_3$) δ 8.32 (d, J = 2.2 Hz, 1H), 7.78 (d, J = 8.6 Hz, 1H), 7.74 (dd, J = 8.7, 2.3 Hz, 1H), 7.46 (dd, J = 8.2, 1.4 Hz, 1H), 7.39-7.37 (m, 2H), 7.34-7.30 (m, 1H), 7.29-7.25 (m, 1H), 7.22-7.18 (m, 3H), 7.04 (dd, J = 7.9, 1.4 Hz, 1H), 1.12 (s, 9H); $^{13}$C NMR (101 MHz, $CDCl_3$) δ 162.5, 155.8, 146.5, 146.4, 135.3, 135.1, 134.3, 133.1, 132.1, 130.8, 130.0, 129.8, 129.6, 129.4, 127.9, 126.69, 126.66, 122.0, 36.7, 31.8; HRMS (m/z): $[M]^+$ calcd for $C_{24}H_{22}ON_2Cl$ 389.1415; found 389.1400.

*Chiral HPLC spectrum of racemic **3j***

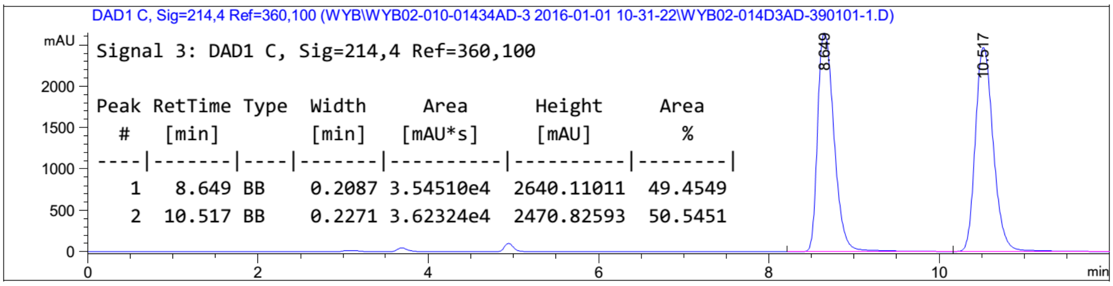


*Chiral HPLC spectrum of **3j***

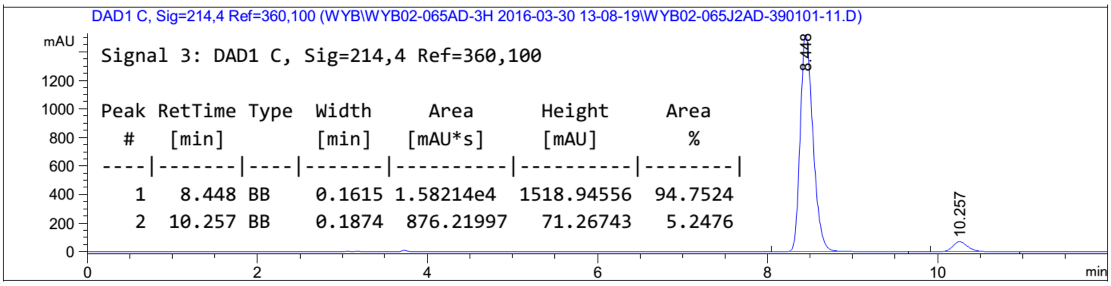


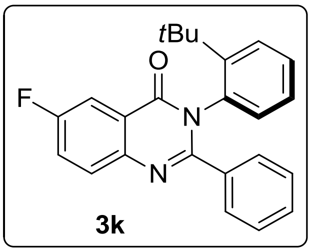


**(*aR*)-3-(2-(*tert*-butyl)phenyl)-6-fluoro-2-phenylquinazolin-4(3*H*)-one (3k)**

According to Procedure D, **3k** was obtained in 97% yield and 94% ee as a colorless oil. The ee was determined by chiral stationary phase HPLC analysis [Daicel CHIRALCEL OD-3, hexane/isopropanol = 90/10, 1.0 mL/min, T = 25 °C, λ = 254 nm, $t_R$ (major) = 7.5 min, $t_R$ (minor) = 8.8 min]. $^1$H NMR (400

MHz, $CDCl_3$) δ 7.99 (dd, J = 8.4, 3.0 Hz, 1H), 7.84 (dd, J = 9.0, 4.8 Hz, 1H), 7.55-7.50 (m, 1H), 7.46 (dd, J = 8.2, 1.4 Hz, 1H), 7.140-7.37 (m, 2H), 7.34-7.29 (m, 1H), 7.29-7.25 (m, 1H), 7.22-7.18 (m, 3H), 7.04 (dd, J = 7.9, 1.4 Hz, 1H), 1.12 (s, 9H); $^{13}$C NMR (101 MHz, $CDCl_3$) δ 162.8 (d, J = 3.5 Hz), 161.31 (d, J = 248.8 Hz), 154.9 (d, J = 2.4 Hz), 146.6, 144.6 (d, J = 1.9 Hz), 135.2, 134.3, 132.1, 130.8, 130.4 (d, J = 8.2 Hz), 130.0, 129.7, 129.4, 127.9, 126.6, 123.40 (d, J = 24.2 Hz), 122.2 (d, J = 8.6 Hz), 112.2 (d, J = 23.6 Hz), 36.7, 31.8; $^{19}$F NMR (376 MHz, $CDCl_3$) δ -112.1; HRMS (m/z): $[M]^+$ calcd for $C_{24}H_{22}ON_2F$ 373.1711; found 536. 373.1700.

*Chiral HPLC spectrum of racemic **3k***

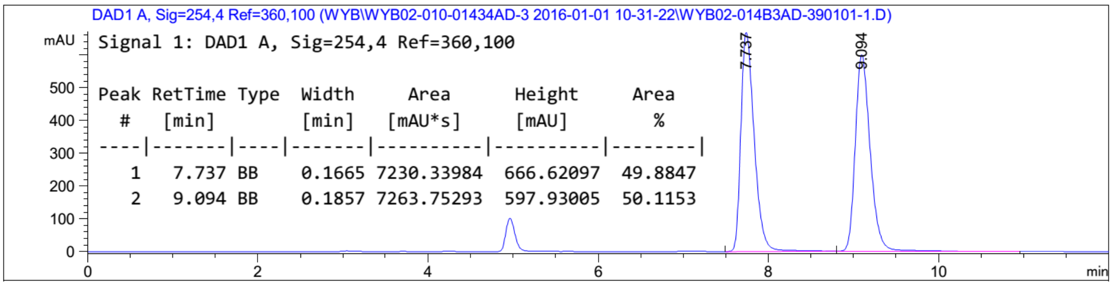


*Chiral HPLC spectrum of **3k***

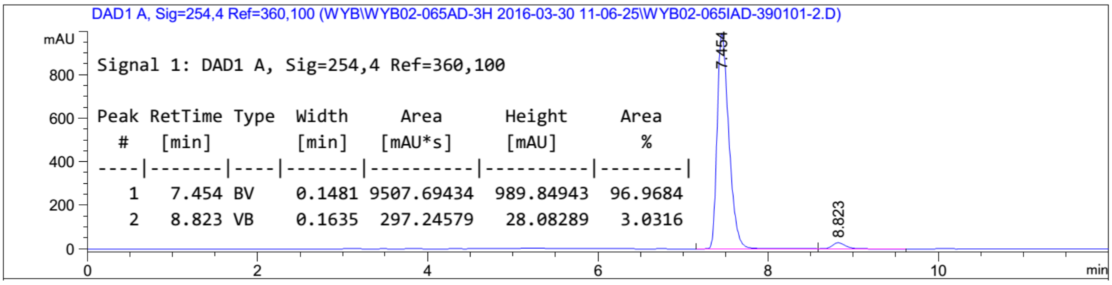


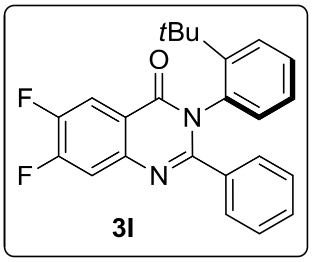


**3l**

**(*aR*)-3-(2-(*tert*-Butyl)phenyl)-6,7-difluoro-2-phenylquinazolin-4(*3H*)-one (3l)**

According to Procedure D, **3l** was obtained in 98% yield and 97% ee as a colorless oil. The ee was determined by chiral stationary phase HPLC analysis [Daicel CHIRALCEL OD-3, hexane/isopropanol = 90/10, 1.0 mL/min, T = 25 °C, λ = 254 nm, $t_R$ (major) = 6.0 min, $t_R$ (minor) = 6.8 min].$^1$H NMR (400 MHz, $CDCl_3$) δ 8.11 (dd, J = 9.9, 8.6 Hz, 1H), 7.60 (dd, J = 10.7, 7.0 Hz, 1H), 7.46 (dd, J = 8.2, 1.4 Hz, 1H), 7.37 (dd, J = 5.3, 3.3 Hz, 2H), 732 (dd, J = 7.3, 1.5 Hz, 1H), 7.30-7.26 (m, 1H), 7.23-7.17 (m, 3H), 7.03 (dd, J = 7.9, 1.4 Hz, 1H), 1.11 (s, 9H); $^{13}$C NMR (101 MHz, $CDCl_3$) δ 162.2 (d, $^4J_{CF}$ = 2.8 Hz), 156.3 (d, $^5J_{CF}$ = 2.3 Hz), 155.3 (dd, $^{1,2}J_{CF}$ = 244.2, 26.7 Hz), 150.2 (dd, $^{1,2}J_{CF}$ = 253.4, 14.3 Hz), 146.5, 145.7 (dd, $^{3,4}J_{CF}$ = 11.4, 2.3 Hz), 135.0, 134.1, 132.1, 130.8, 129.95, 129.93, 129.5, 127.9, 126.7, 117.9 (dd, $^{3,4}J_{CF}$ = 6.6, 2.0 Hz), 115.7 (d, $^2J_{CF}$ = 17.9 Hz), 114.8 (dd, $^2J_{FF}$ = 19.2, 2.2 Hz), 36.7, 31.8; $^{19}$F NMR (376 MHz, $CDCl_3$) δ -126.0 (d, $^3J_{FF}$= 21.6 Hz), -135.6 (d, $^3J_{FF}$ = 21.6 Hz); HRMS (m/z): $[M]^+$ calcd for $C_{24}H_{21}ON_2F_2$ 391.1616; found 391.1604.

*Chiral HPLC spectrum of racemic **3l***

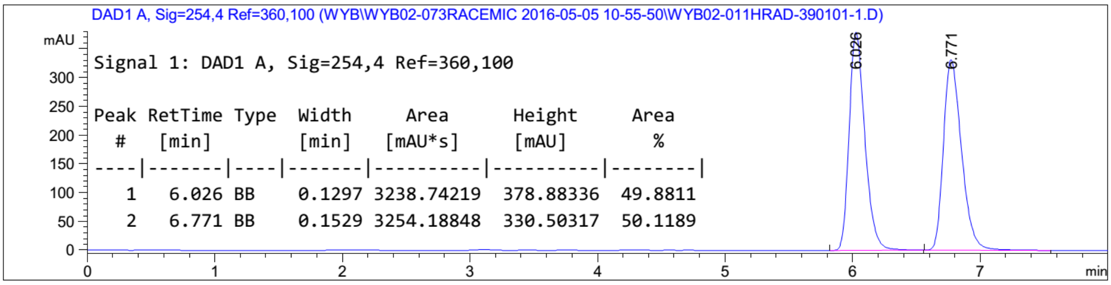


*Chiral HPLC spectrum of **3l***

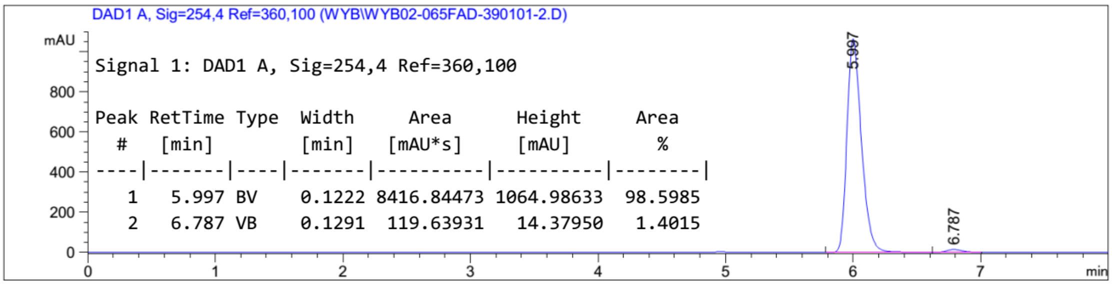


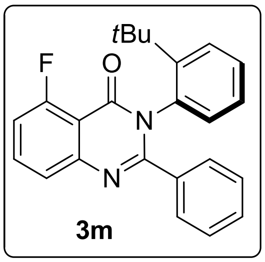


**3m**

**(*aR*)-3-(2-(*tert*-Butyl)phenyl)-5-fluoro-2-phenylquinazolin-4(3*H*)-one (3m)**

According to Procedure D at r.t, **3m** was obtained in 97% yield and 92% ee as a white solid. The ee was determined by chiral stationary phase HPLC analysis [Daicel CHIRALCEL OD-3, hexane/isopropanol = 90/10, 1.0 mL/min, T = 25 °C, λ = 254 nm, $t_R$ (major) = 11.2 min, $t_R$ (minor) = 15.0 min]. $^1$H NMR (400 MHz, $CDCl_3$) δ 7.72 (td, J = 8.1, 5.4 Hz, 1H), 7.62 (d, J = 8.2 Hz, 1H), 7.45 (d, J = 7.4 Hz, 1H), 7.38 (d, J = 7.3 Hz, 2H), 7.33-7.25 (m, 2H), 7.22-7.14 (m, 4H), 7.06 (dd, J = 7.8, 0.8 Hz, 1H), 1.13 (s, 9H); $^{13}$C NMR (101 MHz, $CDCl_3$) δ 161.6 (d, $^1J_{CF}$ = 266.8 Hz), 160.5 (d, $^3J_{CF}$ = 4.4 Hz), 156.5, 149.9, 146.7, 135.095, 135.090 (d, $^3J_{CF}$ = 10.4 Hz), 134.1, 132.3, 130.8, 130.0, 129.8, 129.3, 127.9, 126.6, 123.8 (d, $^4J_{CF}$ = 4.3 Hz), 113.8 (d, $^2J_{CF}$ = 20.9 Hz), 110.7 (d, $^2J_{CF}$ = 6.2 Hz), 36.6, 31.9; $^{19}$F NMR (376 MHz, $CDCl_3$) δ -109.7; HRMS (m/z): $[M]^+$ calcd for $C_{24}H_{22}ON_2F$ 373.1711; found 373.1700.

*Chiral HPLC spectrum of racemic **3m***

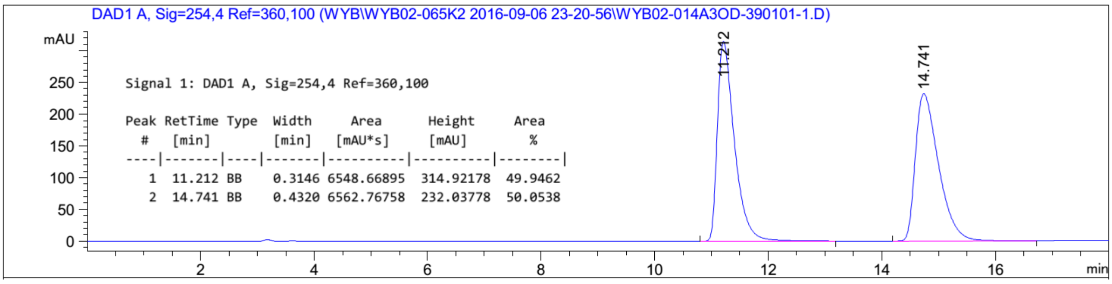


*Chiral HPLC spectrum of **3m***

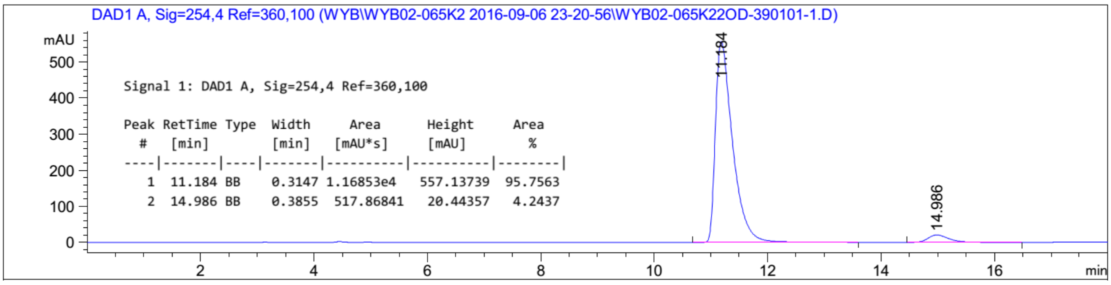


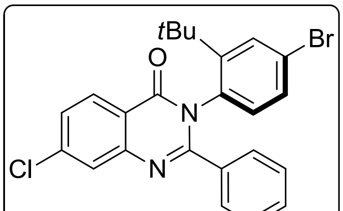


**3n**

**(*aR*)-3-(4-Bromo-2-(*tert*-butyl)phenyl)-7-chloro-2-phenylquinazolin-4(*3H*)-one (3n)**

According to Procedure D, **3n** was obtained in 93% yield and 97% ee as a colorless oil. The ee was determined by chiral stationary phase HPLC analysis [Daicel CHIRALCEL OD-3, hexane/isopropanol = 90/10, 1.0 mL/min, T = 25 °C, $\lambda$ = 254 nm, $t_R$ (minor) = 8.0 min, $t_R$ (major) = 9.5 min]. $^1$H NMR (400 MHz, $CDCl_3$) $\delta$ 8.27 (d, J = 8.5 Hz, 1H), 7.82 (d, J = 1.9 Hz, 1H), 7.58 (d, J = 2.2 Hz, 1H), 7.49 (dd, J = 8.5, 2.0 Hz, 1H), 7.38-7.36 (m, 2H), 7.35-7.31 (m, 2H), 7.28-7.24 (m, 2H), 6.92 (d, J = 8.4 Hz, 1H), 1.11 (s, 9H); $^{13}$C NMR (101 MHz, $CDCl_3$) $\delta$ 162.8, 156.4, 148.9, 148.7, 141.2, 134.8, 134.0, 133.7, 133.5, 130.1, 123.0, 129.9, 128.9, 128.2, 128.1, 127.5, 123.6, 119.1, 36.9, 31.7; HRMS (m/z): $[M]^+$ calcd for $C_{24}H_{21}ON_2BrCl$ 467.0520; found 467.0504.

*Chiral HPLC spectrum of racemic **3n***

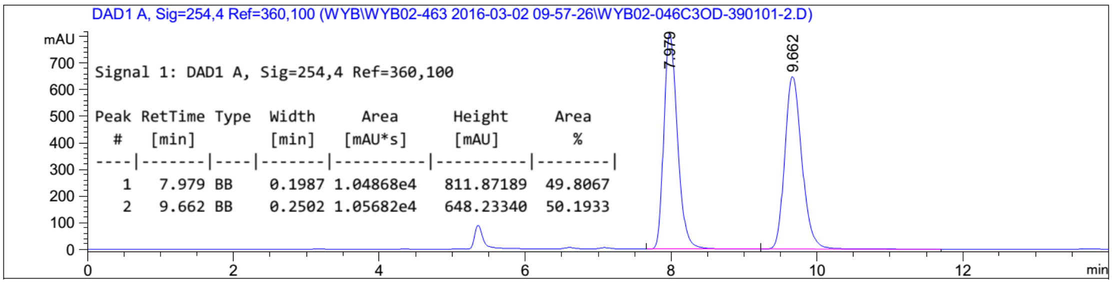


*Chiral HPLC spectrum of **3n***

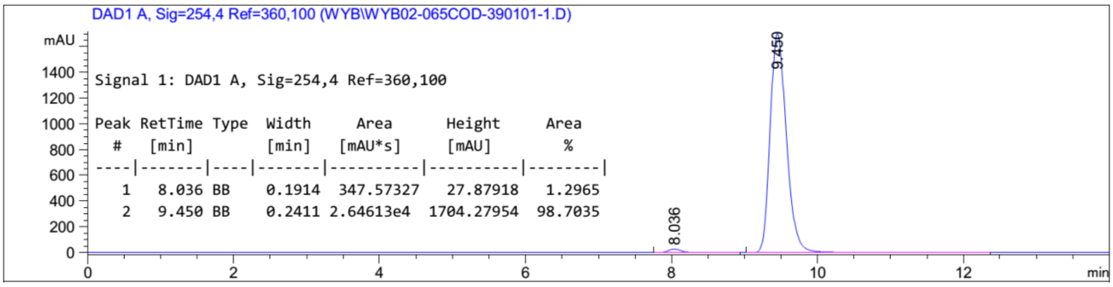

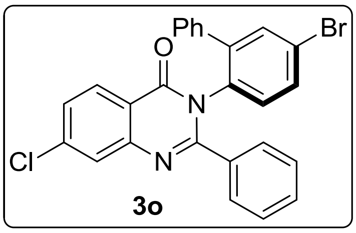


**(*aR*)-3-(5-Bromo-[1,1'-biphenyl]-2-yl)-7-chloro-2-phenylquinazolin-4(*3H*)-one (3o)**

According to Procedure D, **3o** was obtained in 97% yield and 93% ee as a white solid. The ee was determined by chiral stationary phase HPLC analysis [Daicel CHIRALCEL OD-3, hexane/isopropanol = 90/10, 1.0 mL/min, T = 25 °C, λ = 254 nm, $t_R$ (minor) = 20.2 min, $t_R$ (major) = 31.2 min]. $^1$H NMR (400 MHz, $CDCl_3$) δ 8.32 (d, J = 8.5 Hz, 1H), 7.68 (d, J = 1.9 Hz, 1H), 7.61 (dd, J = 8.5, 2.3 Hz, 1H), 7.48 (dd, J = 8.5, 2.0 Hz, 1H), 7.43 (d, J = 8.4 Hz, 1H), 7.38 (d, J = 2.2 Hz, 1H), 7.29-7.21 (m, 2H), 7.15-7.09 (m, 4H), 6.82-6.80 (m, 2H), 6.71-6.69 (m, 2H); $^{13}$C NMR (101 MHz, $CDCl_3$) δ 162.8, 155.8, 148.6, 141.8, 141.2, 136.3, 134.3, 134.2, 134.1, 132.5, 131.1, 129.9, 129.3, 128.9, 128.54, 128.51, 128.4, 128.0, 127.6, 127.5, 123.4, 118.9; HRMS (m/z): $[M]^+$ calcd for $C_{26}H_{25}ON_2$ 487.0207; found 487.0192.

*Chiral HPLC spectrum of racemic **3o***

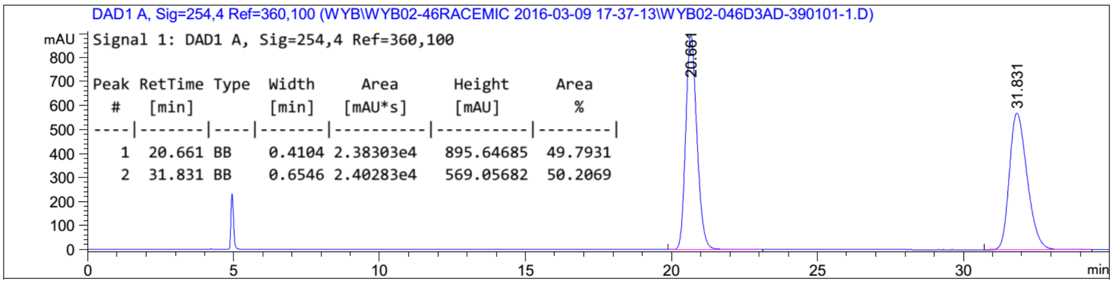


*Chiral HPLC spectrum of **3o***

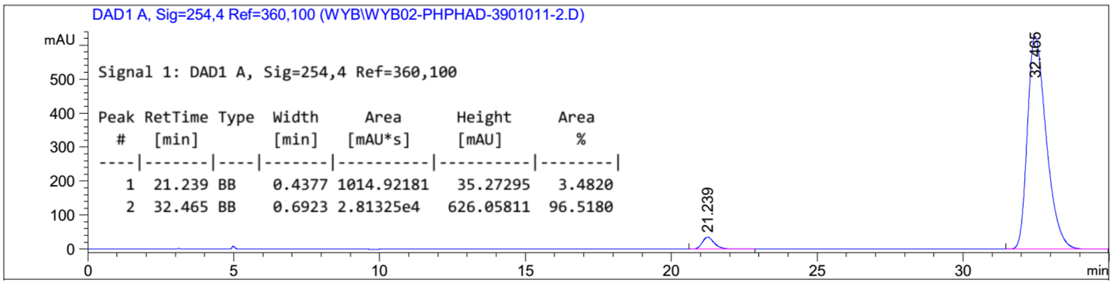


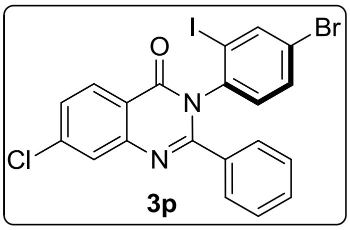


**(*aR*)-3-(4-Bromo-2-iodophenyl)-7-chloro-2-phenylquinazolin-4(*3H*)-one (3p)**

According to Procedure D, **3p** was obtained in 99% yield and 85% ee as a light brown solid. The ee was determined by chiral stationary phase HPLC analysis [Daicel CHIRALCEL OD-3, hexane/isopropanol = 90/10, 1.0 mL/min, T = 25 °C, λ = 254 nm, $t_R$ (major) = 20.1 min, $t_R$ (minor) = 39.2 min]. $^1$H NMR (400 MHz, $CDCl_3$) δ 8.27 (d, J = 8.5 Hz, 1H), 7.94 (d, J = 2.1 Hz, 1H), 7.83 (d, J =

1.9 Hz, 1H), 7.51-7.42 (m, 4H), 7.36-7.31 (m, 1H), 7.29-7.25 (m, 2H), 7.11 (d, J = 8.4 Hz, 1H); $^{13}$C NMR (101 MHz, $CDCl_3$) δ 160.9, 155.7, 148.5, 142.1, 141.5, 139.8, 134.3, 132.4, 131.8, 130.2, 129.0, 128.9, 128.2, 127.7, 123.5, 119.4, 100.6; HRMS (m/z): $[M]^+$ calcd for $C_{20}H_{12}ON_2BrClI$ 536.8861; found 536.8843.

*Chiral HPLC spectrum of racemic **3p***

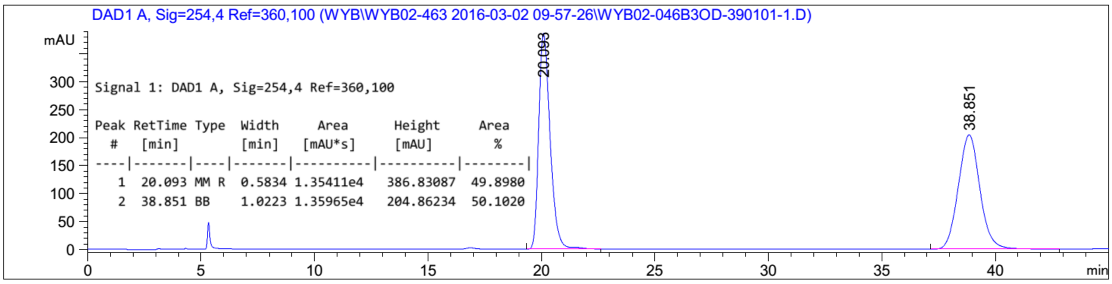


*Chiral HPLC spectrum of **3p***

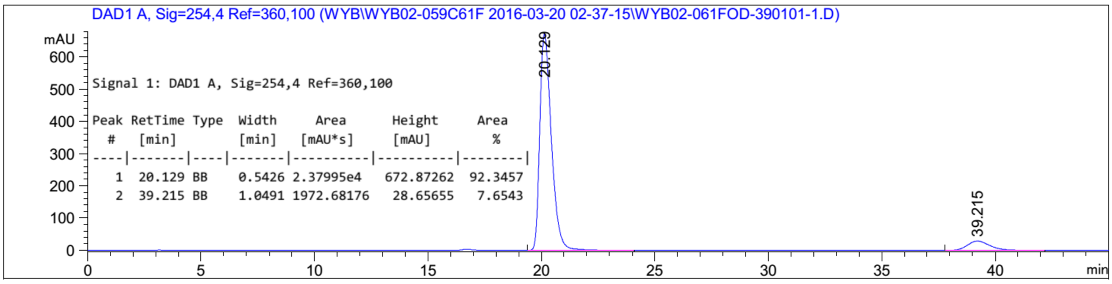


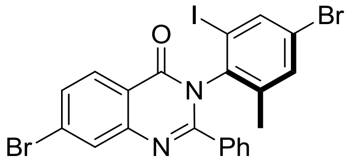


**3q**

**(*aR*)-7-Bromo-3-(4-bromo-2-iodo-6-methylphenyl)-2-phenylquinazolin-4(*3H*)-one (3q)**

According to Procedure D, **3q** was obtained in 96% yield and 94% ee as a white solid. The ee was determined by chiral stationary phase HPLC analysis [Daicel CHIRALCEL AD-3, hexane/isopropanol = 90/10, 1.0 mL/min, T = 25 °C, λ = 254 nm, $t_R$ (minor) = 13.3 min, $t_R$ (major) = 17.5 min]. $^1$H NMR (400 MHz, $CDCl_3$) δ 8.29 (d, J = 8.5 Hz, 1H), 7.85 (d, J = 1.9 Hz, 1H), 7.80 (d, J = 1.9 Hz, 1H), 7.51 (dd, J = 8.5, 2.0 Hz, 1H), 7.47-7.45 (m, 2H), 7.37-7.34 (m, 2H), 7.29-7.25 (m, 2H), 2.18 (s, 3H); $^{13}$C NMR (101 MHz, $CDCl_3$) δ 160.4, 155.6, 148.7, 141.5, 139.8, 139.5, 138.8, 134.1, 134.1, 130.5, 129.0, 128.7, 128.2, 128.1, 127.7, 123.6, 119.3, 101.3, 19.7; HRMS (m/z): $[M]^+$ calcd for $C_{21}H_{14}ON_2BrClI$ 550.9017; found 550.9001.

*Chiral HPLC spectrum of racemic **3q***

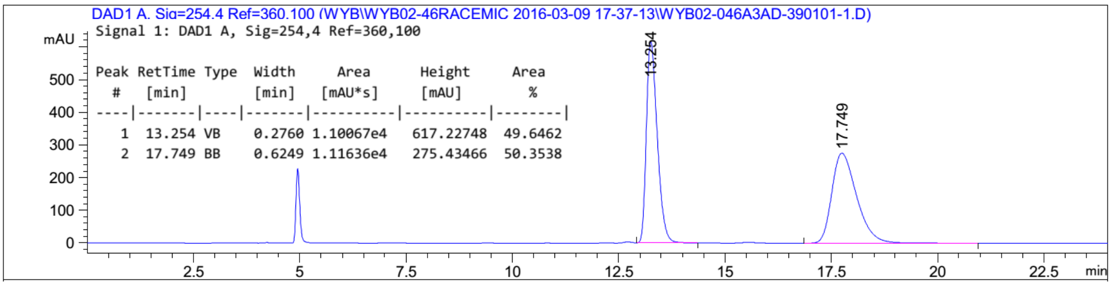

*Chiral HPLC spectrum of **3q***

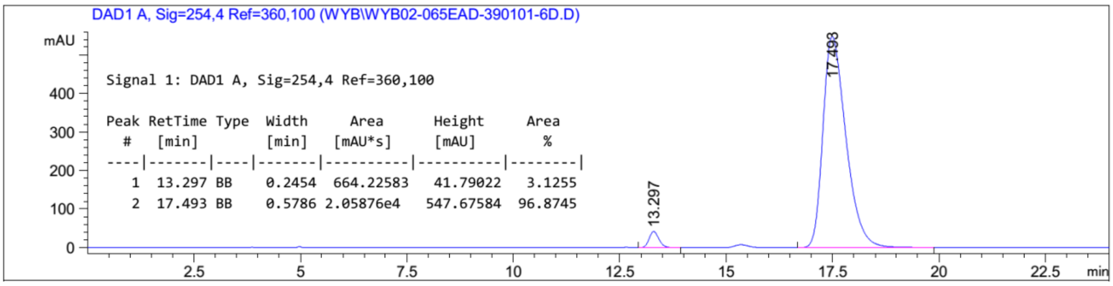


| Peak # | RetTime [min] | Type | Width [min] | Area [mAU*s] | Height [mAU] | Area % |
|---|---|---|---|---|---|---|
| 1 | 13.297 | BB | 0.2454 | 664.22583 | 41.79022 | 3.1255 |
| 2 | 17.493 | BB | 0.5786 | 2.05876e4 | 547.67584 | 96.8745 |

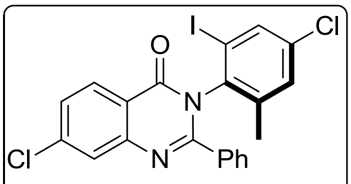

**3r**

**(*aR*)-7-Chloro-3-(4-chloro-2-iodo-6-methylphenyl)-2-phenylquinazolin-4(*3H*)-one (3r):**
According to Procedure D, **3r** was obtained in 98% yield and 93% ee as a white solid. The ee was determined by chiral stationary phase HPLC analysis [Daicel CHIRALCEL AD-3, hexane/isopropanol = 90/10, 1.0 mL/min, T = 25 °C, λ = 254 nm, $t_R$ (minor) = 11.7 min, $t_R$ (major) = 16.6 min]. $^1$H NMR (400 MHz, $CDCl_3$) δ 8.29 (d, J = 8.5 Hz, 1H), 7.85 (d, J = 2.0 Hz, 1H), 7.65 (d, J = 2.2 Hz, 1H), 7.51 (dd, J = 8.5, 2.0 Hz, 1H), 7.48-7.45 (m, 2H), 7.38-7.33 (m, 1H), 7.29-7.25 (m, 2H), 7.19-7.18 (m, 1H), 2.19 (s, 3H); $^{13}$C NMR (101 MHz, $CDCl_3$) δ 160.4, 155.7, 148.8, 141.5, 139.1, 138.4, 137.1, 135.5, 134.2, 131.1, 130.5, 129.0, 128.8, 128.2, 128.1, 127.7, 119.3, 100.8, 19.8; HRMS (m/z): $[M]^+$ calcd for $C_{21}H_{14}ON_2Cl_2I$ 506.9522; found 506.9505.

*Chiral HPLC spectrum of racemic **3r***

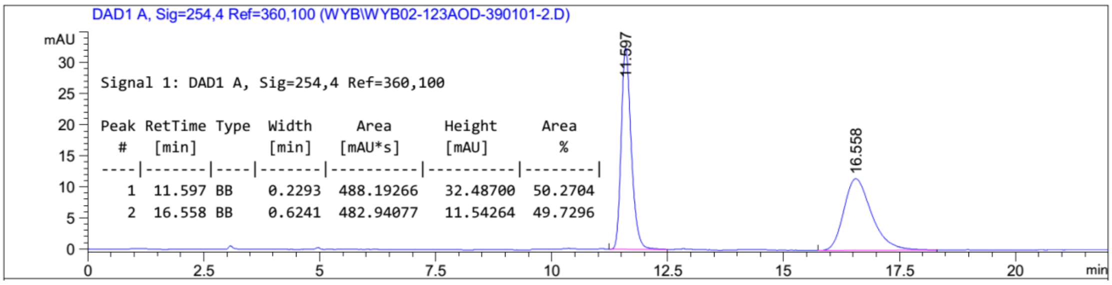


| Peak # | RetTime [min] | Type | Width [min] | Area [mAU*s] | Height [mAU] | Area % |
|---|---|---|---|---|---|---|
| 1 | 11.597 | BB | 0.2293 | 488.19266 | 32.48700 | 50.2704 |
| 2 | 16.558 | BB | 0.6241 | 482.94077 | 11.54264 | 49.7296 |

*Chiral HPLC spectrum of **3r***

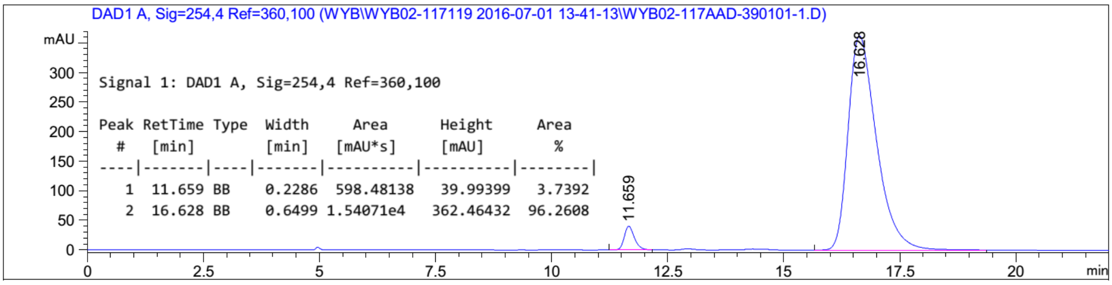


| Peak # | RetTime [min] | Type | Width [min] | Area [mAU*s] | Height [mAU] | Area % |
|---|---|---|---|---|---|---|
| 1 | 11.659 | BB | 0.2286 | 598.48138 | 39.99399 | 3.7392 |
| 2 | 16.628 | BB | 0.6499 | 1.54071e4 | 362.46432 | 96.2608 |

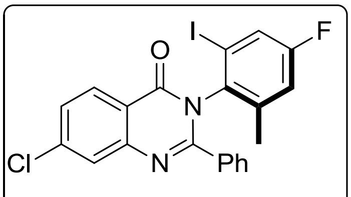


**3s**

**(*aR*)-7-Chloro-3-(4-fluoro-2-iodo-6-methylphenyl)-2-phenylquinazolin-4(*3H*)-one (3s)**

According to Procedure D, **3s** was obtained in 97% yield and 90% ee as a white solid. The ee was determined by chiral stationary phase HPLC analysis [Daicel CHIRALCEL AD-3, hexane/isopropanol = 90/10, 1.0 mL/min, T = 25 °C, λ = 254 nm, $t_R$ (minor) = 9.2 min, $t_R$ (major) = 14.7 min]. $^1$H NMR (400 MHz, $CDCl_3$) δ 8.30 (d, J = 8.5 Hz, 1H), 7.85 (d, J = 2.0 Hz, 1H), 7.51 (dd, J = 8.5, 2.0 Hz, 1H), 7.48-7.44 (m, 2H), 7.38 (dd, J = 7.4, 2.8 Hz, 1H), 7.36-7.32 (m, 1H), 7.28-7.24 (m, 2H), 6.93 (dd, J = 8.7, 2.5 Hz, 1H), 2.20 (s, 3H); $^{13}$C NMR (101 MHz, $CDCl_3$) δ 161.75 (d, $^1J_{CF}$ = 254.2 Hz), 160.6, 156.0, 148.8, 141.5, 139.57 (d, $^3J_{CF}$ = 8.7 Hz), 135.87 (d, $^4J_{CF}$ = 3.5 Hz), 134.3, 130.4, 129.0, 128.8, 128.2, 128.0, 127.7, 124.76 (d, $^2J_{CF}$ = 24.8 Hz), 119.4, 118.0 (d, $^2J_{CF}$ = 22.1 Hz), 100.4 (d, $^3J_{CF}$ = 9.1 Hz), 20.0; $^{19}$F NMR (376 MHz, $CDCl_3$) δ -110.7; HRMS (m/z): $[M]^+$ calcd for $C_{21}H_{14}ON_2ClFI$ 490.9818; found 490.9803.

*Chiral HPLC spectrum of racemic **3s***

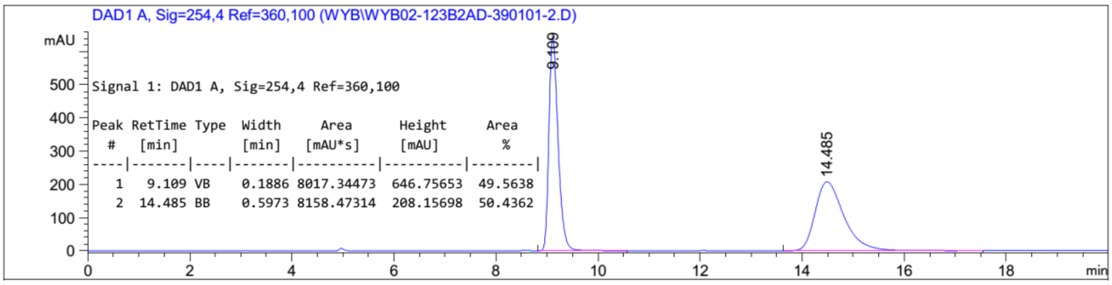


*Chiral HPLC spectrum of **3s***

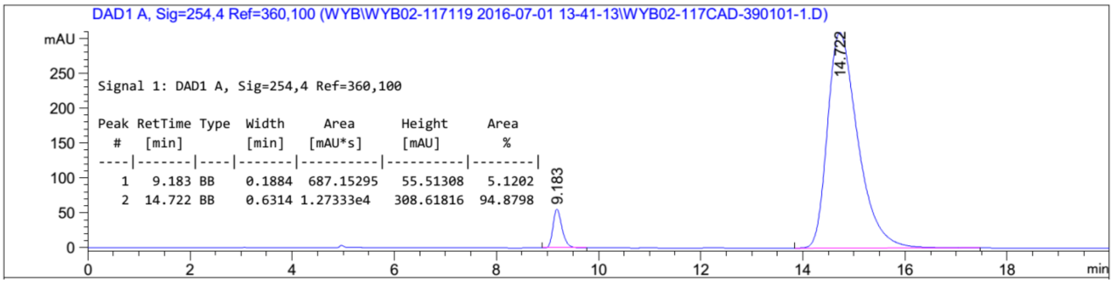


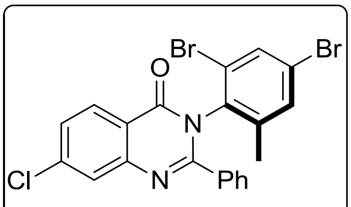


**3t**

**(*aR*)-7-Chloro-3-(2,4-dibromo-6-methylphenyl)-2-phenylquinazolin-4(*3H*)-one (3t)**

According to Procedure D, **3t** was obtained in 97% yield and 83% ee as a colorless oil. The ee was determined by chiral stationary phase HPLC analysis [Daicel CHIRALCEL AD-3, hexane/isopropanol

= 90/10, 1.0 mL/min, T = 25 °C, λ = 254 nm, $t_R$ (minor) = 12.7 min, $t_R$ (major) = 15.5 min]. $^1$H NMR (400 MHz, $CDCl_3$) δ 8.28 (d, J = 8.5 Hz, 1H), 7.84 (d, J = 1.9 Hz, 1H), 7.58 (d, J = 2.0 Hz, 1H), 7.50 (dd, J = 8.5, 2.0 Hz, 1H), 7.47-7.43 (m, 2H), 7.36 (tt, J = 7.4, 2.2 Hz, 1H), 7.31-7.25 (m, 3H), 2.15 (s, 3H); $^{13}$C NMR (101 MHz, $CDCl_3$) δ 160.4, 155.9, 148.8, 141.5, 140.0, 135.6, 134.2, 133.6, 133.2, 130.5, 128.9, 128.3, 128.2, 127.7, 124.6, 123.5, 119.1, 19.0; HRMS (m/z): $[M]^+$ calcd for $C_{21}H_{14}ON_2Br_2Cl$ 502.9156; found 502.9139.

*Chiral HPLC spectrum of racemic **3t***

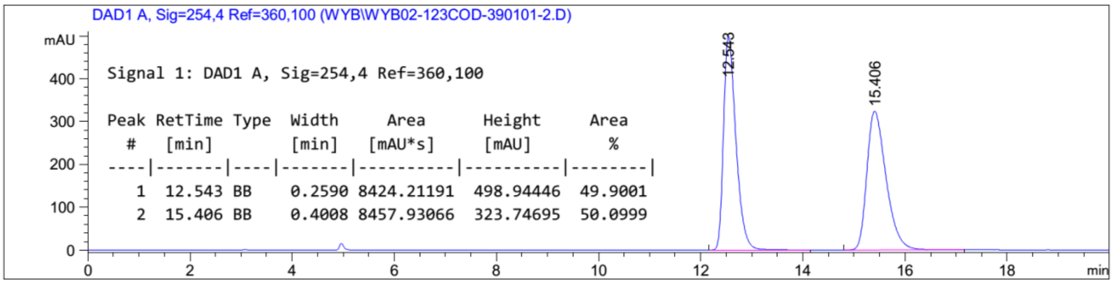


*Chiral HPLC spectrum of **3t***

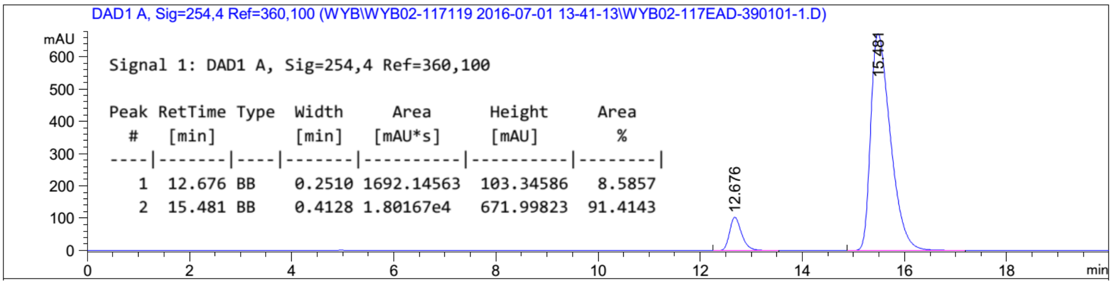


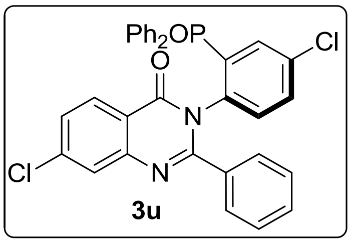


**3u**

**(*aR*)-7-Chloro-3-(4-chloro-2-(diphenylphosphoryl)phenyl)-2-phenylquinazolin-4(*3H*)-one (3u)**

According to Procedure D, **3u** was obtained in 95% yield and 89% ee as a white solid. The product was recrystallized from PE/DCM to afford **3u** in 51% yield and >99% ee as a white solid. The ee was determined by chiral stationary phase HPLC analysis [Daicel CHIRALPAK IA, hexane/isopropanol = 80/20, 1.0 mL/min, T = 25 °C, λ = 254 nm, $t_R$ (major) = 25.1 min, $t_R$ (minor) = 47.2 min]. $^1$H NMR (400 MHz, $CDCl_3$) δ 8.00-7.97 (m, 1H), 7.76 (d, J = 1.9 Hz, 1H), 7.57-7.52 (m, 1H), 7.50-7.40 (m, 9H), 7.37 (dd, J = 8.6, 1.9 Hz, 2H), 7.32-7.22 (m, 4H), 7.20-7.13 (m, 3H); $^{13}$C NMR (101 MHz, $CDCl_3$) δ 162.2, 156.3, 148.6, 140.7, 140.2 (d, $^2J_{CP}$ = 2.0 Hz), 135.0, 134.7 (d, $^3J_{CP}$ = 14.8 Hz), 134.2 (d, $^2J_{CP}$ = 11.2 Hz), 134.0 (d, $^2J_{CP}$ = 8.0 Hz), 132.65 (d, $^1J_{CP}$ = 95.0 Hz), 132.54 (d, $^4J_{CP}$ = 2.1 Hz), 132.40 (d, $^4J_{CP}$ = 2.7 Hz), 132.13, 132.085, 132.03, 131.9 (d, J = 10.2 Hz), 131.5 (d, J = 107.2 Hz), 131.1, 129.9, 129.5, 128.8 (d, J = 12.4 Hz), 128.5, 128.4 (d, J = 12.6 Hz), 128.0, 127.4 (d, $^3J_{CP}$ = 6.7 Hz), 119.3; $^{31}$P NMR (162 MHz, $CDCl_3$) δ 27.3; HRMS (m/z): $[M]^+$ calcd for $C_{33}H_{21}O_2N_2Cl_2P$ 567.0790; found 567.0774.

*Chiral HPLC spectrum of racemic **3u***

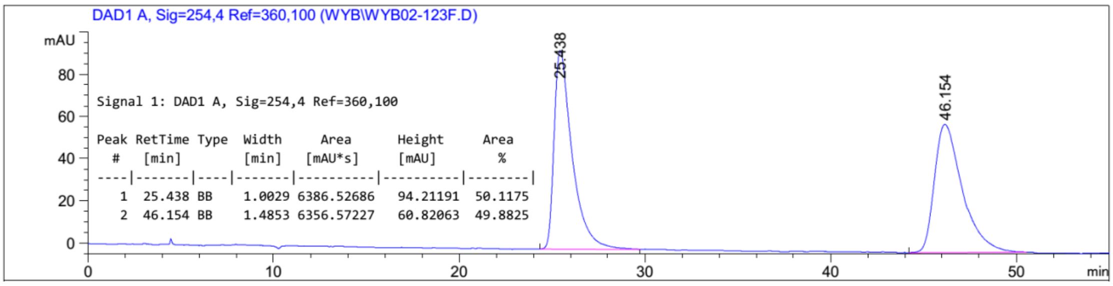


Chiral HPLC spectrum of ***3u***

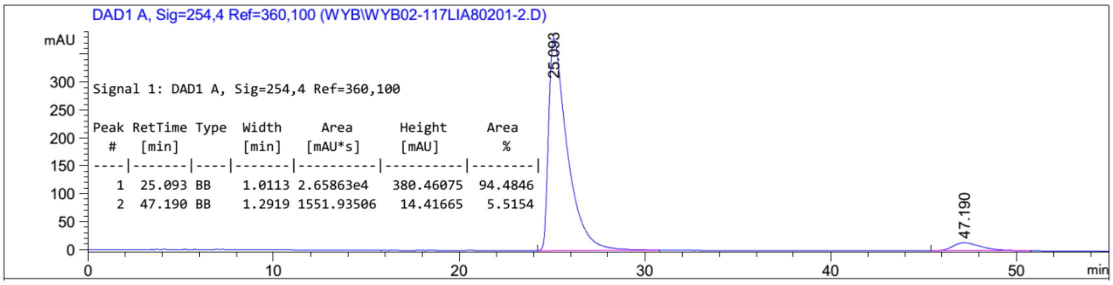


Chiral HPLC spectrum of ***3u***

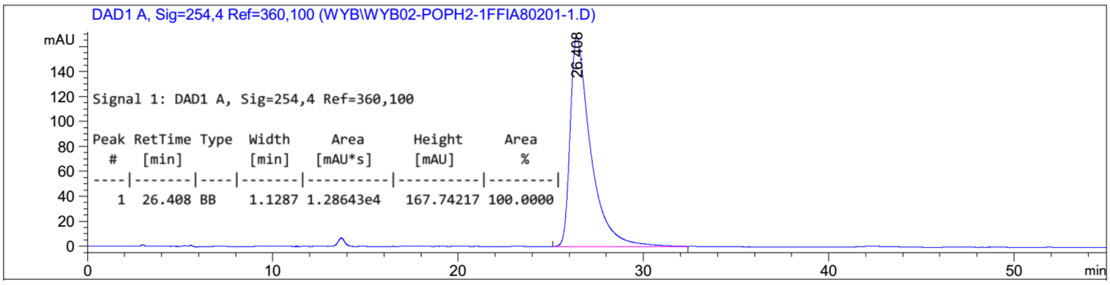


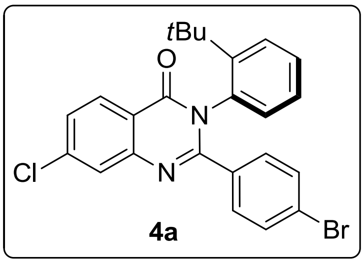


**(*aR*)-2-(4-Bromophenyl)-3-(2-(*tert*-butyl)phenyl)-7-chloroquinazolin-4(*3H*)-one (4a)**

According to Procedure D, **4a** was obtained in 96% yield and 92% ee as a colorless oil. The ee was determined by chiral stationary phase HPLC analysis [Daicel CHIRALCEL OD-3, hexane/isopropanol = 90/10, 1.0 mL/min, T = 25 °C, λ = 254 nm, $t_R$ (major) = 11.4 min, $t_R$ (minor) = 18.5 min]. $^1$H NMR (400 MHz, $CDCl_3$) δ 8.27 (d, J = 8.5 Hz, 1H), 7.81 (d, J = 1.9 Hz, 1H), 7.51-7.47 (m, 2H), 7.38-7.33 (m, 3H), 7.28-7.21 (m, 3H), 7.03 (dd, J = 7.9, 1.4 Hz, 1H), 1.11 (s, 9H); $^{13}$C NMR (101 MHz, $CDCl_3$) δ 162.8, 155.7, 148.7, 146.6, 141.2, 134.11, 134.05, 132.0, 131.6, 131.2, 131.0, 129.7, 128.9, 128.1, 127.5, 126.9, 124.7, 119.4, 36.6, 31.9; HRMS (m/z): $[M]^+$ calcd for $C_{24}H_{21}ON_2BrCl$ 467.0520; found 467.0504.

*Chiral HPLC spectrum of racemic* ***4a***

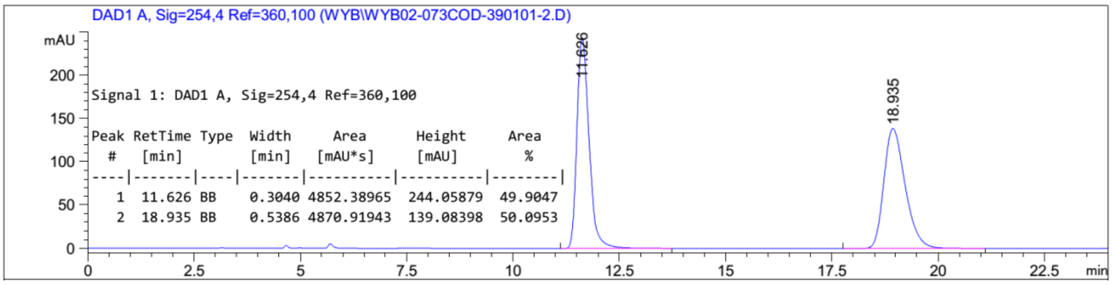


*Chiral HPLC spectrum of **4a***

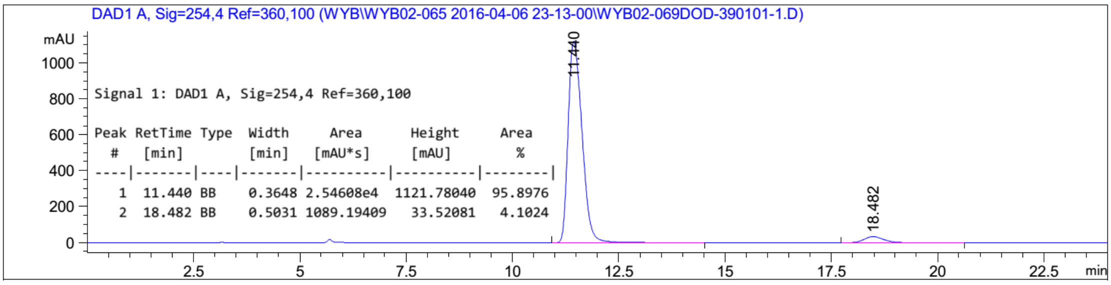


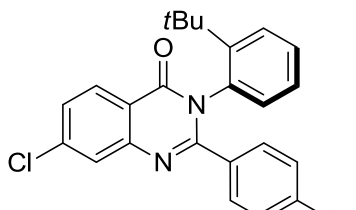

**4b**

**(*aR*)-3-(2-(*tert*-Butyl)phenyl)-7-chloro-2-(4-chlorophenyl)quinazolin-4(*3H*)-one (4b)**

According to Procedure D, **4b** was obtained in 94% yield and 93% ee as a colorless oil. The ee was determined by chiral stationary phase HPLC analysis [Daicel CHIRALCEL OD-3, hexane/isopropanol = 90/10, 1.0 mL/min, T = 25 °C, λ = 254 nm, $t_R$ (major) = 11.3 min, $t_R$ (minor) = 18.9 min]. $^1$H NMR (400 MHz, $CDCl_3$) δ 8.27 (d, J = 8.5 Hz, 1H), 7.81 (d, J = 1.9 Hz, 1H), 7.51-7.47 (m, 2H), 7.38-7.31 (m, 3H), 7.25-7.17 (m, 3H), 7.04 (dd, J = 7.8, 1.4 Hz, 1H), 1.11 (s, 10H); $^{13}$C NMR (101 MHz, $CDCl_3$) δ 162.8, 155.6, 148.6, 146.6, 141.1, 136.3, 134.1, 133.6, 132.0, 131.4, 131.0, 129.6, 128.9, 128.2, 128.1, 127.5, 126.9, 119.3, 36.6, 31.9; HRMS (m/z): $[M]^+$ calcd for $C_{24}H_{21}ON_2Cl_2$ 423.1025; found 423.1011.

*Chiral HPLC spectrum of racemic **4b***

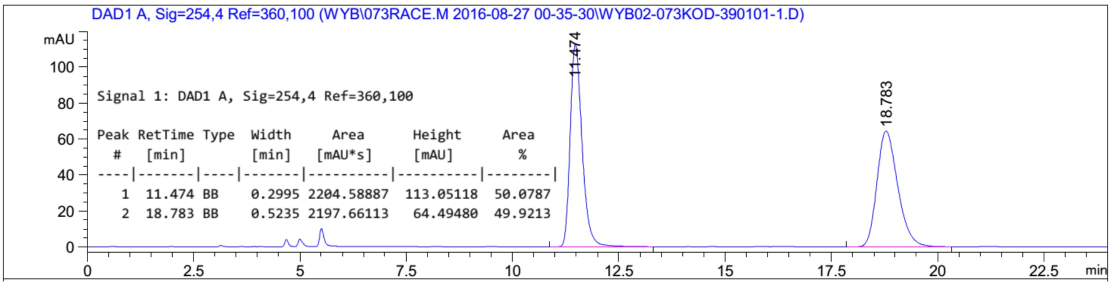


*Chiral HPLC spectrum of **4b***

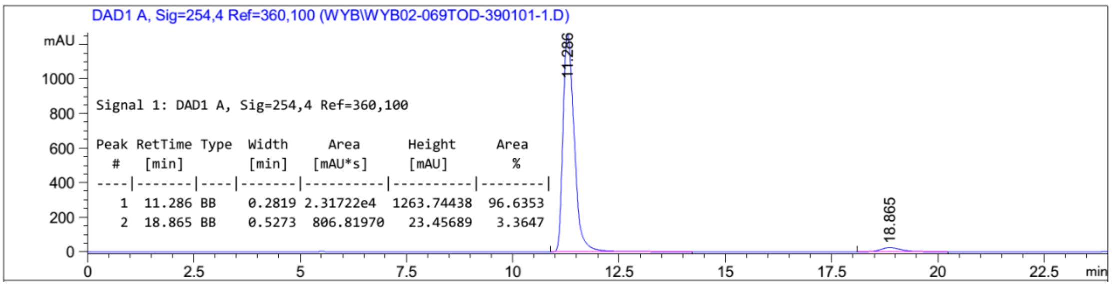


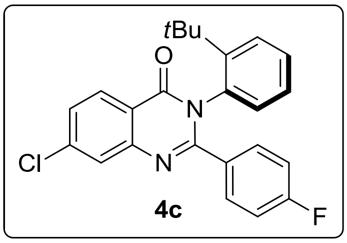


**(a*R*)-3-(2-(*tert*-Butyl)phenyl)-7-chloro-2-(4-fluorophenyl)quinazolin-4(3*H*)-one (4c)**

According to Procedure D, **4c** was obtained in 97% yield and 95% ee as a colorless oil. The ee was determined by chiral stationary phase HPLC analysis [Daicel CHIRALCEL OD-3, hexane/isopropanol = 90/10, 1.0 mL/min, T = 25 °C, λ = 254 nm, $t_R$ (major) = 11.8 min, $t_R$ (minor) = 17.9 min]. $^1$H NMR (400 MHz, $CDCl_3$) δ 8.28 (d, J = 8.5 Hz, 1H), 7.81 (d, J = 1.9 Hz, 1H), 7.50-7.47 (m, 2H), 7.41-7.33 (m, 3H), 7.23 (td, J = 7.6, 1.4 Hz, 1H), 7.05 (dd, J = 7.8, 1.4 Hz, 1H), 6.93-6.87 (m, 2H), 1.10 (s, 9H); $^{13}$C NMR (101 MHz, $CDCl_3$) δ 163.4 (d, $^1J_{CF}$ = 252.1 Hz), 162.9, 155.7, 148.7, 146.5, 141.1, 134.2, 132.30 (d, $^3J_{CF}$ = 8.6 Hz), 132.0, 131.28 (d, $^4J_{CF}$ = 3.4 Hz), 130.9, 129.6, 128.9, 128.0, 127.4, 126.8, 119.3, 115.1 (d, $^2J_{CF}$ = 21.8 Hz), 36.6, 31.9; $^{19}$F NMR (376 MHz, $CDCl_3$) δ -109.7; HRMS (m/z): $[M]^+$ calcd for $C_{24}H_{21}ON_2ClF$ 407.1321; found 407.1307.

*Chiral HPLC spectrum of racemic **4c***

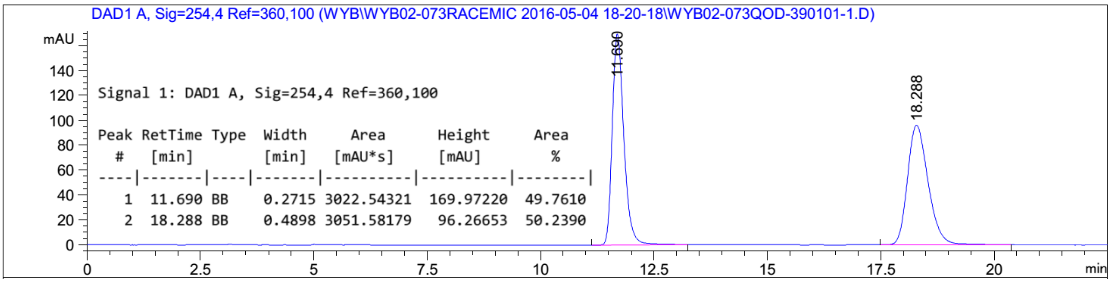


*Chiral HPLC spectrum of **4c***

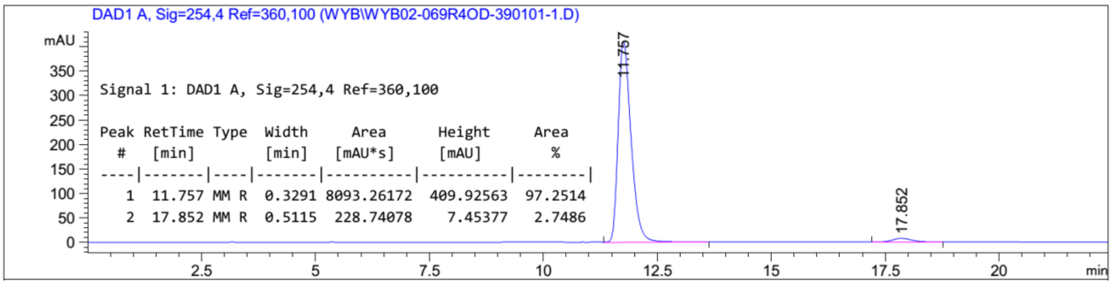

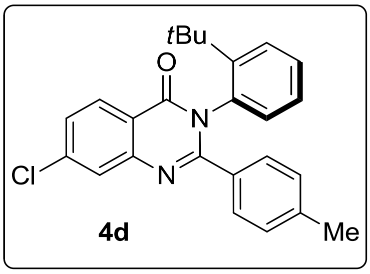


**(*aR*)-3-(2-(*tert*-Butyl)phenyl)-7-chloro-2-(p-tolyl)quinazolin-4(*3H*)-one (4d)**

According to Procedure D, **4d** was obtained in 98% yield and 93% ee as a colorless oil. The ee was determined by chiral stationary phase HPLC analysis [Daicel CHIRALCEL OD-3, hexane/isopropanol = 90/10, 1.0 mL/min, T = 25 °C, λ = 254 nm, $t_R$ (major) = 10.9 min, $t_R$ (minor) = 16.7 min]. $^1$H NMR (400 MHz, $CDCl_3$) δ 8.27 (d, J = 8.5 Hz, 1H), 7.81 (d, J = 1.9 Hz, 1H), 7.49-7.45 (m, 2H), 7.35-7.31 (m, 1H), 7.27-7.25 (m, 1H), 7.21 (td, J = 7.6, 1.4 Hz, 1H), 7.04 (dd, J = 7.9, 1.4 Hz, 1H), 7.01 (d, J = 8.1 Hz, 2H), 2.27 (s, 3H), 1.11 (s, 9H); $^{13}$C NMR (101 MHz, $CDCl_3$) δ 163.0, 156.8, 148.9, 146.5, 140.9, 140.2, 134.4, 132.3, 132.1, 130.8, 130.0, 129.3, 128.9, 128.6, 127.7, 127.4, 126.6, 119.3, 36.6, 31.8, 21.4; HRMS (m/z): $[M]^+$ calcd for $C_{25}H_{24}ON_2ClF$ 403.1572; found 403.1559.

*Chiral HPLC spectrum of racemic **4d***

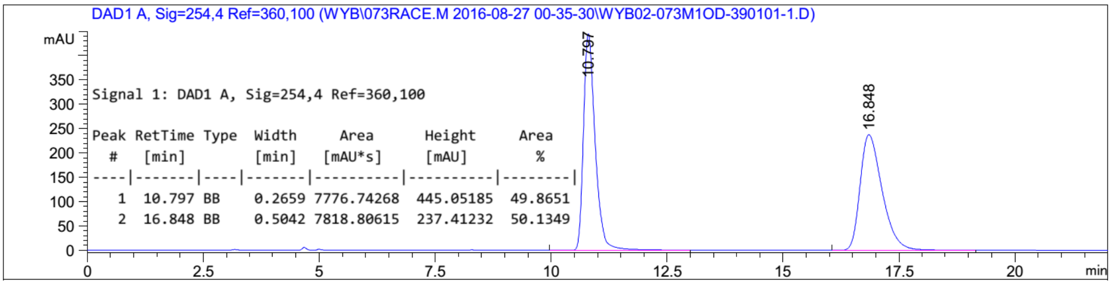


*Chiral HPLC spectrum of **4d***

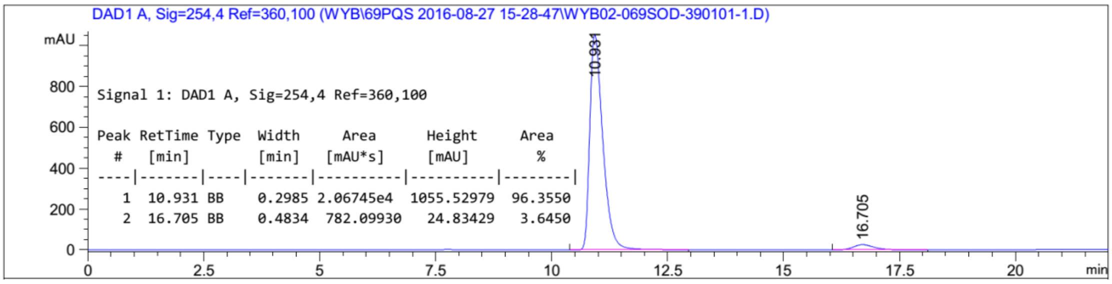


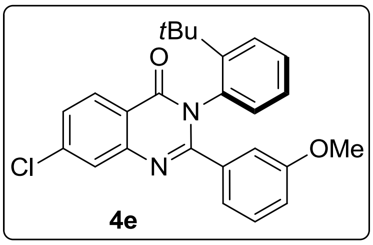


**(*aR*)-3-(2-(*tert*-Butyl)phenyl)-7-chloro-2-(3-methoxyphenyl)quinazolin-4(*3H*)-one (4e)**

According to Procedure D, **4e** was obtained in 95% yield and 94% ee as a colorless oil. The ee was determined by chiral stationary phase HPLC analysis [Daicel CHIRALCEL OD-3, hexane/isopropanol = 90/10, 1.0 mL/min, T = 25 °C, λ = 254 nm, $t_R$ (major) = 16.7 min, $t_R$ (minor) = 25.4 min]. $^1$H NMR (400 MHz, $CDCl_3$) δ 8.28 (d, J = 8.5 Hz, 1H), 7.83 (d, J = 1.9 Hz, 1H), 7.49-7.46 (m, 2H), 7.35-7.31

(m, 1H), 7.22 (td, J = 7.6, 1.4 Hz, 1H), 7.13 (t, J = 8.0 Hz, 1H), 7.06 (dd, J = 7.9, 1.4 Hz, 1H), 7.01 (dt, J = 7.7, 1.2 Hz, 1H), 6.88-6.85 (m, 1H), 6.82 (ddd, J = 8.3, 2.5, 0.7 Hz, 1H), 3.63 (s, 3H), 1.12 (s, 9H); $^{13}$C NMR (101 MHz, $CDCl_3$) δ 162.9, 159.0, 156.5, 148.8, 146.6, 141.0, 136.2, 134.3, 132.1, 130.9, 129.4, 129.0, 128.9, 127.9, 127.5, 126.6, 122.7, 119.4, 116.3, 115.2, 55.5, 36.7, 31.9; HRMS (m/z): $[M]^+$ calcd for $C_{25}H_{24}O_2N_2Cl$ 419.1521; found 419.1507.

*Chiral HPLC spectrum of racemic **4e***

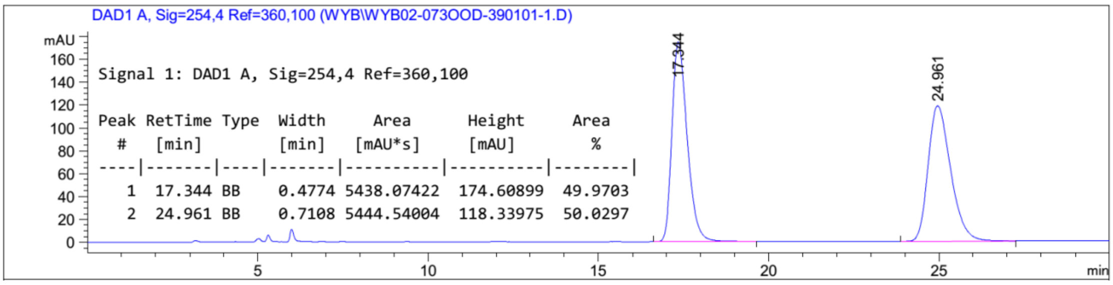


*Chiral HPLC spectrum of **4e***

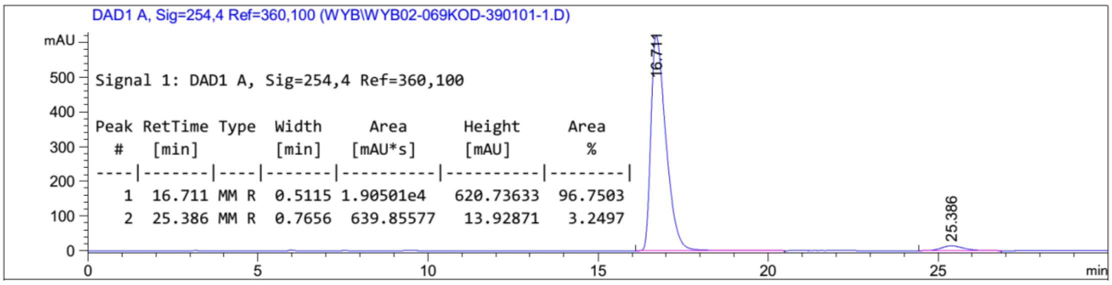


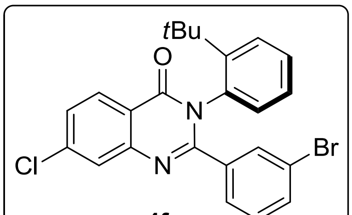


**4f**

**(a*R*)-2-(3-Bromophenyl)-3-(2-(*tert*-butyl)phenyl)-7-chloroquinazolin-4(3*H*)-one (4f)**

According to Procedure D, **4f** was obtained in 63% yield and 92% ee as a colorless oil. The ee was determined by chiral stationary phase HPLC analysis [Daicel CHIRALCEL OD-3, hexane/isopropanol = 90/10, 1.0 mL/min, T = 25 °C, λ = 254 nm, $t_R$ (major) = 15.9 min, $t_R$ (minor) = 21.0 min]. $^1$H NMR (400 MHz, $CDCl_3$) δ 8.28 (d, J = 8.5 Hz, 1H), 7.82 (d, J = 1.9 Hz, 1H), 7.56 (t, J = 1.8 Hz, 1H), δ 7.51 (dd, J = 8.2, 1.4 Hz, 1H), 7.50 (dd, J = 8.5, 2.0 Hz, 1H), 7.41 (ddd, J = 8.0, 1.8, 0.9 Hz, 1H), 7.37-7.33 (m, 1H), 7.30 (ddd, J = 7.9, 1.5, 1.0 Hz, 1H), 7.21 (td, J = 7.6, 1.4 Hz, 1H), 7.07 (t, J = 8.0 Hz, 1H), 6.99 (dd, J = 7.9, 1.4 Hz, 1H), 1.15 (s, 10H); $^{13}$C NMR (101 MHz, $CDCl_3$) δ 162.7, 155.4, 148.6, 146.7, 141.2, 136.9, 134.0, 133.1, 132.9, 131.9, 130.9, 129.7, 129.4, 128.9, 128.5, 128.3, 127.6, 126.9, 122.0, 119.5, 36.7, 31.9; HRMS (m/z): $[M]^+$ calcd for $C_{24}H_{21}ON_2BrCl$ 467.0520; found 467.0505.

*Chiral HPLC spectrum of racemic **4f***

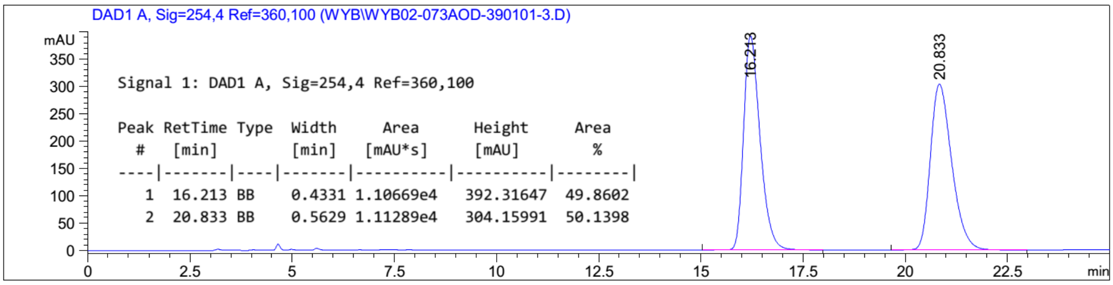


*Chiral HPLC spectrum of **4f***

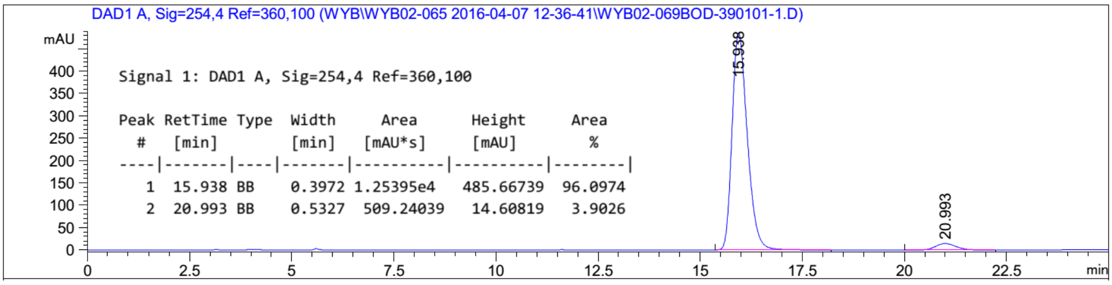


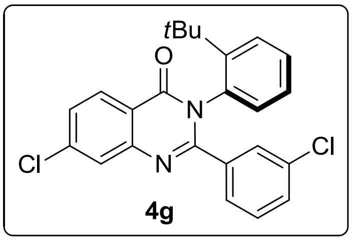


**(*aR*)-3-(2-(*tert*-Butyl)phenyl)-7-chloro-2-(3-chlorophenyl)quinazolin-4(*3H*)-one (4g)**

According to Procedure D, **4g** was obtained in 76% yield and 91% ee as a white solid. The ee was determined by chiral stationary phase HPLC analysis [Daicel CHIRALCEL OD-3, hexane/isopropanol = 90/10, 1.0 mL/min, T = 25 °C, λ = 254 nm, $t_R$ (major) = 15.8 min, $t_R$ (minor) = 20.3 min]. $^1$H NMR (400 MHz, $CDCl_3$) δ 8.29 (d, J = 8.5 Hz, 1H), 7.82 (d, J = 1.9 Hz, 1H), 7.50 (dd, J = 8.6, 1.9 Hz, 2H), 7.41 (t, J = 1.8 Hz, 1H), 7.37-7.33 (m, 1H), 7.27-7.20 (m, 3H), 7.14 (t, J = 7.9 Hz, 1H), 7.00 (dd, J = 7.9, 1.4 Hz, 1H), 1.15 (s, 9H); $^{13}$C NMR (101 MHz, $CDCl_3$) δ 162.7, 155.3, 148.6, 146.6, 141.2, 136.7, 134.03, 133.97, 131.9, 131.0, 130.2, 120.0, 129.7, 129.2, 128.9, 128.3, 128.0, 127.5, 126.9, 119.4, 36.7, 31.9; HRMS (m/z): $[M]^+$ calcd for $C_{24}H_{21}ON_3Cl_2$ 423.1025; found 423.1011.

*Chiral HPLC spectrum of racemic **4g***

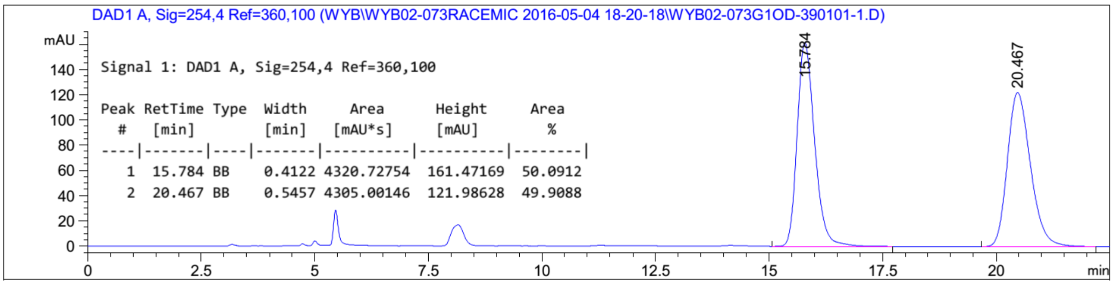


*Chiral HPLC spectrum of **4g***

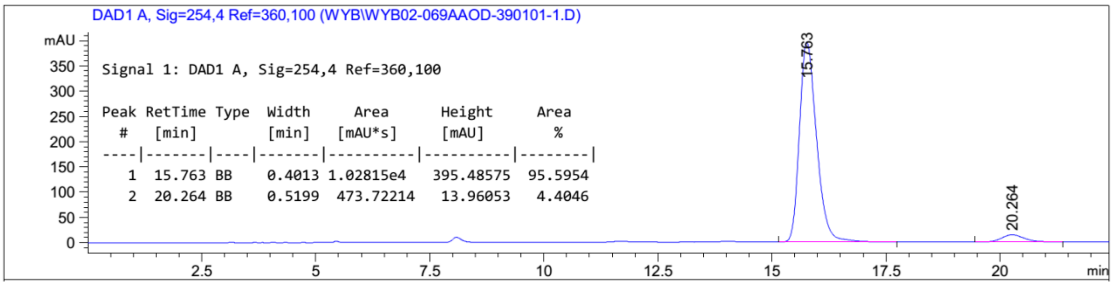


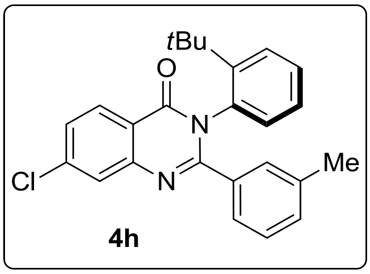


**(*aR*)-3-(2-(*tert*-Butyl)phenyl)-7-chloro-2-(m-tolyl)quinazolin-4(3*H*)-one (4h)**

According to Procedure D, **4h** was obtained in 96% yield and 95% ee as a colorless oil. The ee was determined by chiral stationary phase HPLC analysis [Daicel CHIRALCEL OD-3, hexane/isopropanol = 90/10, 1.0 mL/min, T = 25 °C, λ = 254 nm, $t_R$ (major) = 13.0 min, $t_R$ (minor) = 16.8 min]. $^1$H NMR (400 MHz, $CDCl_3$) δ 8.28 (d, J = 8.5 Hz, 1H), 7.83 (d, J = 1.9 Hz, 1H), 7.49-7.29 (m, 2H), 7.33-7.29 (m, 1H), 7.22 (s, 1H), 7.19 (td, J = 7.6, 1.5 Hz, 1H), 7.14-7.11 (m, 1H), 7.09-7.07 (m, 2H), 7.02 (dd, J = 7.9, 1.4 Hz, 1H), 2.22 (s, 3H), 1.13 (s, 9H); $^{13}$C NMR (101 MHz, $CDCl_3$) δ 163.0, 157.0, 148.8, 146.5, 141.0, 137.7, 134.9, 134.3, 132.1, 130.7, 130.64, 130.56, 129.3, 128.9, 127.8, 127.7, 127.4, 127.0, 126.5, 119.4, 36.7, 31.8, 21.3; HRMS (m/z): $[M]^+$ calcd for $C_{25}H_{24}ON_2Cl$ 403.1572; found 403.1559.

*Chiral HPLC spectrum of racemic **4h***

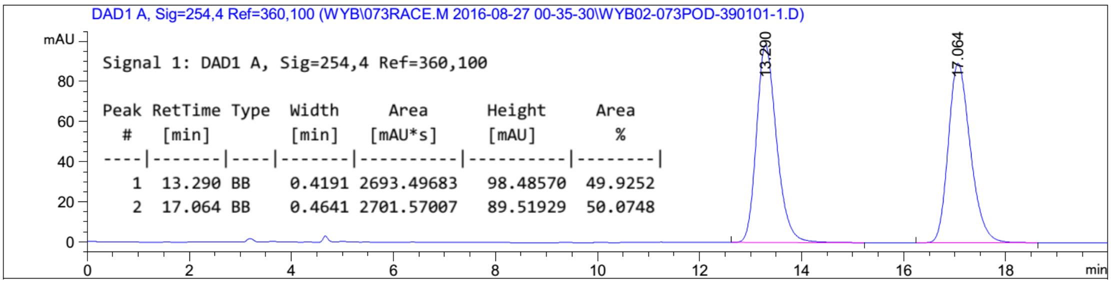


*Chiral HPLC spectrum of **4h***

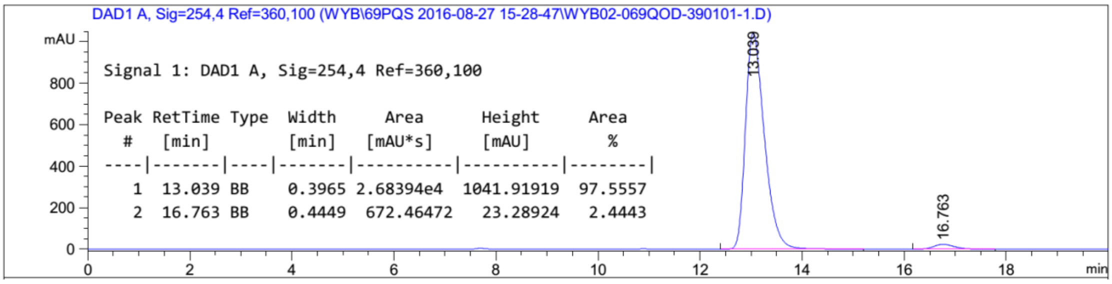

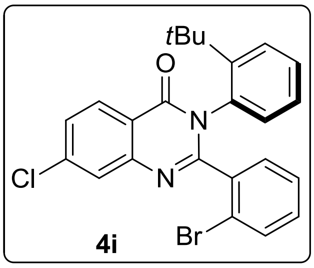


**(*aR*)-2-(2-Bromophenyl)-3-(2-(*tert*-butyl)phenyl)-7-chloroquinazolin-4(*3H*)-one (4i)**

According to Procedure D at r.t, **4i** was obtained in 94% yield and 86% ee as a white solid. The ee was determined by chiral stationary phase HPLC analysis [Daicel CHIRALCEL OD-3, hexane/isopropanol = 90/10, 1.0 mL/min, T = 25 °C, λ = 254 nm, $t_R$ (major) = 9.7 min, $t_R$ (minor) = 11.8 min]. $^1$H NMR (400 MHz, $CDCl_3$) δ 8.32 (d, J = 8.5 Hz, 1H), 7.83 (d, J = 1.9 Hz, 1H), 7.54-7.51 (m, 2H), 7.47 (dd, J = 8.5, 1.4 Hz, 1H), 7.27-7.19 (m, 3H), 7.15-7.08 (m, 2H), 7.02 (t, J = 7.3 Hz, 1H), 1.33 (s, 9H); $^{13}$C NMR (101 MHz, $CDCl_3$) δ 162.8, 155.2, 148.3, 146.2, 141.2, 136.1, 133.8, 133.3, 130.8, 130.6, 129.6, 129.5, 129.1, 128.9, 128.4, 127.6, 127.0, 126.7, 123.1, 120.0, 36.9, 32.2; HRMS (m/z): $[M]^+$ calcd for $C_{24}H_{21}ON_2BrCl$ 467.0520; found 467.0505.

*Chiral HPLC spectrum of racemic* ***4i***

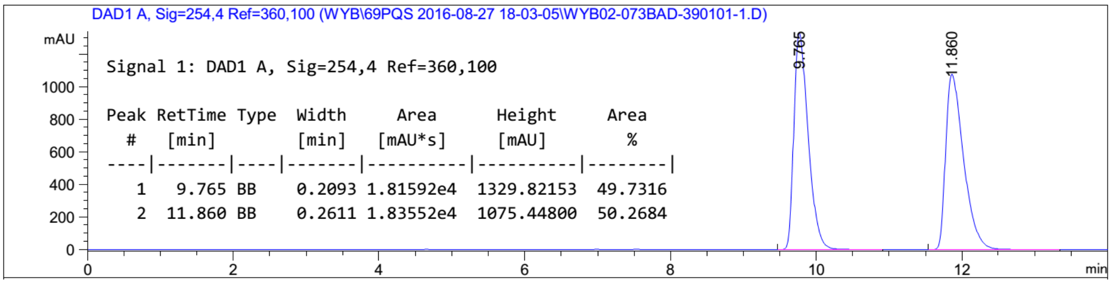


*Chiral HPLC spectrum of* ***4i***

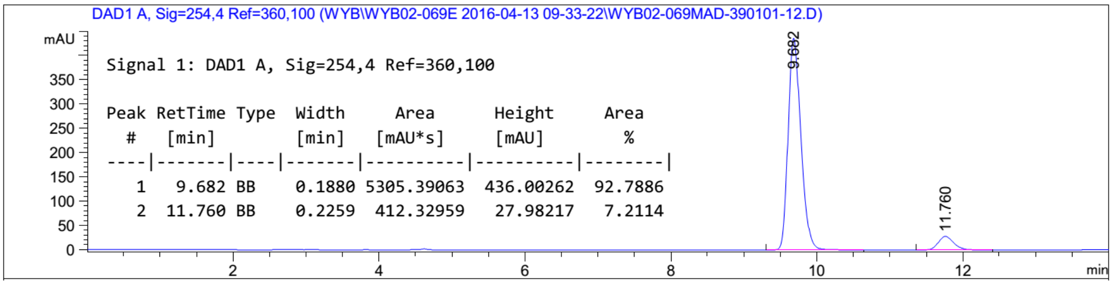


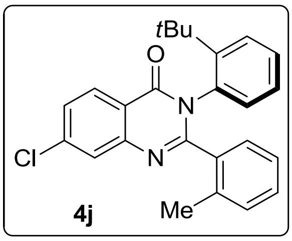


**(*aR*)-3-(2-(*tert*-Butyl)phenyl)-7-chloro-2-(*o*-tolyl)quinazolin-4(*3H*)-one (4j)**

According to Procedure D, **4j** was obtained in 86% yield and 85% ee as a colorless oil. The ee was determined by chiral stationary phase HPLC analysis [Daicel CHIRALCEL AD-3, hexane/isopropanol = 90/10, 1.0 mL/min, T = 25 °C, λ = 254 nm, $t_R$ (major) = 8.0 min, $t_R$ (minor) = 11.0 min]. $^1$H NMR (400 MHz, $CDCl_3$) δ 8.31 (d, J = 8.5 Hz, 1H), 7.80 (d, J = 1.9 Hz, 1H), 7.51-7.46 (m, 2H), 7.22-7.09

(m, 4H), 7.01-6.92 (m, 2H), 6.76 (dd, J = 7.9 Hz, 1.1 Hz, 1H), 2.38 (s, 3H), 1.33 (s, 9H); $^{13}$C NMR (101 MHz, $CDCl_3$) δ 163.1, 156.4, 148.4, 146.4, 141.1, 136.4, 134.4, 134.1, 130.9, 130.6, 129.9, 129.3, 129.2, 128.9, 128.1, 127.9, 127.4, 126.7, 125.0, 119.6, 36.9, 32.1, 20.4; HRMS (m/z): $[M]^+$ calcd for $C_{25}H_{24}ON_2ClF$ 403.1572; found 403.1559.

*Chiral HPLC spectrum of racemic **4j***

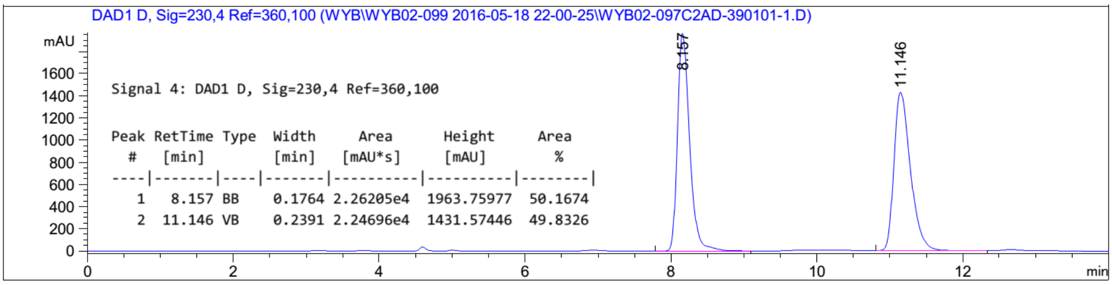


*Chiral HPLC spectrum of **4j***

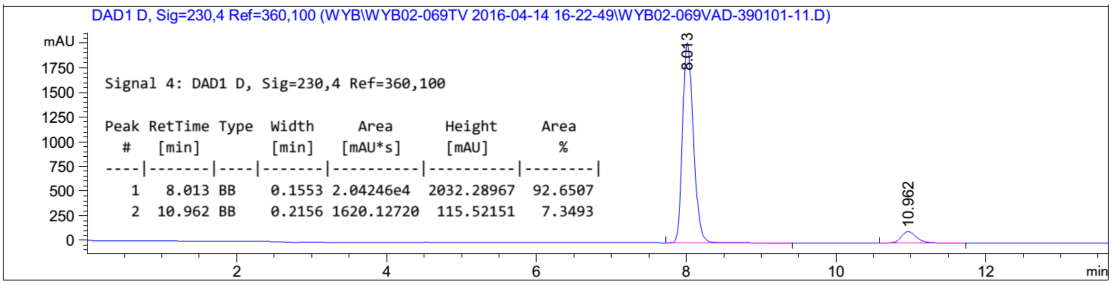


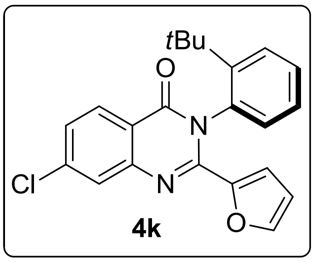


**(a*R*)-3-(2-(*tert*-Butyl)phenyl)-7-chloro-2-(furan-2-yl)quinazolin-4(3*H*)-one (4k)**

According to Procedure D, **4k** was obtained in 83% yield and 87% ee as a light brown oil. The ee was determined by chiral stationary phase HPLC analysis [Daicel CHIRALCEL OD-3, hexane/isopropanol = 90/10, 1.0 mL/min, T = 25 °C, λ = 254 nm, $t_R$ (major) = 13.5 min, $t_R$ (minor) = 15.9 min]. $^1$H NMR (400 MHz, $CDCl_3$) δ 8.24 (d, J = 8.5 Hz, 1H), 7.90 (d, J = 1.9 Hz, 1H), 7.69 (dd, J = 8.2, 1.3 Hz, 1H), 7.56 (d, J = 1.4 Hz, 1H), 7.53 (dd, J = 8.2, 1.4 Hz, 1H), 7.44 (dd, J = 8.5, 2.0 Hz, 1H), 7.37 (td, J = 7.6, 1.4 Hz, 1H), 7.08 (dd, J = 7.8, 1.4 Hz, 1H), 1.16 (s, 9H); $^{13}$C NMR (101 MHz, $CDCl_3$) δ 162.6, 148.8, 147.2, 147.1, 146.1, 145.9, 141.2, 134.8, 131.4, 130.6, 130.3, 128.8, 127.8, 127.7, 127.4, 119.2, 117.8, 112.1, 36.6, 31.8; HRMS (m/z): $[M]^+$ calcd for $C_{22}H_{20}O_2N_2Cl$ 379.1208; found 379.1196.

*Chiral HPLC spectrum of racemic **4k***

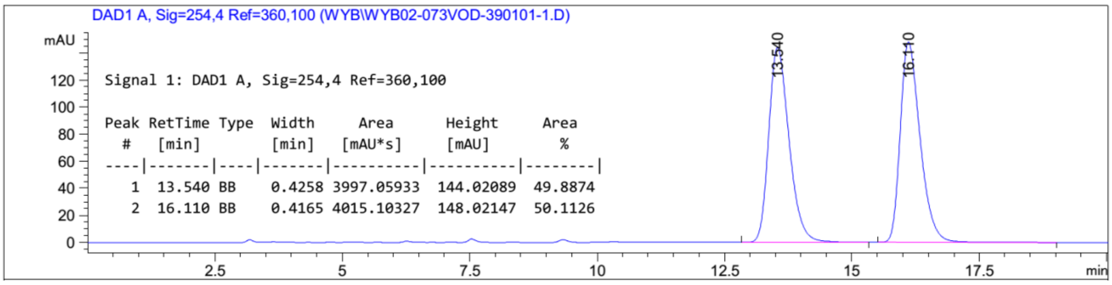


*Chiral HPLC spectrum of **4k***

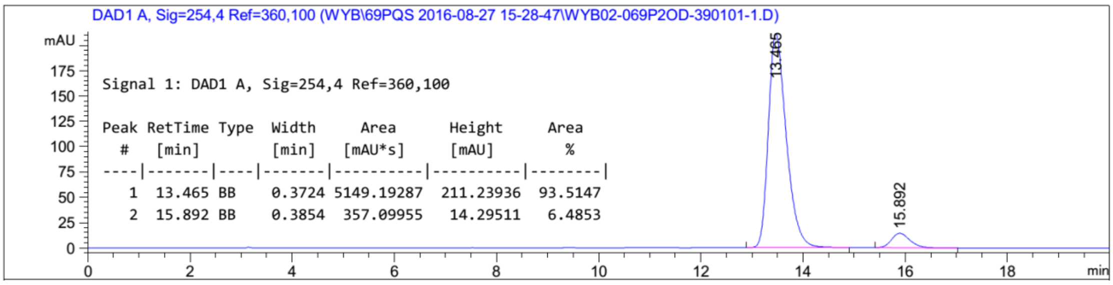


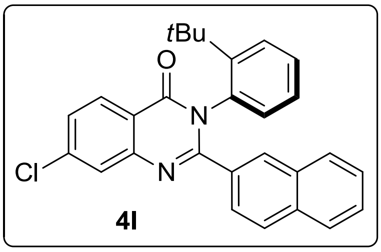


**(*aR*)-3-(2-(*tert*-butyl)phenyl)-7-chloro-2-(naphthalen-2-yl)quinazolin-4(*3H*)-one (4l)**

According to Procedure D **4l** was obtained in 96% yield and 96% ee as a colorless oil. The ee was determined by chiral stationary phase HPLC analysis [Daicel CHIRALCEL OD-3, hexane/isopropanol = 90/10, 1.0 mL/min, T = 25 °C, λ = 254 nm, $t_R$ (major) = 16.2 min, $t_R$ (minor) = 24.7 min]. $^1$H NMR (400 MHz, $CDCl_3$) δ 8.31 (d, J = 8.5 Hz, 1H), 7.85 (dd, J = 2.4, 1.8 Hz, 1H), 7.74 (d, J = 7.9 Hz, 1H), 7.67 (t, J = 7.7 Hz, 2H), 7.53 (dd, J = 8.6, 1.8 Hz, 1H), 7.50-7.42 (m, 4H), 7.31-7.27 (m, 1H), 7.20 (td, J = 7.5, 1.5 Hz, 1H), 7.10 (dd, J = 7.8, 1.5 Hz, 1H), 1.14 (s, 9H); $^{13}$C NMR (101 MHz, $CDCl_3$) δ 163.0, 156.6, 148.9, 146.7, 141.1, 134.5, 133.4, 132.4, 132.3, 132.1, 130.8, 130.7, 129.4, 128.9, 128.8, 127.9, 127.7, 127.61, 127.56, 127.5, 126.8, 126.7, 126.5, 119.4, 36.7, 31.9; HRMS (m/z): $[M]^+$ calcd for $C_{28}H_{24}ON_2Cl$ 439.1572; found 439.1557.

*Chiral HPLC spectrum of racemic **4l***

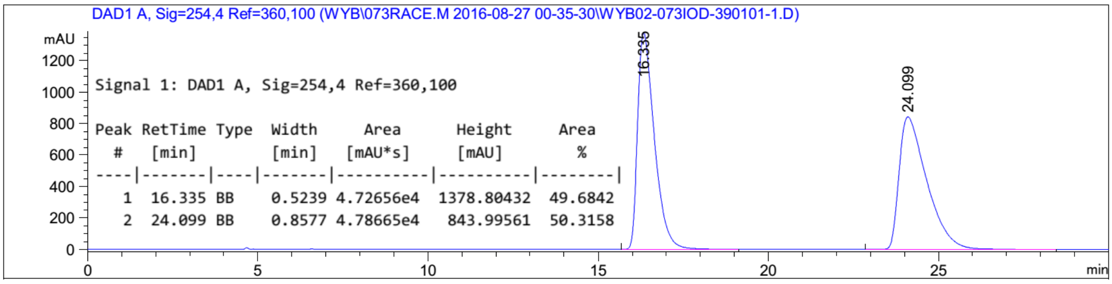


*Chiral HPLC spectrum of **4l***

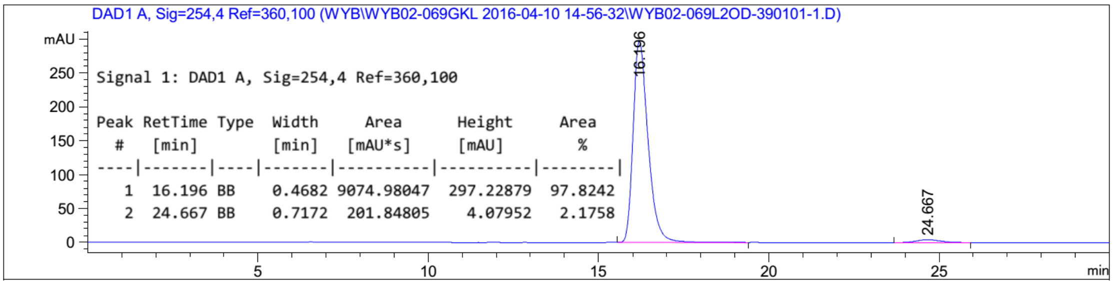


**Procedure E** (Experimental procedure for the synthesis of *racemic* **4m** and **4n**)

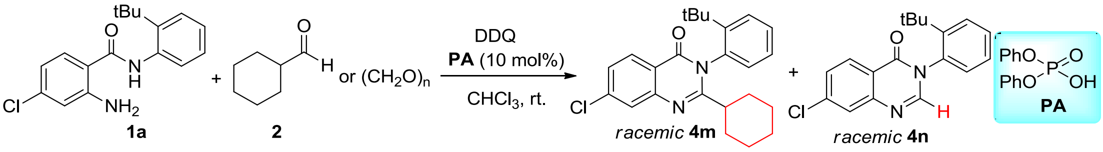


To a 2.0 mL vail, **1a** (6.0 mg, 0.02 mmol), cyclohexanecarboxaldehyde or paraformaldehyde (0.04 mmol), diphenyl phosphate (0.50 mg, 0.002 mmol) and 0.5 mL $CHCl_3$ was added. After stirred for 24 h, DDQ (9.0 mg, 0.04 mmol) was added in one portion. The reaction mixture was stirred at ambient temperature and monitored by TLC. After completion, the mixture was purified by preparative TLC on silica gel to yield the target molecular *racemic* **4m-4n**.

**Procedure F** (Experimental procedure for the asymmetric synthesis of **4m** and **4n**)

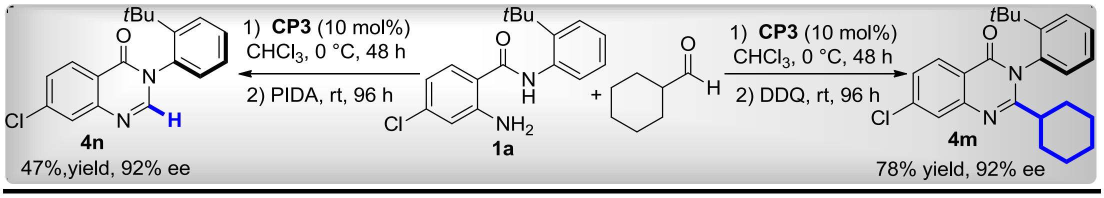


To a dry Schlenk tube (10 mL), 400 mg activated 4 Å MS (molecular sieves) was added and then the MS was reactivated at 300 ºC under reduced pressure for 15 min. After the tube was cooled down, **1a** (0.10 mmol), **CP3** (7.0 mg, 0.01 mmol) and anhydrous $CHCl_3$ (4.0 mL) was added under Ar. The resulting mixture was stirred for 10 min at 0 ºC, and then cyclohexanecarboxaldehyde (24.2 μL, 0.2 mmol) was added in one portion under Ar. After stirred for 48 h at 0 ºC, the mixture was warmed to room temperature and treated with oxidant (3.0 mmol, DDQ or $PhI(OAc)_2$). After stirred for 96 h at room temperature, the reaction mixture directly purified through flash column chromatography on silica gel (gradient elution with PE/EA) to give the pure product **4m** (DDQ as oxidant) or **4n** ($PhI(OAc)_2$ as oxidant).

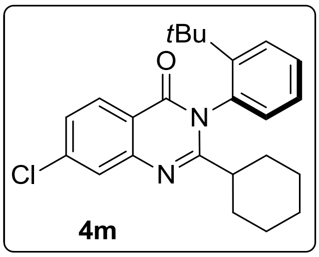


**(*aR*)-3-(2-(*tert*-Butyl)phenyl)-7-chloro-2-cyclohexylquinazolin-4(*3H*)-one (4m)**

According the above procedure, **4m** was obtained in 78% yield and 92% ee as a yellow oil. The ee was

determined by chiral stationary phase HPLC analysis [Daicel CHIRALCEL OD-3, hexane/isopropanol = 90/10, 1.0 mL/min, T = 25 °C, λ = 254 nm, $t_R$ (minor) = 6.4 min, $t_R$ (major) = 8.9 min]. $^1$H NMR (400 MHz, $CDCl_3$) δ 8.19 (d, J = 8.5 Hz, 1H), 7.72 (d, J = 2.0 Hz, 1H), 7.67 (dd, J = 8.2, 1.4 Hz, 1H), 7.48-7.44 (m, 1H), 7.38 (dd, J = 8.5, 2.0 Hz, 1H), 7.32 (td, J = 7.6, 1.5 Hz, 1H), 6.97 (dd, J = 7.8, 1.4 Hz, 1H), 2.16 (tt, J = 11.3, 3.0 Hz, 1H), 2.07-1.97 (m, 1H), 1.87-1.58 (m, 5H), 1.39 (qd, J = 13.0, 3.3 Hz, 1H), 1.27 (ddd, J = 9.4, 7.6, 3.7 Hz, 1H), 1.22 (s, 9H), 1.05 (qt, J = 13.0, 3.3 Hz, 1H), 0.85 (qt, J = 12.9, 3.5 Hz, 1H); $^{13}$C NMR (101 MHz, $CDCl_3$) δ 163.0, 162.9, 149.0, 146.4, 140.6, 134.4, 130.6, 130.3, 129.7, 128.7, 127.3, 127.0, 126.9, 119.4, 43.4, 36.6, 32.8, 32.0, 30.1, 26.1, 25.8, 25.7; HRMS (m/z): $[M]^+$ calcd for $C_{24}H_{28}ON_3Cl$ ; HRMS (m/z): $[M]^+$ calcd for $C_{24}H_{26}$ON2Cl 423.1025; found 423.1011; found 395.1871.

*Chiral HPLC spectrum of racemic **4m***

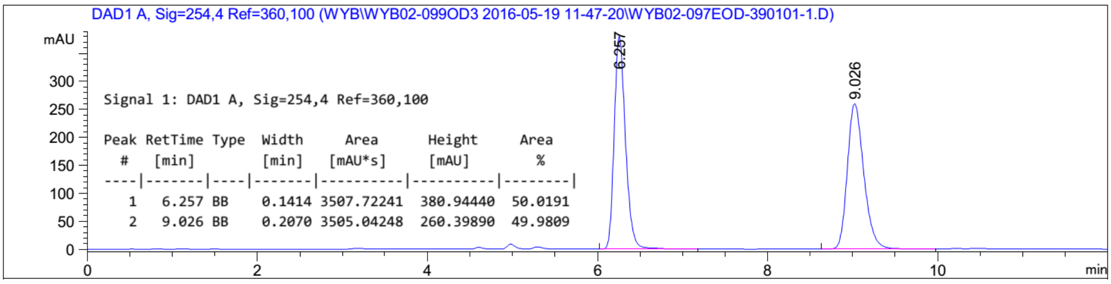


*Chiral HPLC spectrum of **4m***

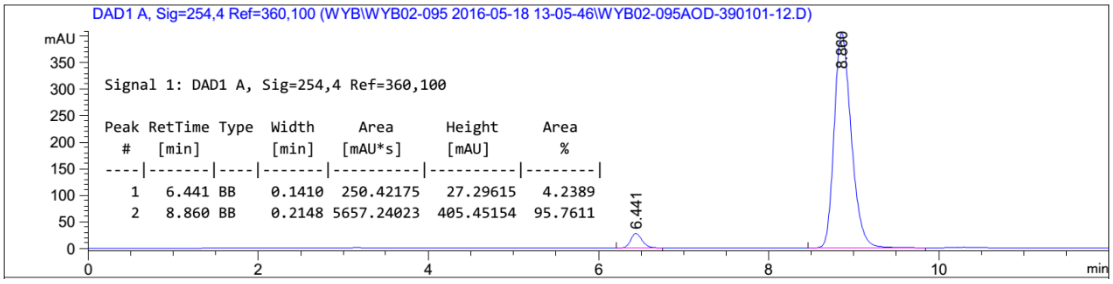


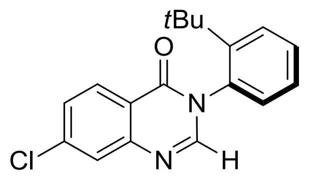


**4n**

**(*aR*)-3-(2-(*tert*-Butyl)phenyl)-7-chloroquinazolin-4(*3H*)-one (4n)**

According the above procedure, **4n** was obtained in 47% yield and 92% ee as a white solid. The ee was determined by chiral stationary phase HPLC analysis [Daicel CHIRALCEL OD-3, hexane/isopropanol = 90/10, 1.0 mL/min, T = 25 °C, λ = 254 nm, $t_R$ (minor) = 7.5 min, $t_R$ (major) = 9.6 min]. $^1$H NMR (400 MHz, $CDCl_3$) δ 8.29 (d, J = 8.5 Hz, 1H), 8.04 (s, 1H), 7.77 (d, J = 2.0 Hz, 1H), 7.67 (dd, J = 8.2, 1.4 Hz, 1H), 7.51-7.45 (m, 2H), 7.35 (td, J = 7.5, 1.4 Hz, 1H), 7.09 (dd, J = 7.8, 1.4 Hz, 1H), 1.28 (s, 10H); $^{13}$C NMR (101 MHz, $CDCl_3$) δ 161.5, 149.3, 148.7, 147.4, 141.0, 135.6, 130.9, 130.2, 129.4, 128.8, 128.3, 127.8, 127.4, 121.2, 36.2, 32.0; HRMS (m/z): $[M]^+$ calcd for $C_{18}H_{18}$ON2Cl 313.1102; found 313.1090.

*Chiral HPLC spectrum of racemic **4n***

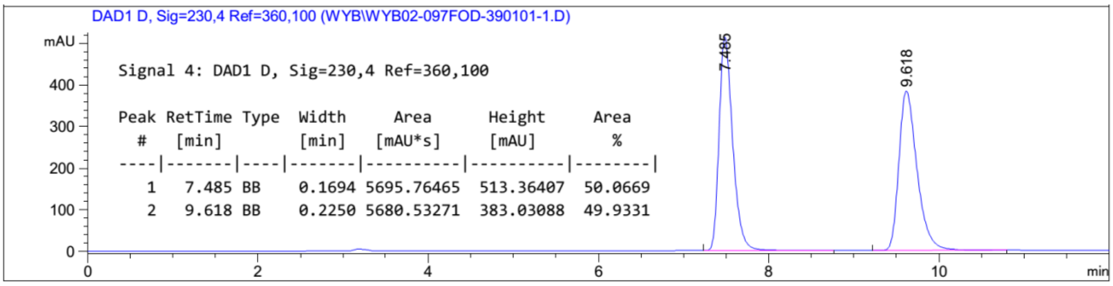


*Chiral HPLC spectrum of* ***4n***

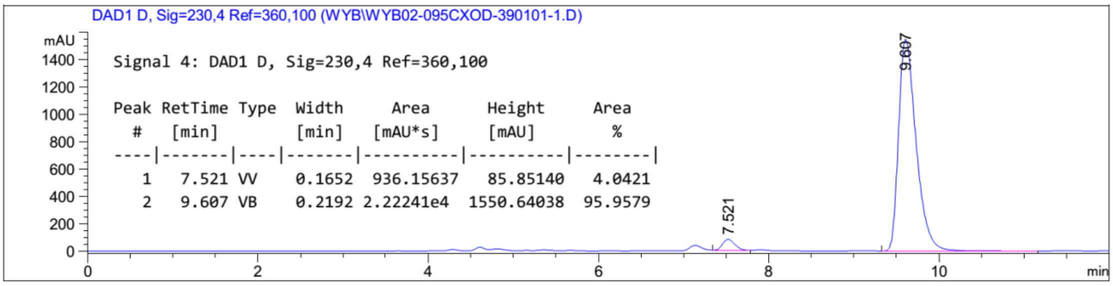


**Procedure G** (Experimental procedure for the synthesis of *racemic* **6a-6f**)

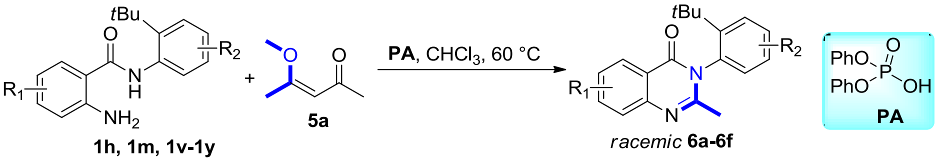


To a 10 mL dry Schlenk tube, **1h**, **1m** or **1v-1y** (0.02 mmol), **5a** (4.6 mg, 0.04 mmol), **PA** (0.5 mg, 0.002 mmol) and anhydrous $CHCl_3$ (1.0 mL) was added under Ar. The resulting solution was heated to 60 ºC. After stirred for 96 h, the solution was cooled down to room temperature and then directly purified with preparative TLC on silica gel to yield the target molecular to yield the target molecular *racemic* **6a-6f**.

**Procedure H** (Experimental procedure for the asymmetric synthesis of **6a-6f**)

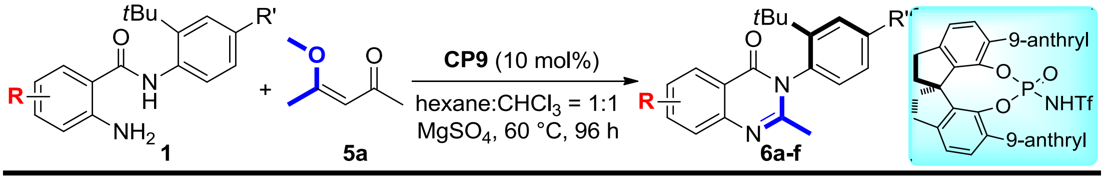


To a 10 mL dry Schlenk tube, **1h**, **1m** or **1v-1y** (0.10 mmol), **5a**[7] (22.8 mg, 0.2 mmol), $MgSO_4$ (6.0 mg), **CP9** (8.0 mg, 0.01 mmol) and anhydrous *n*-Hexane/$CHCl_3$ = 1/1 (4.0 mL) was added under Ar. The resulting solution was heated to 60 ºC. After stirred for 96 h, the solution was cooled down to room temperature and then directly purified through flash column chromatography on silica gel (gradient elution with PE/EA) to give the pure product.

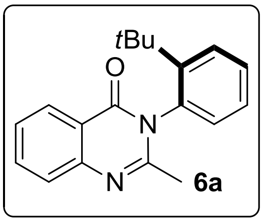

**(*aS*)-3-(2-(*tert*-Butyl)phenyl)-2-methylquinazolin-4(*3H*)-one (6a)**

According to Procedure H, **6a** was obtained in 83% yield and 83% ee as a yellowish solid. The product was recrystallized from PE and the filtrate afforded **6a** in 84% yield and 97% ee as a yellowish solid. The ee was determined by chiral stationary phase HPLC analysis [Daicel CHIRALCEL AD-3, hexane/isopropanol = 90/10, 1.0 mL/min, T = 25 °C, λ = 254 nm, $t_R$ (major) = 8.9 min, $t_R$ (minor) = 13.5 min]. $^1$H NMR (400 MHz, $CDCl_3$) δ 8.29 (dd, J = 8.0, 1.2 Hz, 1H), 7.77 (ddd, J = 8.5, 7.1, 1.5 Hz, 1H), 7.70-7.66 (m, 2H), 7.49-7.43 (m, 2H), 7.34 (td, J = 7.6, 1.5 Hz, 1H), 6.99 (dd, J = 7.8, 1.4 Hz, 1H), 2.24 (s, 3H), 1.26 (s, 9H); $^{13}$C NMR (101 MHz, $CDCl_3$) δ 163.1, 155.3, 147.7, 146.3, 135.4, 134.7, 130.5, 130.4, 129.7, 127.8, 127.3, 126.9, 126.7, 121.0, 36.5, 31.9, 25.0; HRMS (m/z): $[M]^+$ calcd for $C_{19}H_{21}ON_2$ 293.1648; found 293.1638.

*Chiral HPLC spectrum of racemic **6a***

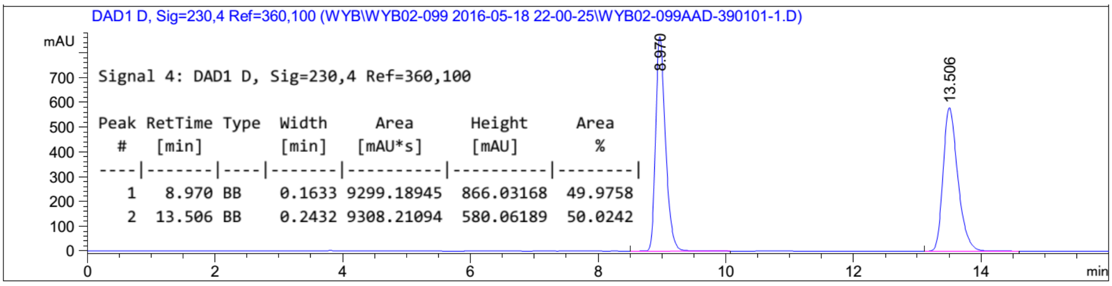


*Chiral HPLC spectrum of **6a***

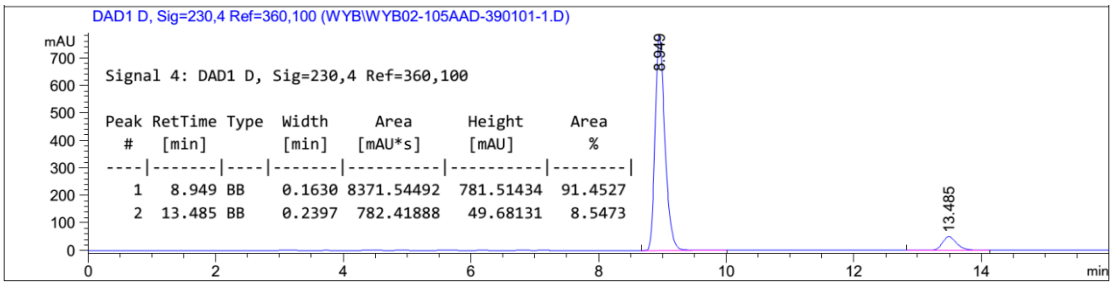


*Chiral HPLC spectrum of **6a***

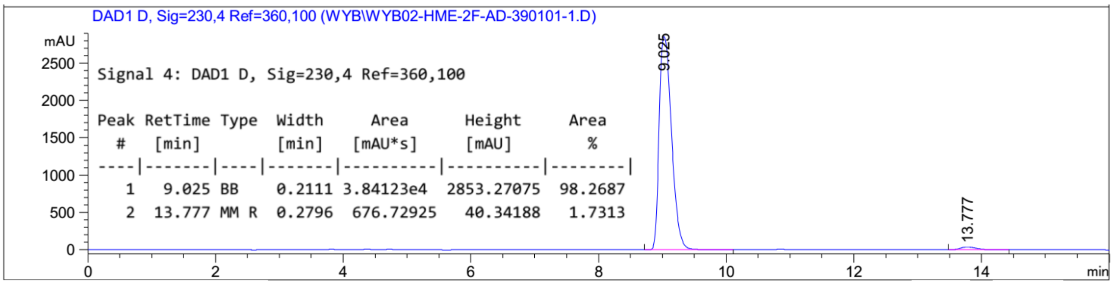


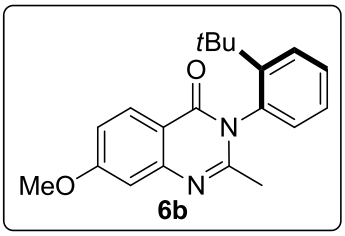


**(*aS*)-3-(2-(*tert*-Butyl)phenyl)-7-methoxy-2-methylquinazolin-4(*3H*)-one (6b)**

According to Procedure H, **6b** was obtained in 95% yield and 82% ee as a yellowish solid. The product was recrystallized from PE/DCM to afford **6b** in 64% yield and 99% ee as a colorless crystal. The ee was determined by chiral stationary phase HPLC analysis [Daicel CHIRALCEL AD-3,

hexane/isopropanol = 90/10, 1.0 mL/min, T = 25 °C, λ = 254 nm, $t_R$ (major) = 10.7 min, $t_R$ (minor) = 25.9 min].$^1$H NMR (400 MHz, $CDCl_3$) δ 8.18 (d, J = 8.8 Hz, 1H), 7.66 (d, J = 8.0 Hz, 1H), 7.44 (t, J = 7.3 Hz, 1H), 7.33 (t, J = 7.4 Hz, 1H), 7.08 (d, J = 1.8 Hz, 1H), 7.04 (dd, J = 8.8, 2.1 Hz, 1H), 6.99 (d, J = 7.6 Hz, 1H), 3.93 (s, 3H), 2.21 (s, 3H), 1.26 (s, 9H); $^{13}$C NMR (101 MHz, $CDCl_3$) δ 165.0, 162.7, 156.0, 149.9, 146.4, 135.4, 130.6, 130.3, 129.6, 128.9, 127.7, 116.8, 114.5, 107.7, 55.8, 36.5, 31.9, 25.0; HRMS (m/z): $[M]^+$ calcd for $C_{20}H_{23}O_2N_2$ 323.1754; found 323.1743.

*Chiral HPLC spectrum of racemic* ***6b***

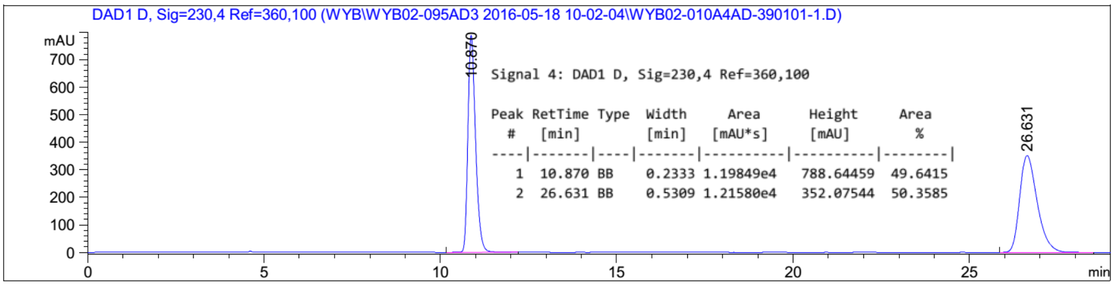


*Chiral HPLC spectrum of* ***6b***

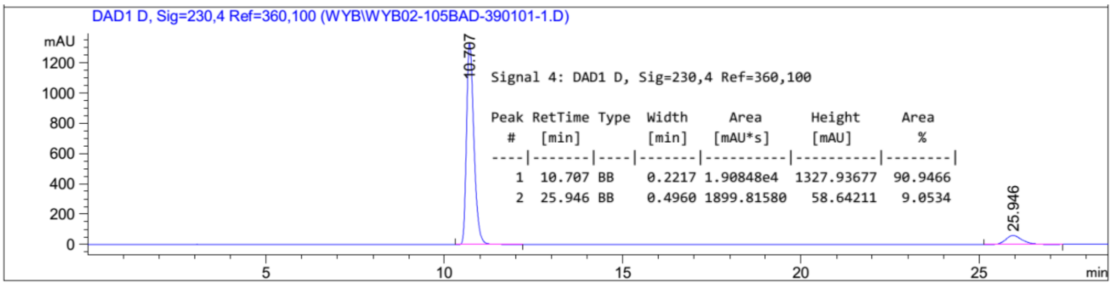


*Chiral HPLC spectrum of* ***6b***

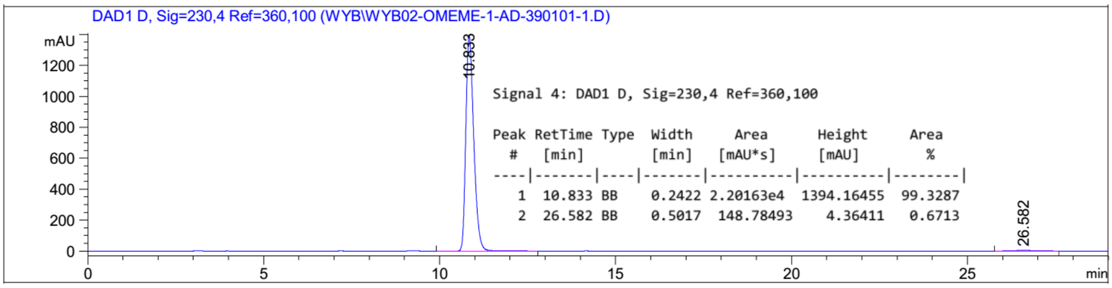


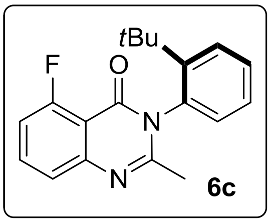


**(*aS*)-3-(2-(*tert*-Butyl)phenyl)-5-fluoro-2-methylquinazolin-4(3*H*)-one (6c)**

According to Procedure H, **6c** was obtained in 75% yield and 89% ee as a white solid. The product was recrystallized from PE to afford **6c** in 86% yield and 98% ee as a colorless crystal. The ee was determined by chiral stationary phase HPLC analysis [Daicel CHIRALCEL AD-3, hexane/isopropanol = 90/10, 1.0 mL/min, T = 25 °C, λ = 254 nm, $t_R$ (major) = 8.3 min, $t_R$ (minor) = 11.0 min]. $^1$H NMR (400 MHz, $CDCl_3$) δ 7.71-7.65 (m, 2H), 7.48-7.43 (m, 2H), 7.33 (td, J = 7.6, 1.4 Hz, 1H), 7.10 (ddd, J = 10.3, 8.3, 0.7 Hz, 1H), 6.97 (dd, J = 7.8, 1.4 Hz, 1H), 2.21 (s, 3H), 1.28 (s, 5H); $^{13}$C NMR (101 MHz, $CDCl_3$) δ 161.7 (d, J = 266.6 Hz), 160.1 (d, J = 4.2 Hz), 156.4, 149.8, 146.4, 135.0 (d, J = 10.5 Hz),

134.9, 130.5, 130.4, 129.7, 127.8, 122.9 (d, J = 4.3 Hz), 113.3 (d, J = 20.8 Hz), 110.8 (d, J = 6.0 Hz), 36.5, 32.0, 24.9; $^{19}$F NMR (376 MHz, $CDCl_3$) δ -110.0; HRMS (m/z): $[M]^+$ calcd for $C_{19}H_{20}ON_2F$ 311.1554; found 311.1543.

*Chiral HPLC spectrum of racemic **6c***

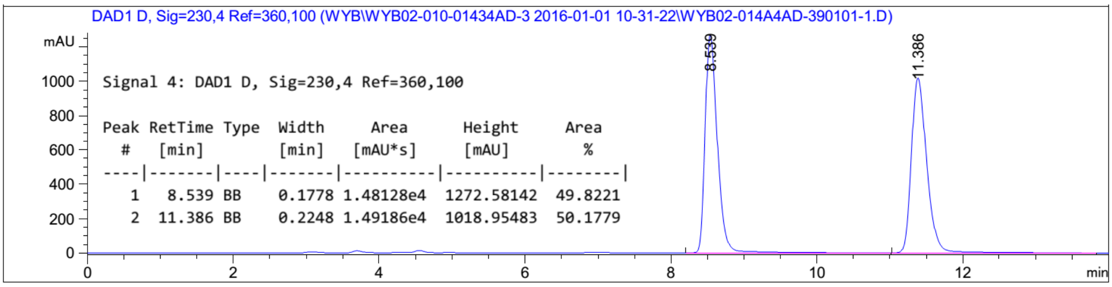


*Chiral HPLC spectrum of **6c***

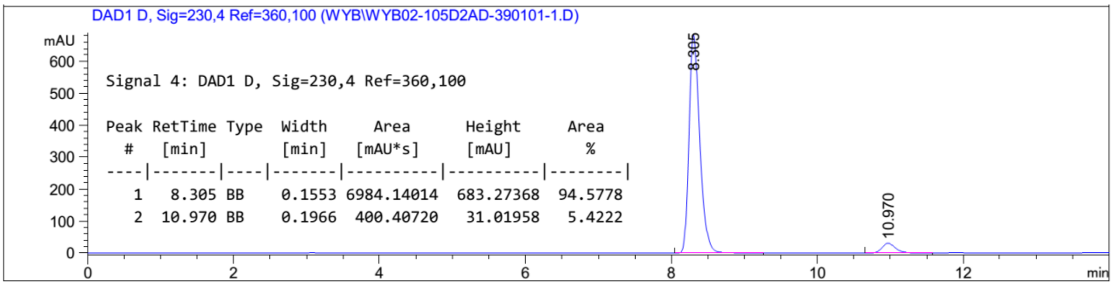


*Chiral HPLC spectrum of **6c***

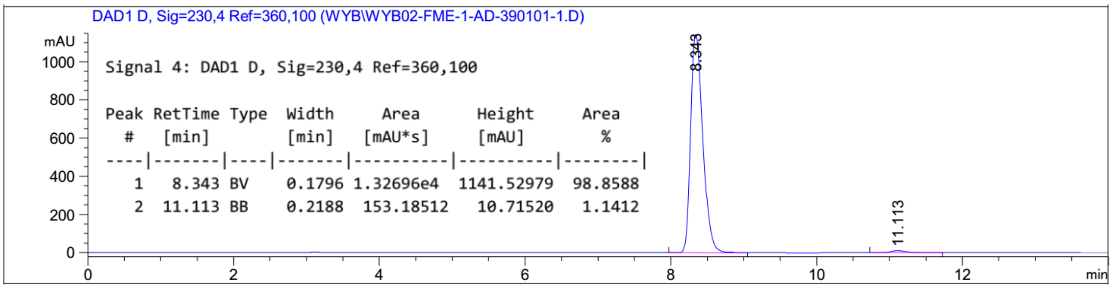


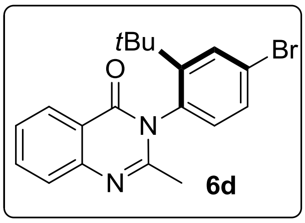


**(*aS*)-3-(4-Bromo-2-(*tert*-butyl)phenyl)-2-methylquinazolin-4(*3H*)-one (6d)**

According to Procedure H, **6d** was obtained in 82% yield and 89% ee as a yellowish solid. The product was recrystallized from PE and the filtrate afforded **6d** in 87% yield and 98% ee. The ee was determined by chiral stationary phase HPLC analysis [Daicel CHIRALCEL AD-3, hexane/isopropanol = 90/10, 1.0 mL/min, T = 25 °C, λ = 254 nm, $t_R$ (major) = 13.6 min, $t_R$ (minor) = 33.1 min]. $^1$H NMR (400 MHz, $CDCl_3$) δ 8.27 (dd, J = 8.0, 1.1 Hz, 1H), 7.80-7.76 (m, 2H), 7.68 (d, J = 7.9 Hz, 1H), 7.50-7.46 (m, 2H), 6.88 (d, J = 8.3 Hz, 1H), 2.24 (s, 3H), 1.24 (s, 9H); $^{13}$C NMR (101 MHz, $CDCl_3$) δ 163.0, 154.7, 148.8, 147.6, 134.9, 134.6, 133.6, 132.2, 131.1, 127.3, 127.0, 126.9, 123.9, 120.8, 36.7, 31.7, 24.9; HRMS (m/z): $[M]^+$ calcd for $C_{19}H_{20}ON_2Br$ 371.0754; found 371.0742.

*Chiral HPLC spectrum of racemic **6d***

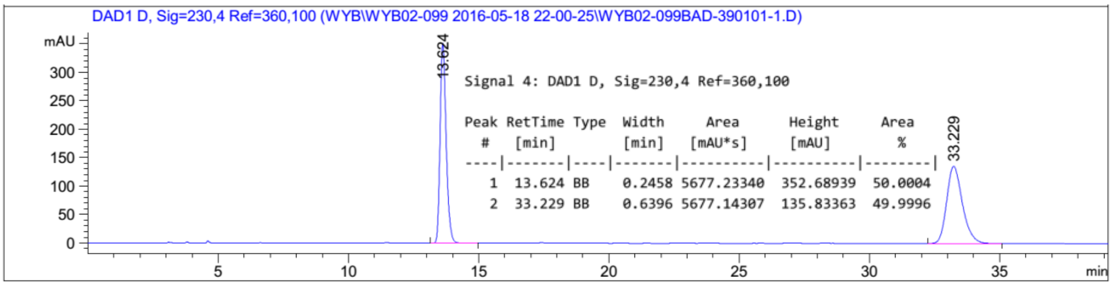


*Chiral HPLC spectrum of **6d***

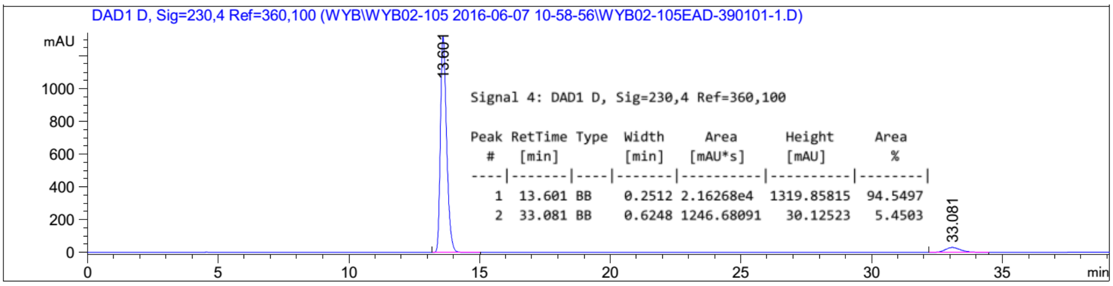


*Chiral HPLC spectrum of **6d***

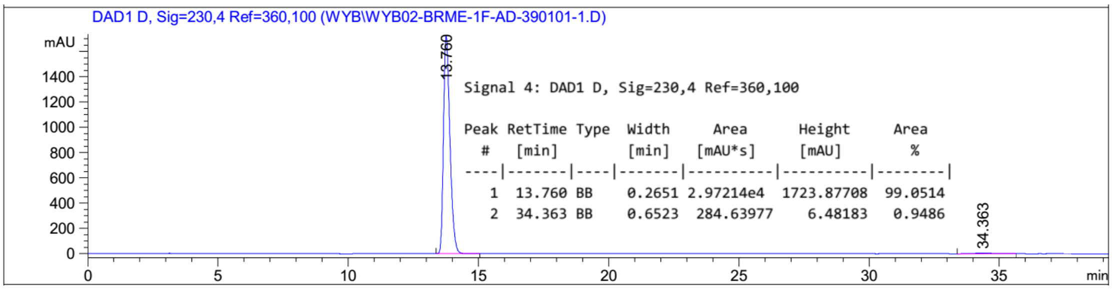


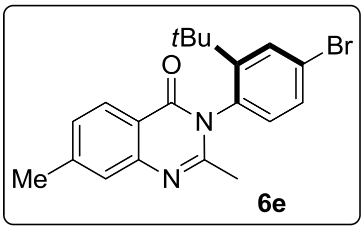


**(*aS*)-3-(4-Bromo-2-(*tert*-butyl)phenyl)-2,7-dimethylquinazolin-4(3*H*)-one (6e)**

According to Procedure H, **6e** was obtained in 80% yield and 85% ee as a yellowish oil. The ee was determined by chiral stationary phase HPLC analysis [Daicel CHIRALCEL AD-3, hexane/isopropanol = 90/10, 1.0 mL/min, T = 25 °C, λ = 254 nm, $t_R$ (major) = 12.4 min, $t_R$ (minor) = 47.0 min]. $^1$H NMR (400 MHz, $CDCl_3$) δ 8.15 (d, J = 8.1 Hz, 1H), 7.78 (d, J = 2.2 Hz, 1H), 7.48-7.45 (m, 2H), 7.30 (dd, J = 8.1, 1.1 Hz, 1H), 6.87 (d, J = 8.3 Hz, 1H), 2.52 (s, 3H), 2.22 (s, 3H), 1.23 (s, 9H); $^{13}$C NMR (101 MHz, $CDCl_3$) δ 162.9, 154.7, 148.8, 147.7, 145.9, 134.7, 133.6, 132.2, 131.0, 128.5, 127.1, 126.8, 123.8, 118.4, 36.7, 31.7, 24.9, 22.1; HRMS (m/z): $[M]^+$ calcd for $C_{20}H_{22}ON_2Br$ 385.0910; found 385.0898.

*Chiral HPLC spectrum of racemic **6e***

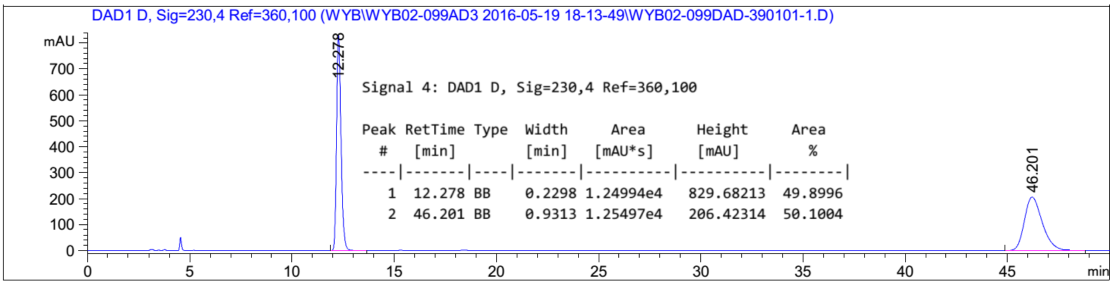


*Chiral HPLC spectrum of* ***6e***

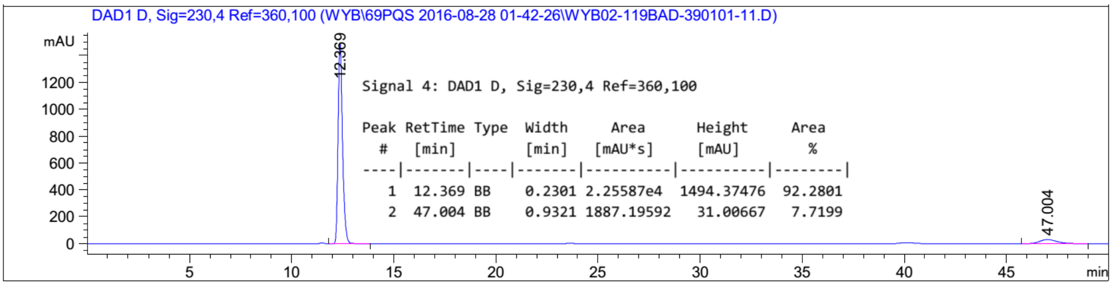


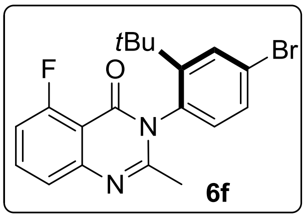


**(*aS*)-3-(4-Bromo-2-(*tert*-butyl)phenyl)-5-fluoro-2-methylquinazolin-4(*3H*)-one (6f)**

According to Procedure H, **6f** was obtained in 80% yield and 95% ee as a white solid. The ee was determined by chiral stationary phase HPLC analysis [Daicel CHIRALCEL AD-3, hexane/isopropanol = 90/10, 1.0 mL/min, T = 25 °C, $\lambda$ = 254 nm, $t_R$ (major) = 14.4 min, $t_R$ (minor) = 26.3 min]. $^{1}$H NMR (400 MHz, $CDCl_3$) $\delta$ 7.78 (d, J = 2.2 Hz, 1H), 7.69 (td, J = 8.2, 5.4 Hz, 1H), 7.47 (dd, J = 8.3, 2.3 Hz, 2H), 7.11 (ddd, J = 10.3, 8.3, 0.7 Hz, 1H), 6.86 (d, J = 8.3 Hz, 1H), 2.21 (s, 3H), 1.26 (s, 10H); $^{13}$C NMR (101 MHz, $CDCl_3$) $\delta$ 161.6 (d, J = 266.8 Hz), 159.9 (d, J = 4.3 Hz), 155.9, 149.6, 148.8, 135.2 (d, J = 10.4 Hz), 134.1, 133.7, 132.2, 131.1, 124.0, 122.9 (d, J = 4.3 Hz), 113.5 (d, J = 20.8 Hz), 110.6 (d, J = 6.2 Hz), 36.7, 31.8, 24.9; $^{19}$F NMR (376 MHz, $CDCl_3$) $\delta$ -109.8; HRMS (m/z): $[M]^+$ calcd for $C_{19}H_{19}ON_2BrF$ 389.0659; found 389.0647.

*Chiral HPLC spectrum of racemic* ***6f***

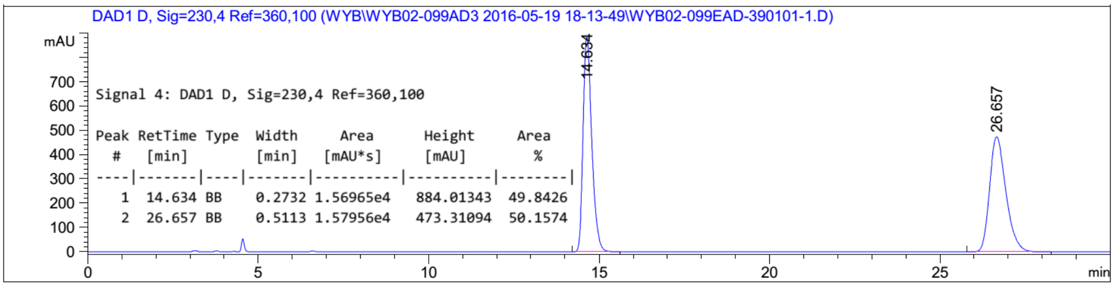


*Chiral HPLC spectrum of* ***6f***

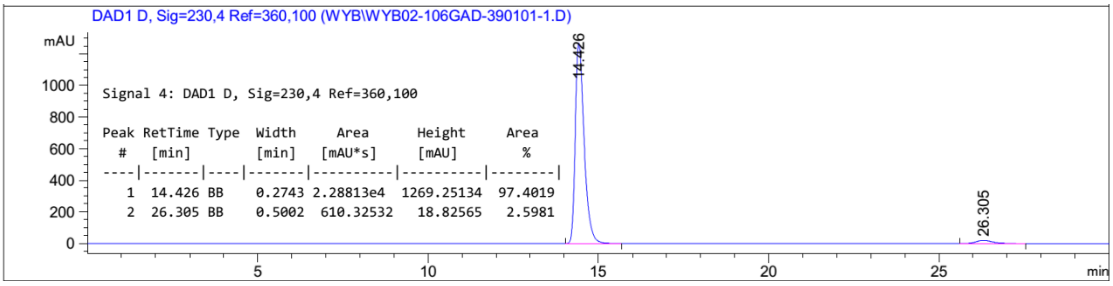


## Transformation of the compound 6f

### Procedure I

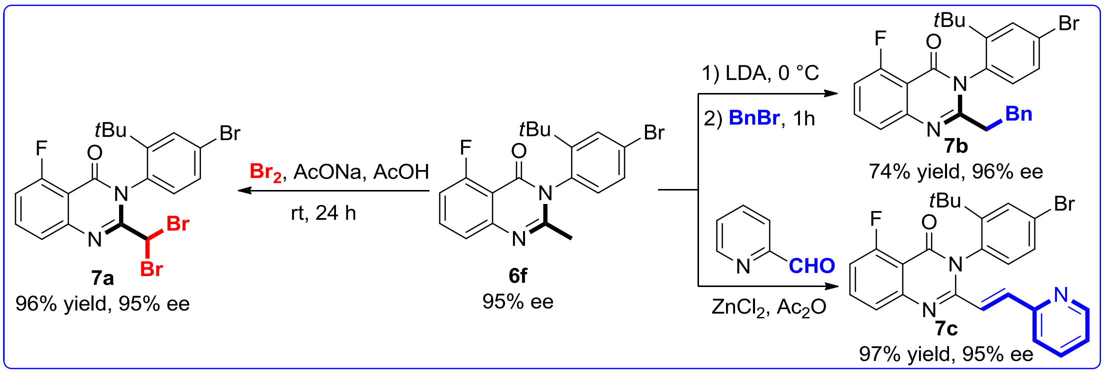


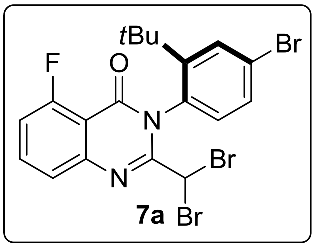


**(*aS*)-3-(4-Bromo-2-(*tert*-Butyl)phenyl)-2-(dibromomethyl)-5-fluoroquinazolin-4(3*H*)-one (7a)**

To a 10 mL round bottom flask, **6f** (95% ee, 30 mg, 0.077 mmol), AcONa (42 mg, 0.31 mmol) and 1.0 mL AcOH was added. After stirred for 5 min at room temperature, a solution of $Br_2$ (49 mg, 0.31 mmol) in 0.5 mL AcOH was added drop-wise, and the mixture was stirred for additional 12 h. The mixture was added to saturated $NaHCO_3$ solution slowly, and then the aqueous phase was extracted with DCM (2 x 30 mL). The combined organic layer was washed with saturated $Na_2S_2O_3$ solution and brine, dried over $NaSO_4$ and concentrated under reduced pressure. The crude product was purified through flash chromatography on silica gel to yield the desired product **7a** (40.6 mg, 96.4% yield, 95% ee) as a white solid[8]. The *racemic* **7a** was synthesized according to the above procedure using *racemic* **6f**. The ee was determined by chiral stationary phase HPLC analysis [Daicel CHIRALCEL AD-3, hexane/isopropanol = 90/10, 1.0 mL/min, T = 25 °C, λ = 254 nm, $t_R$ (major) = 5.6 min, $t_R$ (minor) = 8.1 min]. $^1$H NMR (400 MHz, $CDCl_3$) δ 7.82 (d, J = 2.2 Hz, 1H), 7.81-7.76 (m, 1H), 7.70 (d, J = 8.1 Hz, 1H), 7.52 (dd, J = 8.4, 2.2 Hz, 1H), 7.21 (ddd, J = 10.2, 8.1, 1.0 Hz, 1H), 7.04 (d, J = 8.4 Hz, 1H), 5.87 (s, 1H), 1.26 (s, 9 H); $^{13}$C NMR (101 MHz, $CDCl_3$) δ 161.5 (d, $^1J_{CF}$ = 267.9 Hz), 159.1 (d, $^3J_{CF}$ = 4.4 Hz), 153.1, 149.5, 148.9, 135.7 (d, $^3J_{CF}$ = 10.3 Hz), 133.8, 133.0, 131.4, 131.2, 125.1, 124.3 (d, $^4J_{CF}$ = 4.3 Hz), 115.2 (d, $^2J_{CF}$ = 20.6 Hz), 110.8 (d, $^2J_{CF}$ = 6.2 Hz), 36.8, 34.7, 32.0; $^{19}$F NMR (376 MHz, $CDCl_3$) δ -109.1;

HRMS (m/z): $[M]^+$ calcd for $C_{19}H_{17}ON_2Br_3F$ 544.8870; found 544.8852

*Chiral HPLC spectrum of racemic* ***7a***

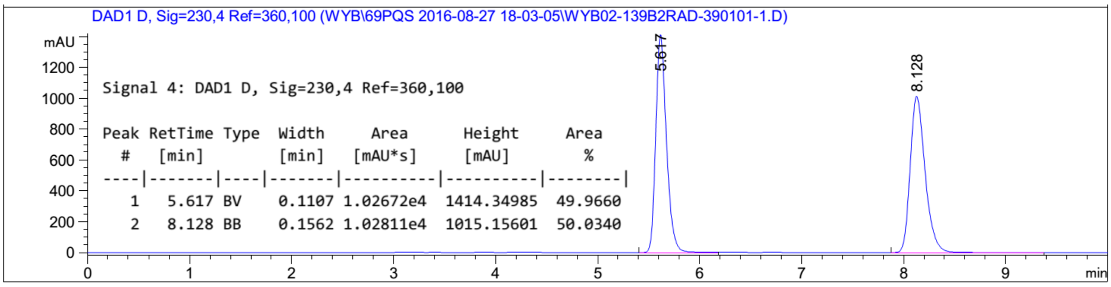


*Chiral HPLC spectrum of* ***7a***

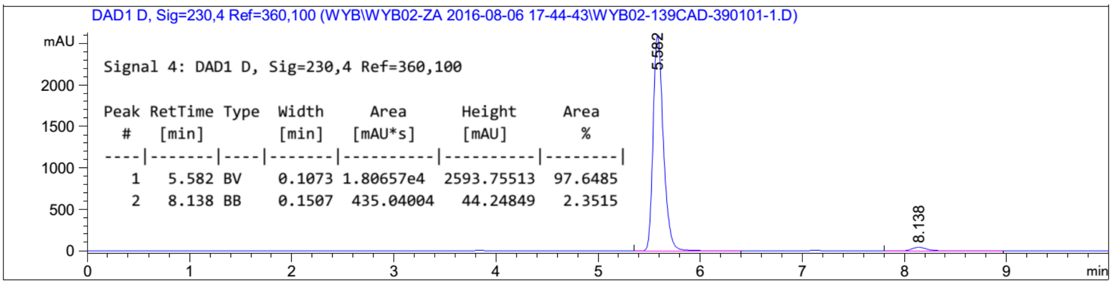


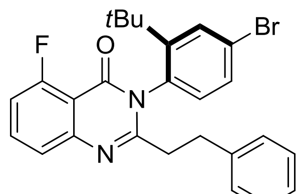


**7b**

**(*aS*)-3-(4-Bromo-2-(*tert*-Butyl)phenyl)-5-fluoro-2-phenethylquinazolin-4(*3H*)-one (7b)**

To a 10 mL anhydrous Schlenk tube, **6f** (95% ee, 30 mg, 0.077 mmol) and 1.0 mL anhydrous THF was added under Ar. 1.0 M LDA in THF/hexane (0.094 mL mL, 0.094 mmol) was added to the solution at 0 ºC in ice-water bath under Ar. After stirred for 1.0 h, BnBr (11 μL, 0.93 mmol) was added slowly. Stirred for additional 1 h at 0 ºC, the reaction was treated with 4.0 mL $H_2O$ drop-wise. And then the mixture was extracted with DCM (2 x 8 mL), the organic layer was combined, dried over $NaSO_4$ and concentrated under reduced pressure. The residue was purified through flash chromatography on silica gel to yield the desired product **7b** (27.2 mg, 74.2% yield, 96 % ee) as a yellow oil[9]. The *racemic* **7b** was synthesized according to the above procedure using *racemic* **6f**. The ee was determined by chiral stationary phase HPLC analysis [Daicel CHIRALCEL AD-3, hexane/isopropanol = 90/10, 1.0 mL/min, T = 25 °C, λ = 254 nm, $t_R$ (major) = 7.9 min, $t_R$ (minor) = 12.3 min]. $^1$H NMR (400 MHz, $CDCl_3$) δ 7.74-7.69 (m, 2H), 7.56 (d, J = 8.1 Hz, 1H), 7.38 (dd, J = 8.3, 1.9 Hz, 1H), 7.24 (t, J = 6.6 Hz, 2H), 7.19 (d, J = 7.0 Hz, 1H), 7.16-7.10 (m, 1H), 7.07 (d, J = 7.0 Hz, 2H), 6.59 (d, J = 8.3 Hz, 1H), 3.18-3.04 (m, 2H), 2.69-2.53 (m, 2H), 1.20 (s, 9H); $^{13}$C NMR (101 MHz, $CDCl_3$) δ 161.6 (d, $^1J_{CF}$ = 266.9 Hz), 160.0 (d, $^3J_{CF}$ = 4.3 Hz), 157.5, 149.6, 148.8, 140.6, 135.1 (d, $^3J_{CF}$ = 10.4 Hz), 133.5, 132.4, 130.9, 128.7, 126.5, 123.9, 123.2 (d, $^4J_{CF}$ = 4.1 Hz), 113.5 (d, $^2J_{CF}$ = 20.7 Hz), 110.6 (d, $^2J_{CF}$ = 5.8 Hz), 38.2, 36.7, 32.8, 31.7; $^{19}$F NMR (376 MHz, $CDCl_3$) δ -109.7; HRMS (m/z): $[M]^+$ calcd for $C_{26}H_{25}ON_2BrF$ 479.1129; found 479.1114.

*Chiral HPLC spectrum of racemic* ***7b***

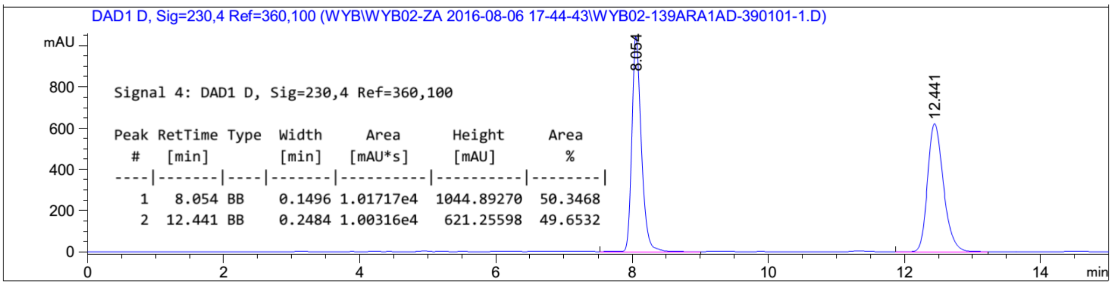


*Chiral HPLC spectrum of **7b***

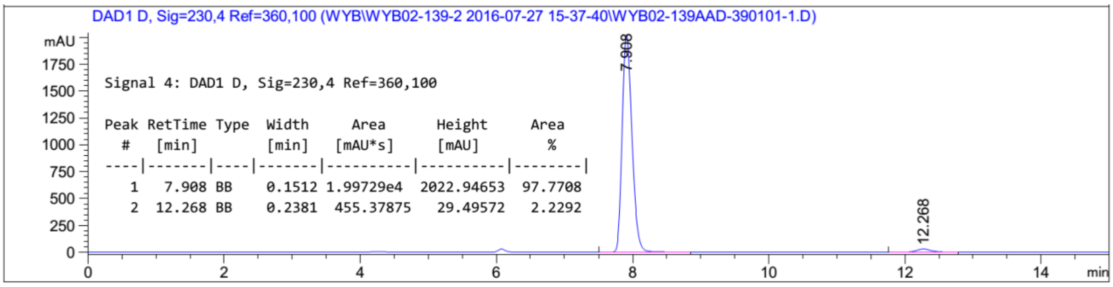


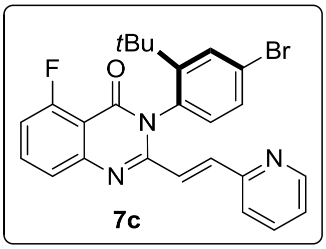


**(*aS*)-3-(4-Bromo-2-(*tert*-Butyl)phenyl)-5-fluoro-2-phenethylquinazolin-4(*3H*)-one (7c)**

Anhydrous $ZnCl_2$ (40.9 mg, 0.30 mmol) was added to a 10 mL anhydrous Schlenk tube and the solid was heated to 300 °C under reduced pressure for 10 min to remove the water. After the tube was cooled to room temerature, **6f** (95% ee, 38.9 mg, 0.10 mmol), pyridine-2-carboxaldehyde (32.1 mg, 0.3 mmol), $Ac_2O$ (28.6 µL, 0.3 mmol) and 3.0 mL anhydrous 1,4-dioxane was added under Ar. The reaction solution was heated to 100 ºC and stirred for 4 h. The resulting mixture was slowly added to saturated 15 mL $NaHCO_3$ and the mixture was extracted with EA (3 x 10 mL). The combined organic layer was washed with $H_2O$ (10 mL) and brine (10 mL), dried over $Na_2SO_4$ and concentrated under reduced pressure. The residue was purified through flash chromatography on silica gel to yield the desired product **7c** (46.5 mg, 97% yield, 95 % ee) as a yellowish oil.[10] The *racemic* **7c** was synthesized according to the above procedure using *racemic* **6f**. The ee was determined by chiral stationary phase HPLC analysis [Daicel CHIRALCEL OD-3, hexane/isopropanol = 90/10, 1.0 mL/min, T = 25 °C, λ = 254 nm, $t_R$ (major) = 9.3 min, $t_R$ (minor) = 17.6 min]. $^1H$ NMR (400 MHz, $CDCl_3$) δ 8.55 (d, 4.6, 0.8 Hz, 1H), , 8.03 (d, J = 15.0 Hz, 1H), 7.82 (d, J = 2.2 Hz, 1H), 7.72 (td, J = 8.2, 5.4 Hz, 1H), 7.65 (td, J = 7.7, 1.8 Hz, 1H), 7.59 (d, J = 8.2 Hz, 1H), 7.50 (dd, J = 8.3, 2.2 Hz, 1H), 7.31 (d, J = 7.8 Hz, 1H), 7.19 (ddd, J = 7.6, 4.8, 0.9 Hz, 1H), 7.12 (ddd, J = 10.3, 8.1, 0.8 Hz, 1H), 6.93 (d, J = 10.3 Hz, 1H), 6.91 (d, J = 3.6 Hz, 1H), 1.25 (s, 9H); $^{13}C$ NMR (101 MHz, $CDCl_3$) δ 161.7 (d, $^1J_{CF}$ = 266.6 Hz), 160.1 (d, $^3J_{CF}$ = 4.4 Hz), 153.3, 152.9 (d, $^3J_{CF}$ = 0.8 Hz), 150.33 (s), 149.8, 149.6, 140.2, 136.7, 135.2 (d, $^3J_{CF}$ = 10.4 Hz), 133.43, 133.42, 132.7, 131.2, 124.4, 124.1, 124.0, 123.6 (d, $^4J_{CF}$ = 4.2 Hz), 123.5, 113.6 (d, $^2J_{CF}$ = 20.8 Hz), 110.7 (d, $^2J_{CF}$ = 5.9 Hz), 36.7, 31.8; $^{19}F$ NMR (376 MHz, $CDCl_3$) δ -109.7.HRMS (m/z): $[M]^+$ calcd for $C_{25}H_{22}ON_3BrF$ 478.0925; found478.0928.

*Chiral HPLC spectrum of racemic* ***7c***

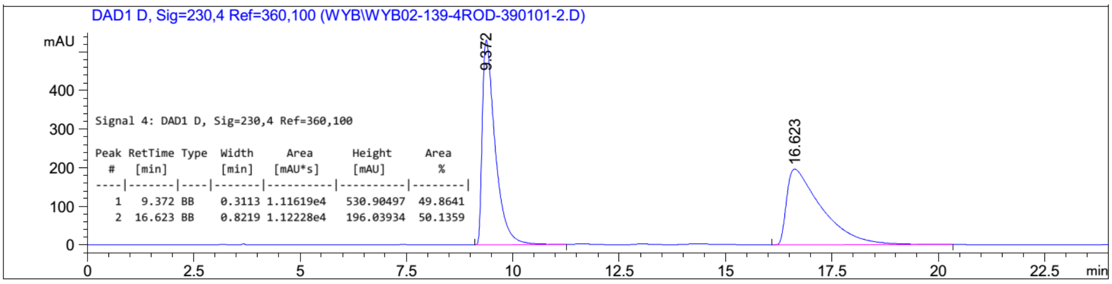


*Chiral HPLC spectrum of* ***7c***

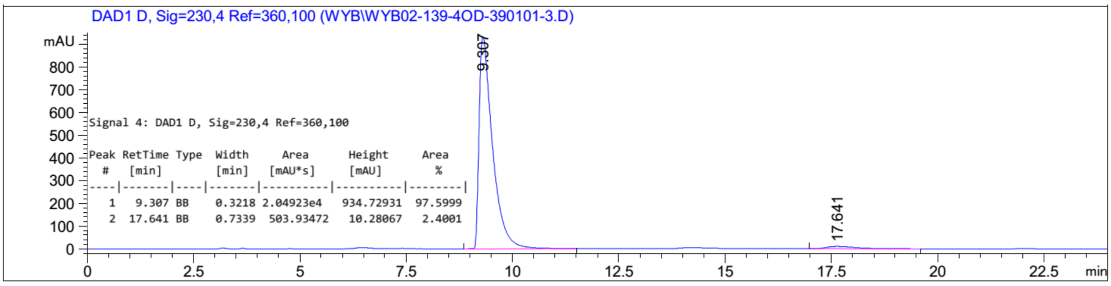

# Supplementary Note 4

## Experimental procedure for the asymmetric total synthesis of eupolyphagin.

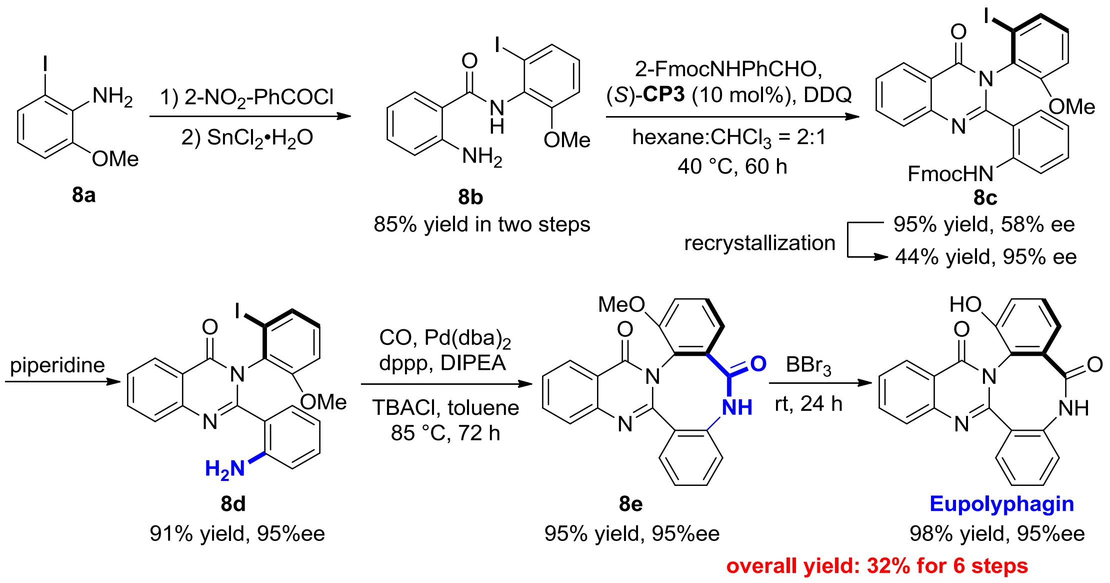


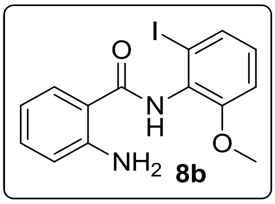


**2-Amino-*N*-(2-iodo-6-methoxyphenyl)benzamide (8b)**

According to Procedure A with **8a** which was synthesis according to the previous report[11], **8b** was obtained as a white solid in 85% overall yield for two steps. $^1$H NMR (400 MHz, $CDCl_3$) δ 7.65-7.62 (m, 1H), 7.49 (dd, J = 7.9, 1.2 Hz, 1H), 7.39 (s, 1H), 7.28-7.24 (m, 1H), 6.99 (t, J = 8.1 Hz, 1H), 6.92 (dd, J = 8.3, 1.1 Hz, 1H), 6.74-6.70 (m, 2H), 5.55 (s, 2H), 3.80 (s, 3H); $^{13}$C NMR (101 MHz, $CDCl_3$) δ 167.9, 155.4, 149.4, 133.0, 131.0, 129.4, 128.5, 128.2, 117.5, 116.7, 115.6, 111.7, 99.7, 56.2; HRMS (m/z): $[M]^+$ calcd for $C_{14}H_{14}O_2N_2$ 369.0094; found 369.0085.

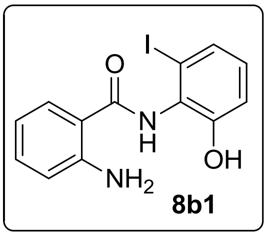


**2-amino-N-(2-hydroxy-6-iodophenyl)benzamide (8b1)**

To a 25 mL dry Schlenk tube, **8b-1** (368 mg, 1.0 mmol) and 15 mL anhydrous DCM was added under Ar, $BBr_3$ (587 µL, 5 mmol) was added slowly and the mixture was stirred for 24 h at room temperature. The solution was slowly added to 50 mL saturated $NaHCO_3$ solution and the resulting mixture was adjusted to pH 7.0 with 1M HCl. The aqueous phase was extracted with EA (3 x 20 mL), the combined organic layer was dried over $Na_2SO_4$ and concentrated under reduced pressure. The residual white solid

was purified through flash chromatography on silica gel to yield corresponding product **8b-1** as a white solid. $^{1}$H NMR (400 MHz, DMSO) δ 9.72 (s, 1H), 9.47 (s, 1H), 7.80 (d, J = 7.1 Hz, 1H), 7.34 – 7.29 (m, 1H), 7.20 – 7.16 (m, 1H), 6.92 – 6.87 (m, 2H), 6.72 (dd, J = 8.3, 1.0 Hz, 1H), 6.58 – 6.54 (m, 1H), 6.45 (s, 2H); 13C NMR (101 MHz, DMSO) δ 167.8, 154.6, 150.1, 132.0, 129.5, 129.0, 128.6, 127.8, 116.3, 116.1, 114.4, 114.2, 102.7; HRMS (m/z): $[M]^+$ calcd for $C_{13}H_{12}O_2N_2I$ 354.9938; found 354.9931.

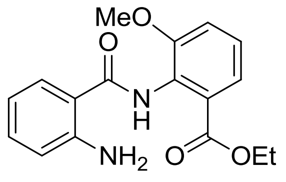


**8b2**

**2-Amino-N-(2- ethoxycarbonyl -6- hydroxyphenyl)benzamide (8b2)**

According to Procedure A, **8b-2** was obtained as a white solid. $^{1}$H NMR (400 MHz, DMSO) δ 9.44 (s, 1H), 7.76 (d, J = 8.0 Hz, 1H), 7.35 – 7.17 (m, 4H), 6.73 (d, J = 8.2 Hz, 1H), 6.57 (t, J = 7.5 Hz, 1H), 6.39 (s, 2H), 4.13 (q, J = 7.1 Hz, 2H), 3.81 (s, 3H), 1.12 (t, J = 7.1 Hz, 3H); $^{13}$C NMR (101 MHz, DMSO) δ 167.7, 166.3, 154.2, 150.0, 132.2, 129.2, 128.9, 126.3, 125.8, 121.2, 116.4, 114.9, 114.6, 114.3, 60.5, 56.0, 13.87; HRMS (m/z): $[M]^+$ calcd for $C_{17}H_{19}O_4N_2$ 315.1339; found 315.1331.

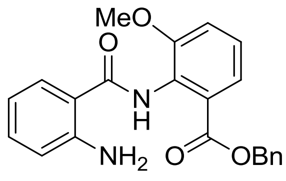


**8b3**

**2-Amino-N-(2- benzyloxycarbonyl -6- hydroxyphenyl)benzamide (8b3)**

According to Procedure A, **8b-3** was obtained as a white solid. $^{1}$H NMR (400 MHz, DMSO) δ 9.46 (s, 1H), 7.77 – 7.68 (m, 1H), 7.37 (dd, J = 7.1, 2.1 Hz, 1H), 7.35 – 7.26 (m, 7H)., 7.22 – 7.18 (m, 1H), 6.75 (d, J = 8.2 Hz, 1H), 6.55 (t, J = 7.5 Hz, 1H), 6.42 (s, 2H), 5.18 (s, 2H), 3.81 (s, 3H); $^{13}$C NMR (101 MHz, DMSO) δ 167.7, 166.0, 154.2, 150.1, 135.8, 132.2, 128.9, 128.8, 128.3, 128.0, 127.9, 126.3, 125.9, 121.1, 116.4, 115.1, 114.6, 114.2, 66.2, 56.1; HRMS (m/z): $[M]^+$ calcd for $C_{22}H_{11}O_4N_2$ 377.1496; found 3691488.

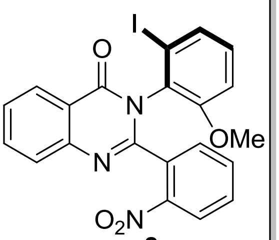


**8c-a**

**(*aS*)-3-(2-Iodo-6-methoxyphenyl)-2-(2-Nitrophenyl)quinazolin-4(*3H*)-one (8c-a)**

According to Procedure D in anhydrous c-Hexane:$CHCl_3$ = 1:1 (4.0 mL) at 25 °C for 96 h, **8c-a** was obtained in 99% yield and 31% ee when (*S*)-**CP3** was used as catalyst. The ee was determined by chiral stationary phase HPLC analysis [Daicel CHIRALCEL AD-3, hexane/isopropanol = 80/20, 1.0 mL/min, T = 25 °C, λ = 254 nm, $t_R$ (minor) = 23.5 min, $t_R$ (major) = 38.4 min]. $^1$H NMR (500 MHz, $CDCl_3$) δ 8.42 – 8.40 (m, 1H), 8.01 (dd, J = 8.2, 0.9 Hz, 1H), 7.88 – 7.81 (m, 3H), 7.60 – 7.57 (m, 1H), 7.53 (td, J = 7.6, 1.1 Hz, 1H), 7.46 (td, J = 8.1, 1.3 Hz, 1H), 7.34 (d, J = 7.9 Hz, 1H), 6.93 (t, J = 8.2 Hz, 1H), 6.73 (d, J = 8.3 Hz, 1H), 3.62 (s, 3H). $^{13}$C NMR (126 MHz, $CDCl_3$) δ 160.8, 156.2, 151.4, 147.4, 146.6, 135.0, 132.9, 131.9, 131.2, 130.78, 130.77, 129.4, 128.6, 127.9, 127.7, 127.6, 124.6, 121.7, 111.6, 101.3, 55.94; HRMS (m/z): $[M]^+$ calcd for $C_{21}H_{15}O_4N_3I$ 500.0102; found 500.0096.

*Chiral HPLC spectrum of racemic **8c-a***

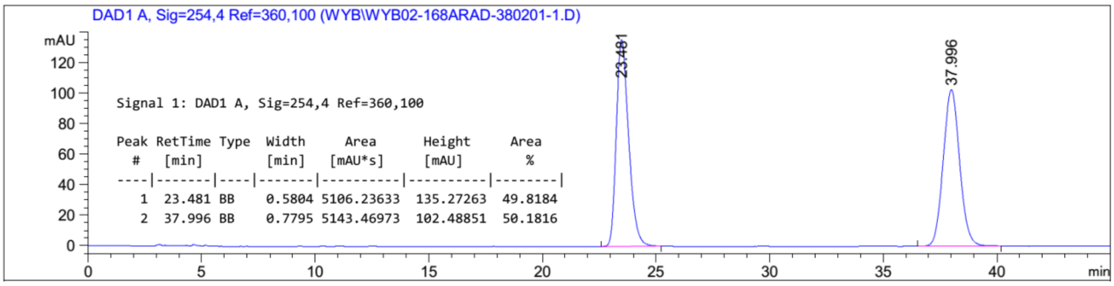


*Chiral HPLC spectrum of **8c-a***

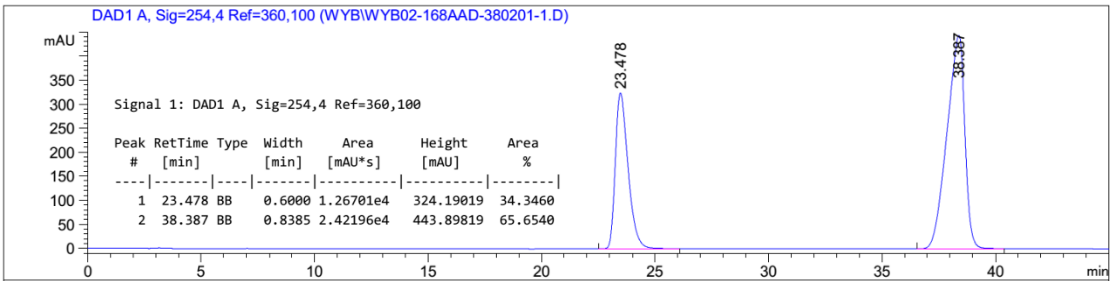


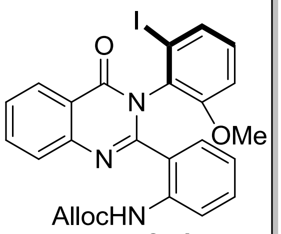


**8c-b**

**(*aS*)-2-(2-(Allyloxycarbonyl)amino)-3-(2-Iodo-6-methoxyphenyl)quinazolin-4(*3H*)-one (8c-b)**

According to Procedure D in anhydrous c-Hexane:$CHCl_3$ = 1:1 (4.0 mL) at 25 °C for 96 h, **8c-b** was obtained in 94% yield and 56% ee when (*S*)-**CP3** was used as catalyst. The ee was determined by chiral stationary phase HPLC analysis [Daicel CHIRALCEL AD-3, hexane/isopropanol = 80/20, 1.0 mL/min, T = 25 °C, λ = 254 nm, $t_R$ (minor) = 11.2 min, $t_R$ (major) = 15.0 min]. $^1$H NMR (500 MHz, $CDCl_3$) δ 8.64 (s, 1H), 8.40 (dd, J = 8.0, 1.1 Hz, 1H), 8.07 (d, J = 7.7 Hz, 1H), 7.89 – 7.84 (m, 1H), 7.84 – 7.82 (m, 1H), 7.60 – 7.57 (m, 1H), 7.42 (dd, J = 8.0, 0.7 Hz, 1H), 7.26 – 7.23 (m, 1H), 7.20 (dd, J = 7.9, 1.3 Hz, 1H), 6.97 (t, J = 8.2 Hz, 1H), 6.77 – 6.73 (m, 2H), 5.94 (ddt, J = 16.2, 10.6, 5.8 Hz, 1H), 5.32 (dd, J = 17.2, 1.4 Hz, 1H), 5.22 (dd, J = 10.4, 1.1 Hz, 1H), 4.68 – 4.60 (m, 2H), 3.61 (s, 3H); $^{13}$C NMR (126 MHz, $CDCl_3$) δ 161.1, 155.5, 153.5, 153.3, 146.7, 136.4, 135.1, 132.6, 131.4, 131.3, 130.7, 129.7,

128.6, 127.74, 127.64, 127.56, 122.9, 121.8, 121.4, 120.9, 118.5, 111.6, 101.2, 65.9, 56.0; HRMS (m/z): $[M]^+$ calcd for $C_{25}H_{21}O_4N_3I$ 554.0571; found 554.0564.

*Chiral HPLC spectrum of racemic **8c-b***

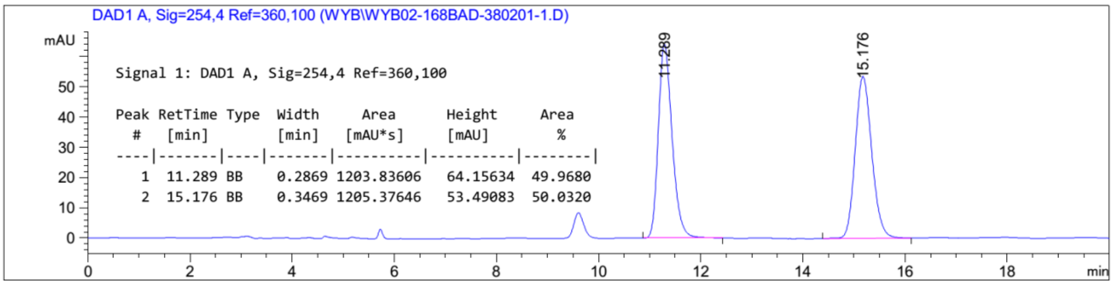


*Chiral HPLC spectrum of **8c-b***

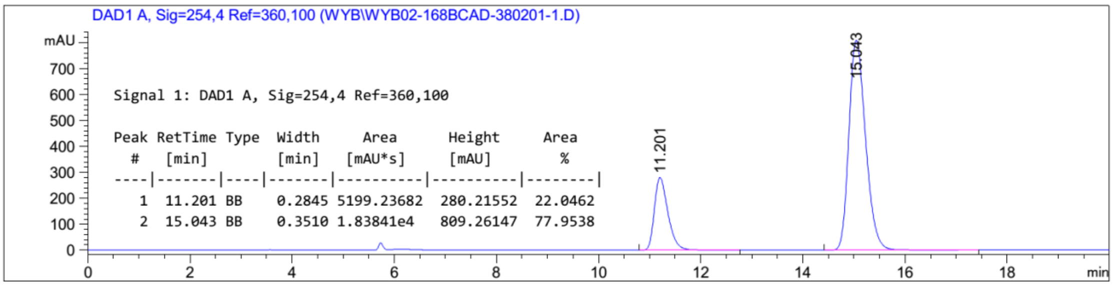


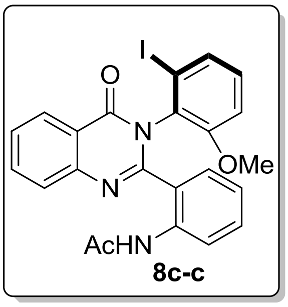


**(*aS*)-2-(2-(Acetamido)-3-(2-Iodo-6-methoxyphenyl)quinazolin-4(*3H*)-one (8c-c)**

According to Procedure D in anhydrous c-Hexane:$CHCl_3$ = 1:1 (4.0 mL) at 25 °C for 96 h, **8c-c** was obtained in 97% yield and 50% ee when (*S*)-**CP3** was used as catalyst. The ee was determined by chiral stationary phase HPLC analysis [Daicel CHIRALCEL AD-3, hexane/isopropanol = 80/20, 1.0 mL/min, T = 25 °C, λ = 254 nm, $t_R$ (minor) = 13.9 min, $t_R$ (major) = 17.7 min]. $^1H$ NMR (500 MHz, $CDCl_3$) δ 9.47 (s, 1H), 8.41 (dd, J = 7.9, 1.0 Hz, 1H), 8.23 (d, J = 8.3 Hz, 1H), 7.89 – 7.85 (m, 1H), 7.81 (d, J = 7.9 Hz, 1H), 7.61 – 7.58 (m, 1H), 7.42 (d, J = 8.0 Hz, 1H), 7.26 – 7.23 (m, 1H), 7.18 (d, J = 7.8 Hz, 1H), 6.98 (t, J = 8.2 Hz, 1H), 6.77 (t, J = 8.0 Hz, 2H), 3.60 (s, 3H), 2.13 (s, 3H); $^{13}C$ NMR (126 MHz, $CDCl_3$) δ 168.0, 167.0, 155.5, 153.7, 146.6, 136.3, 135.2, 131.5, 131.3, 130.7, 129.6, 128.5, 127.82, 127.78, 127.1, 123.0, 122.4, 122.4, 121.4, 111.7, 101.1, 56.2, 25.1; HRMS (m/z): $[M]^+$ calcd for $C_{23}H_{19}O_3N_3I$ 512.0466; found 512.0457.

*Chiral HPLC spectrum of racemic **8c-c***

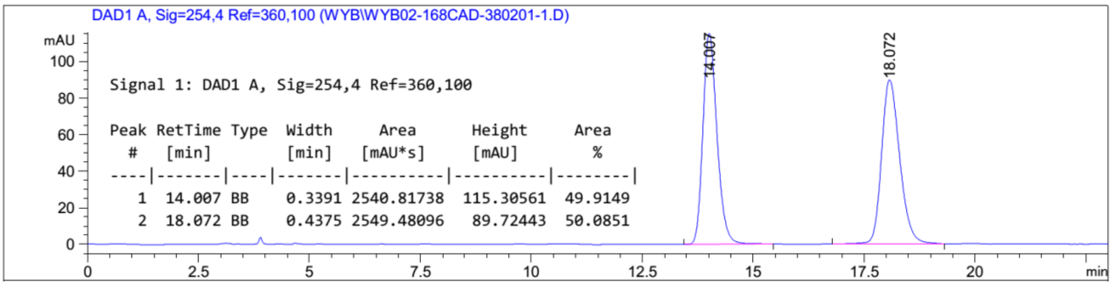


*Chiral HPLC spectrum of **8c-c***

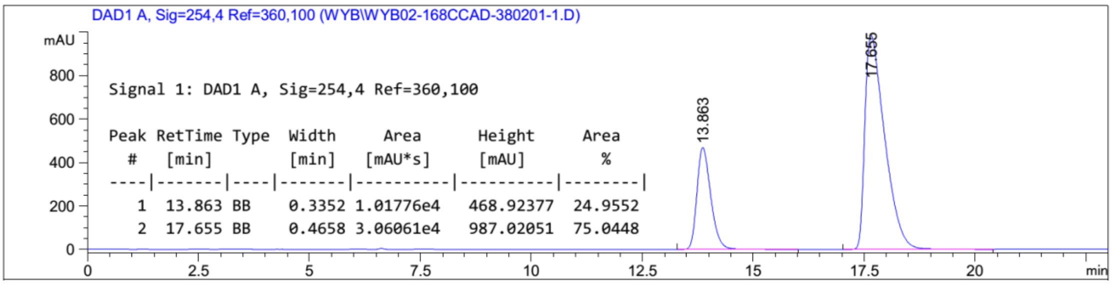


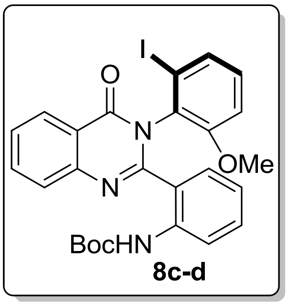


**(*aS*)-2-(2-(*tert*-Butyloxycarbonyl)amino)-3-(2-Iodo-6-methoxyphenyl)quinazolin-4(*3H*)-one (8c-d)**

According to Procedure D in anhydrous *c*-Hexane:$CHCl_3$ = 1:1 (4.0 mL) at 25 °C for 96 h, **8c-d** was obtained in 91% yield and 52% ee when (*S*)-**CP3** was used as catalyst. The ee was determined by chiral stationary phase HPLC analysis [Daicel CHIRALCEL AD-3, hexane/isopropanol = 90/10, 1.0 mL/min, T = 25 °C, λ = 254 nm, $t_R$ (minor) = 10.3 min, $t_R$ (major) = 13.4 min]. $^1$H NMR (500 MHz, $CDCl_3$) δ 8.41 (dd, J = 8.0, 1.1 Hz, 1H), 8.36 (s, 1H), 8.07 (d, J = 8.3 Hz, 1H), 7.88 – 7.85 (m, 1H), 7.82 (d, J = 7.1 Hz, 1H), 7.60 – 7.57 (m, 1H), 7.43 (dd, J = 8.0, 0.9 Hz, 1H), 7.23 – 7.20 (m, 1H), 7.18 (dd, J = 7.9, 1.4 Hz, 1H), 6.97 (t, J = 8.2 Hz, 1H), 6.75 (d, J = 8.0 Hz, 1H), 6.73 – 6.69 (m, 1H), 3.61 (s, 3H), 1.48 (s, 9H); $^{13}$C NMR (126 MHz, $CDCl_3$) δ 161.2, 155.4, 153.7, 152.8, 146.9, 136.9, 135.1, 131.4, 131.2, 130.6, 129.7, 128.6, 127.63, 127.62, 127.5, 122.6, 121.4, 121.3, 120.8, 111.6, 101.1, 80.6, 55.9, 28.5; HRMS (m/z): $[M]^+$ calcd for $C_{26}H_{25}O_4N_3I$ 570.0884; found 570.0879.

*Chiral HPLC spectrum of racemic **8c-d***

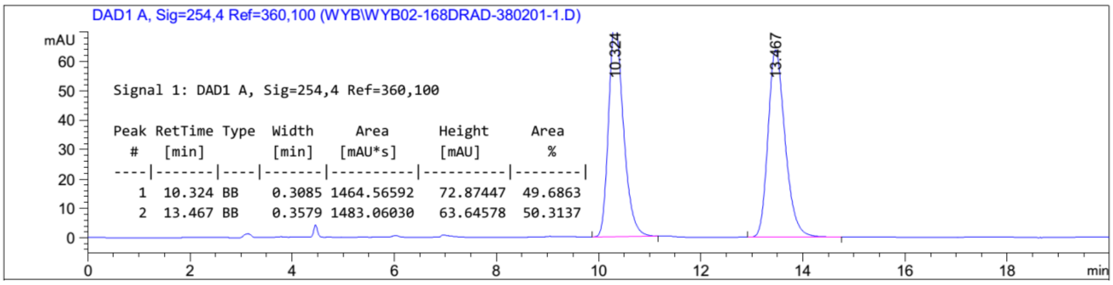


*Chiral HPLC spectrum of **8c-d***

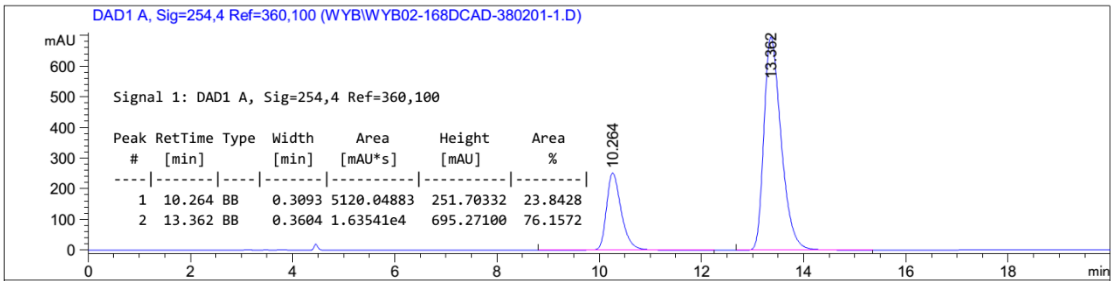


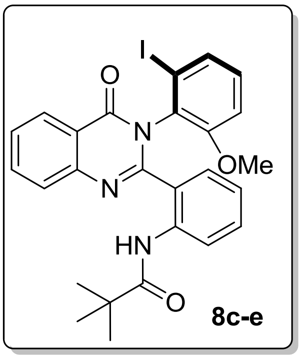


**(*aS*)-2-(2-pivaloylamino)-3-(2-iodo-6-methoxyphenyl)quinazolin-4(*3H*)-one (8c-e)**

According to Procedure D in anhydrous c-Hexane:$CHCl_3$ = 1:1 (4.0 mL) at 25 °C for 96 h, **8c-e** was obtained in 87% yield and 42% ee when (*S*)-**CP3** was used as catalyst. The ee was determined by chiral stationary phase HPLC analysis [Daicel CHIRALCEL AD-3, hexane/isopropanol = 90/10, 1.0 mL/min, T = 25 °C, λ = 254 nm, $t_R$ (minor) = 15.2 min, $t_R$ (major) = 20.8 min]. $^1$H NMR (500 MHz, $CDCl_3$) δ 10.06 (s, 1H), 8.41 (dd, J = 7.9, 1.0 Hz, 1H), 8.33 (d, J = 8.3 Hz, 1H), 7.88 – 7.85 (m, 1H), 7.78 (d, J = 8.0 Hz, 1H), 7.59 (t, J = 7.5 Hz, 1H), 7.42 (d, J = 8.0 Hz, 1H), 7.26 – 7.23 (m, 1H), 7.14 (dd, J = 7.9, 1.2 Hz, 1H), 6.99 (t, J = 8.2 Hz, 1H), 6.78 (d, J = 8.3 Hz, 1H), 6.74 (t, J = 7.6 Hz, 1H), 3.60 (s, 3H), 1.24 (s, 9H); $^{13}$C NMR (126 MHz, $CDCl_3$) δ 176.5, 161.1, 155.6, 153.8, 146.4, 136.9, 135.3, 131.5, 131.3, 130.8, 129.7, 128.5, 127.9, 127.8, 127.0, 122.8, 122.3, 122.1, 121.3, 111.7, 101.2, 56.2, 40.0, 27.8; HRMS (m/z): $[M]^+$ calcd for $C_{26}H_{25}O_4N_3I$ 554.0935; found 554.0928.

*Chiral HPLC spectrum of racemic **8c-e***

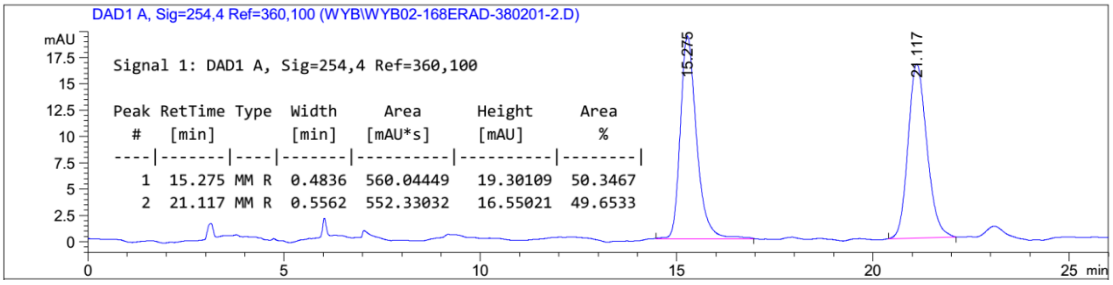


*Chiral HPLC spectrum of **8c-e***

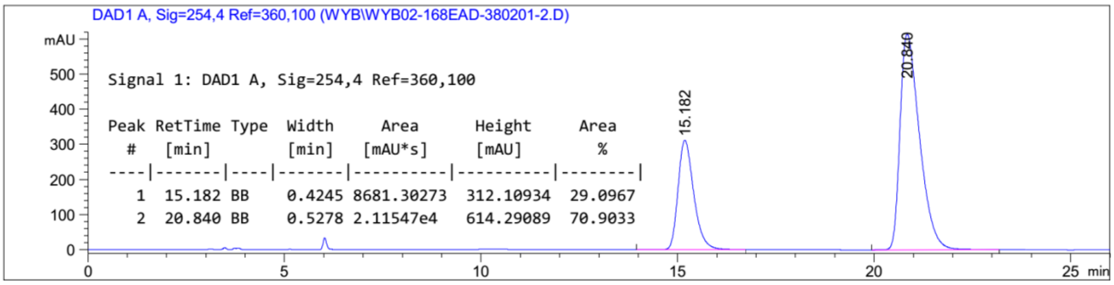

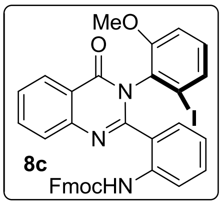


**(*aS*)-2-(2-((((9*H*-Fluoren-9-yl)methoxy)carbonyl)amino)phenyl)-3-(2-iodo-3-methoxyphenyl)quinazolin-4(*3H*)-one (8c)**

According to Procedure D at 40 °C for 60 h, **8c** was obtained in 95% yield and 58% ee when (*S*)-**CP3** was used as catalyst. The obtained solid was recrystallized from DCM/EA to afford **8c** in 44% yield and 95% ee as a white solid. The ee was determined by chiral stationary phase HPLC analysis [Daicel CHIRALCEL AD-3, hexane/isopropanol = 80/20, 1.0 mL/min, T = 25 °C, λ = 254 nm, $t_R$ (minor) = 20.2 min, $t_R$ (major) = 31.0 min]. $^1$H NMR (400 MHz, $CDCl_3$) δ 9.02 (brs, 1H), 8.44 (d, J = 7.7 Hz, 2H), 8.00 (brs, 1H), 7.91 (t, J = 7.3 Hz, 1H), 7.86 (d, J = 7.7 Hz, 1H), 7.70 (dd, J = 7.2, 1.9 Hz, 2H), 7.63 (t, J = 7.2 Hz, 1H), 7.50 (t, J = 8.1 Hz, 2H), 7.40-7.32 (m, 3H), 7.20 (dd, J = 16.2, 8.2 Hz, 2H), 7.13 (t, J = 8.2 Hz, 1H), 6.96 (t, J = 8.2 Hz, 1H), 6.75 (dd, J = 7.5, 4.7 Hz, 2H), 4.48 (dd, J = 10.1, 7.5 Hz, 1H), 4.40 (t, J = 6.2 Hz, 1H), 4.23 (t, J = 7.0 Hz, 1H), 3.53 (s, 3H); $^{13}$C NMR (101 MHz, $CDCl_3$) δ 161.1, 155.6, 153.6, 153.3, 146.7, 143.8, 143.7, 141.4, 136.5, 135.2, 131.4, 131.3, 130.9, 129.9, 128.6, 127.89, 127.87, 127.82, 127.75, 127.4, 127.16, 127.12, 125.13, 125.10, 122.8, 121.9, 121.4, 121.1, 120.1, 111.6, 101.2, 67.0, 56.0, 47.1; HRMS (m/z): $[M]^+$ calcd for $C_{36}H_{27}O_4N_3$ 692.1030; found 692.1041.

*Chiral HPLC spectrum of racemic **8c***

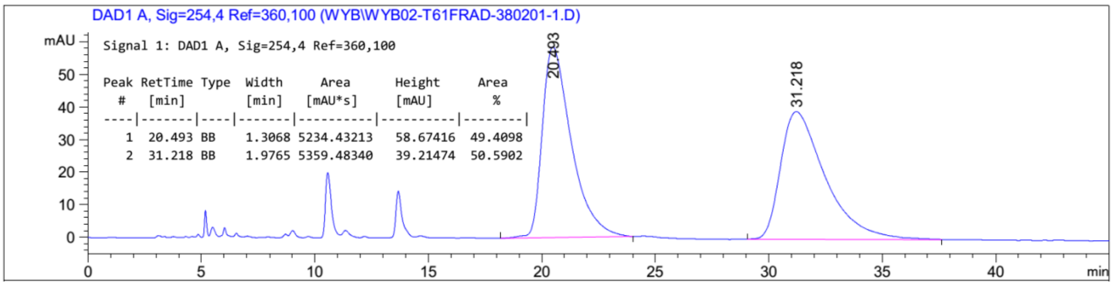


*Chiral HPLC spectrum of **8c***

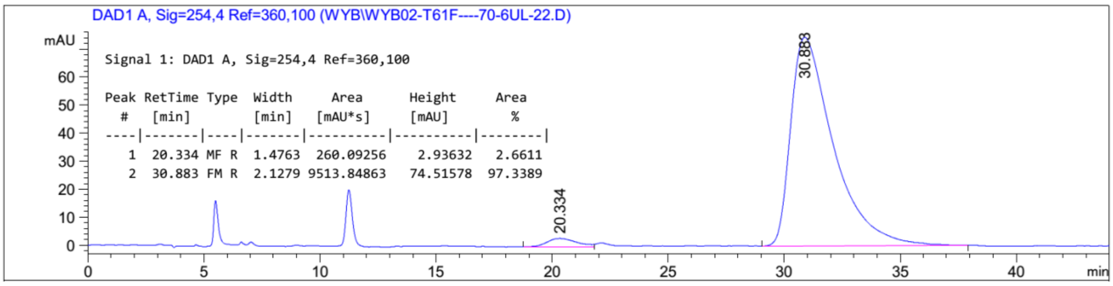


*Chiral HPLC spectrum of **8c***

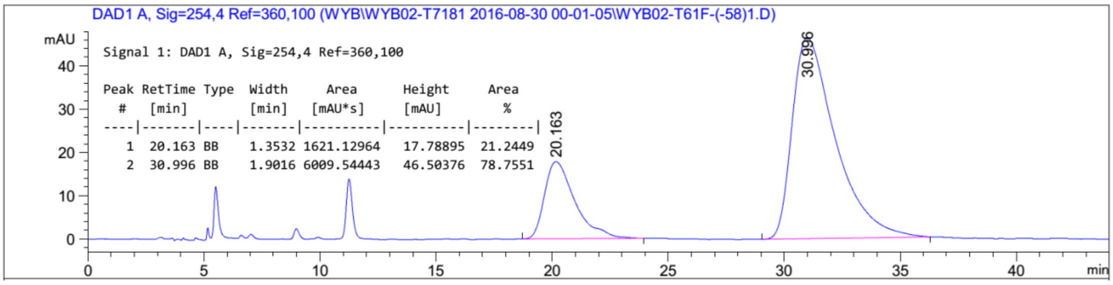

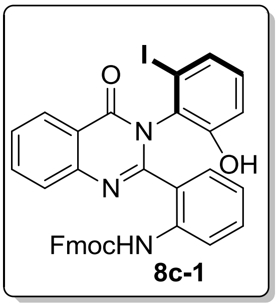


**($aS$)-2-(2-((((9$H$-Fluoren-9-yl)methoxy)carbonyl)amino)phenyl)-3-(2-hydroxy-6-iodophenyl)quinazolin-4(3$H$)-one (8c)**

To a dry Schlenk tube (10 mL), 400 mg activated 4 Å MS (molecular sieves) was added and then the MS was reactivated under reduced pressure for 15 min. After the tube was cooled down, **8b-1** (35.4 mg, 0.10 mmol), **CP3** (7.0 mg, 0.01 mmol) and anhydrous *c*-Hexane:$CHCl_3$ = 1:1 (4.0 mL) was added under Ar. The resulting mixture was stirred for 10 min at 0 ºC, and then *N*-Fmoc-2-aminobenzaldehyde (68.7 mg, 0.2 mmol) was added in one portion. After stirred for 48 h at 0 ºC, DDQ (31.8 mg, 0.14 mmol) was added and the mixture was stirred for additional 96 h. The resulting mixture was directly purified through flash column chromatography on silica gel (gradient elution with PE/EA) to give the pure product in 94% yield and 5% ee. The ee was determined by chiral stationary phase HPLC analysis [Daicel CHIRALCEL AD-3, hexane/isopropanol = 80/20, 1.0 mL/min, T = 25 °C, λ = 254 nm, $t_R$ (minor) = 12.6 min, $t_R$ (major) = 20.9 min]. $^1$H NMR (400 MHz, $CDCl_3$) δ 9.12 (brs, 1H), 8.74 (s, 1H), 8.36 (d, J = 7.9 Hz, 1H), 7.95 – 7.76 (m, 3H), 7.65 (t, J = 7.9 Hz, 2H), 7.54 – 7.49 (m, 1H), 7.43 (t, J = 7.7 Hz, 2H), 7.36 (d, J = 7.8 Hz, 1H), 7.30 (td, J = 7.2, 1.6 Hz, 2H), 7.24 (d, J = 7.2 Hz, 1H), 7.16 (d, J = 7.8 Hz, 1H), 7.11 (t, J = 7.5 Hz, 1H), 7.04 (t, J = 7.4 Hz, 1H), 6.81 (t, J = 7.6 Hz, 1H), 6.60 (t, J = 8.0 Hz, 1H), 6.55 (d, J = 8.0 Hz, 1H), 4.38 – 4.22 (m, 2H), 4.06 (s, 1H); $^{13}$C NMR (101 MHz, $CDCl_3$) δ 162.3, 154.0, 153.4, 146.7, 143.8, 143.5, 141.33, 141.28, 136.4, 135.5, 131.6, 131.1, 130.7, 129.0, 128.26, 128.25, 128.0, 127.82, 127.78, 127.6, 127.3, 127.10, 127.07, 125.2, 125.1, 122.7, 120.9, 120.0, 117.4, 99.7, 67.3, 47.0; HRMS (m/z): $[M]^+$ calcd for $C_{35}H_{25}O_4N_3I$ 678.0884; found 678.0872.

*Chiral HPLC spectrum of racemic **8c-1***

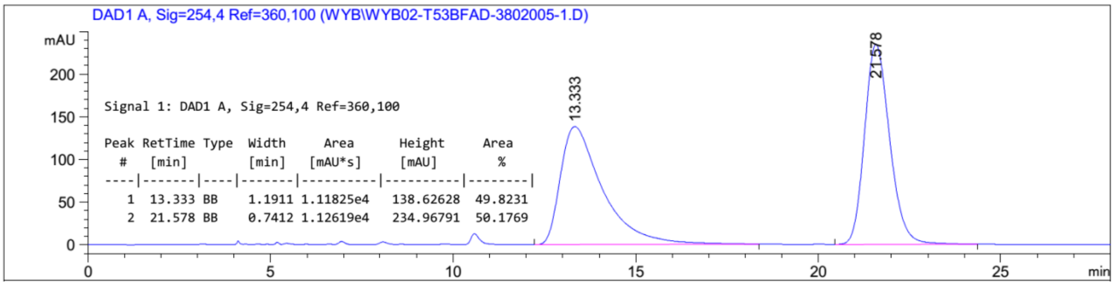


*Chiral HPLC spectrum of **8c-1***

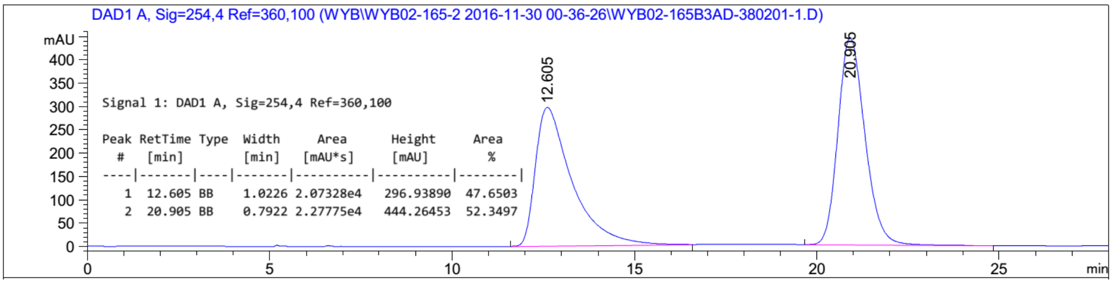

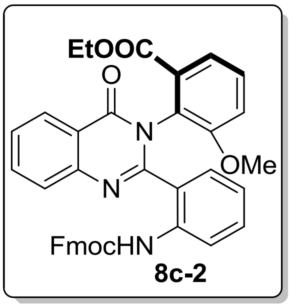


**(*aR*)-2-(2-((((9*H*-Fluoren-9-yl)methoxy)carbonyl)amino)phenyl)-3-(2-ethyloxycarbonyl -6-methoxyphenyl)quinazolin-4(3*H*)-one (8c-2)**

According to Procedure D in anhydrous c-Hexane:$CHCl_3$ = 1:1 (4.0 mL) at 25 °C for 96 h, **8c-1** was obtained in 91% yield and 44% ee when (**S**)-**CP3** was used as catalyst. The ee was determined by chiral stationary phase HPLC analysis [Daicel CHIRALCEL AD-3, hexane/isopropanol = 80/20, 0.5 mL/min, T = 25 °C, λ = 254 nm, $t_R$ (minor) = 40.4 min, $t_R$ (major) = 48.2 min]. $^1H$ NMR (400 MHz, $CDCl_3$) δ 8.94 (s, 1H), 8.38 (d, J = 7.9 Hz, 1H), 8.01 (brs, 1H), 7.90 – 7.85 (m, 2H), 7.73 (d, J = 7.6 Hz, 2H), 7.60 – 7.54 (m, 4H), 7.35 (ddd, J = 13.5, 9.0, 5.5 Hz, 3H), 7.24 – 7.14 (m, 4H), 6.92 (d, J = 8.3 Hz, 1H), 6.70 (t, J = 7.6 Hz, 1H), 4.45 (dd, J = 18.1, 10.6 Hz, 2H), 4.26 (t, J = 7.2 Hz, 1H), 4.23 – 4.12 (m, 2H), 3.51 (s, 3H), 1.11 (t, J = 7.1 Hz, 3H). $^{13}C$ NMR (101 MHz, $CDCl_3$) δ 165.2, 162.0, 154.5, 153.7, 153.4, 146.9, 143.8, 143.7, 141.4, 136.3, 135.0, 130.6, 130.0, 129.9, 128.7, 127.9, 127.5, 127.3, 127.2, 127.1, 126.3, 125.2, 123.3, 122.8, 121.9, 121.2, 120.5, 120.1, 115.5, 67.1, 61.6, 55.9, 47.1, 13.9; HRMS (m/z): $[M]^+$ calcd for $C_{39}H_{32}O_6N_3$ 638.2286; found 638.2274.

*Chiral HPLC spectrum of racemic **8c-2***

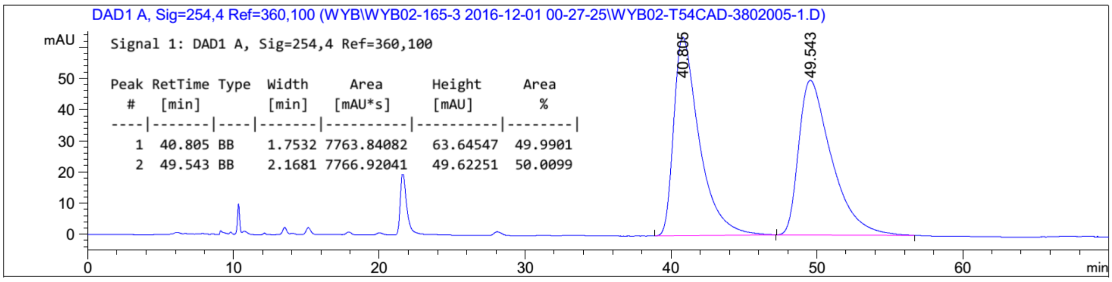


*Chiral HPLC spectrum of **8c-2***

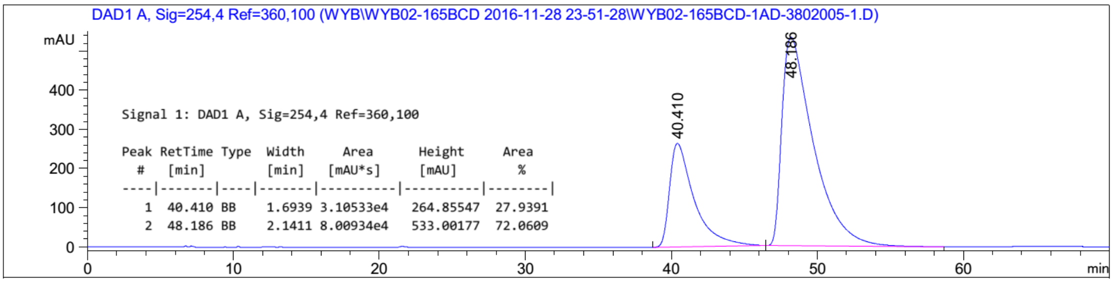

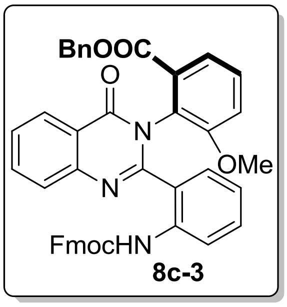


**(*aR*)-3-(2-Benzyloxycarbonyl -6-methoxyphenyl)-2-(2-((((9*H*-fluoren-9-yl)methoxy)carbonyl)amino)phenyl)quinazolin-4(3*H*)-one (8c-3)**

According to Procedure D in anhydrous *c*-Hexane:$CHCl_3$ = 1:1 (4.0 mL) at 25 °C for 96 h, **8c-1** was obtained in 67% yield and 39% ee when (**S**)-**CP3** was used as catalyst. The ee was determined by chiral stationary phase HPLC analysis [Daicel CHIRALCEL AD-3, hexane/isopropanol = 80/20, 0.5 mL/min, T = 25 °C, λ = 254 nm, $t_R$ (minor) = 55.3 min, $t_R$ (major) = 71.9 min]. $^1$H NMR (400 MHz, $CDCl_3$) δ 8.92 (s, 1H), 8.31 (d, J = 7.8 Hz, 1H), 8.00 (brs, 1H), 7.86 (t, J = 7.5 Hz, 1H), 7.81 (d, J = 7.8 Hz, 1H), 7.72 (d, J = 7.6 Hz, 2H), 7.61 (d, J = 7.8 Hz, 1H), 7.58 – 7.53 (m, 3H), 7.33 (ddd, J = 18.7, 11.7, 5.8 Hz, 3H), 7.24 – 7.12 (m, 9H), 6.90 (d, J = 8.3 Hz, 1H), 6.68 (t, J = 7.6 Hz, 1H), 5.22 (d, J = 12.2 Hz, 1H), 5.12 (d, J = 12.2 Hz, 1H), 4.48 – 4.37 (m, 2H), 4.25 (t, J = 7.2 Hz, 1H), 3.47 (s, 3H). $^{13}$C NMR (101 MHz, $CDCl_3$) δ 165.1, 161.9, 154.4, 153.5, 153.4, 146.8, 143.8, 143.7, 141.4, 141.4, 136.3, 135.0, 134.9, 130.6, 130.0, 129.8, 128.7, 128.5, 128.4, 128.4, 127.9, 127.6, 127.4, 127.3, 127.2, 127.1, 126.3, 125.2, 123.3, 122.9, 121.9, 121.1, 120.6, 120.1, 115.7, 67.5, 67.1, 55.9, 47.1; HRMS (m/z): $[M]^+$ calcd for $C_{44}H_{34}O_6N_3$ 700.2442; found 700.2425.

*Chiral HPLC spectrum of racemic* ***8c-3***

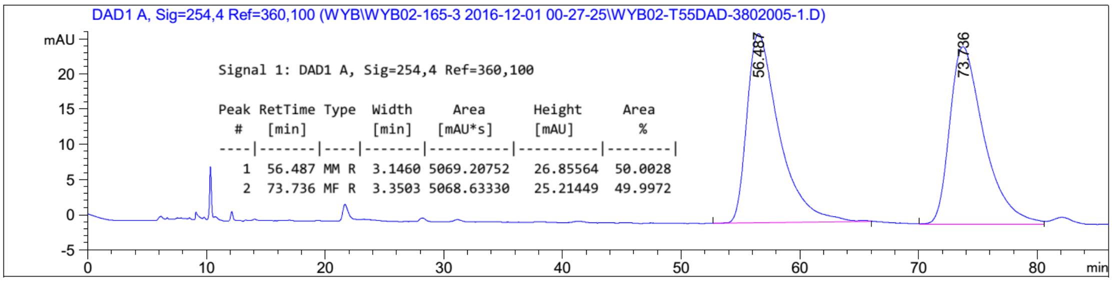


*Chiral HPLC spectrum of* ***8c-3***

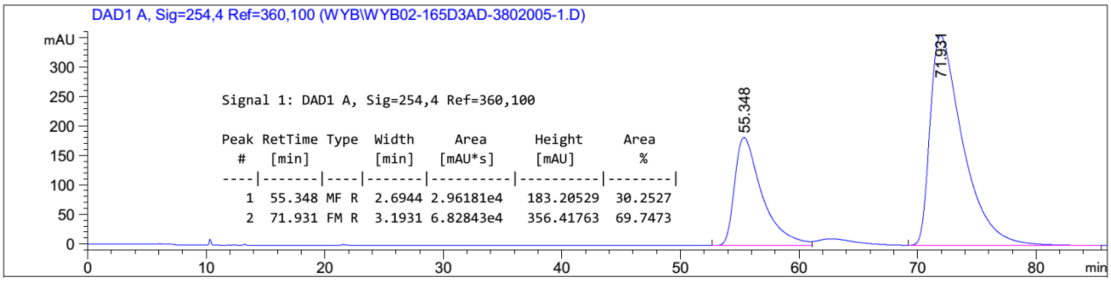

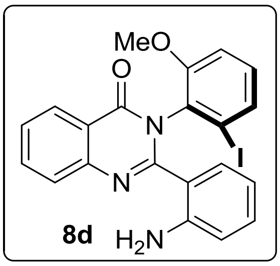


**(*aS*)-2-(2-Aminophenyl)-3-(2-iodo-6-methoxyphenyl)quinazolin-4(*3H*)-one (8d)**

**8d** (95% ee, 120 mg, 0.17 mmol) was added to 6 mL DMF, and then piperidine (3 mL) was added to the solution. The reaction became yellow rapidly and the solution was stirred for 2 h at room temperature. The resulting solution was diluted with water (30 mL) and the mixture was extracted with EA (3 x 20 mL). The combined organic layer was washed with water (4 x 20 mL) and bine (20 mL), dried over $Na_2SO_4$ and concentrated under reduced pressure. The crude product was purified through flash chromatography on silica gel to yield the corresponding product **8d** (91.1% yield, 95% ee, 74.2 mg). The *racemic* **8d** was synthesized according to the above procedure using *racemic* **8c**.The ee was determined by chiral stationary phase HPLC analysis [Daicel CHIRALCEL AD-3, hexane/isopropanol = 80/20, 1.0 mL/min, T = 25 °C, λ = 254 nm, $t_R$ (minor) = 19.2 min, $t_R$ (major) = 22.1 min]. $^1$H NMR (400 MHz, DMSO-$d_6$) δ 8.20 (dd, J = 8.0, 1.2 Hz, 1H), 7.92 (ddd, J = 8.5, 7.3, 1.5 Hz, 1H), 7.77 (d, J = 7.7 Hz, 1H), 7.63-7.59 (m, 1H), 7.42 (dd, J = 7.7, 1.3 Hz, 1H), 7.06 (d, J = 8.3 Hz, 1H), 7.01 (dd, J = 8.4, 1.2 Hz, 1H), 6.96-6.92 (m, 1H), 6.86 (dd, J = 7.8, 1.4 Hz, 1H), 6.69 (dd, J = 8.2, 0.6 Hz, 1H), 6.24-6.20 (m, 1H), 5.46 (s, 2H), 3.64 (s, 3H); $^{13}$C NMR (101 MHz, DMSO-$d_6$) δ 160.6, 155.6, 154.1, 147.1, 146.9, 135.1, 131.4, 130.3, 130.3, 129.5, 128.2, 127.35, 127.31, 126.5, 120.5, 116.8, 115.8, 114.3, 112.0, 102.1, 56.1; HRMS (m/z): $[M]^+$ calcd for $C_{21}H_{17}O_2N_3$ 470.0360; found 470.0356.

*Chiral HPLC spectrum of racemic **8d***

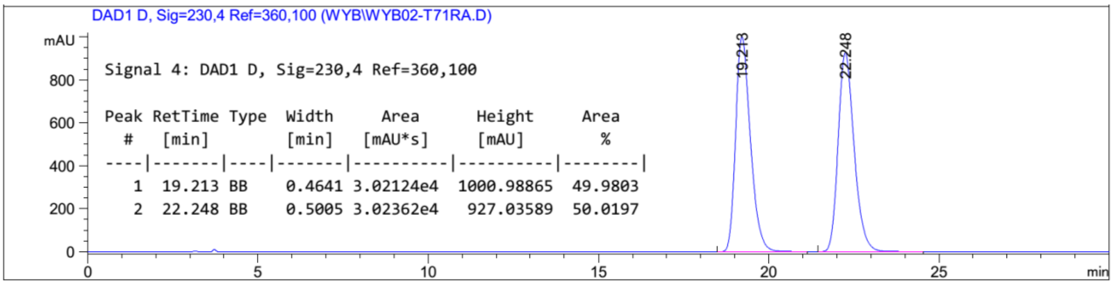


*Chiral HPLC spectrum of **8d***

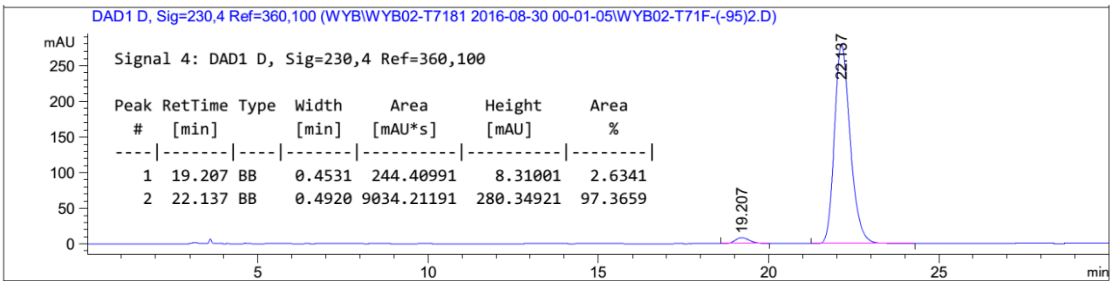

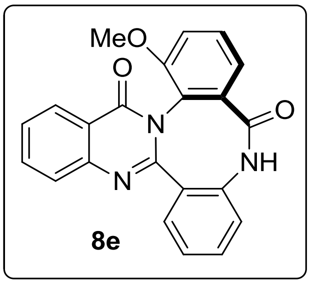


**(*aS*)-15-Methoxy-10H-dibenzo[3,4:7,8][1,5]diazocino[2,1-b]quinazoline-11,17-dione (8e)**

To a 25 mL dry Schlenk tube, **8d** (95%ee, 46.9 mg, 0.1 mmol), Pddba$_2$ (8.6 mg, 0.015 mmol), 1,3-bis(diphenyl-phosphino) propane (8.25 mg, 0.020 mmol), tetrabutylammonium chloride hydrate (77.9 mg, 0.3 mmol), DIPEA (49.6 μL, 0.3 mmol) and 2.0 mL degased toluene, was added under Ar. The mixture was cooled to -78 ºC, evacuated, backfilled with CO (1 atm), evacuation and backfilling repeated for 4 times. After sealed the tube, the mixture was warmed to room temperature and then heated to 85 ºC. After stirred for 72 h, the mixture was diluted with $CH_3CN$ and concentrated under reduced pressure. The residue was purified through flash chromatography on silica gel to yield the corresponding product **8e** in 95% yield and 95% ee as a white solid. The *racemic* **8e** was synthesized according to the above procedure using *racemic* **8d**. The ee was determined by chiral stationary phase HPLC analysis [Daicel CHIRALCEL AD-3, hexane/isopropanol = 80/20, 1.0 mL/min, T = 25 °C, λ = 254 nm, $t_R$ (minor) = 20.7 min, $t_R$ (major) = 27.7 min]. $^1$H NMR (400 MHz, DMSO-$d_6$) δ 10.22 (s, 1H), 8.21 (dd, J = 7.9, 1.3 Hz, 1H), 7.95 (ddd, J = 8.5, 7.3, 1.5 Hz, 1H), 7.78 (d, J = 7.8 Hz, 1H), 7.67-7.63 (m, 1H), 7.52 (dd, J = 7.6, 1.5 Hz, 1H), 7.43 (td, J = 7.9, 1.9 Hz, 2H), 7.33 (td, J = 7.5, 1.0 Hz, 1H), 7.16-7.13 (m, 1H), 6.98 (dd, J = 7.7, 1.1 Hz, 1H), 3.67 (s, 3H); $^{13}$C NMR (101 MHz, DMSO-$d_6$) δ 168.3, 159.8, 153.42, 153.38, 147.1, 136.5, 135.4, 134.5, 133.5, 131.5, 131.2, 128.7, 127.9, 127.6, 127.5, 126.6, 126.4, 122.6, 120.1, 118.4, 113.8, 56.4; HRMS (m/z): $[M]^+$ calcd for $C_{22}H_{16}O_3N_3$ 370.1186; found 370.1181.

*Chiral HPLC spectrum of racemic **8e***

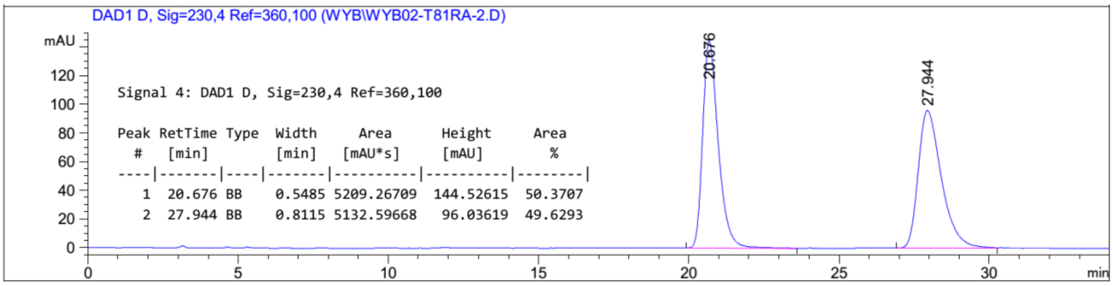


*Chiral HPLC spectrum of **8e***

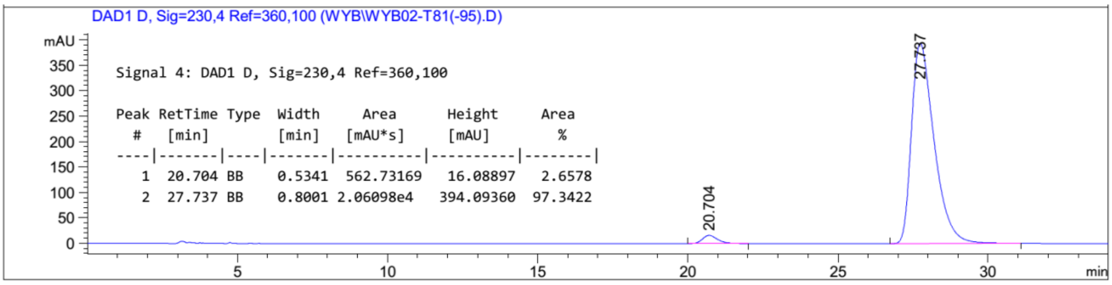

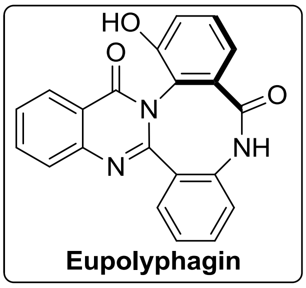


**(*aS*)-15-Hydroxy-10*H*-dibenzo[3,4:7,8][1,5]diazocino[2,1-b]quinazoline-11,17-dione (Eupolyphagin)**

To a 10 mL dry Schlenk tube, **8e** (30 mg, 0.81 mmol) and 4.0 mL anhydrous DCM was added under Ar, $BBr_3$ (56 μL, 4.86 mmol) was added slowly and the mixture was stirred for 24 h at room temperature. The solution was slowly added to 10 mL saturated $NaHCO_3$ solution and the resulting mixture was acidified with 1M HCl. The aqueous phase was extracted with EA (3 x 15 mL), the combined organic layer was dried over $Na_2SO_4$ and concentrated under reduced pressure. The residual white solid was purified through flash chromatography on silica gel to yield corresponding natural product **Eupolyphagin** in 98% yield and 95% ee as a white solid. The *racemic* **Eupolyphagin** was synthesized according to the above method using *racemic* **8e**. The ee was determined by chiral stationary phase HPLC analysis [Daicel CHIRALCEL AD-3, hexane/isopropanol = 80/20, 1.0 mL/min, T = 25 °C, λ = 254 nm, $t_R$ (major) = 11.1 min, $t_R$ (minor) = 17.8 min]. $^1$H NMR (400 MHz, $CD_3OD$) δ 8.28 (dd, J = 8.0, 1.1 Hz, 1H), 7.91 (ddd, J = 8.5, 7.3, 1.5 Hz, 1H), 7.78 (d, J = 7.9 Hz, 1H), 7.65-7.61 (m, 1H), 7.61-7.59 (m, 1H), 7.45 (td, J = 7.7, 1.6 Hz, 1H), 7.37 (td, J = 7.6, 1.1 Hz, 1H), 7.27 (t, J = 7.7 Hz, 1H), 7.16 (dd, J = 7.9, 0.8 Hz, 1H), 6.91 (dd, J = 5.2, 1.2 Hz, 1H), 6.89 (dd, J = 4.5, 1.2 Hz, 1H); $^{13}$C NMR (126 MHz, $CD_3OD$) δ 172.2, 162.4, 155.9, 153.8, 148.7, 137.8, 136.5, 135.7, 135.2, 132.6, 132.4, 130.0, 129.12, 129.05, 128.3, 128.0, 127.6, 123.1, 121.9, 119.1, 118.6; HRMS (m/z): $[M]^+$ calcd for $C_{21}H_{14}O_3N_3$ 356.1030; found 356.1017; $[\alpha]_D^{24}$ +67.5° (ref. +50.8° [12]) (c 0.1, MeOH).

*Chiral HPLC spectrum of racemic **Eupolyphagin***

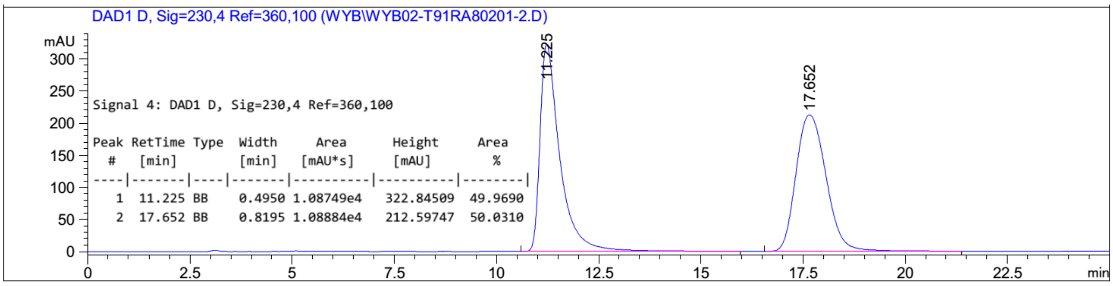


*Chiral HPLC spectrum of* **Eupolyphagin**

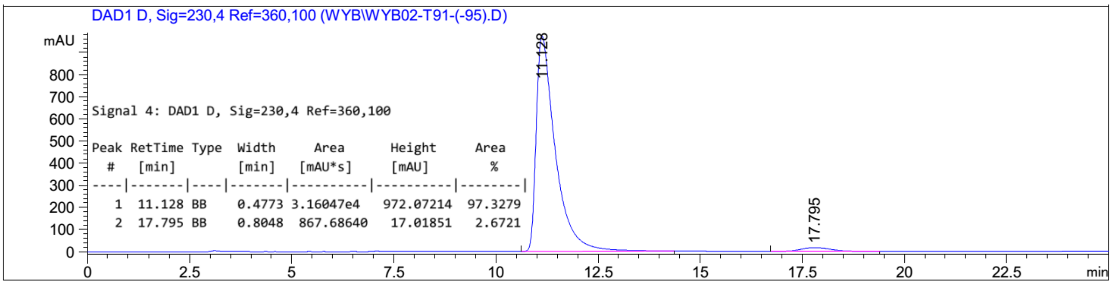

# Supplementary Note 5

## Experimental procedure for the reaction mechanism.

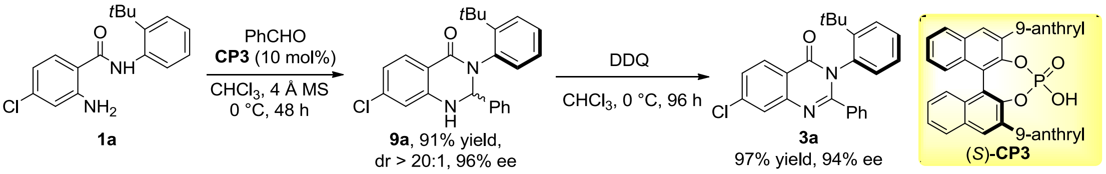


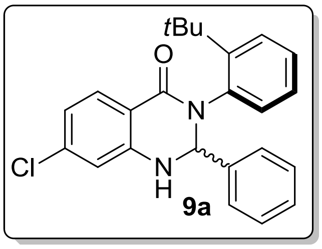


**($aR$)-3-(2-(*tert*-Butyl)phenyl)-7-chloro-2-phenyl-2,3-dihydroquinazolin-4(1H)-one (9a).**
To a dry Schlenk tube (10 mL), 400 mg activated 4 Å MS (molecular sieves) was added and then the MS was reactivated under reduced pressure for 15 min. After the tube was cooled down, **1a** (30.3 mg, 0.10 mmol), **CP3** (7.0 mg, 0.01 mmol) and anhydrous $CHCl_3$ (4.0 mL) was added under Ar. The resulting mixture was stirred for 10 min at 0 ºC, and then benzaldehyde (21.2 mg, 0.2 mmol) was added in one portion. After stirred for 48 h at 0 ºC, the resulting mixture was directly purified through flash column chromatography on silica gel (gradient elution with PE/EA) to give the pure product in 90% yield as a light brown solid. The ee was determined by chiral stationary phase HPLC analysis [Daicel CHIRALCEL OD-3, hexane/isopropanol = 90/10, 1.0 mL/min, T = 25 °C, λ = 254 nm, $t_R$ (major) = 6.3 min, $t_R$ (minor) = 9.1 min]. $^1H$ NMR (400 MHz, $CDCl_3$) δ 7.90 (d, J = 8.2 Hz, 1H), 7.54 (d, J = 7.8 Hz, 1H), 7.35 – 7.23 (m, 6H), 6.92 (t, J = 7.2 Hz, 1H), 6.79 (d, J = 8.0 Hz, 1H), 6.51 (s, 1H), 6.42 (d, J = 7.6 Hz, 1H), 5.66 (s, 1H), 5.10 (s, 1H), 1.46 (s, 9H); $^{13}C$ NMR (101 MHz, $CDCl_3$) δ 163.1, 147.1, 146.4, 140.7, 140.0, 138.0, 132.7, 130.5, 129.5, 129.1, 128.8, 128.6, 126.6, 126.5, 119.3, 114.2, 76.6, 35.8, 31.7; HRMS (m/z): $[M]^+$ calcd for $C_{24}H_{24}ON_2Cl$ 391.1572; found 391.1565.

To a dry Schlenk tube (10 mL), **9a** (96% ee, 39.1 mg, 0.10 mmol) and anhydrous $CHCl_3$ (4.0 mL) was added under Ar. The resulting mixture was stirred for 10 min at 0 ºC, and then DDQ (31.8 mg, 0.14 mmol) was added in one portion. After stirred for 96 h at 0 ºC, the resulting mixture was directly purified through flash column chromatography on silica gel (gradient elution with PE/EA) to give the pure product **3a** in 92% yield and 94% ee as a white solid.

*Chiral HPLC spectrum of racemic **9a***

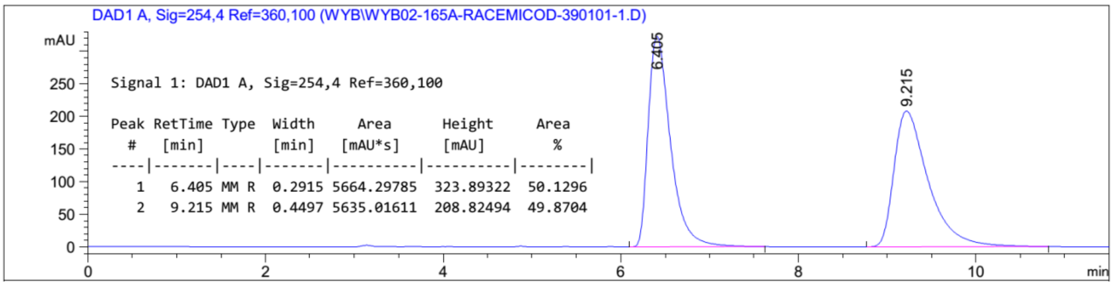

*Chiral HPLC spectrum of* ***9a***

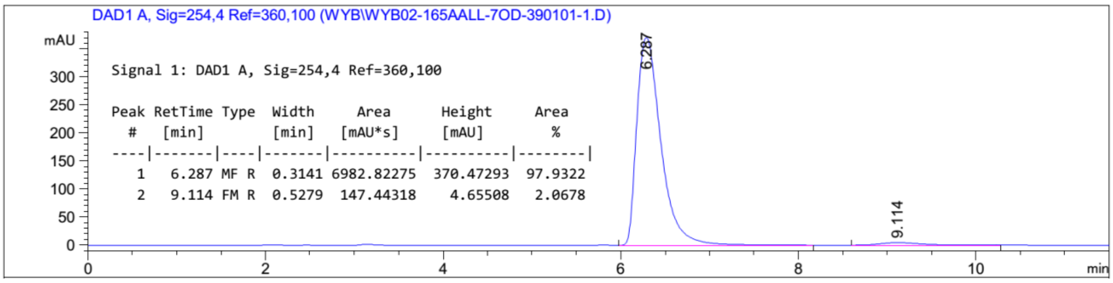


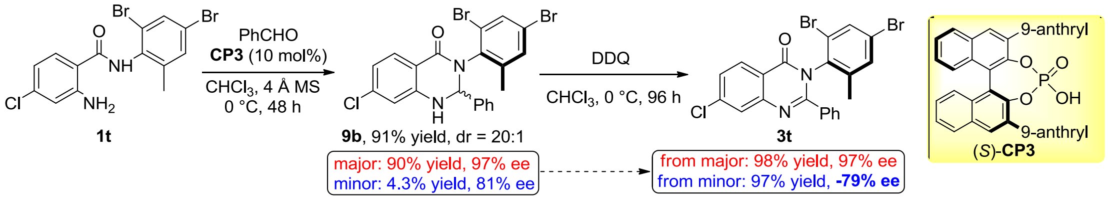


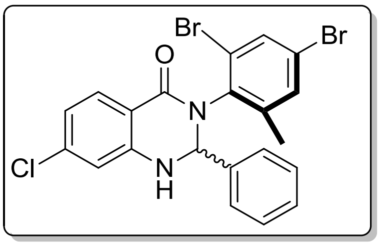

**(a*R*)-3-(2,4-Dibromo -6-methylphenyl)-7-chloro-2-phenyl-2,3-dihydroquinazolin-4(1H)-one ((*R*)-9b)**

To a dry Schlenk tube (10 mL), 400 mg activated 4 Å MS (molecular sieves) was added and then the MS was reactivated under reduced pressure for 15 min. After the tube was cooled down, **1t** (41.9 mg, 0.10 mmol), **CP3** (7.0 mg, 0.01 mmol) and anhydrous $CHCl_3$ (4.0 mL) was added under Ar. The resulting mixture was stirred for 10 min at 0 ºC, and then benzaldehyde (21.2 mg, 0.2 mmol) was added in one portion. After stirred for 48 h at 0 ºC, the resulting mixture (dr = 20:1 detected by H NMR) was directly purified through flash column chromatography on silica gel (gradient elution with PE/EA) to give the pure product (*R*)-**9b** in 90% yield and 97% ee and (*S*)-**9b** in 4.3% yield and 81% ee.

The ee of (*R*)-**9b** was determined by chiral stationary phase HPLC analysis [Daicel CHIRALCEL AD-3, hexane/isopropanol = 80/20, 0.5 mL/min, T = 25 °C, λ = 254 nm, $t_R$ (major) = 28.8 min, $t_R$ (minor) = 32.3 min]. $^1$H NMR (500 MHz, $CDCl_3$) δ 7.96 (d, J = 8.4 Hz, 1H), 7.50 – 7.48 (m, 1H), 7.43 (d, J = 2.1 Hz, 1H), 7.34 – 7.30 (m, 1H), 7.26 – 7.23 (m, 3H), 7.21 (d, J = 1.9 Hz, 1H), 6.92 (dd, J = 8.4, 1.8 Hz, 1H), 6.76 (d, J = 1.8 Hz, 1H), 6.10 (s, 1H), 4.73 (s, 1H), 2.36 (s, 3H); $^{13}$C NMR (126 MHz, $CDCl_3$) δ 161.4, 148.1, 142.3, 140.2, 136.8, 136.7, 133.3, 132.8, 131.0, 130.3, 128.7, 127.5, 124.4, 122.1, 120.1, 114.2, 114.0, 73.8, 19.1; HRMS (m/z): $[M]^+$ calcd for $C_{21}H_{16}ON_2BrCl$ 504.9312; found 504.9305.

*Chiral HPLC spectrum of racemic* (*R*)-**9b**

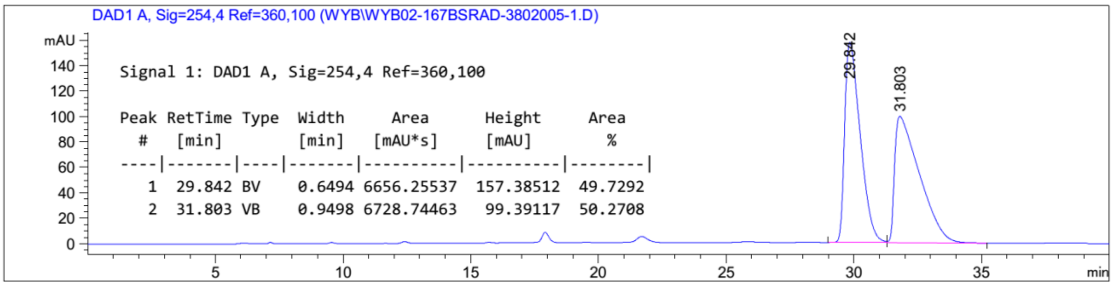


*Chiral HPLC spectrum of (R)-**9b***

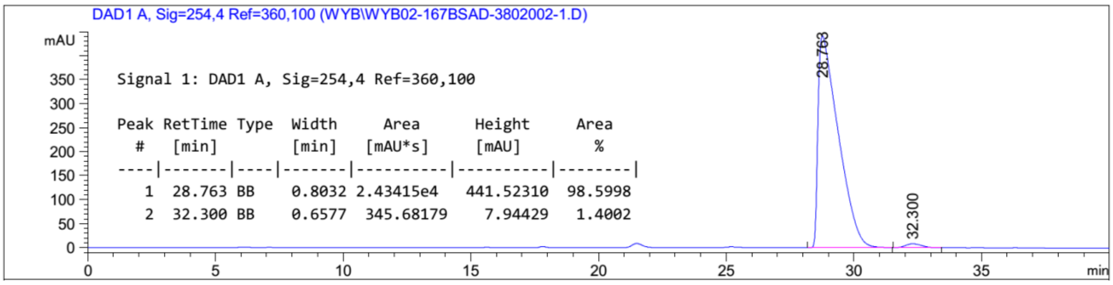


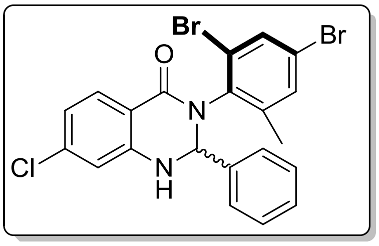


**(*aS*)-3-(2,4-Dibromo-6-methylphenyl)-7-chloro-2-phenyl-2,3-dihydroquinazolin-4(1H)-one ((*S*)-9b)**

The ee of **9bS** was determined by chiral stationary phase HPLC analysis [Daicel CHIRALCEL AD-3, hexane/isopropanol = 80/20, 0.5 mL/min, T = 25 °C, λ = 254 nm, $t_R$ (major) = 25.0 min, $t_R$ (minor) = 32.0 min]. $^1$H NMR (500 MHz, $CDCl_3$) δ 7.96 (d, J = 8.4 Hz, 1H), 7.50 – 7.48 (m, 1H), 7.43 (d, J = 2.1 Hz, 1H), 7.34 – 7.30 (m, 1H), 7.26 – 7.23 (m, 2H), 7.21 (d, J = 1.9 Hz, 1H), 6.92 (dd, J = 8.4, 1.8 Hz, 1H), 6.76 (d, J = 1.8 Hz, 1H), 6.10 (s, 1H), 4.73 (s, 1H), 2.36 (s, 3H); $^{13}$C NMR (126 MHz, $CDCl_3$) δ 161.6, 148.1, 140.2, 140.2, 137.0, 135.8, 133.4, 133.0, 131.3, 130.5, 128.7, 128.0, 126.7, 122.0, 120.4, 114.6, 114.4, 74.8, 19.7; HRMS (m/z): $[M]^+$ calcd for $C_{21}H_{16}ON_2BrCl$ 504.9312; found 504.9297.

*Chiral HPLC spectrum of racemic (S)-**9b***

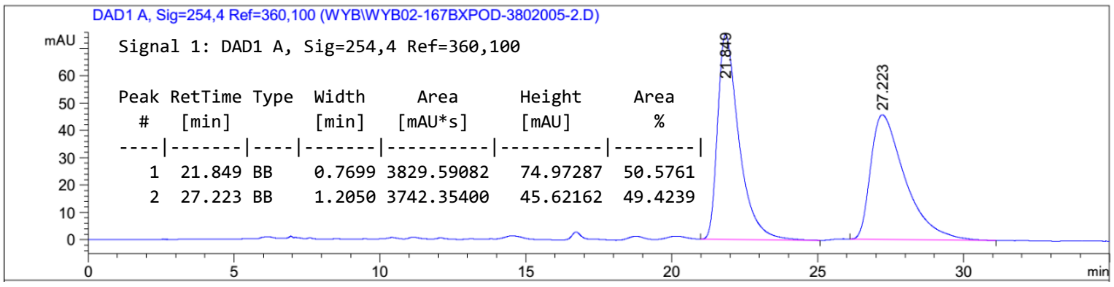


*Chiral HPLC spectrum of (S)-**9b***

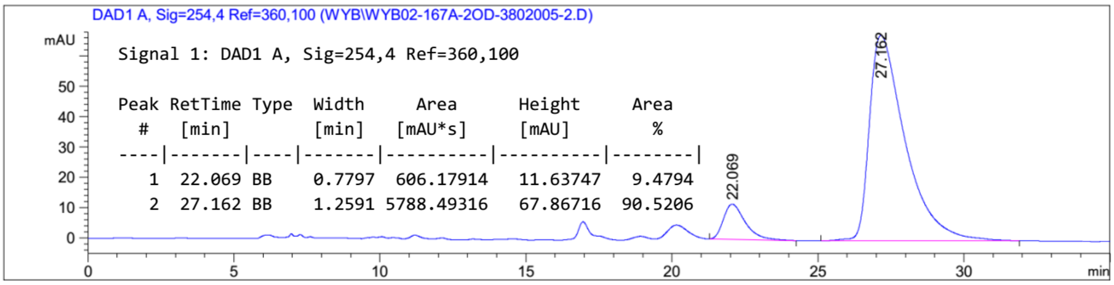


To a dry Schlenk tube (10 mL), (*R*)-**9b** (96% ee, 39.1 mg, 0.10 mmol) and anhydrous $CHCl_3$ (4.0 mL) was added under Ar. The resulting mixture was stirred for 10 min at 0 ºC, and then DDQ (31.8 mg, 0.14 mmol) was added in one portion. After stirred for 96 h at 0 ºC, the resulting mixture was directly purified through flash column chromatography on silica gel (gradient elution with PE/EA) to give the pure product **3t** in 98% yield and 97% ee as a white solid. According to the above mentioned process, (*S*)-**9b** afforded **3t** in 97% yield and -79%ee.

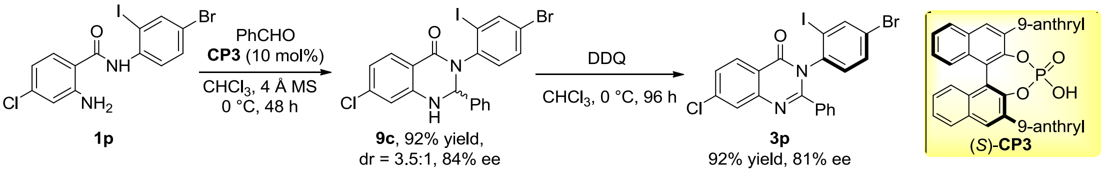


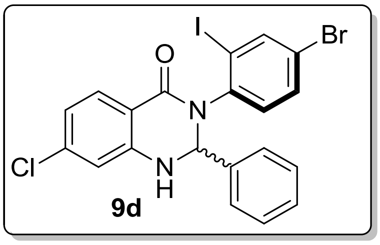


**(*aR*)-3-(4-Bromo -2-idodophenyl)-7-chloro-2-phenyl-2,3-dihydroquinazolin-4(1H)-one (9d)**

To a dry Schlenk tube (10 mL), 400 mg activated 4 Å MS (molecular sieves) was added and then the MS was reactivated under reduced pressure for 15 min. After the tube was cooled down, **1a** (30.3 mg, 0.10 mmol), **CP3** (7.0 mg, 0.01 mmol) and anhydrous $CHCl_3$ (4.0 mL) was added under Ar. The resulting mixture was stirred for 10 min at 0 ºC, and then benzaldehyde (21.2 mg, 0.2 mmol) was added in one portion. After stirred for 48 h at 0 ºC, the resulting mixture was directly purified through flash column chromatography on silica gel (gradient elution with PE/EA) to give the pure product **9d** (dr = 3.5:1 in CDCl3 or 1.6:1 in DMSO-$d_6$ detected by H NMR) in 92% yield and 84% ee. The ee was determined by chiral stationary phase HPLC analysis [Daicel CHIRALCEL OD-3, hexane/isopropanol = 80/20, 0.5 mL/min, T = 25 °C, λ = 254 nm, $t_R$ (major) = 28.8 min, $t_R$ (minor) = 32.3 min]. $^1$H NMR (500 MHz, DMSO) δ 8.14 (d, J = 2.2 Hz, $1H_{major}$), 7.86 (d, J = 1.8 Hz, $1H_{major}$), 7.84 (d, J = 2.2 Hz, $1H_{minor}$), 7.74 (d, J = 8.4 Hz, $1H_{minor}$), 7.72 (d, J = 8.3 Hz, $1H_{major}$), 7.63 (s, $1H_{minor}$), 7.57 (d, J = 3.7 Hz, $1H_{minor}$), 7.56 (d, J = 2.1 Hz, $1H_{minor}$), 7.46 – 7.42 (m, $1H_{major+minor}$), 7.39 – 7.37 (m, $2H_{major}$+$1H_{minor}$), 7.34 – 7.32 (m, $3H_{major}$), 7.28 – 7.26 (m, $3H_{minor}$), 6.90 (d, J = 1.9 Hz, $1H_{minor}$), 6.82 (dd, J = 8.4, 2.0 Hz, $1H_{minor}$), 6.79 (d, J = 1.9 Hz, $1H_{major}$), 6.76 (dd, J = 8.3, 2.0 Hz, $1H_{major}$), 6.66 (d, J = 8.4 Hz, $1H_{major}$), 6.54 (s, $1H_{minor}$), 6.06 (d, J = 1.9 Hz, $1H_{major}$); $^{13}$C NMR (126 MHz, DMSO) δ 161.9, 160.7, 149.4, 148.0, 141.9, 141.8, 141.0, 140.1, 139.5, 138.7, 138.5, 136.7, 132.7, 131.6, 131.3, 130.7, 130.3, 130.0, 129.5, 129.20, 129.18, 129.1, 128.8, 128.0, 126.7, 121.5, 120.2, 117.8, 117.3, 113.8, 113.5, 112.7,

104.4, 102.0, 74.1, 73.1; HRMS (m/z): $[M]^+$ calcd for $C_{20}H_{14}ON_2BrClI$ 538.9017; found 538.9012.

*Chiral HPLC spectrum of racemic* ***9bR***

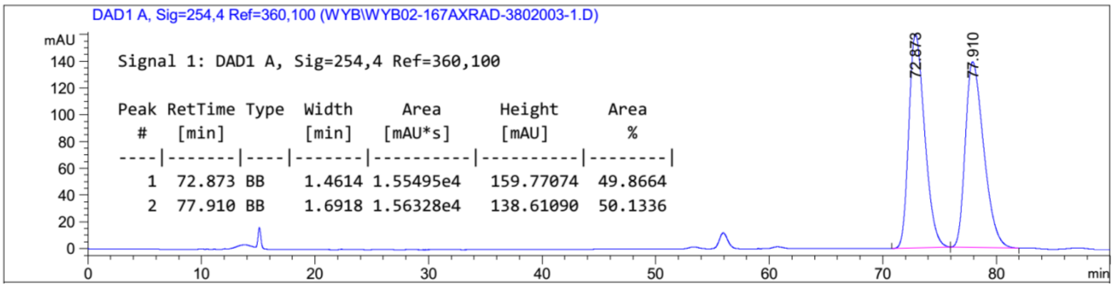


*Chiral HPLC spectrum of* ***9bR***

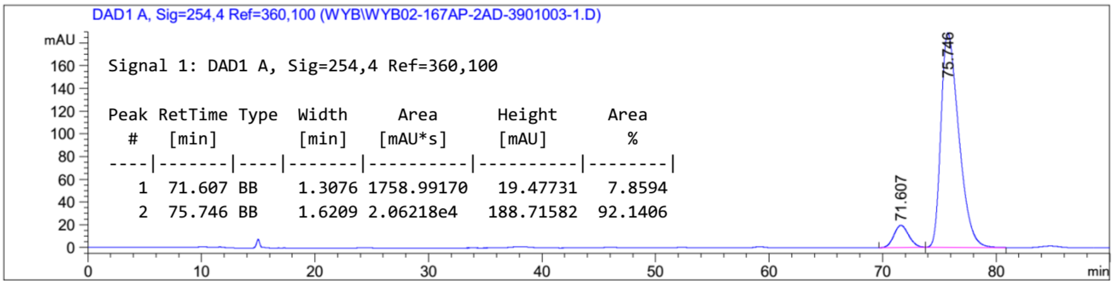


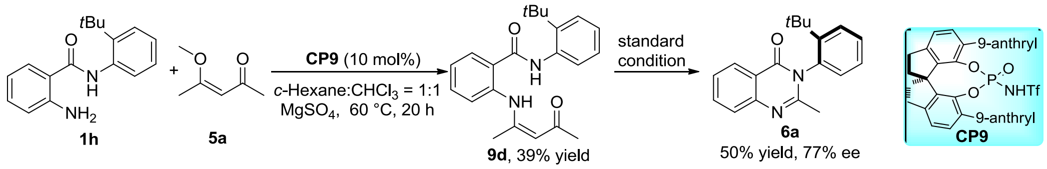


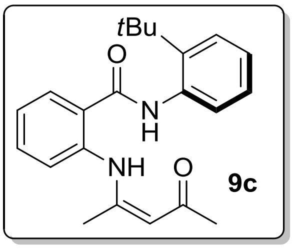


**N-(2-(*tert*-butyl)phenyl)-2-((4-oxopent-2-en-2-yl)amino)benzamide (9c)**

To a 10 mL dry Schlenk tube, **1h** (0.10 mmol), **5a**[4] (22.8 mg, 0.2 mmol), $MgSO_4$ (6.0 mg), **CP9** (8.0 mg, 0.01 mmol) and anhydrous *c*-Hexane/$CHCl_3$ = 1:1 (4.0 mL) was added under Ar. The resulting solution was heated to 60 ºC. After stirred for 20 h, the solution was cooled down to room temperature and then directly purified through flash column chromatography on silica gel (gradient elution with PE/EA/TEA) to give the pure product in 39% yield as a colorless oil. $^1$H NMR (400 MHz, $CDCl_3$) δ 12.40 (s, 1H), 8.26 (s, 1H), 7.88 (d, J = 7.4 Hz, 1H), 7.53 (d, J = 7.2 Hz, 1H), 7.46 (t, J = 7.2 Hz, 1H), 7.41 (dd, J = 7.8, 1.6 Hz, 1H), 7.37 (t, J = 7.4 Hz, 1H), 7.25 – 7.17 (m, 2H), 7.11 (d, J = 7.8 Hz, 1H), 5.27 (s, 1H), 1.97 (s, 3H), 1.91 (s, 3H), 1.35 (s, 9H); $^{13}$C NMR (101 MHz, $CDCl_3$) δ 196.9, 165.7, 160.7, 144.0, 136.6, 135.0, 133.1, 131.4, 129.5, 129.1, 128.1, 127.1, 127.0, 126.8, 126.7, 99.3, 34.7, 30.8, 29.2, 19.8; HRMS (m/z): $[M]^+$ calcd for $C_{22}H_{27}O_2N_2$ 351.2067; found 351.2060.

To a 10 mL dry Schlenk tube, **9c** (0.10 mmol), $MgSO_4$ (6.0 mg), **CP9** (8.0 mg, 0.01 mmol)

and anhydrous *c*-Hexane/$CHCl_3$ = 1/1 (4.0 mL) was added under Ar. The resulting solution was heated to 60 ºC. After stirred for 96 h, the solution was cooled down to room temperature and then directly purified through flash column chromatography on silica gel (gradient elution with PE/EA/TEA) to give the pure product in 50% yield and 77% ee.

# Supplementary References


1. Li, Z. *et al.* Metal- and oxidant-free synthesis of quinazolinones from β-ketoesters with o-aminobenzamides via phosphorous acid-catalyzed cyclocondensation and selective C–C bond cleavage. *J. Org. Chem.* **80**, 9392-9400, (2015).
2. Shen, G. *et al.* Brønsted acid-catalyzed selective C-C bond cleavage of 1,3-diketones: a facile synthesis of 4(*3H*)-quinazolinones in aqueous ethyl lactate. *RSC Advances* **5**, 85646-85651, (2015).
3. Shen, G., Zhou, H., Sui, Y., Liu, Q. & Zou, K. $FeCl_3$-catalyzed tandem condensation/intramolecular nucleophilic addition/C–C bond cleavage: a concise synthesis of 2-substitued quinazolinones from 2-aminobenzamides and 1,3-diketones in aqueous media. *Tetrahedron Lett.* **57**, 587-590, (2016).
4. Chizhov, D. L. *et al.* Trialkyl borate assisted amination of fluorinated 1,3-diketones for synthesis of *N,N′*-1,2-phenylen-bis(β-aminoenones) and their Ni(II), Cu(II) and Pd(II) complexes. *J. Fluorine Chem.* **132**, 394-401, (2011).
5. Congiu, C., Cocco, M. T., Lilliu, V. & Onnis, V. New potential anticancer agents based on the anthranilic acid scaffold. synthesis and evaluation of biological activity. *J. Med. Chem.* **48**, 8245-8252, (2005).
6. Quesnel, J. S. & Arndtsen, B. A. A palladium-catalyzed carbonylation approach to acid chloride synthesis. *J. Am. Chem. Soc.* **135**, 16841-16844, (2013).
7. Stork, G. & Kraus, G. A. A new synthesis of vinylogous aldols and polyenones. *J. Am. Chem. Soc.* **98**, 2351-2352, (1976).
8. Hermecz, I., Szilágyi, I., Orfi, L., Kökösi, J. & Szász, G. Nitrogen bridgehead compounds. Part 85. Synthesis and reactivity of 3,4-dihydro-*1H,6H*[1,4]oxazino[3,4-b]quinazolin-6-ones. *J. Heterocycl. Chem.* **30**, 1413-1420, (1993).
9. Rathman, T. L., Sleevi, M. C., Krafft, M. E. & Wolfe, J. F. Functionalization of 2-methyl-3-o-tolyl-4(*3H*)-quinazolinone and related compounds through carbanion reactions at the 2-methyl group. *J. Org. Chem.* **45**, 2169-2176, (1980).
10. Welch, W. M. *et al.* Atropisomeric quinazolin-4-one derivatives are potent noncompetitive α-amino-3-hydroxy-5-methyl-4-isoxazolepropionic acid (AMPA) receptor antagonists. *Bioorg. Med. Chem. Lett.* **11**, 177-181, (2001).
11. Nakamura, I., Yamagishi, U., Song, D., Konta, S. & Yamamoto, Y. Gold- and indium-catalyzed synthesis of 3- and 6-sulfonylindoles from ortho-alkynyl-*N*-sulfonylanilines. *Angew. Chem. Int. Ed.* **46**, 2284-2287, (2007).
12. Jiang, H.-L. *et al.* New isocoumarins and alkaloid from Chinese insect medicine, *Eupolyphaga sinensis* walker. *Fitoterapia* **83**, 1275-1280, (2012).
